# Catalytic Oxidative Ligand Transfer of Rh-Carbynoids and Alkyl Iodides

**DOI:** 10.1021/jacs.5c09559

**Published:** 2025-08-21

**Authors:** Josep Esteve Guasch, Debasis Pal, Marcos G. Suero

**Affiliations:** ‡ 202569 Institute of Chemical Research of Catalonia (ICIQ-CERCA), The Barcelona Institute of Science and Technology, Països Catalans 16, 43007 Tarragona, Spain; ∧ ICREA, Pg. Lluis Companys 23, 08010 Barcelona, Spain; † Departament de Química Analítica i Química Orgánica, Universitat Rovira i Virgili, Calle Marcel·lí Domingo, 1, 43007 Tarragona, Spain

## Abstract

Herein, we disclose the first catalytic generation of alkyl–I^(III)^ Rh^(II)^-carbynoids via oxidative ligand transfer between alkyl iodides and aryl–I^(III)^ Rh^(II)^-carbynoids. When homoallylic iodides were used, alkyl–I^(III)^ Rh^(II)^-carbynoids evolved through a diastereoselective cyclopropanation that led to highly electrophilic bicyclic alkyl–I^(III)^ species. We observed that the latter species reacted with a plethora of nucleophiles, enabling access to a valuable class of cyclopropanes that were converted to housanes.

## Graphical abstract

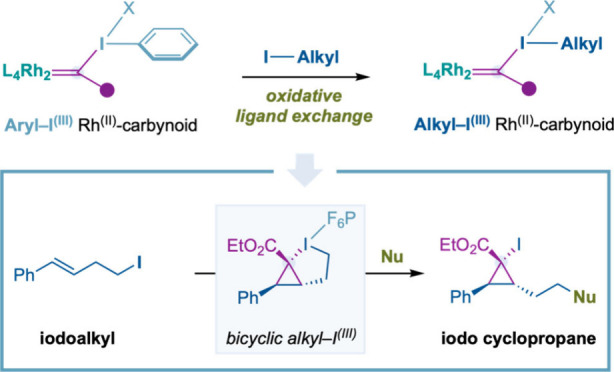

Rh^(II)^-carbynoids, a class of Rh^(II)^-carbenes substituted with a leaving group at the carbene carbon atom, have emerged as key species in the catalytic transfer of monovalent cationic carbon units (:^+^C–R) (Figure 1A).^1^ Our group pioneered their catalytic generation using diazo hypervalent iodine reagents, and we demonstrated that our Rh^(II)^-carbynoids can behave as a Rh^(II)^-carbene and undergo [2 + 1] cycloadditions with alkenes and alkynes.^1b,2,3^ The latter processes led to cyclopropyl–I^(III)^ and cyclopropenyl–I^(III)^ that ultimately evolved to allyl and cyclopropenium cations, respectively. On the other hand, we also observed that the Rh^(II)^-carbynoids could exhibit the reactivity of hypervalent iodine species and undergo nucleophilic attack by carboxylic acids to give a Fischer-type acyloxy Rh^(II)^-carbene^4^ or *para*-selective electrophilic aromatic substitution to generate a donor/acceptor Rh^(II)^-carbene.^5^ DFT calculations carried out for the reaction with carboxylic acids underpinned an initial attack of the carboxylic acid to the hypervalent iodine^(III)^.^4^

**Figure 1:**
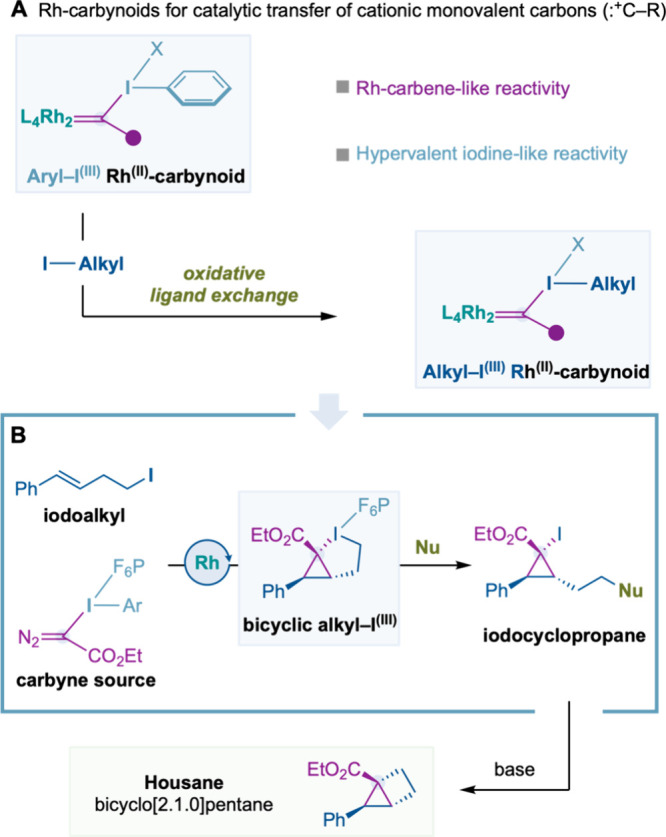
Oxidative ligand exchange of Rh-carbynoids with alkyl iodides.

Such behavior prompted us to question whether our Rh^(II)^-carbynoids could undergo other classes of activations characteristic of hypervalent iodine^(III)^. An interesting process in this area is the oxidative ligand transfer between an aryl–I^(III)^X_2_ and an alkyl–I^(I)^, which produces an alkyl–I^(III)^X_2_ and aryl–I^(I)^.^6^ Taking into account that our Rh^(II)^-carbynoids are functionalized with an aryl–I^(III)^ moiety, we wondered whether such oxidative ligand transfer could occur with readily available alkyl–I^(I)^ compounds. If successful, this process would catalytically generate alkyl–I^(III)^ Rh^(II)^-carbynoids, which remain inaccessible since the corresponding hypervalent iodine reagents are synthetically inaccessible. Moreover, we anticipated that alkyl–I^(III)^ Rh-carbynoids could participate in [2 + 1] cycloadditions with unsaturated substrates and generate novel and unexplored classes of alkyl–I^(III)^.^7^

Here, we report the successful development of such a concept for the catalytic generation of alkyl–I^(III)^ Rh-carbynoids. Using alkyl iodides substituted with an alkenyl group in a remote position, the novel Rh-carbynoid underwent an intramolecular diastereoselective cyclopropanation that led to a distinct class of bicyclic alkyl–I^(III)^ intermediates. The latter species were unstable above −20 °C and, consistent with their electrophilic nature,^8^ underwent regioselective nucleophilic attack by a broad range of nucleophiles. We found that the iodocyclopropane products obtained had utility for the construction of housanes.

Originally, we proposed a mechanism where alkyl iodide **1a** would undergo an oxidative ligand exchange with an aryl–I^(III)^ Rh^(II)^-carbynoid ***int-I***, forming alkyl–I^(III)^ Rh^(II)^-carbynoid ***int-II***. The latter species would evolve through intramolecular cyclopropanation, leading to bicyclic hypervalent iodine ***int-III***. Finally, the formation of cyclopropanes **3** would take place from an α- or β-attack by suitable nucleophiles (Figure 2).

The feasibility of the proposed catalytic generation of alkyl–I^(III)^ Rh-carbynoids was initially explored at −50 °C by stirring alkyl iodide **1a** with Du Bois catalyst^9^ Rh_2_(esp)_2_ in dichloromethane, followed by slow addition of aryliodine^(III)^ diazo reagent **2a** and subsequent addition of tributylmethyl­phosphonium dimethylphosphate as the nucleophile. To our delight, iodocyclopropane **3a** was obtained in 58% yield with excellent diastereoselectivity (*dr* > 20:1, Table 1, entry 1) by selective α-attack of the nucleophile to the corresponding ***int-III***. We did not observe formation of allylic phosphate 3a* resulting from the direct cyclopropanation of ***int-I*** and **1a** followed by electrocyclic ring opening.^1b,2^

**Table 1:**
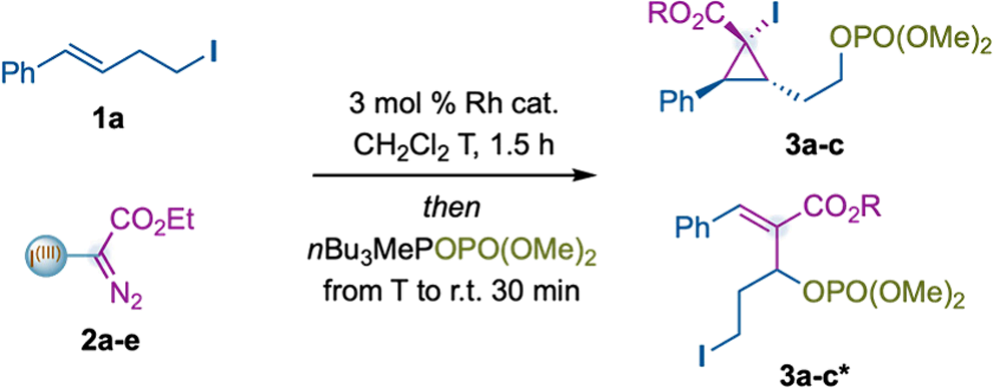
Optimization Studies^a^

entry	**2**	**1a**:**2**	Rh cat.	*T* (°C)	yield **3a**–**c** (%)^a^	ratio **3**:3*^b^
1	**2a**	1:1.2	Rh_2_(esp)_2_	–50	58	>20:1
2	**2b**	1:1.2	Rh_2_(esp)_2_	–50	50	10:1
3	**2c**	1:1.2	Rh_2_(esp)_2_	–50	40	>20:1
4	**2a**	1:1.2	Rh_2_(OAc)_4_	–50	n.d.	-
5	**2a**	1:1.2	Rh_2_(TFA)_4_	–50	n.d.	-
6	**2a**	1:1.2	Rh_2_(TPA)_4_	–50	n.d.	-
7	**2a**	1:1.2	Rh_2_(oct)_4_	–50	13	>20:1
8	**2a**	1:1.2	Rh_2_(adc)_4_	–50	50	5:1
9	**2a**	1:1.2	Rh_2_(OPiv)_4_	–50	35	18:1
10	**2a**	1:1.2	Rh_2_(esp)_2_	–40	61	>20:1
11	**2a**	1.2:1	Rh_2_(esp)_2_	–40	72	>20:1
12	**2d**	1.2:1	Rh_2_(esp)_2_	–40	65	>20:1
13	**2e**	1.2:1	Rh_2_(esp)_2_	–40	67	>20:1

a Reactions performed at 0.1 mmol scale. Yields reported on the basis of ^1^H NMR analysis of the crude reaction using CH_2_Br_2_ as internal standard.

b **3**:3* ratios were determined by ^1^H NMR analysis of the crude. esp = α,α,α′,α′-tetramethy-1,3-benzenedipropanoate. TFA = trifluoroacetate. TPA = triphenylacetate. Oct = octanoate. Adc = adamantane-1-carboxylate. OPiv = pivaloate.

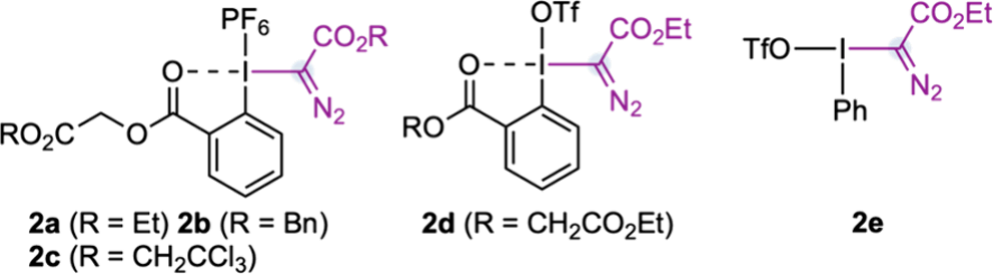

Modulation of the ester substitution in reagent **2** to benzyl (**2b**) or trichloroethyl (**2c**) affected the reaction outcome: the former enhanced the formation of the carbon-insertion product 3b* (*E*/*Z* > 20:1), while the latter slightly reduced the yield (Table 1, entries 2, 3). Alternative rhodium paddlewheel catalysts, including Rh_2_(OAc)_4_, the more electrophilic Rh_2_(TFA)_4_, Rh_2_(TPA)_4_, and Rh_2_(oct)_4_, failed to catalyze this transformation (Table 1, entries 4–7). In contrast, the more sterically demanding Rh_2_(adc)_4_ and Rh_2_(OPiv)_4_ delivered **3a**, albeit in diminished yields (Table 1, entries 8, 9), suggesting that steric and electronic tuning of the catalyst substantially impacts the efficiency of the oxidative ligand transfer process. Further optimization involved elevating the reaction temperature to −40 °C and adjusting the stoichiometry between **1a** and **2a**, both of which improved the reaction outcome (Table 1, entries 10, 11). Finally, we observed that both pseudocyclic and linear triflate reagents led to **3a** in slightly lower yields (entries 12, 13).^10^

After the optimization studies, we explored a broad range of heteroatomic nucleophiles and observed that negatively charged nucleophiles derived from tetrabutylammonium salts of nitrate (**3d**), mesylate (**3e**), triflate (**3f**), halides (**3g**–**j**), cyanide (**3k**), azide (**3l**), or benzoate (**3m**) provided the corresponding cyclopropanes from moderate to good yields (Table 2A). Neutral heteroatomic nucleophiles such as alcohols (**3n**), ethers (**3o**), thiol (**3p**), secondary and tertiary amines (**3q**–**s**), cyclic and linear amides (**3u**,**v**), and nitrogen-heterocycles (**3w**) performed well. On the other hand, activated arenes such as anisole (**3x**) or mesytilene (**3y**) as well as allyltrimethylsilane (**3z**) provided the corresponding C–C bond-forming products. It is worth highlighting that, in any case, we did not observe even traces of the corresponding β-attack products.

**Figure 2:**
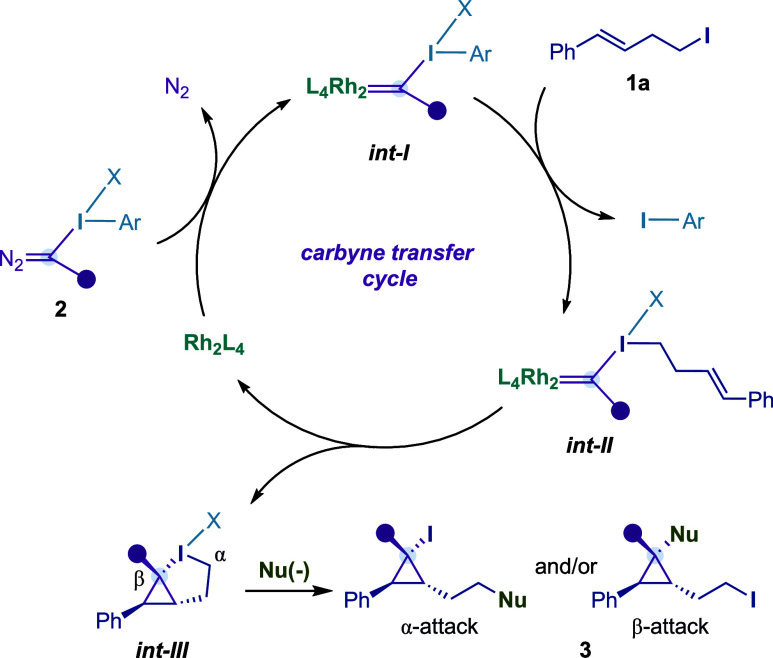
Mechanistic hypothesis.

**Table 2:**
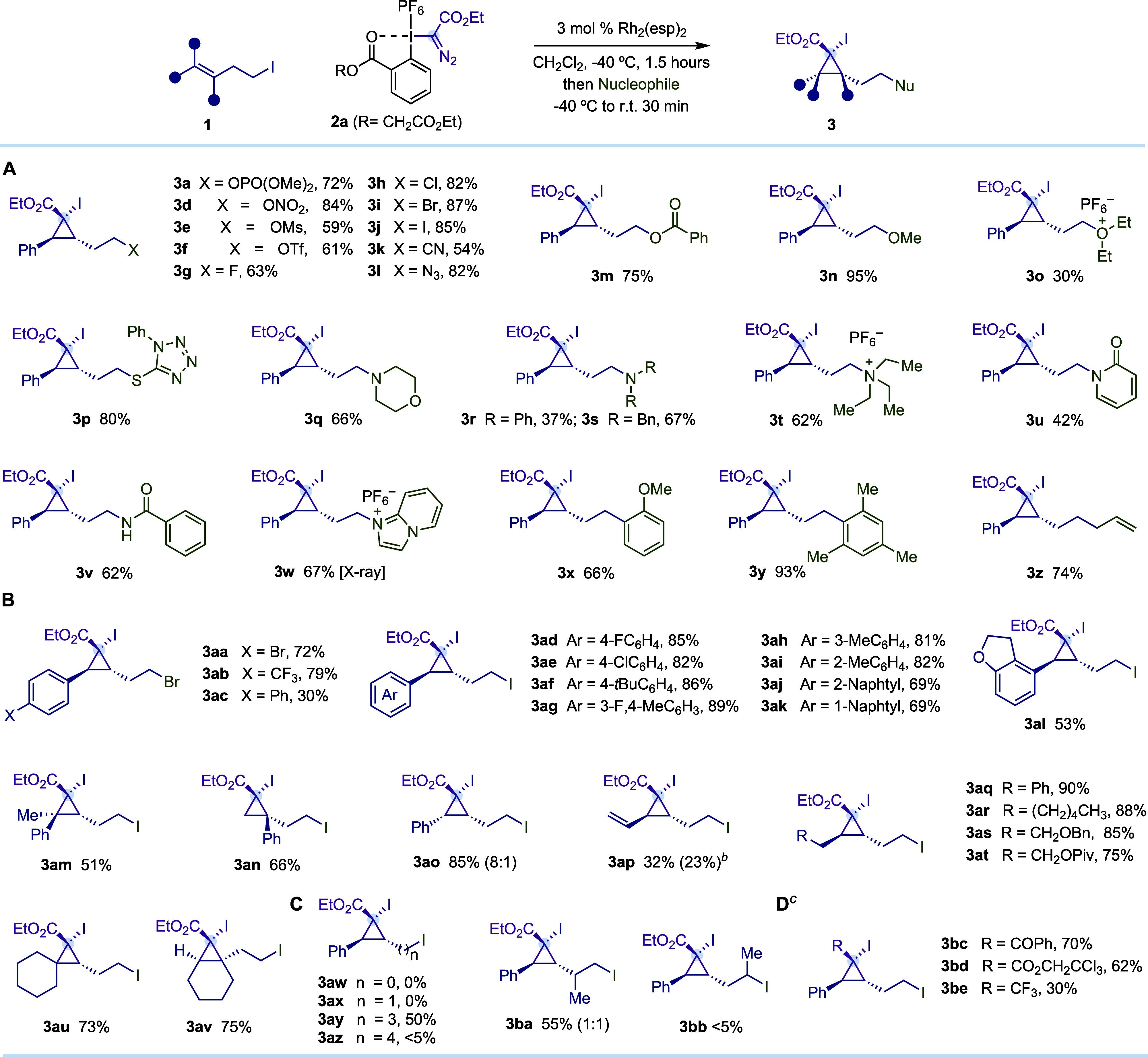
Nucleophile and Alkyl Iodide Scope for the Synthesis of Cyclopropanes **3** ^a^

a Reactions performed at 0.2 mmol scale using 1.2 equiv of the corresponding alkyl iodide and 1.0 equiv of reagent **2**.

b Allylic iodide byproduct 3ap* was isolated in 20% yield.

c Linear hypervalent iodine reagents Ph­(OTf)­I–R **2f** (R = COPh), **2g** (R = CO_2_CH_2_Cl_3_), and **2h** (R = CF_3_) were used. Yields are reported on the basis of the isolated pure product using flash column chromatography. Diastereomeric ratios are >20:1 unless otherwise stated in brackets and were determined by ^1^H NMR analysis of the crude reaction. Relative configurations of **3a**, **3am**, and **3ao** were assigned based on ^1^H–^1^H NOESY experiments and confirmed for **3w** by single-crystal X-ray diffraction analysis.

After this, we decided to evaluate the alkyl iodide scope using *trans*-styryl derivatives substituted at the *para* (**3aa**–**af**), *meta* (**3ag**–**ah**), and *ortho* (**3ai**) positions as well as naphthalene derivatives (**3aj**,**ak**) and heterocycles (**3al**), which were well tolerated (Table 2B). The methodology proved amenable to trisubstituted olefins, as exemplified by the efficient formation of **3am** with excellent diastereoselectivity. 1,1-Disubstituted terminal alkenes were also tolerated (**3an**), highlighting the method’s capacity to accommodate differently hindered alkene substitution patterns. The use of *cis*-styryl homoallylic iodide (*Z*/*E* > 20:1) led to the formation of **3ao** as an 8:1 mixture of diastereoisomers, suggesting that the cyclopropanation step likely proceeds via a cationic intermediate susceptible to isomerization. Notably, 1,3-diene provided access to **3ap**, indicating selective reaction at the homoallylic double bond while preserving the distal olefin for potential functionalization. In addition, single-carbon insertion byproduct 3ap* was also isolated. Nonactivated aliphatic olefins derived from 2-pentenylbenzene and octene delivered **3aq** and **3ar** in excellent yields, and alcohol-protected aliphatic alkenes were also well tolerated (**3as**,**at**). Moreover, our process enabled the synthesis of spirocyclic compound **3au** and bicyclic product **3av**, thus highlighting the versatility of our protocol in reaching complex cyclopropanes.

After this, we investigated the influence of the iodoalkyl chain length of **1** (Table 2C). *trans*-β-Iodostyrene and cinnamyl iodide failed to deliver the expected products **3aw** and **3ax**, likely due to the high ring strain associated with the formation of the corresponding three- and four-membered bicyclic alkyl–I^(III)^ intermediates (***int-III***). However, extending the carbon chain by two methylene units (*n* = 3) led to the formation of **3ay** in 50% yield. Further elongation of the alkyl chain resulted in a loss of reactivity with traces of detectable formation of the desired product **3az**.

The effect of substitution in the alkyl chain was examined by introducing a methyl group in the allylic position of the homoallylic iodide, furnishing **3ba** in 55% yield as an equimolecular mixture of diastereoisomers (Table 2C). In contrast, methyl substitution in the same carbon bearing the iodine led to traces of **3bb**, likely due to steric clashes between the methyl group and Rh^(II)^-carbynoid. Finally, we were delighted to observe that alternative ketone, ester, or trifluoromethyl linear reagents **2f**–**h** were well tolerated (Table 2D, **3bc**–**be**).^11^

Encouraged by the success of the scope evaluation, we next wondered whether alkyl–I^(III)^ Rh-carbynoids could be generated from simple alkyl iodides such as methyl iodide and evolve through intermolecular alkene cyclopropanation. We observed that iodocyclopropanes **4a**,**b** could be obtained from styrenes albeit in low efficiency and low or no diastereocontrol.^12^ However, cyclic alkenes such as cyclohexene or 1,4-cyclohexadiene provided cyclopropanes **4c** and **4d** in good yields and diastereoselectivity (Figure 3A).

**Figure 3:**
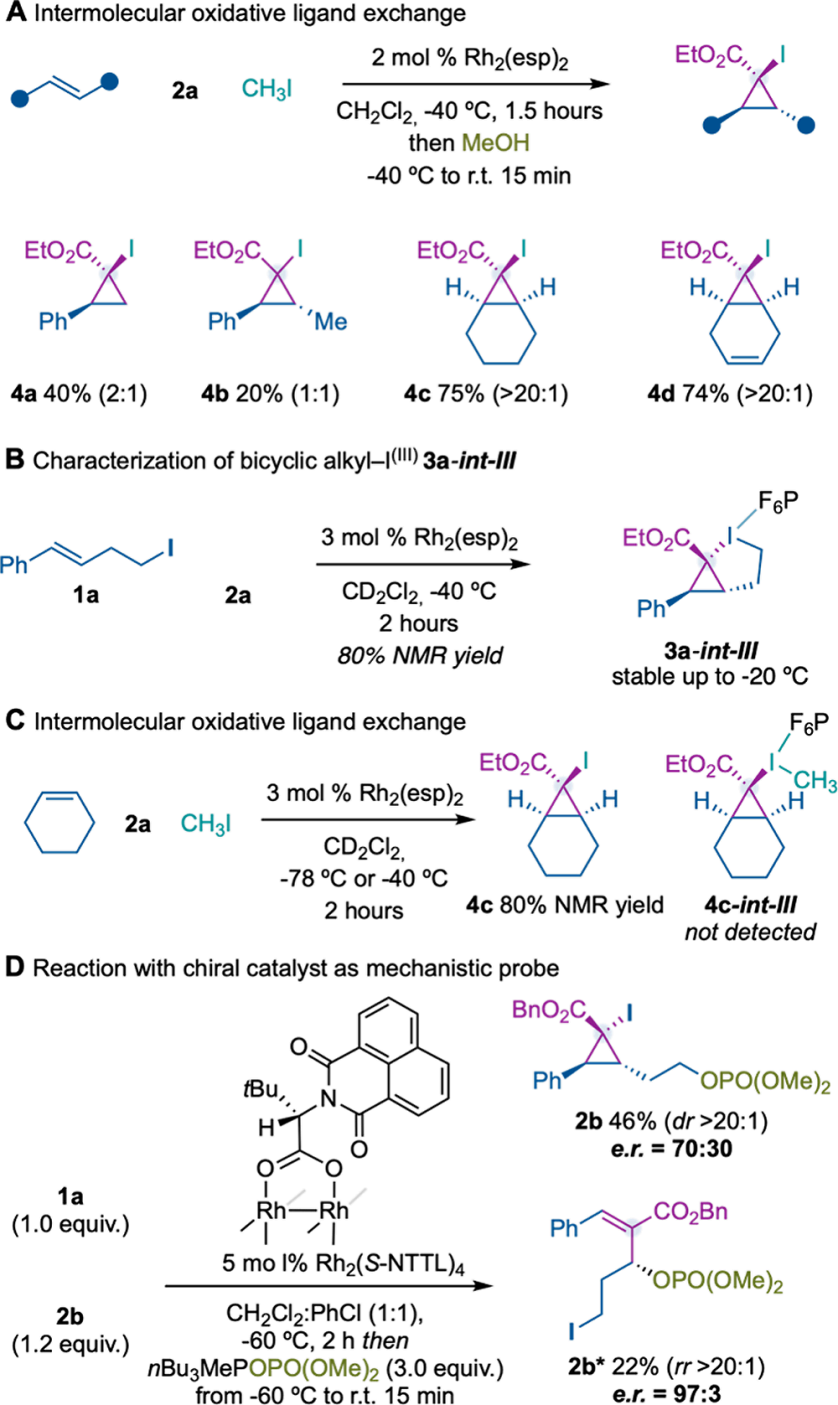
Intermolecular oxidative ligand exchange, detection of **3a*-int-III***, and control experiments.

On the other hand, successful attempts to detect the corresponding bicyclic hypervalent iodine ***int-III*** intermediates (see Figure 2) were carried out with **1a** and **2a** under the optimized reaction conditions in CD_2_Cl_2_. We observed the clean formation of **3a**-***int-III*** at −40 °C, whose structure was assigned by 1D/2D NMR experiments and HRMS. Although we observed that **3a**-***int-III*** was stable up to −20 °C, we failed to confirm its structure by X-ray diffraction analysis (Figure 3B). Encouraged by these results, we focused on detecting by ^1^H NMR intermediate **4c**-***int-III*** produced from cyclohexene, reagent **2a**, and methyl iodide. However, only formation of **4c** was observed, which suggested a lower stability of **4c**-***int-III*** in comparison to **3a**-***int-III*** (Figure 3C).^6a^

Finally, a reaction carried out with chiral catalyst Rh_2_(*S*-NTTL)_4_ (under the previously optimized reaction conditions for the enantioselective single-carbon insertion into alkenes)^2^ with homoallylic iodide **1a** and **2b** led to a separable mixture of cyclopropane **2b** and allylic phosphate 2b* (Figure 3D). The disparity in enantiocontrol in the formation of both products clearly supported two different reaction mechanisms. In this sense, the formation of ***int-III*** by alkene cyclopropanation with ***int-I*** and subsequent oxidative ligand exchange is less likely than the proposed mechanism depicted in Figure 2.

After the development of our oxidative ligand transfer of alkyl iodides with Rh-carbynoids, we questioned the synthetic potential of the iodocyclopropane **3** products. In 2024, the group of Marek reported a stereocontrolled synthesis of bicyclo[1.1.0]­butanes (BCBs) using iodocyclopropanes containing a leaving group in an appropriate position.^13^ The cyclization was promoted by the generation of a lithium cyclopropyl intermediate with *n*BuLi via Li–I exchange. Inspired by this work, we thought that our cyclopropyl derivatives **3** could be suitable starting materials for the synthesis of bicyclo[2.1.0]­pentanes, usually named housanes. Such strained, *sp* ^3^-rich scaffolds remain underexplored in medicinal chemistry due to lack of general methodologies.^14^ This is in sharp contrast with other small-ring systems such as cyclopropanes, cyclobutanes, or bicyclo[1.1.1]­pentanes (BCPs) known to improve pharmacokinetic properties, target selectivity, and clinical success rates.^15^

To prove our hypothesis, we treated bromide derivative **3i** and *n*BuLi in THF at −78 °C and observed the desired housane product **5a** (35% yield) and cyclopropane 5* (50% yield), which was generated from the protonation of the corresponding cyclopropyllithium intermediate (Figure 4A, entry 1). Then we observed that while *t*BuLi provided poor yields, LDA (Li–N*i*Pr_2_) led to **5a** as the major compound (Figure 4a, entries 2, 3).^16^ As expected, the nature of the leaving group significantly impacted the reaction outcome, observing the highest yields for the iodocyclopropyl derivative **3j**, and no formation of 5* took place (Figure 4A, entries 4–7).

**Figure 4:**
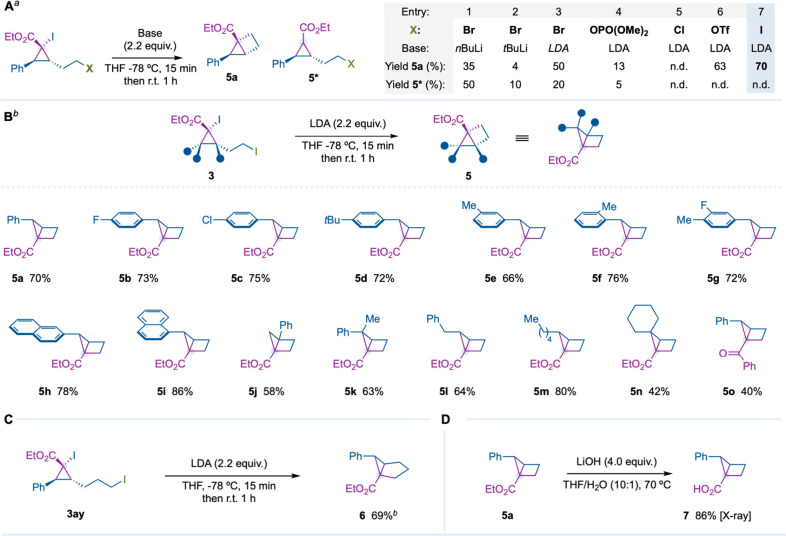
^*a*^Reactions performed at 0.1 mmol with **3a**, **3f**, **3h**, **3l**, **3j**, and 2.2 equiv of lithium base in THF at −78 °C for 1 h; yields are reported on the basis of ^1^H NMR analysis of the crude reaction using CH_2_Br_2_ as the internal standard. ^*b*^Reactions performed with **3j**, **3ad**–**ak**, **3am**, **3an**, **3aq**, **3ar**, **3ay**, **3au**, **3bc**, **3bd** (0.1 mmol, 1.0 equiv), LDA (0.22 mmol, 2.2 equiv), and THF (2.0 mL) at −78 °C for 1 h; yields are reported on the basis of the isolated pure product using flash column chromatography. The relative configuration of **5** was assigned by analogy of that of **7**, which was determined by single-crystal X-ray diffraction analysis. Diastereomeric ratios are >20:1 and were determined by ^1^H NMR analysis of the crude reaction.

After identifying iodide as the suitable leaving group and LDA as the lithium–halogen exchange agent, we were delighted to observe that a series of iodocyclopropane derivatives substituted with different aromatic and aliphatic groups were well tolerated, observing formation of housanes **5b**–**p** in good yields and excellent diastereocontrol. Moreover, under the optimized reaction conditions, compound **3ay** with a one-carbon longer iodoalkyl chain was successfully transformed in bicyclo[3.1.0]­hexane **6** in good yield (Figure 4C). Finally, ester hydrolysis of **5a** under basic conditions provided housane carboxylic acid **7**, whose structure was confirmed by single-crystal X-ray diffraction analysis (Figure 4D).

In conclusion, we have discovered a new catalytic activation of alkyl iodides with aryl–I^(III)^ Rh-carbynoids that led to alkyl–I^(III)^ Rh-carbynoids. The latter species evolved through diastereoselective inter- and intramolecular cyclopropanations to produce linear and cyclic alkyl–I^(III)^. While we were unable to detect linear alkyl–I^(III)^ species at low temperatures, we characterized cyclic derivative **3a**-***int-III*** produced from a homoallylic iodide. Such cyclic alkyl–I^(III)^ species were generated from a broad range of homoallylic iodides and derivatized with negatively charged and neutral heteroatomic/carbon nucleophiles to produce iodocyclopropanes with excellent diastereoselectivity. The latter served as precursors of functionalized housanes by using a lithium–iodide exchange and intramolecular alkylation. Current work focuses on the search and design of a dirhodium catalyst that provides access to cyclopropanes **3** with excellent enantiocontrol while suppressing the direct single-carbon insertion into the alkene.

## Supplementary Material



## Notes

Experimental procedures and spectral data (PDF)

## References

[1] Mortén M., Hennum M., Bonge-Hansen T. (2015). Synthesis
of Quinoline-3-Carboxylates by a Rh­(II)-Catalyzed Cyclopropanation-Ring
Expansion Reaction of Indoles with Halodiazoacetates. Beilstein J. Org. Chem..

[2] Teo W. J., Esteve Guasch J., Jiang L., Li B., Suero M. G. (2024). Rh-Catalyzed
Enantioselective Single-Carbon Insertion of Alkenes. J. Am. Chem. Soc..

[3] Tu H.-F., Jeandin A., Suero M. G. (2022). Catalytic Synthesis of Cyclopropenium
Cations with Rh-Carbynoids. J. Am. Chem. Soc..

[4] Palomo E., Sharma A. K., Wang Z., Jiang L., Maseras F., Suero M. G. (2023). Generating Fischer-Type
Rh-Carbenes with Rh-Carbynoids. J. Am. Chem.
Soc..

[5] Palomo E., Krech A., Hsueh Y. J., Li Z., Suero M. G. (2025). Rh-Catalyzed
Enantioselective Aryl C–H Bond Cyclopropylation. J. Am. Chem. Soc..

[6] Thiele J., Peter W. (1905). Ueber aliphatische Jodidchloride
und Jodoschloride. Ber. Dtsch. Chem. Ges..

[7] Zhdankin V. V., Stang P. J. (2008). Chemistry of Polyvalent
Iodine. Chem. Rev..

[8] Yashin N. V., Averina E. B., Grishin Yu. K., Rybakov V. B., Kuznetsova T. S., Zefirov N. S. (2016). Oxidative Nucleophilic
Substitution
Reaction of Alkyl Iodides upon Treatment with Perchlorate and Dinitramide
Anions. Synthesis of Alkyl-Substituted Perchlorates. Russ. Chem. Bull..

[9] Espino C. G., Fiori K. W., Kim M., Du Bois J. (2004). Expanding theScope
of C-H Amination through Catalyst Design. J.
Am. Chem. Soc..

[10] We have reported that homoallylic bromides are suitable substrates for the enantioselective single-carbon insertion into alkenes (see ref 2) and never observed products coming from an oxidative ligand transfer with the alkyl-bromide moiety.

[11] Reactions proceeded with full conversion of reagent **2**, and we generally observed unreactive excess of alkyl iodide **1**, nucleophile, and traces (<2% yield) of the corresponding allylic products **3***.

[12] Bonge H. T., Pintea B., Hansen T. (2008). Highly Efficient Formation of Halodiazoacetates
and Their Use in Stereoselective Synthesis of Halocyclopropanes. Org. Biomol. Chem..

[13] Suresh R., Orbach N., Marek I. (2024). Synthesis of Stereodefined Polysubstituted
Bicyclo[1.1.0]­Butanes. J. Am. Chem. Soc..

[14] Gassman P. G., Mansfield K. T. (1967). Bridgehead Substituted Bicyclo[2.1.0]­Pentanes. J. Org. Chem..

[15] Lovering F., Bikker J., Humblet C. (2009). Escape from
Flatland: Increasing Saturation as an Approach to Improving Clinical
Success. J. Med. Chem..

[16] Rocca P., Cochennec C., Marsais F., Thomas-dit-Dumont L., Mallet M., Godard A., Queguiner G. (1993). First Metalation of Aryl Iodides: Directed Ortho-Lithiation
of Iodopyridines, Halogen-Dance, and Application to Synthesis. J. Org. Chem..

