# Supplementary material for: Catalytic Oxidative Ligand Transfer of Rh-Carbynoids and Alkyl Iodides
Source: J Am Chem Soc. 2025 Aug 21;147(35):31444–9. doi: 10.1021/jacs.5c09559 (PMC12412146; doi:10.1021/jacs.5c09559)
Supplement: Supplementary file 1 [file ja5c09559_si_001.pdf]

Catalytic Oxidative Ligand Transfer of Rh-Carbynoids and Alkyl Iodides

Josep Esteve Guasch[‡,†], Debasis Pal[‡], Marcos G. Suero[‡,^,*]

[‡]*Institute of Chemical Research of Catalonia (ICIQ-CERCA), The Barcelona Institute of Science and Technology, Països Catalans 16, 43007 Tarragona, Spain.* [^]*ICREA, Pg. Lluis Companys 23, 08010 Barcelona, Spain.* [†]*Departament de Química Analítica i Química Orgánica, Universitat Rovira i Virgili, Calle Marcel·lí Domingo, 1, Tarragona, 43007, Spain*

*Correspondence to: mgsuero@iciq.es

Supporting Information

Table of Contents

1. General Information **S2**

2. Starting Materials **S3**

2.1. Bis[rhodium(α,α,α′,α′-tetramethyl-1,3-benzenedipropionic acid)] $Rh_2(esp)_2$ S3

2.2. Synthesis of homoallylic iodides 1 .... S3

2.3. Synthesis of hypervalent iodine reagents 2 .... S10

3. Synthesis of iodocyclopropanes 3 .... **S12**

3.1. Reaction optimization S12

3.2. Iodocyclopropane scope S15

4. Synthesis of iodocyclopropanes 4 .... **S43**

5. Synthesis of bicyclic alkyl–$I^{(III)}$ 3a-int-III **S46**

6. Enantiomeric difference mechanistic experiment **S48**

7. Synthesis of housanes **S51**

7.1. Reaction optimization S51

7.2. Housane scope S53

8. Housane derivatization **S62**

9. References **S63**

10. Copies of NMR spectra **S64**

1. General Information

All reagents were used as purchased without further purification. Ethyl diazoacetate, (≥13 wt. % dichloromethane) was purchased from Sigma-Aldrich (Ref. E22201) and used without further purification. Anhydrous solvents were dried by passing through an activated alumina column on a PureSolvTM solvent purification system (Innovative Technologies, Inc., MA). Analytical thin layer chromatography (TLC) was carried out using aluminum sheets with 0.2 mm of silica gel (Merck GF234). Visualization of the developed chromatogram was performed by irradiation with UV light or treatment with a solution of potassium permanganate or vanillin stain followed by heating. Flash column chromatography was performed on silica gel (Aldrich, 230-400 mesh) or neutral silica gel (Material Harvest Ltd., 230-400 mesh). Organic solutions were concentrated under reduced pressure on a Büchi rotatory evaporator. Unless otherwise stated, reactions were carried out under argon atmosphere. Yields refer to purified compounds unless otherwise noted. NMR spectra were recorded at 298 K on Bruker Avance 300, Bruker Avance 400 Ultrashield or Bruker Avance 500 Ultrashield apparatuses. Coupling constants (*J*) are quoted in hertz (Hz). Multiplicity is reported with the following abbreviations: s = singlet, brs = broad singlet, d = doublet, t = triplet, q = quartet, dt = doublet of triplets, td = triplet of doublets, tt = triplet of triplets, sept = septet, m = multiplet, app = apparent. Melting points were measured using open glass capillaries in a Büchi B540 apparatus. Mass spectra were recorded on a Waters LCT Premier spectrometer. Liquid chromatography-mass spectrometry (LC-MS) and gas chromatography-mass spectrometry (GC-MS) analysis were carried out in Agilent 1260 Infinity – 6130 Quadrupole and Agilent 7890B - 5977A MSD, respectively.

2. Starting Materials

2.1. Bis[rhodium(α,α,α′,α′-tetramethyl-1,3-benzenedipropionic acid)] $Rh_2(esp)_2$

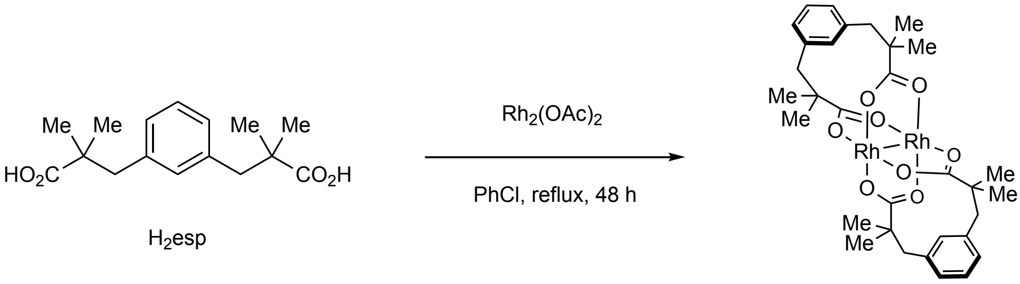

$Rh_2(OAc)_4$ (565 mg, 1.29 mmol, 1.00 equiv.) and H_2esp (863 mg, 3.10 mmol, 2.40 equiv.) were dissolved in chlorobenzene (50 mL) and the mixture was refluxed for 48 hours. The reaction mixture was cooled down to room temperature and the solvent was removed under rotary evaporation. The reaction crude was purified by flash column chromatography (CH_2Cl_2/acetone: 100/0 – 90/10). The solvent was removed under rotary evaporation and the green solid was further dried in high vacuum at 60 ºC to afford $Rh_2(esp)_2$ as light green powder (827 mg, 85% yield). ^1H and ^{13}C NMR matches with the reported data.[1]

2.2. Synthesis of homoallylic iodides 1

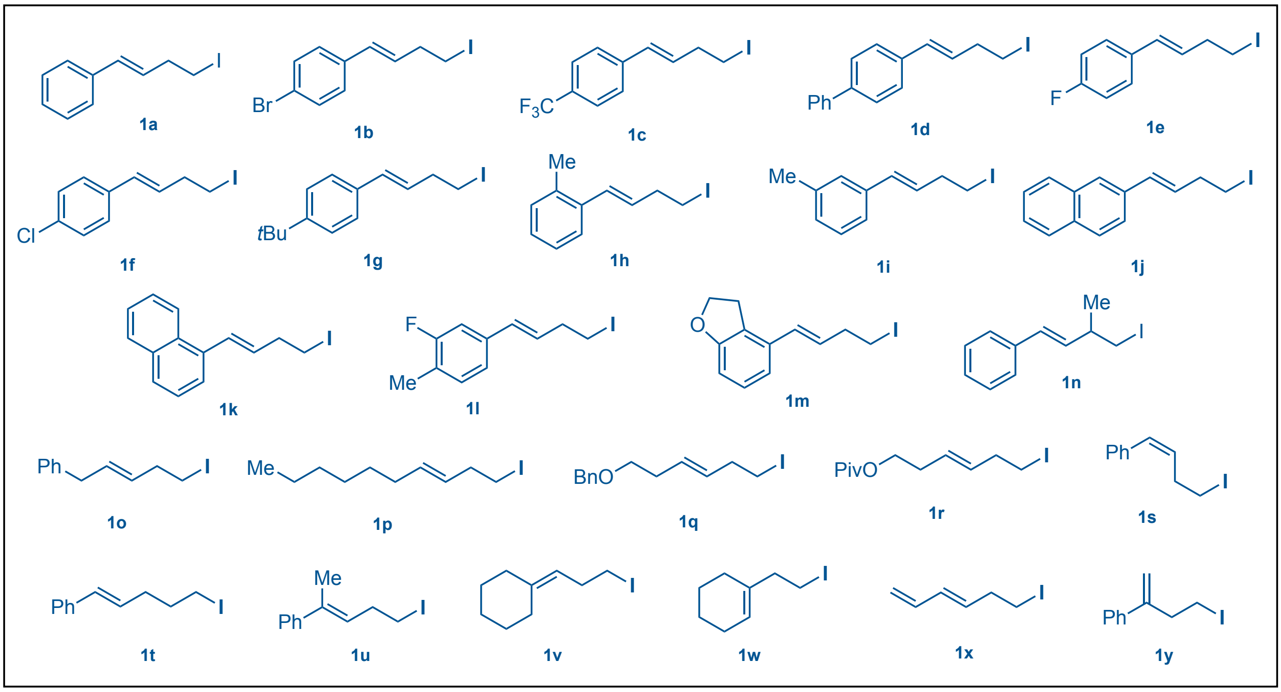

Substrates **1a**[2], **1c**[3], **1d**[2], **1e**[3], **1f**[4], **1h**[2], **1t**[5], **1u**[6], **1v**[3], **1w**[7], **1x**[8], **1y**[9] are known compounds. Substrates **1b, 1g, 1i, 1j**, **1k**, **1l**, **1m**, **1n**, **1o**, **1p**, **1q, 1r, 1s** have been prepared according to the following procedures:

General procedure A:

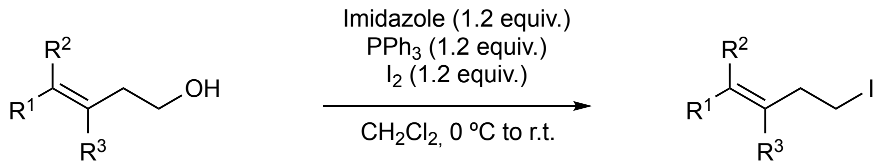

To a solution of imidazole (0.41 g, 6.0 mmol), PPh_3 (1.57 g, 6.0 mmol) and I_2 (1.52 g, 6.0 mmol) in CH_2Cl_2 (50 mL) at 0 ºC, the corresponding homoallylic alcohol (5.0 mmol) was added carefully. After 5 minutes, the reaction was allowed to warm to room temperature and stirred until full conversion of the homoallylic alcohol, monitored by TLC. The reaction was filtered by gravity and washed with a saturated solution of $Na_2S_2O_3$ (40 mL). The organic layer was dried over anhydrous Na_2SO_4 and the solvent removed under rotary evaporation. The crude residue was purified by flash column chromatography to yield the corresponding homoallylic iodides.

General procedure B:

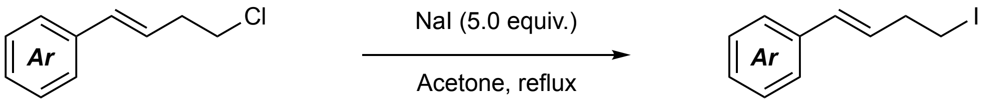

A solution of the corresponding homoallylic chloride (2.0 mmol) and NaI (1.50 g, 10.0 mmol) in acetone (10 mL) was refluxed until full conversion of the homoallylic chloride, monitored by GC–MS (usually 24–48 hours reaction time). Then the reaction was allowed to cool down to room temperature and diluted with Et_2O (20 mL), filtered, washed with a saturated solution of $Na_2S_2O_3$ (40 mL) and dried over anhydrous Na_2SO_4. The solvent was removed under rotary evaporation to yield the corresponding homoallylic iodides.

(*E*)-1-Bromo-4-(4-iodobut-1-en-1-yl)benzene (1b)

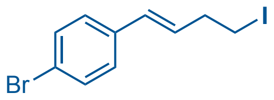

This compound was synthetized following the general procedure **B** using (*E*)-1-bromo-4-(4-chlorobut-1-en-1-yl)benzene (1.23 g, 5.0 mmol), affording the title compound as colourless oil (1.44 g, 86% yield).

^{1}H NMR (400 MHz, $CDCl_3$) δ 7.47 – 7.39 (m, 2H), 7.25 – 7.20 (m, 2H), 6.41 (d, *J* = 15.9 Hz, 1H), 6.14 (dt, *J* = 15.9, 6.9 Hz, 1H), 3.24 (t, *J* = 7.1 Hz, 2H), 2.77 (qd, *J* = 7.1, 1.4 Hz, 2H).

^{13}C NMR (101 MHz, $CDCl_3$) δ 136.1, 131.8, 131.3, 129.5, 127.9, 121.3, 37.0, 4.8.

HRMS: (APCI) calculated for $C_{10}H_{11}BrI$ $[M+H]^+$ m/z: 336.9083, found: 336.9085.

(*E*)-1-(*tert*-Butyl)-4-(4-iodobut-1-en-1-yl)benzene (1g)

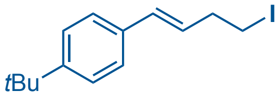

This compound was synthetized following the general procedure **B** using (*E*)-1-(*tert*-butyl)-4-(4-chlorobut-1-en-1-yl)benzene (445.5 mg, 2.0 mmol), affording the title compound as colourless oil (442 mg, 70% yield).

^{1}H NMR (500 MHz, $CDCl_3$) δ 7.36 – 7.29 (m, 4H), 6.45 (d, J = 15.8 Hz, 1H), 6.11 (dt, J = 15.8, 7.1 Hz, 1H), 3.23 (t, J = 7.1 Hz, 2H), 2.77 (qd, J = 7.1, 1.4 Hz, 2H), 1.31 (s, 9H).

^{13}C NMR (126 MHz, $CDCl_3$) δ 150.7, 134.4, 132.2, 127.9, 126.0, 125.7, 37.2, 34.7, 31.4, 5.4.

HRMS: (APCI) calculated for $C_{14}H_{20}I$ $[M+H]^+$ m/z: 315.0604, found: 315.0607.

(*E*)-1-(4-Iodobut-1-en-1-yl)-3-methylbenzene (1i)

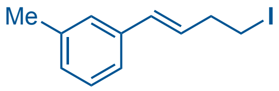

This compound was synthetized following the general procedure **A** using (*E*)-4-(*m*-tolyl)but-3-en-1-ol (811.1 mg, 5.0 mmol). Purification by flash column chromatography (hexane/ethyl acetate: 100/0 → 99/1) provided the title compound as colourless oil (1.20 g, 88% yield).

^{1}H NMR (400 MHz, $CDCl_3$) δ 7.24 – 7.13 (m, 3H), 7.09 –7.03 (m, 1H), 6.45 (dt, J = 15.8, 1.4 Hz, 1H), 6.14 (dt, J = 15.8, 7.0 Hz, 1H), 3.24 (t, J = 7.2 Hz, 2H), 2.78 (qd, J = 7.2, 1.4 Hz, 2H), 2.35 (s, 3H).

^{13}C NMR (126 MHz, $CDCl_3$) δ 138.3, 137.1, 132.5, 128.6, 128.4, 128.4, 127.0, 123.5, 37.2, 21.5, 5.2.

HRMS: (APCI) calculated for $C_{11}H_{14}I$ $[M+H]^+$ m/z: 273.0135, found: 273.0132.

(*E*)-2-(4-Iodobut-1-en-1-yl)naphthalene (1j)

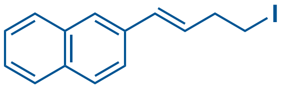

This compound was synthetized following the general procedure **B** using (*E*)-2-(4-chlorobut-1-en-1-yl)naphthalene (1.08 g, 5.0 mmol), affording the title compound as colourless oil (940 mg, 61% yield).

^{1}H NMR (500 MHz, $CDCl_3$) δ 7.84 – 7.74 (m, 3H), 7.73 – 7.69 (m, 1H), 7.59 (dd, *J* = 8.5, 1.8 Hz, 1H), 7.48 – 7.40 (m, 2H), 6.64 (d, *J* = 16.0 Hz, 1H), 6.28 (dt, *J* = 16.0, 7.0 Hz, 1H), 3.29 (t, *J* = 7.2 Hz, 2H), 2.85 (qd, *J* = 7.2, 1.4 Hz, 2H).

^{13}C NMR (126 MHz, $CDCl_3$) δ 134.7, 133.8, 133.1, 132.5, 129.1, 128.4, 128.1, 127.8, 126.4, 126.1, 125.9, 123.6, 37.3, 5.1.

HRMS: (APCI) calculated for $C_{14}H_{14}I$ $[M+H]^+$ m/z: 309.0135, found: 309.0135.

(*E*)-1-(4-Iodobut-1-en-1-yl)naphthalene (1k)

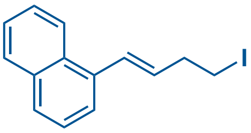

This compound was synthetized following the general procedure **B** using (*E*)-1-(4-chlorobut-1-en-1-yl)naphthalene (1.08 g, 5.0 mmol), affording the title compound as colourless oil (1.25 g, 81% yield).

^{1}H NMR (400 MHz, $CDCl_3$) δ 8.17 – 8.08 (m, 1H), 7.89 – 7.81 (m, 1H), 7.78 (d, *J* = 8.2 Hz, 1H), 7.58 (dd, *J* = 7.2, 1.2 Hz, 1H), 7.55 – 7.41 (m, 3H), 7.22 (d, *J* = 15.6 Hz, 1H), 6.16 (dt, *J* = 15.6, 6.9 Hz, 1H), 3.34 (t, *J* = 7.1 Hz, 2H), 2.91 (qd, *J* = 7.1, 1.5 Hz, 2H).

^{13}C NMR (101 MHz, $CDCl_3$) δ 135.1, 133.8, 131.9, 131.2, 129.9, 128.6, 128.0, 126.2, 125.9, 125.8, 124.1, 124.0, 37.4, 5.4.

HRMS: (APCI) calculated for $C_{14}H_{14}I$ $[M+H]^+$ m/z: 309.0135, found: 309.0137.

(*E*)-2-Fluoro-4-(4-iodobut-1-en-1-yl)-1-methylbenzene (1l)

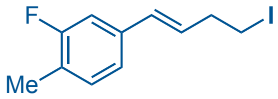

This compound was synthetized following the general procedure **A** using (*E*)-4-(3-fluoro-4-methylphenyl)but-3-en-1-ol (360.4 mg, 2.0 mmol). Purification by flash column chromatography (hexane/ethyl acetate: 100/0 → 99/1) provided the title compound as colourless oil (452.5 mg, 78% yield).

^{1}H NMR (400 MHz, $CDCl_3$) δ 7.10 (t, *J* = 7.8 Hz, 1H), 7.02 (d, *J* = 9.4 Hz, 2H), 6.40 (d, *J* = 15.8 Hz, 1H), 6.10 (dt, *J* = 15.8, 6.9 Hz, 1H), 3.24 (t, *J* = 7.2 Hz, 2H), 2.77 (qd, *J* = 7.1, 1.4 Hz, 2H), 2.25 (d, *J* = 1.9 Hz, 3H).

^{13}C NMR (101 MHz, $CDCl_3$) δ 161.7 (d, *J* = 244.1 Hz), 137.0 (d, *J* = 7.7 Hz), 131.6 (d, *J* = 5.5 Hz), 131.4 (d, *J* = 2.6 Hz), 129.0, 124.1 (d, *J* = 17.6 Hz), 121.9 (d, *J* = 2.9 Hz), 112.4 (d, *J* = 22.7 Hz), 37.0, 14.5 (d, *J* = 3.3 Hz), 4.9.

^{19}F NMR (376 MHz, $CDCl_3$) δ -118.00.

HRMS: (APCI) calculated for $C_{11}H_{13}FI$ $[M+H]^+$ m/z: 291.0041, found: 291.0033.

(*E*)-4-(4-Iodobut-1-en-1-yl)-2,3-dihydrobenzofuran (1m)

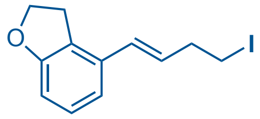

This compound was synthetized following the general procedure **A** using (*E*)-4-(2,3-dihydrobenzofuran-4-yl)but-3-en-1-ol (285.4 mg, 1.5 mmol). Purification by flash column chromatography (hexane/ethyl acetate: 100/0 → 97/3) provided the title compound as colourless oil (330.2 mg, 73% yield).

^{1}H NMR (400 MHz, $CDCl_3$) δ 7.08 (t, *J* = 7.8 Hz, 1H), 6.93 (d, *J* = 7.8 Hz, 1H), 6.69 (d, *J* = 7.9 Hz, 1H), 6.44 (dt, *J* = 15.9, 1.4 Hz, 1H), 6.10 (dt, *J* = 15.8, 6.9 Hz, 1H), 4.59 (t, *J* = 8.6 Hz, 2H), 3.27 – 3.22 (m, 4H), 2.79 (qd, *J* = 7.1, 1.3 Hz, 2H).

^{13}C NMR (101 MHz, $CDCl_3$) δ 160.4, 134.1, 130.4, 130.3, 128.3, 124.7, 117.9, 108.3, 71.1, 37.3, 29.4, 5.2.

HRMS: (APCI) calculated for $C_{12}H_{14}IO$ $[M+H]^+$ m/z: 301.0084, found: 301.0081.

(*E*)-(4-Iodo-3-methylbut-1-en-1-yl)benzene (1n)

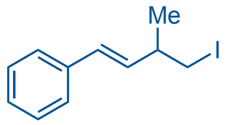

This compound was synthetized following the general procedure **A** using (*E*)-2-methyl-4-phenylbut-3-en-1-ol (973.4 mg, 6.0 mmol). Purification by flash column chromatography (hexane/ethyl acetate: 100/0 → 99/1) provided the title compound as colourless oil (1.32 g, 81% yield).

^{1}H NMR (400 MHz, $CDCl_3$) δ 7.40 – 7.34 (m, 2H), 7.31 (dd, *J* = 8.5, 6.7 Hz, 2H), 7.25 – 7.20 (m, 1H), 6.44 (d, *J* = 15.8 Hz, 1H), 6.08 (dd, *J* = 15.9, 7.5 Hz, 1H), 3.27 (dd, *J* = 9.6, 5.9 Hz, 1H), 3.21 (dd, *J* = 9.6, 6.7 Hz, 1H), 2.56 (hept, *J* = 6.3 Hz, 1H), 1.23 (d, *J* = 6.7 Hz, 3H).

^{13}C NMR (101 MHz, $CDCl_3$) δ 137.3, 133.3, 130.4, 128.7, 127.5, 126.4, 39.2, 21.0, 15.0.

HRMS: (APCI) calculated for $C_{11}H_{14}I$ $[M+H]^+$ m/z: 273.0135, found: 273.0132.

(*E*)-(5-Iodopent-2-en-1-yl)benzene (1o)

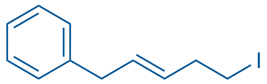

This compound was synthetized following the general procedure **A** using (*E*)-5-phenylpent-3-en-1-ol (811.2 mg, 5.0 mmol). Purification by flash column chromatography (hexane/ethyl acetate: 100/0 → 99/1) provided the title compound as colourless oil (1.13 g, 83% yield).

^{1}H NMR (500 MHz, $CDCl_3$) δ 7.32 – 7.27 (m, 2H), 7.24 – 7.16 (m, 3H), 5.69 (dtt, *J* = 15.0, 6.8, 1.3 Hz, 1H), 5.46 (dtt, *J* = 15.1, 6.8, 1.5 Hz, 1H), 3.35 (d, *J* = 6.8 Hz, 2H), 3.17 (t, *J* = 7.2 Hz, 2H), 2.63-2.56 (m, 2H).

^{13}C NMR (126 MHz, $CDCl_3$) δ 140.4, 132.0, 130.1, 128.7, 128.6, 126.2, 39.1, 36.7, 5.9.

HRMS: (APCI) calculated for $C_{11}H_{14}I$ $[M+H]^+$ m/z: 273.0135, found: 273.0131.

(*E*)-1-Iododec-3-ene (1p)

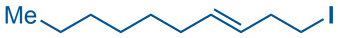

This compound was synthetized following the general procedure **A** using (*E*)-dec-3-en-1-ol (468.8 mg, 3.0 mmol). Purification by flash column chromatography (hexane/ethyl acetate: 100/0) provided the title compound as colourless oil (702.7 mg, 88% yield).

^{1}H NMR (500 MHz, $CDCl_3$) δ 5.52 (dtt, *J* = 14.8, 6.7, 1.3 Hz, 1H), 5.34 (dtt, *J* = 15.0, 6.7, 1.4 Hz, 1H), 3.14 (t, *J* = 7.3 Hz, 2H), 2.54 (qd, *J* = 7.3, 1.2 Hz, 2H), 2.02 – 1.95 (m, 2H), 1.45 – 1.15 (m, 8H), 0.88 (t, *J* = 7.0 Hz, 3H).

^{13}C NMR (126 MHz, $CDCl_3$) δ 133.8, 128.4, 36.9, 32.6, 31.9, 29.4, 29.0, 22.8, 14.3, 6.4.

HRMS: (APCI) calculated for $C_{10}H_{11}BrI$ $[M+H]^+$ m/z: 267.0604, found: 267.0601.

(*E*)-(((6-Iodohex-3-en-1-yl)oxy)methyl)benzene (1q)

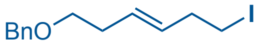

This compound was synthetized following the general procedure **A** using (*E*)-6-(benzyloxy)hex-3-en-1-ol (361.0 mg, 1.75 mmol). Purification by flash column chromatography (hexane/ethyl acetate: 100/0 → 95/5) provided the title compound as colourless oil (475.8 mg, 86% yield).

^{1}H NMR (500 MHz, $CDCl_3$) δ 7.39 – 7.32 (m, 4H), 7.32 – 7.26 (m, 1H), 5.57 (dtt, *J* = 15.6, 6.5, 1.2 Hz, 1H), 5.46 (dtt, *J* = 15.4, 6.6, 1.3 Hz, 1H), 4.52 (s, 2H), 3.51 (t, *J* = 6.7 Hz, 2H), 3.15 (t, *J* = 7.3 Hz, 2H), 2.57 (dtt, *J* = 7.4, 6.4, 1.1 Hz, 2H), 2.34 (qq, *J* = 6.7, 1.0 Hz, 2H).

^{13}C NMR (126 MHz, $CDCl_3$) δ 138.6, 130.5, 129.7, 128.5, 127.8, 127.7, 73.0, 69.9, 36.9, 33.1, 5.8.

HRMS: (ESI) calculated for $C_{13}H_{17}INaO$ $[M+Na]^+$ m/z: 339.0216, found: 339.0216.

(*E*)-6-Iodohex-3-en-1-yl pivalate (1r)

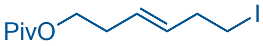

This compound was synthetized following the general procedure **A** using (*E*)-6-hydroxyhex-3-en-1-yl pivalate (400.6 mg, 2.0 mmol). Purification by flash column chromatography (hexane/ethyl acetate: 100/0 → 96/4) provided the title compound as colourless oil (446.7 mg, 72% yield).

^{1}H NMR (500 MHz, $CDCl_3$) δ 5.57 – 5.40 (m, 2H), 4.08 (t, *J* = 6.6 Hz, 2H), 3.13 (t, *J* = 7.2 Hz, 2H), 2.60 – 2.51 (m, 2H), 2.37 – 2.28 (m, 2H), 1.19 (s, 9H).

^{13}C NMR (126 MHz, $CDCl_3$) δ 178.6, 131.3, 128.7, 63.6, 38.9, 36.8, 32.1, 27.4, 5.5.

HRMS: (ESI) calculated for $C_{11}H_{19}INaO_2$ $[M+Na]^+$ m/z: 333.0322, found: 333.0320.

(*Z*)-(4-Iodobut-1-en-1-yl)benzene (1s)

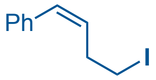

This compound was synthesized following the general procedure **A** using (*Z*)-4-phenylbut-3-en-1-ol (874 mg, 5.9 mmol). Purification by flash column chromatography (100% hexane) provided the title compound as colourless oil (1.2 g, 80% yield, >20:1 with *trans* isomer).

^{1}H NMR (400 MHz, $CDCl_3$) δ 7.38 – 7.32 (m, 2H), 7.26 (dt, *J* = 8.9, 3.1 Hz, 3H), 6.58 (dd, *J* = 11.5, 1.8 Hz, 1H), 5.62 (dt, *J* = 11.5, 7.1 Hz, 1H), 3.22 (t, *J* = 7.1 Hz, 2H), 2.92 (qd, *J* = 7.1, 1.8 Hz, 2H).

^{13}C NMR (101 MHz, $CDCl_3$) δ 137.2, 131.1, 130.8, 128.7, 128.4, 127.1, 32.5, 5.1.

HRMS: (APCI) calculated for $C_{10}H_{12}I$ $[M+H]^+$ m/z: 258.9978, found: 258.9977.

2.3. Synthesis of hypervalent iodine reagents 2

Reagents **2a**, **2b** and **2c** were synthesized according to an existing procedure[10]:

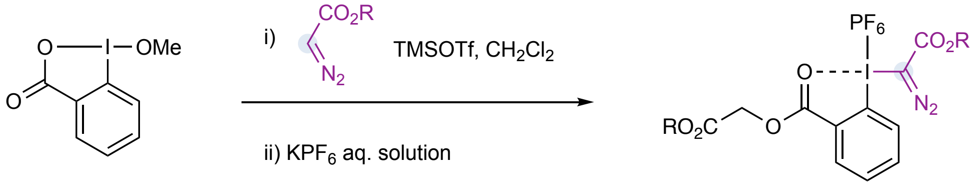

A solution of 1-methoxy-1,2-benziodoxol-3(1*H*)-one (4.0 g, 14.3 mmol) in dichloromethane (30 mL) was treated with trimethylsilyl trifluoromethanesulfonate (3.2 g, 14.3 mmol) at room temperature. After 30 minutes, a cloudy suspension was observed and benzyl 2-diazoacetate (5.57 g, 31.6 mmol), ethyl 2-diazoacetate (3.61 g, 31.6 mmol) or 2,2,2-trichloroethyl 2-diazoacetate (6.87 g, 31.6 mmol) was added dropwise during 10 minutes. Nitrogen evolution was observed and the resulting reaction mixture was stirred at room temperature until a clear yellow solution was observed (1 hour). The solvent was removed under rotary evaporation and the reaction crude was redissolved in dichloromethane (0.2 M) and washed with a saturated aqueous solution of KPF_6 (3 x 25 mL) in a separation funnel. The combined organic layers were dried over Na_2SO_4 and solvent was removed under rotary evaporation. The crude mixture was sonicated in the mixture of Et_2O/Hexane/CH_2Cl_2 (8:1.5:0.5) and decanted from the yellow solid residue. The yellow solid was dried under vacuum to obtain **2a-c** (**2a**, R = Et, 7.28 g, 86% yield), (**2b**, R = Bn, 8.75 g, 85% yield), (**2c**, R = CH_2CCl_3, 8.00 g, 70% yield).

(*Note: if* **2a-c** *contain impurities, can be recrystallized with CH_2Cl_2/Et_2O (1:4) at -30 ºC*).

(1-diazo-2,2,2-trichloroethyl)(2-(2,2,2-trifluoroethyl-2-oxoethoxyl)carbonylphenyl)iodonium hexafluorophosphate (2c)

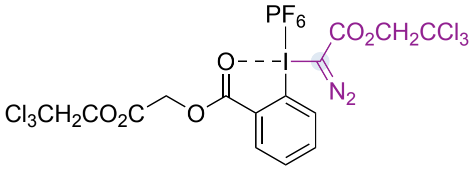

^{1}H NMR (400 MHz, $(CD_3)_2CO$) δ 8.55 (dd, *J* = 7.5, 1.7 Hz, 1H), 8.38 (d, *J* = 8.4 Hz, 1H), 8.15 (ddd, *J* = 8.4, 7.5, 1.7 Hz, 1H), 8.04 (td, *J* = 7.5, 0.9 Hz, 1H), 5.45 (s, 2H), 5.07 (s, 2H), 5.01 (s, 2H).
^{13}C NMR (126 MHz, $CDCl_3$) δ 169.7, 164.7, 160.1, 139.0, 133.9, 132.5, 128.9, 124.9, 115.3, 94.2, 94.1, 75.6, 74.7, 63.6. (the resonance resulting from the carbonyl carbon in the 2,2,2-trichloroethyl ester was not detected)
^{19}F NMR (376 MHz, $(CD_3)_2CO$) δ -71.8 (d, *J* = 708.7 Hz).
^{31}P NMR (162 MHz, $(CD_3)_2CO$) δ -141.17 (hept, *J* = 708.7 Hz).
HRMS (MALDI): calculated for $C_{15}H_{10}Cl_6IN_2O_6$ $[M-PF_6]^+$ m/z: 650.7685, found: 650.7670.

Reagents **2d-f** were synthetized according to an existing procedure: [11]

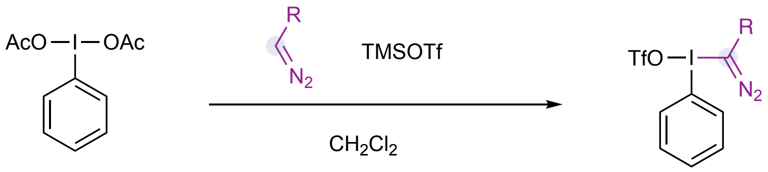

To a solution of phenyliodoso diacetate (1.6 g, 5.0 mmol) in dichloromethane (20 mL, 0.5 M) was added trimethylsilyl trifluoromethanesulfonate (0.9 mL, 5.0 mmol) at room temperature. Then the corresponding diazoacetate (11.0 mmol) was added dropwise during 10 minutes. The resulting reaction mixture was stirred at room temperature for 2 hours. Solvent was removed under vacuum and the crude was recrystallized from a mixture of diethyl ether/dichloromethane (5/1) during 12 hours at -30 °C. The desired product was collected by filtration washed with cold diethyl ether (100 mL) and dried under high vacuum to obtain **2f-h** as yellow solid (**2f**, R = COPh, 1.02 g, 41% yield), (**2g**, R = $CO_2CH_2CCl_3$, 2.15 g, 76% yield), (**2h**, R = CF_3, 1.61 g, 71% yield).

3. Synthesis of iodocyclopropanes 3

3.1. Reaction optimization

General procedure C: To a 10 mL oven-dried reaction vial equipped with a stirring bar was added the corresponding dirhodium catalyst Rh_2L_4 (0.003 mmol, 3 mol%) and homoallylic iodide **1a**. The tube was sealed before being evacuated and backfilled with argon three times. Dichloromethane (0.3 mL) was added, and the resulting mixture was cooled at the corresponding temperature. Then, a solution of reagent **2** in dichloromethane (1.0 mL) was added dropwise over 30 minutes using a syringe pump and after stirred for 60 minutes at the corresponding temperature. After this, a solution of tributyl(methyl)phosphonium dimethylphosphate (102.7 mg, 0.3 mmol) in dichloromethane (1.0 mL) was added dropwise during 10 minutes. Then the resulting reaction mixture was allowed to warm to room temperature during 15 minutes followed by the removal of solvent by rotatory evaporation. The crude residue was dissolved in $CDCl_3$ and analyzed by ^1H NMR spectroscopy using CH_2Br_2 (7 µL, 0.1 mmol) as internal standard. The crude residue was dissolved in $CDCl_3$ and analyzed by ^1H NMR using CH_2Br_2 (7 µL, 0.1 mmol) as internal standard, with yields calculated relative to CH_2Br_2 and the **5**/**5*** ratio determined by signal integration.

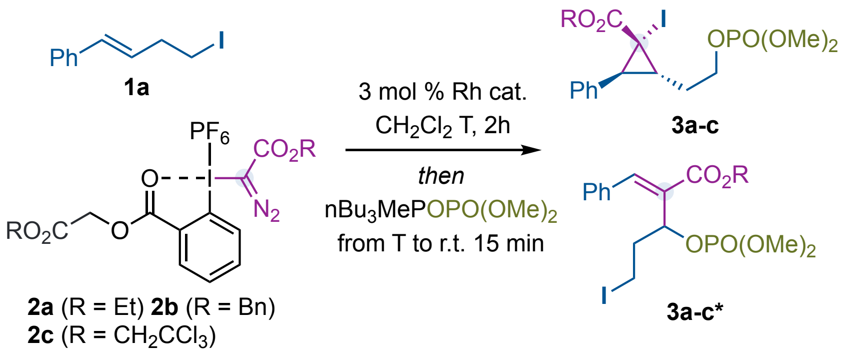

entry	2	1a:2	Rh cat.	T (°C)	Yield 3a-c (%)[a]	ratio 3:3*[b]
1	**2a**	1:1.2	$Rh_2(esp)_2$	-50	58	>20:1
2	**2b**	1:1.2	$Rh_2(esp)_2$	-50	50	10:1
3	**2c**	1:1.2	$Rh_2(esp)_2$	-50	40	>20:1
4	**2a**	1:1.2	$Rh_2(OAc)_4$	-50	n.d.	-
5	**2a**	1:1.2	$Rh_2(TFA)_4$	-50	n.d.	-
6	**2a**	1:1.2	$Rh_2(TPA)_4$	-50	n.d.	-
7	**2a**	1:1.2	$Rh_2(oct)_4$	-50	13	>20:1
8	**2a**	1:1.2	$Rh_2(adc)_4$	-50	50	5:1
9	**2a**	1:1.2	$Rh_2(OPiv)_4$	-50	35	18:1
10	**2a**	1:1.2	$Rh_2(esp)_2$	-40	61	>20:1
11	**2a**	1.2:1	$Rh_2(esp)_2$	-40	72	>20:1

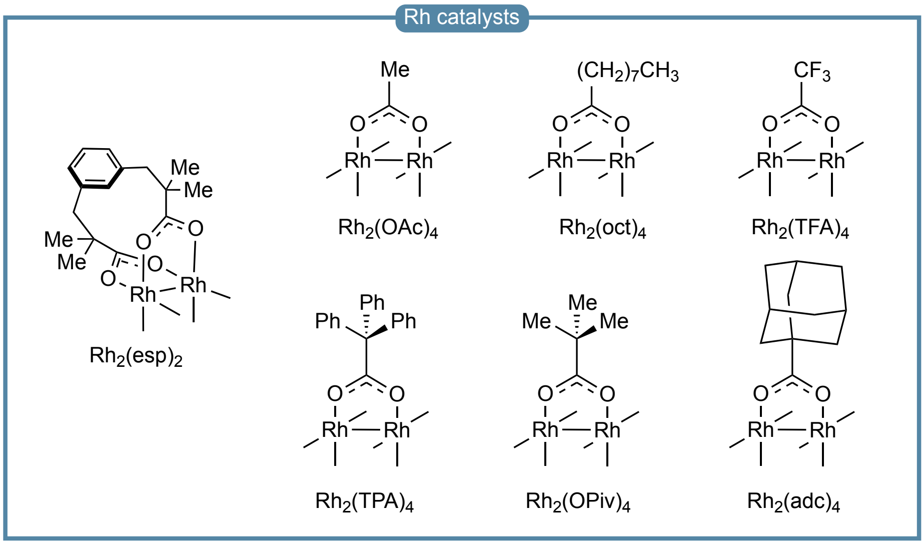

Ethyl (*E*)-2-benzylidene-3-((dimethoxyphosphoryl)oxy)-5-iodopentanoate (3a*)

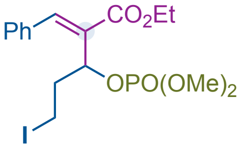

This compound was synthetized following the general procedure **C** using $Rh_2(adc)_4$ (2.7 mg, 0.03 mmol), **2a** and tributyl(methyl)phosphonium dimethyl phosphate (102.7 mg, 0.6 mmol). Purification by flash column chromatography (hexane/ethyl acetate: 2/1) provided the title compound as colourless oil (3.7 mg, 8% yield).

^{1}H NMR (300 MHz, $CDCl_3$) δ 7.87 (s, 1H), 7.47 – 7.34 (m, 5H), 5.55 (ddd, *J* = 9.3, 7.0, 4.0 Hz, 1H), 4.30 (q, *J* = 7.1 Hz, 2H), 3.72 – 3.57 (m, 6H), 3.22 (ddd, *J* = 8.5, 5.9, 2.5 Hz, 2H), 2.93 (ddt, *J* = 15.0, 9.3, 5.9 Hz, 1H), 2.28 (dtt, *J* = 15.2, 7.8, 3.7 Hz, 1H), 1.36 (t, *J* = 7.1 Hz, 3H).

^{13}C NMR (126 MHz, $CDCl_3$) δ 165.9, 143.5, 134.1, 130.6 (d, $J_{C\text{-}P}$ = 2.1 Hz), 129.3, 129.3, 128.8, 74.5 (d, $J_{C\text{-}P}$ = 5.9 Hz), 61.2, 54.5 (d, $J_{C\text{-}P}$ = 10.9 Hz), 38.6 (d, $J_{C\text{-}P}$ = 7.5 Hz), 29.9, 14.4, 1.0.

^{31}P NMR (202 MHz, $CDCl_3$) δ 3.2.

HRMS: (ESI) calculated for $C_{16}H_{22}INaO_6P$ $[M+Na]^+$ m/z: 491.0091, found: 491.0076.

Benzyl (1*R,2*R**,3*S**)-2-(2-((dimethoxyphosphoryl)oxy)ethyl)-1-iodo-3-phenylcyclopropane-1-carboxylate (3b)**

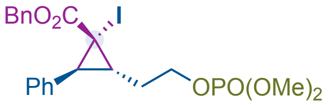

This compound was synthetized following the general procedure **C** using **2b** and tributyl(methyl)phosphonium dimethyl phosphate (103.6 mg, 0.3 mmol). Purification by flash column chromatography (hexane/ethyl acetate: 1/1) provided the title compound as white oil (22.3 mg, 42% yield).

^{1}H NMR (500 MHz, $CDCl_3$) δ 7.27 – 7.24 (m, 3H), 7.21 (tt, J = 3.6, 1.7 Hz, 3H), 7.19 – 7.16 (m, 2H), 7.03 – 6.98 (m, 2H), 4.85 (d, J = 12.3 Hz, 1H), 4.75 (d, J = 12.3 Hz, 1H), 4.27 (q, J = 6.5 Hz, 2H), 3.73 (d, J = 0.9 Hz, 3H), 3.71 (d, J = 0.9 Hz, 3H), 2.69 (d, J = 8.2 Hz, 1H), 2.13 (dqd, J = 13.7, 6.0, 1.2 Hz, 1H), 2.02 (dq, J = 13.9, 6.8 Hz, 1H), 1.92 (ddd, J = 8.2, 7.4, 6.5 Hz, 1H).

^{13}C NMR (126 MHz, $CDCl_3$) δ 167.3, 135.2, 135.0, 128.5, 128.5, 128.2, 128.2, 127.6, 68.2, 66.2 (d, J_{C-P} = 5.7 Hz), 54.5 (d, J_{C-P} = 6.0 Hz), 43.1, 36.4 (d, J_{C-P} = 7.0 Hz), 25.6, 12.5.

^{31}P NMR (202 MHz, $CDCl_3$) δ 4.24.

HRMS: (ESI) calculated for $C_{21}H_{24}INaO_6P$ $[M+Na]^+$ m/z: 553.0247, found: 553.0243.

Benzyl (*E*)-2-benzylidene-3-((dimethoxyphosphoryl)oxy)-5-iodopentanoate (3b*)

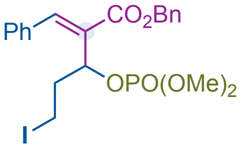

This compound was synthetized following the general procedure **C** using **2b** and tributyl(methyl)phosphonium dimethyl phosphate (103.6 mg, 0.3 mmol). Purification by flash column chromatography (hexane/ethyl acetate: 1/1) provided the title compound as colourless oil (2.5 mg, 5% yield).

^{1}H NMR (400 MHz, $CDCl_3$) δ 7.91 (s, 1H), 7.46 – 7.33 (m, 10H), 5.57 (ddd, J = 9.3, 7.0, 4.0 Hz, 1H), 5.29 (d, J = 2.5 Hz, 2H), 3.62 (d, J = 11.2 Hz, 3H), 3.54 (d, J = 11.2 Hz, 3H), 3.28 – 3.14 (m, 2H), 2.93 (ddt, J = 15.1, 9.3, 5.9 Hz, 1H), 2.35 – 2.21 (m, 1H).

^{13}C NMR (101 MHz, $CDCl_3$) δ 165.7, 144.1, 136.0, 133.9, 130.3 (d, J = 2.2 Hz), 129.4, 129.3, 128.9, 128.8, 128.5, 128.5, 74.4 (d, J_{C-P} = 5.9 Hz), 67.0, 54.5 (d, J_{C-P} = 6.1 Hz), 54.4 (d, J_{C-P} = 6.0 Hz), 38.6 (d, J_{C-P} = 7.5 Hz), 0.9.

^{31}P NMR (162 MHz, $CDCl_3$) δ 3.3.

HRMS: (ESI) calculated for $C_{21}H_{24}INaO_6P$ $[M+Na]^+$ m/z: 553.0247, found: 553.0245.

2,2,2-trichloroethyl (1*R,2*R**,3*S**)-2-(2-((dimethoxyphosphoryl)oxy)ethyl)-1-iodo-3-phenylcyclopropane-1-carboxylate (3c)**

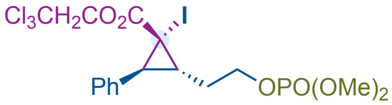

This compound was synthetized following the general procedure **C** using reagent **2c** (68.3 mg, 0.12 mmol) instead of **2a** and tributyl(methyl)phosphonium dimethyl phosphate (103.6 mg, 0.3 mmol). Purification by flash column chromatography (hexane/ethyl acetate: 1/1) provided the title compound as colourless oil (22.9 mg, 40% yield).

^{1}H NMR (500 MHz, $CDCl_3$) δ 7.27 – 7.21 (m, 5H), 4.43 – 4.35 (m, 2H), 4.28 (q, *J* = 6.4 Hz, 2H), 3.76 (dd, *J* = 11.1, 4.4 Hz, 6H), 2.77 (d, *J* = 8.2 Hz, 1H), 2.16 (dqd, *J* = 13.3, 6.1, 1.2 Hz, 1H), 2.10 – 1.95 (m, 2H).

^{13}C NMR (126 MHz, $CDCl_3$) δ 166.1, 134.4, 128.6, 128.6, 94.2, 75.6, 66.0 (d, $J_{C\text{-}P}$ = 5.6 Hz), 54.5 (d, $J_{C\text{-}P}$ = 6.0 Hz), 43.8, 36.3 (d, $J_{C\text{-}P}$ = 7.0 Hz), 26.0, 10.3.

^{31}P NMR (202 MHz, $CDCl_3$) δ 4.3.

HRMS: (ESI) calculated for $C_{16}H_{19}Cl_3INaO_6P$ $[M+Na]^+$ m/z: 592.8922, found: 592.8914.

3.2. Iodocyclopropane scope

General procedure D for nucleophile scope:

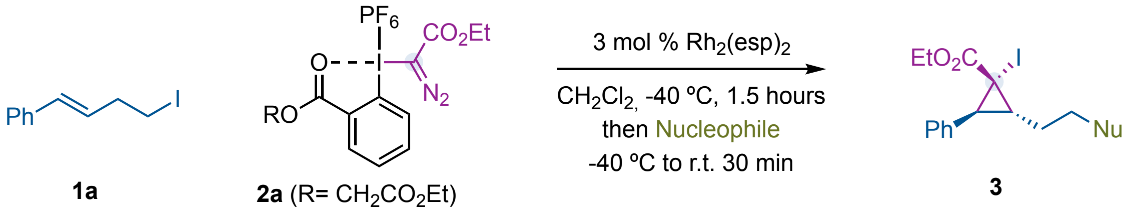

To a 10 mL oven-dried reaction vial equipped with a stirring bar was added $Rh_2(esp)_2$ (4.5 mg, 0.006 mmol, 3 mol%) and homoallylic iodide **1a** (61.9 mg, 0.24 mmol). The tube was sealed before being evacuated and backfilled with argon three times. Dichloromethane (0.5 mL) was added, and the resulting mixture was cooled to -40 ºC. Then, a solution of reagent **2a** (118.4 mg, 0.2 mmol) in dichloromethane (2.0 mL) was added dropwise over 30 minutes using a syringe pump and the reaction mixture was stirred for 60 minutes at the same temperature. After this, a solution of the corresponding nucleophile (0.6–1.0 mmol) in dichloromethane (1.0 mL) was added dropwise over 15 minutes. Then the resulting reaction mixture was allowed to warm to room temperature over 15 minutes followed by the removal of solvent by rotatory evaporation. Purification of the crude residue by flash column chromatography afforded iodocyclopropanes **3a** – **3z**. All diastereomeric ratios were determined to be >20:1 by analysis of ^{1}H NMR of the crude unless otherwise stated. The relative configuration of

3a was assigned by ^1H-^1H 2D NOESY, and analysis of $^3J_{H-H}$ *trans* constants of the cyclopropane in ^1H NMR spectra.

General procedure E for olefin scope:

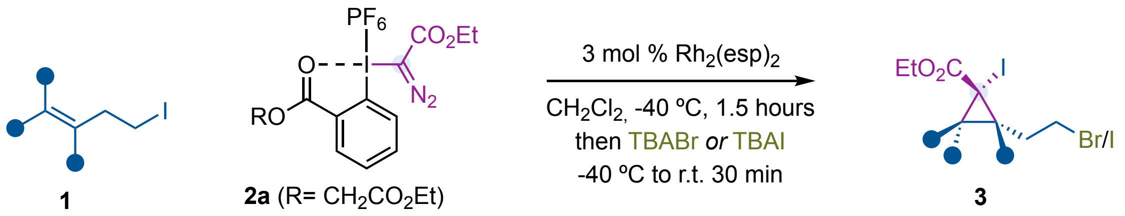

To a 10 mL oven-dried reaction vial equipped with a stirring bar was added $Rh_2(esp)_2$ (4.5 mg, 0.006 mmol, 3 mol%) and homoallylic iodide **1** (0.24 mmol). The tube was sealed before being evacuated and backfilled with argon three times. Dichloromethane (0.5 mL) was added, and the resulting mixture was cooled to -40 ºC. Then, a solution of reagent **2a** (118.4 mg, 0.2 mmol) in dichloromethane (2.0 mL) was added dropwise over 30 minutes using a syringe pump and the reaction mixture was stirred for 60 minutes at the same temperature. After this, a solution of the tetrabutylammonium bromide (96.7 mg, 0.3 mmol) or tetrabutylammonium iodide (119.8 mg, 0.3 mmol) in dichloromethane (1.0 mL) was added dropwise over 15 minutes. Then the resulting reaction mixture was allowed to warm to room temperature over 15 minutes followed by the removal of solvent by rotatory evaporation. Purification of the crude residue by flash column chromatography afforded iodocyclopropanes **3aa** – **3bb**. All diastereomeric ratios were determined to be >20:1 by analysis of ^1H NMR of the crude unless otherwise stated. The relative configuration of the compounds was assigned by ^1H-^1H 2D NOESY,

General procedure F for hypervalent iodine reagent scope:

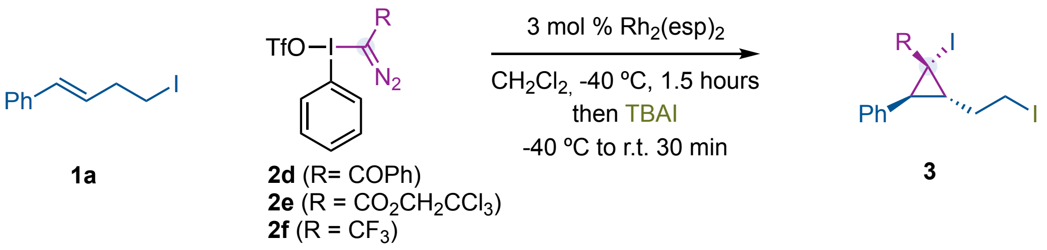

To a 10 mL oven-dried reaction vial equipped with a stirring bar was added $Rh_2(esp)_2$ (4.5 mg, 0.006 mmol, 3 mol%) and homoallylic iodide **1a** (61.9 mg, 0.24 mmol). The tube was sealed before being evacuated and backfilled with argon three times. Dichloromethane (0.5 mL) was added, and the resulting mixture was cooled to -40 ºC. Then, a solution of reagent **2d/2e/2f** (0.2 mmol) in dichloromethane (2.0 mL) was added dropwise over 30 minutes using a syringe pump and the reaction mixture was stirred for 60 minutes at the same temperature. After this, a solution of tetrabutylammonium iodide (119.8 mg, 0.3 mmol) in dichloromethane (1.0 mL) was added dropwise

over 15 minutes. Then the resulting reaction mixture was allowed to warm to room temperature over 15 minutes followed by the removal of solvent by rotatory evaporation. Purification of the crude residue by flash column chromatography afforded iodocyclopropanes **3bc** – **3be**. All diastereomeric ratios were determined to be >20:1 by analysis of ^{1}H NMR of the crude unless otherwise stated. The relative configuration of the compounds was assigned by analogy of compound **3j**, and analysis of $^3J_{H\text{-}H}$ *trans* constants of the cyclopropane in ^{1}H NMR spectra.

Ethyl (1*R,2*R**,3*S**)-2-(2-((dimethoxyphosphoryl)oxy)ethyl)-1-iodo-3-phenylcyclopropane-1-carboxylate (3a)**

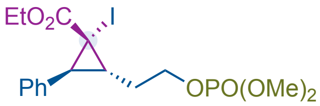

This compound was synthetized following the general procedure **D** using tributyl(methyl)phosphonium dimethyl phosphate (207.2 mg, 0.6 mmol). Purification by flash column chromatography (hexane/ethyl acetate: 1/1) provided the title compound as colourless oil (67.2 mg, 72% yield).

^{1}H NMR (500 MHz, MeOD) δ 7.31 – 7.18 (m, 5H), 4.29 (qd, *J* = 5.9, 1.1 Hz, 2H), 3.85 – 3.78 (m, 2H), 3.77 (d, *J* = 3.5 Hz, 3H), 3.74 (d, *J* = 3.5 Hz, 3H), 2.71 (d, *J* = 8.0 Hz, 1H), 2.11 (dtd, *J* = 13.8, 6.2, 1.4 Hz, 1H), 2.08 – 2.00 (m, 1H), 1.91 (dt, *J* = 8.0, 6.9 Hz, 1H), 0.83 (t, *J* = 7.1 Hz, 3H).

^{13}C NMR (126 MHz, MeOD) δ 169.0, 136.7, 129.4, 129.3, 128.4, 67.8 (d, $J_{C\text{-}P}$ = 6.0 Hz), 63.2, 55.2 (d, $J_{C\text{-}P}$ = 6.1 Hz), 43.5, 37.1 (d, $J_{C\text{-}P}$ = 7.2 Hz), 26.2, 14.0, 12.7.

^{31}P NMR (202 MHz, MeOD) δ 3.9.

HRMS: (ESI) calculated for $C_{16}H_{23}IO_6P$ $[M+H]^+$ m/z: 469.0271, found: 469.0268.

^{1}H-^{1}H NOESY, ^{1}H-^{13}C HSQC and ^{1}H-^{13}C HMBC were recorded.

Ethyl (1*R,2*R**,3*S**)-1-iodo-2-(2-(nitrooxy)ethyl)-3-phenylcyclopropane-1-carboxylate (3d)**

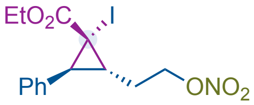

This compound was synthetized following the general procedure **D** using tetrabutylammonium nitrate (109.6 mg, 0.6 mmol). Purification by flash column chromatography (hexane/ethyl acetate: 100/2) provided the title compound as white oil (68.1 mg, 84% yield).

^{1}H NMR (500 MHz, $CDCl_3$) δ 7.29 – 7.26 (m, 2H), 7.26 – 7.23 (m, 1H), 7.21 – 7.15 (m, 2H), 4.67 (td, *J* = 6.5, 2.1 Hz, 2H), 3.95 – 3.78 (m, 2H), 2.66 (d, *J* = 8.1 Hz, 1H), 2.21 – 2.05 (m, 2H), 1.87 (dt, *J* = 8.1, 7.2 Hz, 1H), 0.86 (t, *J* = 7.1 Hz, 3H).

^{13}C NMR (126 MHz, $CDCl_3$) δ 167.1, 134.7, 128.5, 128.4, 127.7, 71.4, 62.6, 42.6, 33.2, 25.1, 13.7, 12.8.

HRMS: (ESI) calculated for $C_{14}H_{16}INNaO_5$ $[M+Na]^+$ m/z: 427.9965, found: 427.9974.

Ethyl (1*R,2*R**,3*S**)-1-iodo-2-(2-((methylsulfonyl)oxy)ethyl)-3-phenylcyclopropane-1-carboxylate (3e)**

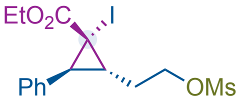

This compound was synthetized following the general procedure **D** using tetrabutylammonium methanesulfonate (202.5 mg, 0.6 mmol). Purification by flash column chromatography (hexane/ethyl acetate: 20/1 → 5/1) provided the title compound as pale-yellow oil (51.7 mg, 59% yield).

^{1}H NMR (400 MHz, $CDCl_3$) δ 7.30 – 7.26 (m, 1H), 7.26 – 7.23 (m, 2H), 7.22 – 7.17 (m, 2H), 4.44 (td, *J* = 6.3, 0.9 Hz, 2H), 3.93 – 3.76 (m, 2H), 3.01 (s, 3H), 2.67 (d, *J* = 8.1 Hz, 1H), 2.20 – 2.09 (m, 2H), 1.90 (dt, *J* = 8.1, 7.0 Hz, 1H), 0.85 (t, *J* = 7.1 Hz, 3H).

^{13}C NMR (101 MHz, $CDCl_3$) δ 167.3, 134.8, 128.4, 128.4, 127.7, 67.9, 62.5, 42.6, 37.6, 35.2, 25.0, 13.7, 12.7.

HRMS: (ESI) calculated for $C_{15}H_{19}INaO_5S$ $[M+Na]^+$ m/z: 460.9890, found: 460.9885.

Ethyl (1*R,2*S**,3*R**)-1-iodo-2-phenyl-3-(2-(((trifluoromethyl)sulfonyl)oxy)ethyl)cyclopropane-1-carboxylate (3f)**

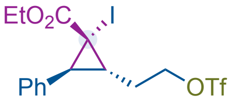

This compound was synthetized following the general procedure **D** using tetrabutylammonium trifluoromethanesulfonate (234.9 mg, 0.6 mmol). Purification by flash column chromatography (hexane/ethyl acetate: 100/2) provided the title compound as white oil (60.1 mg, 61% yield).

^{1}H NMR (400 MHz, $CDCl_3$) δ 7.31 – 7.26 (m, 2H), 7.26 – 7.22 (m, 1H), 7.21 – 7.17 (m, 2H), 4.74 (t, *J* = 6.4 Hz, 2H), 3.93 – 3.78 (m, 2H), 2.69 (d, *J* = 8.1 Hz, 1H), 2.25 (q, *J* = 6.6 Hz, 2H), 1.91 (dt, *J* = 8.1, 7.1 Hz, 1H), 0.86 (t, *J* = 7.1 Hz, 3H).

^{13}C NMR (101 MHz, $CDCl_3$) δ 166.9, 134.4, 128.5, 128.4, 127.8, 118.8 (q, *J* = 320.1 Hz), 75.2, 62.6, 42.5, 35.4, 24.4, 13.7, 12.4.

^{19}F NMR (376 MHz, $CDCl_3$) δ -74.6.

HRMS: (ESI) calculated for $C_{15}H_{16}F_3INaO_3S$ $[M+Na]^+$ m/z: 514.9715, found: 514.9711.

Ethyl (1*R,2*R**,3*S**)-2-(2-fluoroethyl)-1-iodo-3-phenylcyclopropane-1-carboxylate (3g)**

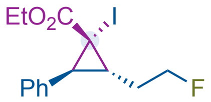

This compound was synthetized following the general procedure **D** using tetrabutylammonium fluoride (156.9 mg, 0.6 mmol). Purification by flash column chromatography (hexane/ethyl acetate: 99/1 – 98/2) provided the title compound as pale-yellow oil (45.6 mg, 63% yield).

^{1}H NMR (400 MHz, $CDCl_3$) δ 7.31 – 7.18 (m, 5H), 4.79 – 4.67 (m, 1H), 4.64 – 4.55 (m, 1H), 3.91 – 3.78 (m, 2H), 2.67 (d, *J* = 7.8 Hz, 1H), 2.30 – 2.09 (m, 1H), 2.06 – 1.89 (m, 2H), 0.85 (t, *J* = 7.1 Hz, 3H).

^{13}C NMR (101 MHz, $CDCl_3$) δ 167.4, 135.2, 128.6, 128.4, 127.5, 82.5 (d, *J* = 167.0 Hz), 62.4, 42.8, 36.6 (d, *J* = 19.8 Hz), 25.3 (d, *J* = 6.3 Hz), 13.8, 13.4.

^{19}F NMR (376 MHz, $CDCl_3$) δ -180.1.

HRMS: (ESI) calculated for $C_{14}H_{17}FIO_2$ $[M+H]^+$ m/z: 363.0252, found: 363.0249.

Ethyl (1*R,2*R**,3*S**)-2-(2-chloroethyl)-1-iodo-3-phenylcyclopropane-1-carboxylate (3h)**

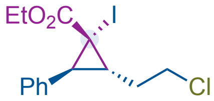

This compound was synthetized following the general procedure **D** using tetrabutylammonium chloride (166.8 mg, 0.6 mmol). Purification by flash column chromatography (hexane/ethyl acetate: 100/1 → 20/1) provided the title compound as pale-yellow oil (61.9 mg, 82% yield).

^{1}H NMR (500 MHz, $CDCl_3$) δ 7.31 – 7.20 (m, 5H), 3.91 – 3.78 (m, 2H), 3.75 (t, *J* = 6.5 Hz, 2H), 2.69 (d, *J* = 8.1 Hz, 1H), 2.25 (dq, *J* = 14.5, 6.3 Hz, 1H), 2.09 (dq, *J* = 14.2, 6.9 Hz, 1H), 1.98 (ddd, *J* = 8.1, 7.5, 6.4 Hz, 1H), 0.85 (t, *J* = 7.1 Hz, 3H).

^{13}C NMR (126 MHz, $CDCl_3$) δ 167.3, 135.1, 128.6, 128.4, 127.6, 62.5, 43.1, 42.9, 38.7, 26.5, 13.8, 13.0.

HRMS: (ESI) calculated for $C_{14}H_{16}ClINaO_2$ $[M+Na]^+$ m/z: 400.9776, found: 400.9763.

Ethyl (1*R,2*R**,3*S**)-2-(2-bromoethyl)-1-iodo-3-phenylcyclopropane-1-carboxylate (3i)**

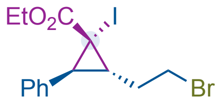

This compound was synthetized following the general procedure **D** using tetrabutylammonium bromide (193.4 mg, 0.6 mmol). Purification by flash column chromatography (hexane/ethyl acetate: 100/1 → 20/1) provided the title compound as pale-yellow oil (73.6 mg, 87% yield).

^{1}H NMR (500 MHz, $CDCl_3$) δ 7.33 – 7.20 (m, 5H), 3.90 – 3.78 (m, 2H), 3.60 (td, *J* = 6.5, 0.8 Hz, 2H), 2.69 (d, *J* = 8.1 Hz, 1H), 2.33 (dq, *J* = 14.5, 6.5 Hz, 1H), 2.19 (dq, *J* = 14.5, 7.0 Hz, 1H), 2.03 – 1.90 (m, 1H), 0.85 (t, *J* = 7.1 Hz, 3H).

^{13}C NMR (126 MHz, $CDCl_3$) δ 167.3, 135.0, 128.6, 128.4, 127.6, 62.5, 42.8, 38.8, 31.0, 27.5, 13.8, 12.8.

HRMS: (ESI) calculated for $C_{14}H_{16}BrINaO_2$ $[M+Na]^+$ m/z: 444.9271, found: 444.9262.

Ethyl (1*R,2*R**,3*S**)-1-iodo-2-(2-iodoethyl)-3-phenylcyclopropane-1-carboxylate (3j)**

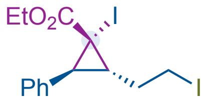

This compound was synthetized following the general procedure **D** using tetrabutylammonium iodide (226.8 mg, 0.6 mmol). Purification by flash column chromatography (hexane/ethyl acetate: 100/1 → 50/1) provided the title compound as brown oil (79.9 mg, 85% yield).

^{1}H NMR (400 MHz, $CDCl_3$) δ 7.33 – 7.19 (m, 5H), 3.84 (m, 2H), 3.43 – 3.29 (m, 2H), 2.68 (d, *J* = 8.1 Hz, 1H), 2.38 – 2.14 (m, 2H), 1.94 (dt, *J* = 8.1, 6.8 Hz, 1H), 0.85 (t, *J* = 7.1 Hz, 3H).

^{13}C NMR (101 MHz, $CDCl_3$) δ 167.3, 135.0, 128.6, 128.4, 127.6, 62.5, 42.7, 39.6, 29.5, 13.8, 12.6, 2.7.

HRMS: (ESI) calculated for $C_{14}H_{16}I_2NaO_2$ $[M+Na]^+$ m/z: 492.9132, found: 492.9147.

Ethyl (1*R,2*R**,3*S**)-2-(2-cyanoethyl)-1-iodo-3-phenylcyclopropane-1-carboxylate (3k)**

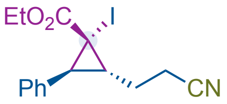

This compound was synthetized following the general procedure **D** using tetrabutylammonium cyanide (168.1 mg, 0.6 mmol). Purification by flash column chromatography (hexane/ethyl acetate: 100/0 → 10/1) provided the title compound as colourless oil (39.9 mg, 54% yield).

^{1}H NMR (400 MHz, $CDCl_3$) δ 7.33 – 7.23 (m, 3H), 7.21 (ddd, *J* = 7.2, 1.9, 0.8 Hz, 2H), 3.93 – 3.76 (m, 2H), 2.69 (d, *J* = 8.0 Hz, 1H), 2.61 (td, *J* = 7.0, 3.7 Hz, 2H), 2.19 – 1.97 (m, 2H), 1.90 (dt, *J* = 8.0, 7.2 Hz, 1H), 0.85 (t, *J* = 7.1 Hz, 3H).

^{13}C NMR (101 MHz, $CDCl_3$) δ 167.0, 134.5, 128.5, 128.4, 127.7, 118.9, 62.6, 42.7, 31.8, 27.1, 16.1, 13.7, 12.7.

HRMS: (ESI) calculated for $C_{15}H_{16}INNaO_2$ $[M+Na]^+$ m/z: 392.0118, found: 392.0110.

Ethyl (1*R,2*R**,3*S**)-2-(2-azidoethyl)-1-iodo-3-phenylcyclopropane-1-carboxylate (3l)**

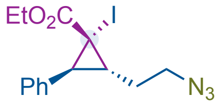

This compound was synthetized following the general procedure **D** using tetrabutylammonium azide (170.7 mg, 0.6 mmol). Purification by flash column chromatography (hexane/ethyl acetate: 50/1) provided the title compound as colourless oil (63.2 mg, 82% yield).

^{1}H NMR (500 MHz, $CDCl_3$) δ 7.30 – 7.23 (m, 3H), 7.22 – 7.19 (m, 2H), 3.92 – 3.77 (m, 2H), 3.61 – 3.47 (m, 2H), 2.66 (d, *J* = 8.1 Hz, 1H), 2.06 – 1.90 (m, 2H), 1.85 (dt, *J* = 8.1, 7.0 Hz, 1H), 0.85 (t, *J* = 7.1 Hz, 3H).

^{13}C NMR (126 MHz, $CDCl_3$) δ 167.4, 135.0, 128.5, 128.4, 127.6, 62.5, 50.2, 42.8, 35.1, 26.1, 13.8, 13.3.

HRMS: (ESI) calculated for $C_{14}H_{16}IN_3NaO_2$ $[M+Na]^+$ m/z: 408.0179, found: 408.01859.

2-((1*R,2*R**,3*S**)-2-(ethoxycarbonyl)-2-iodo-3-phenylcyclopropyl)ethyl benzoate (3m)**

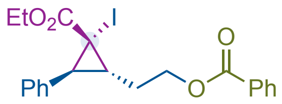

This compound was synthetized following the general procedure **D** using tetrabutylammonium benzoateonate (218.2 mg, 0.6 mmol). Purification by flash column chromatography (hexane/ethyl acetate: 20/1 → 15/1) provided the title compound as pale-yellow oil (69.5 mg, 75% yield).

^{1}H NMR (500 MHz, $CDCl_3$) δ 8.05 – 7.99 (m, 2H), 7.57 – 7.51 (m, 1H), 7.45 – 7.36 (m, 2H), 7.25 – 7.18 (m, 5H), 4.63 – 4.51 (m, 2H), 3.92 – 3.77 (m, 2H), 2.70 (d, J = 8.1 Hz, 1H), 2.25 (dq, J = 14.5, 6.2 Hz, 1H), 2.08 (dq, J = 14.5, 7.2 Hz, 1H), 1.95 (td, J = 7.9, 6.2 Hz, 1H), 0.86 (t, J = 7.1 Hz, 3H).

^{13}C NMR (126 MHz, $CDCl_3$) δ 167.5, 166.7, 135.1, 133.1, 130.2, 129.8, 128.5, 128.5, 128.4, 127.5, 63.5, 62.4, 43.0, 35.0, 25.9, 13.8, 13.1.

HRMS: (ESI) calculated for $C_{21}H_{21}INaO_4$ $[M+Na]^+$ m/z: 487.0377, found: 487.0379.

Ethyl (1*R,2*R**,3*S**)-1-iodo-2-(2-methoxyethyl)-3-phenylcyclopropane-1-carboxylate (3n)**

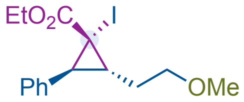

This compound was synthetized following the general procedure **D** using methanol (0.1 mL, 2.5 mmol). Purification by flash column chromatography (hexane/ethyl acetate: 100/2) provided the title compound as white oil (71.1 mg, 95% yield).

^{1}H NMR (500 MHz, $CDCl_3$) δ 7.26 – 7.19 (m, 5H), 3.83 (m, 2H), 3.64 – 3.55 (m, 2H), 3.40 (s, 3H), 2.64 (d, J = 7.6 Hz, 1H), 2.08 – 1.95 (m, 1H), 1.92 – 1.81 (m, 2H), 0.84 (t, J = 7.1 Hz, 3H).

^{13}C NMR (126 MHz, $CDCl_3$) δ 167.7, 135.7, 128.6, 128.3, 127.4, 71.2, 62.3, 59.0, 43.0, 35.9, 26.2, 14.1, 13.8.

HRMS: (ESI) calculated for $C_{15}H_{19}INaO_3$ $[M+Na]^+$ m/z: 397.0271, found: 397.0276.

(2-((1*R,2*R**,3*S**)-2-(ethoxycarbonyl)-2-iodo-3-phenylcyclopropyl)ethyl)diethyloxonium hexafluorophosphate (3o)**

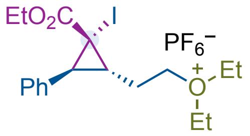

This compound was synthetized following the general procedure **D** using diethyl ether (2.0 mL, 19 mmol) in slow addition at -40 ºC. The precipitate formed was filtrated and washed with cold diethyl ether to yield the title product as off white amorphous solid (33.7 mg, 30% yield). *NMR characterization was performed at -40 ºC due to instability of the product in solution at room temperature.*

^{1}H NMR (500 MHz, CD_2Cl_2) δ 7.32 – 7.21 (m, 3H), 7.20 – 7.14 (m, 2H), 4.83 (q, *J* = 7.1 Hz, 4H), 4.80 – 4.76 (m, 2H), 3.88 – 3.72 (m, 2H), 2.74 (d, *J* = 7.9 Hz, 1H), 2.60 (dtd, *J* = 15.3, 7.5, 4.6 Hz, 1H), 2.06 (ddt, *J* = 15.3, 9.4, 6.3 Hz, 1H), 1.80 (ddd, *J* = 9.4, 7.9, 4.6 Hz, 1H), 1.65 (t, *J* = 7.1 Hz, 6H), 0.78 (t, *J* = 7.1 Hz, 3H).

^{13}C NMR (126 MHz, CD_2Cl_2) δ 166.6, 133.7, 128.4, 128.1, 127.8, 86.0, 84.6, 62.8, 41.4, 32.0, 23.0, 13.4, 12.6, 11.6.

^{31}P NMR (202 MHz, CD_2Cl_2) δ -141.6.

HRMS: (ESI) calculated for $C_{18}H_{26}IO_3$ $[M\text{-}PF_6]^+$ m/z: 417.0921, found: 417.0914.

Ethyl (1*R,2*S**,3*R**)-1-iodo-2-phenyl-3-(2-((1-phenyl-1*H*-tetrazol-5-yl)thio)ethyl)cyclopropane-1-carboxylate (3p)**

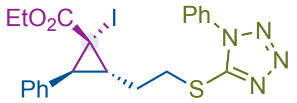

This compound was synthetized following the general procedure **D** using 1-phenyl-1*H*-tetrazole-5-thiol (106.9 mg, 0.6 mmol). Purification by flash column chromatography (hexane/ethyl acetate: 10/1 → 8/1) provided the title compound as pale-yellow oil (83.3 mg, 80% yield).

^{1}H NMR (400 MHz, $CDCl_3$) δ 7.53 (s, 5H), 7.25 – 7.20 (m, 3H), 7.18 – 7.13 (m, 2H), 3.92 – 3.75 (m, 2H), 3.73 – 3.56 (m, 2H), 2.66 (d, *J* = 8.1 Hz, 1H), 2.35 – 2.16 (m, 2H), 1.94 (dt, *J* = 8.1, 6.9 Hz, 1H), 0.84 (t, *J* = 7.1 Hz, 3H).

^{13}C NMR (101 MHz, $CDCl_3$) δ 167.3, 154.2, 134.8, 133.6, 130.3, 129.9, 129.4, 128.4, 128.3, 127.5, 123.9, 123.9, 62.5, 42.9, 35.2, 31.6, 27.4, 13.7, 13.0.

HRMS: (ESI) calculated for $C_{21}H_{21}IN_4NaO_2S$ $[M+Na]^+$ m/z: 543.0322, found: 543.0314.

Ethyl (1*R,2*R**,3*S**)-1-iodo-2-(2-morpholinoethyl)-3-phenylcyclopropane-1-carboxylate (3q)**

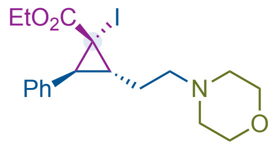

This compound was synthetized following the general procedure **D** using morpholine (52.3 mg, 0.6 mmol). Purification by flash column chromatography using neutral silica (hexane/ethyl acetate: 1/1) provided the title compound as colourless oil (56.7 mg, 66% yield).

^{1}H NMR (500 MHz, $CDCl_3$) δ 7.28 – 7.20 (m, 5H), 3.91 – 3.78 (m, 2H), 3.78 – 3.70 (m, 4H), 2.68 – 2.57 (m, 3H), 2.53 (bs, 4H), 1.99 – 1.88 (m, 1H), 1.86 – 1.75 (m, 2H), 0.85 (t, *J* = 7.1 Hz, 3H).

^{13}C NMR (126 MHz, $CDCl_3$) δ 167.7, 135.6, 128.6, 128.3, 127.4, 67.1, 62.3, 57.5, 53.9, 43.0, 32.7, 27.1, 14.0, 13.8.

HRMS: (ESI) calculated for $C_{18}H_{25}INO_3$ $[M+H]^+$ m/z: 430.0874, found: 430.0886.

Ethyl (1*R,2*R**,3*S**)-2-(2-(diphenylamino)ethyl)-1-iodo-3-phenylcyclopropane-1-carboxylate (3r)**

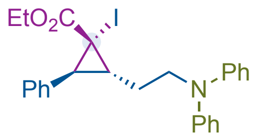

This compound was synthetized following the general procedure **D** using diphenylamine (101.5 mg, 0.6 mmol). Purification by flash column chromatography (hexane/ethyl acetate: 25/1) provided the title compound as colourless oil (37.8 mg, 37% yield).

^{1}H NMR (500 MHz, $CDCl_3$) δ 7.32 – 7.27 (m, 4H), 7.24 (td, *J* = 5.9, 2.6 Hz, 3H), 7.16 – 7.12 (m, 2H), 7.10 – 7.06 (m, 4H), 6.98 (tt, *J* = 7.4, 1.1 Hz, 2H), 4.05 (ddd, *J* = 14.9, 9.1, 6.0 Hz, 1H), 3.94 (ddd, *J* = 14.9, 9.1, 6.0 Hz, 1H), 3.90 – 3.77 (m, 2H), 2.63 (d, *J* = 8.1 Hz, 1H), 2.16 – 1.99 (m, 2H), 1.83 (dt, *J* = 8.1, 7.0 Hz, 1H), 0.85 (t, *J* = 7.1 Hz, 3H).

^{13}C NMR (101 MHz, $CDCl_3$) δ 167.5, 147.9, 135.2, 129.5, 128.5, 128.3, 127.4, 121.6, 121.2, 62.3, 50.7, 42.9, 33.8, 26.7, 13.9, 13.7.

HRMS: (ESI) calculated for $C_{26}H_{27}INO_2$ $[M+H]^+$ m/z: 512.1081, found: 512.1076.

Ethyl (1*R,2*R**,3*S**)-2-(2-(dibenzylamino)ethyl)-1-iodo-3-phenylcyclopropane-1-carboxylate (3s)**

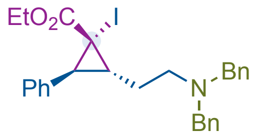

This compound was synthetized following the general procedure **D** using dibenzylamine (118.4 mg, 0.6 mmol). Purification by flash column chromatography (hexane/ethyl acetate: 100/2) provided the title compound as white oil (72.3 mg, 67% yield).

^{1}H NMR (400 MHz, $CDCl_3$) δ 7.43 – 7.36 (m, 4H), 7.33 – 7.27 (m, 4H), 7.25 – 7.19 (m, 5H), 7.15 – 7.10 (m, 2H), 3.92 – 3.75 (m, 2H), 3.71 – 3.58 (m, 4H), 2.70 (td, *J* = 6.8, 1.5 Hz, 2H), 2.55 (d, *J* = 7.8 Hz, 1H), 1.93 – 1.79 (m, 3H), 0.86 (t, *J* = 7.1 Hz, 3H).

^{13}C NMR (101 MHz, $CDCl_3$) δ 167.7, 139.8, 135.6, 128.9, 128.6, 128.4, 128.2, 127.3, 127.0, 62.2, 58.7, 52.1, 43.3, 33.6, 26.8, 14.5, 13.8.

HRMS: (ESI) calculated for $C_{28}H_{31}INO_2$ $[M+H]^+$ m/z: 540.1394, found: 540.1402.

2-((1*R,2*R**,3*S**)-2-(ethoxycarbonyl)-2-iodo-3-phenylcyclopropyl)-*N*,*N*,*N*-triethylethan-1-aminium hexafluorophosphate (3t)**

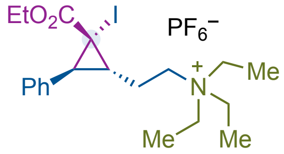

This compound was synthetized following the general procedure **D** using triethylamine (60.7 mg, 0.6 mmol). Purification by flash column chromatography (dichloromethane/methanol: 50/1) provided the title compound as white solid (73.1 mg, 62% yield).

m.p. = 165 – 166 ºC

^{1}H NMR (400 MHz, $CDCl_3$) δ 7.22 (dd, *J* = 8.2, 5.7 Hz, 5H), 3.89 – 3.73 (m, 2H), 3.40 – 3.16 (m, 8H), 2.71 (d, *J* = 7.1 Hz, 1H), 2.37 – 2.23 (m, 1H), 1.86 – 1.73 (m, 2H), 1.34 (t, *J* = 7.2 Hz, 10H), 0.82 (t, *J* = 7.1 Hz, 3H).

^{13}C NMR (101 MHz, $CDCl_3$) δ 166.9, 134.2, 128.6, 128.5, 127.8, 62.8, 55.1, 53.5, 42.1, 27.9, 25.2, 13.6, 13.2, 7.7.

^{19}F NMR (376 MHz, $CDCl_3$) δ -72.2 (d, *J* = 712.4 Hz).

^{31}P NMR (162 MHz, $CDCl_3$) δ -141.3 (hept, *J* = 712.4 Hz).

HRMS: (ESI) calculated for $C_{20}H_{31}INO_2$ $[M-PF_6]^+$ m/z: 444.1394, found: 444.1399.

Ethyl (1*R,2*R**,3*S**)-1-iodo-2-(2-(2-oxopyridin-1(2*H*)-yl)ethyl)-3-phenylcyclopropane-1-carboxylate (3u)**

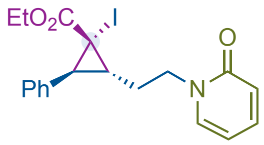

This compound was synthetized following the general procedure **D** using pyridin-2-ol (57.1 mg, 0.6 mmol). Purification by flash column chromatography (hexane/dichloromethane: 1/2) provided the title compound as colourless oil (36.7 mg, 42% yield).

^{1}H NMR (400 MHz, $CDCl_3$) δ 8.15 (ddd, *J* = 5.1, 2.1, 0.8 Hz, 1H), 7.56 (ddd, *J* = 8.3, 7.1, 2.1 Hz, 1H), 7.25 – 7.18 (m, 5H), 6.86 (ddd, *J* = 7.1, 5.1, 1.0 Hz, 1H), 6.74 (dt, *J* = 8.3, 0.9 Hz, 1H), 4.54 (t, *J* = 6.4 Hz, 2H), 3.91 – 3.74 (m, 2H), 2.70 (d, *J* = 8.0 Hz, 1H), 2.25 (dq, *J* = 14.0, 6.1 Hz, 1H), 2.16 – 2.01 (m, 1H), 1.97 (td, *J* = 8.0, 6.1 Hz, 1H), 0.84 (t, *J* = 7.1 Hz, 3H).

^{13}C NMR (101 MHz, $CDCl_3$) δ 167.6, 163.8, 146.9, 138.8, 135.5, 128.6, 128.3, 127.4, 116.9, 111.3, 64.3, 62.3, 43.1, 35.4, 26.3, 13.8, 13.7.

HRMS: (ESI) calculated for $C_{19}H_{21}INO_3$ $[M+H]^+$ m/z 438.0561, found: 438.0544.

Ethyl (1*R,2*R**,3*S**)-2-(2-benzamidoethyl)-1-iodo-3-phenylcyclopropane-1-carboxylate (3v)**

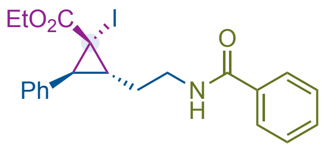

This compound was synthetized following the general procedure **D** using benzamide (72.7 mg, 0.6 mmol). Purification by flash column chromatography (hexane/ethyl acetate/triethylamine: 75/25/1) provided the title compound as colourless oil (57.5 mg, 62% yield).

^{1}H NMR (400 MHz, $CDCl_3$) δ 7.74 – 7.67 (m, 2H), 7.48 – 7.41 (m, 1H), 7.41 – 7.33 (m, 2H), 7.25 – 7.18 (m, 5H), 4.53 (td, *J* = 6.3, 1.4 Hz, 2H), 3.84 (qq, *J* = 10.7, 7.1 Hz, 2H), 2.72 (d, *J* = 8.1 Hz, 1H), 2.29 (dq, *J* = 14.2, 6.0 Hz, 1H), 2.18 – 2.05 (m, 1H), 2.00 (td, *J* = 8.1, 6.1 Hz, 1H), 0.85 (t, *J* = 7.1 Hz, 3H).

^{13}C NMR (101 MHz, $CDCl_3$) δ 168.0, 167.5, 135.3, 132.6, 131.0, 128.6, 128.5, 128.3, 127.4, 126.8, 64.7, 62.3, 43.1, 35.0, 26.2, 13.7, 13.4.

HRMS: (ESI) calculated for $C_{21}H_{23}INO_3$ $[M+H]^+$ m/z: 464.0717, found: 464.0724.

1-(2-((1*R,2*R**,3*S**)-2-(ethoxycarbonyl)-2-iodo-3-phenylcyclopropyl)ethyl)imidazo[1,2-*a*]pyridin-1-ium hexafluorophosphate (3w)**

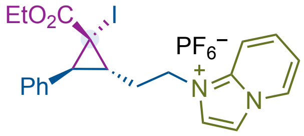

This compound was synthetized following the general procedure **D** using pyrimidazole (56.9 mg, 0.6 mmol). Purification by flash column chromatography (dichloromethane/methanol: 25/1) provided the title compound as pale brown solid (81.2 mg, 67% yield).

m.p. = 178 – 180ºC

^{1}H NMR (500 MHz, $(CD_3)_2CO$) δ 8.94 (dt, *J* = 6.8, 1.1 Hz, 1H), 8.43 (dd, *J* = 2.3, 0.7 Hz, 1H), 8.40 – 8.36 (m, 1H), 8.35 (d, *J* = 2.3 Hz, 1H), 8.09 (ddd, *J* = 9.2, 7.1, 1.1 Hz, 1H), 7.60 (td, *J* = 6.9, 1.0 Hz, 1H), 7.28 – 7.18 (m, 3H), 7.12 – 7.07 (m, 2H), 4.98 (t, *J* = 6.9 Hz, 2H), 3.86 – 3.74 (m, 2H), 2.63 (d, *J* = 8.0 Hz, 1H), 2.51 (dq, *J* = 13.8, 6.9 Hz, 1H), 2.39 (dq, *J* = 14.0, 6.8 Hz, 1H), 1.97 (dt, *J* = 8.0, 6.9 Hz, 1H), 0.80 (t, *J* = 7.1 Hz, 3H).

^{13}C NMR (126 MHz, $(CD_3)_2CO$) δ 167.8, 140.7, 135.9, 135.0, 130.8, 129.1, 128.9, 128.2, 126.8, 118.6, 116.6, 112.3, 62.7, 47.1, 42.2, 36.0, 26.3, 14.0, 12.8.

^{31}P NMR (162 MHz, $(CD_3)_2CO$) δ -130.4 – -152.6 (m).

HRMS: (ESI) calculated for $C_{21}H_{22}IN_2O_2$ $[M-PF_6]^+$ m/z: 461.0721, found: 461.0711.

The crystal structure of **3w** has been deposited at the Cambridge Crystallographic Data Centre, **CCDC** 2407009

Ethyl (1*R,2*R**,3*S**)-1-iodo-2-(4-methoxyphenethyl)-3-phenylcyclopropane-1-carboxylate (3x)**

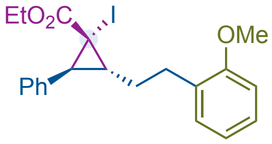

This compound was synthetized following the general procedure **D** using anisole (64.9 mg, 0.6 mmol). Purification by flash column chromatography (hexane/ethyl acetate: 100/0 → 100/3) provided the title compound as pale-yellow oil (59.4 mg, 66% yield).

^{1}H NMR (500 MHz, $CDCl_3$) δ 7.25 – 7.15 (m, 7H), 6.89 (td, *J* = 7.4, 1.1 Hz, 1H), 6.85 (dd, *J* = 8.7, 1.1 Hz, 1H), 3.91 – 3.75 (m, 5H), 2.88 (dqt, *J* = 13.5, 8.7, 4.7 Hz, 2H), 2.60 (d, *J* = 8.2 Hz, 1H), 1.96 (ddq, *J* = 19.5, 9.0, 6.9 Hz, 2H), 1.78 (dt, *J* = 8.2, 7.0 Hz, 1H), 0.85 (t, *J* = 7.1 Hz, 3H).

^{13}C NMR (126 MHz, $CDCl_3$) δ 167.8, 157.6, 135.8, 130.2, 129.8, 128.5, 128.2, 127.5, 127.3, 120.5, 110.4, 62.2, 55.3, 43.2, 36.1, 29.1, 28.6, 14.6, 13.8.

HRMS: (ESI) calculated for $C_{21}H_{23}INaO_3$ $[M+Na]^+$ m/z: 473.0584, found: 473.0591.

Ethyl (1*R,2*S**,3*R**)-1-iodo-2-phenyl-3-(2,4,6-trimethylphenethyl)cyclopropane-1-carboxylate (3y)**

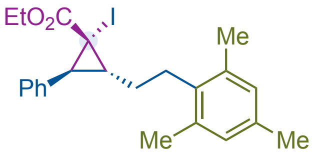

This compound was synthetized following the general procedure **D** using mesitylene (83 μL, 0.6 mmol). Purification by flash column chromatography (hexane/ethyl acetate: 100/1 → 50/1) provided the title compound as colourless oil (86.0 mg, 93% yield).

^{1}H NMR (400 MHz, $CDCl_3$) δ 7.33 – 7.18 (m, 5H), 6.86 (s, 2H), 3.96 – 3.78 (m, 2H), 2.97 (ddd, *J* = 13.6, 11.2, 5.1 Hz, 1H), 2.85 – 2.72 (m, 1H), 2.64 (d, *J* = 7.3 Hz, 1H), 2.36 (s, 6H), 2.27 (s, 3H), 1.92 – 1.71 (m, 3H), 0.88 (t, *J* = 7.1 Hz, 3H).

^{13}C NMR (101 MHz, $CDCl_3$) δ 167.8, 136.2, 135.6, 135.4, 135.1, 129.1, 128.5, 128.3, 127.4, 62.3, 43.2, 35.4, 29.2, 28.2, 20.9, 20.2, 14.3, 13.8.

HRMS: (ESI) calculated for $C_{23}H_{27}INaO_2$ $[M+Na]^+$ m/z: 485.0948, found: 485.0951.

Ethyl (1*R,2*R**,3*S**)-2-(hex-5-en-1-yl)-1-iodo-3-phenylcyclopropane-1-carboxylate (3z)**

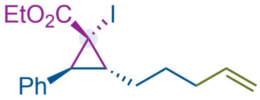

This compound was synthetized following the general procedure **D** using allyltrimethylsilane (68.6 mg, 0.6 mmol). Purification by flash column chromatography (hexane/ethyl acetate: 100/1 → 50/1) provided the title compound as pale-yellow oil (58.9 mg, 74% yield).

^{1}H NMR (400 MHz, $CDCl_3$) δ 7.29 – 7.16 (m, 5H), 5.85 (ddt, *J* = 17.0, 10.2, 6.7 Hz, 1H), 5.05 (dq, *J* = 17.0, 1.7 Hz, 1H), 4.99 (ddt, *J* = 10.2, 2.2, 1.3 Hz, 1H), 3.94 – 3.74 (m, 2H), 2.59 (d, *J* = 7.5 Hz, 1H), 2.19 (dtt, *J* = 8.0, 4.4, 1.4 Hz, 2H), 1.80 – 1.60 (m, 4H), 0.85 (t, *J* = 7.1 Hz, 3H).

^{13}C NMR (126 MHz, $CDCl_3$) δ 167.8, 138.6, 135.7, 128.5, 128.3, 127.3, 115.0, 62.2, 43.2, 35.1, 33.5, 28.6, 27.7, 14.5, 13.8.

HRMS: (ESI) calculated for $C_{17}H_{21}INaO_2$ $[M+Na]^+$ m/z: 407.0478, found: 407.0487.

Ethyl (1*R,2*R**,3*S**)-2-(2-bromoethyl)-3-(4-bromophenyl)-1-iodocyclopropane-1-carboxylate (3aa)**

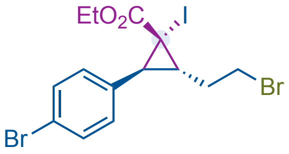

This compound was synthetized following the general procedure **E** using (*E*)-1-bromo-4-(4-iodobut-1-en-1-yl)benzene (80.9 mg, 0.24 mmol) and tetrabutylammonium bromide (96.7 mg, 0.3 mmol). Purification by flash column chromatography (hexane/ethyl acetate: 100/0 → 98/2) provided the title compound as colourless oil (72.3 mg, 72% yield).

^{1}H NMR (500 MHz, $CDCl_3$) δ 7.44 – 7.36 (m, 2H), 7.18 – 7.07 (m, 2H), 3.92 – 3.85 (m, 2H), 3.59 (t, *J* = 6.6 Hz, 2H), 2.61 (d, *J* = 8.1 Hz, 1H), 2.33 (dq, *J* = 14.8, 6.3 Hz, 1H), 2.16 (dq, *J* = 14.3, 7.0 Hz, 1H), 1.94 (td, *J* = 7.7, 6.4 Hz, 1H), 0.93 (t, *J* = 7.1 Hz, 3H).

^{13}C NMR (101 MHz, $CDCl_3$) δ 167.1, 134.1, 131.5, 130.4, 121.6, 62.7, 42.3, 38.6, 31.0, 27.9, 13.9, 12.3.

HRMS: (ESI) calculated for $C_{14}H_{16}Br_2IO_2$ $[M+H]^+$ m/z: 500.8556, found: 500.8539.

Ethyl (1*R,2*R**,3*S**)-2-(2-bromoethyl)-1-iodo-3-(4-(trifluoromethyl)phenyl)cyclopropane-1-carboxylate (3ab)**

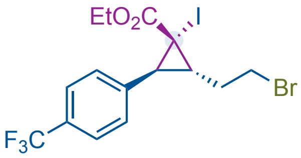

This compound was synthetized following the general procedure **E** using (*E*)-1-(4-iodobut-1-en-1-yl)-4-(trifluoromethyl)benzene (78.3 mg, 0.24 mmol) and tetrabutylammonium bromide (96.7 mg, 0.3 mmol). Purification by flash column chromatography (hexane/ethyl acetate: 100/0 → 98/2) provided the title compound as colourless oil (77.6 mg, 79% yield).

^{1}H NMR (500 MHz, $CDCl_3$) δ 7.54 (d, *J* = 8.2 Hz, 2H), 7.37 (d, *J* = 8.3 Hz, 2H), 3.87 (qd, *J* = 7.1, 4.1 Hz, 2H), 3.61 (t, *J* = 6.5 Hz, 2H), 2.70 (d, *J* = 8.1 Hz, 1H), 2.37 (dq, *J* = 14.8, 6.2 Hz, 1H), 2.18 (dq, *J* = 14.3, 7.0 Hz, 1H), 2.01 (td, *J* = 7.8, 6.3 Hz, 1H), 0.87 (t, *J* = 7.1 Hz, 3H).

^{13}C NMR (126 MHz, $CDCl_3$) δ 167.0, 139.1, 129.8 (q, *J* = 32.6 Hz), 129.1, 125.3 (q, *J* = 3.9 Hz), 124.2 (q, *J* = 272.6 Hz), 62.7, 42.5, 38.5, 31.0, 28.0, 13.7, 12.1.

^{19}F NMR (376 MHz, $CDCl_3$) δ -62.69.

HRMS: (ESI) calculated for $C_{15}H_{15}BrF_3INaO_2$ $[M+Na]^+$ m/z: 512.9144, found: 512.9153.

Ethyl (1*R,2*S**,3*R**)-2-([1,1'-biphenyl]-4-yl)-3-(2-bromoethyl)-1-iodocyclopropane-1-carboxylate (3ac)**

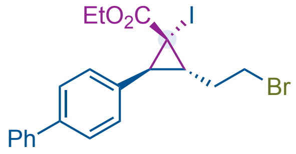

This compound was synthetized following the general procedure **E** using (*E*)-4-(4-iodobut-1-en-1-yl)-1,1'-biphenyl (80.2 mg, 0.24 mmol) and tetrabutylammonium bromide (96.7 mg, 0.3 mmol). Purification by flash column chromatography (hexane/ethyl acetate: 100/0 → 98/2) provided the title compound as colourless oil (30.0 mg, 30% yield).

^{1}H NMR (500 MHz, $CDCl_3$) δ 7.58 – 7.54 (m, 2H), 7.53 – 7.49 (m, 2H), 7.45 – 7.40 (m, 2H), 7.37 – 7.28 (m, 3H), 3.92 – 3.84 (m, 2H), 3.62 (t, *J* = 6.7 Hz, 2H), 2.72 (d, *J* = 8.1 Hz, 1H), 2.36 (dq, *J* = 14.7, 6.4 Hz, 1H), 2.21 (dq, *J* = 14.3, 7.0 Hz, 1H), 2.02 (ddd, *J* = 8.2, 7.3, 6.4 Hz, 1H), 0.88 (t, *J* = 7.1 Hz, 3H).

^{13}C NMR (126 MHz, $CDCl_3$) δ 167.4, 140.8, 140.5, 134.1, 129.1, 128.9, 127.5, 127.1, 127.1, 62.6, 42.6, 38.8, 31.0, 27.7, 13.8, 12.8.

HRMS: (ESI) calculated for $C_{20}H_{20}BrINaO_2$ $[M+Na]^+$ m/z: 520.9584, found: 520.9601.

Ethyl (1*R,2*S**,3*R**)-2-(4-fluorophenyl)-1-iodo-3-(2-iodoethyl)cyclopropane-1-carboxylate (3ad)**

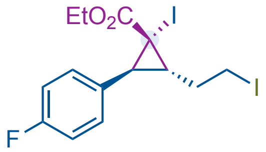

This compound was synthetized following the general procedure **E** using (*E*)-1-fluoro-4-(4-iodobut-1-en-1-yl)benzene (66.3 mg, 0.24 mmol) and tetrabutylammonium iodide (110.8 mg, 0.3 mmol). Purification by flash column chromatography (hexane/ethyl acetate: 100/0 → 98/2) provided the title compound as colourless oil (83.0 mg, 85% yield).

^{1}H NMR (400 MHz, $CDCl_3$) δ 7.25 – 7.18 (m, 2H), 7.03 – 6.91 (m, 2H), 3.95 – 3.79 (m, 2H), 3.41 – 3.29 (m, 2H), 2.64 (d, *J* = 8.1 Hz, 1H), 2.30 (dq, *J* = 15.0, 6.6 Hz, 1H), 2.18 (dq, *J* = 14.5, 7.2 Hz, 1H), 1.90 (dt, *J* = 8.1, 6.8 Hz, 1H), 0.91 (t, *J* = 7.1 Hz, 3H).

^{13}C NMR (101 MHz, $CDCl_3$) δ 167.2, 162.25 (d, *J* = 246.3 Hz), 130.73 (d, *J* = 3.3 Hz), 130.33 (d, *J* = 8.4 Hz), 115.30 (d, *J* = 21.6 Hz), 62.6, 42.1, 39.4, 29.8, 13.9, 12.3 (d, *J* = 1.1 Hz), 2.7.

^{19}F NMR (471 MHz, $CDCl_3$) δ -114.61.

HRMS: (ESI) calculated for $C_{14}H_{15}FI_2NaO_2$ $[M+Na]^+$ m/z: 510.9038, found: 510.9018.

Ethyl (1*R,2*S**,3*R**)-2-(4-chlorophenyl)-1-iodo-3-(2-iodoethyl)cyclopropane-1-carboxylate (3ae)**

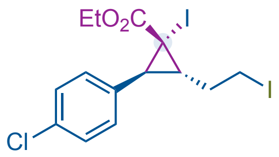

This compound was synthetized following the general procedure **E** using (*E*)-1-chloro-4-(4-iodobut-1-en-1-yl)benzene (70.2 mg, 0.24 mmol) and tetrabutylammonium iodide (110.8 mg, 0.3 mmol). Purification by flash column chromatography (hexane/ethyl acetate: 100/0 → 98/2) provided the title compound as colourless oil (82.7 mg, 82% yield).

^{1}H NMR (400 MHz, $CDCl_3$) δ 7.27 – 7.22 (m, 2H), 7.21 – 7.15 (m, 2H), 3.93 – 3.84 (m, 2H), 3.41 – 3.29 (m, 2H), 2.63 (d, *J* = 8.1 Hz, 1H), 2.30 (dq, *J* = 14.6, 6.6 Hz, 1H), 2.18 (dq, *J* = 14.5, 7.2 Hz, 1H), 1.90 (dt, *J* = 8.2, 6.8 Hz, 1H), 0.93 (t, *J* = 7.1 Hz, 3H).

^{13}C NMR (101 MHz, $CDCl_3$) δ 167.1, 133.5, 133.5, 130.1, 128.6, 62.7, 42.2, 39.4, 29.8, 13.9, 12.2, 2.6.

HRMS: (ESI) calculated for $C_{14}H_{15}ClI_2NaO_2$ $[M+Na]^+$ m/z: 526.8742, found: 526.8730.

Ethyl (1*R,2*S**,3*R**)-2-(4-(*tert*-butyl)phenyl)-1-iodo-3-(2-iodoethyl)cyclopropane-1-carboxylate (3af)**

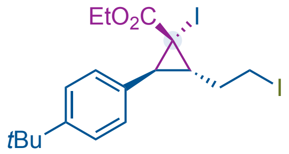

This compound was synthetized following the general procedure **E** using (*E*)-1-(*tert*-butyl)-4-(4-iodobut-1-en-1-yl)benzene (75.4 mg, 0.24 mmol) and tetrabutylammonium iodide (110.8 mg, 0.3 mmol). Purification by flash column chromatography (hexane/ethyl acetate: 100/0 → 98/2) provided the title compound as colourless oil (90.5 mg, 86% yield).

^{1}H NMR (400 MHz, $CDCl_3$) δ 7.31 – 7.26 (m, 2H), 7.19 – 7.13 (m, 2H), 3.92 – 3.76 (m, 2H), 3.41 – 3.29 (m, 2H), 2.64 (d, *J* = 8.1 Hz, 1H), 2.29 (dq, *J* = 13.7, 6.8 Hz, 1H), 2.19 (dq, *J* = 14.5, 7.2 Hz, 1H), 1.92 (dt, *J* = 8.1, 6.9 Hz, 1H), 1.27 (s, 9H), 0.78 (t, *J* = 7.1 Hz, 3H).

^{13}C NMR (101 MHz, $CDCl_3$) δ 167.4, 150.6, 131.9, 128.3, 125.3, 62.4, 42.3, 39.7, 34.6, 31.4, 29.4, 13.6, 12.8, 2.7.

HRMS: (ESI) calculated for $C_{18}H_{25}I_2O_2$ $[M+H]^+$ m/z: 526.9939, found: 526.9922.

Ethyl (1*R,2*S**,3*R**)-2-(3-fluoro-4-methylphenyl)-1-iodo-3-(2-iodoethyl)cyclopropane-1-carboxylate (3ag)**

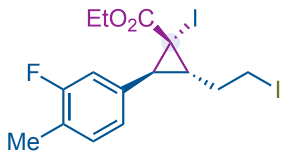

This compound was synthetized following the general procedure **E** using (*E*)-2-fluoro-4-(4-iodobut-1-en-1-yl)-1-methylbenzene (69.6 mg, 0.24 mmol) and tetrabutylammonium iodide (110.8 mg, 0.3 mmol). Purification by flash column chromatography (hexane/ethyl acetate: 100/0 → 98/2) provided the title compound as colourless oil (89.4 mg, 89% yield).

^{1}H NMR (500 MHz, $CDCl_3$) δ 7.07 (t, *J* = 7.9 Hz, 1H), 6.93 – 6.86 (m, 2H), 3.96 – 3.85 (m, 2H), 3.39 – 3.29 (m, 2H), 2.62 (d, *J* = 8.1 Hz, 1H), 2.28 (dq, *J* = 14.8, 6.7 Hz, 1H), 2.22 (d, *J* = 2.1 Hz, 3H), 2.18 (dq, *J* = 14.7, 7.2 Hz, 1H), 1.87 (dt, *J* = 8.2, 6.9 Hz, 1H), 0.95 (t, *J* = 7.1 Hz, 3H).

^{13}C NMR (126 MHz, $CDCl_3$) δ 167.2, 161.1 (d, *J* = 245.0 Hz), 134.6 (d, *J* = 7.8 Hz), 131.3 (d, *J* = 5.5 Hz), 124.1, 124.1 (d, *J* = 21.4 Hz), 115.3 (d, *J* = 23.0 Hz), 62.6, 42.2, 39.5, 29.8, 14.4 (d, *J* = 3.7 Hz), 13.8, 12.3, 2.6.

^{19}F NMR (471 MHz, $CDCl_3$) δ -117.53.

HRMS: (ESI) calculated for $C_{15}H_{18}FI_2O_2$ $[M+H]^+$ m/z: 502.9375, found: 502.9377.

Ethyl (1*R,2*R**,3*S**)-1-iodo-2-(2-iodoethyl)-3-(*m*-tolyl)cyclopropane-1-carboxylate (3ah)**

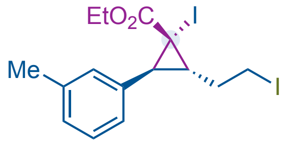

This compound was synthetized following the general procedure **E** using (*E*)-1-(4-iodobut-1-en-1-yl)-3-methylbenzene (65.3 mg, 0.24 mmol) and tetrabutylammonium iodide (110.8 mg, 0.3 mmol). Purification by flash column chromatography (hexane/ethyl acetate: 100/0 → 98/2) provided the title compound as colourless oil (78.4 mg, 81% yield).

^{1}H NMR (400 MHz, $CDCl_3$) δ 7.20 – 7.11 (m, 1H), 7.08 – 6.97 (m, 3H), 3.92 – 3.78 (m, 2H), 3.43 – 3.28 (m, 2H), 2.64 (d, *J* = 8.2 Hz, 1H), 2.31 (s, 3H), 2.33 – 2.25 (m, 1H), 2.20 (dq, *J* = 14.5, 7.2 Hz, 1H), 1.91 (dt, *J* = 8.1, 6.8 Hz, 1H), 0.87 (t, *J* = 7.1 Hz, 3H).

^{13}C NMR (101 MHz, $CDCl_3$) δ 167.4, 138.0, 134.9, 129.4, 128.3, 128.3, 125.6, 62.4, 42.7, 39.7, 29.5, 21.5, 13.8, 12.7, 2.6.

HRMS: (ESI) calculated for $C_{15}H_{18}I_2NaO_2$ $[M+Na]^+$ m/z: 506.9288, found: 506.9310.

Ethyl (1*R,2*R**,3*S**)-1-iodo-2-(2-iodoethyl)-3-(*o*-tolyl)cyclopropane-1-carboxylate (3ai)**

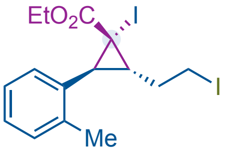

This compound was synthetized following the general procedure **E** using (*E*)-1-(4-iodobut-1-en-1-yl)-2-methylbenzene (65.3 mg, 0.24 mmol) and tetrabutylammonium iodide (110.8 mg, 0.3 mmol). Purification by flash column chromatography (hexane/ethyl acetate: 100/0 → 98/2) provided the title compound as colourless oil (79.4 mg, 82% yield).

^{1}H NMR (400 MHz, $CDCl_3$) δ 7.19 – 7.08 (m, 4H), 3.88 – 3.71 (m, 2H), 3.44 – 3.30 (m, 2H), 2.51 (d, *J* = 8.3 Hz, 1H), 2.39 (s, 3H), 2.33 (dq, *J* = 14.5, 6.8 Hz, 1H), 2.23 (dq, *J* = 14.5, 7.2 Hz, 1H), 2.06 (dt, *J* = 8.2, 6.9 Hz, 1H), 0.79 (t, *J* = 7.1 Hz, 3H).

^{13}C NMR (101 MHz, $CDCl_3$) δ 167.2, 139.0, 133.8, 129.8, 128.2, 127.6, 125.7, 62.4, 42.6, 39.7, 29.4, 20.4, 13.7, 13.3, 2.8.

HRMS: (ESI) calculated for $C_{15}H_{18}I_2NaO_2$ $[M+Na]^+$ m/z: 506.9288, found: 506.9267.

Ethyl (1*R,2*R**,3*S**)-1-iodo-2-(2-iodoethyl)-3-(naphthalen-2-yl)cyclopropane-1-carboxylate (3aj)**

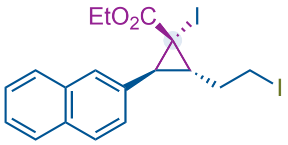

This compound was synthetized following the general procedure **E** using (*E*)-2-(4-iodobut-1-en-1-yl)naphthalene (74.0 mg, 0.24 mmol) and tetrabutylammonium iodide (110.8 mg, 0.3 mmol). Purification by flash column chromatography (hexane/ethyl acetate: 100/1 → 20/1) provided the title compound as pale-yellow oil (71.8 mg, 69% yield).

^{1}H NMR (400 MHz, $CDCl_3$) δ 7.82 – 7.77 (m, 2H), 7.75 (d, *J* = 8.5 Hz, 1H), 7.68 (d, *J* = 1.9 Hz, 1H), 7.50 – 7.42 (m, 2H), 7.38 (dd, *J* = 8.5, 1.9 Hz, 1H), 3.86 – 3.70 (m, 2H), 3.48 – 3.31 (m, 2H), 2.83 (d, *J* = 8.1 Hz, 1H), 2.42 – 2.20 (m, 2H), 2.07 (dt, *J* = 8.1, 6.8 Hz, 1H), 0.76 (t, *J* = 7.1 Hz, 3H).

^{13}C NMR (101 MHz, $CDCl_3$) δ 167.3, 133.3, 132.8, 132.5, 128.0, 127.9, 127.8, 127.3, 126.8, 126.4, 126.1, 62.5, 42.9, 39.6, 29.8, 13.7, 12.7, 2.7.

HRMS: (ESI) calculated for $C_{18}H_{18}I_2NaO_2$ $[M+Na]^+$ m/z: 542.9288, found: 542.9301.

Ethyl (1*R,2*R**,3*S**)-1-iodo-2-(2-iodoethyl)-3-(naphthalen-1-yl)cyclopropane-1-carboxylate (3ak)**

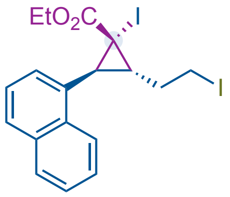

This compound was synthetized following the general procedure **E** using (*E*)-1-(4-iodobut-1-en-1-yl)naphthalene (74.0 mg, 0.24 mmol) and tetrabutylammonium iodide (110.8 mg, 0.3 mmol). Purification by flash column chromatography (hexane/ethyl acetate: 100/1 → 20/1) provided the title compound as pale-yellow oil (71.8 mg, 69% yield).

^{1}H NMR (400 MHz, $CDCl_3$) δ 8.12 (dq, *J* = 8.4, 1.0 Hz, 1H), 7.83 (dt, *J* = 8.1, 0.9 Hz, 1H), 7.75 (dd, *J* = 6.8, 2.7 Hz, 1H), 7.60 (ddd, *J* = 8.4, 6.9, 1.3 Hz, 1H), 7.50 (ddd, *J* = 8.1, 6.9, 1.3 Hz, 1H), 7.45 – 7.37 (m, 2H), 3.56 (qd, *J* = 7.1, 5.0 Hz, 2H), 3.50 – 3.38 (m, 2H), 3.01 (d, *J* = 8.1 Hz, 1H), 2.51 – 2.28 (m, 2H), 2.19 (dt, *J* = 8.1, 6.9 Hz, 1H), 0.38 (t, *J* = 7.1 Hz, 3H).

^{13}C NMR (101 MHz, $CDCl_3$) δ 167.0, 133.6, 133.2, 132.1, 128.6, 128.2, 126.6, 126.3, 126.1, 125.3, 125.1, 62.3, 42.1, 39.8, 29.9, 14.2, 13.2, 2.8.

HRMS: (ESI) calculated for $C_{18}H_{18}I_2NaO_2$ $[M+Na]^+$ m/z: 542.9288, found: 542.9294.

Ethyl (1*R,2*S**,3*R**)-2-(2,3-dihydrobenzofuran-4-yl)-1-iodo-3-(2-iodoethyl)cyclopropane-1-carboxylate (3al)**

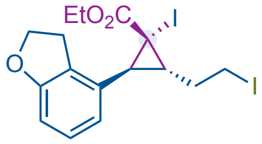

This compound was synthetized following the general procedure **E** using (*E*)-4-(4-iodobut-1-en-1-yl)-2,3-dihydrobenzofuran (72.0 mg, 0.24 mmol) and tetrabutylammonium iodide (110.8 mg, 0.3 mmol). Purification by flash column chromatography (hexane/ethyl acetate: 100/0 – 97/3) provided the title compound as colourless oil (54.3 mg, 53% yield).

^{1}H NMR (500 MHz, $CDCl_3$) δ 7.03 (t, *J* = 7.8 Hz, 1H), 6.67 (d, *J* = 8.0 Hz, 1H), 6.65 (dt, *J* = 7.7, 0.9 Hz, 1H), 4.63 – 4.54 (m, 2H), 3.88 (dq, *J* = 10.8, 7.1 Hz, 1H), 3.82 (dq, *J* = 10.8, 7.1 Hz, 1H), 3.40 – 3.19 (m, 4H), 2.51 (d, *J* = 8.2 Hz, 1H), 2.29 (dq, *J* = 14.7, 6.7 Hz, 1H), 2.21 (dq, *J* = 14.4, 7.1 Hz, 1H), 1.99 (dt, *J* = 8.2, 6.9 Hz, 1H), 0.86 (t, *J* = 7.1 Hz, 3H).

^{13}C NMR (126 MHz, $CDCl_3$) δ 167.3, 159.9, 132.0, 128.1, 127.8, 119.7, 108.7, 71.3, 62.6, 41.6, 39.5, 29.4, 29.3, 13.8, 12.2, 2.6.

HRMS: (ESI) calculated for $C_{16}H_{18}I_2NaO_3$ $[M+Na]^+$ m/z: 534.9238, found: 534.9231.

Ethyl (1*R,2*S**,3*R**)-1-iodo-3-(2-iodoethyl)-2-methyl-2-phenylcyclopropane-1-carboxylate (3am)**

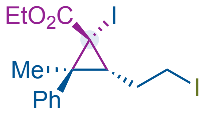

This compound was synthetized following the general procedure **E** using (*E*)-(5-iodopent-2-en-2-yl)benzene (65.3 mg, 0.24 mmol) and tetrabutylammonium iodide (110.8 mg, 0.3 mmol). Purification by flash column chromatography (hexane/ethyl acetate: 100/1 → 20/1) provided the title compound as pale colourless oil (49.4 mg, 51% yield).

^{1}H NMR (400 MHz $CDCl_3$) δ 7.28 – 7.24 (m, 4H), 7.22 – 7.15 (m, 1H), 3.83 – 3.64 (m, 2H), 3.32 (qt, *J* = 9.6, 7.0 Hz, 2H), 2.17 (dd, *J* = 7.7, 5.9 Hz, 1H), 2.15 – 2.06 (m, 2H), 1.54 (s, 3H), 0.85 (t, *J* = 7.1 Hz, 3H).

^{13}C NMR (101 MHz, $CDCl_3$) δ 168.5, 141.6, 128.5, 128.3, 127.3, 62.4, 37.2, 35.2, 32.0, 26.1, 25.6, 13.7, 2.9.

HRMS: (ESI) calculated for $C_{15}H_{19}I_2O_2$ $[M+H]^+$ m/z: 484.9469, found 484.9465.

^{1}H-^{1}H NOESY, ^{1}H-^{13}C HSQC and ^{1}H-^{13}C HMBC were recorded.

Ethyl (1*S,2*R**)-1-iodo-2-(2-iodoethyl)-2-phenylcyclopropane-1-carboxylate (3an)**

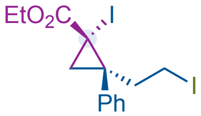

This compound was synthetized following the general procedure **E** using (4-iodobut-1-en-2-yl)benzene (61.9 mg, 0.24 mmol) and tetrabutylammonium iodide (110.8 mg, 0.3 mmol). Purification by flash column chromatography (hexane/ethyl acetate: 100/1 → 20/1) provided the title compound as colourless oil (62.1 mg, 66% yield).

^{1}H NMR (400 MHz, $CDCl_3$) δ 7.32 – 7.19 (m, 5H), 3.82 – 3.62 (m, 2H), 3.10 (td, *J* = 9.4, 4.2 Hz, 1H), 2.85 (td, *J* = 9.4, 7.8 Hz, 1H), 2.71 (dddd, *J* = 13.2, 9.0, 4.2, 1.6 Hz, 1H), 2.63 (dd, *J* = 6.7, 1.6 Hz, 1H), 2.28 (ddd, *J* = 14.1, 9.4, 7.8 Hz, 1H), 1.41 (d, *J* = 6.7 Hz, 1H), 0.84 (t, *J* = 7.1 Hz, 3H).

^{13}C NMR (101 MHz, $CDCl_3$) δ 168.1, 136.1, 129.3, 128.6, 127.8, 62.4, 48.1, 38.7, 28.2, 13.7, 11.3, 1.5.

HRMS: (ESI) calculated for $C_{14}H_{16}I_2NaO_2$ $[M+Na]^+$ m/z: 492.9132, found 492.9127.

^{1}H-^{1}H NOESY, ^{1}H-^{13}C HSQC and ^{1}H-^{13}C HMBC were recorded.

Ethyl (1*S,2*S**,3*S**)-1-iodo-2-(2-iodoethyl)-3-phenylcyclopropane-1-carboxylate (3ao)**

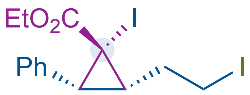

This compound was synthetized following the general procedure **E** using (*Z*)-(4-iodobut-1-en-1-yl)benzene (61.9 mg, 0.24 mmol) and tetrabutylammonium iodide (110.8 mg, 0.3 mmol). Purification by flash column chromatography (hexane/ethyl acetate: 99/1 → 20/1) provided the title compound as pale colourless oil (79.9 mg, 85% yield, *dr* = 8:1).

^{1}H NMR (300 MHz, $CDCl_3$) δ 7.39 – 7.28 (m, 3H), 7.20 (ddt, *J* = 6.3, 2.3, 1.1 Hz, 2H), 4.26 (q, *J* = 7.1 Hz, 2H), 3.42 – 3.13 (m, 2H), 2.74 (d, *J* = 10.2 Hz, 1H), 2.50 – 2.38 (m, 1H), 1.91 – 1.73 (m, 2H), 1.34 (t, *J* = 7.1 Hz, 3H).

^{13}C NMR (101 MHz, $CDCl_3$) δ 170.3, 135.7, 130.7, 128.5, 127.5, 63.3, 36.1, 34.6, 32.6, 19.0, 14.2, 2.5.

HRMS: (ESI) calculated for $C_{14}H_{16}I_2NaO_2$ $[M+Na]^+$ m/z: 492.9132, found 492.9125.

^{1}H-^{1}H NOESY, ^{1}H-^{13}C HSQC and ^{1}H-^{13}C HMBC were recorded.

Ethyl (1*R,2*R**,3*R**)-1-iodo-2-(2-iodoethyl)-3-vinylcyclopropane-1-carboxylate (3ap)**

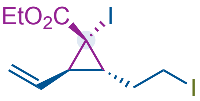

This compound was synthetized following the general procedure **E** using ((*E*)-6-iodohexa-1,3-diene (49.9 mg, 0.24 mmol) and tetrabutylammonium iodide (110.8 mg, 0.3 mmol). Purification by flash column chromatography (hexane/ethyl acetate: 100/0 → 99/1) provided the title compound as colourless oil (26.9 mg, 32% yield).

^{1}H NMR (500 MHz, $CDCl_3$) δ 5.59 (ddd, *J* = 17.1, 10.3, 8.5 Hz, 1H), 5.28 (ddd, *J* = 17.1, 1.5, 0.7 Hz, 1H), 5.17 (ddd, *J* = 10.3, 1.5, 0.6 Hz, 1H), 4.19 (q, *J* = 7.1 Hz, 2H), 3.30 (ddd, *J* = 9.8, 7.4, 6.2 Hz, 1H), 3.22 (dt, *J* = 9.8, 7.5 Hz, 1H), 2.17 – 2.11 (m, 2H), 2.06 (t, *J* = 8.2 Hz, 1H), 1.46 (q, *J* = 7.2 Hz 1H), 1.28 (t, *J* = 7.1 Hz, 3H).

^{13}C NMR (126 MHz, $CDCl_3$) δ 168.1, 133.1, 118.9, 62.9, 42.5, 39.4, 32.3, 14.3, 11.6, 2.3.

HRMS: (ESI) calculated for $C_{10}H_{14}I_2NaO_2$ $[M+Na]^+$ m/z: 442.8975, found: 442.8965.

Ethyl (2*Z*,4*E*)-7-iodo-2-(iodomethyl)hepta-2,4-dienoate (3ap*)

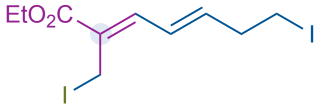

This compound was synthetized following the general procedure **E** using ((E)-6-iodohexa-1,3-diene (49.9 mg, 0.24 mmol) and tetrabutylammonium iodide (110.8 mg, 0.3 mmol). Purification by flash column chromatography (hexane/ethyl acetate: 100/0 → 98/2) provided the title compound as colourless oil (19.3 mg, 23% yield).

^{1}H NMR (400 MHz, CDCl3) δ 7.23 (dd, *J* = 11.5, 0.8 Hz, 1H), 6.46 (ddt, *J* = 15.0, 11.5, 1.4 Hz, 1H), 6.20 (dtd, *J* = 14.9, 7.0, 0.8 Hz, 1H), 4.54 – 4.02 (m, 4H), 3.23 (t, *J* = 7.0 Hz, 2H), 2.78 (qd, *J* = 7.0, 1.4 Hz, 2H), 1.34 (t, *J* = 7.1 Hz, 3H).

^{13}C NMR (126 MHz, CDCl3) δ 165.8, 142.9, 139.9, 128.7, 127.6, 61.3, 37.3, 14.4, 2.9, -3.2.

HRMS: (ESI) calculated for C10H14I2NaO2 [M+Na]+ m/z: 442.8975, found: 442.8961

Ethyl (1*S,2*R**,3*R**)-2-benzyl-1-iodo-3-(2-iodoethyl)cyclopropane-1-carboxylate (3aq)**

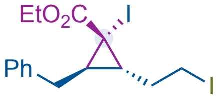

This compound was synthetized following the general procedure **E** using (*E*)-(5-iodopent-2-en-1-yl)benzene (65.3 mg, 0.24 mmol) and tetrabutylammonium iodide (110.8 mg, 0.3 mmol). Purification by flash column chromatography (hexane/ethyl acetate: 100/0 → 98/2) provided the title compound as colourless oil (87.1 mg, 90% yield).

^{1}H NMR (400 MHz, $CDCl_3$) δ 7.33 – 7.28 (m, 2H), 7.24 – 7.15 (m, 3H), 4.16 (q, *J* = 7.1 Hz, 2H), 3.19 – 3.07 (m, 2H), 2.89 (dd, *J* = 15.1, 7.2 Hz, 1H), 2.80 (dd, *J* = 15.1, 7.5 Hz, 1H), 2.13 (dq, *J* = 13.7, 6.8 Hz, 1H), 2.03 (dq, *J* = 14.6, 7.3 Hz, 1H), 1.71 (q, *J* = 7.5 Hz, 1H), 1.34 (dt, *J* = 8.1, 6.9 Hz, 1H), 1.23 (t, *J* = 7.1 Hz, 3H).

^{13}C NMR (101 MHz, $CDCl_3$) δ 168.6, 139.7, 128.7, 128.4, 126.5, 62.9, 40.6, 39.8, 34.4, 32.1, 14.2, 10.7, 2.7.

HRMS: (ESI) calculated for $C_{15}H_{18}I_2NaO_2$ $[M+Na]^+$ m/z: 506.9288, found: 506.9302.

Ethyl (1*S,2*R**,3*R**)-2-hexyl-1-iodo-3-(2-iodoethyl)cyclopropane-1-carboxylate (3ar)**

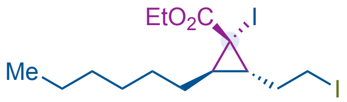

This compound was synthetized following the general procedure **E** using (*E*)-1-iododec-3-ene (63.9 mg, 0.2 mmol) and tetrabutylammonium iodide (110.8 mg, 0.3 mmol). Purification by flash column chromatography (hexane/ethyl acetate: 100/0 → 99/1) provided the title compound as colourless oil (84.2 mg, 88% yield).

^{1}H NMR (400 MHz, $CDCl_3$) δ 4.20 (q, *J* = 7.2 Hz, 2H), 3.32 – 3.16 (m, 2H), 2.16 – 2.01 (m, 2H), 1.51 – 1.22 (m, 11H), 1.29 (t, *J* = 7.1 Hz, 3H), 1.12 (q, *J* = 7.1 Hz, 1H), 0.88 (t, *J* = 6.8 Hz, 3H).

^{13}C NMR (126 MHz, $CDCl_3$) δ 168.9, 62.7, 40.2, 39.8, 31.8, 31.8, 29.1, 28.8, 28.6, 22.7, 14.3, 14.2, 10.8, 2.9.

HRMS: (ESI) calculated for $C_{14}H_{25}I_2O_2$ $[M+H]^+$ m/z: 478.9939, found: 478.9924.

Ethyl (1*S,2*R**,3*R**)-2-(2-(benzyloxy)ethyl)-1-iodo-3-(2-iodoethyl)cyclopropane-1-carboxylate (3as)**

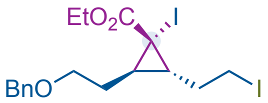

This compound was synthetized following the general procedure **E** using (*E*)-(((6-iodohex-3-en-1-yl)oxy)methyl)benzene (75.9 mg, 0.24 mmol) and tetrabutylammonium iodide (110.8 mg, 0.3 mmol). Purification by flash column chromatography (hexane/ethyl acetate: 100/0 → 97/3) provided the title compound as colourless oil (89.8 mg, 85% yield).

^{1}H NMR (400 MHz, $CDCl_3$) δ 7.43 – 7.26 (m, 5H), 4.53 (d, *J* = 12,0 Hz, 1H), 4.49 (d, *J* = 12,0 Hz, 1H), 4.15 (qd, *J* = 7.1, 3.8 Hz, 2H), 3.51 (td, *J* = 6.0, 1.2 Hz, 2H), 3.28 – 3.12 (m, 2H), 2.13 (dq, *J* = 13.8, 6.8 Hz, 1H), 2.03 (dq, *J* = 14.6, 7.3 Hz, 1H), 1.92 – 1.81 (m, 1H), 1.77 – 1.67 (m, 1H), 1.58 (q, *J* = 7.2 Hz, 1H), 1.25 (t, *J* = 7.1 Hz, 3H), 1.17 (dt, *J* = 8.1, 6.9 Hz, 1H).

^{13}C NMR (101 MHz, $CDCl_3$) δ 168.8, 138.4, 128.6, 127.9, 127.8, 73.2, 69.0, 62.7, 39.7, 37.4, 31.7, 28.8, 14.2, 10.5, 3.0.

HRMS: (ESI) calculated for $C_{17}H_{23}I_2O_3$ $[M+H]^+$ m/z: 528.9731, found: 528.9732.

Ethyl (1*S,2*R**,3*R**)-1-iodo-2-(2-iodoethyl)-3-(2-(pivaloyloxy)ethyl)cyclopropane-1-carboxylate (3at)**

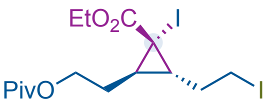

This compound was synthetized following the general procedure **E** using (*E*)-6-iodohex-3-en-1-yl pivalate (74.4 mg, 0.24 mmol) and tetrabutylammonium iodide (110.8 mg, 0.3 mmol). Purification by flash column chromatography (hexane/ethyl acetate: 100/0 → 98/2) provided the title compound as colourless oil (78.3 mg, 75% yield).

^{1}H NMR (400 MHz, $CDCl_3$) δ 4.21 (qd, *J* = 7.1, 1.1 Hz, 2H), 4.15 – 4.02 (m, 2H), 3.33 – 3.14 (m, 2H), 2.10 (qd, *J* = 7.2, 2.7 Hz, 2H), 1.94 – 1.76 (m, 2H), 1.49 (q, *J* = 7.5 Hz, 1H), 1.30 (t, *J* = 7.1 Hz, 3H), 1.21 (s, 9H), 1.17 (q, *J* = 7.0 Hz, 1H).

^{13}C NMR (101 MHz, $CDCl_3$) δ 178.6, 168.6, 63.2, 63.0, 39.5, 38.9, 37.1, 31.8, 27.7, 27.4, 14.2, 9.8, 2.6.

HRMS: (ESI) calculated for $C_{15}H_{24}I_2NaO_4$ $[M+Na]^+$ m/z: 544.9656, found: 544.9650.

Ethyl (1*R,2*S**)-1-iodo-2-(2-iodoethyl)spiro[2.5]octane-1-carboxylate (3au)**

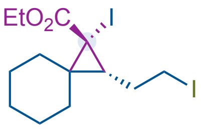

This compound was synthetized following the general procedure **E** using (3-iodopropylidene)cyclohexane (60.0 mg, 0.24 mmol) and tetrabutylammonium iodide (110.8 mg, 0.3 mmol). Purification by flash column chromatography (hexane/ethyl acetate: 100/0 → 99/1) provided the title compound as colourless oil (67.5 mg, 73% yield).

^{1}H NMR (400 MHz, $CDCl_3$) δ 4.20 (qd, *J* = 7.1, 1.5 Hz, 2H), 3.28 (ddd, *J* = 9.6, 8.6, 5.6 Hz, 1H), 3.17 (ddd, *J* = 9.6, 8.5, 7.2 Hz, 1H), 2.04 (dddd, *J* = 14.5, 8.6, 7.2, 5.8 Hz, 1H), 1.87 (dtd, *J* = 14.3, 8.1, 5.7 Hz, 1H), 1.63 – 1.53 (m, 3H), 1.52 – 1.38 (m, 6H), 1.32 – 1.22 (m, 1H), 1.28 (t, *J* = 7.1 Hz, 3H), 1.19 (dd, *J* = 7.8, 5.8 Hz, 1H).

^{13}C NMR (101 MHz, $CDCl_3$) δ 169.9, 62.3, 34.6, 33.9, 33.4, 33.1, 32.7, 25.9, 25.1, 24.8, 23.9, 14.2, 3.0.

HRMS: (ESI) calculated for $C_{13}H_{20}I_2NaO_2$ $[M+Na]^+$ m/z: 484.9445, found: 484.9441.

Ethyl (1*S,7*R**)-7-iodo-1-(2-iodoethyl)bicyclo[4.1.0]heptane-7-carboxylate (3av)**

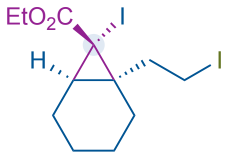

This compound was synthetized following the general procedure **E** using 1-(2-iodoethyl)cyclohex-1-ene (56.7 mg, 0.24 mmol) and tetrabutylammonium iodide (110.8 mg, 0.3 mmol). Purification by flash column chromatography (hexane/ethyl acetate: 99/1 → 20/1) provided the title compound as pale-yellow oil (67.5 mg, 75% yield).

^{1}H NMR (400 MHz, $CDCl_3$) δ 4.20 (qd, *J* = 7.1, 0.7 Hz, 2H), 3.38 (ddd, *J* = 11.4, 9.6, 4.5 Hz, 1H), 3.24 (ddd, *J* = 11.2, 9.6, 6.1 Hz, 1H), 2.34 (ddd, *J* = 14.0, 11.4, 6.1 Hz, 1H), 2.14 (ddd, *J* = 14.0, 11.1, 4.5 Hz, 1H), 2.10 – 1.92 (m, 2H), 1.88 – 1.77 (m, 1H), 1.71 (ddd, *J* = 14.0, 7.4, 5.6 Hz, 1H), 1.36 – 1.16 (m, 6H), 1.00 (tdd, *J* = 12.4, 5.9, 2.8 Hz, 2H).

^{13}C NMR (101 MHz, $CDCl_3$) δ 168.9, 61.7, 50.0, 32.1, 28.0, 23.0, 21.0, 20.6, 19.6, 13.9, 13.8, -0.4.

HRMS: (ESI) calculated for $C_{12}H_{18}I_2NaO_2$ $[M+Na]^+$ m/z: 470.9288, found 470.9282.

Ethyl (1*R,2*R**,3*S**)-1-iodo-2-(3-iodopropyl)-3-phenylcyclopropane-1-carboxylate (3ay)**

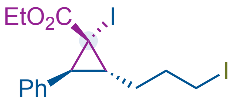

This compound was synthetized following the general procedure **E** using (*E*)-(5-iodopent-1-en-1-yl)benzene (65.3 mg, 0.24 mmol) and tetrabutylammonium iodide (110.8 mg, 0.3 mmol). Purification by flash column chromatography (hexane/ethyl acetate: 100/1 → 50/1) provided the title compound as colourless oil (48.4 mg, 50% yield).

^{1}H NMR (400 MHz, $CDCl_3$) δ 7.27 – 7.21 (m, 3H), 7.20 – 7.16 (m, 2H), 3.91 – 3.76 (m, 2H), 3.30 (t, *J* = 6.9 Hz, 2H), 2.63 (d, *J* = 7.5 Hz, 1H), 2.28 – 1.98 (m, 2H), 1.88 – 1.69 (m, 3H), 0.85 (t, *J* = 7.1 Hz, 3H).

^{13}C NMR (101 MHz, $CDCl_3$) δ 167.6, 135.3, 128.4, 128.4, 127.5, 62.4, 42.9, 36.4, 32.1, 27.5, 13.8, 13.8, 5.9.

HRMS: (ESI) calculated for $C_{15}H_{18}I_2NaO_2$ $[M+Na]^+$ m/z: 506.9288, found: 506.9283.

Ethyl (1*R,2*R**,3*S**)-1-iodo-2-(1-iodopropan-2-yl)-3-phenylcyclopropane-1-carboxylate (3ba)**

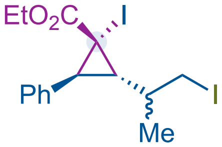

This compound was synthetized following the general procedure **E** using (*E*)-(4-iodo-3-methylbut-1-en-1-yl)benzene (65.3 mg, 0.24 mmol) and tetrabutylammonium iodide (110.8 mg, 0.3 mmol). Purification by flash column chromatography (hexane/ethyl acetate: 100/0 → 98/2) provided the title compound as colourless oil (53.3 mg, 55% yield, *dr* = 1:1).

Spectral data for the mixture of diastereomers is reported.

^{1}H NMR (500 MHz, $CDCl_3$) δ 7.33 – 7.19 (m, 10H), 3.90 – 3.78 (m, 4H), 3.47 (dd, *J* = 9.8, 3.0 Hz, 1H), 3.43 – 3.35 (m, 2H), 3.32 (dd, *J* = 9.8, 7.4 Hz, 1H), 2.75 (d, *J* = 8.7 Hz, 1H), 2.71 (d, *J* = 8.8 Hz, 1H), 1.69 (dd, *J* = 11.5, 8.3 Hz, 1H), 1.67 (dd, *J* = 11.5, 8.3 Hz, 1H), 1.58 – 1.48 (m, 2H), 1.36 (d, *J* = 6.6 Hz, 3H), 1.26 (d, *J* = 6.5 Hz, 3H), 0.88 (t, *J* = 7.1 Hz, 3H), 0.84 (t, *J* = 7.1 Hz, 3H).

^{13}C NMR (126 MHz, $CDCl_3$) δ 167.3, 167.1, 135.2, 135.0, 128.8, 128.5, 128.4, 128.3, 127.6, 127.6, 62.5, 42.9, 42.5, 41.8, 41.6, 34.6, 34.3, 20.3, 19.5, 14.4, 13.8, 13.7, 13.4, 12.9, 12.6.

HRMS: (ESI) calculated for $C_{15}H_{19}I_2O_2$ $[M+H]^+$ m/z: 484.9469, found: 484.9472.

((1*R,2*R**,3*S**)-1-iodo-2-(2-iodoethyl)-3-phenylcyclopropyl)(phenyl)methanone (3bc)**

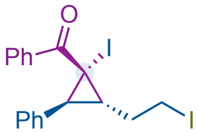

This compound was synthetized following the general procedure **F** using **2d** (99.6 mg, 0.2 mmol), (*E*)-(4-iodobut-1-en-1-yl)benzene (61.6 mg, 0.24 mmol) and tetrabutylammonium iodide (110.8 mg, 0.3 mmol). Purification by flash column chromatography (hexane/ethyl acetate: 100/1 → 20/1) provided the title compound as pale brown amorphous solid (70.3 mg, 70% yield).

^{1}H NMR (400 MHz, $CDCl_3$) δ 7.83 – 7.78 (m, 2H), 7.40 – 7.33 (m, 1H), 7.29 – 7.22 (m, 2H), 7.13 (tt, *J* = 7.9, 1.5 Hz, 2H), 7.10 – 7.00 (m, 3H), 3.47 – 3.30 (m, 2H), 2.76 (d, *J* = 7.8 Hz, 1H), 2.48 – 2.27 (m, 2H), 1.90 (q, *J* = 7.0 Hz, 1H).

^{13}C NMR (101 MHz, $CDCl_3$) δ 192.8, 135.6, 134.6, 133.1, 130.2, 128.7, 128.0, 127.4, 127.1, 41.9, 40.8, 27.9, 20.5, 2.3.

HRMS: (ESI) calculated for $C_{18}H_{16}I_2NaO$ $[M+Na]^+$ m/z: 524.9183, found: 524.9178.

2,2,2-Trichloroethyl (1*R,2*R**,3*S**)-1-iodo-2-(2-iodoethyl)-3-phenylcyclopropane-1-carboxylate (3bd)**

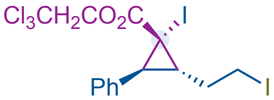

This compound was synthetized following the general procedure **F** using **2e** (113.9 mg, 0.2 mmol), (*E*)-(4-iodobut-1-en-1-yl)benzene (61.6 mg, 0.24 mmol) and tetrabutylammonium iodide (110.8 mg, 0.3 mmol). Purification by flash column chromatography (hexane/ethyl acetate: 100/0 → 97/3) provided the title compound as colourless oil (71.1 mg, 62% yield).

^{1}H NMR (500 MHz, $CDCl_3$) δ 7.29 – 7.22 (m, 5H), 4.42 (d, J = 11.9 Hz, 1H), 4.36 (d, J = 11.9 Hz, 1H), 3.39 (dt, J = 9.9, 6.7 Hz, 1H), 3.35 (dt, J = 9.9, 7.1 Hz, 1H), 2.80 (d, J = 8.3 Hz, 1H), 2.32 (dq, J = 15.0, 6.6 Hz, 1H), 2.25 (dq, J = 15.1, 7.1 Hz, 1H), 2.03 (dt, J = 8.3, 6.8 Hz, 1H).

^{13}C NMR (126 MHz, $CDCl_3$) δ 166.0, 134.3, 128.7, 128.6, 127.9, 94.2, 75.6, 43.7, 39.3, 30.2, 10.0, 2.4.

HRMS: (ESI) calculated for $C_{14}H_{13}Cl_3I_2NaO_2$ $[M+Na]^+$ m/z: 594.7963, found: 594.7958.

((1*S,2*R**,3*R**)-2-iodo-3-(2-iodoethyl)-2-(trifluoromethyl)cyclopropyl)benzene (3be)**

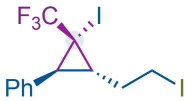

This compound was synthetized following the general procedure **F** using **2f** (92.4 mg, 0.2 mmol), (*E*)-(4-iodobut-1-en-1-yl)benzene (61.6 mg, 0.24 mmol) and tetrabutylammonium iodide (110.8 mg, 0.3 mmol). Purification by flash column chromatography (hexane/ethyl acetate: 99/1 → 20/1) provided the title compound as pale-yellow oil (28.0 mg, 30% yield).

^{1}H NMR (500 MHz, $CDCl_3$) δ 7.36 – 7.29 (m, 5H), 3.46 – 3.31 (m, 2H), 2.76 (d, J = 8.3 Hz, 1H), 2.31 (q, J = 6.8 Hz, 2H), 1.55 (dt, J = 8.3, 6.8 Hz, 1H).

^{13}C NMR (126 MHz, $CDCl_3$) δ 133.7, 128.9, 128.6, 127.9, 123.8 (q, J = 274.2 Hz), 40.0, 39.1, 26.4 (q, J = 1.9 Hz), 10.7 (q, J = 35.6 Hz), 2.1.

^{19}F NMR (471 MHz, $CDCl_3$) δ -61.9.

HRMS: (ESI) calculated for $C_{12}H_{12}F_3I_2$ $[M+H]^+$ m/z: 466.8975, found: 466.8964.

4. Synthesis of iodocyclopropanes 4

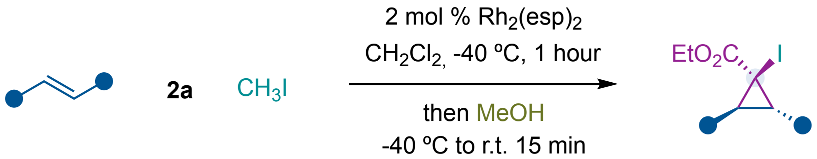

To a 10 mL oven-dried reaction vial equipped with a stirring bar was added $Rh_2(esp)_2$ (3.0 mg, 0.004 mmol, 2 mol%) and the tube was sealed before being evacuated and backfilled with argon three times. The corresponding alkene (0.24 mmol), methyl iodide (15 µL, 0.24 mmol) and dichloromethane (0.5 mL) were added, and the resulting mixture was cooled at -40 ºC. Then a solution of reagent **2a** (118.4 mg, 0.2 mmol) in dichloromethane (2.0 mL) was added dropwise during 30 minutes using a syringe pump and was stirred for 60 minutes at the same temperature. After this, methanol (0.2 mL) was added. Then the resulting reaction mixture was allowed to warm to room temperature over 15 minutes followed by the removal of solvent by rotatory evaporation. The crude residue was purified by flash column chromatography to afford the corresponding iodocyclopropanes **4**.

Ethyl (1*S,2*S**)-1-iodo-2-phenylcyclopropane-1-carboxylate (4a)**

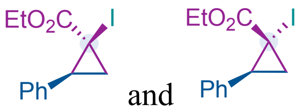

This compound was synthetized following the procedure outlined above (section **4**) using styrene (25 mg, 0.24 mmol). Purification by flash column chromatography (hexane/ethyl acetate: 99/1) provided the title compound as mixture of diastereoisomers, pale-yellow oil (25.3 mg, 40% yield, *dr* = 2:1). Spectral data for this compound matches with the reported literature.[12]

Diastereoisomer 1:

^{1}H NMR (400 MHz, $CDCl_3$) δ 7.14 – 7.38 (m, 5H), 4.28 – 4.19 (m, 2H), 2.58 (dd, *J* = 9.9, 8.3 Hz, 1H), 2.29 (dd, *J* = 10.0, 5.9 Hz, 1H), 1.74 (dd, *J* = 8.3, 5.9 Hz, 1H), 1.33 (t, *J* = 7.1 Hz, 3H).

^{13}C NMR (101 MHz, $CDCl_3$): δ 170.0, 138.0, 129.3, 128.1, 127.6, 63.0, 33.1, 25.2, 14.1, 9.5.

Diastereoisomer 2:

^{1}H NMR (400 MHz, $CDCl_3$) δ 7.14 – 7.38 (m, 5H), 3.90 – 3.73 (m, 2H), 3.01 – 2.92 (m, 1H), 2.36 (dd, *J* = 8.0, 6.7 Hz, 1H), 1.65 (dd, *J* = 9.5, 6.7 Hz, 1H), 0.84 (t, *J* = 7.1 Hz, 3H).

^{13}C NMR (101 MHz, $CDCl_3$): δ 168.8, 133.8, 128.7, 128.2, 127.3, 62.3, 37.2, 22.8, 13.9, 13.6.

Ethyl (2*R,3*S**)-1-iodo-2-methyl-3-phenylcyclopropane-1-carboxylate (4b)**

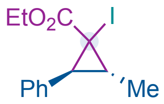

This compound was synthesized following the procedure outlined in section **4** using (*E*)-β-methylstyrene (25.0 mg, 0.24 mmol). Purification by flash column chromatography (hexane/ethyl acetate: 99/1) provided the title compound as mixture of diastereoisomers, pale-yellow oil (13.2 mg, 20% yield, *dr* = 1:1).

^{1}H NMR (500 MHz, $CDCl_3$) δ 7.36 – 7.28 (m, 3H), 7.26 – 7.17 (m, 7H), 4.26 (q, *J* = 7.1 Hz, 2H), 3.91 – 3.76 (m, 2H), 2.56 (d, *J* = 8.1 Hz, 1H), 2.49 (d, *J* = 8.4 Hz, 1H), 2.09 (dq, *J* = 8.4, 6.3 Hz, 1H), 1.82 (dq, *J* = 8.1, 6.2 Hz, 1H), 1.41 (d, *J* = 6.2 Hz, 3H), 1.38 – 1.30 (m, 6H), 0.85 (t, *J* = 7.1 Hz, 3H).

^{13}C NMR (101 MHz, $CDCl_3$) δ 168.9, 167.9, 139.0, 135.7, 129.2, 128.5, 128.3, 128.2, 127.5, 127.3, 62.7, 62.2, 44.2, 36.8, 32.3, 23.5, 20.8, 15.8, 15.0, 14.4, 13.8, 13.5.

HRMS: (ESI) calculated for $C_{13}H_{15}INaO_2$ $[M+Na]^+$ m/z: 353.0009, found: 353.0009.

Ethyl (1*R,6*S**,7*S**)-7-iodobicyclo[4.1.0]heptane-7-carboxylate (4c)**

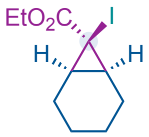

This compound was synthesized following the procedure outlined in section **4** using cyclohexene (19.7 mg, 0.24 mmol). Purification by flash column chromatography (hexane/ethyl acetate: 99/1) provided the title compound as pale-yellow oil (44.1 mg, 75% yield, *dr* > 20:1).

^{1}H NMR (500 MHz, $CDCl_3$) δ 4.13 (q, *J* = 7.1 Hz, 2H), 2.19 – 2.08 (m, 2H), 1.77 – 1.70 (m, 2H), 1.59 – 1.48 (m, 2H), 1.44 – 1.36 (m, 2H), 1.28 – 1.23 (m, 6H).

^{13}C NMR (126 MHz, $CDCl_3$) δ 171.5, 62.8, 25.0, 25.0, 23.0, 20.6, 14.2.

HRMS: (ESI) calculated for $C_{10}H_{15}INaO_2$ $[M+Na]^+$ m/z: 317.0009, found: 317.0016.

^{1}H-^{1}H NOESY, ^{1}H-^{13}C HSQC and ^{1}H-^{13}C HMBC were recorded.

Ethyl (1*R,6*S**,7*S**)-7-iodobicyclo[4.1.0]hept-3-ene-7-carboxylate (4d)**

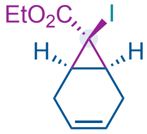

This compound was synthetized following the procedure outlined in section **4** using cyclohexa-1,4-diene (19.2 mg, 0.24 mmol). Purification by flash column chromatography (hexane/ethyl acetate: 99/1) provided the title compound as pale-yellow oil (43.2 mg, 74% yield, *dr* > 20:1).

^{1}H NMR (400 MHz, $CDCl_3$) δ 5.53 (s, 2H), 4.16 (q, J = 7.1 Hz, 2H), 2.74 – 2.51 (m, 2H), 1.95 – 1.77 (m, 4H), 1.28 (t, J = 7.2 Hz, 3H).

^{13}C NMR (101 MHz, $CDCl_3$) δ 171.3, 123.2, 62.9, 29.9, 24.2, 23.1, 14.2.

HRMS: (ESI) calculated for $C_{10}H_{13}INaO_2$ $[M+Na]^+$ m/z: 314.9852, found: 314.9858.

^{1}H-^{1}H NOESY, ^{1}H-^{13}C HSQC and ^{1}H-^{13}C HMBC were recorded.

5. Synthesis of bicyclic alkyl–I$^{(III)}$ 3a-*int-III*

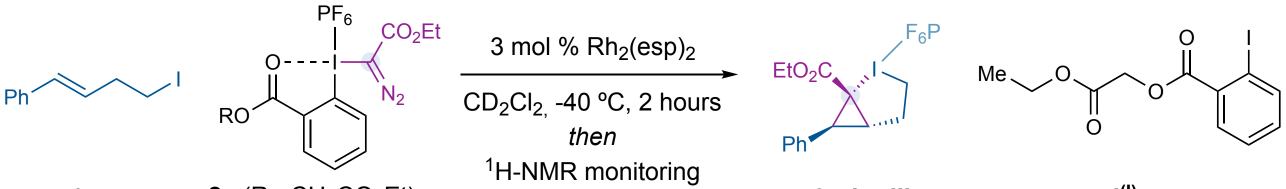

To a 10 mL reaction vial equipped with a stirring bar was added $Rh_2(esp)_2$ (2.3 mg, 0.003 mmol, 3 mol%) and homoallylic iodide **1a** (31.0 mg, 0.12 mmol, 1.2 equiv.). The tube was sealed before being evacuated and backfilled with argon. Deuterated dichloromethane (0.3 mL) was added and the resulting mixture was cooled at -40 ºC. Then, a solution of reagent **2a** (59.2 mg, 0.1 mmol, 1.0 equiv.) in deuterated dichloromethane (1.0 mL) was added dropwise during 30 minutes using a syringe pump and was stirred for 60 min at -40 ºC. Dibromomethane (7.0 μL, 0.1 mmol) was added as an internal standard to the reaction and was rapidly transferred (0.6 mL) to a previously backfilled with argon NMR tube at -40 ºC via canula. The NMR spectra of the reaction crude were measured at -40 ºC in a 500 MHz NMR spectrometer. The yield of **3a-*int-III*** was determined using ^{1}H-NMR spectroscopy using CH_2Br_2 as internal standard (80%). The same NMR sample was then subjected to a temperature ramp and ^{1}H-NMR were recorded every 5 ºC higher temperature.

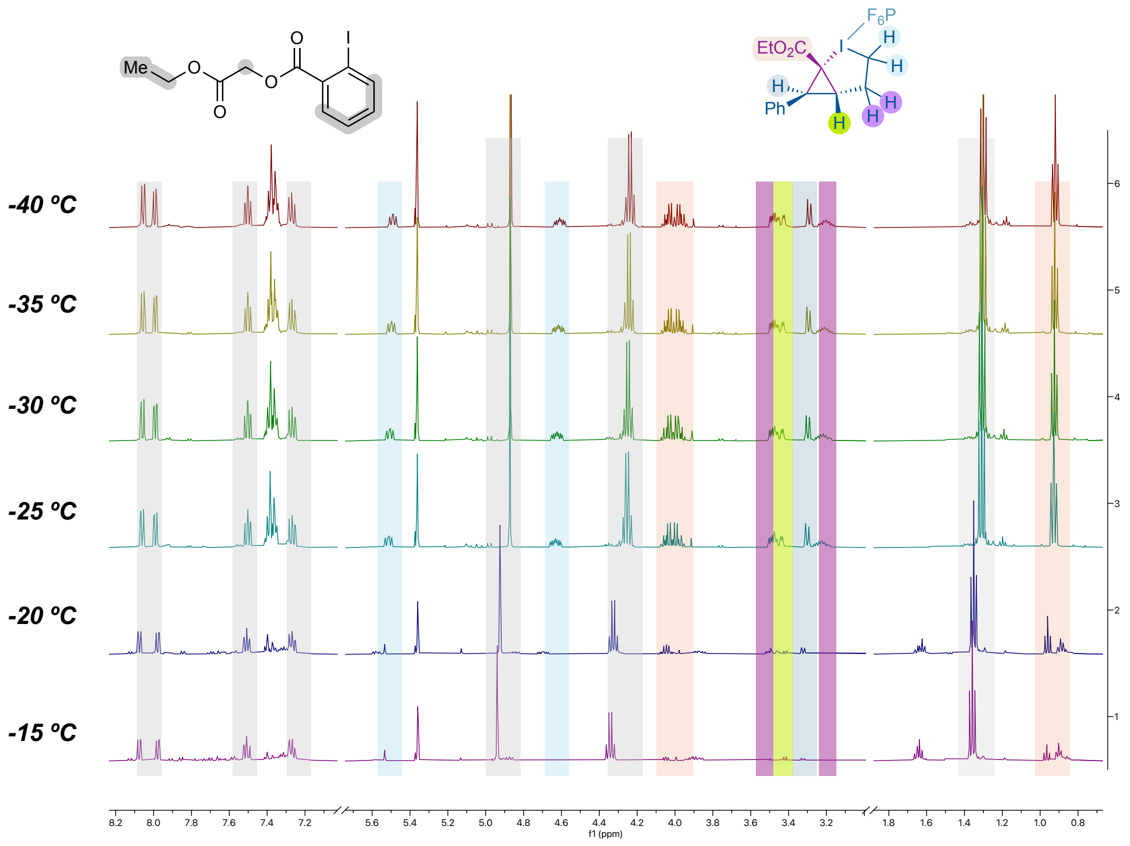

Figure 1. ^{1}H-NMR crude spectrum of the reaction outlined in section 5 yielding **3a-*int-III*** and byproduct **I$^{(I)}$** at different temperatures.

Characterization of **3a-*int-III*** was performed following the same procedure as for the temperature ramp excluding the internal standard. ^{1}H, ^{13}C, ^{31}P, ^{1}H-^{13}C HSQC, ^{1}H-^{13}C HMBC and ^{1}H-^{1}H 2D NOESY were recorded at -40 ºC. For HRMS, an aliquot of the same reaction was diluted 10 times with CH_2Cl_2 and filtered through a syringe filter prior to a direct injection to the mass spectrometer:

(1*R,5*R**,6*S**)-1-(ethoxycarbonyl)-6-phenylbicyclo[3.1.0]hexan-2-ium hexafluorophosphate (3a-*int-III*).**

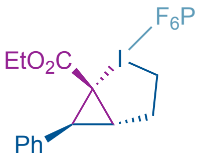

^{1}H NMR (500 MHz, CD_2Cl_2) δ 7.39 – 7.29 (m, 5H), 5.47 (dd, *J* = 9.3, 7.0 Hz, 1H), 4.58 (ddd, *J* = 12.8, 9.8, 4.7 Hz, 1H), 4.04 – 3.90 (m, 2H), 3.47 – 3.43 (m, 1H), 3.43 – 3.38 (m, 1H), 3.26 (d, *J* = 7.2 Hz, 1H), 3.17 (dddd, *J* = 15.0, 12.2, 7.1, 4.7 Hz, 1H), 0.89 (t, *J* = 7.1 Hz, 3H).
^{13}C NMR (126 MHz, CD_2Cl_2) δ 164.8, 130.4, 129.1, 128.7, 126.0, 65.0, 50.3, 47.1, 38.3, 37.5, 36.6, 13.4.
^{31}P NMR (202 MHz, CD_2Cl_2) δ -141.6 (sept, *J* = 706.0 Hz).
HRMS: (MALDI) calculated for $C_{14}H_{16}IO_2$ $[M\text{-}PF_6]^+$ m/z: 343.0190, found: 343.0188.

Analysis Info

Analysis Name	D:\Data\MALDI\2025\250117\LOW\JE680_0_F4_MS.d	Acquisition Date	1/17/2025 1:18:25 PM
Method	small molecules tims off pos DD.m	Operator	TOF-User
Sample Name	JE680	Instrument / Ser#	timsTOF flex 1893754.3 0739
Comment	dctb in CH2Cl2, sample as received, MALDI+		

Acquisition Parameter

Source Type	MALDI	Ion Polarity	Positive
Focus	Not active		
Scan Begin	230 m/z	Set Capillary	3500 V
Scan End	800 m/z	Set End Plate Offset	-500 V

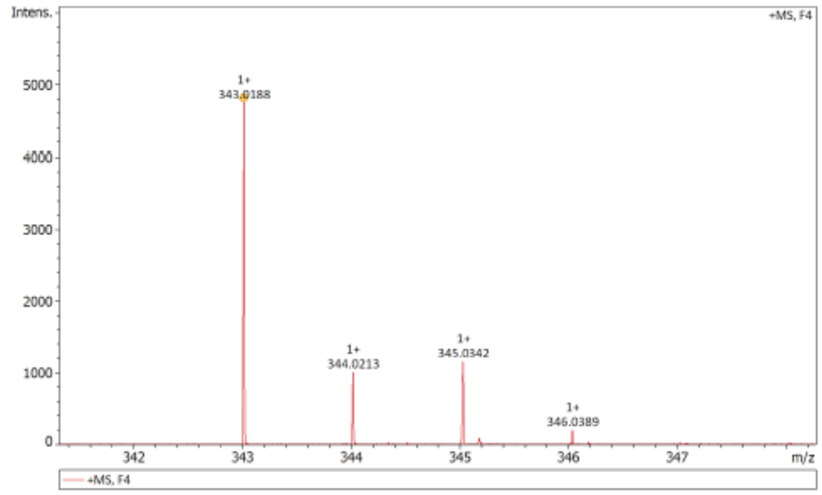

Meas. m/z	Ion Formula	m/z	err [ppm]	err [mDa]	mSigma	e⁻ Conf	z
343.0188	C13H5N5O7	343.0183	-1.4	-0.5	32.9	odd	1+
343.0188	C14H16IO2	343.0190	0.4	0.1	136.3	even	1+

6. Enantiomeric difference mechanistic experiment

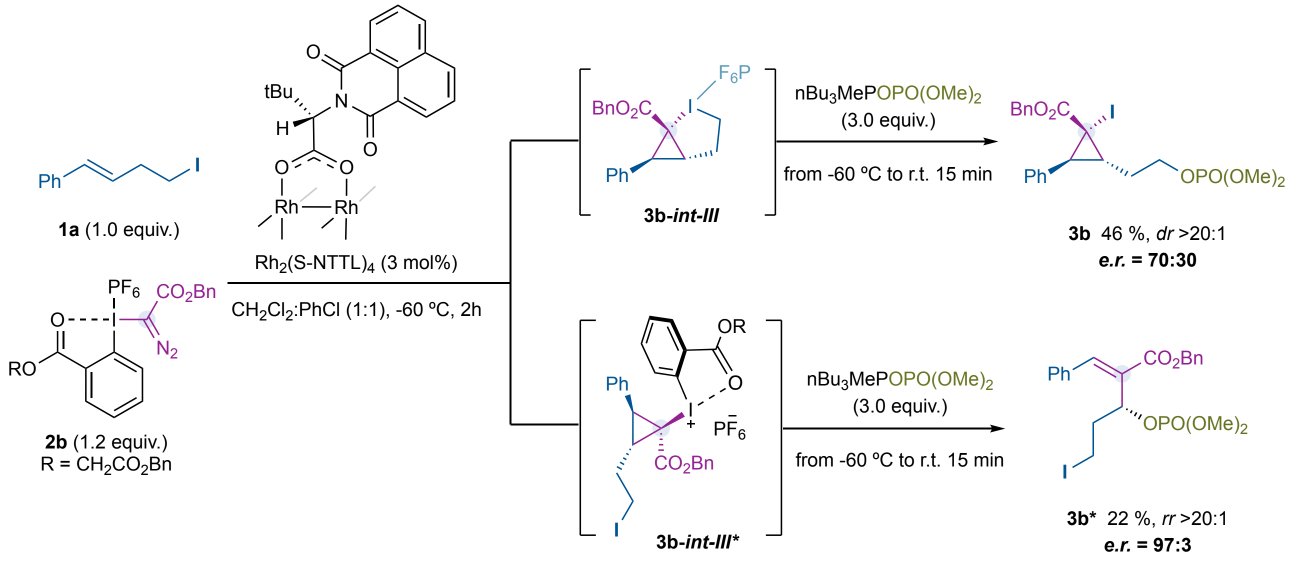

To a 10 mL oven-dried reaction vial equipped with a stirring bar was added Rh_2(S-NTTL)$_4$ (4.8 mg, 0.003 mmol, 3 mol%) and homoallylic iodide **1a** (25.8 mg, 0.1 mmol, 1.0 equiv.). The tube was sealed before being evacuated and backfilled with argon three times. A 1:1 mixture of dichloromethane and chlorobenzene (0.3 mL) was added, and the resulting mixture was cooled at -60 ºC. Then, a solution of reagent **2b** (85.9 mg, 0.12 mmol, 1.2 equiv.) in a mixture of dichloromethane and chlorobenzene (1.0 mL) was added dropwise during 30 minutes using a syringe pump and after stirred for 60 min at -60 ºC. After this, a solution of tributyl(methyl)phosphonium dimethylphosphate (102.7 mg, 0.3 mmol) in dichloromethane (1.0 mL) was added dropwise during 10 minutes. Then the resulting reaction mixture was allowed to warm to room temperature during 1 hour followed by the removal of solvent by rotatory evaporation. The crude residue was dissolved in $CDCl_3$ and analyzed by ^1H NMR spectroscopy using CH_2Br_2 (7 µL, 0.1 mmol) as internal standard. The crude residue was purified by flash column chromatography with hexane / ethyl acetate 1:1 to yield **3b** (24.4 mg, 46 %) and **3b*** (111.7 mg, 22 %)

Enantiomeric ratio of compound **3b** was determined to be 70:30 by SFC analysis on a chiral stationary phase (CHIRALPAK IB-3, 1 mL/min, 10 % methanol, l = 210 nm, tr(major) = 2.346 min, tr(minor) = 2.086 min)

Enantiomeric ratio of compound **3b*** was determined to be 97:3 by SFC analysis on a chiral stationary phase (CHIRALPAK IB-3, 1 mL/min, 10 % isopropanol, l = 210 nm, tr(major) = 2.787 min, tr(minor) = 3.082 min)

3b

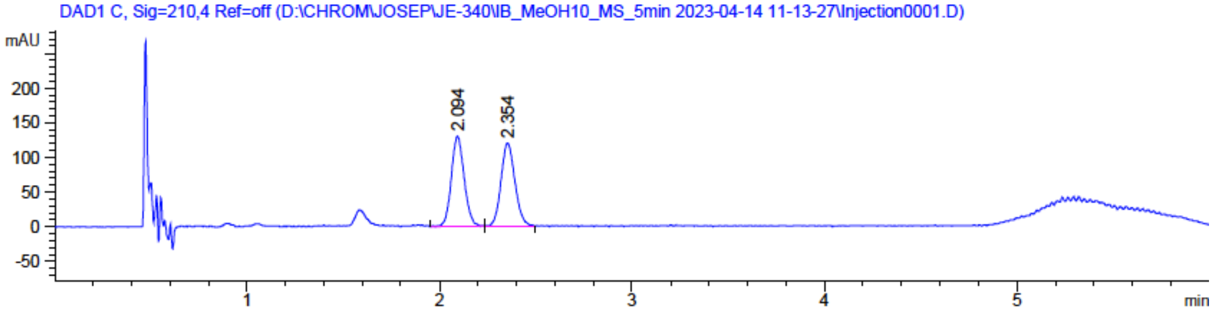

Peak #	RetTime [min]	Type	Width [min]	Area [mAU*s]	Height [mAU]	Area %
1	2.094	VV R	0.0729	600.76581	129.93875	50.0776
2	2.354	VV R	0.0769	598.90302	119.69264	49.9224

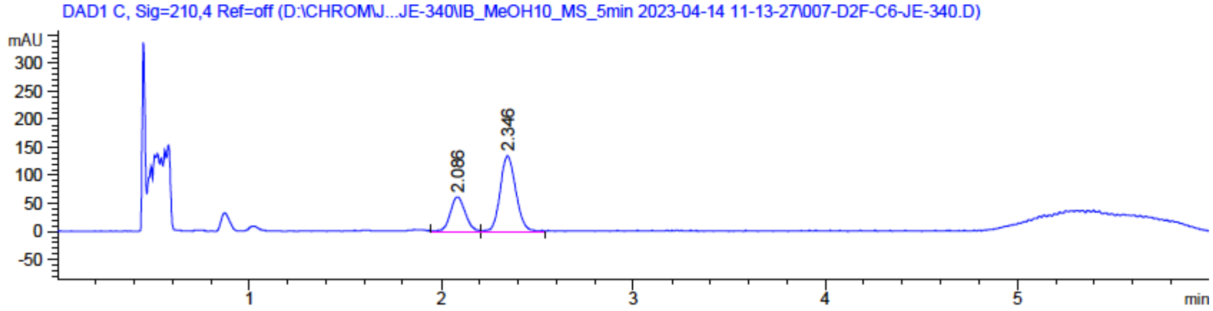

Peak #	RetTime [min]	Type	Width [min]	Area [mAU*s]	Height [mAU]	Area %
1	2.086	BV	0.0833	326.34430	61.07206	30.1523
2	2.346	VV R	0.0861	755.97498	134.35304	69.8477

3b*

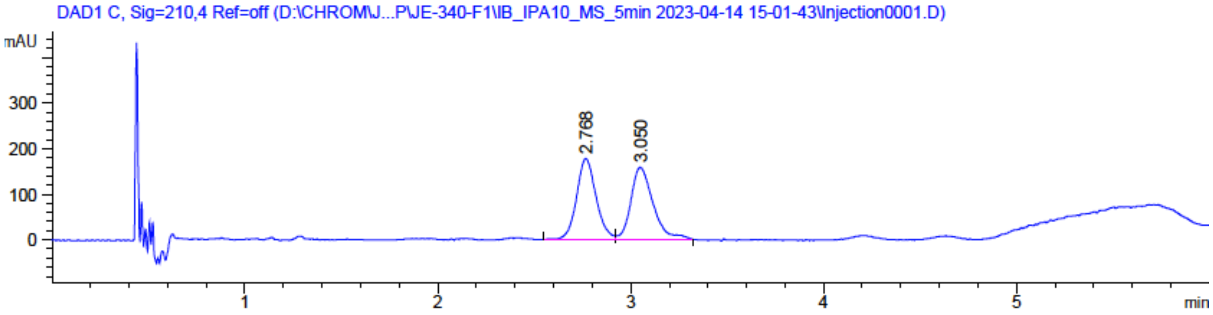

Peak #	RetTime [min]	Type	Width [min]	Area [mAU*s]	Height [mAU]	Area %
1	2.768	VV R	0.1047	1246.92151	176.98619	49.6032
2	3.050	VV R	0.1153	1266.86902	158.36537	50.3968

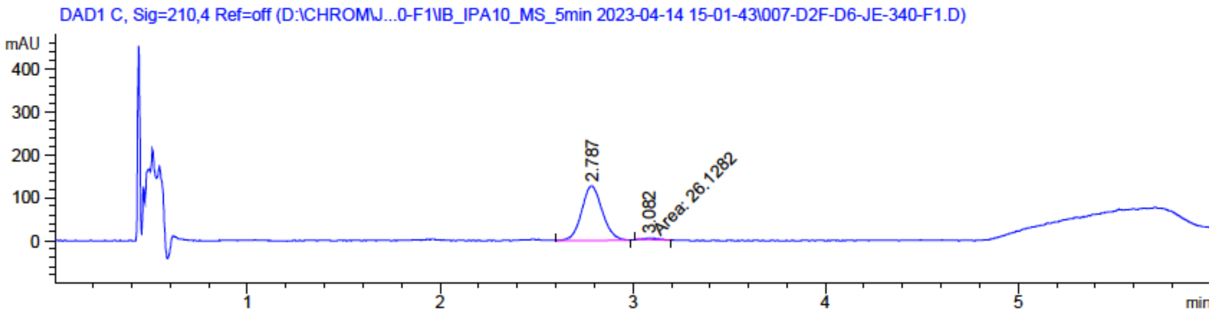

Peak #	RetTime [min]	Type	Width [min]	Area [mAU*s]	Height [mAU]	Area %
1	2.787	BV R	0.1081	938.01727	126.38653	97.2900
2	3.082	MM	0.1094	26.12819	3.98194	2.7100

7. Synthesis of housanes

7.1. Reaction optimization

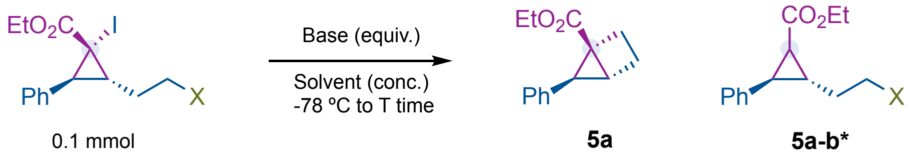

To a 10 mL oven-dried reaction vial equipped with a stirring bar was added the corresponding iodocyclopropane **3** (0.1 mmol) and solvent and the mixture was cooled to -78 ºC and stirred for 15 minutes. Then the base was added dropwise, and the reaction was stirred at -78 ºC for 15 minutes. Then the reaction was warmed up to the corresponding temperature over 1 hour until it was quenched with H_2O (1.0 mL). The aqueous phase was extracted with Et_2O (3 x 2 mL) and the combined organic layers were washed with brine, dried over anhydrous Na_2SO_4 and the solvent removed under rotary evaporation. The crude residue was dissolved in $CDCl_3$ and analyzed by ^1H NMR using CH_2Br_2 (7 µL, 0.1 mmol) as internal standard, with yields calculated relative to CH_2Br_2 and the **5**/**5*** ratio determined by signal integration.

Entry	X	Base (equiv.)	Solvent (conc.)	T (ºC)	time (min)	Yield **5a**	Yield **5***
1	Br	*n*BuLi (1.2)	THF (0.2 M)	-20	60	30	40
2	Br	*n*BuLi (1.2)	Et_2O (0.2 M)	-20	60	10	45
3	Br	*n*BuLi (1.2)	THF (0.05 M)	r.t.	60	33	35
4	Br	*n*BuLi (2.2)	THF (0.05 M)	r.t.	60	35	50
5	Br	*t*BuLi (2.2)	THF (0.05 M)	r.t.	60	4	10
6	Br	LDA (2.2)	THF (0.05 M)	r.t.	60	50	20
7	Br	MeLi (2.2)	THF (0.05 M)	r.t.	60	-	-
8	Br	PhLi (2.2)	THF (0.05 M)	r.t.	60	-	-
9	$OPO(OMe)_2$	LDA (2.2)	THF (0.05 M)	r.t.	60	13	5
10	Cl	LDA (2.2)	THF (0.05M)	r.t.	60	-	-
11	I	LDA (2.2)	THF (0.05M)	r.t.	60	68	-
12	OTf	LDA (2.2)	THF (0.05M)	r.t.	60	63	-
13	I	LDA (2.2)	THF (0.05M)	-78 ºC	0	-	90

nBuLi 2.5 M in hexanes, tBuLi 1.7 M in pentane, MeLi 1.6 M in diethyl ether, PhLi 1.9 M in dibutyl ether, LDA (lithium diisopropyl amide) 2.0 M in tetrahydrofuran.

Ethyl (2*R,3*R**)-2-(2-bromoethyl)-3-phenylcyclopropane-1-carboxylate (5a*)**

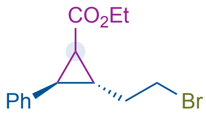

This compound was synthetized following the procedure outlined in section **6.1** using ethyl (1*R**,2*R**,3*S**)-1-iodo-2-(2-bromoethyl)-3-phenylcyclopropane-1-carboxylate **3i** (42.3 mg, 0.1 mmol). Purification by flash column chromatography (hexane/diethyl ether: 100/1 → 50/1) provided the title compound as a mixture of diastereoisomers (8.9 mg, 30% yield).

Diastereomer 1:

^{1}H NMR (400 MHz, $CDCl_3$) δ 7.29 (dd, *J* = 8.2, 6.8 Hz, 2H), 7.24 – 7.18 (m, 1H), 7.12 (dd, *J* = 7.0, 1.7 Hz, 2H), 4.18 (q, *J* = 7.1 Hz, 2H), 3.52 – 3.41 (m, 2H), 2.51 (dd, *J* = 6.7, 5.1 Hz, 1H), 2.43 – 2.24 (m, 2H), 2.09 (dd, *J* = 9.1, 5.0 Hz, 1H), 1.84 (dq, *J* = 9.0, 6.8 Hz, 1H), 1.29 (t, *J* = 7.2 Hz, 3H).

^{13}C NMR (126 MHz, $CDCl_3$) δ 171.8, 139.8, 128.7, 126.7, 126.5, 60.9, 33.1, 31.4, 30.0, 29.5, 28.3, 14.5.

HRMS: (ESI) calculated for $C_{14}H_{17}BrNaO_2$ $[M+Na]^+$ m/z: 319.0305, found: 319.0309.

Diastereomer 2:

^{1}H NMR (500 MHz, $CDCl_3$) δ 7.28 – 7.25 (m, 4H), 7.22 – 7.18 (m, 1H), 3.88 (qd, *J* = 7.1, 1.3 Hz, 2H), 3.54 (td, *J* = 6.7, 2.6 Hz, 2H), 2.46 (dd, *J* = 9.4, 6.7 Hz, 1H), 2.27 – 2.12 (m, 2H), 1.99 – 1.88 (m, 2H), 0.98 (t, *J* = 7.1 Hz, 3H).

^{13}C NMR (126 MHz, $CDCl_3$) δ 170.4, 136.2, 129.4, 128.1, 127.0, 60.5, 36.0, 32.5, 32.0, 28.5, 24.2, 14.1.

HRMS: (APCI) calculated for $C_{14}H_{18}BrO_2$ $[M+H]^+$ m/z: 297.0485, found: 297.0481.

Ethyl (2*R,3*R**)-2-(2-iodoethyl)-3-phenylcyclopropane-1-carboxylate (5b*)**

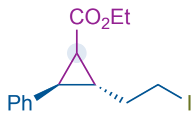

This compound was synthetized following the procedure outlined in section **6.1**, using ethyl (1*R**,2*R**,3*S**)-1-iodo-2-(2-iodoethyl)-3-phenylcyclopropane-1-carboxylate **3j** (47.0 mg, 0.1 mmol). Purification by flash column chromatography (hexane/diethyl ether: 100/1 → 50/1) provided the title compound as pale colourless oil (17.8 mg, 60% yield). Characterization data reported for a single diastereoisomer as just one diastereoisomer could be cleanly isolated.

^{1}H NMR (400 MHz, $CDCl_3$) δ 7.27 (s, 3H), 7.21 (tt, *J* = 8.7, 4.0 Hz, 2H), 3.94 – 3.83 (m, 2H), 3.30 (ddd, *J* = 7.0, 5.5, 2.0 Hz, 2H), 2.45 (dd, *J* = 9.5, 6.3 Hz, 1H), 2.22 – 2.11 (m, 2H), 2.00 – 1.86 (m, 2H), 0.98 (td, *J* = 7.2, 0.8 Hz, 3H).

^{13}C NMR (101 MHz, $CDCl_3$) δ 170.4, 136.2, 129.4, 128.1, 127.0, 60.5, 36.8, 32.5, 28.4, 26.2, 14.2, 3.8.

HRMS: (ESI) calculated for $C_{14}H_{18}IO_2$ $[M+H]^+$ m/z: 345.0346, found: 345.0353.

7.2. Housane scope

General Procedure G:

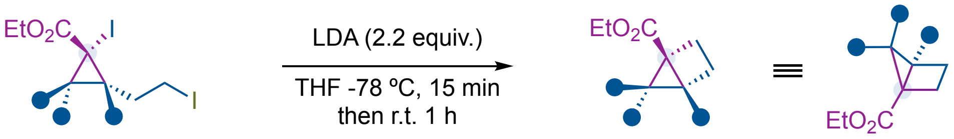

3 **5**

To a 10 mL oven-dried reaction vial equipped with a stirring bar was added the corresponding iodocyclopropane **3** (0.1 mmol) and THF (2.0 mL) and the mixture was cooled down to -78 ºC and stirred for 15 minutes. Then lithium diisopropylamide (0.11 mL, 0.22 mmol, 2.0 M in THF) was added dropwise and the reaction was stirred at -78 ºC for 15 minutes. Then the reaction was warmed up to room temperature over 1 hour until it was quenched with H_2O (1.0 mL). The aqueous phase was extracted with Et_2O (3 x 2 mL) and the combined organic layers were washed with brine, dried over anhydrous Na_2SO_4 and the solvent removed under rotary evaporation. The crude residue was purified by flash column chromatography to obtain housanes **5**. All diastereomeric ratios were >20:1 unless otherwise stated.

ethyl (1*S,4*R**,5*S**)-5-phenylbicyclo[2.1.0]pentane-1-carboxylate (5a)**

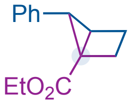

This compound was synthetized following the general procedure **G** using ethyl (1*R**,2*R**,3*S**)-1-iodo-2-(2-iodoethyl)-3-phenylcyclopropane-1-carboxylate **3j** (47.0 mg, 0.1 mmol). Purification by flash column chromatography (hexane/diethyl ether: 100/1 → 50/1) provided the title compound as colourless oil (15.1 mg, 70% yield).

^{1}H NMR (400 MHz, $CDCl_3$) δ 7.28 – 7.16 (m, 5H), 3.83 (m, 2H), 2.83 (d, *J* = 2.6 Hz, 1H), 2.81 (ddd, *J* = 5.1, 2.6, 1.3 Hz, 1H), 2.53 (td, *J* = 11.1, 4.1 Hz, 1H), 2.25 (tt, *J* = 11.1, 4.6 Hz, 1H), 1.96 (dddd, *J* = 11.1, 6.2, 4.6, 1.3 Hz, 1H), 1.62 – 1.56 (m, 1H), 0.86 (t, *J* = 7.1 Hz, 3H).

^{13}C NMR (101 MHz, $CDCl_3$) δ 172.0, 136.1, 129.2, 127.9, 126.7, 59.9, 42.9, 33.6, 30.1, 26.3, 20.9, 13.9.

HRMS: (ESI) calculated for $C_{14}H_{16}NaO_2$ $[M+Na]^+$ m/z: 239.1043, found: 239.1049.

^{1}H – ^{1}H NOESY spectrum was measured.

Ethyl (1*S,4*R**,5*S**)-5-(4-fluorophenyl)bicyclo [2.1.0]pentane-1-carboxylate (5b)**

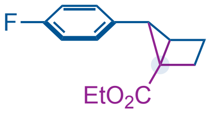

This compound was synthetized following the general procedure **G** using ethyl (1*R**,2*S**,3*R**)-2-(4-fluorophenyl)-1-iodo-3-(2-iodoethyl)cyclopropane-1-carboxylate **3ad** (48.8 mg, 0.1 mmol). Purification by flash column chromatography (hexane/ethyl acetate: 100/0 → 97/3) provided the title compound as colourless oil (17.1 mg, 73% yield).

^{1}H NMR (400 MHz, $CDCl_3$) δ 7.21 – 7.15 (m, 2H), 7.01 – 6.89 (m, 2H), 3.89 (dq, *J* = 10.8, 7.2 Hz, 1H), 3.83 (dq, *J* = 10.8, 7.2 Hz, 1H), 2.79 (d, *J* = 2.6 Hz, 1H), 2.75 (ddt, *J* = 4.9, 2.5, 1.0 Hz, 1H), 2.53 (td, *J* = 11.2, 4.1 Hz, 1H), 2.25 (tt, *J* = 11.1, 4.7 Hz, 1H), 1.94 (dddd, *J* = 11.2, 6.2, 4.6, 1.4 Hz, 1H), 1.57 (dddd, *J* = 11.1, 6.3, 4.2, 0.7 Hz, 1H), 0.91 (t, *J* = 7.1 Hz, 3H).

^{13}C NMR (101 MHz, $CDCl_3$) δ 171.9, 161.8 (d, *J* = 244.4 Hz), 131.8 (d, *J* = 3.3 Hz), 130.7 (d, *J* = 8.1 Hz), 114.7 (d, *J* = 21.2 Hz), 60.0, 42.1, 33.5, 30.3, 26.2, 20.9, 14.0.

^{19}F NMR (471 MHz, $CDCl_3$) δ -116.30.

HRMS: (ESI) calculated for $C_{14}H_{16}FO_2$ $[M+H]^+$ m/z: 235.1129, found: 235.1132.

Ethyl (1*S,4*R**,5*S**)-5-(4-chlorophenyl)bicyclo[2.1.0]pentane-1-carboxylate (5c)**

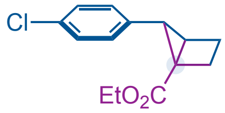

This compound was synthetized following the general procedure **G** using Ethyl (1*R**,2*S**,3*R**)-2-(4-chlorophenyl)-1-iodo-3-(2-iodoethyl)cyclopropane-1-carboxylate **3ae** (50.5 mg, 0.1 mmol). Purification by flash column chromatography (hexane/ethyl acetate: 100/0 → 97/3) provided the title compound as colourless oil (18.8 mg, 75% yield).

^{1}H NMR (400 MHz, $CDCl_3$) δ 7.25 – 7.19 (m, 2H), 7.17 – 7.12 (m, 2H), 3.90 (dq, *J* = 10.8, 7.2 Hz, 1H), 3.84 (dq, *J* = 10.8, 7.2 Hz, 1H), 2.78 (d, *J* = 2.7 Hz, 1H), 2.77 – 2.73 (m, 1H), 2.54 (td, *J* = 11.2, 4.1 Hz, 1H), 2.25 (tt, *J* = 11.1, 4.6 Hz, 1H), 1.94 (dddd, *J* = 12.2, 6.1, 4.7, 1.2 Hz, 1H), 1.58 (ddd, *J* = 11.2, 6.6, 4.4 Hz, 1H), 0.93 (t, *J* = 7.1 Hz, 3H).

^{13}C NMR (101 MHz, $CDCl_3$) δ 171.7, 134.6, 132.4, 130.5, 128.0, 60.1, 42.1, 33.7, 30.3, 26.2, 20.9, 14.0.

HRMS: (ESI) calculated for $C_{14}H_{16}ClO_2$ $[M+H]^+$ m/z: 251.0833, found: 251.0838.

Ethyl (1*S,4*R**,5*S**)-5-(4-(*tert*-butyl)phenyl)bicyclo[2.1.0]pentane-1-carboxylate (5d)**

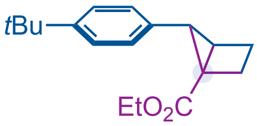

This compound was synthetized following the general procedure **G** using Ethyl (1*R**,2*S**,3*R**)-2-(4-(*tert*-butyl)phenyl)-1-iodo-3-(2-iodoethyl)cyclopropane-1-carboxylate **3af** (52.6 mg, 0.1 mmol). Purification by flash column chromatography (hexane/ethyl acetate: 100/0 → 97/3) provided the title compound as colourless oil (19.5 mg, 72% yield).

^{1}H NMR (500 MHz, $CDCl_3$) δ 7.30 – 7.24 (m, 2H), 7.18 – 7.11 (m, 2H), 3.87 (dq, *J* = 10.8, 7.1 Hz, 1H), 3.76 (dq, *J* = 10.8, 7.1 Hz, 1H), 2.80 (d, *J* = 2.6 Hz, 1H), 2.78 (ddt, *J* = 4.8, 2.5, 1.0 Hz, 1H), 2.52 (td, *J* = 11.2, 4.1 Hz, 1H), 2.24 (tt, *J* = 11.1, 4.7 Hz, 1H), 1.94 (dddd, *J* = 11.2, 6.2, 4.6, 1.4 Hz, 1H), 1.59 – 1.54 (m, 1H), 1.29 (s, 9H), 0.80 (t, *J* = 7.1 Hz, 3H).

^{13}C NMR (126 MHz, $CDCl_3$) δ 172.3, 149.5, 133.0, 128.9, 124.8, 59.8, 42.6, 34.5, 33.4, 31.5, 30.2, 26.3, 20.9, 13.8.

HRMS: (ESI) calculated for $C_{18}H_{25}O_2$ $[M+H]^+$ m/z: 273.1849, found: 273.1852.

Ethyl (1*S,4*R**,5*S**)-5-(*m*-tolyl)bicyclo[2.1.0]pentane-1-carboxylate (5e)**

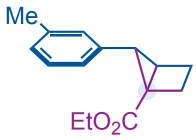

This compound was synthetized following the general procedure **G** using Ethyl (1*R**,2*R**,3*S**)-1-iodo-2-(2-iodoethyl)-3-(*m*-tolyl)cyclopropane-1-carboxylate **3ah** (48.4 mg, 0.1 mmol). Purification by flash column chromatography (hexane/ethyl acetate: 100/0 → 97/3) provided the title compound as colourless oil (15.2 mg, 66% yield).

^{1}H NMR (400 MHz, $CDCl_3$) δ 7.14 (t, J = 7.5 Hz, 1H), 7.04 (s, 1H), 7.02 – 6.98 (m, 2H), 3.87 (dq, J = 10.4, 6.9 Hz, 1H), 3.81 (dq, J = 10.5, 7.0 Hz, 1H), 2.80 – 2.77 (m, 2H), 2.52 (td, J = 11.2, 4.1 Hz, 1H), 2.31 (s, 3H), 2.24 (tt, J = 11.0, 4.4 Hz, 1H), 1.95 (dddd, J = 11.5, 6.1, 4.7, 1.2 Hz, 1H), 1.57 (ddd, J = 10.4, 6.2, 4.0 Hz, 1H), 0.88 (t, J = 7.1 Hz, 3H).

^{13}C NMR (101 MHz, $CDCl_3$) δ 172.1, 137.3, 135.9, 130.0, 127.8, 127.4, 126.2, 59.9, 42.9, 33.5, 30.0, 26.3, 21.5, 20.9, 13.9.

HRMS: (ESI) calculated for $C_{15}H_{18}NaO_2$ $[M+Na]^+$ m/z: 253.1199, found: 253.1203.

Ethyl (1*S,4*R**,5*S**)-5-(*o*-tolyl)bicyclo[2.1.0]pentane-1-carboxylate (5f)**

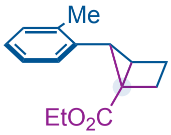

This compound was synthetized following the general procedure **G** using Ethyl (1*R**,2*R**,3*S**)-1-iodo-2-(2-iodoethyl)-3-(*o*-tolyl)cyclopropane-1-carboxylate **3ai** (48.4 g, 0.1 mmol). Purification by flash column chromatography (hexane/ethyl acetate: 100/0 → 97/3) provided the title compound as colourless oil (17.5 mg, 76% yield).

^{1}H NMR (400 MHz, $CDCl_3$) δ 7.14 – 7.09 (m, 4H), 3.83 (dq, J = 10.7, 7.1 Hz, 1H), 3.73 (dq, J = 10.8, 7.1 Hz, 1H), 2.90 (ddt, J = 5.0, 2.4, 1.1 Hz, 1H), 2.71 (d, J = 2.8 Hz, 1H), 2.56 (td, J = 11.2, 4.1 Hz, 1H), 2.29 (tt, J = 11.0, 4.7 Hz, 1H), 2.29 (s, 3H), 1.98 (dddd, J = 11.2, 6.1, 4.6, 1.3 Hz, 1H), 1.60 (dddd, J = 11.2, 6.4, 4.1, 1.0 Hz, 1H), 0.77 (t, J = 7.1 Hz, 3H).

^{13}C NMR (101 MHz, $CDCl_3$) δ 172.3, 138.3, 134.8, 129.5, 128.4, 126.9, 125.5, 59.8, 41.9, 32.1, 30.0, 25.7, 20.9, 19.5, 13.8.

HRMS: (ESI) calculated for $C_{15}H_{18}NaO_2$ $[M+Na]^+$ m/z: 253.1199, found: 253.1189.

Ethyl (1*S,4*R**,5*S**)-5-(3-fluoro-4-methylphenyl)bicyclo[2.1.0]pentane-1-carboxylate (5g)**

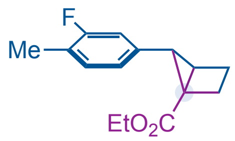

This compound was synthetized following the general procedure **G** using Ethyl (1*R**,2*S**,3*R**)-2-(3-fluoro-4-methylphenyl)-1-iodo-3-(2-iodoethyl)cyclopropane-1-carboxylate **3ag** (50.2 mg, 0.1 mmol). Purification by flash column chromatography (hexane/ethyl acetate: 100/0 → 97/3) provided the title compound as colourless oil (17.9 mg, 72% yield).

^{1}H NMR (500 MHz, $CDCl_3$) δ 7.04 (t, *J* = 7.5 Hz, 1H), 6.89 (dd, *J* = 7.7, 1.8 Hz, 1H), 6.86 (dd, *J* = 11.0, 1.8 Hz, 1H), 3.91 (dq, *J* = 10.9, 7.1 Hz, 1H), 3.85 (dq, *J* = 10.8, 7.1 Hz, 1H), 2.77 (d, *J* = 2.6 Hz, 1H), 2.74 (ddt, *J* = 4.8, 2.4, 1.1 Hz, 1H), 2.52 (td, *J* = 11.2, 4.1 Hz, 1H), 2.25 (tt, *J* = 11.1, 4.7 Hz, 1H), 2.22 (d, *J* = 1.9 Hz, 3H), 1.93 (dddd, *J* = 11.2, 6.2, 4.6, 1.4 Hz, 1H), 1.57 (dddd, *J* = 11.3, 6.4, 4.0, 1.0 Hz, 1H), 0.94 (t, *J* = 7.1 Hz, 3H).

^{13}C NMR (126 MHz, $CDCl_3$) δ 171.8, 160.9 (d, *J* = 244.4 Hz), 135.7 (d, *J* = 7.8 Hz), 130.7 (d, *J* = 6.0 Hz), 124.6 (d, *J* = 3.2 Hz), 123.0 (d, *J* = 17.0 Hz), 115.7 (d, *J* = 23.0 Hz), 60.1, 42.2, 33.6, 30.2, 26.2, 20.9, 14.4 (d, *J* = 3.2 Hz), 14.0.

^{19}F NMR (471 MHz, $CDCl_3$) δ -118.74.

HRMS: (ESI) calculated for $C_{15}H_{17}FNaO_2$ $[M+Na]^+$ m/z: 271.1105, found: 271.1105.

Ethyl (1*S,4*R**,5*S**)-5-(naphthalen-2-yl)bicyclo[2.1.0]pentane-1-carboxylate (5h)**

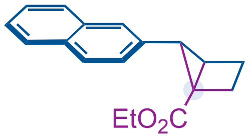

This compound was synthetized following the general procedure **G** using ethyl (1*S**,2*S**,3*R**)-1-iodo-2-(2-iodoethyl)-3-(naphthalen-2-yl)cyclopropane-1-carboxylate **3aj** (52.0 mg, 0.1 mmol). Purification by flash column chromatography (hexane/diethyl ether: 100/1 → 50/1) provided the title compound as colourless oil (22.9 mg, 78% yield).

^{1}H NMR (400 MHz, $CDCl_3$) δ 7.94 – 7.64 (m, 5H), 7.47 – 7.38 (m, 2H), 7.35 (dd, *J* = 8.5, 1.8 Hz, 1H), 3.77 (qd, *J* = 7.1, 4.7 Hz, 2H), 2.99 (d, *J* = 2.6 Hz, 1H), 2.95 (ddd, *J* = 4.1, 2.6, 1.4 Hz, 1H), 2.58 (td, *J* = 11.2, 4.1 Hz, 1H), 2.31 (tt, *J* = 11.2, 4.7 Hz, 1H), 2.01 (dddd, *J* = 11.2, 6.2, 4.7, 1.4 Hz, 1H), 1.64 (ddd, *J* = 10.7, 6.2, 4.2 Hz, 1H), 0.74 (t, *J* = 7.1 Hz, 3H).

^{13}C NMR (101 MHz, $CDCl_3$) δ 172.0, 133.7, 133.3, 132.5, 127.8, 127.7, 127.7, 127.7, 127.2, 126.0, 125.6, 60.0, 43.1, 33.8, 30.4, 26.4, 21.0, 13.9.

HRMS: (ESI) calculated for $C_{18}H_{18}NaO_2$ $[M+Na]^+$ m/z: 289.1199, found: 289.1208.

Ethyl (1*S,4*R**,5*S**)-5-(naphthalen-1-yl)bicyclo[2.1.0]pentane-1-carboxylate (5i)**

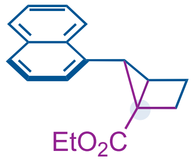

This compound was synthetized following the general procedure **G** using ethyl (1*R**,2*R**,3*S**)-1-iodo-2-(2-iodoethyl)-3-(naphthalen-1-yl)cyclopropane-1-carboxylate **3ak** (52.0 mg, 0.1 mmol). Purification by flash column chromatography (hexane/diethyl ether: 100/1 → 50/1) provided the title compound as colourless oil (20.8 mg, 86% yield).

^{1}H NMR (300 MHz, $CDCl_3$) δ 8.15 – 8.08 (m, 1H), 7.87 – 7.79 (m, 1H), 7.74 (dd, *J* = 7.3, 2.2 Hz, 1H), 7.56 – 7.31 (m, 4H), 3.47 (qd, *J* = 7.1, 4.7 Hz, 2H), 3.16 (d, *J* = 2.6 Hz, 1H), 3.02 (ddt, *J* = 5.0, 2.6, 1.1 Hz, 1H), 2.65 (td, *J* = 11.1, 4.1 Hz, 1H), 2.36 (tt, *J* = 11.1, 4.7 Hz, 1H), 2.16 (dddd, *J* = 11.1, 6.1, 4.7, 1.4 Hz, 1H), 1.78 – 1.67 (m, 1H), 0.34 (t, *J* = 7.1 Hz, 3H).

^{13}C NMR (101 MHz, $CDCl_3$) δ 172.2, 133.6, 133.5, 132.9, 128.5, 127.5, 126.2, 126.2, 125.7, 125.3, 124.0, 59.6, 40.9, 32.4, 30.2, 25.7, 21.0, 13.3.

HRMS: (ESI) calculated for $C_{18}H_{18}NaO_2$ $[M+Na]^+$ m/z: 289.1199, found: 289.1208.

Ethyl (1*R,4*R**)-4-phenylbicyclo[2.1.0]pentane-1-carboxylate (5j)**

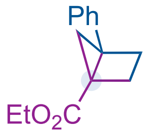

This compound was synthetized following the general procedure **G** using ethyl (1*S**,2*R**)-1-iodo-2-(2-iodoethyl)-2-phenylcyclopropane-1-carboxylate **3an** (47.0 mg, 0.1 mmol). Purification by flash column chromatography (hexane/diethyl ether: 100/1 → 50/1) provided the title compound as colourless oil (12.5 mg, 58% yield).

^{1}H NMR (400 MHz, $CDCl_3$) δ 7.35 – 7.27 (m, 4H), 7.22 – 7.16 (m, 1H), 4.12 – 3.91 (m, 2H), 2.66 – 2.54 (m, 2H), 2.33 (dt, *J* = 4.7, 1.9 Hz, 1H), 1.87 – 1.78 (m, 1H), 1.72 – 1.68 (m, 1H), 1.68 – 1.63 (m, 1H), 1.07 (t, *J* = 7.1 Hz, 3H).

^{13}C NMR (101 MHz, $CDCl_3$) δ 170.9, 138.4, 128.2, 127.3, 126.6, 60.3, 42.8, 35.7, 29.1, 25.2, 21.4, 14.4.

HRMS: (ESI) calculated for $C_{14}H_{16}NaO_2$ $[M+Na]^+$ m/z: 239.1043, found: 239.1036.

Ethyl (1*S,4*R**,5*S**)-5-methyl-5-phenylbicyclo[2.1.0]pentane-1-carboxylate (5k)**

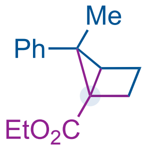

This compound was synthetized following the general procedure **G** using ethyl (1*R**,2*S**,3*R**)-1-iodo-3-(2-iodoethyl)-2-methyl-2-phenylcyclopropane-1-carboxylate **3am** (47.0 mg, 0.1 mmol). Purification by flash column chromatography (hexane/diethyl ether: 100/1 → 50/1) provided the title compound as colourless oil (29.0 mg, 63% yield).

^{1}**H NMR** (400 MHz, $CDCl_3$) δ 7.26 – 7.15 (m, 5H), 3.82 – 3.66 (m, 2H), 2.74 (dt, *J* = 5.0, 1.1 Hz, 1H), 2.44 (ddd, *J* = 11.7, 10.9, 3.9 Hz, 1H), 2.29 – 2.12 (m, 1H), 2.00 (dddd, *J* = 11.7, 5.7, 4.3, 1.3 Hz, 1H), 1.58 (s, 3H), 0.79 (t, *J* = 7.1 Hz, 3H).

^{13}**C NMR** (101 MHz, $CDCl_3$) δ 173.3, 143.3, 129.1, 128.2, 126.5, 59.8, 39.7, 33.9, 32.0, 22.5, 17.3, 16.6, 13.8.

HRMS: (ESI) calculated for $C_{15}H_{18}NaO_2$ $[M+Na]^+$ m/z: 253.1199, found: 253.1191.

Ethyl (1*S,4*R**,5*R**)-5-benzylbicyclo[2.1.0]pentane-1-carboxylate (5l)**

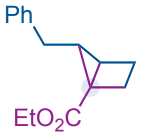

This compound was synthetized following the general procedure **G** using ethyl (1*S**,2*R**,3*R**)-2-benzyl-1-iodo-3-(2-iodoethyl)cyclopropane-1-carboxylate **3aq** (48.4 mg, 0.1 mmol). Purification by flash column chromatography (hexane/ethyl acetate: 100/0 → 97/3) provided the title compound as colourless oil (14.8 mg, 64% yield).

^{1}**H NMR** (400 MHz, $CDCl_3$) δ 7.33 – 7.23 (m, 4H), 7.23 – 7.17 (m, 1H), 4.12 (qd, *J* = 7.1, 1.0 Hz, 2H), 2.97 (dd, *J* = 16.3, 7.3 Hz, 1H), 2.93 (dd, *J* = 16.4, 7.1 Hz, 1H), 2.44 (td, *J* = 11.2, 4.0 Hz, 1H), 2.24 – 2.22 (m, 1H), 2.12 (tt, *J* = 11.1, 4.7 Hz, 1H), 1.85 (td, *J* = 7.2, 2.4 Hz, 1H), 1.73 (dddd, *J* = 11.2, 6.2, 4.7, 1.4 Hz, 1H), 1.43 (dddd, *J* = 10.3, 6.6, 4.1, 0.9 Hz, 1H), 1.21 (t, *J* = 7.1 Hz, 3H).

^{13}**C NMR** (101 MHz, $CDCl_3$) δ 173.4, 141.6, 128.5, 128.4, 126.1, 60.3, 39.8, 32.7, 30.4, 29.9, 25.3, 21.0, 14.5.

HRMS: (ESI) calculated for $C_{15}H_{18}NaO_2$ $[M+Na]^+$ m/z: 253.1199, found: 253.1194.

Ethyl (1*S,4*R**,5*R**)-5-hexylbicyclo[2.1.0]pentane-1-carboxylate (5m)**

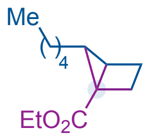

This compound was synthetized following the general procedure **G** using ethyl (1*S**,2*R**,3*R**)-2-hexyl-1-iodo-3-(2-iodoethyl)cyclopropane-1-carboxylate **3ar** (47.8 mg, 0.1 mmol). Purification by flash column chromatography (hexane/ethyl acetate: 100/0 → 98/2) provided the title compound as colourless oil (17.9 mg, 80% yield).

^{1}H NMR (400 MHz, $CDCl_3$) δ 4.15 (dq, *J* = 10.8, 7.1 Hz, 1H), 4.10 (dq, *J* = 10.8, 7.1 Hz, 1H), 2.37 (td, *J* = 11.1, 4.0 Hz, 1H), 2.08 (tt, *J* = 10.9, 4.7 Hz, 1H), 2.03 – 2.00 (m, 1H), 1.68 (dddd, *J* = 11.1, 6.2, 4.5, 1.6 Hz, 1H), 1.56 – 1.51 (m, 2H), 1.41 – 1.21 (m, 9H), 1.38 (ddd, *J* = 10.2, 6.3, 3.7 Hz, 1H), 1.25 (t, *J* = 7.1 Hz, 3H), 0.89 (t, *J* = 6.9 Hz, 3H).

^{13}C NMR (101 MHz, $CDCl_3$) δ 173.7, 60.1, 40.1, 32.7, 32.0, 30.0, 29.9, 29.3, 27.0, 25.4, 22.8, 21.1, 14.5, 14.2.

HRMS: (ESI) calculated for $C_{14}H_{25}O_2$ $[M+H]^+$ m/z: 225.1849, found: 225.1843.

Ethyl (1*S,4*R**)-spiro[bicyclo[2.1.0]pentane-5,1'-cyclohexane]-1-carboxylate (5n)**

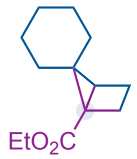

This compound was synthetized following the general procedure **G** with modification, using ethyl (1*R**,2*S**)-1-iodo-2-(2-iodoethyl)spiro[2.5]octane-1-carboxylate **3au** (46.2 mg, 0.1 mmol) and THF/HMPA = 9:1 as solvent. Purification by flash column chromatography (pentane/diethyl ether: 100/0 → 97/3) provided the title compound as colourless oil (8.7 mg, 42% yield).

^{1}H NMR (500 MHz, $CDCl_3$) δ 4.15 (dq, *J* = 10.3, 6.9 Hz, 1H), 4.09 (dq, *J* = 10.8, 6.9 Hz, 1H), 2.32 (td, *J* = 11.3, 3.9 Hz, 1H), 2.05 (dd, *J* = 5.1, 1.4 Hz, 1H), 2.01 (tt, *J* = 11.1, 4.5 Hz, 1H), 1.77 – 1.69 (m, 1H), 1.75 (dddd, *J* = 11.7, 5.8, 4.2, 1.5 Hz, 1H), 1.67 – 1.61 (m, 3H), 1.58 – 1.48 (m, 4H), 1.40 – 1.32 (m, 2H), 1.30 – 1.19 (m, 1H), 1.25 (t, *J* = 7.1 Hz, 3H).

^{13}C NMR (126 MHz, $CDCl_3$) δ 174.2, 60.1, 38.1, 36.1, 33.7, 30.8, 26.9, 26.3, 25.9, 24.3, 22.0, 17.1, 14.5.

HRMS: (ESI) calculated for $C_{13}H_{21}O_2$ $[M+H]^+$ m/z: 209.1536, found: 209.1534.

Phenyl((1*S,4*R**,5*S**)-5-phenylbicyclo[2.1.0]pentan-1-yl)methanone (5o)**

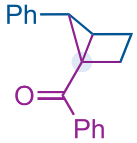

This compound was synthetized following the general procedure **G** using ((1*R**,2*R**,3*S**)-1-iodo-2-(2-iodoethyl)-3-phenylcyclopropyl)(phenyl)methanone **3bc** (50.2 mg, 0.1 mmol). Purification by flash column chromatography (hexane/diethyl ether: 100/1 → 50/1) provided the title compound as colourless oil (9.9 mg, 40% yield).

^{1}H NMR (400 MHz, $CDCl_3$) δ 7.82 – 7.77 (m, 2H), 7.53 – 7.44 (m, 1H), 7.44 – 7.34 (m, 2H), 7.26 (m, 1H), 7.18 (d, *J* = 3.8 Hz, 4H), 7.15 – 7.06 (m, 1H), 3.11 (d, *J* = 2.5 Hz, 1H), 2.90 (dt, *J* = 4.3, 1.7 Hz, 1H), 2.63 – 2.52 (m, 1H), 2.37 – 2.18 (m, 2H), 1.78 – 1.68 (m, 1H).

^{13}C NMR (126 MHz, $CDCl_3$) δ 198.2, 137.2, 135.7, 132.7, 128.6, 128.6, 128.5, 128.1, 126.8, 46.4, 41.7, 29.8, 29.6, 21.4.

HRMS: (ESI) calculated for $C_{18}H_{16}NaO$ $[M+Na]^+$ m/z: 271.1093, found: 271.1091.

Ethyl (1*S,5*R**,6*S**)-6-phenylbicyclo[3.1.0]hexane-1-carboxylate (6)**

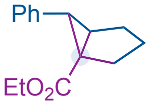

This compound was synthetized following the general procedure **G** using ethyl (1*R**,2*R**,3*S**)-1-iodo-2-(3-iodopropyl)-3-phenylcyclopropane-1-carboxylate (48.4 mg, 0.1 mmol). Purification by flash column chromatography (hexane/ethyl acetate: 100/0 – 97/3) provided the title compound as colourless oil (15.9 mg, 69% yield).

^{1}H NMR (500 MHz, $CDCl_3$) δ 7.28 – 7.22 (m, 4H), 7.21 – 7.16 (m, 1H), 3.81 (qd, *J* = 7.1, 0.7 Hz, 2H), 2.53 – 2.43 (m, 2H), 2.22 (ddd, *J* = 13.0, 8.5, 0.9 Hz, 1H), 2.13 (ddd, *J* = 13.1, 11.0, 8.5 Hz, 1H), 2.05 – 1.91 (m, 2H), 1.82 (dt, *J* = 13.6, 8.1 Hz, 1H), 1.52 – 1.39 (m, 1H), 0.85 (t, *J* = 7.1 Hz, 3H).

^{13}C NMR (126 MHz, $CDCl_3$) δ 172.5, 137.4, 129.3, 127.9, 126.4, 60.0, 40.1, 33.0, 31.0, 29.5, 27.3, 21.6, 13.9.

HRMS: (ESI) calculated for $C_{15}H_{18}NaO_2$ $[M+Na]^+$ m/z: 253.1199, found: 253.1188.

8. Housane derivatization

Synthesis of **(1*S**,4*R**,5*S**)-5-phenylbicyclo[2.1.0]pentane-1-carboxylic acid** (**7**):

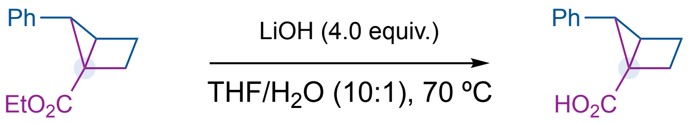

To a 10 mL reaction vial equipped with a stirring bar was added ethyl (1*S**,5*S**)-5-phenylbicyclo[2.1.0]pentane-1-carboxylate **5a** (103.8 mg, 0.48 mmol) and LiOH (46.0 mg, 1.92 mmol). Then THF (5.0 mL) and water (0.5 mL) were added and the reaction was heated at 70 ºC for 16 hours. After this time, the reaction mixture was allowed to cool down to room temperature and extracted with a saturated aqueous solution of $NaHCO_3$ (10 mL) and washed with ethyl acetate (10 mL). The aqueous phase was acidified with 6 M HCl, until pH = 4 and extracted with ethyl acetate (3 x 10 mL). The organic layer was dried over anhydrous Na_2SO_4 and the solvent removed under rotary evaporation to yield **7** as white solid (78 mg, 86% yield).

m.p. = 150 – 152 ºC

^{1}H NMR (400 MHz, $CDCl_3$) δ 7.27 – 7.16 (m, 5H), 2.91 (d, *J* = 2.9 Hz, 1H), 2.83 (dd, *J* = 5.1, 2.9 Hz, 1H), 2.52 (td, *J* = 11.3, 4.1 Hz, 1H), 2.25 (tt, *J* = 11.0, 4.7 Hz, 1H), 1.92 (dtd, *J* = 11.3, 4.8, 2.3 Hz, 1H), 1.58 (ddd, *J* = 11.0, 6.5, 4.2 Hz, 1H).

^{13}C NMR (101 MHz, $CDCl_3$) δ 177.5, 135.3, 129.2, 128.0, 126.9, 44.0, 33.2, 31.6, 26.1, 20.9.

HRMS: (ESI) calculated for $C_{12}H_{12}NaO_2$ $[M+Na]^+$ m/z: 211.0730, found: 211.0725.

The crystal structure of **7** has been deposited at the Cambridge Crystallographic Data Centre, **CCDC** 2444608

9. References

(1) Das, A.; Chen, Y.-S.; Reibenspies, J. H.; Powers, D. C. Characterization of a Reactive Rh_2 Nitrenoid by Crystalline Matrix Isolation. *J. Am. Chem. Soc.* **2019**, *141* (41), 16232–16236.

(2) Ashtekar, K. D.; Gholami, H.; Moemeni, M.; Chakraborty, A.; Kiiskila, L.; Ding, X.; Toma, E.; Rahn, C.; Borhan, B. A Mechanistically Inspired Halenium Ion Initiated Spiroketalization: Entry to Mono- and Dibromospiroketals. *Angew. Chem. Int. Ed.* **2022**, *61* (8), e202115173.

(3) Leškovskis, K.; Gulbe, K.; Mishnev, A.; Turks, M. Ring Opening of Methylenecyclopropanes with Halides in Liquid Sulfur Dioxide. *Tetrahedron Letters* **2020**, *61* (46), 152528.

(4) Lacey, K. D.; Quarels, R. D.; Du, S.; Fulton, A.; Reid, N. J.; Firesheets, A.; Ragains, J. R. Acid-Catalyzed *O* -Glycosylation with Stable Thioglycoside Donors. *Org. Lett.* **2018**, *20* (17), 5181–5185. https://doi.org/10.1021/acs.orglett.8b02125.

(5) Yalavac, I.; Lyons, S. E.; Webb, M. R.; Procter, D. J. SmI_2 –H_2 O-Mediated 5-Exo/6-Exo Lactone Radical Cyclisation Cascades. *Chem. Commun.* **2014**, *50* (85), 12863–12866.

(6) Harada, S.; Matsuda, D.; Morikawa, T.; Nishida, A. Direct Synthesis of Enones by Visible-Light-Promoted Oxygenation of Trisubstituted Olefins Using Molecular Oxygen. *Synlett* **2020**, *31* (14), 1372–1377.

(7) Ghorai, P.; Dussault, P. H.; Hu, C. Synthesis of Spiro-Bisperoxyketals. *Org. Lett.* **2008**, *10* (12), 2401–2404.

(8) Taber, D. F.; Guo, P.; Guo, N. Intramolecular [1 + 4 + 1] Cycloaddition: Establishment of the Method. *J. Am. Chem. Soc.* **2010**, *132* (32), 11179–11182.

(9) Bellido, M.; Riego-Mejías, C.; Diaz-Moreno, A.; Verdaguer, X.; Riera, A. Enantioselective Ir-Catalyzed Hydrogenation of Terminal Homoallyl Sulfones: Total Synthesis of (−)-Curcumene. *Org. Lett.* **2023**, *25* (9), 1453–1457.

(10) Teo, W. J.; Esteve Guasch, J.; Jiang, L.; Li, B.; Suero, M. G. Rh-Catalyzed Enantioselective Single-Carbon Insertion of Alkenes. *J. Am. Chem. Soc.* **2024**, *146* (31), 21837–21846.

(11) Wu, F.-P.; Chintawar, C. C.; Lalisse, R.; Mukherjee, P.; Dutta, S.; Tyler, J.; Daniliuc, C. G.; Gutierrez, O.; Glorius, F. Ring Expansion of Indene by Photoredox-Enabled Functionalized Carbon-Atom Insertion. *Nat. Catal.* **2024**, *7* (3), 242–251.

(12) Schnaars, C.; Hennum, M.; Bonge-Hansen, T. Nucleophilic Halogenations of Diazo Compounds, a Complementary Principle for the Synthesis of Halodiazo Compounds: Experimental and Theoretical Studies. *J. Org. Chem.* **2013**, *78* (15), 7488–7497.

10. Copies of NMR spectra

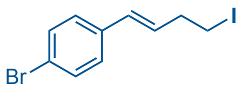

¹H NMR of **1b** (400 MHz, CDCl₃)

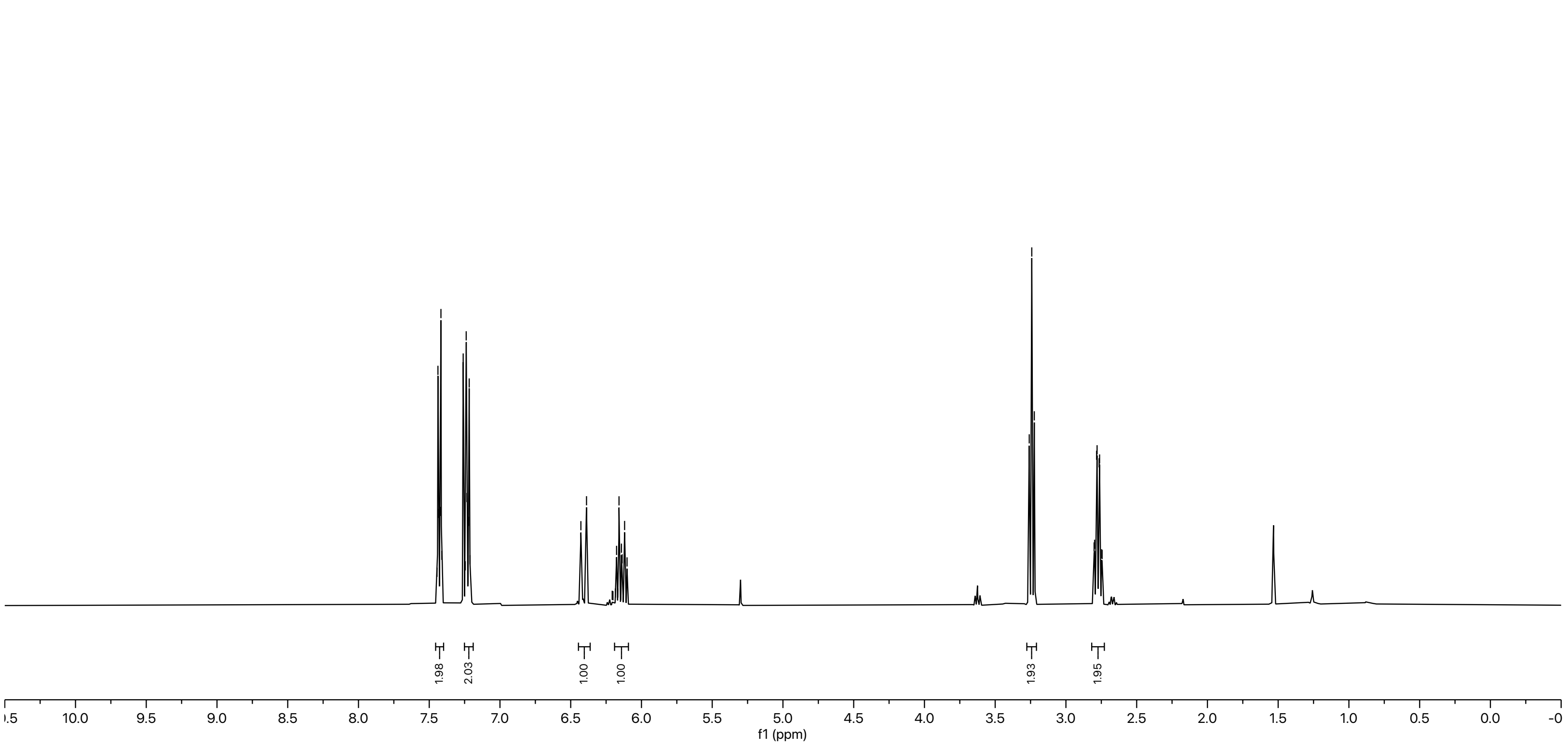

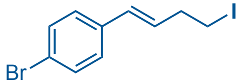

^{13}C NMR of **1b** (101 MHz, $CDCl_3$)

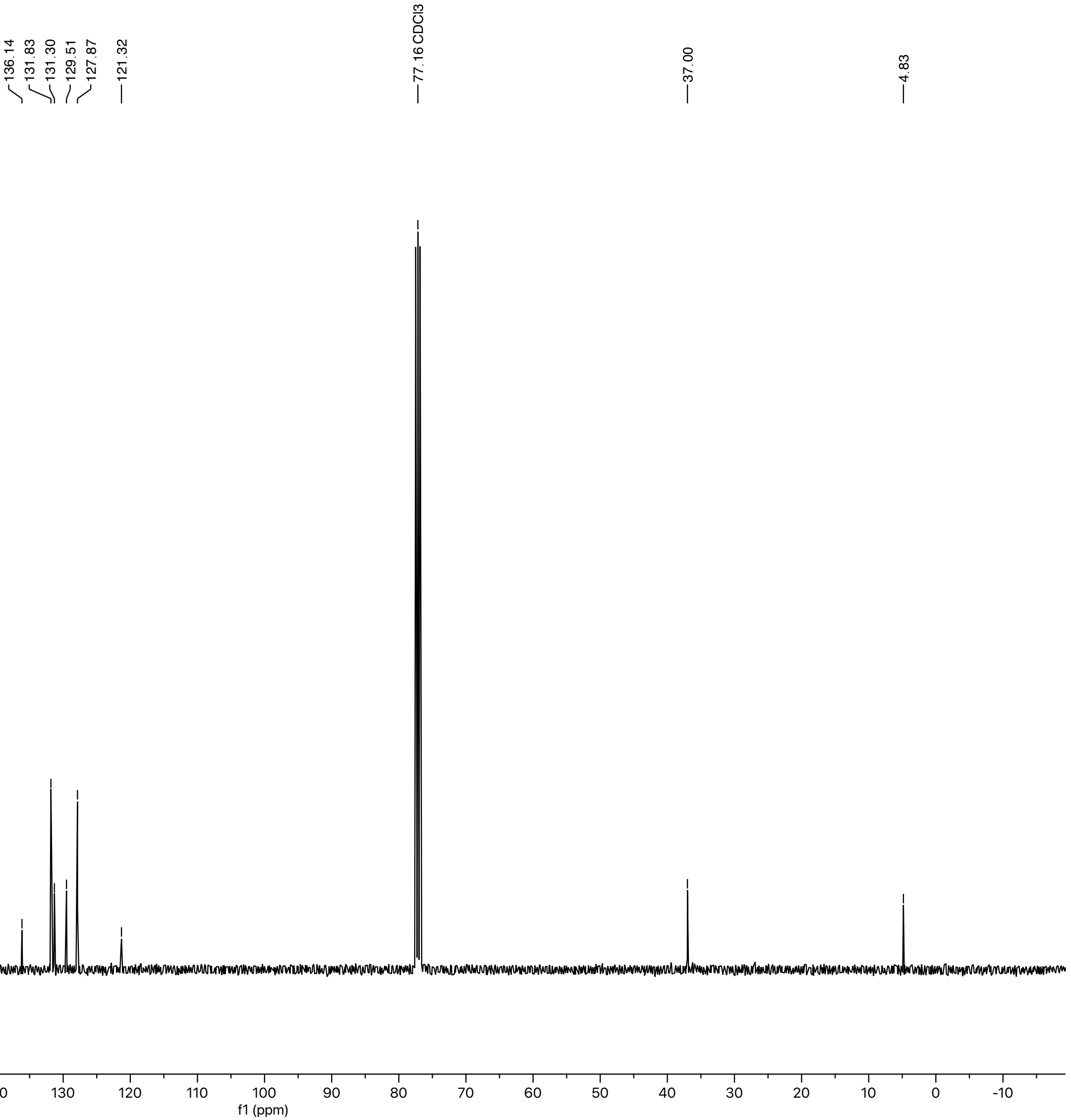

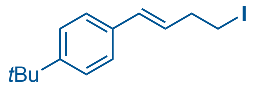

¹H NMR of **1g** (500 MHz, $CDCl_3$)

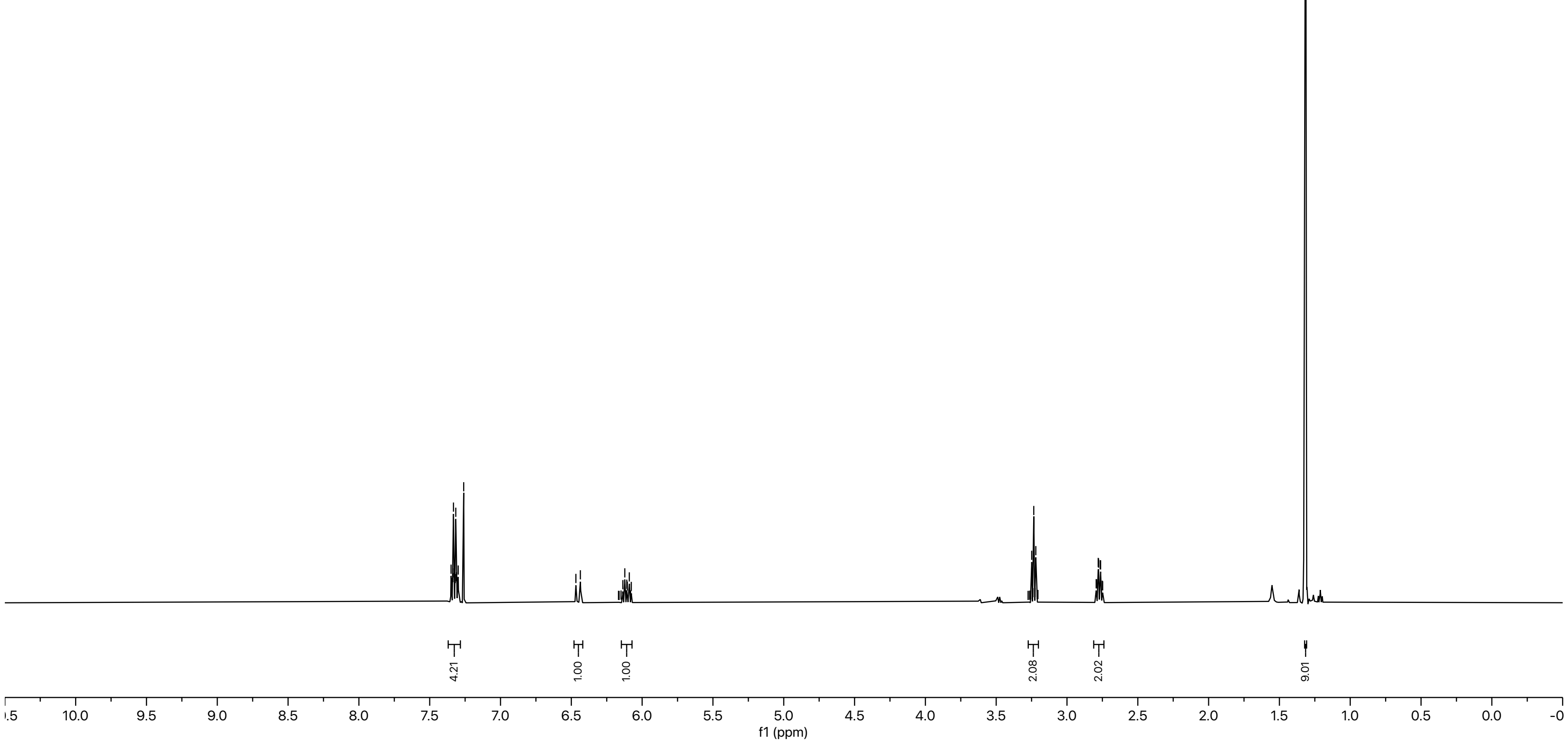

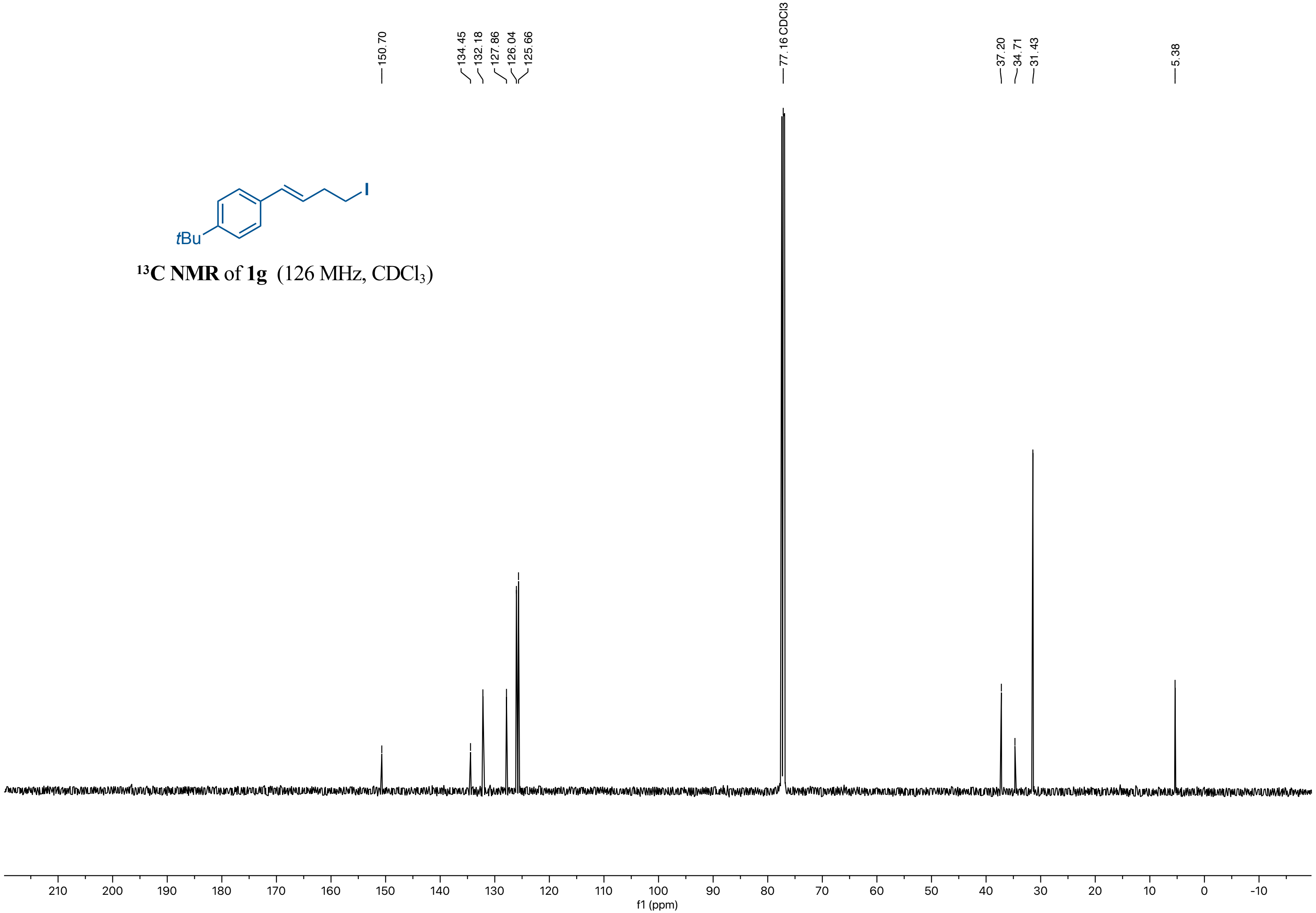

^{13}C NMR of **1g** (126 MHz, $CDCl_3$)

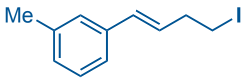

^{1}H NMR of **1i** (400 MHz, $CDCl_3$)

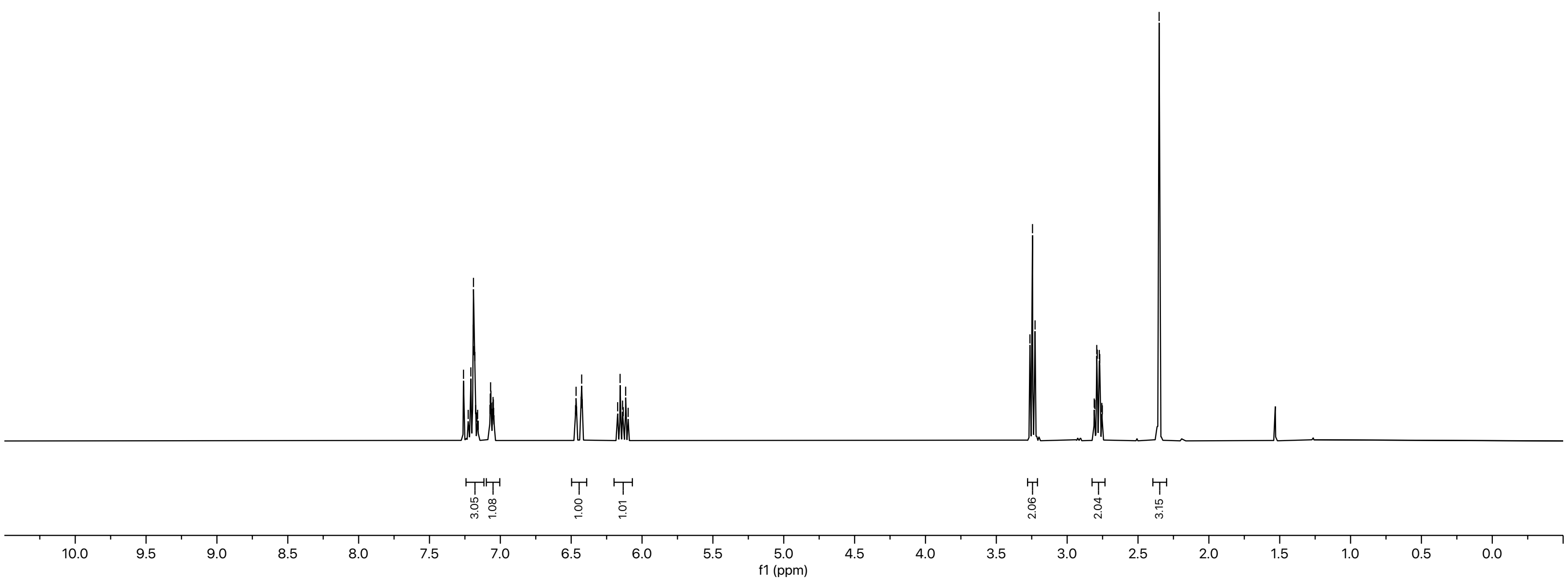

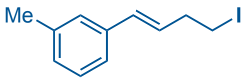

^{13}C NMR of **1i** (126 MHz, $CDCl_3$)

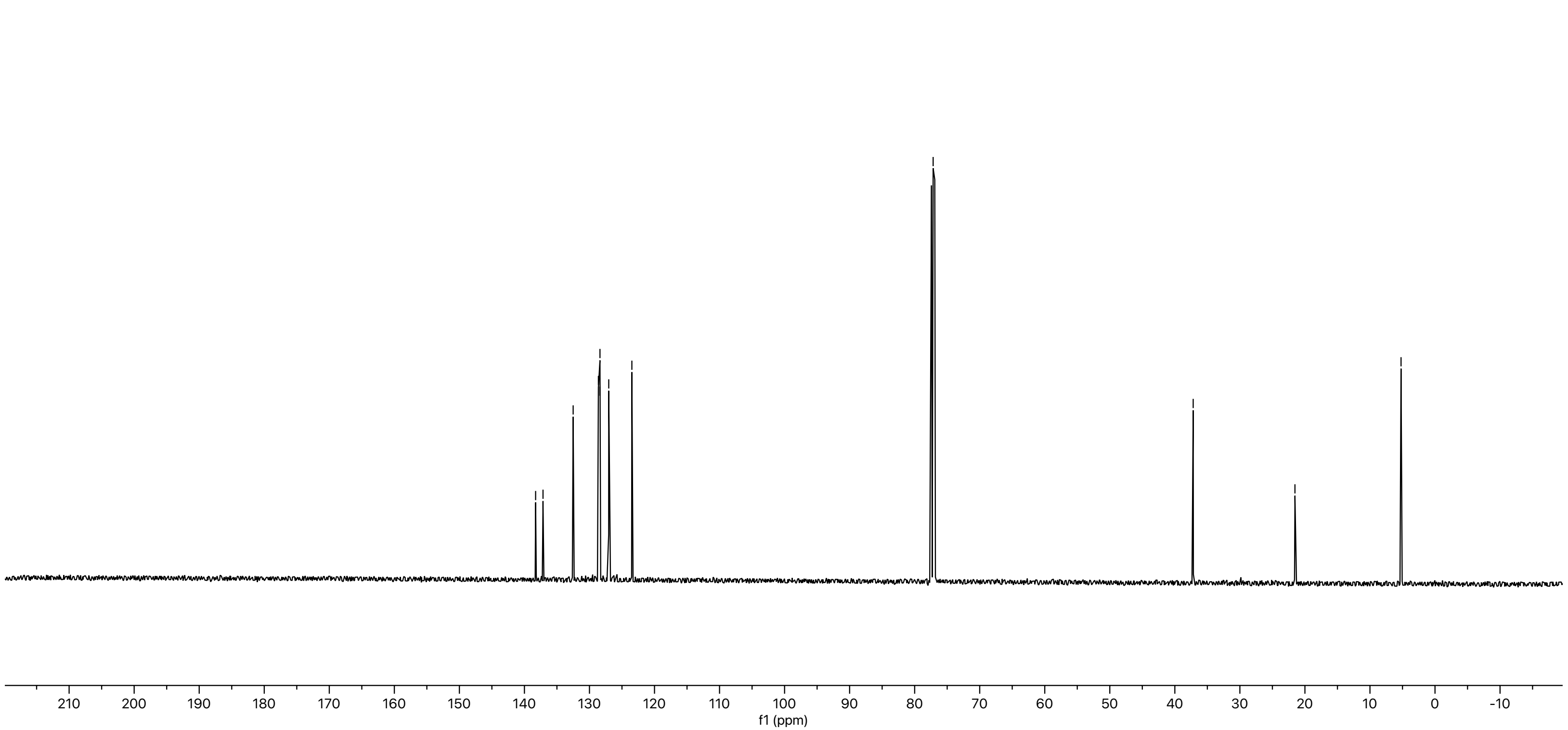

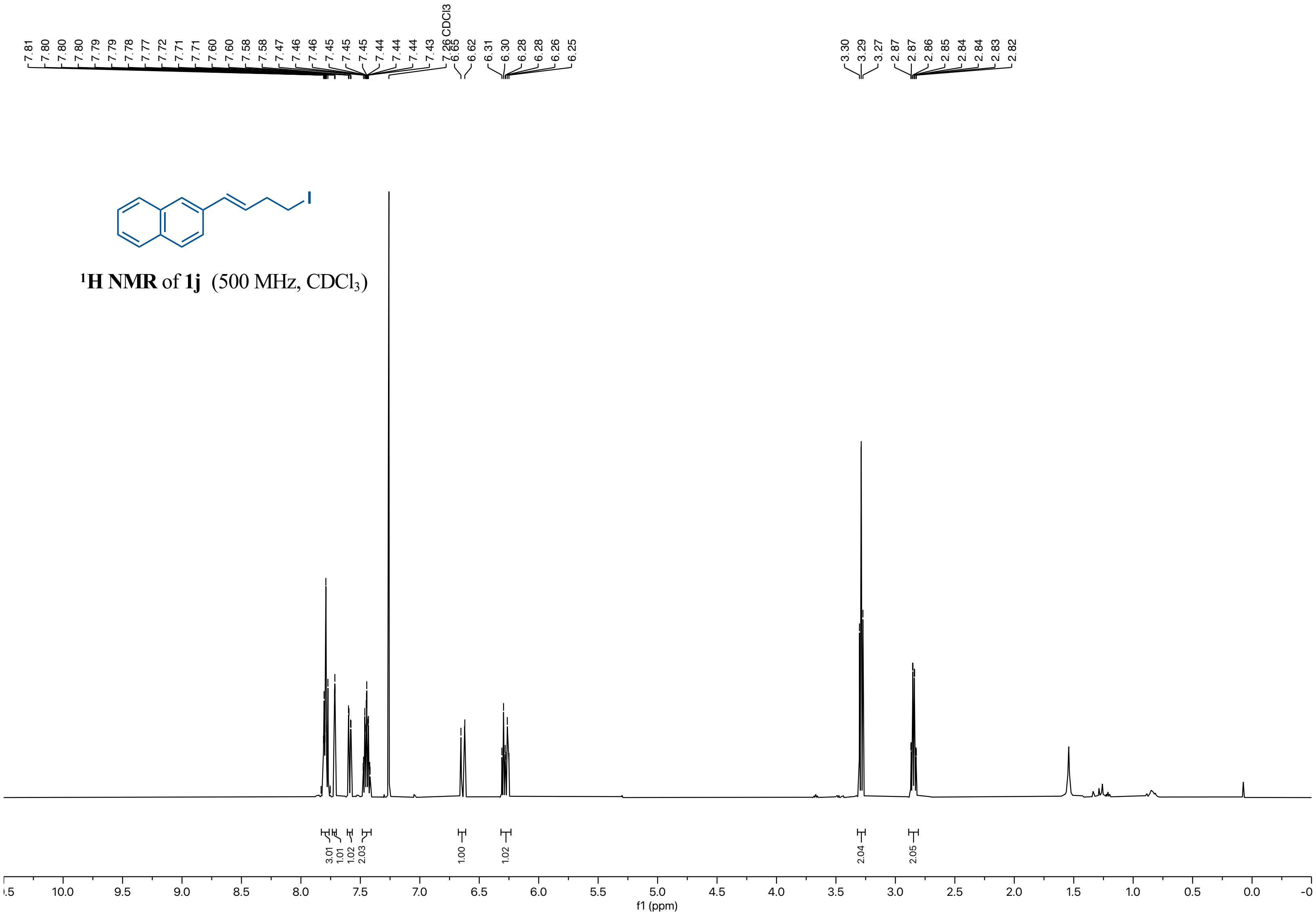

^{1}H NMR of **1j** (500 MHz, $CDCl_3$)

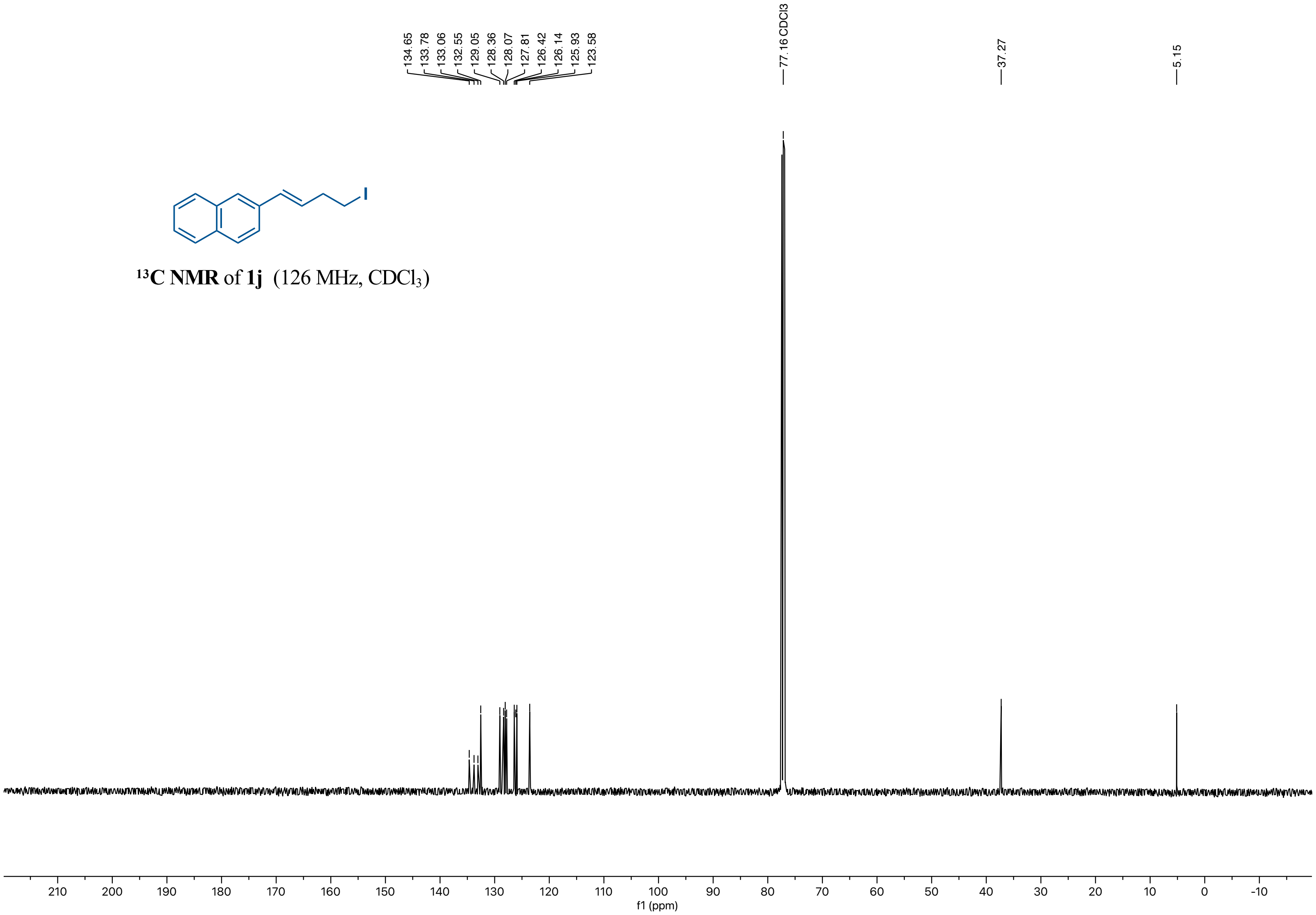

^{13}C NMR of 1j (126 MHz, CDCl$_3$)

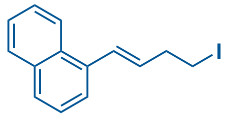

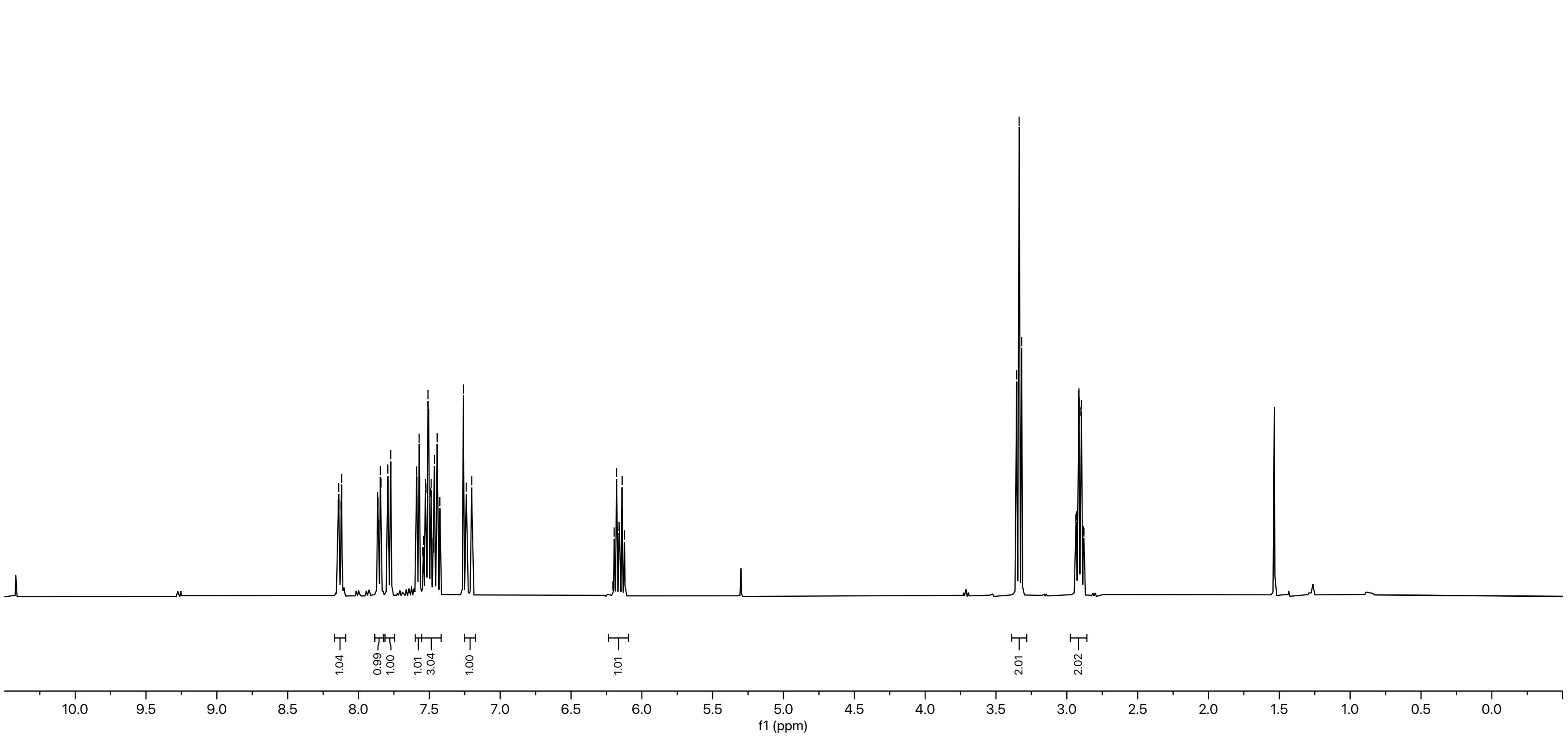

^{1}**H NMR** of **1k** (400 MHz, $CDCl_3$)

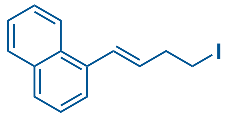

^{13}**C NMR** of **1k** (101 MHz, $CDCl_3$)

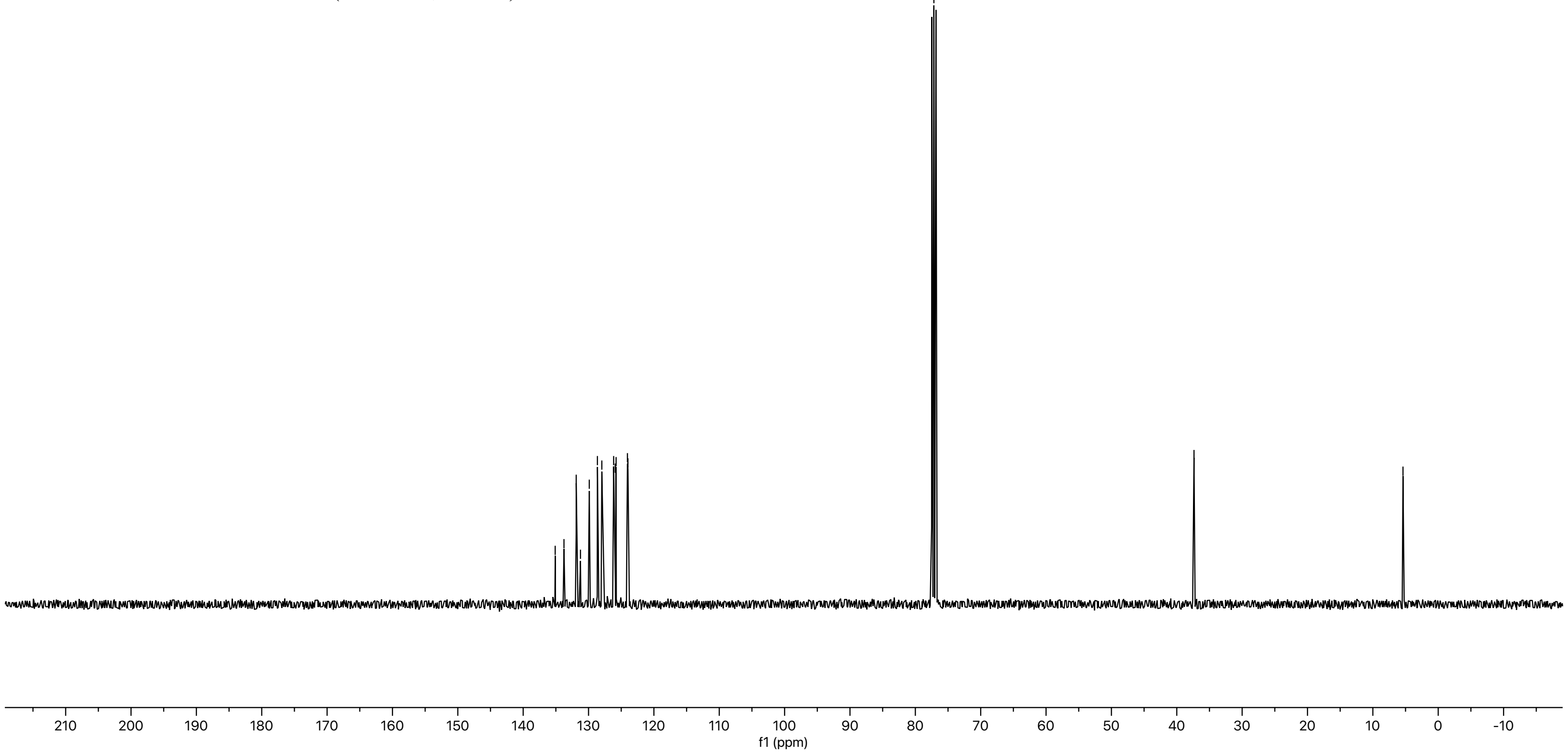

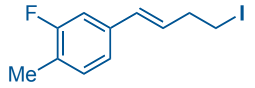

^{1}**H NMR** of **1l** (400 MHz, $CDCl_3$)

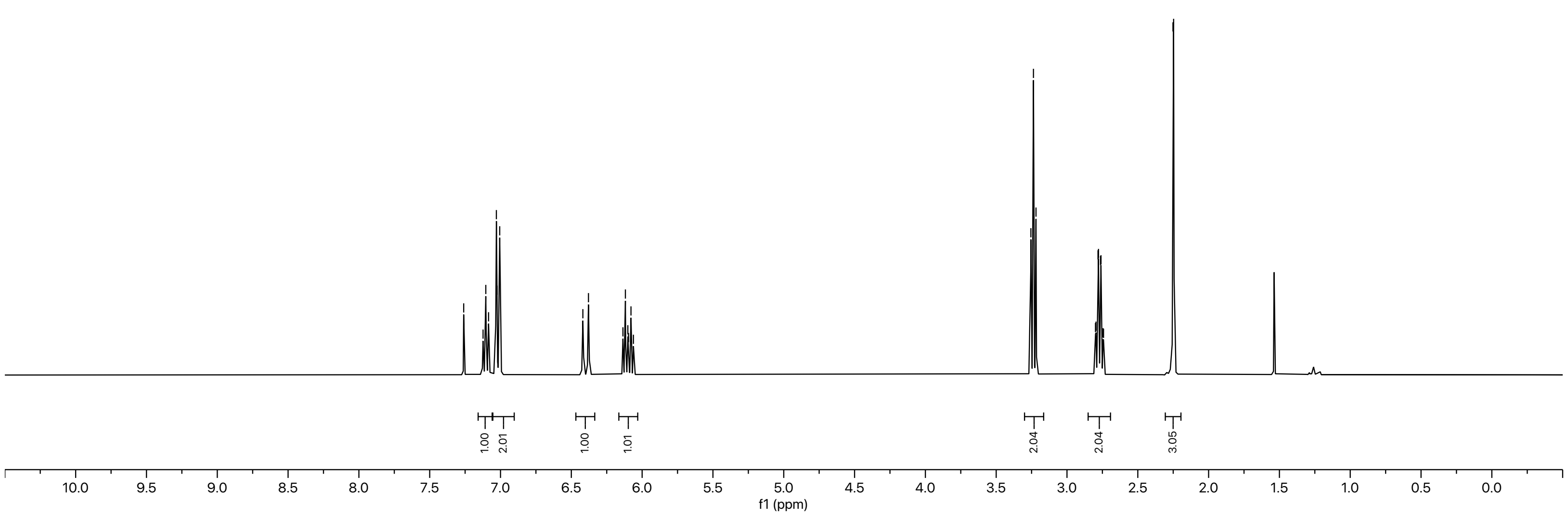

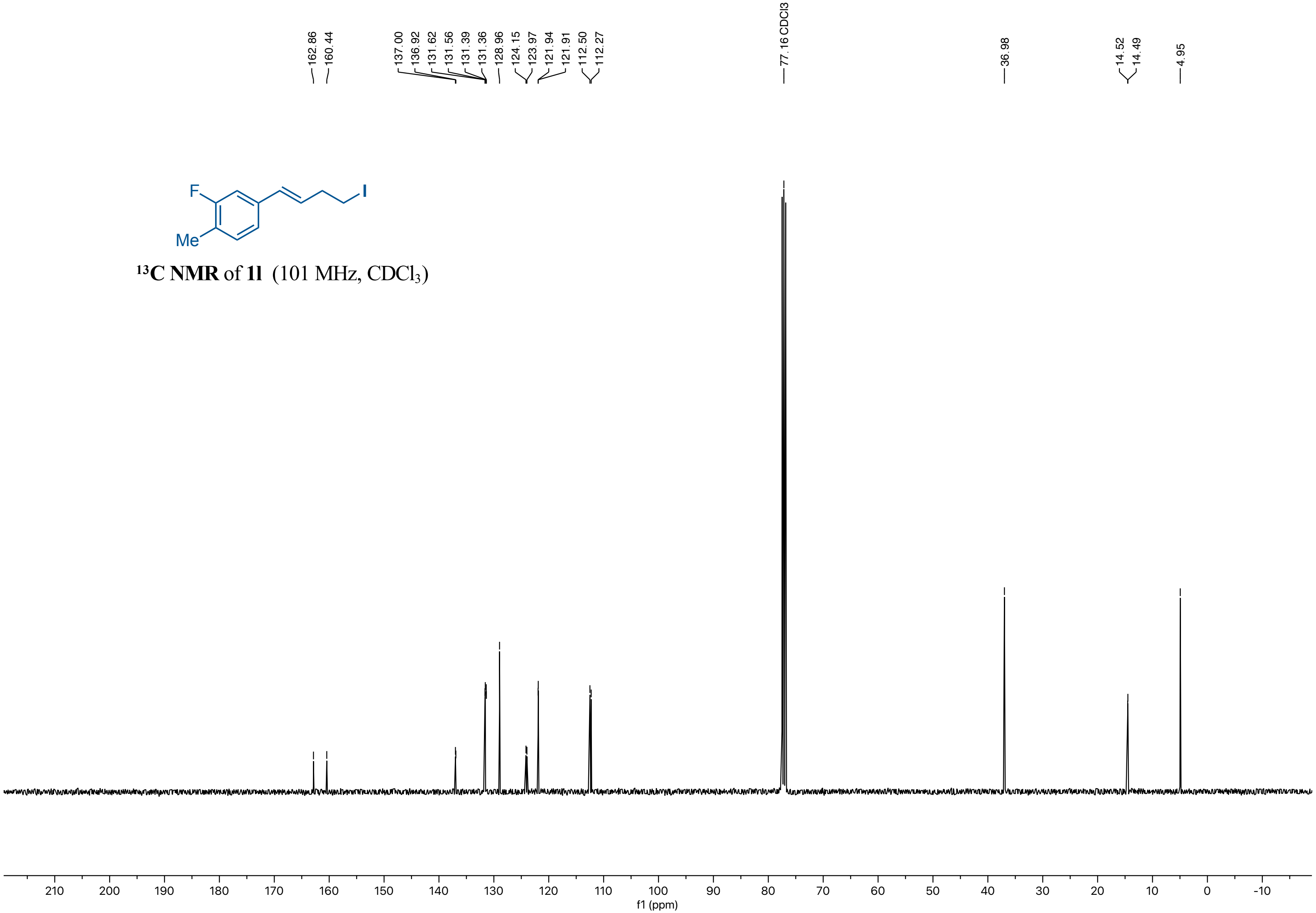

^{13}C NMR of **1l** (101 MHz, $CDCl_3$)

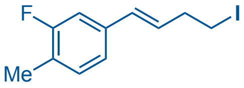

^{19}F NMR of **1l** (376 MHz, $CDCl_3$)

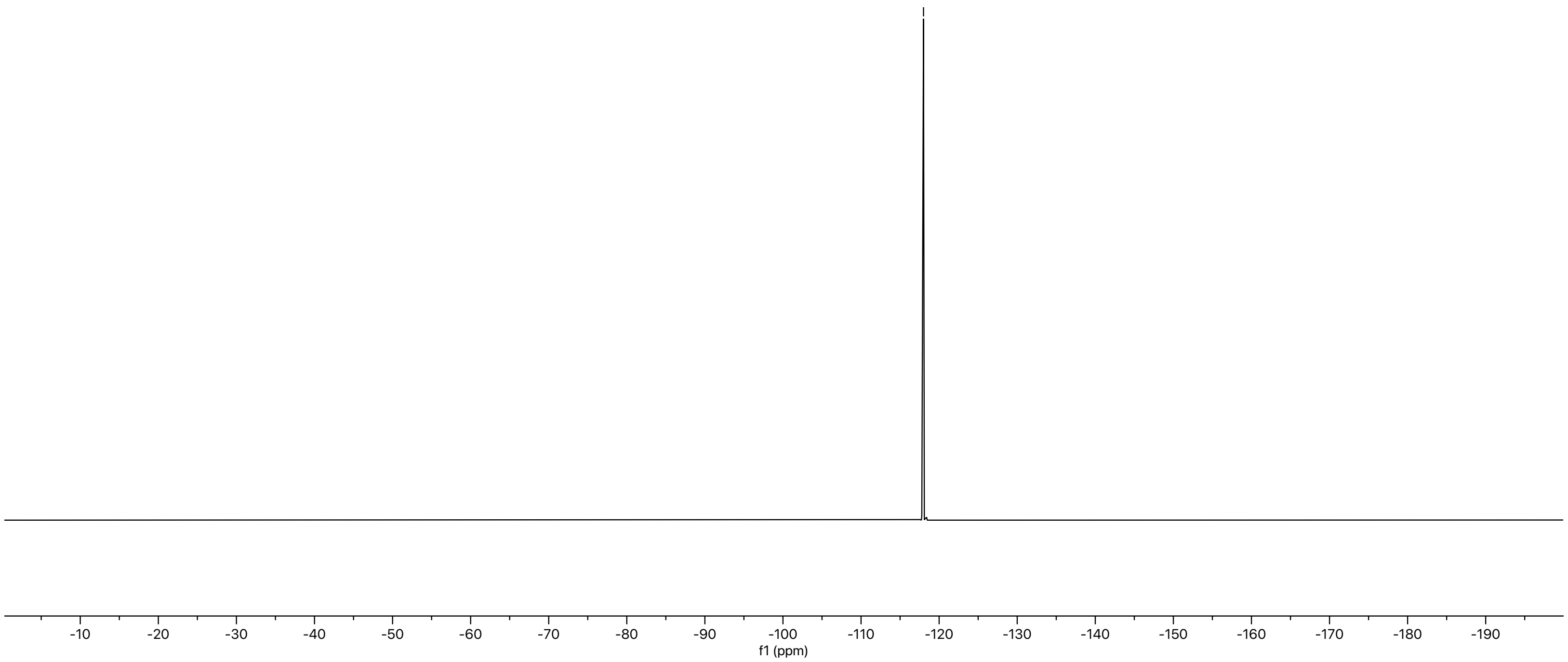

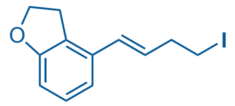

^{1}H NMR of **1m** (400 MHz, $CDCl_3$)

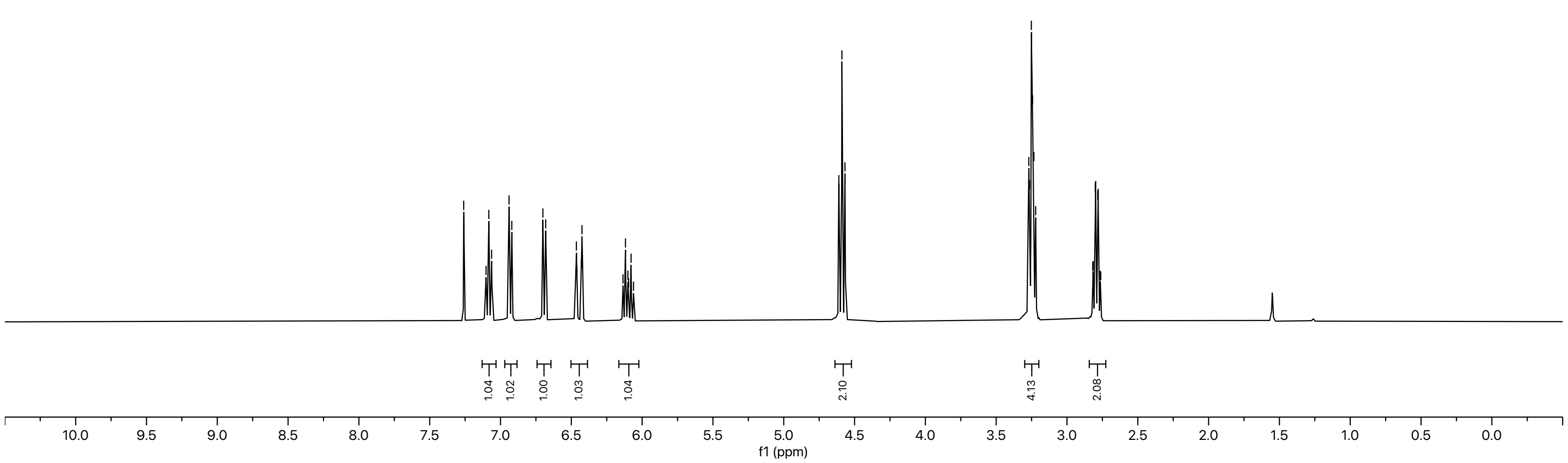

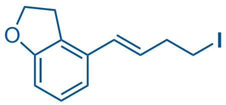

^{13}**C NMR** of **1m** (101 MHz, $CDCl_3$)

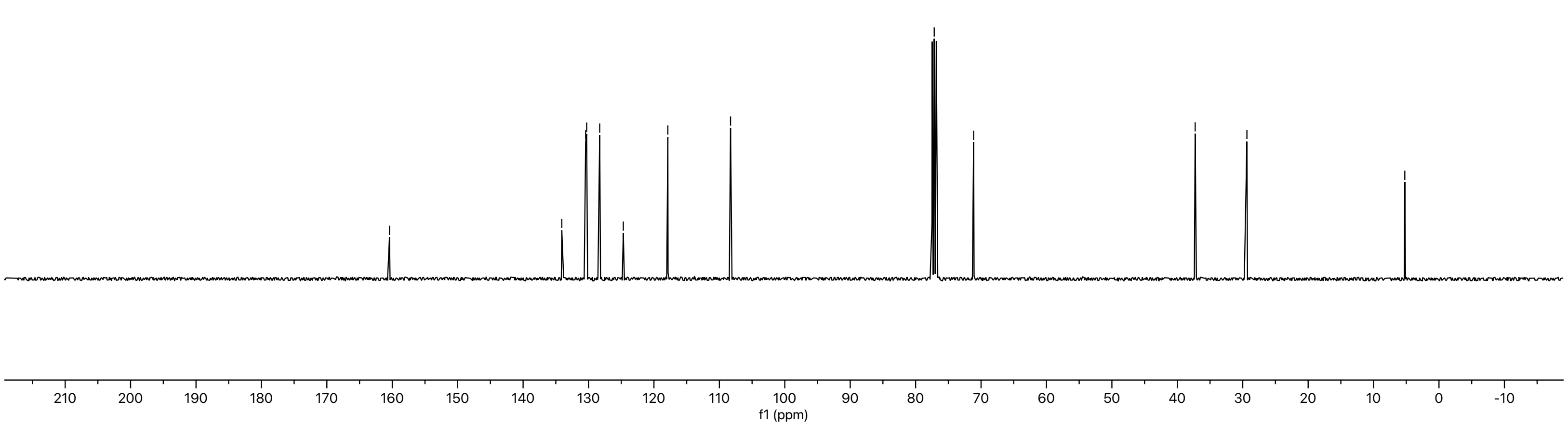

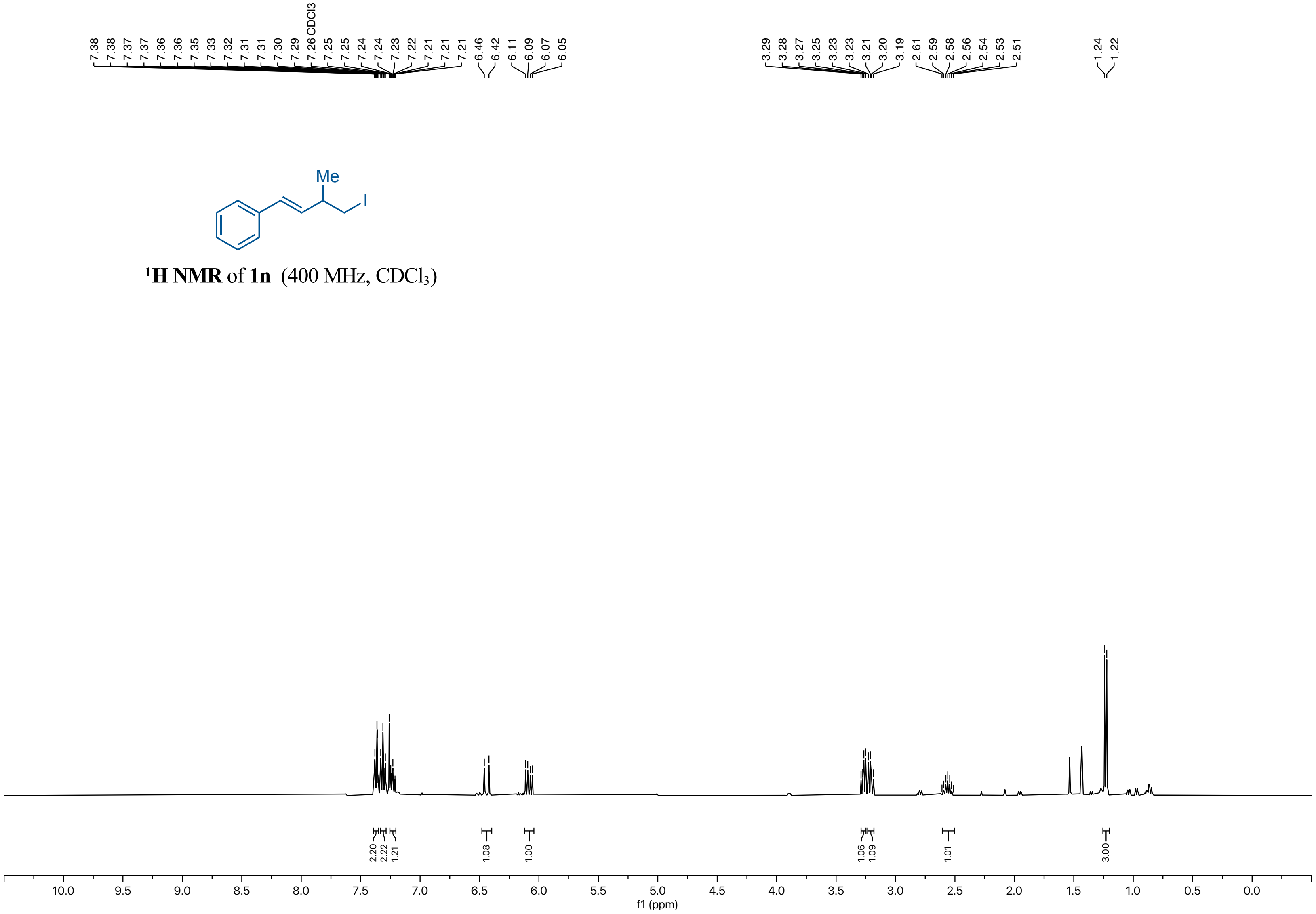

¹H NMR of **1n** (400 MHz, $CDCl_3$)

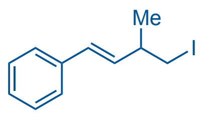

^{13}C NMR of **1n** (101 MHz, $CDCl_3$)

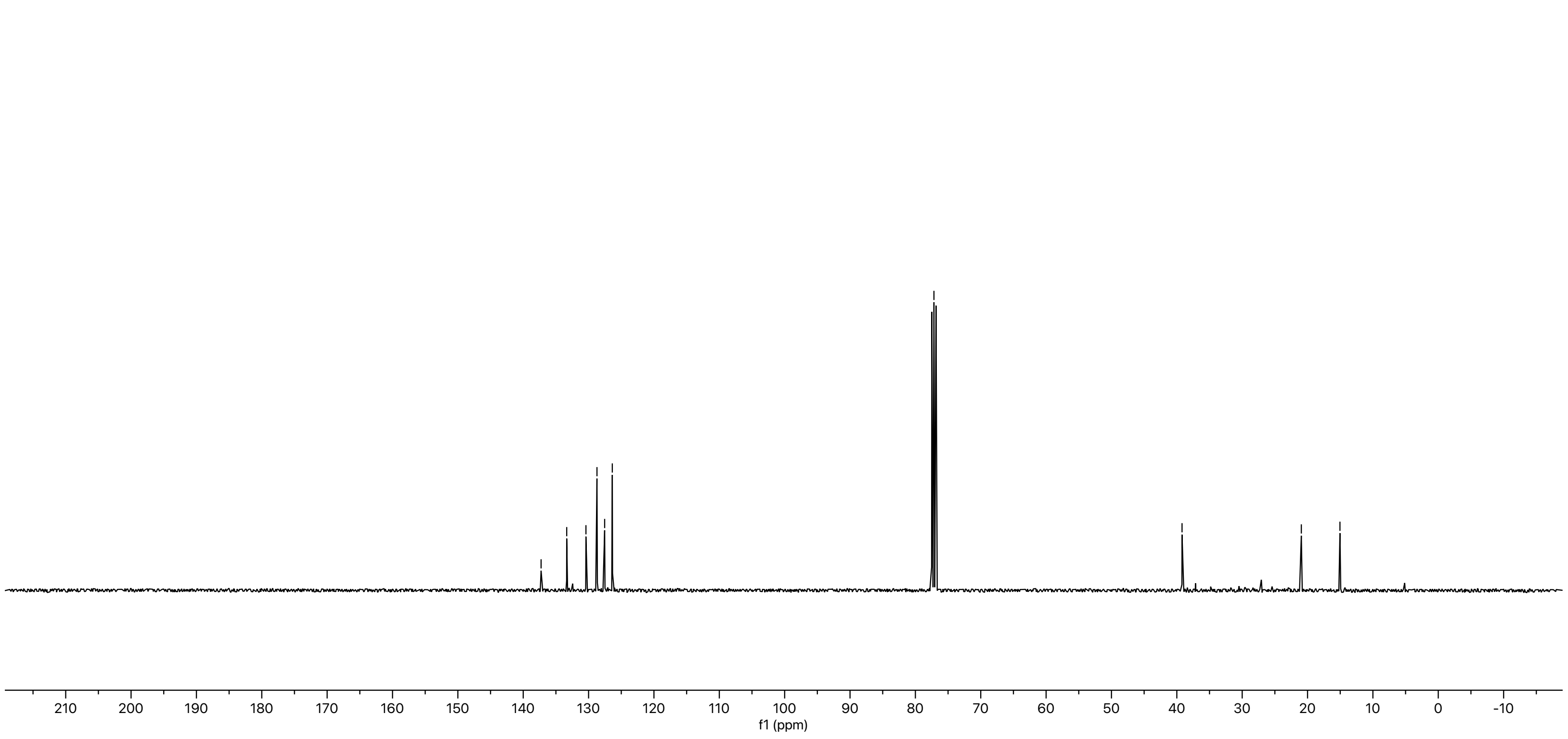

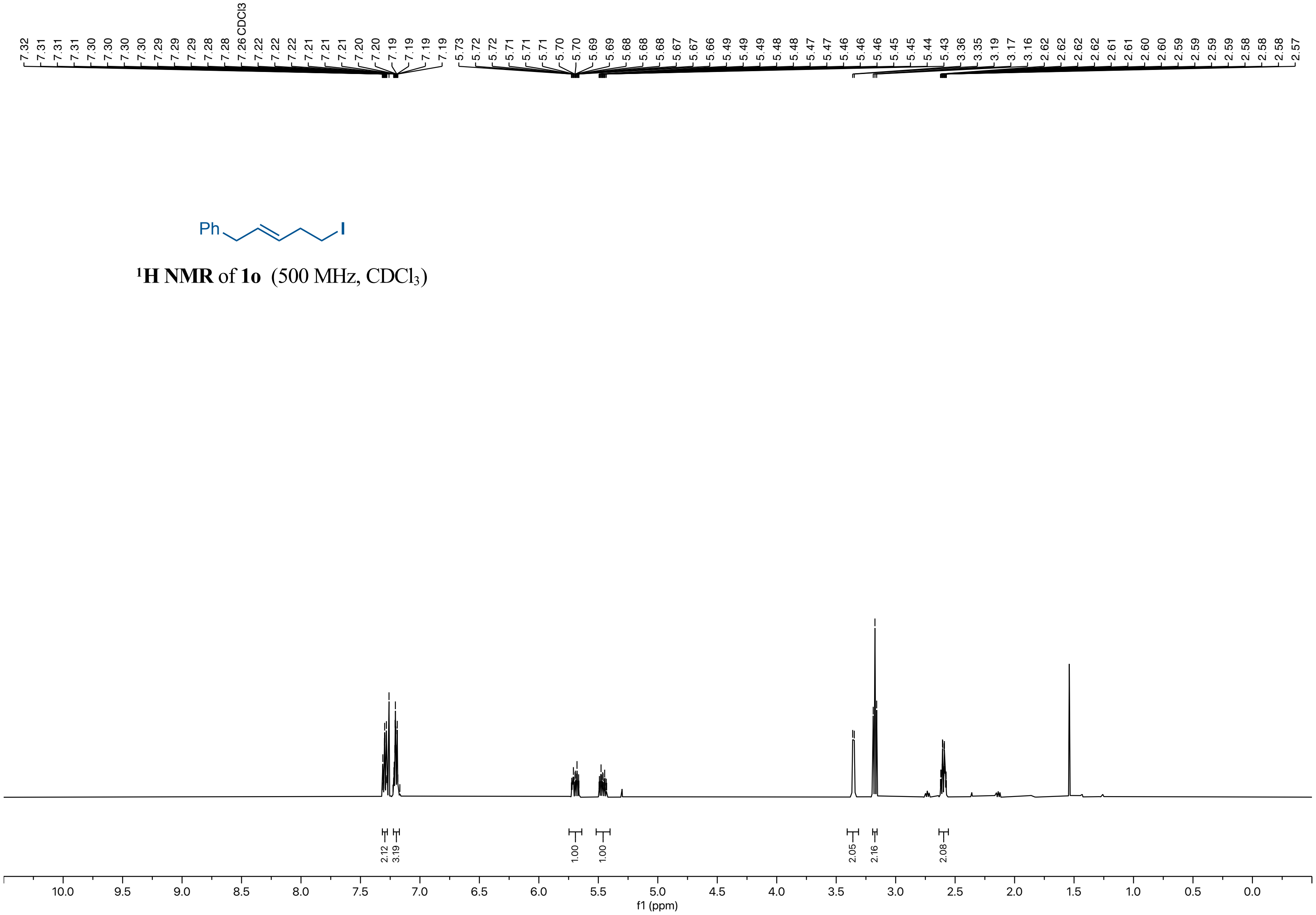

^{1}**H NMR** of **1o** (500 MHz, $CDCl_3$)

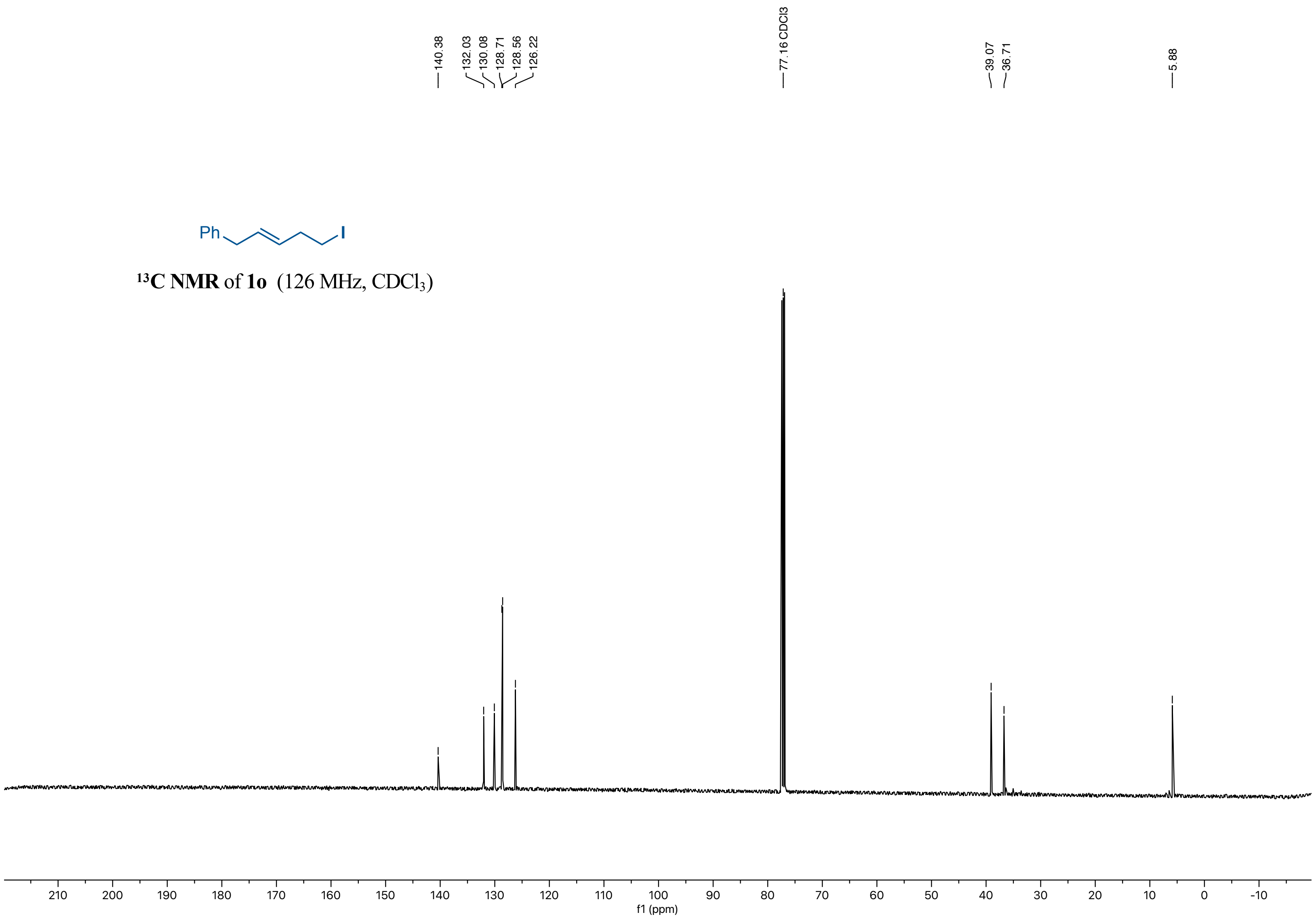

^{13}C NMR of **1o** (126 MHz, $CDCl_3$)

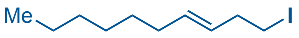

^{1}H NMR of **1p** (500 MHz, $CDCl_3$)

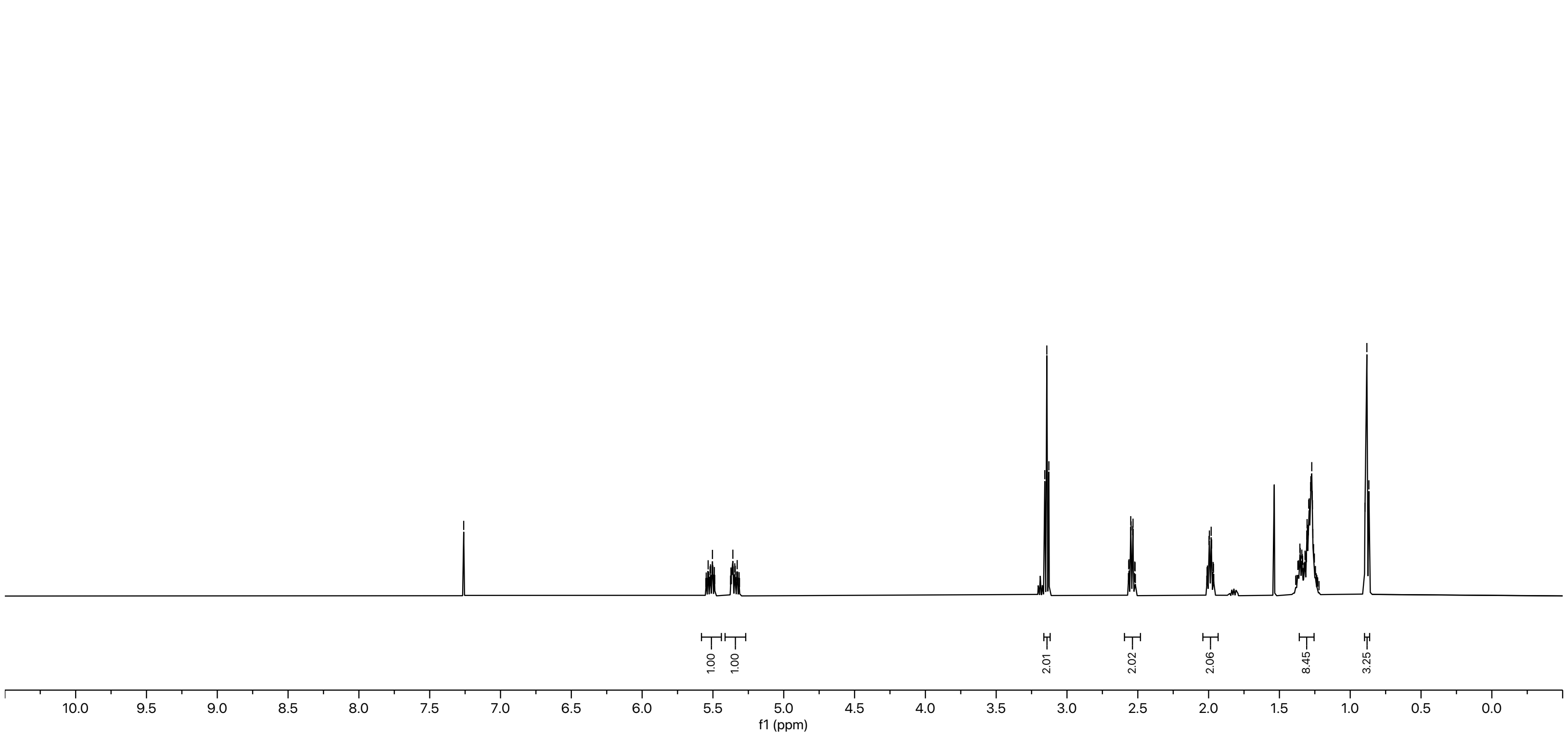

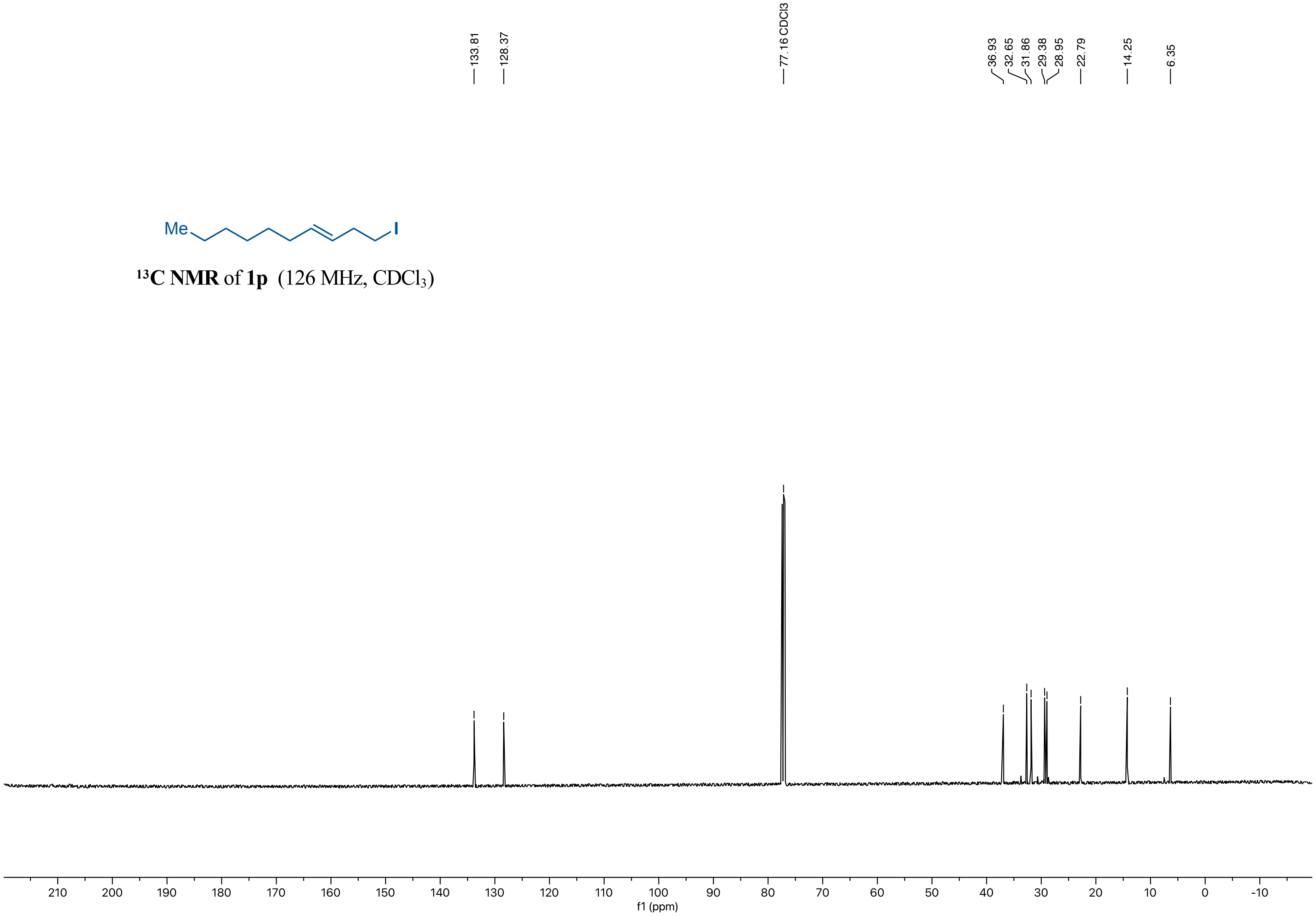

^{13}**C NMR** of **1p** (126 MHz, $CDCl_3$)

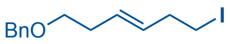

^{1}**H NMR** of **1q** (500 MHz, $CDCl_3$)

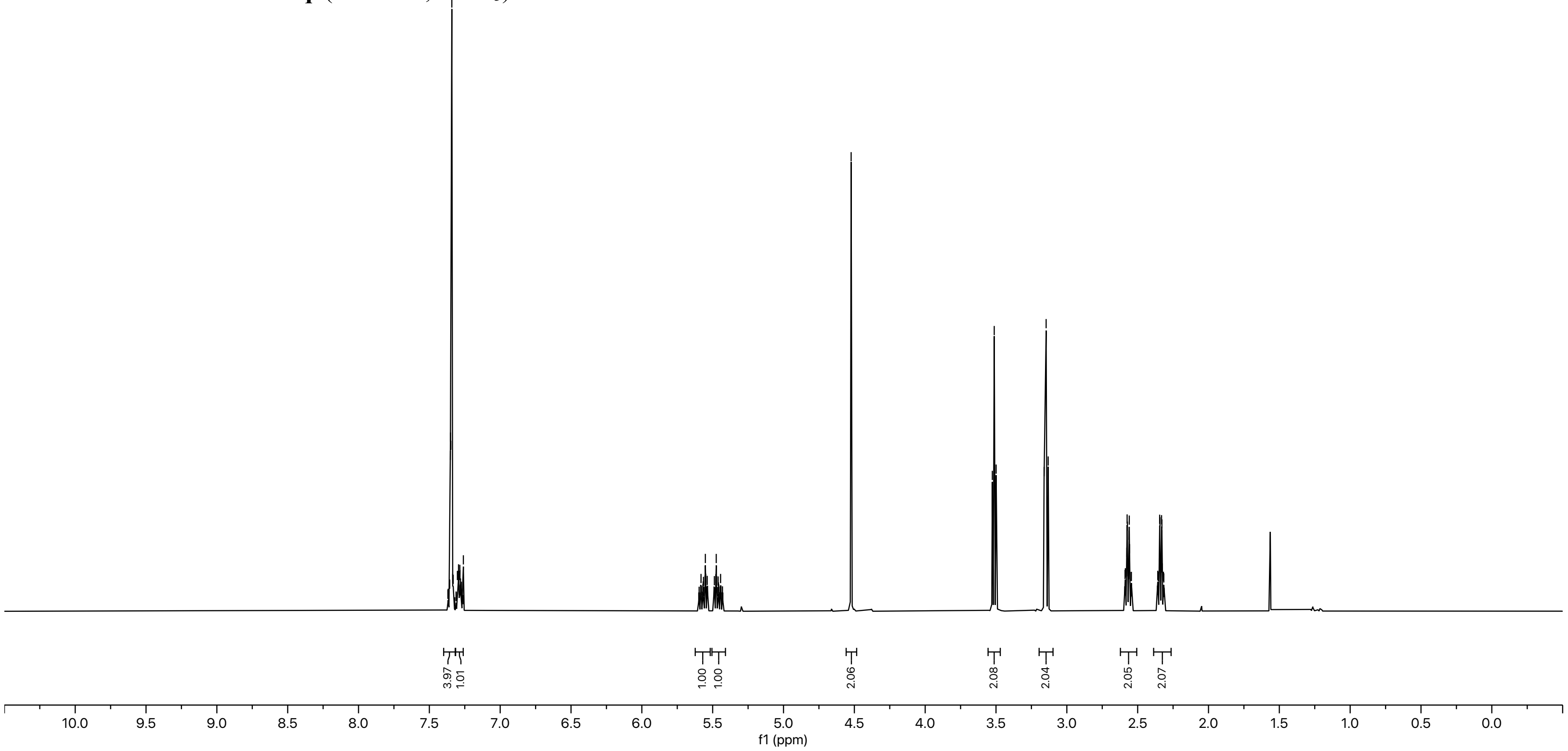

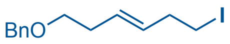

^{13}C NMR of **1q** (126 MHz, $CDCl_3$)

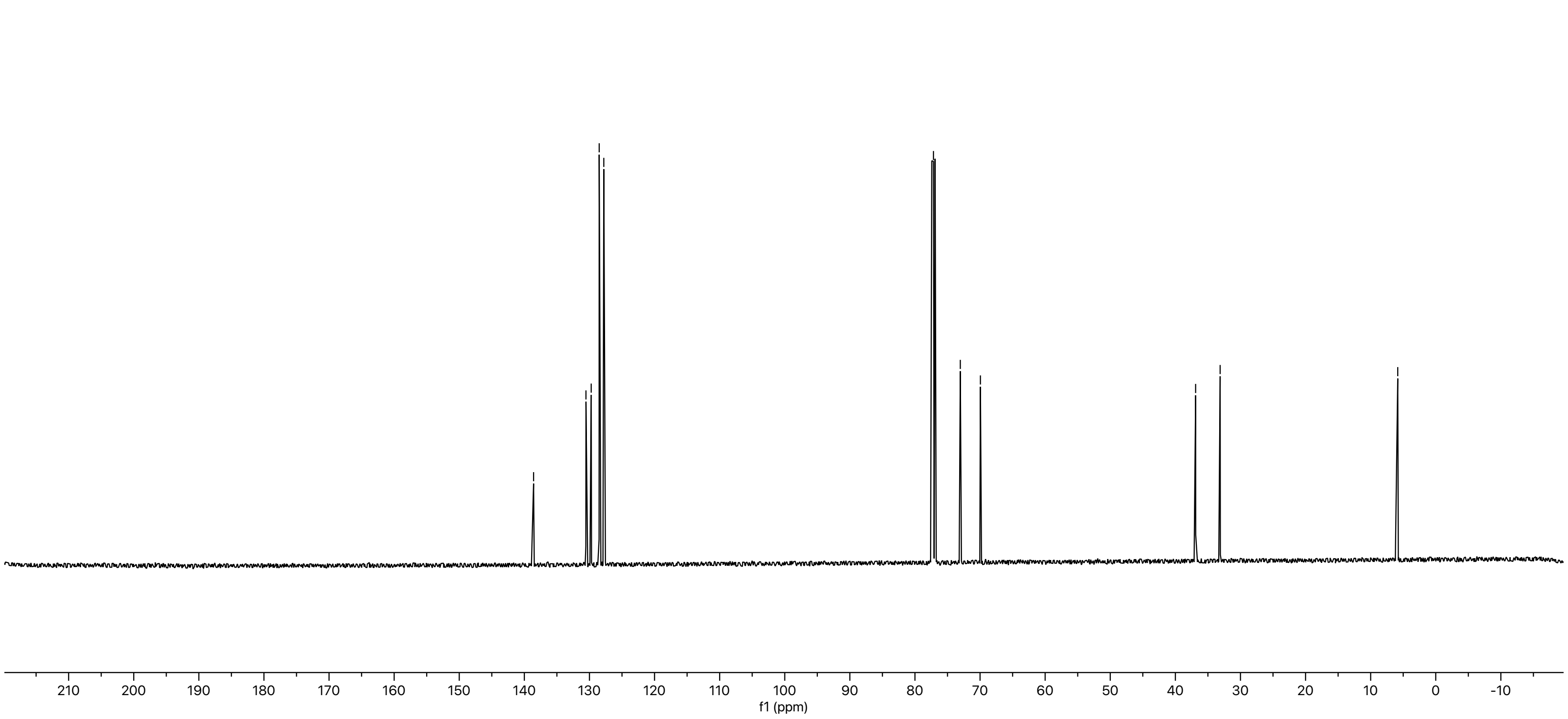

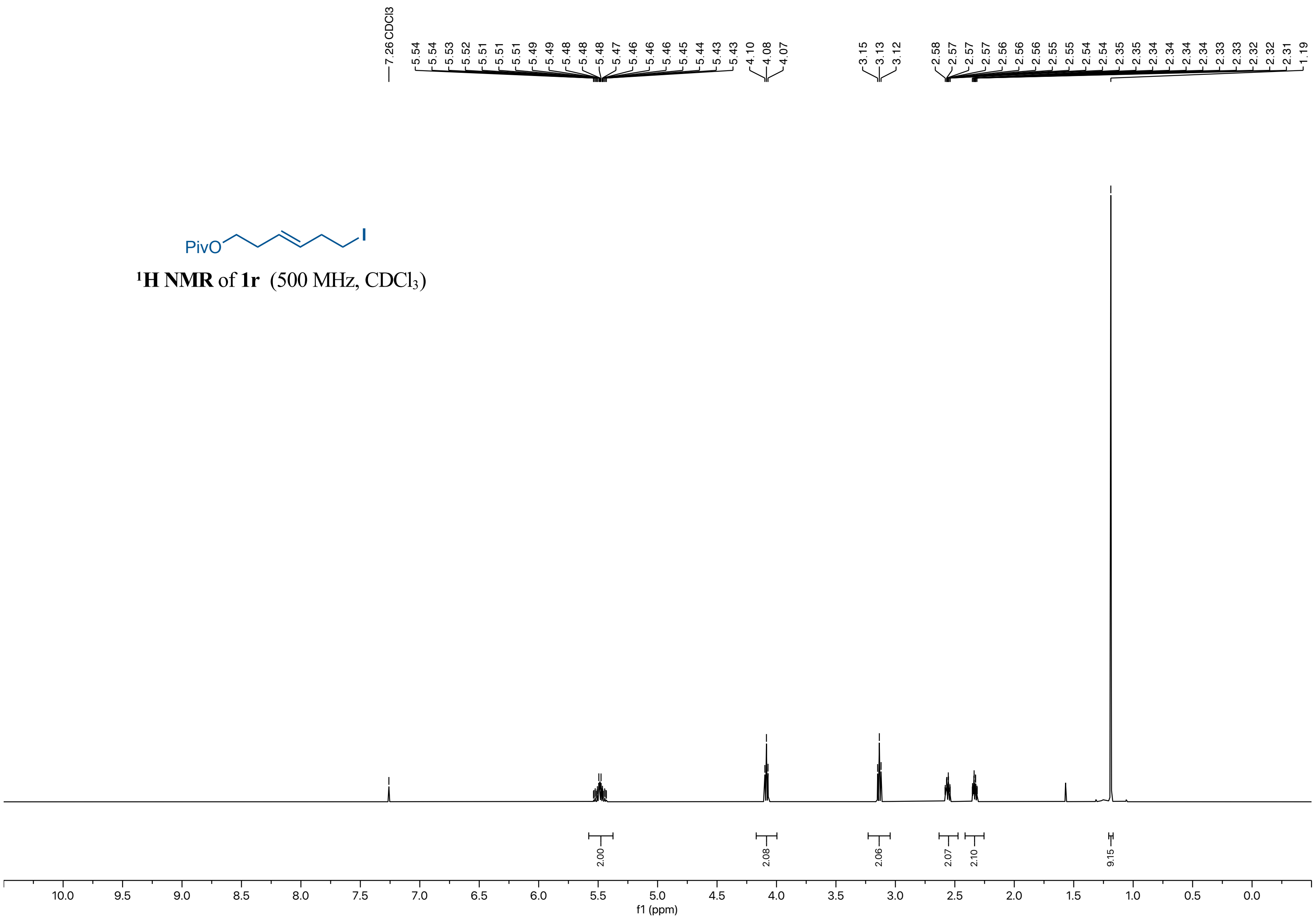

¹H NMR of **1r** (500 MHz, $CDCl_3$)

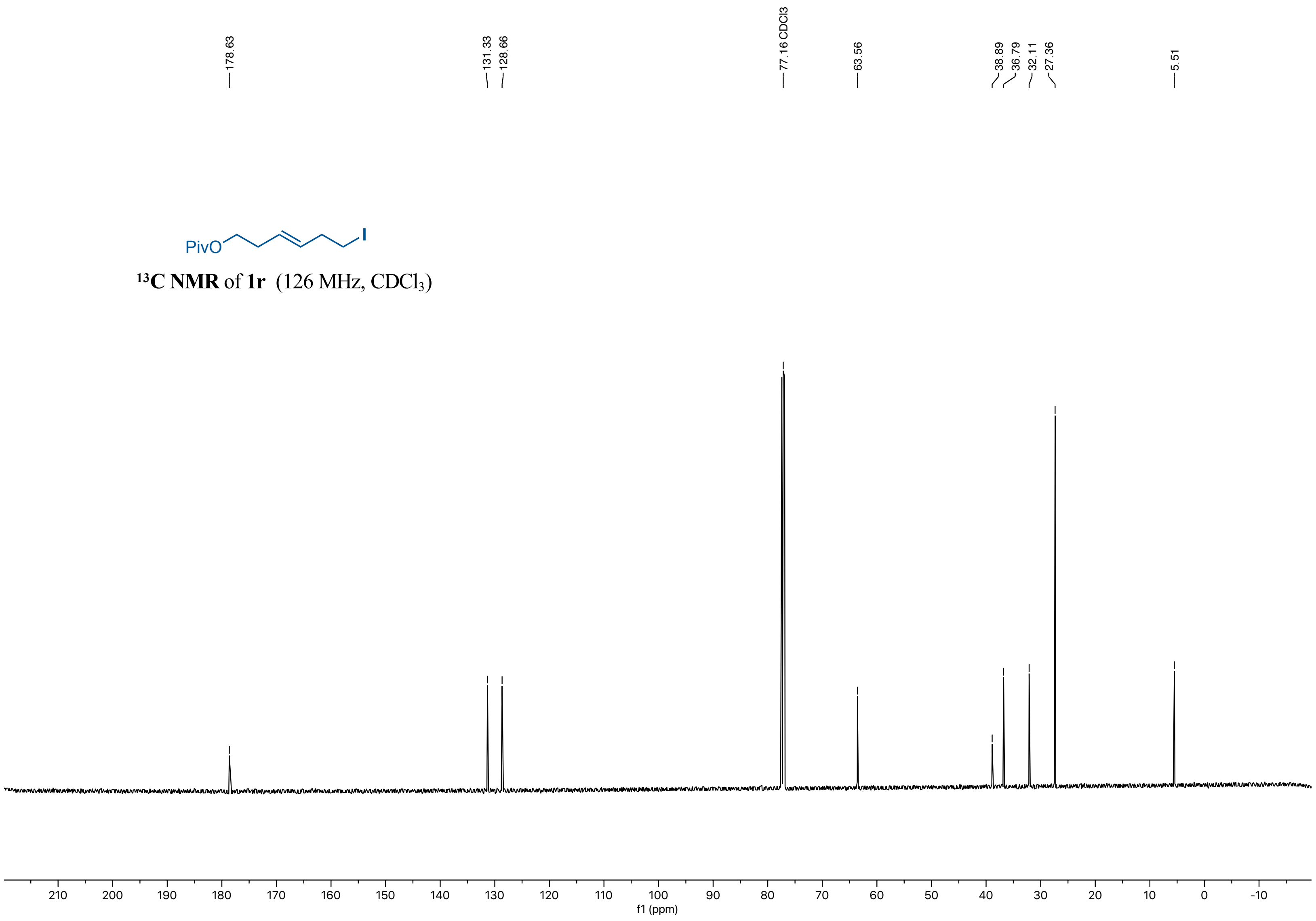

^{13}C NMR of **1r** (126 MHz, $CDCl_3$)

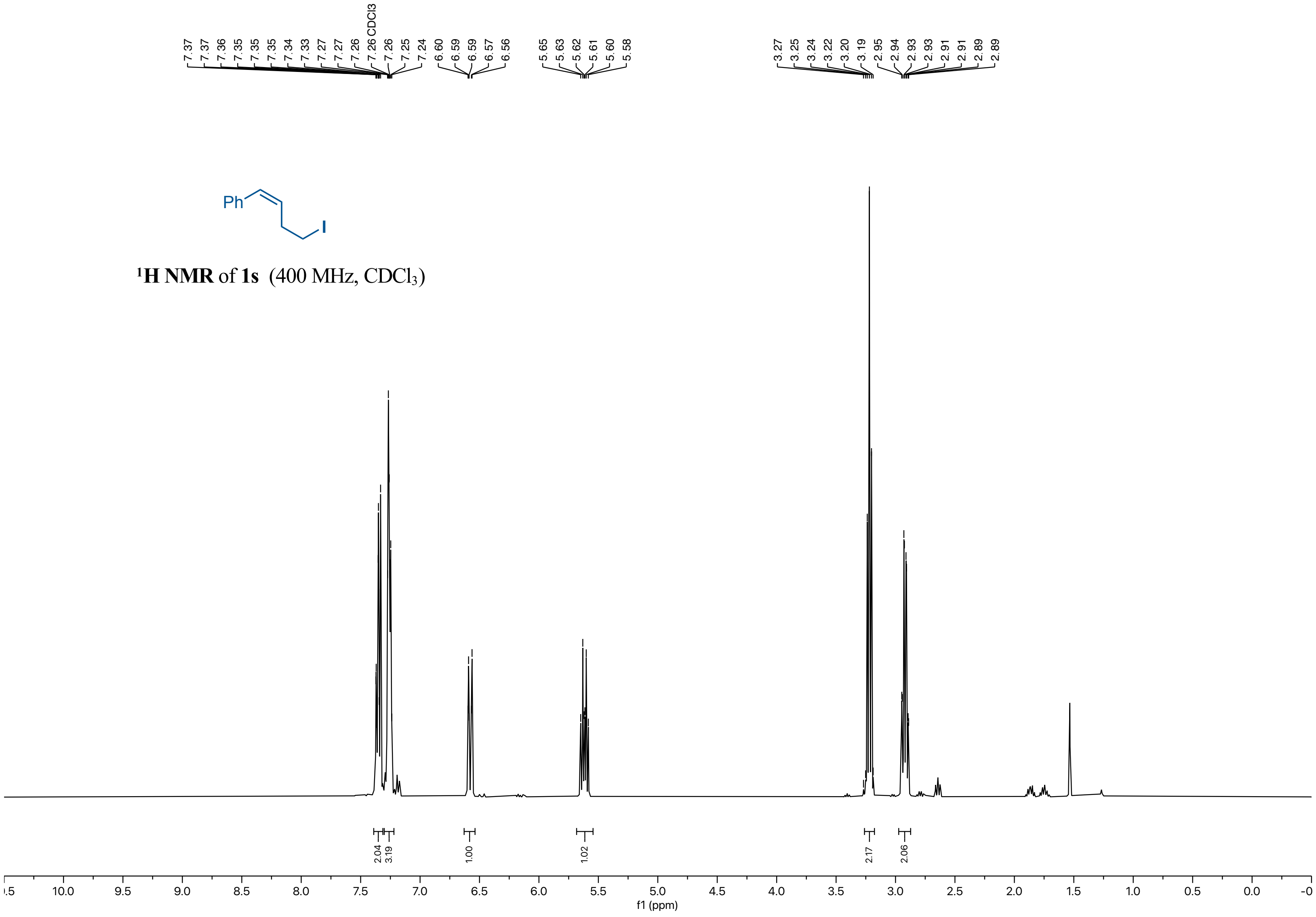

^{1}H NMR of **1s** (400 MHz, $CDCl_3$)

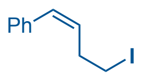

^{13}C NMR of **1s** (101 MHz, $CDCl_3$)

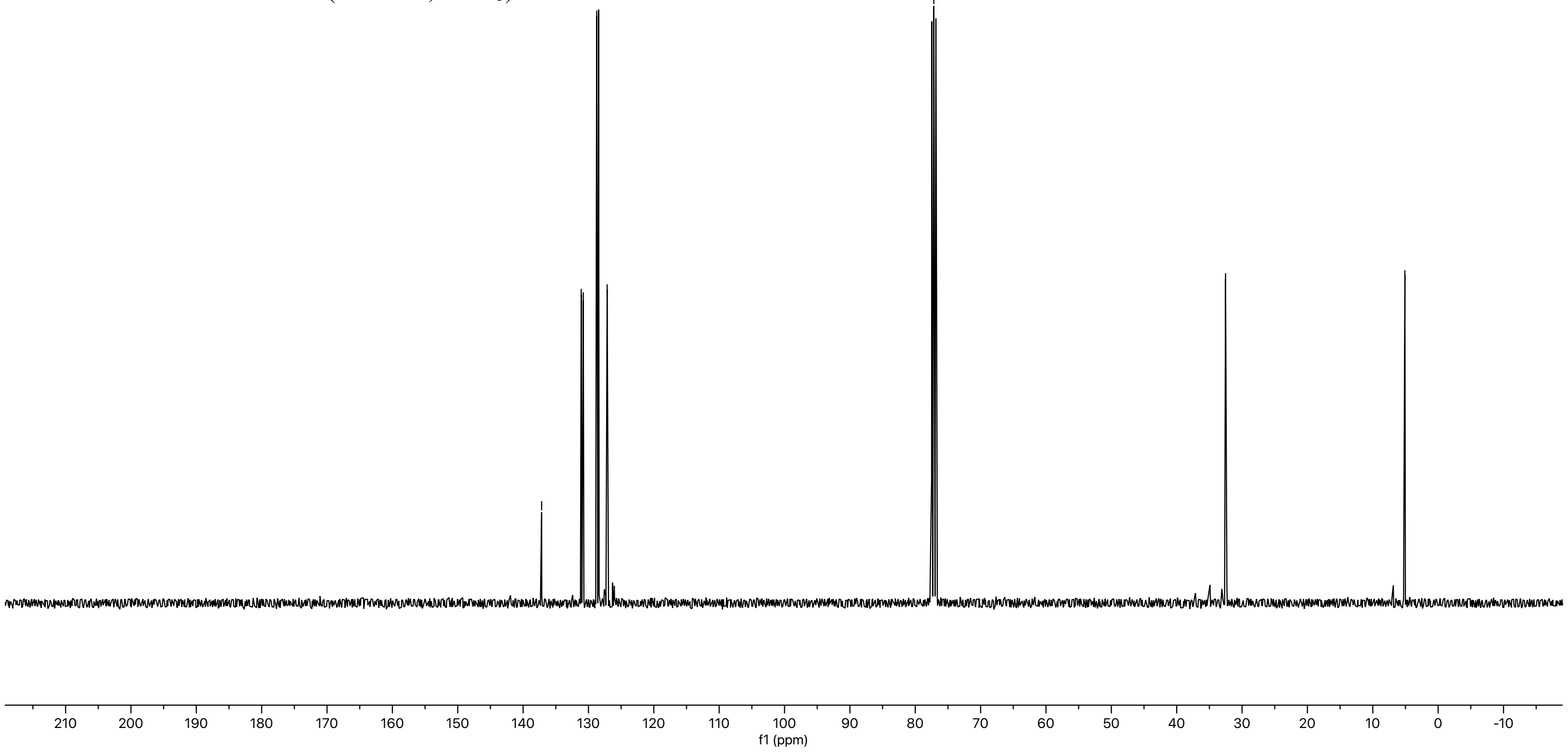

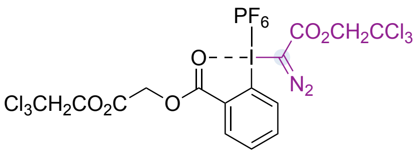

^{1}H NMR of **2c** (400 MHz, $(CD_3)_2CO$)

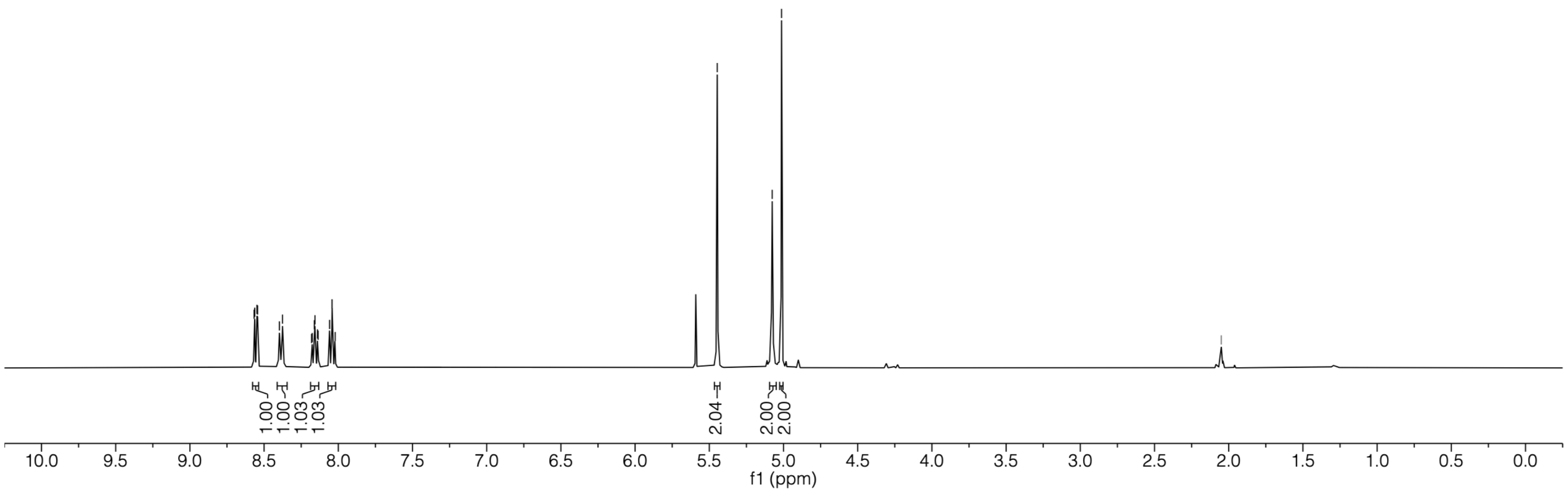

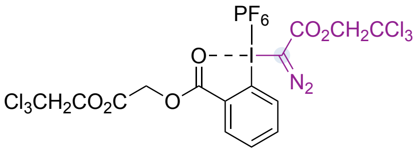

^{13}C NMR of **2c** (126 MHz, $CDCl_3$)

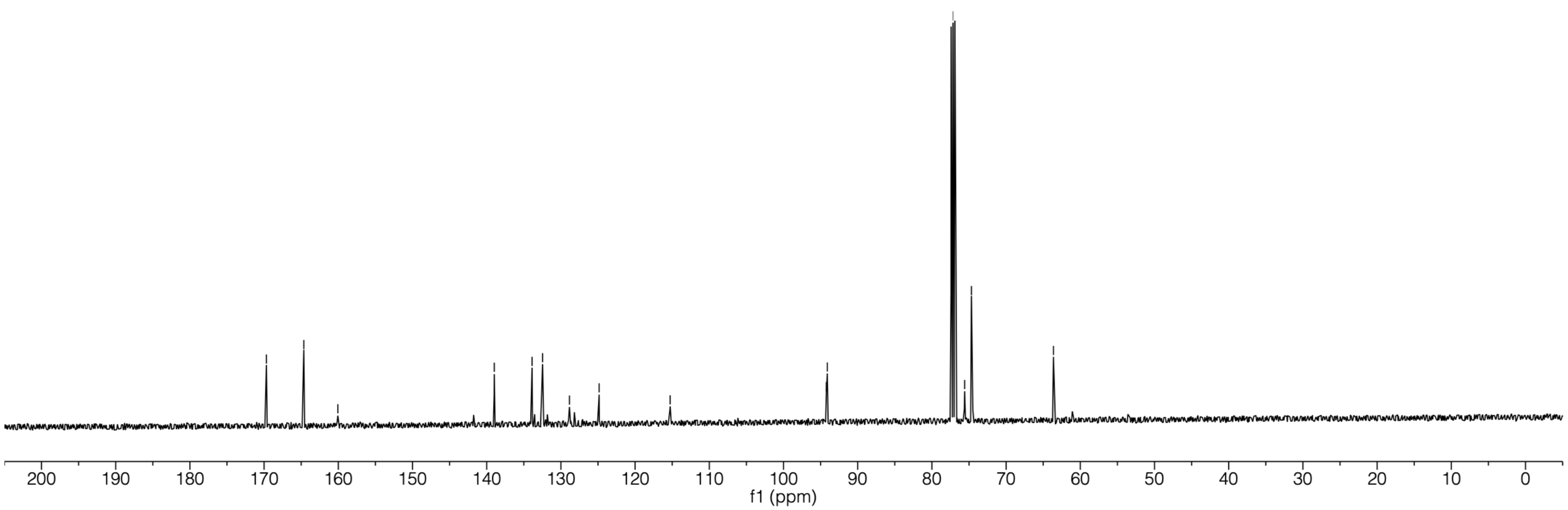

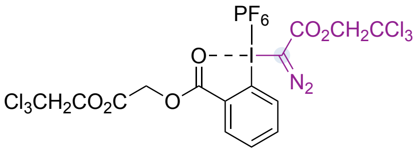

^{19}F NMR of **2c** (376 MHz, $(CD_3)_2CO$)

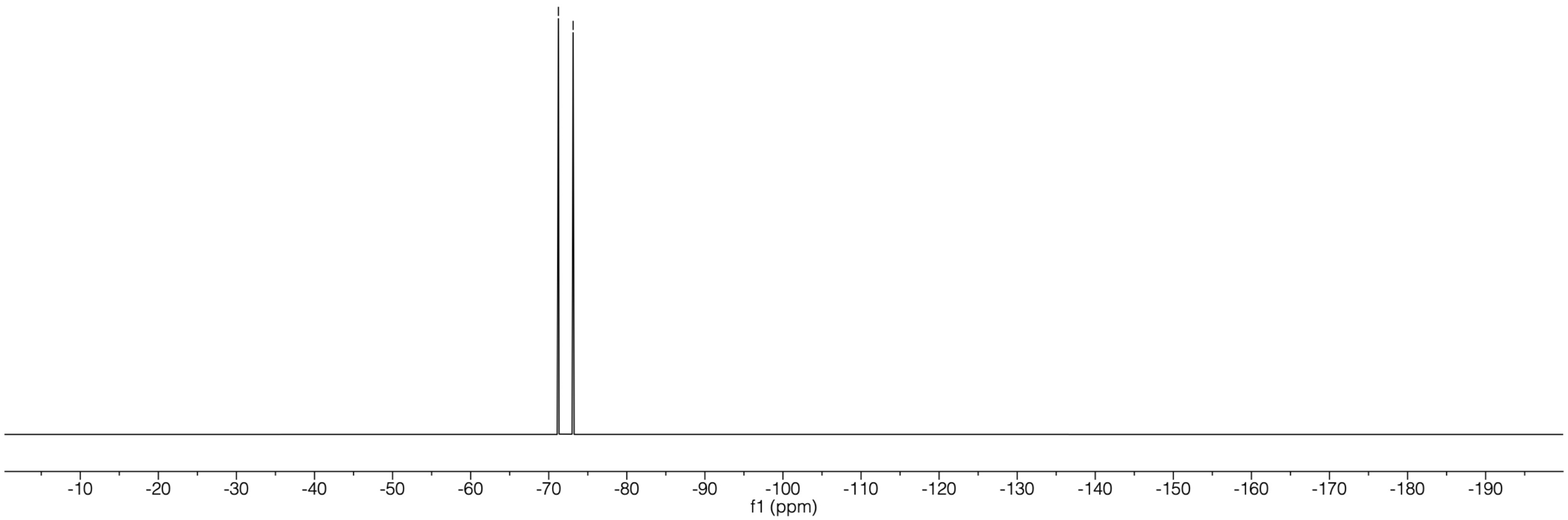

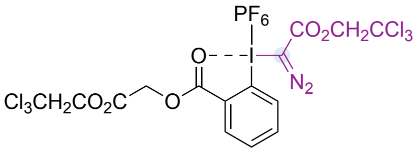

^{31}**P NMR** of **2c** (162 MHz, $(CD_3)_2CO$)

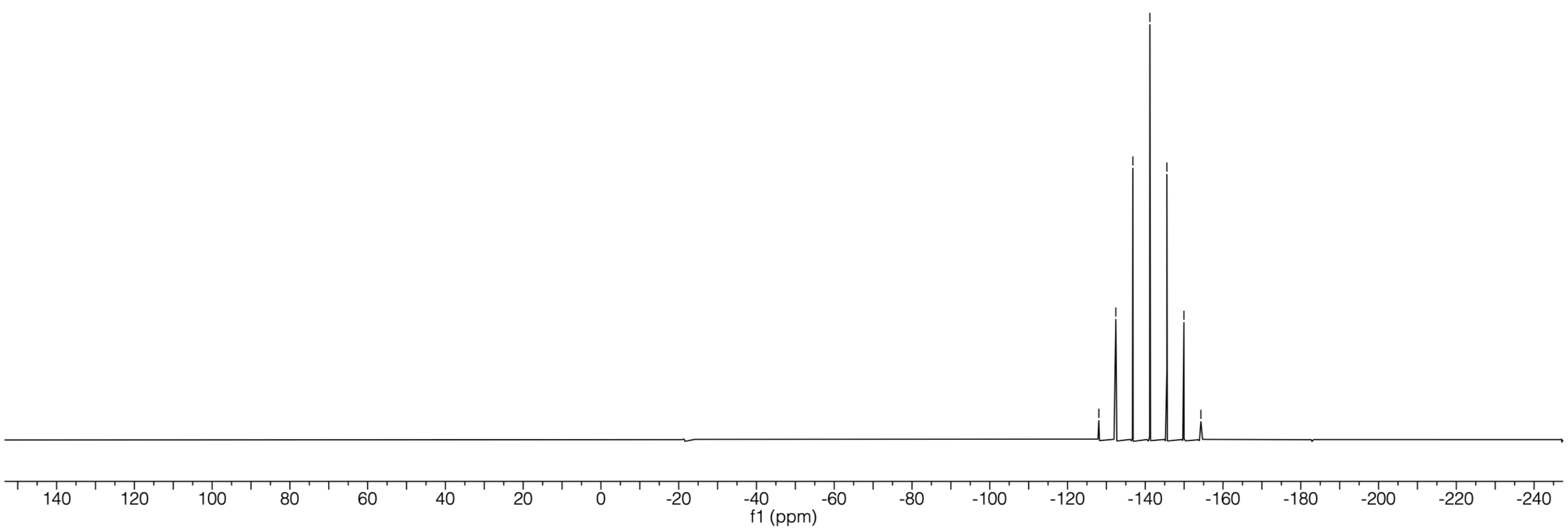

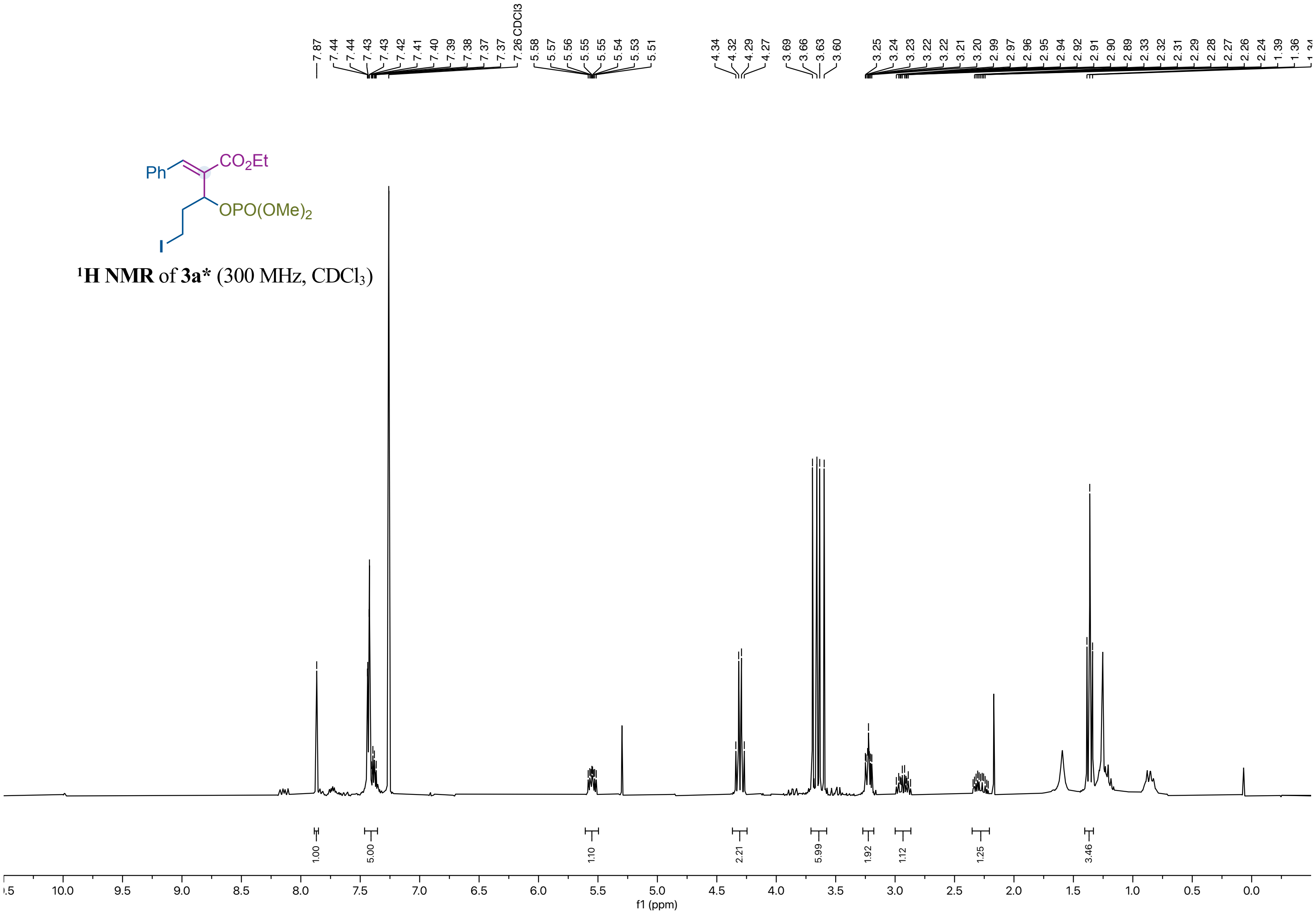

¹H NMR of **3a*** (300 MHz, $CDCl_3$)

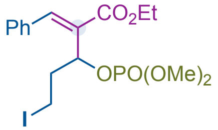

^{13}**C NMR** of **3a*** (126 MHz, $CDCl_3$)

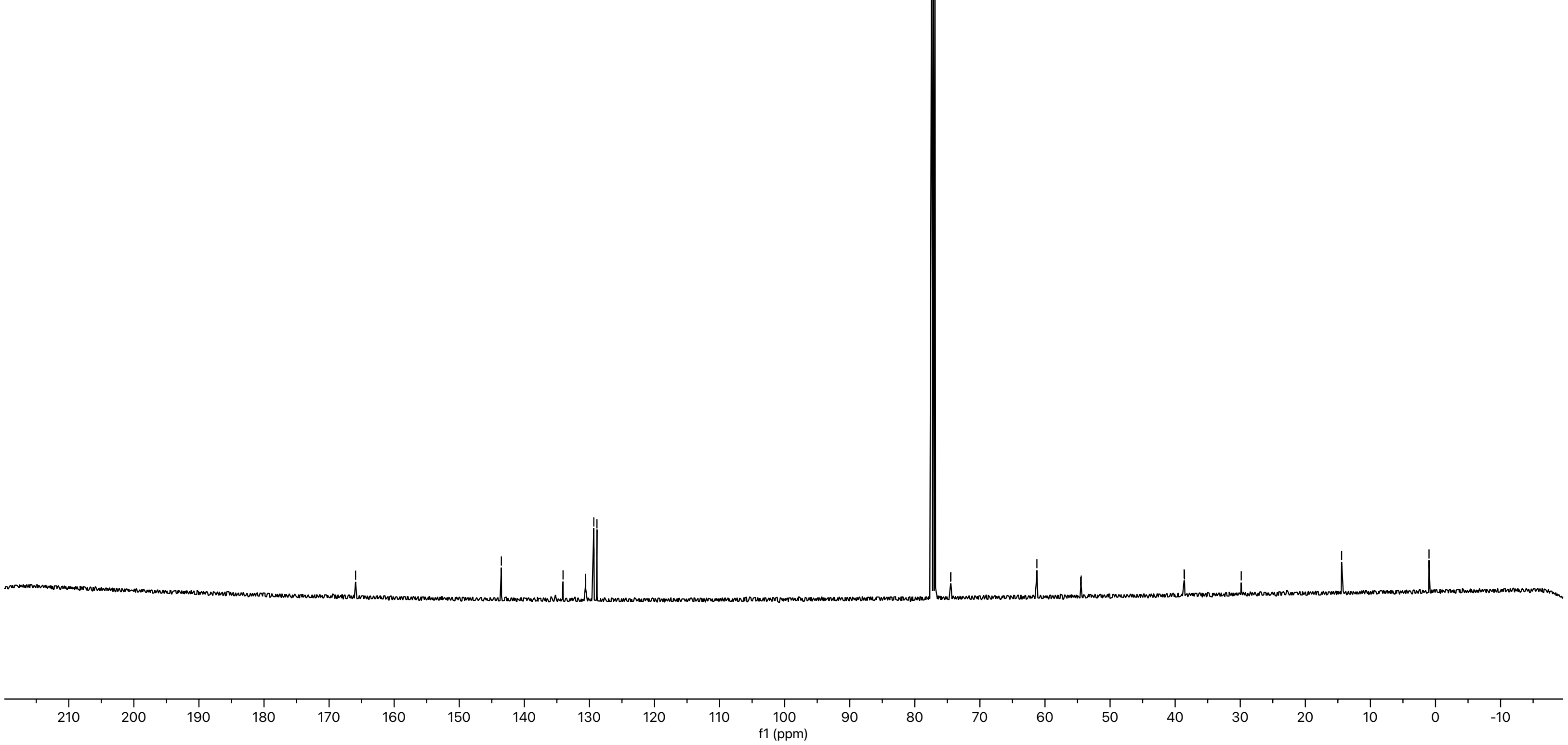

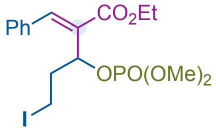

^{31}**P NMR** of **3a*** (202 MHz, $CDCl_3$)

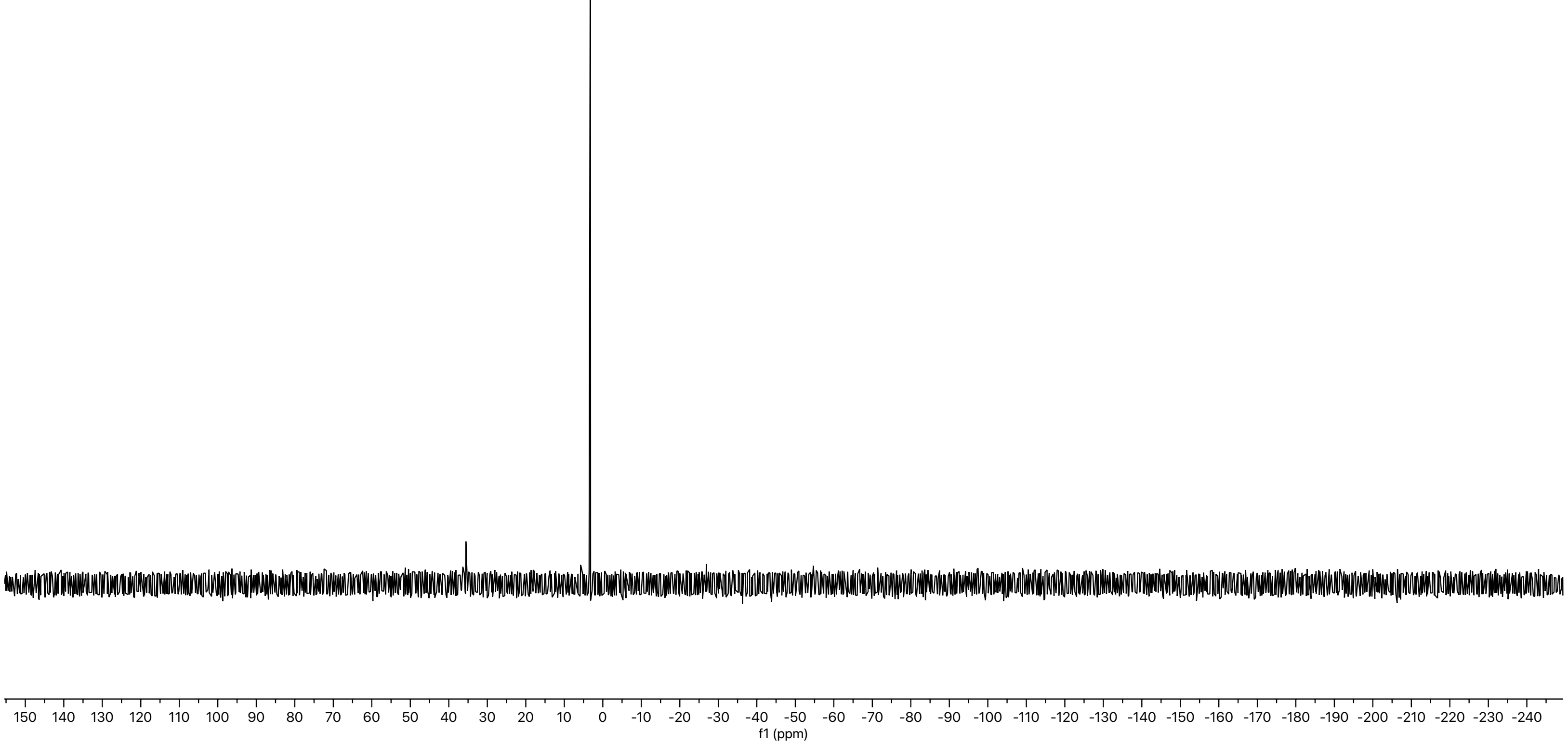

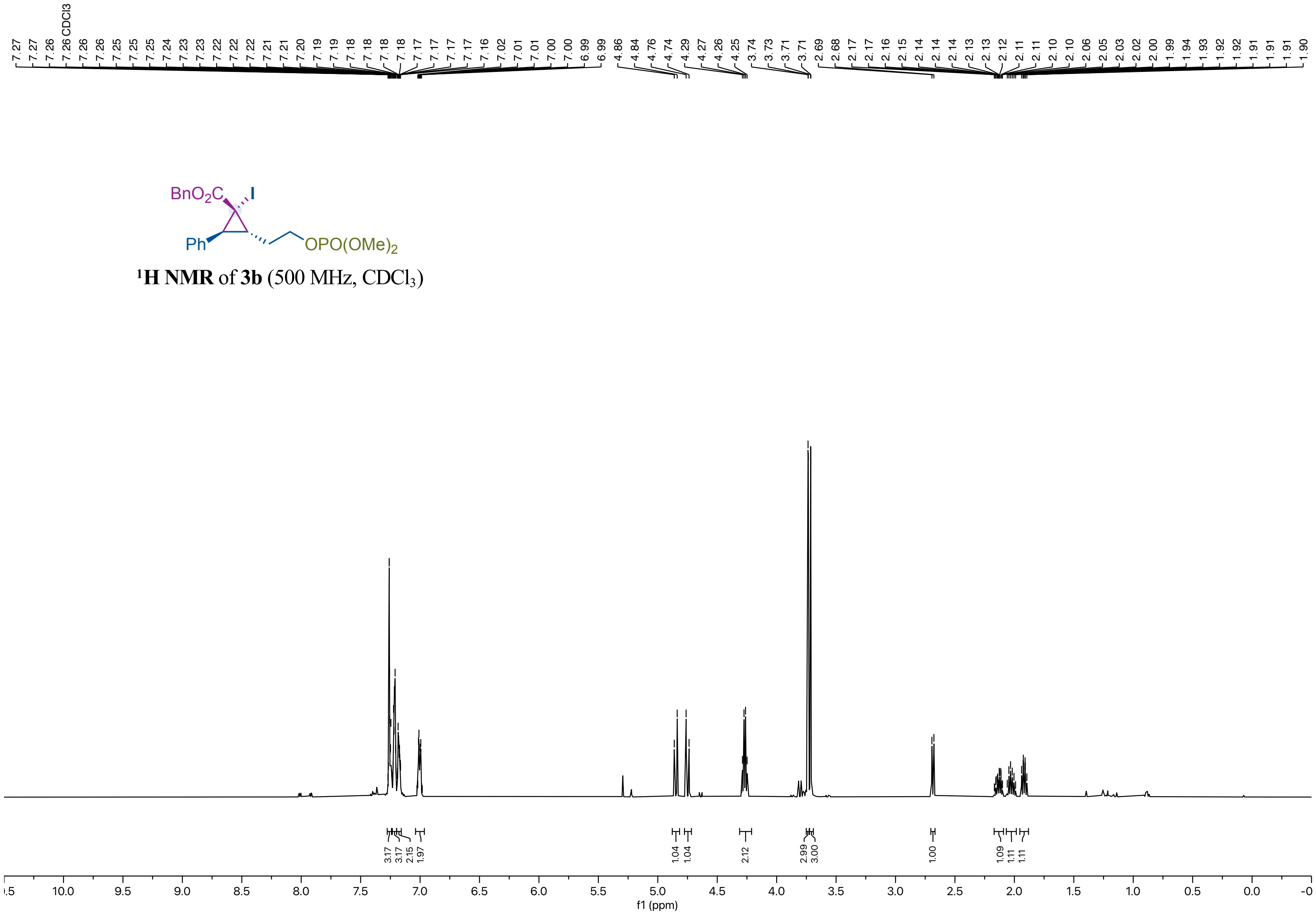

[1]**H NMR** of **3b** (500 MHz, $CDCl_3$)

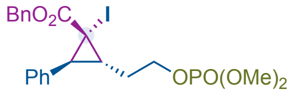

^{13}C NMR of **3b** (126 MHz, $CDCl_3$)

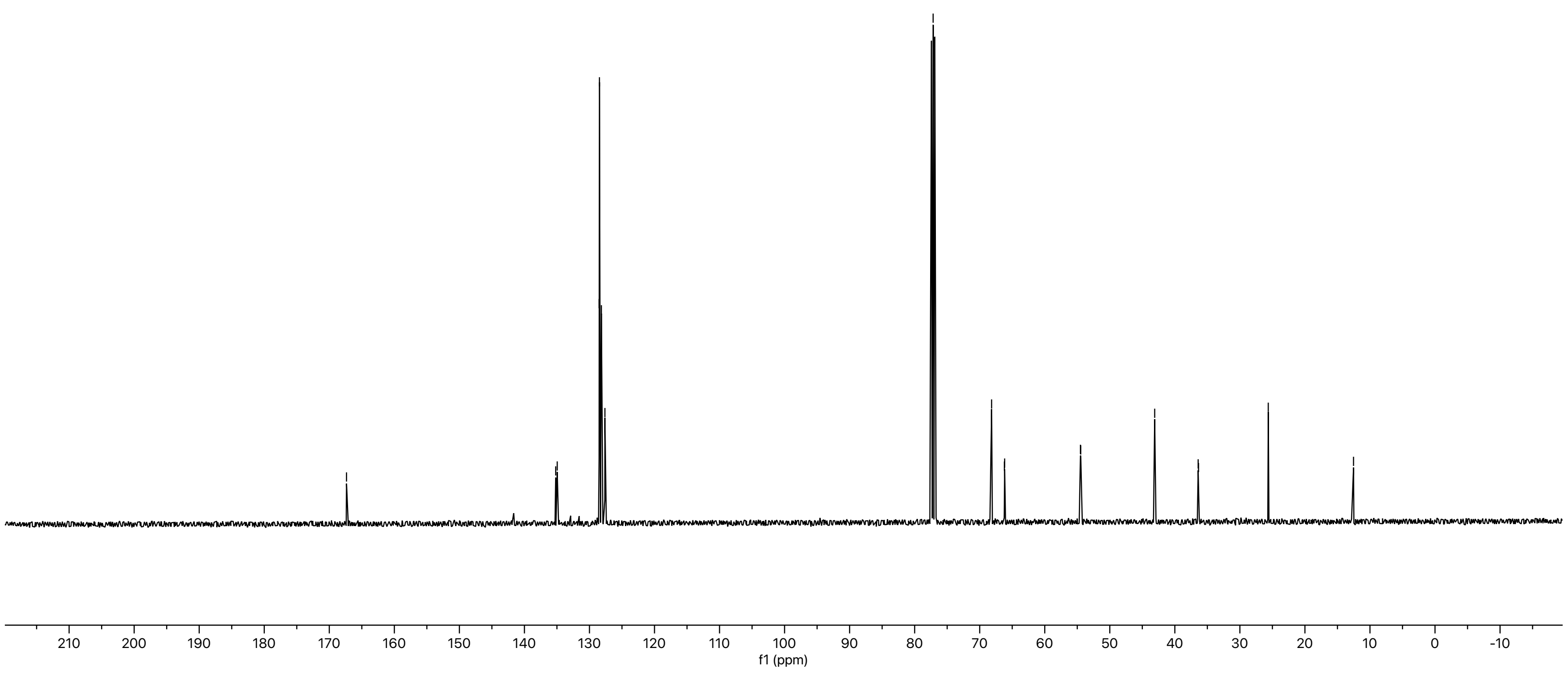

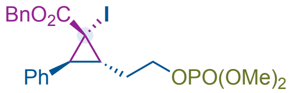

^{31}P NMR of **3b** (202 MHz, $CDCl_3$)

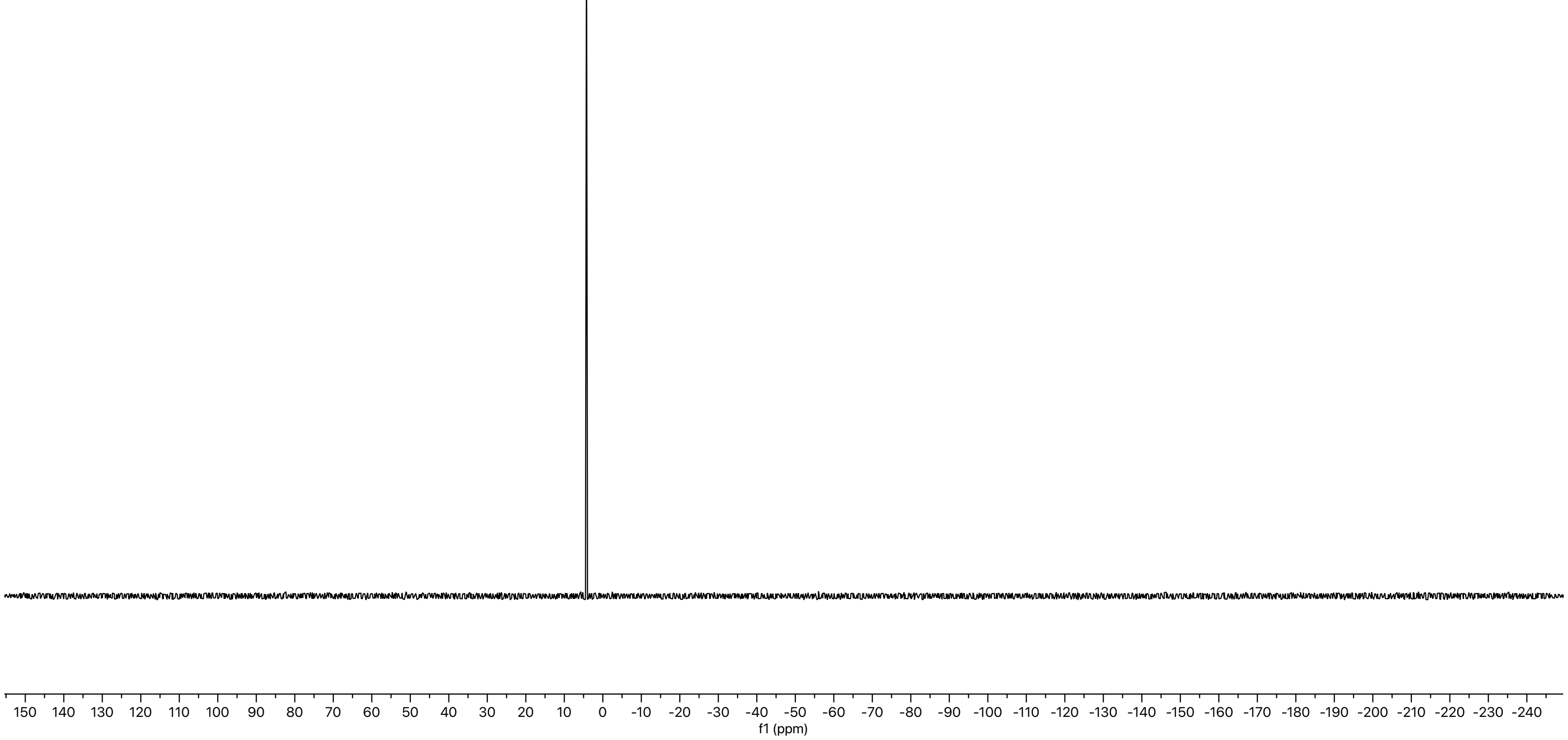

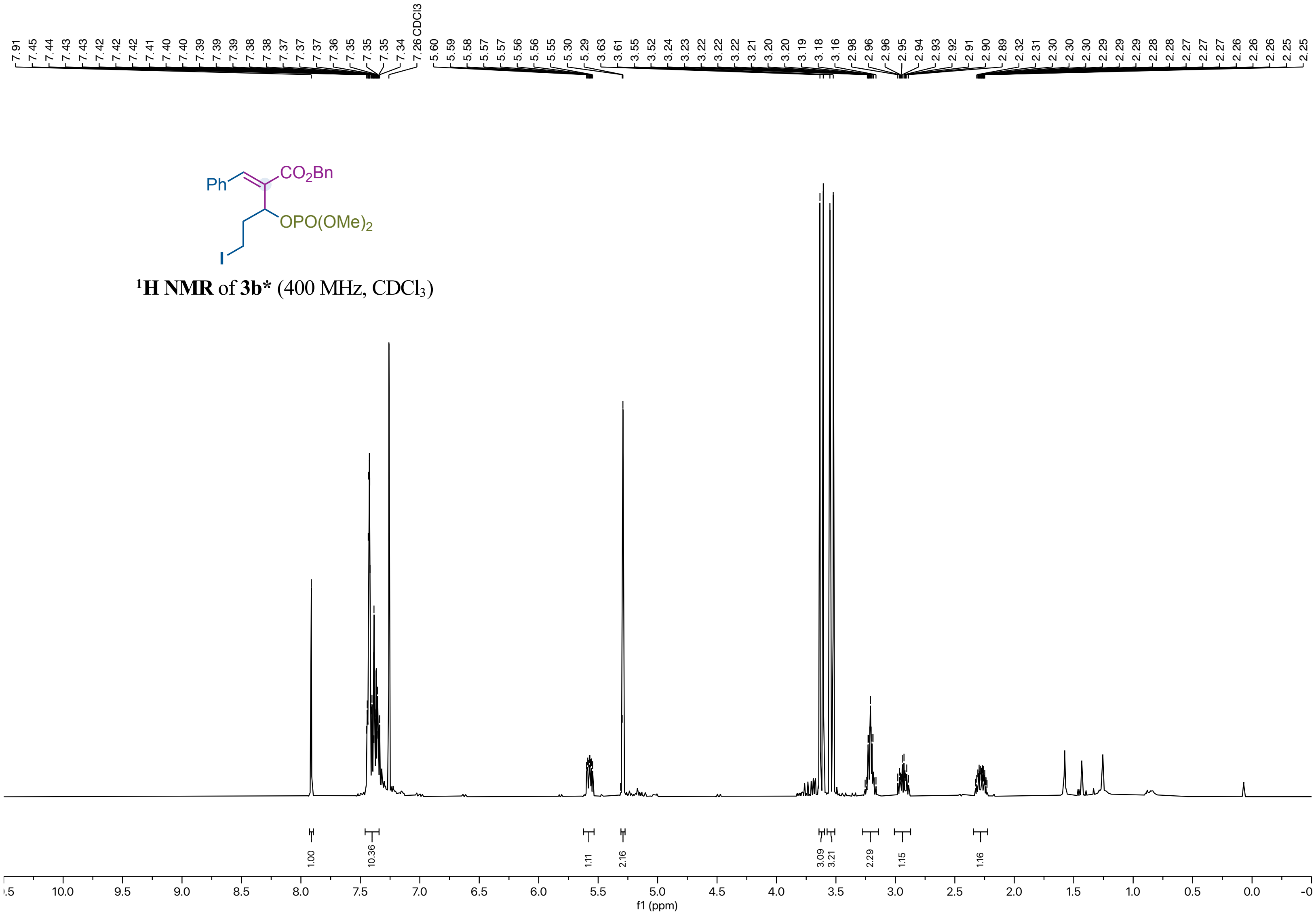

^{1}H NMR of **3b*** (400 MHz, $CDCl_3$)

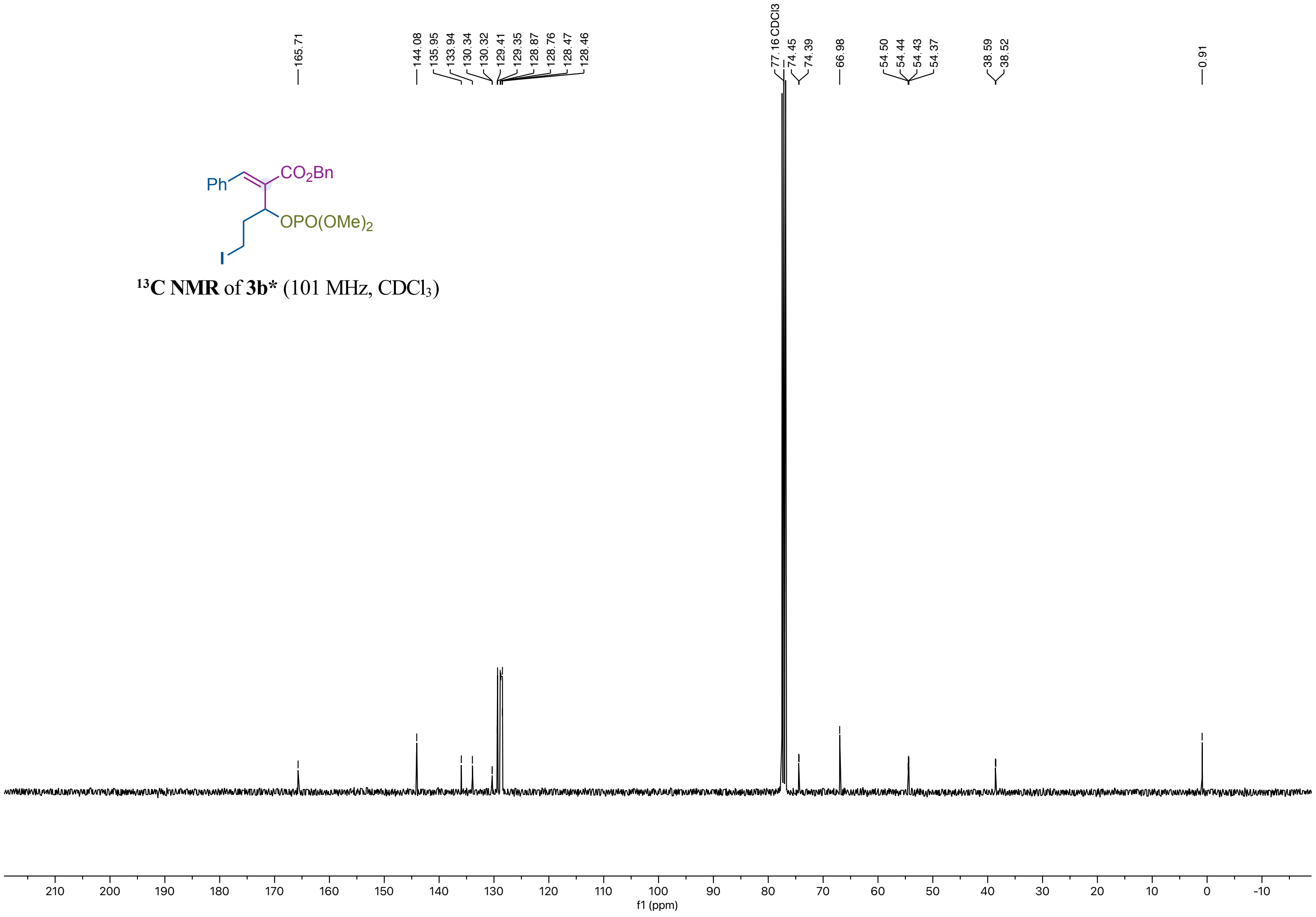

^{13}C NMR of **3b*** (101 MHz, $CDCl_3$)

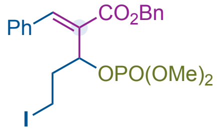

^{31}P NMR of **3b*** (162 MHz, $CDCl_3$)

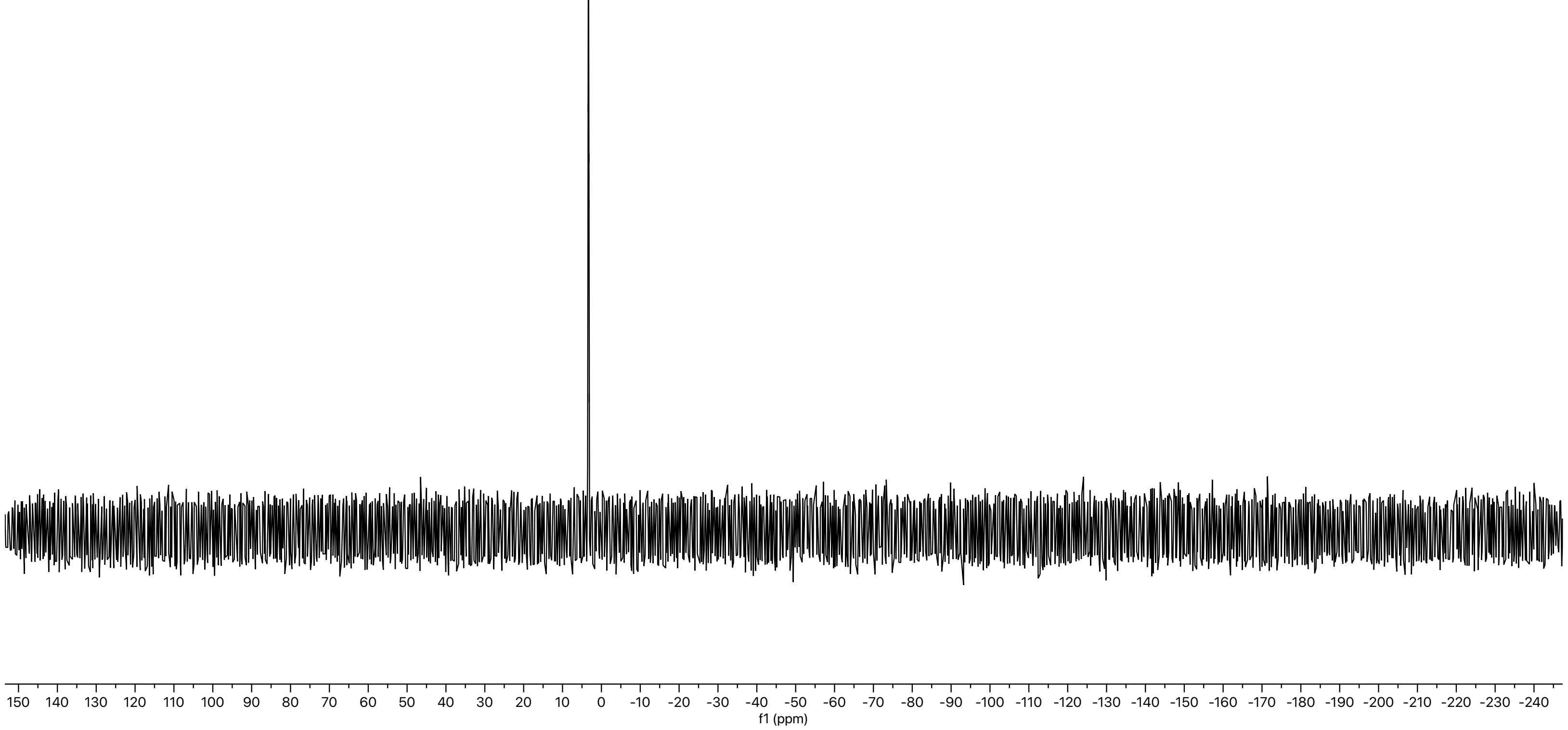

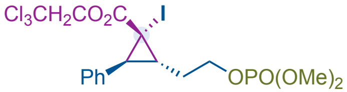

¹H NMR of **3c** (500 MHz, $CDCl_3$)

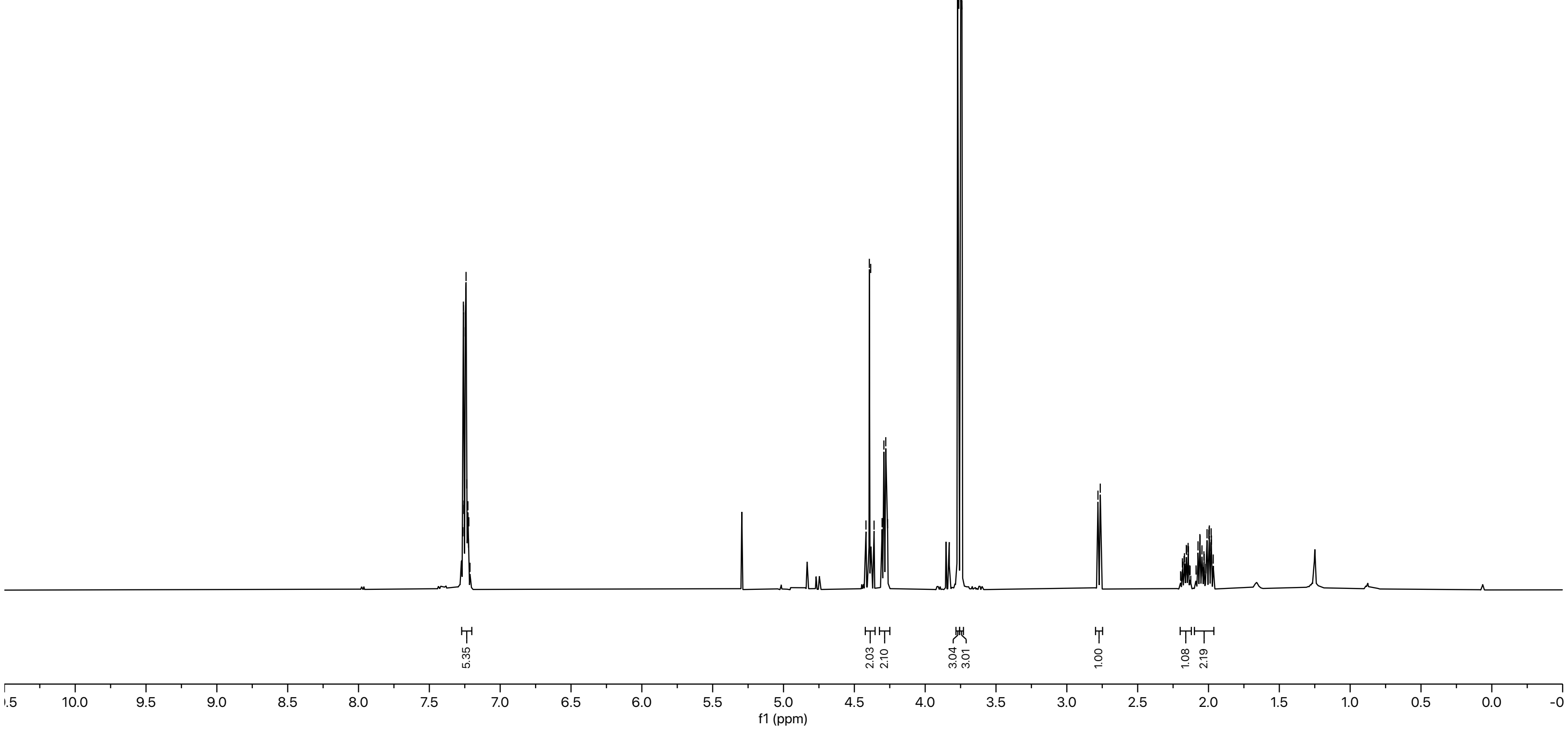

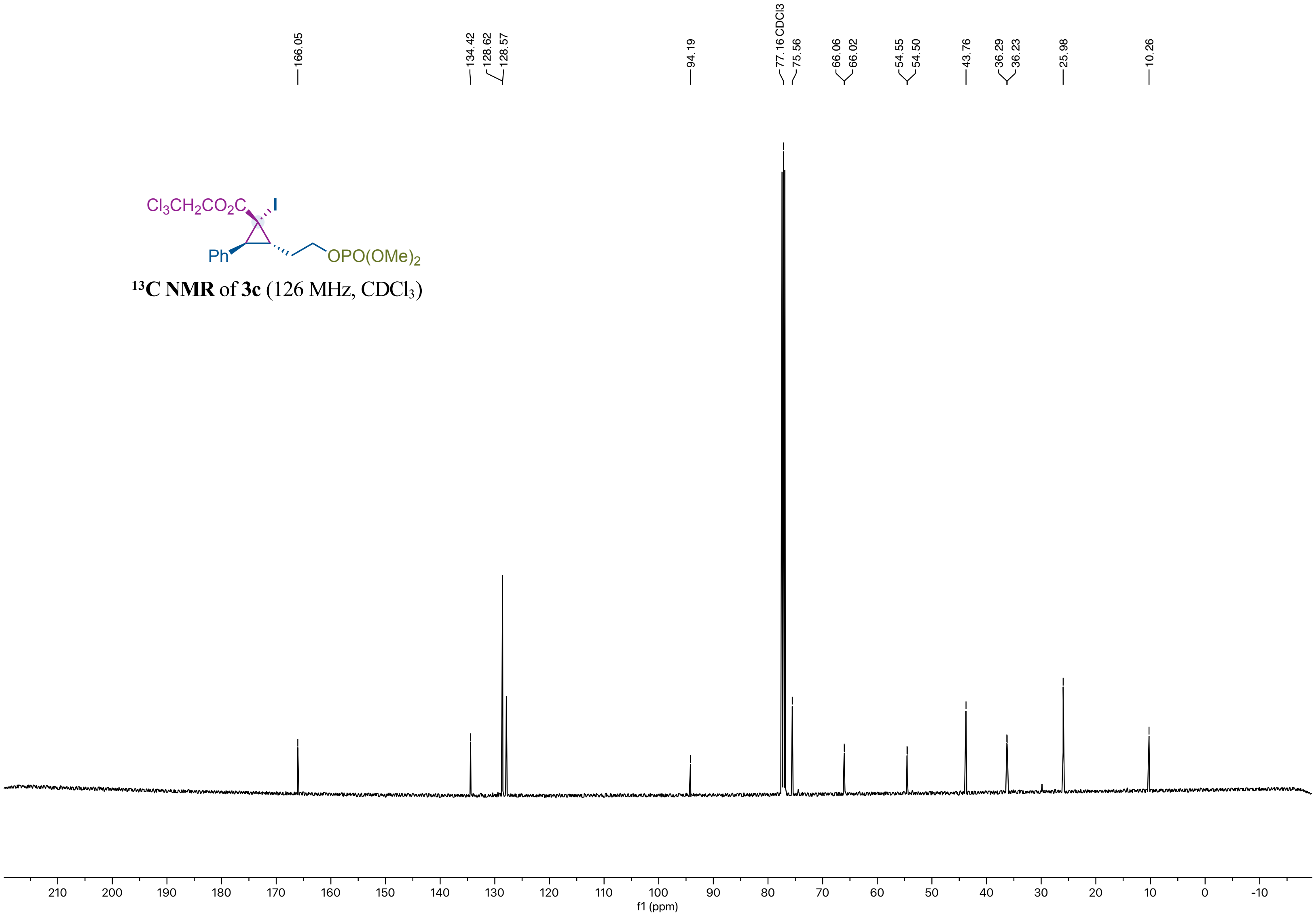

^{13}C NMR of **3c** (126 MHz, $CDCl_3$)

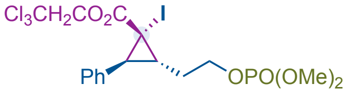

^{31}**P NMR** of **3c** (202 MHz, $CDCl_3$)

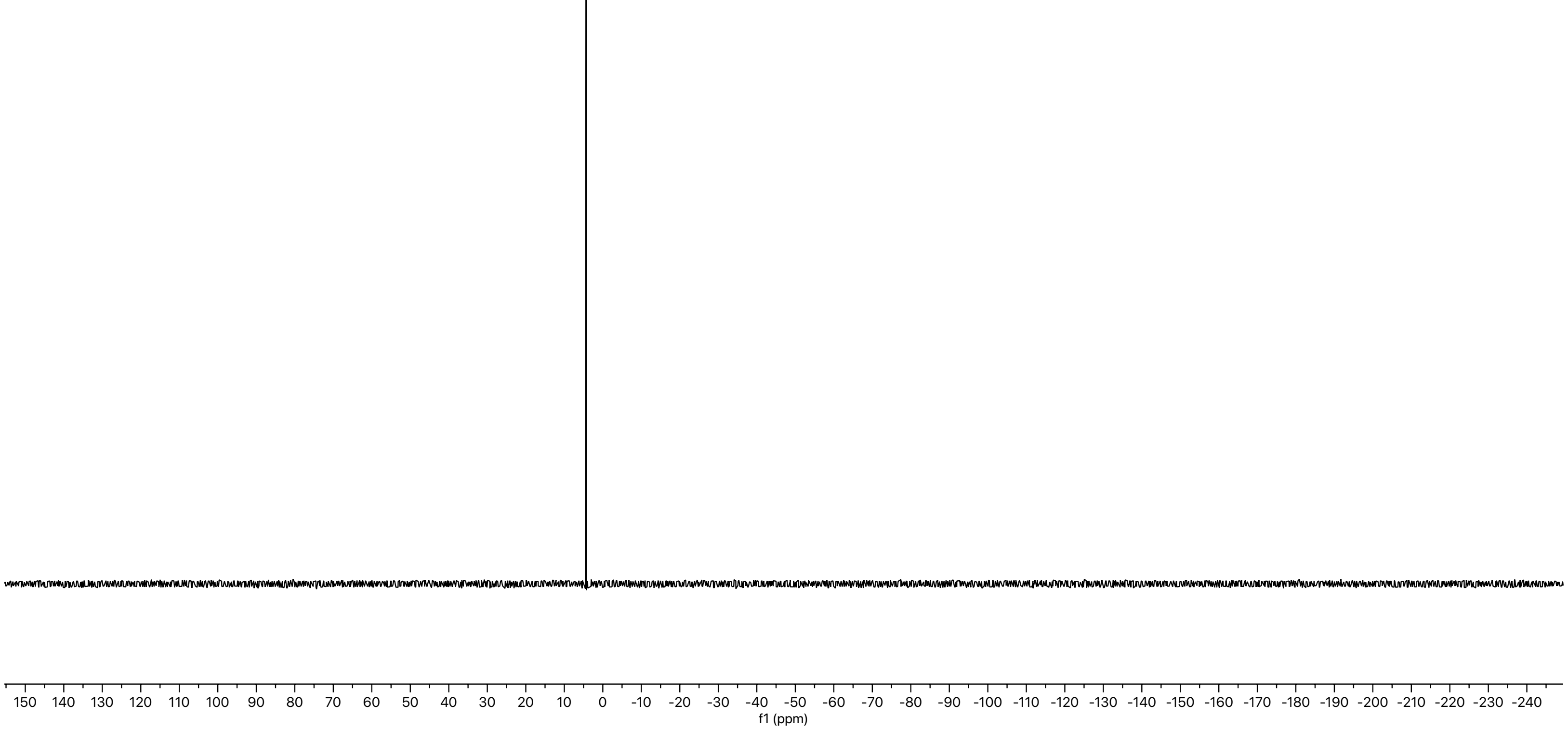

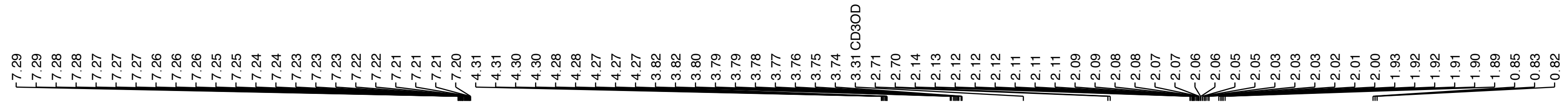

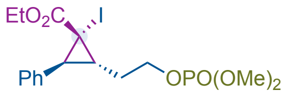

¹H NMR of **3a** (500 MHz, CD_3OD)

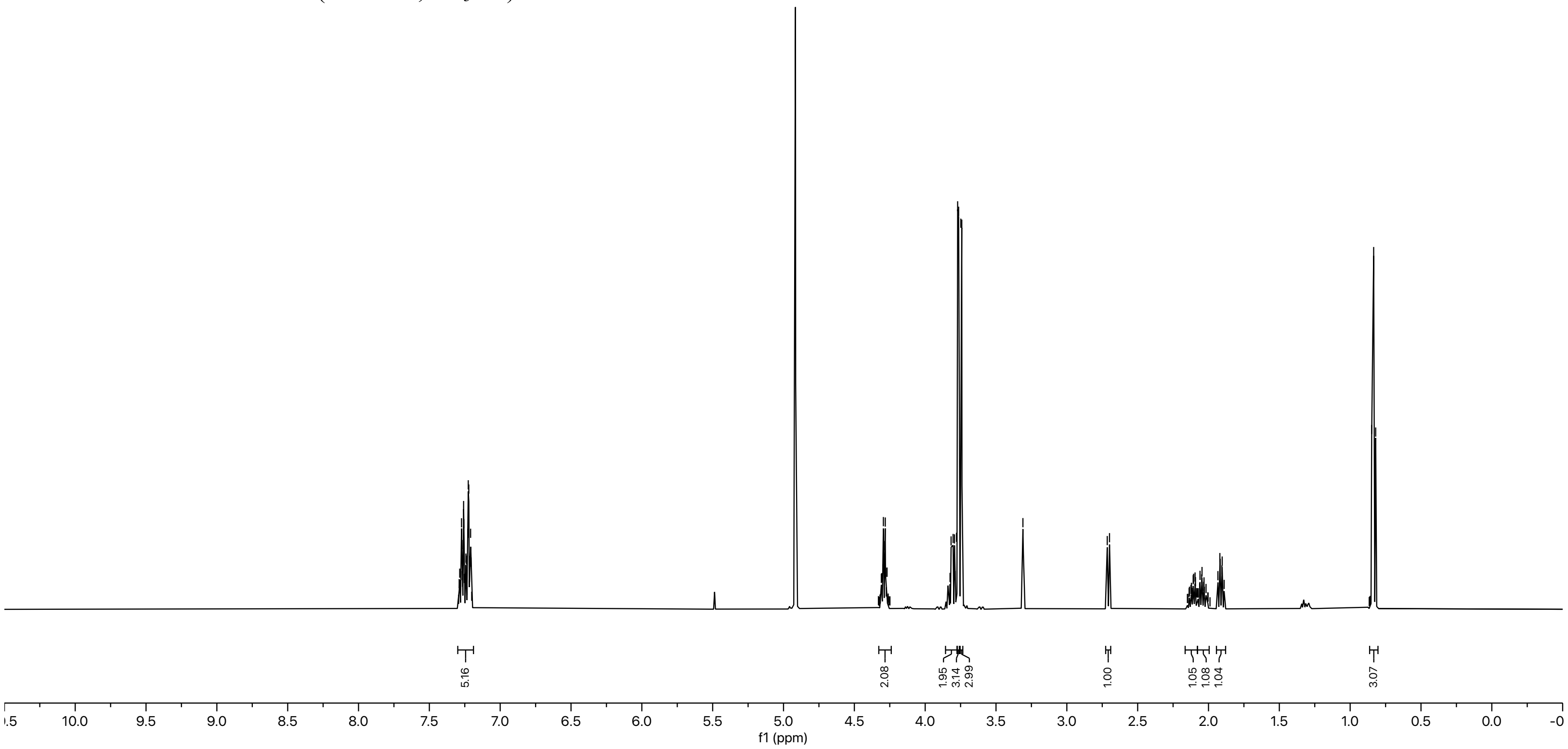

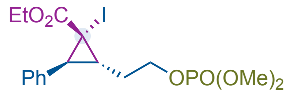

^{13}C NMR of **3a** (126 MHz, CD_3OD)

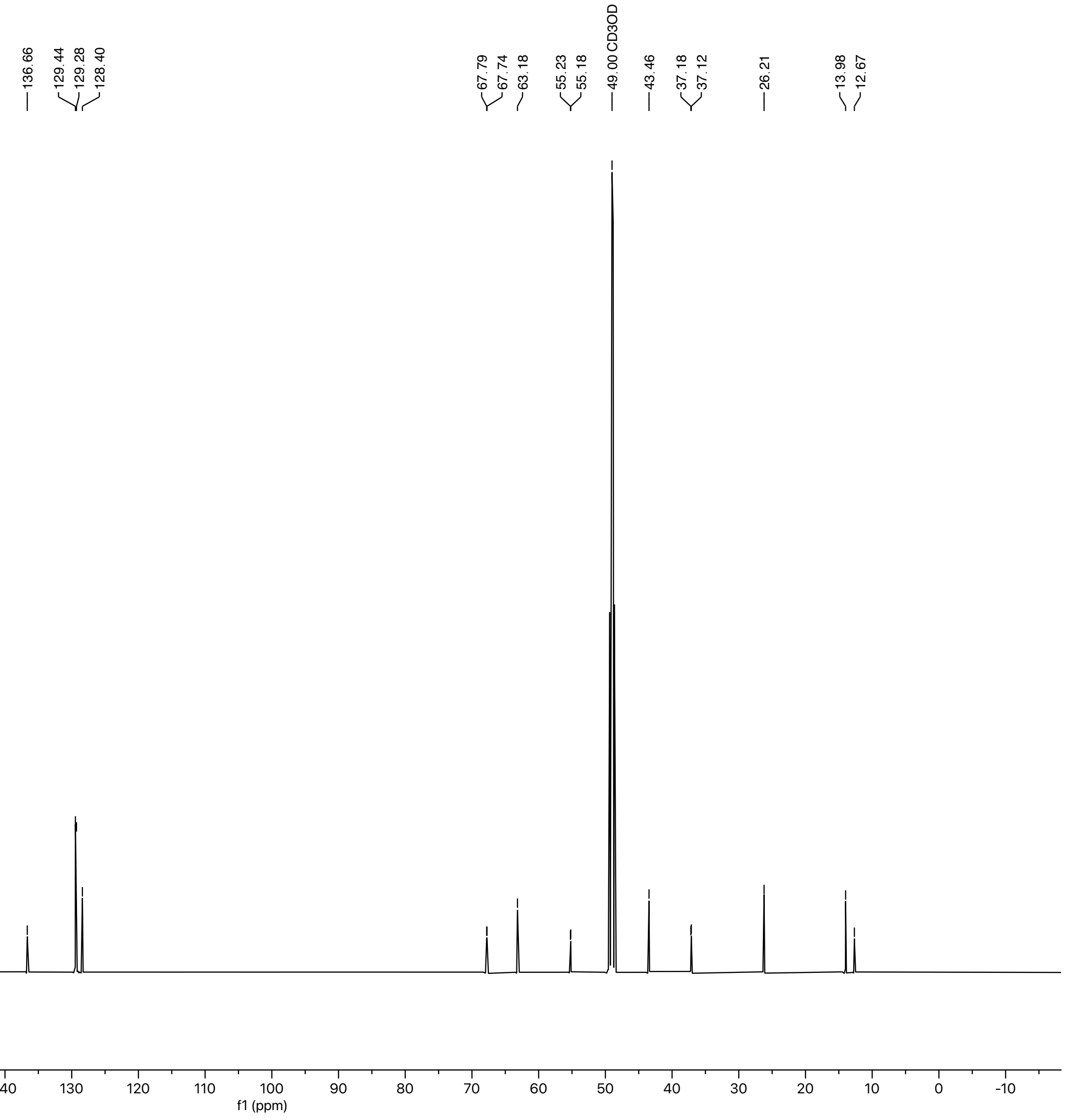

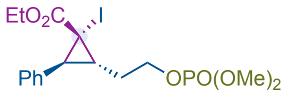

^{31}P NMR of **3a** (202 MHz, CD_3OD)

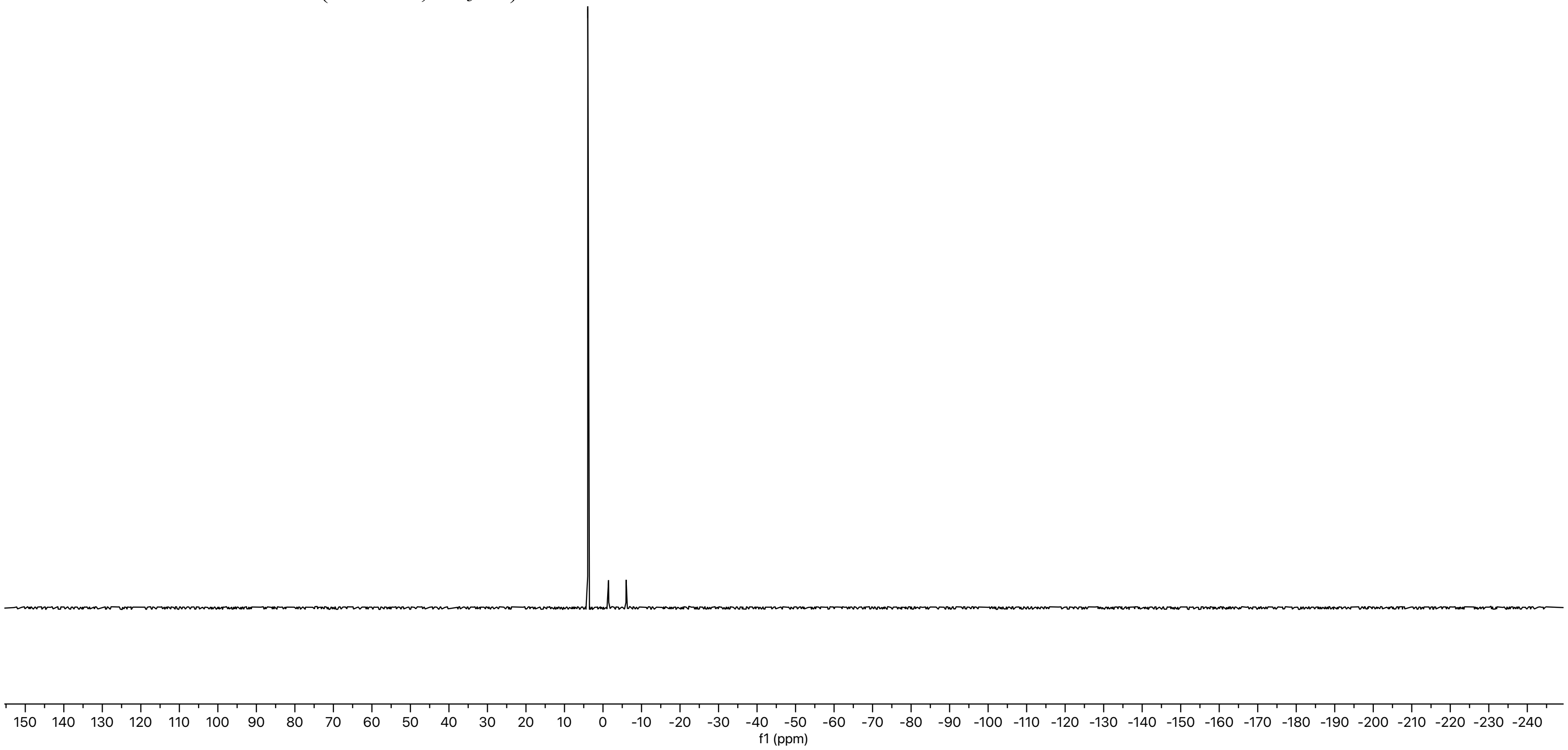

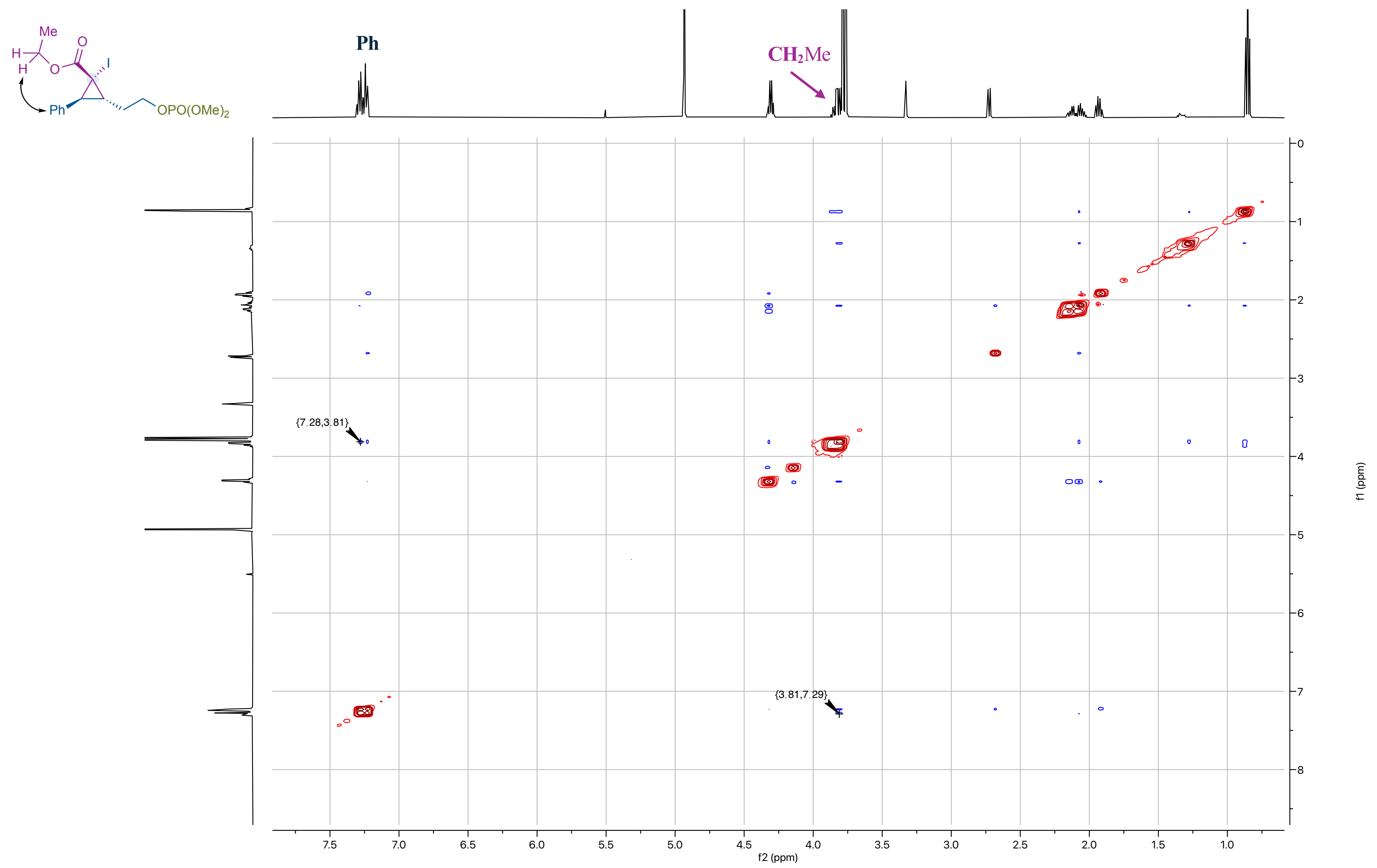
Me
O
I
H
H
O
Ph
OPO(OMe)2
Ph
CH2Me
{7.28,3.81}
{3.81,7.29}
f2 (ppm)
f1 (ppm)

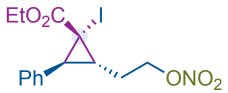

¹H NMR of **3d** (500 MHz, $CDCl_3$)

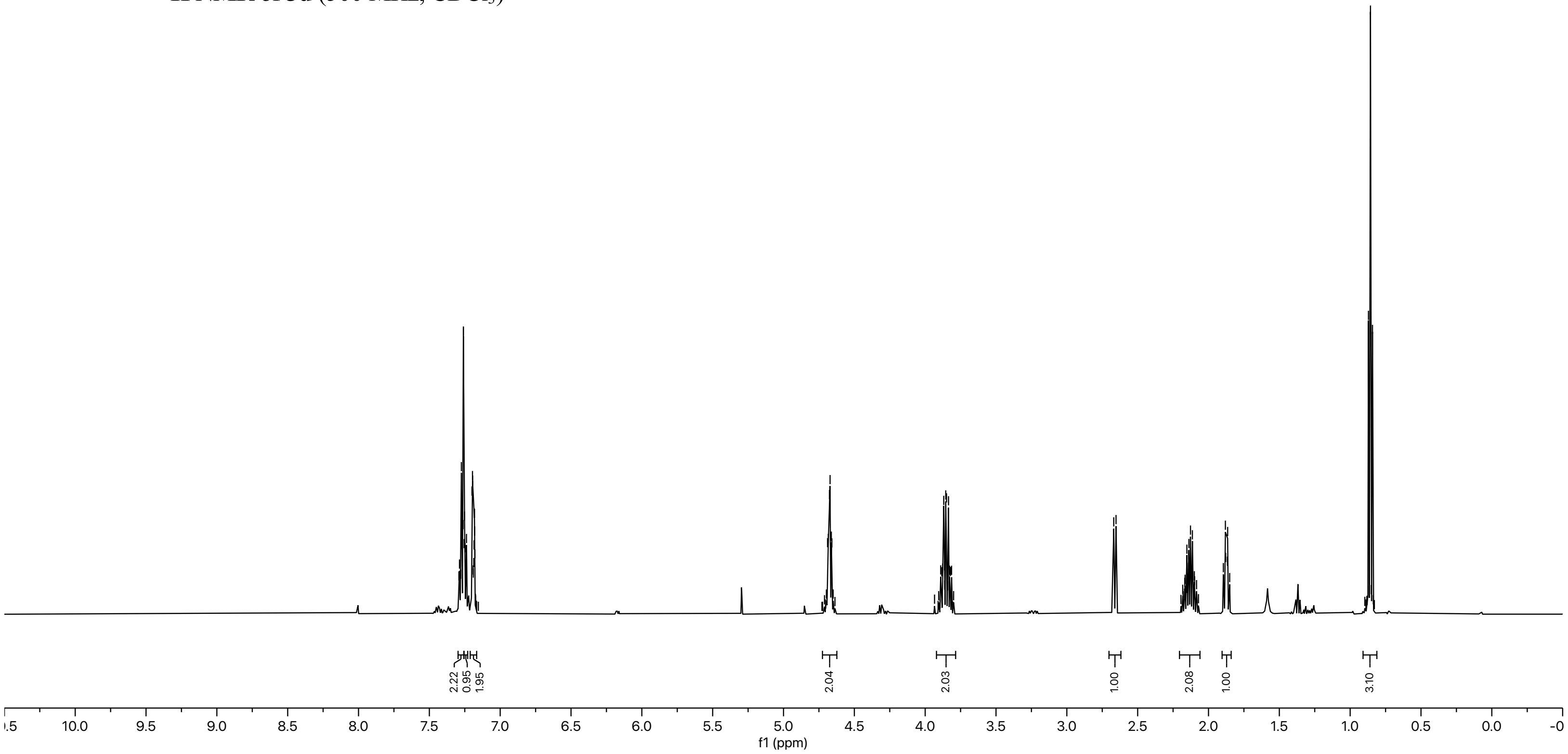

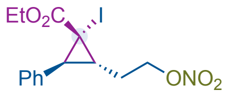

13C NMR of **3d** (126 MHz, $CDCl_3$)

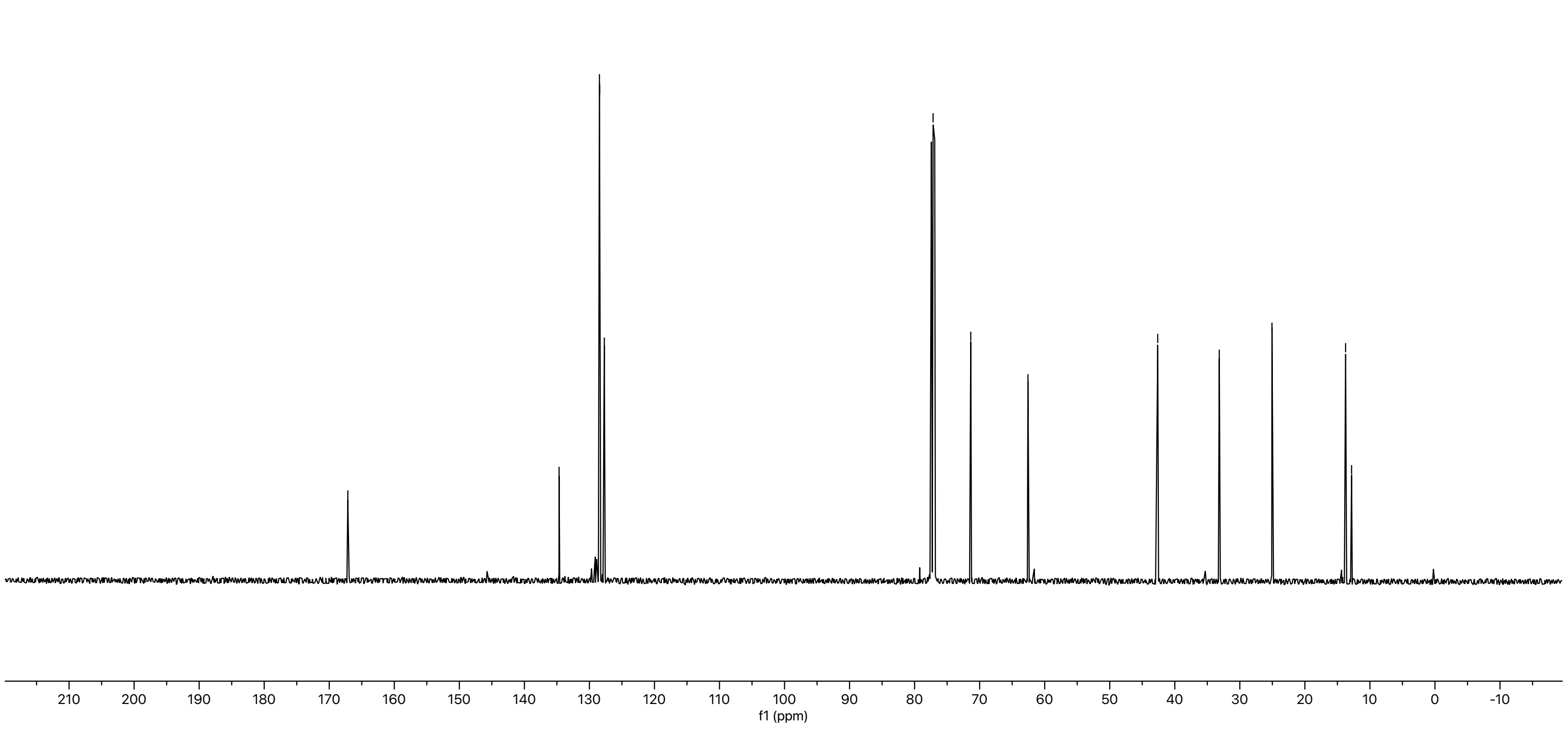

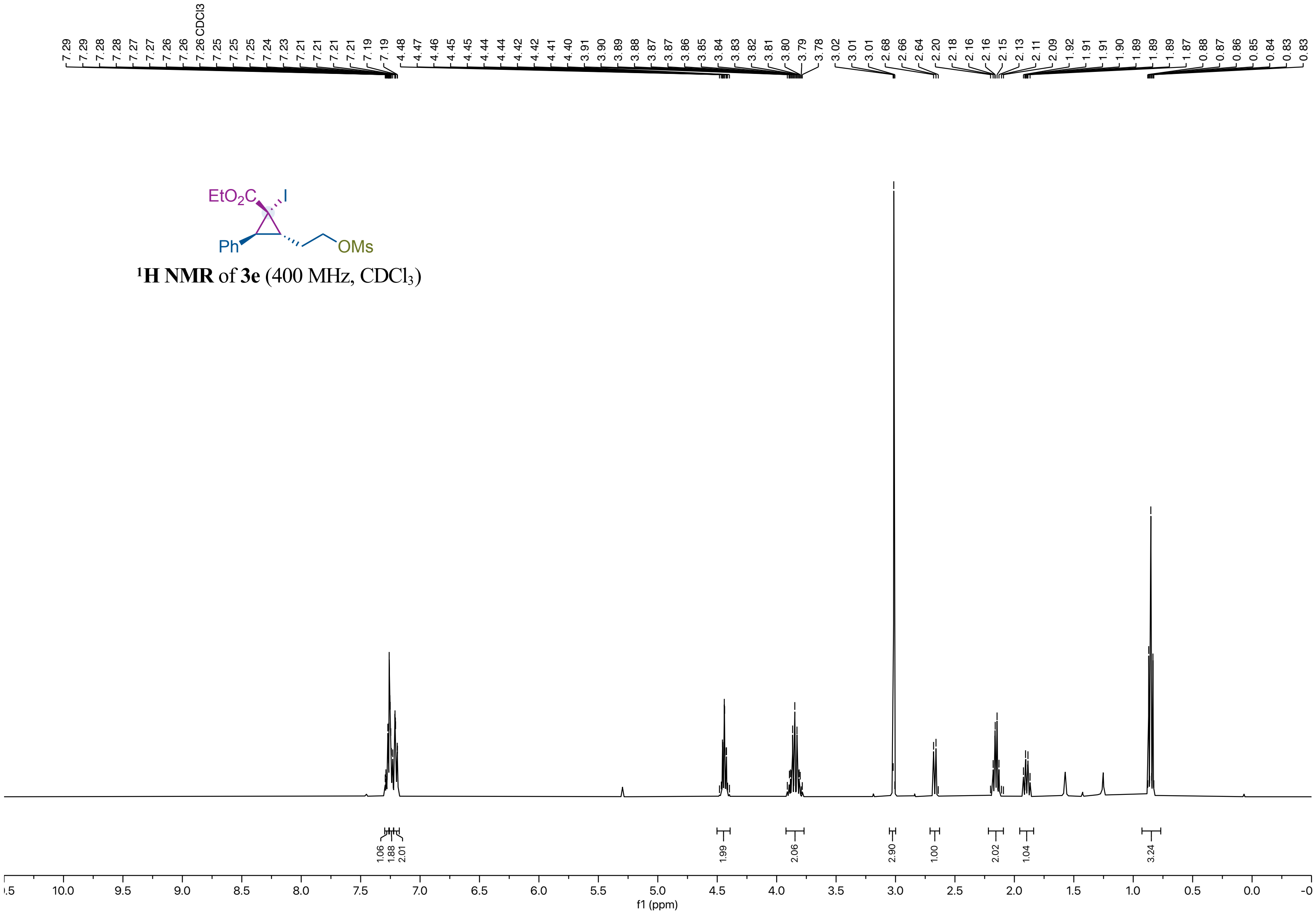

^{1}**H NMR** of **3e** (400 MHz, $CDCl_3$)

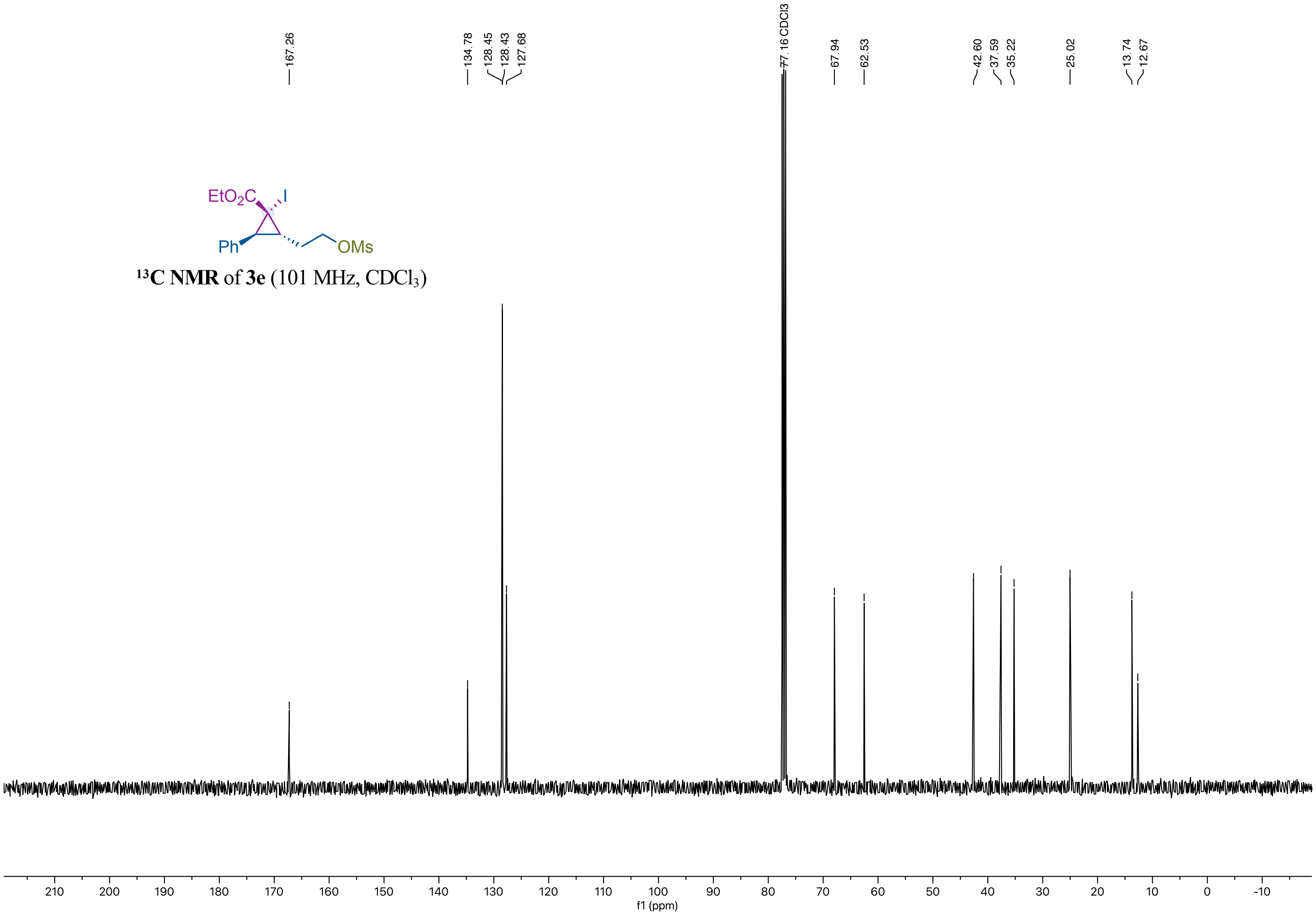

^{13}C NMR of **3e** (101 MHz, $CDCl_3$)

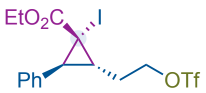

¹H NMR of **3f** (400 MHz, $CDCl_3$)

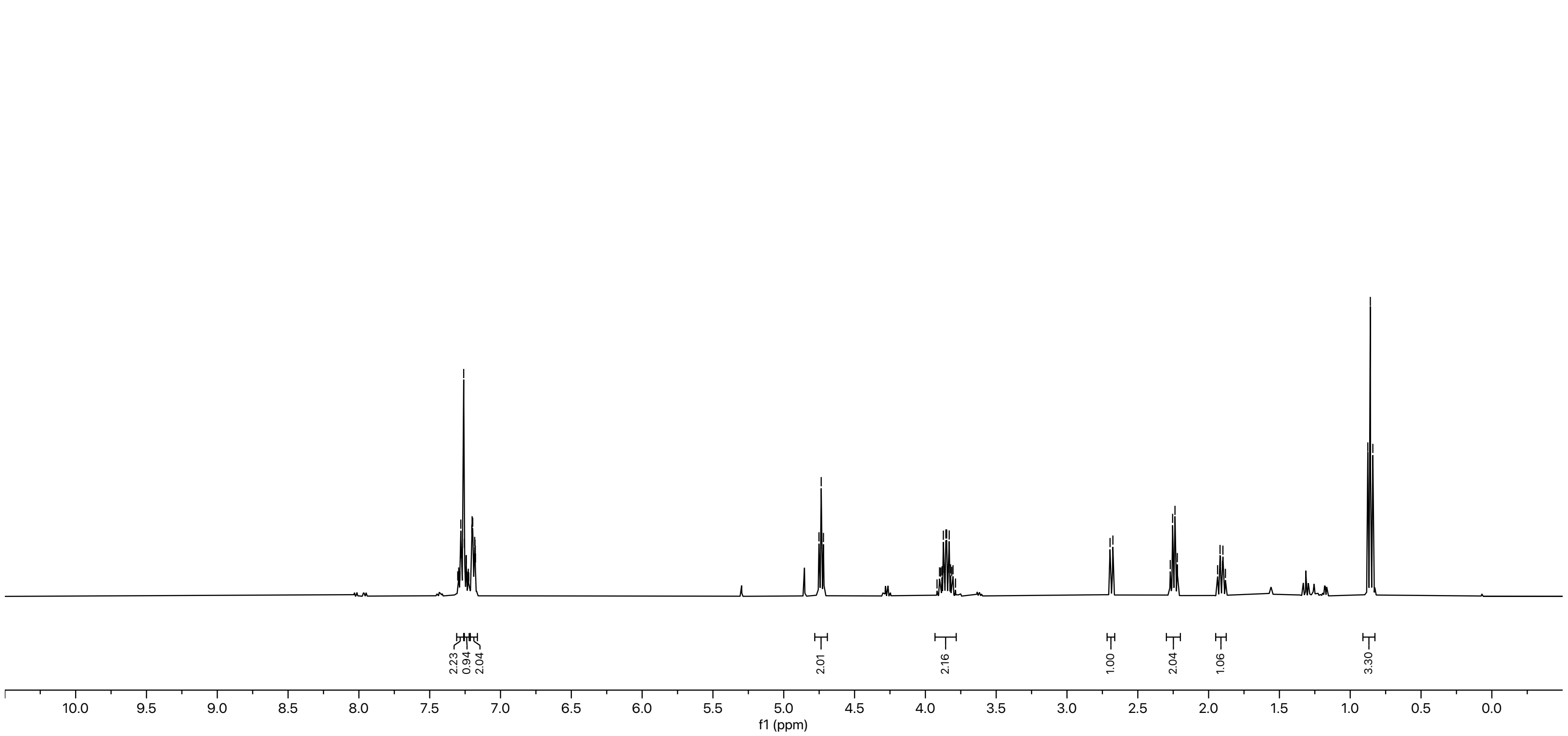

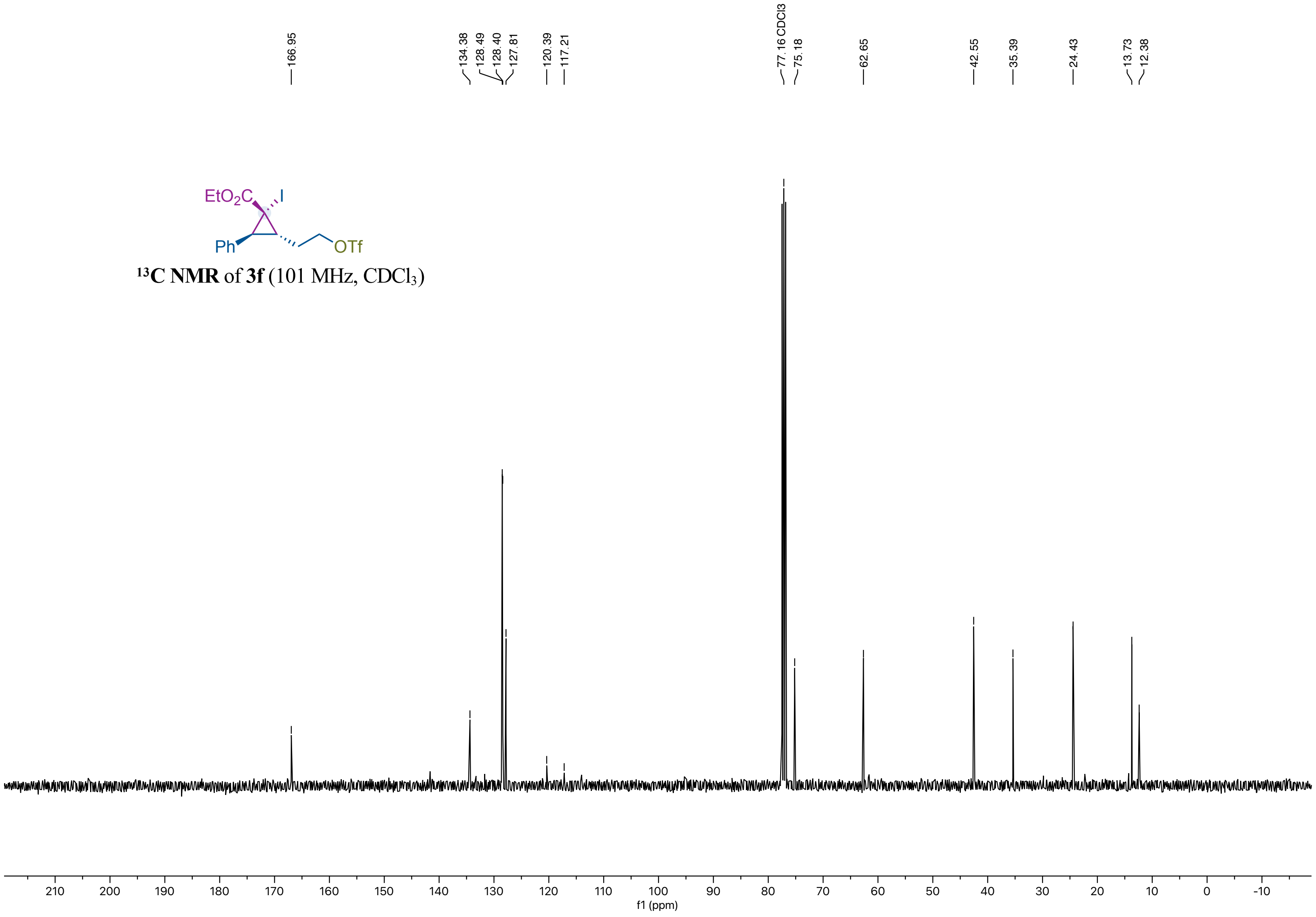

^{13}C NMR of **3f** (101 MHz, $CDCl_3$)

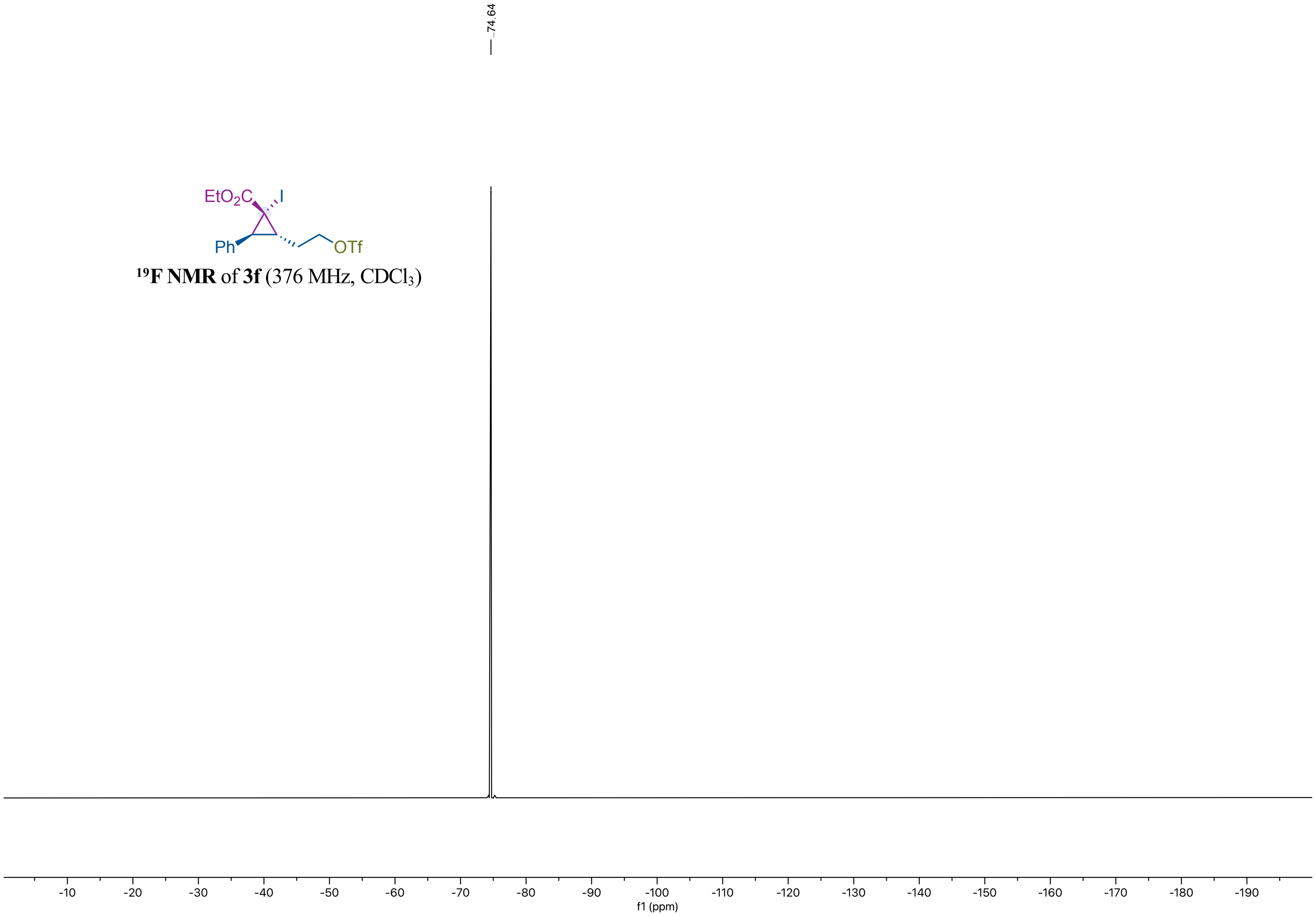

^{19}F NMR of **3f** (376 MHz, $CDCl_3$)

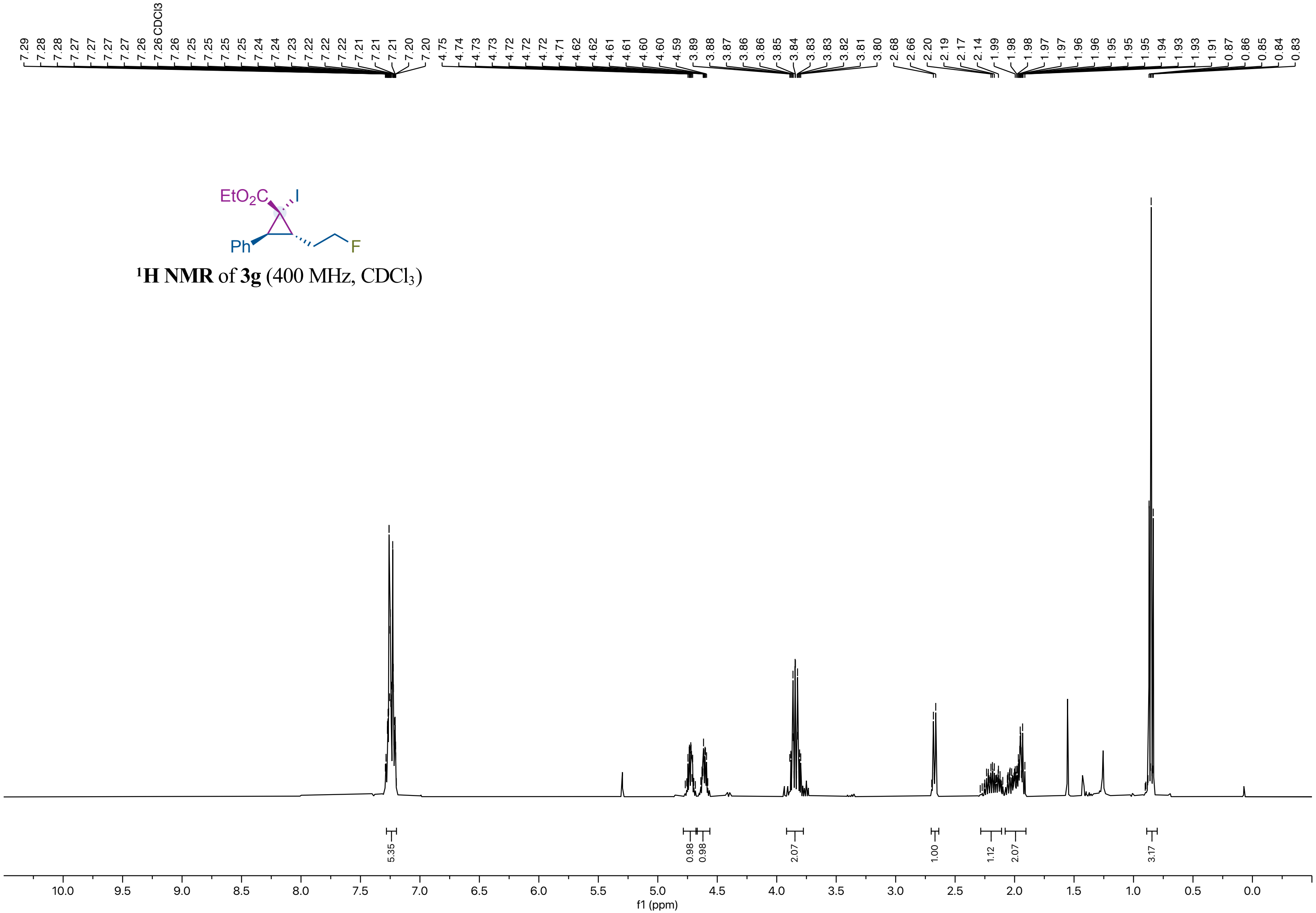

^{1}H NMR of **3g** (400 MHz, $CDCl_3$)

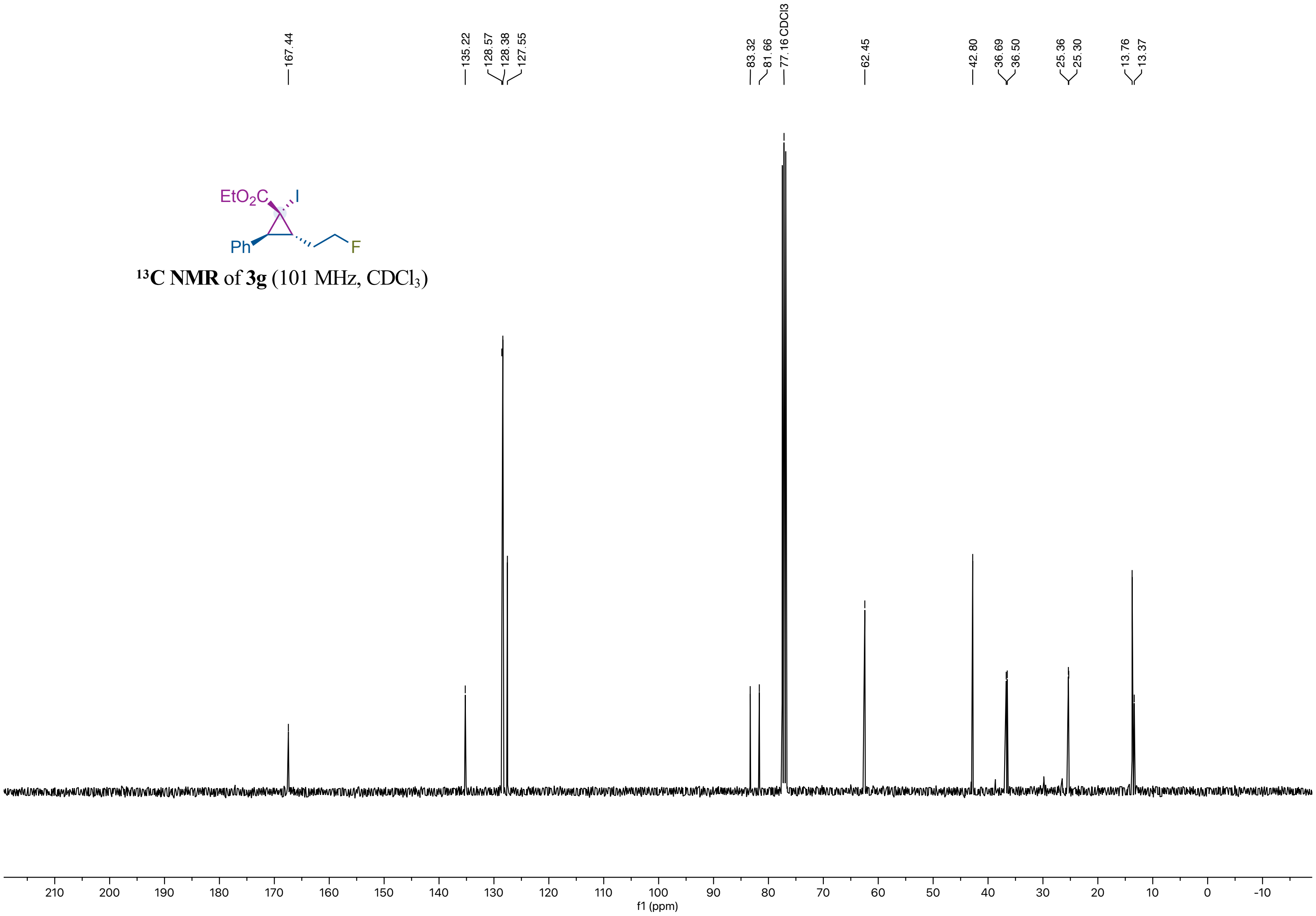

^{13}C NMR of **3g** (101 MHz, $CDCl_3$)

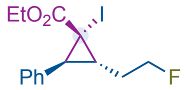

^{19}F NMR of **3g** (376 MHz, $CDCl_3$)

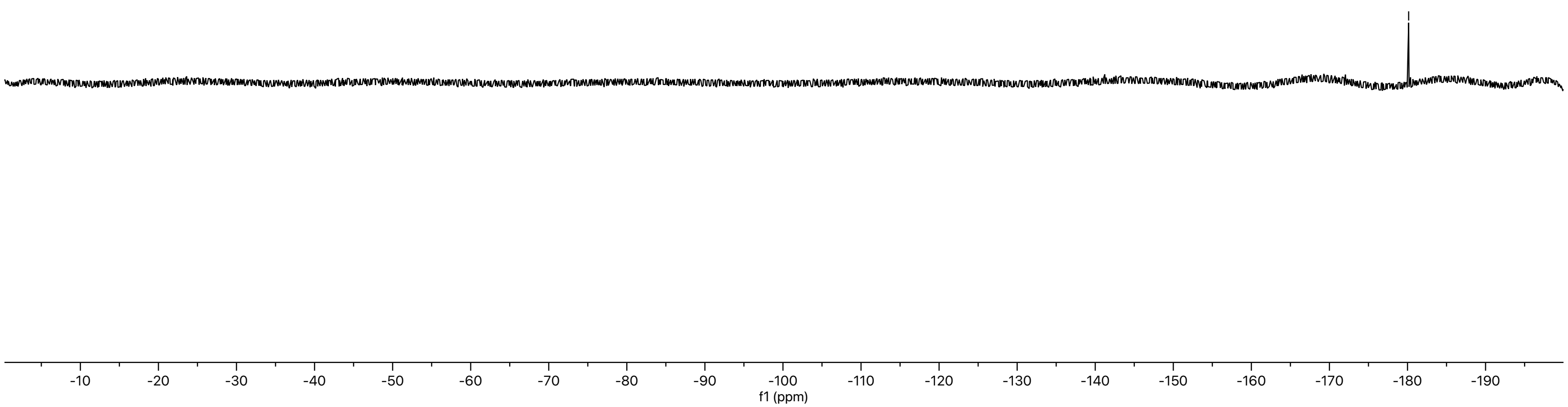

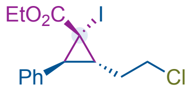

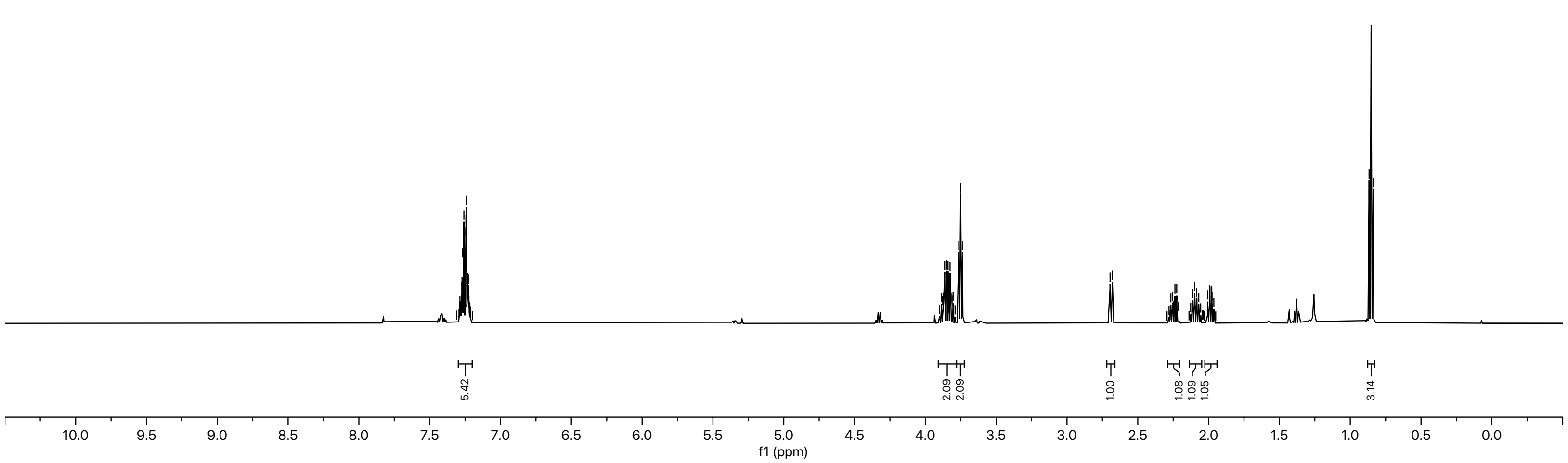

^{1}H NMR of **3h** (500 MHz, $CDCl_3$)

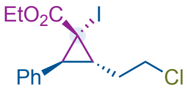

^{13}C NMR of **3h** (126 MHz, $CDCl_3$)

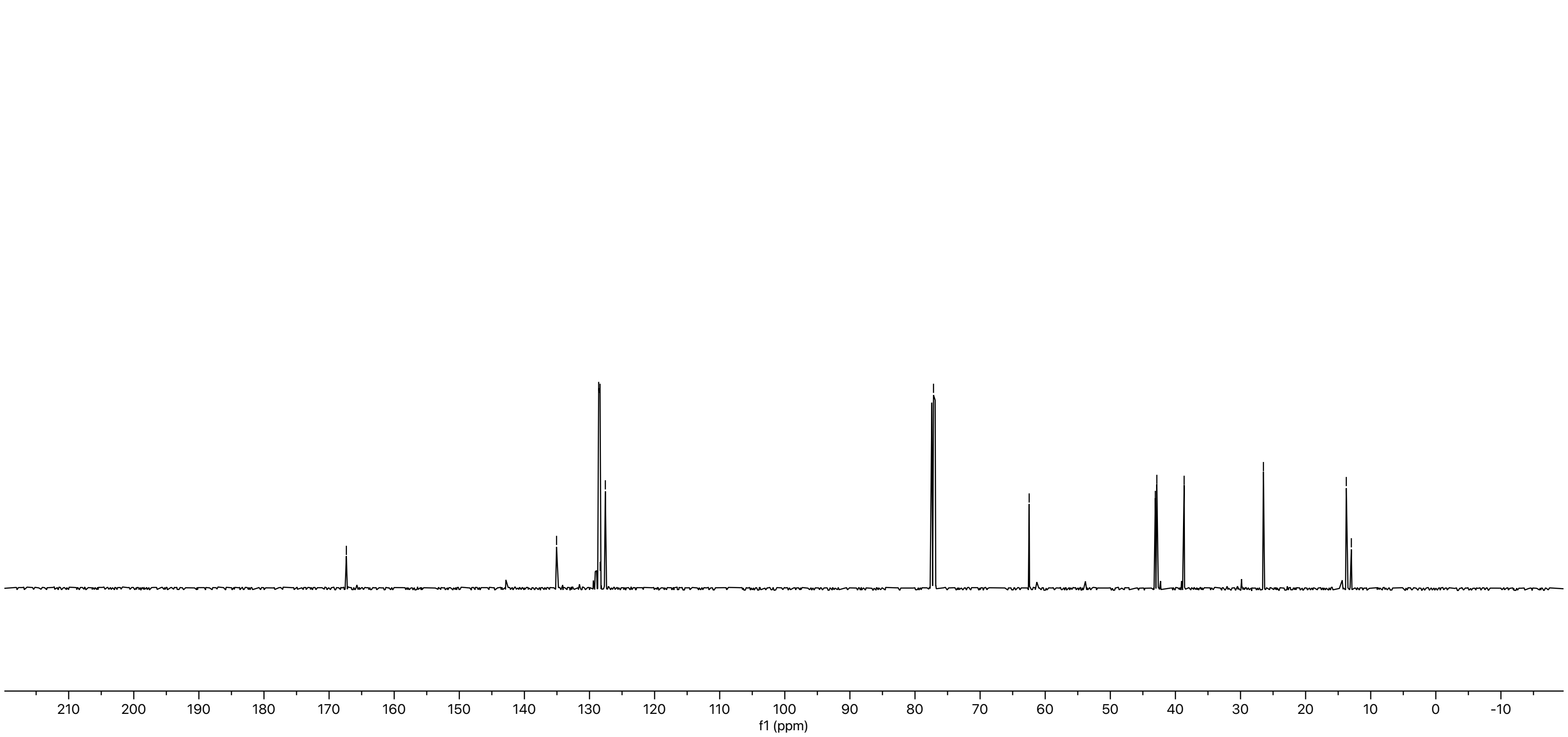

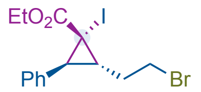

^{1}H NMR of **3i** (500 MHz, $CDCl_3$)

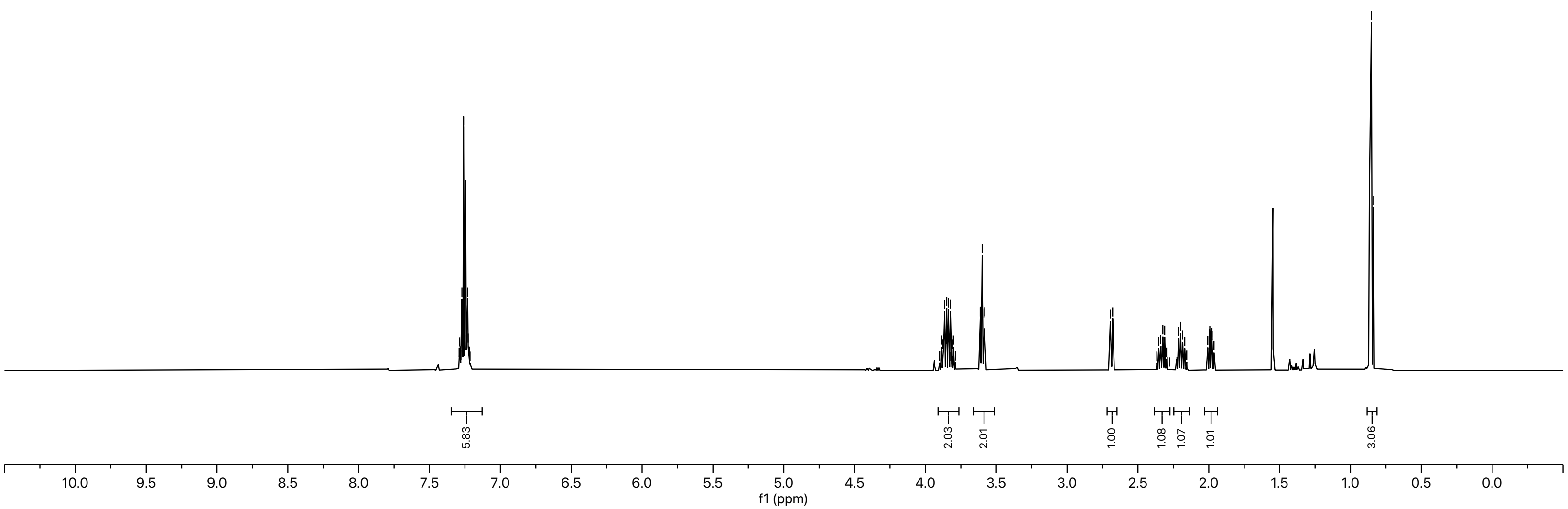

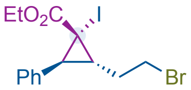

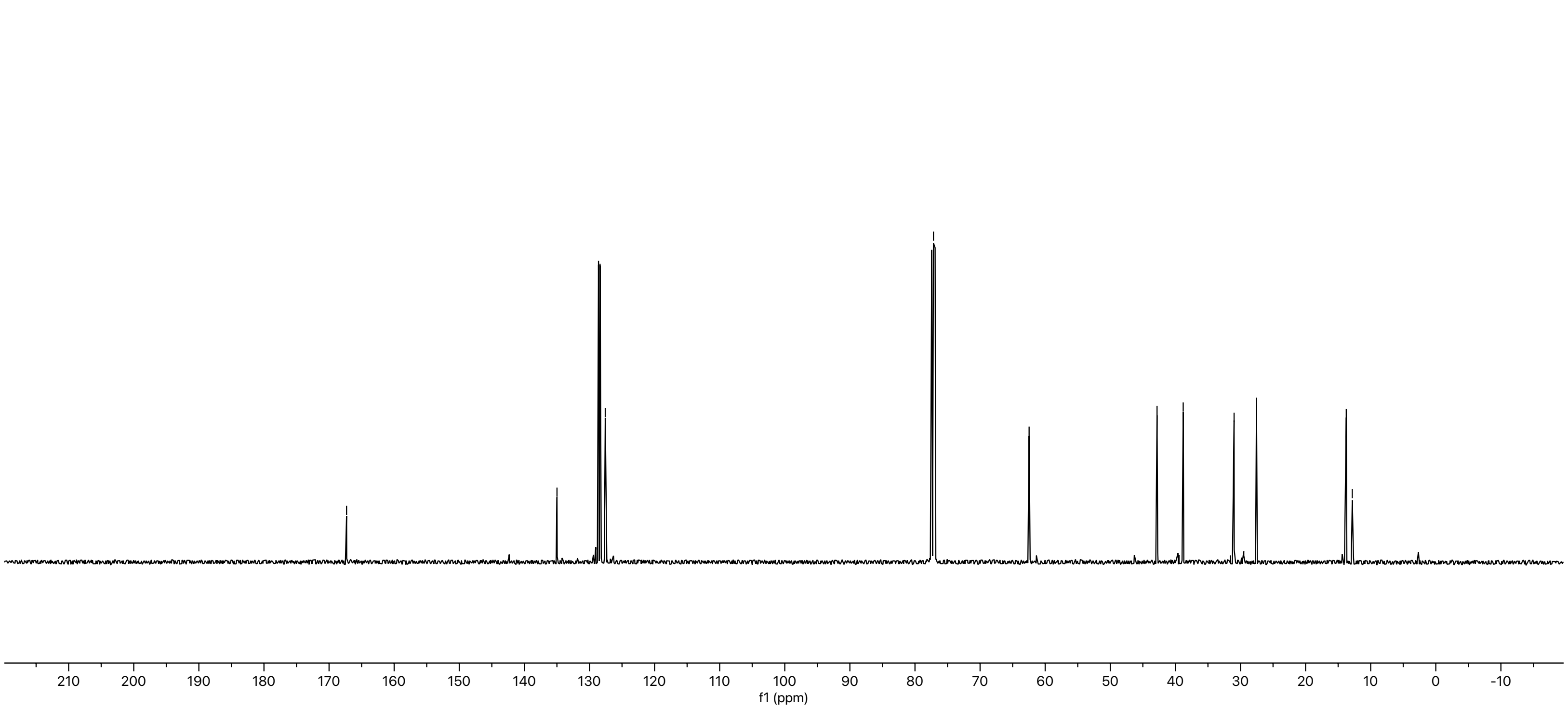

^{13}C NMR of **3i** (126 MHz, $CDCl_3$)

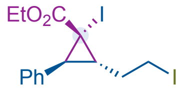

^{1}H NMR of **3j** (400 MHz, $CDCl_3$)

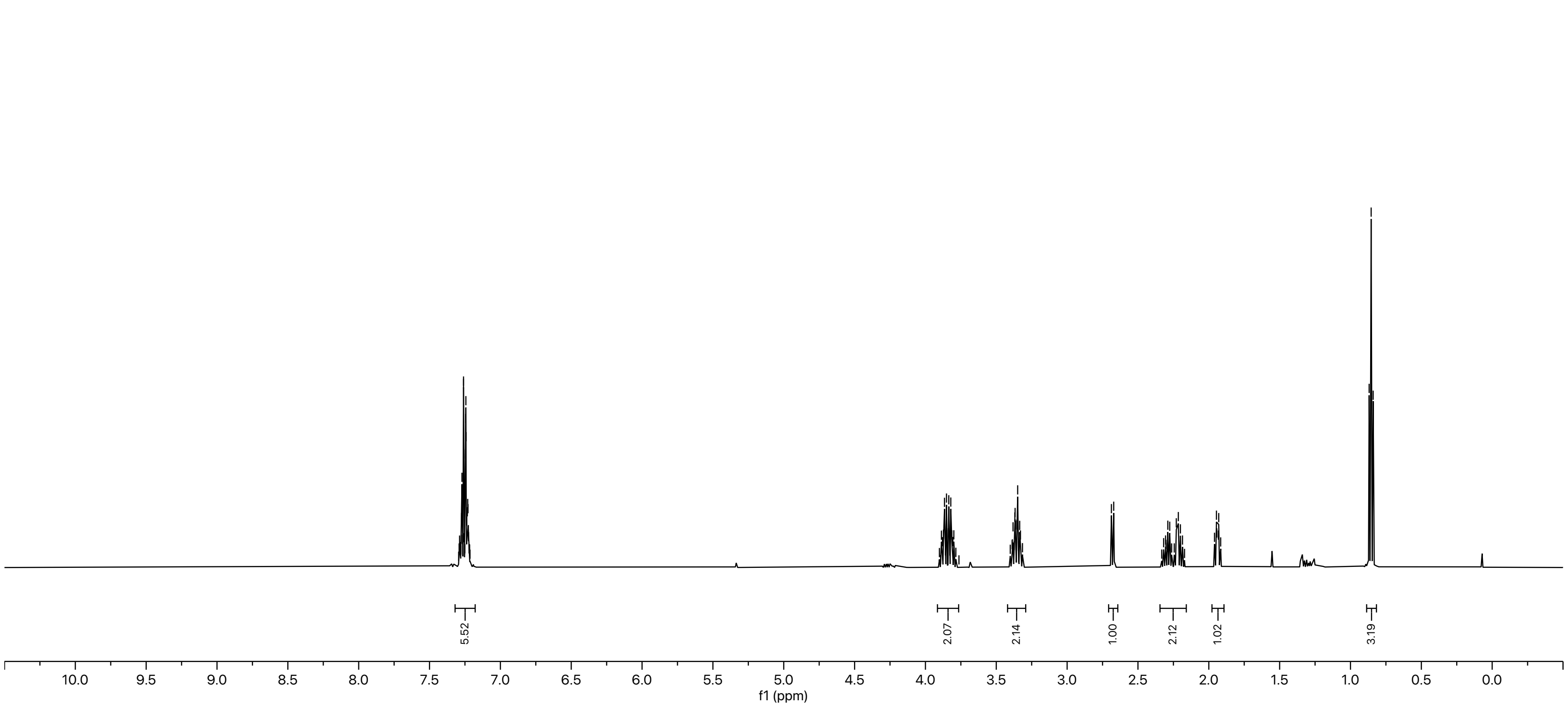

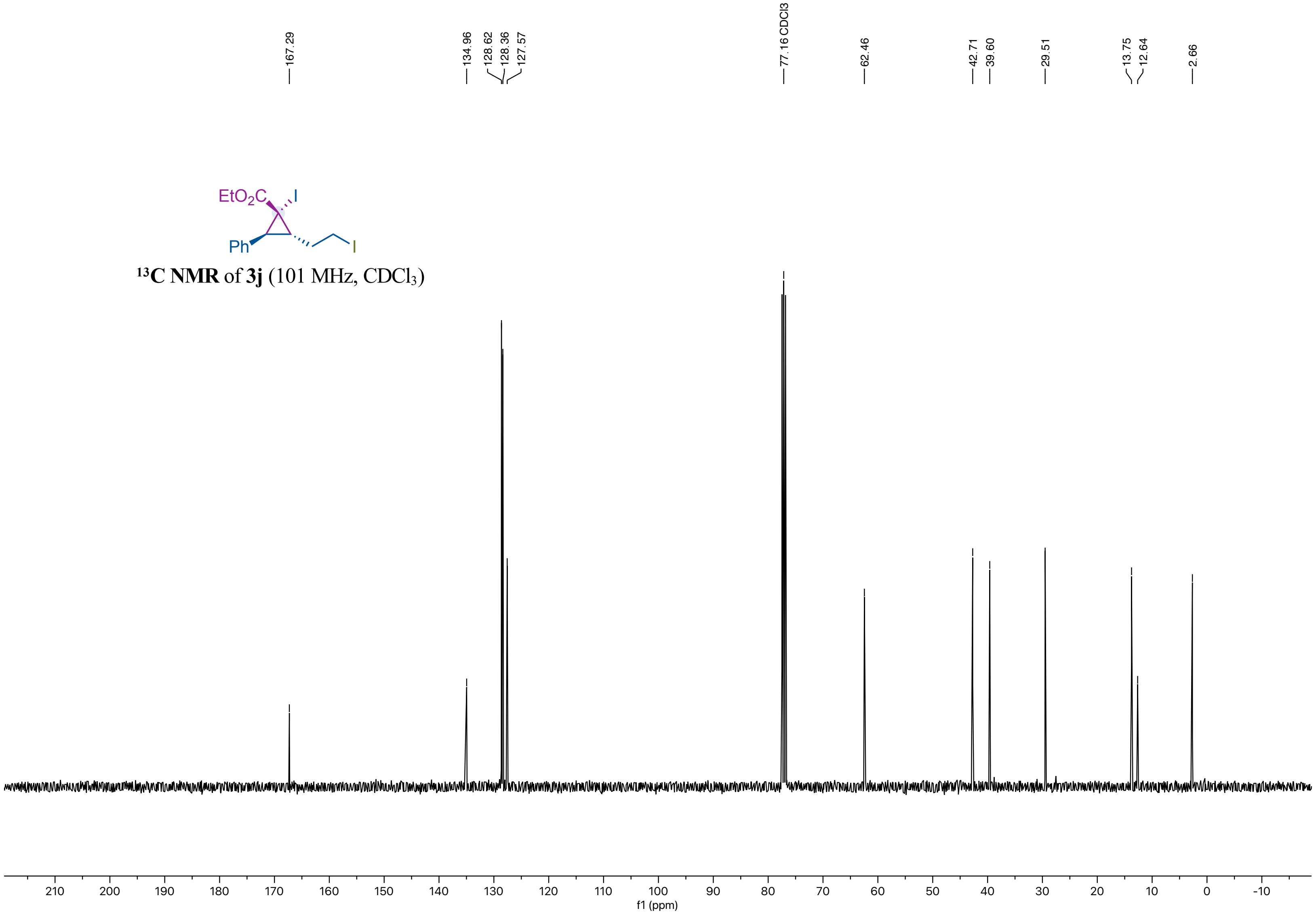

13C NMR of **3j** (101 MHz, $CDCl_3$)

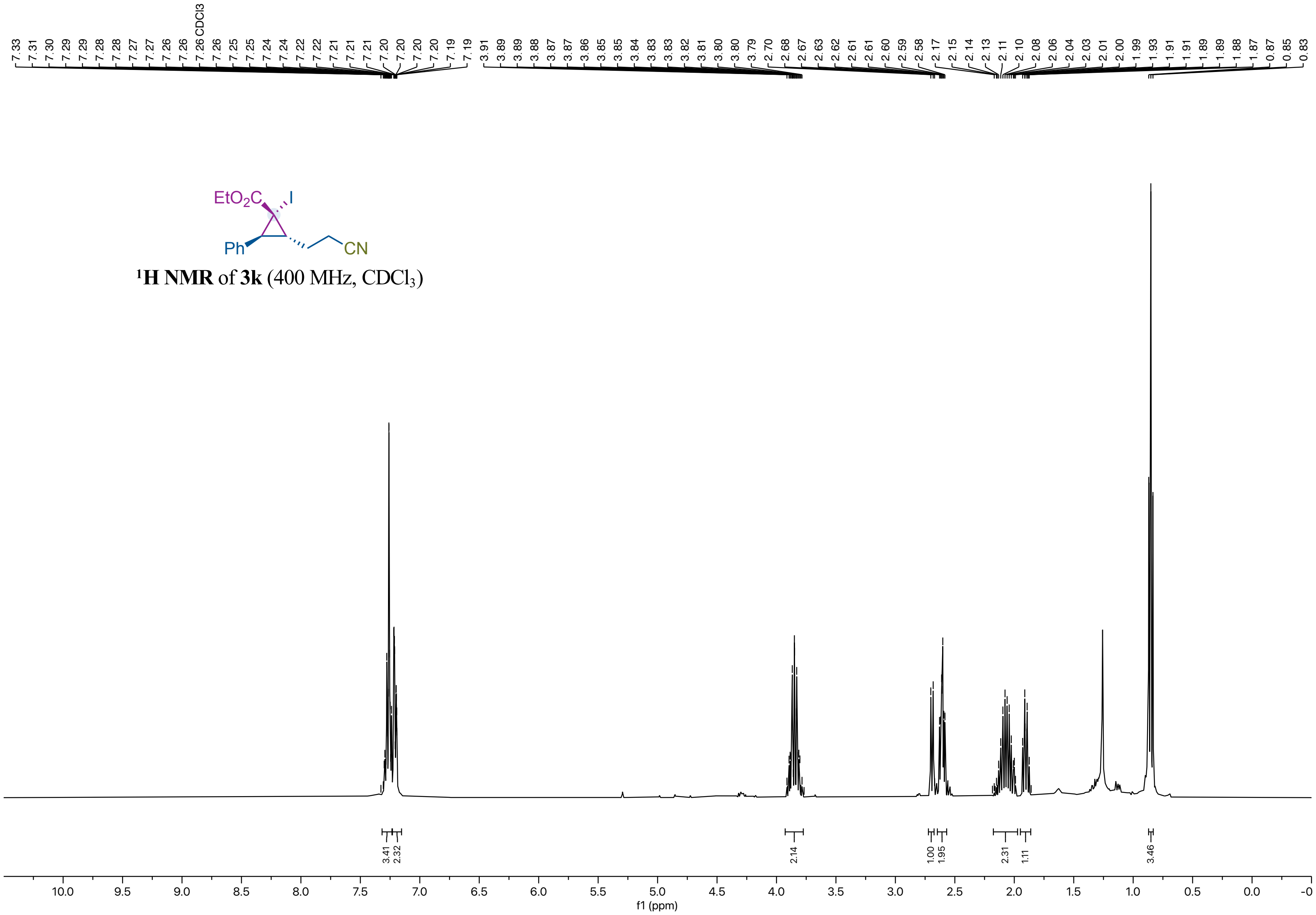

^{1}H NMR of **3k** (400 MHz, $CDCl_3$)

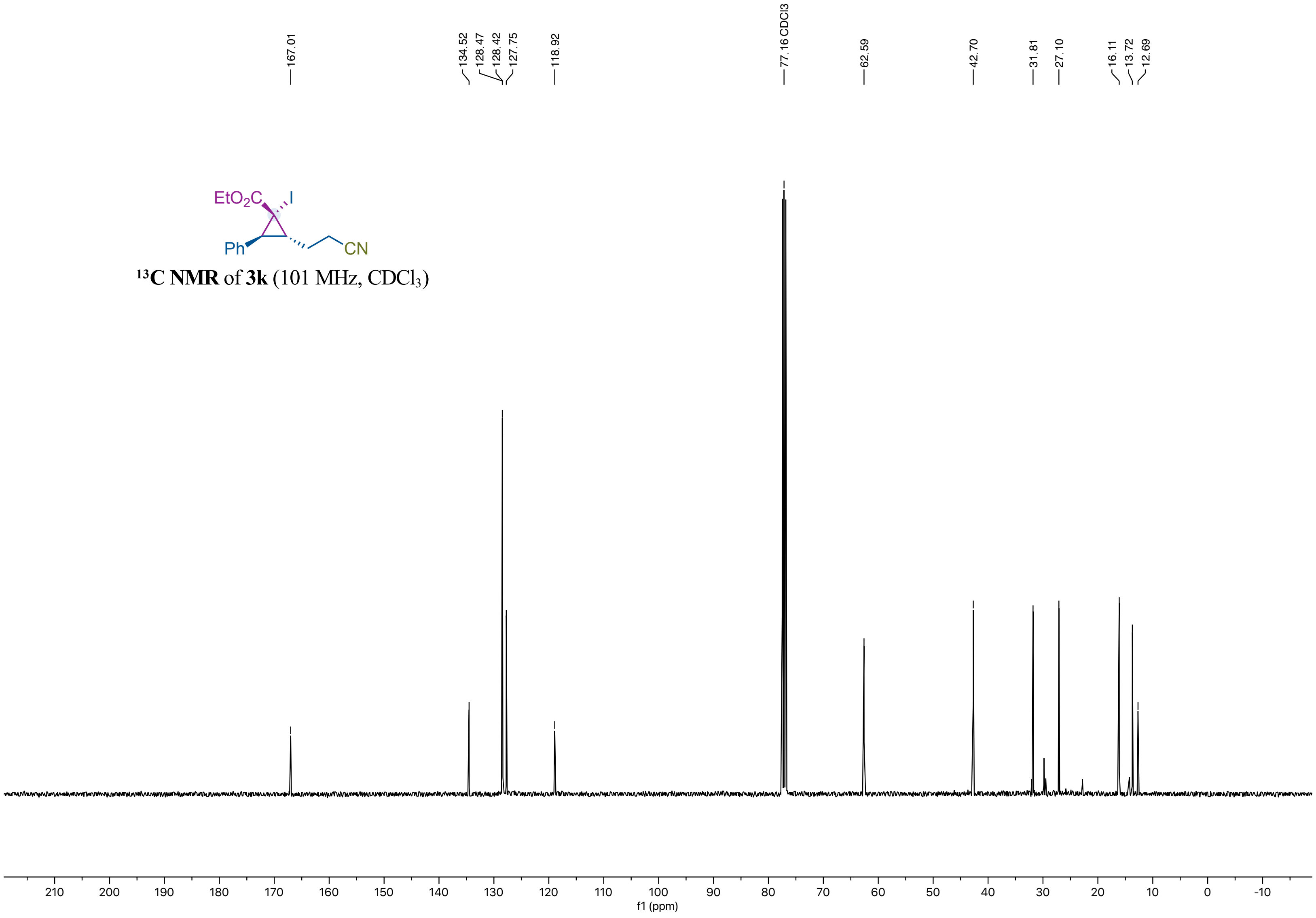

^{13}C NMR of **3k** (101 MHz, $CDCl_3$)

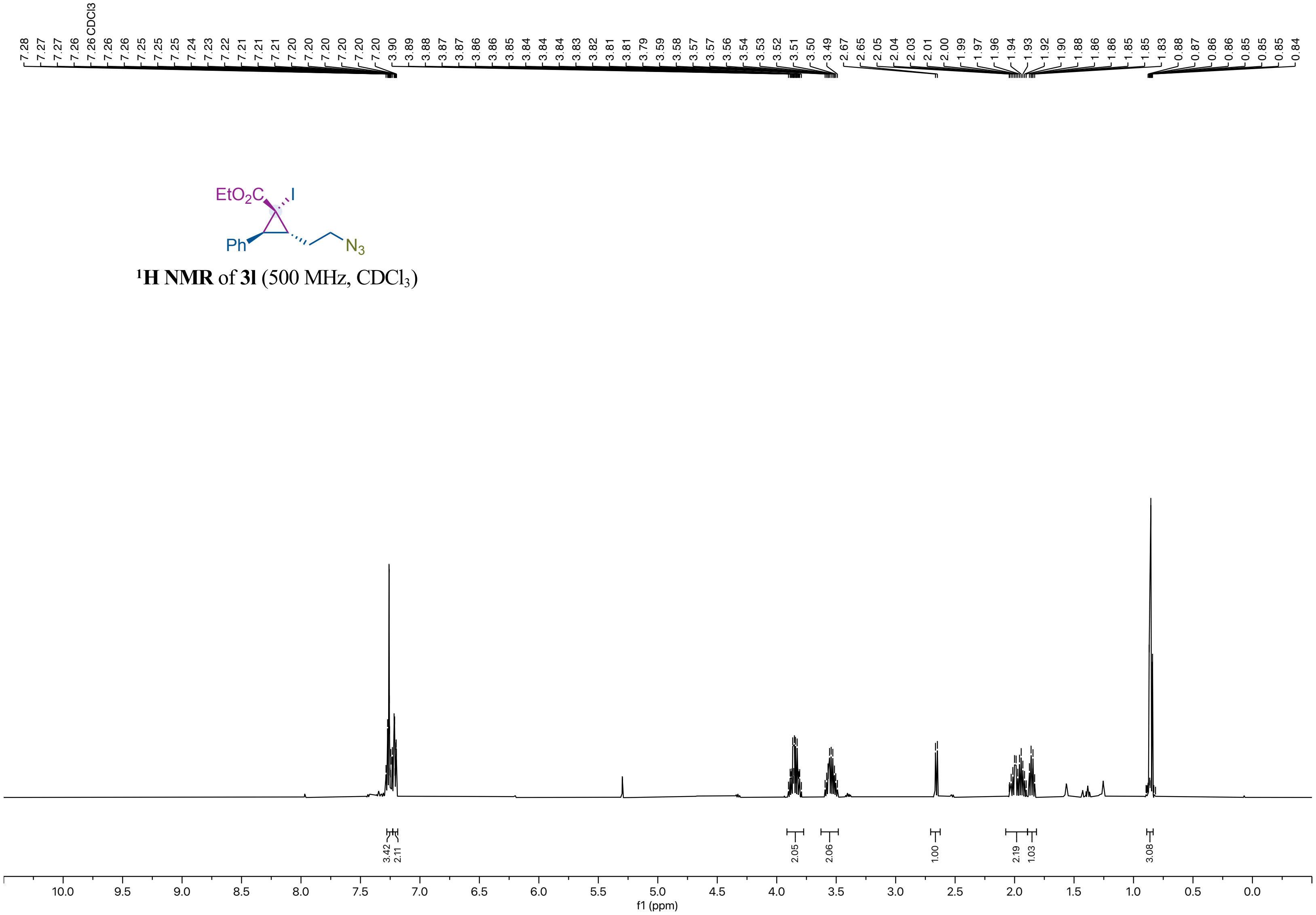

^{1}H NMR of **3l** (500 MHz, $CDCl_3$)

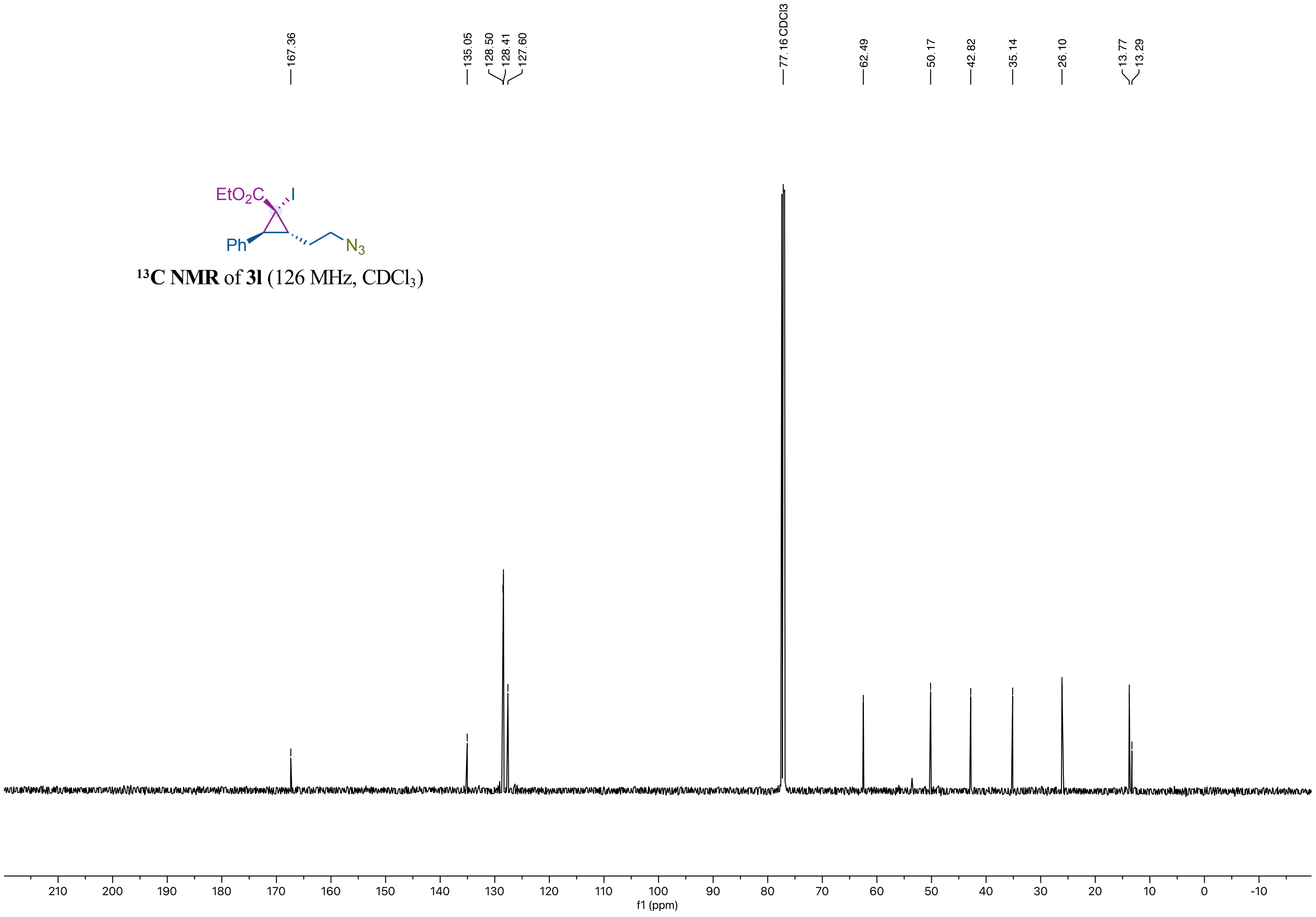

^{13}C NMR of **3l** (126 MHz, $CDCl_3$)

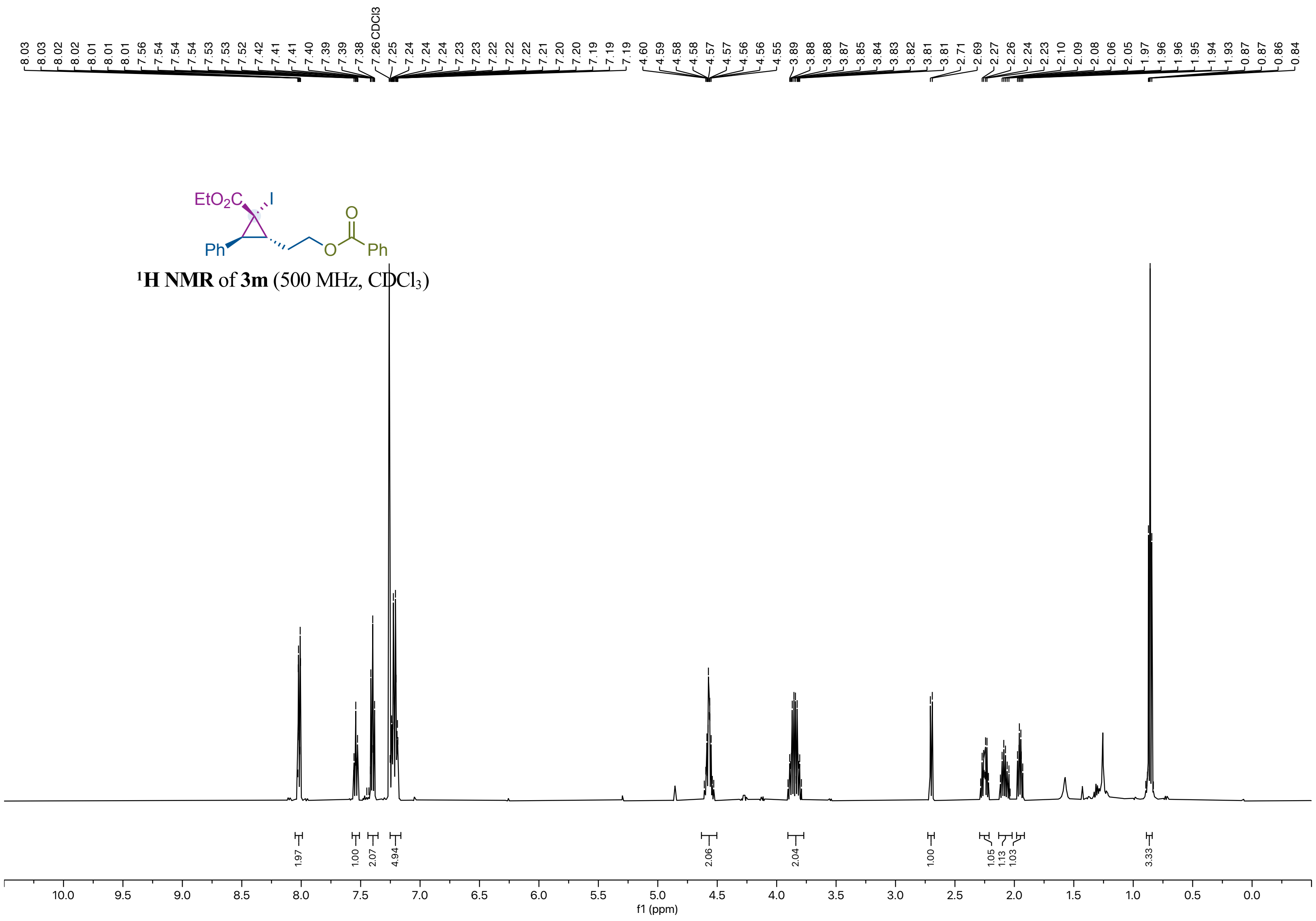

¹H NMR of **3m** (500 MHz, $CDCl_3$)

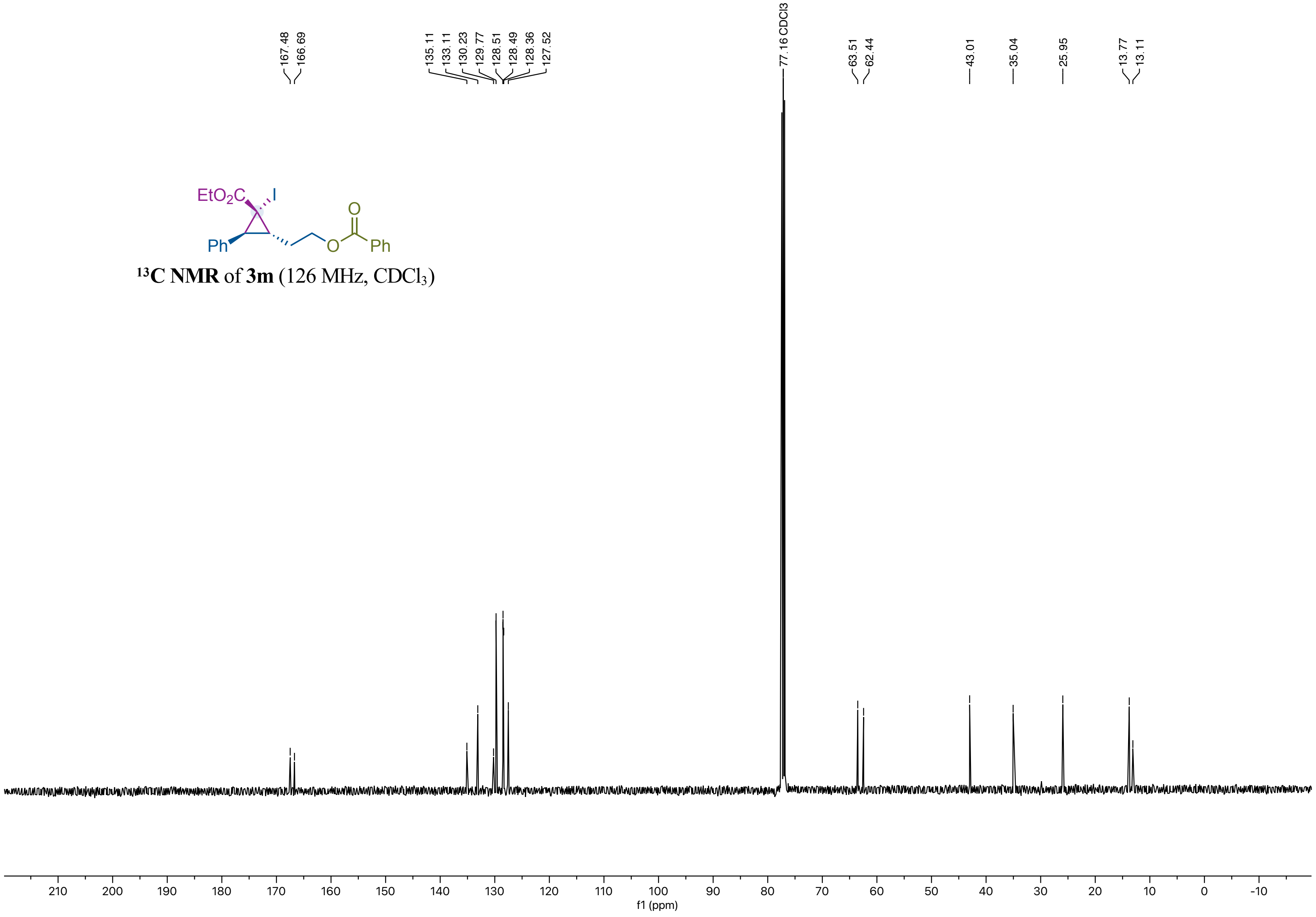

13C NMR of **3m** (126 MHz, $CDCl_3$)

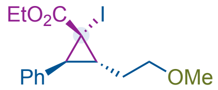

¹H NMR of **3n** (500 MHz, $CDCl_3$)

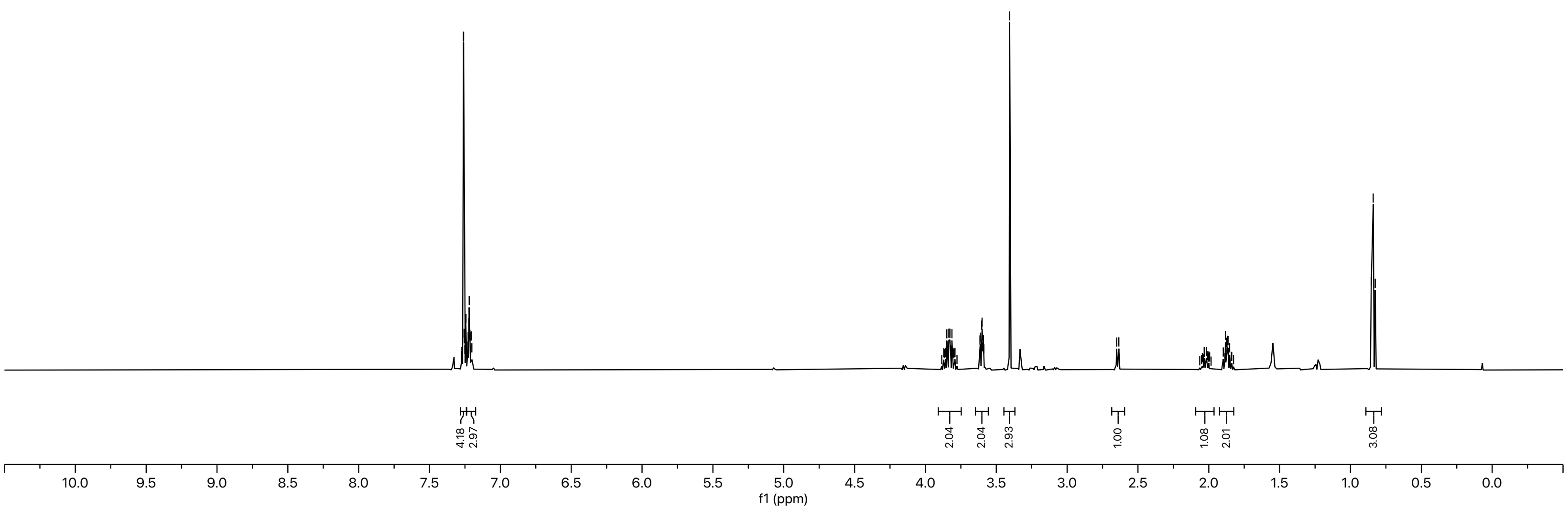

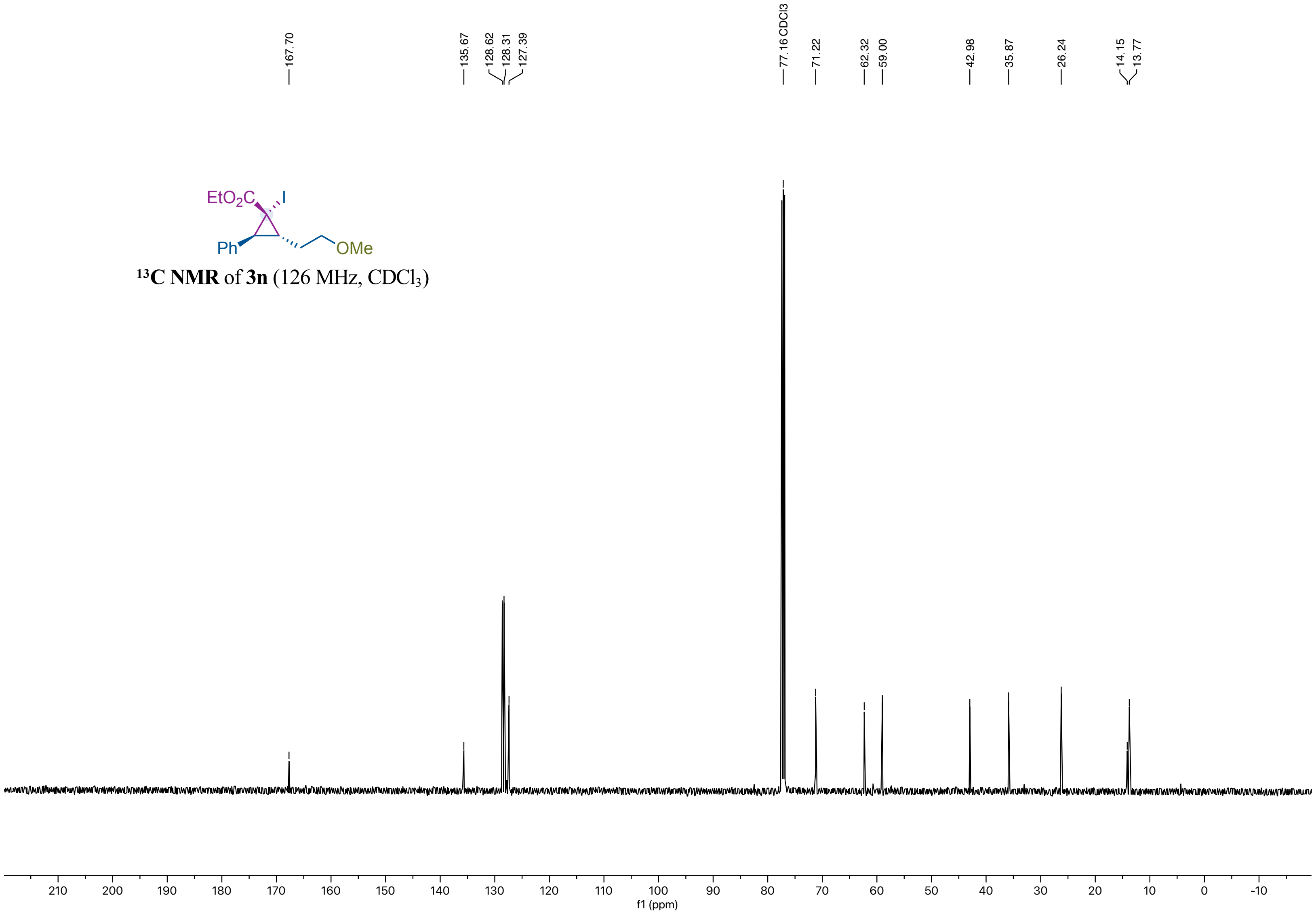

^{13}C NMR of **3n** (126 MHz, $CDCl_3$)

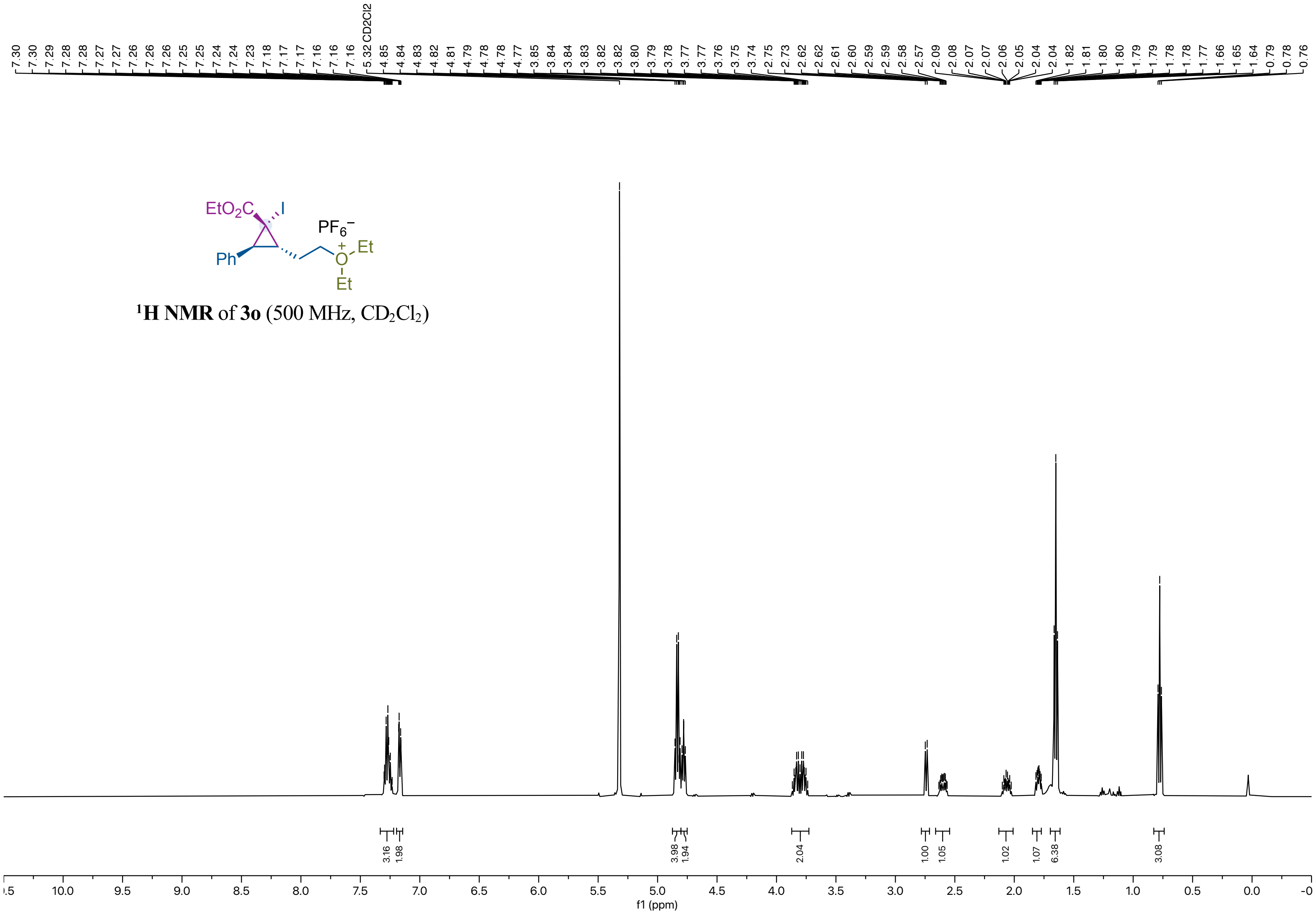

[1]**H NMR** of **3o** (500 MHz, CD_2Cl_2)

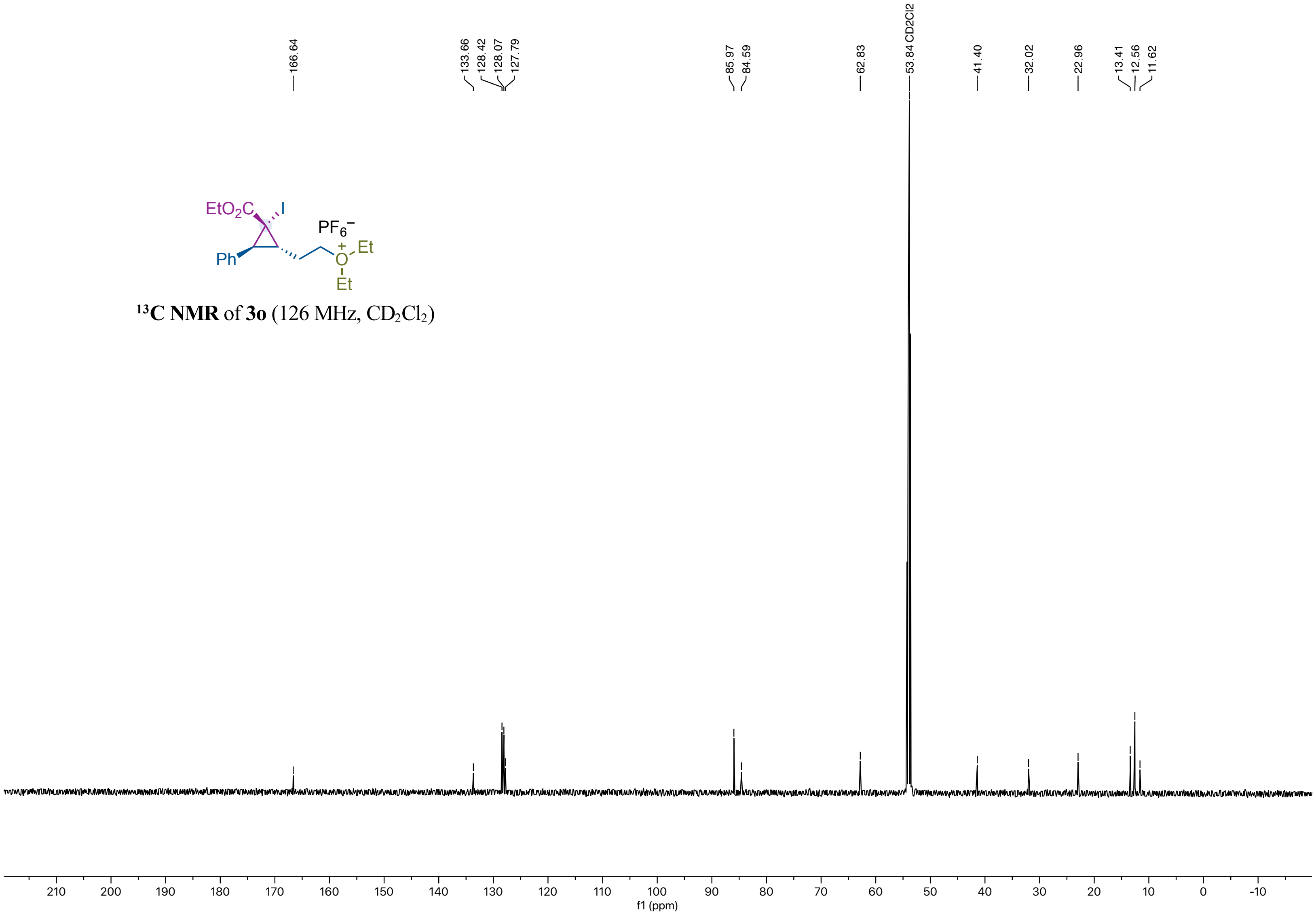

^{13}C NMR of **3o** (126 MHz, CD_2Cl_2)

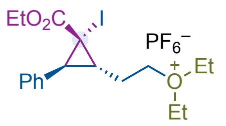

^{31}P NMR of **3o** (202 MHz, CD_2Cl_2)

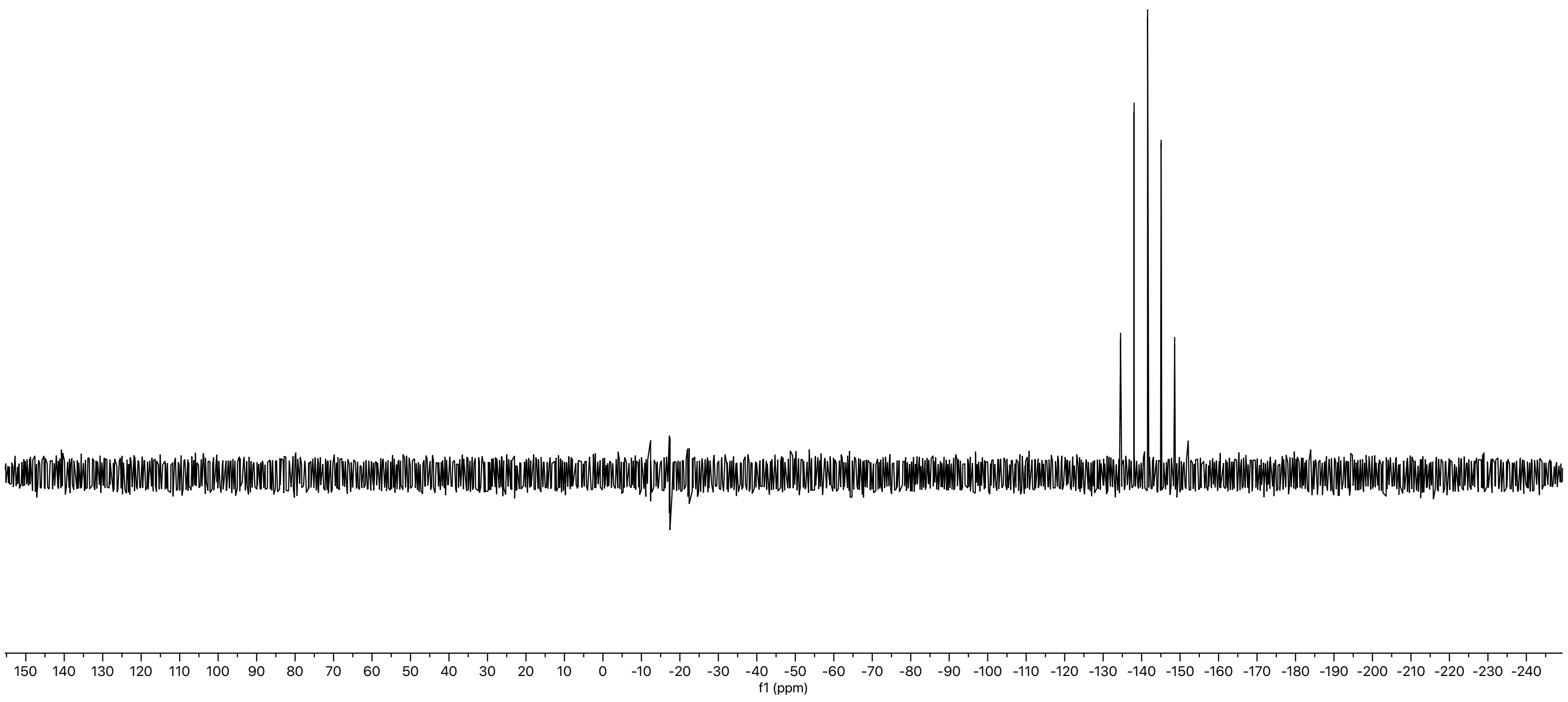

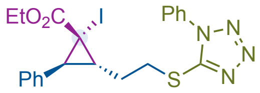

¹H NMR of **3p** (400 MHz, $CDCl_3$)

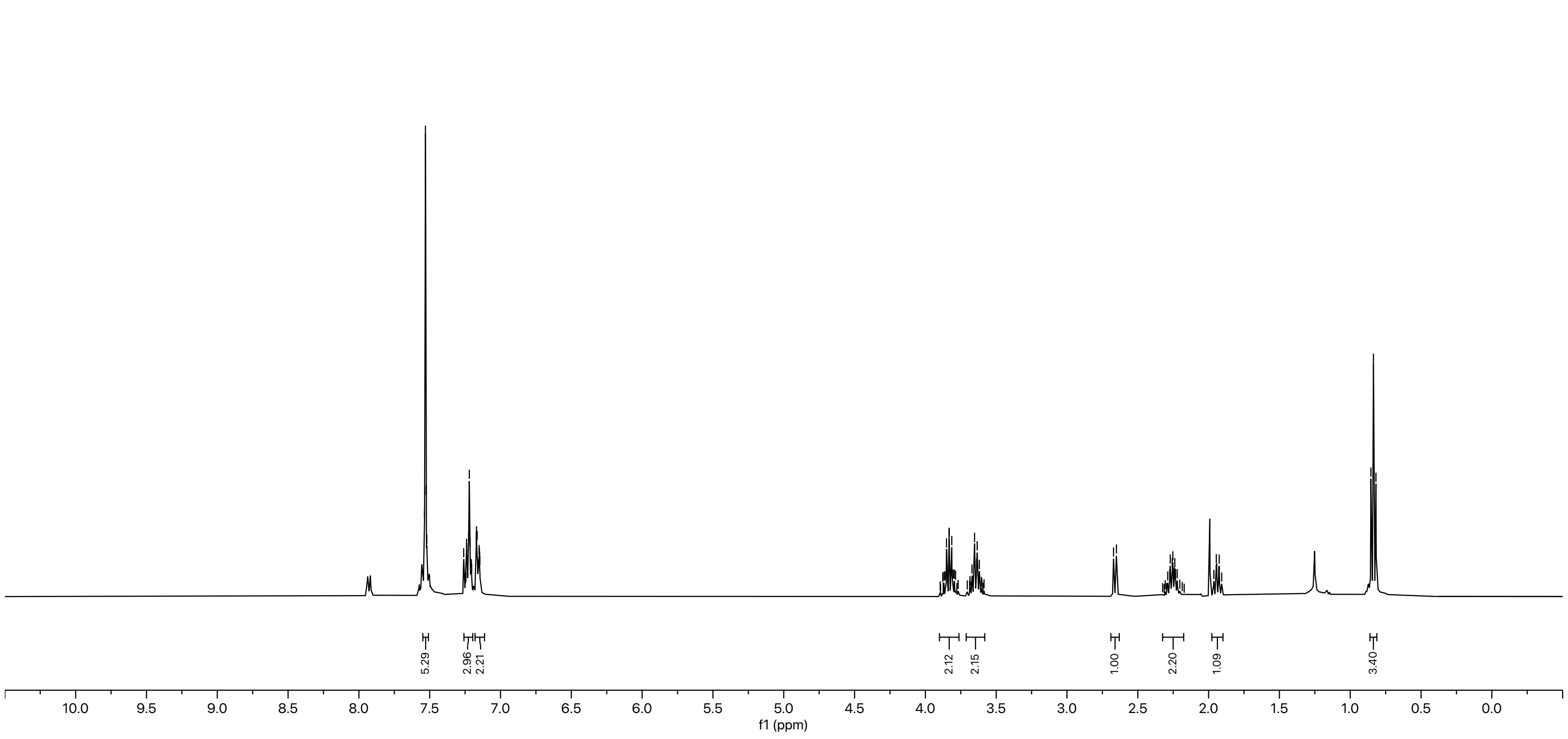

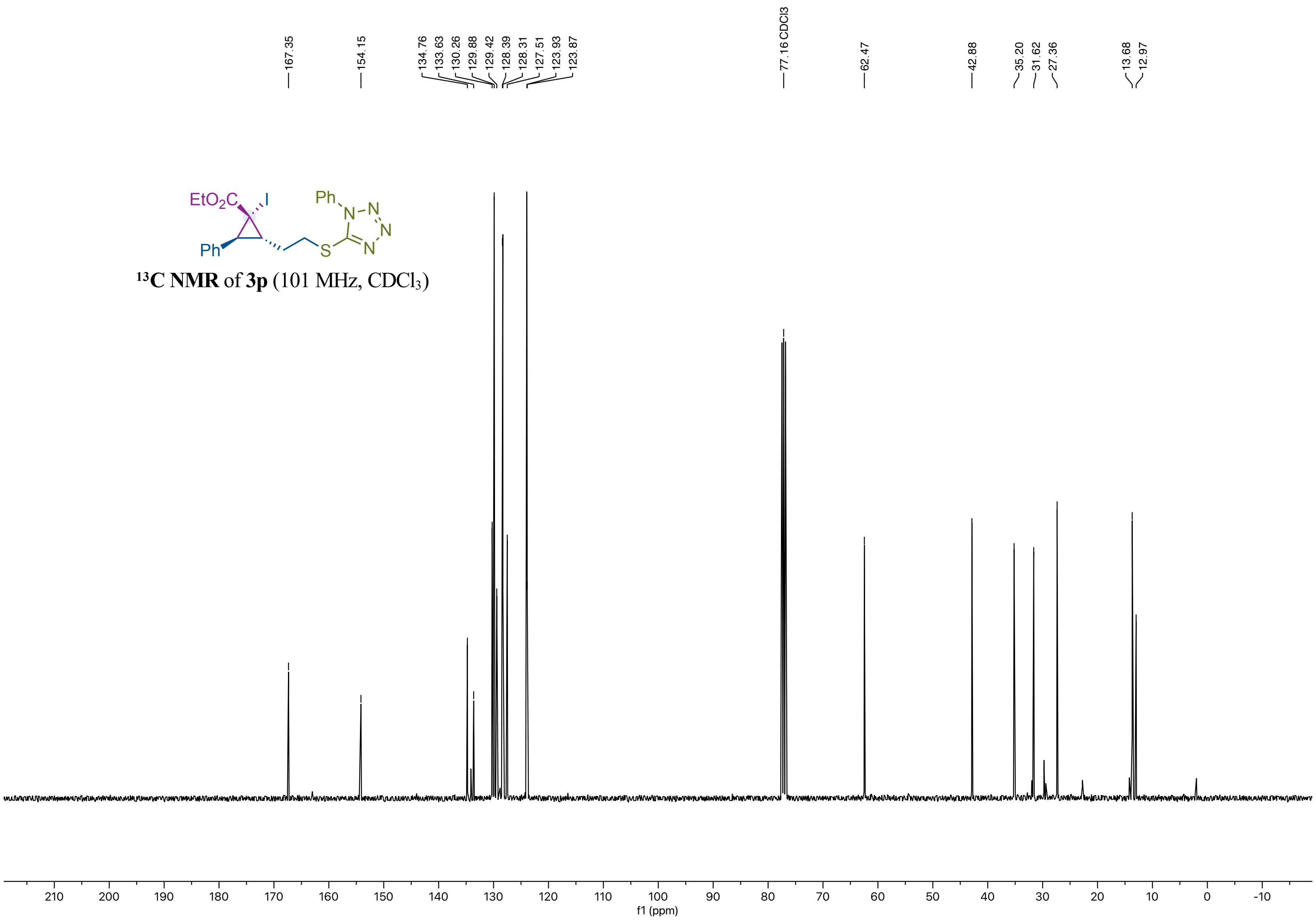

^{13}C NMR of **3p** (101 MHz, $CDCl_3$)

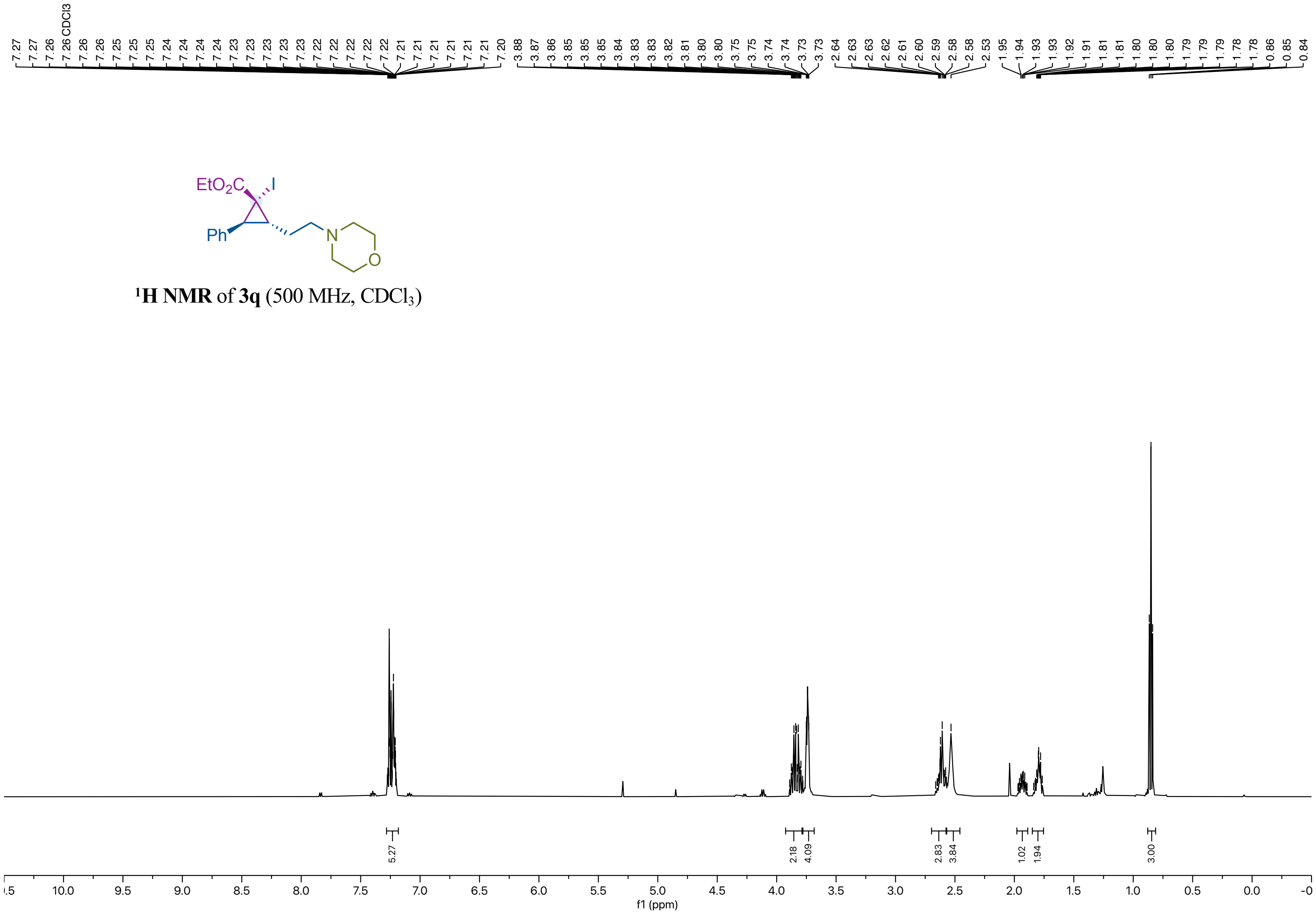

¹H NMR of **3q** (500 MHz, $CDCl_3$)

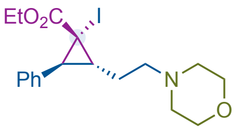

^{13}C NMR of **3q** (126 MHz, $CDCl_3$)

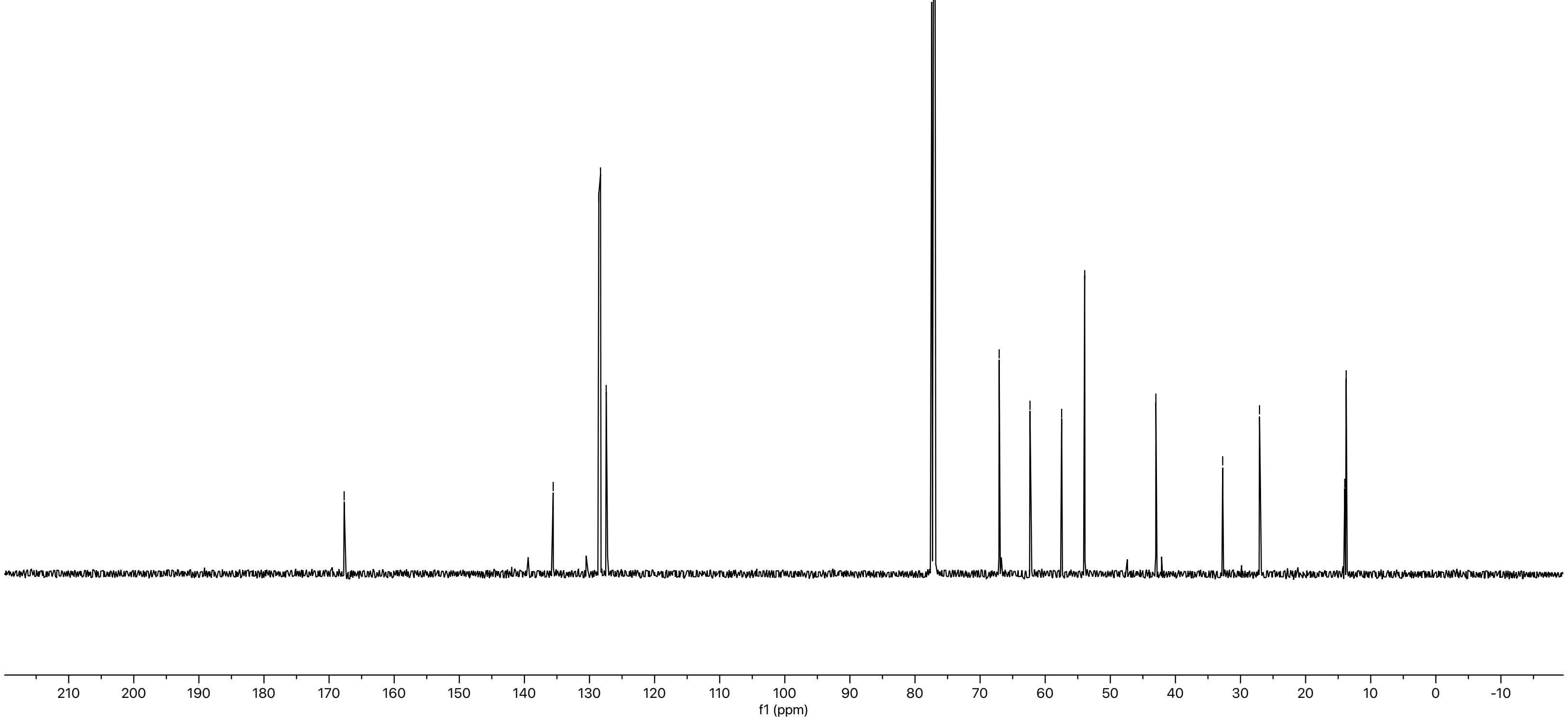

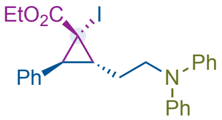

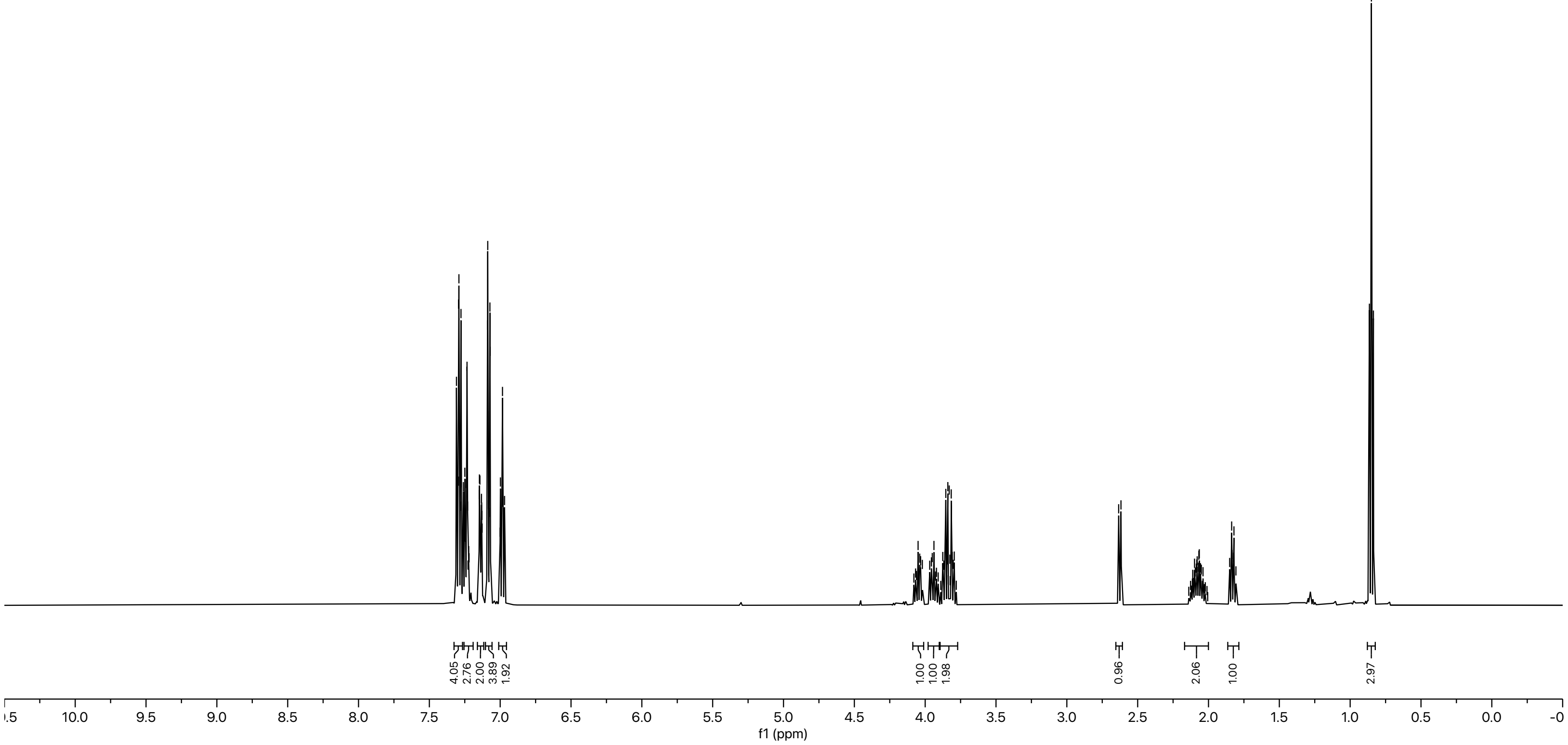

^{1}H NMR of **3r** (500 MHz, $CDCl_3$)

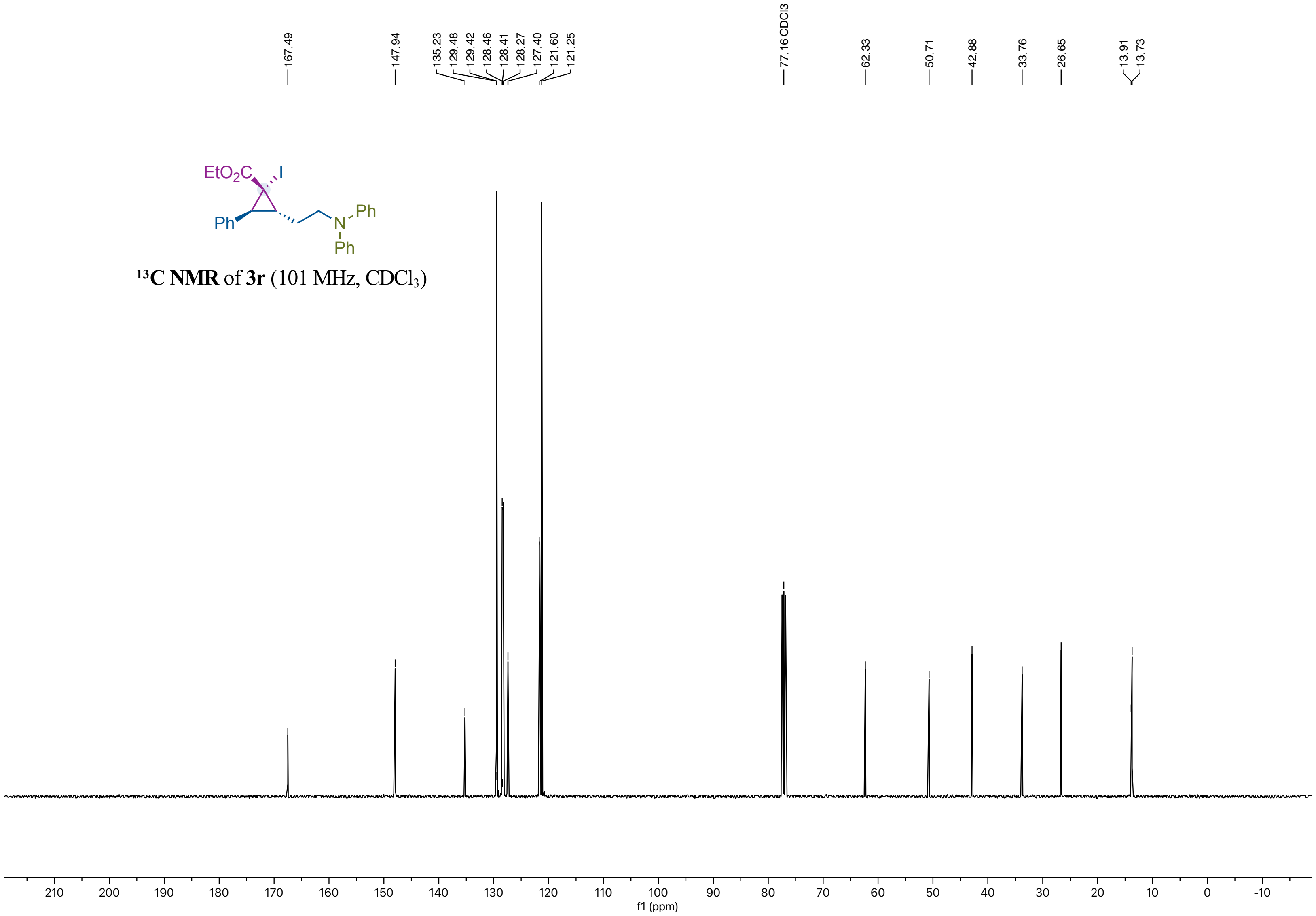

^{13}C NMR of **3r** (101 MHz, $CDCl_3$)

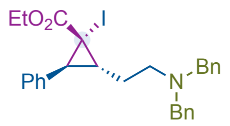

¹H NMR of **3s** (400 MHz, $CDCl_3$)

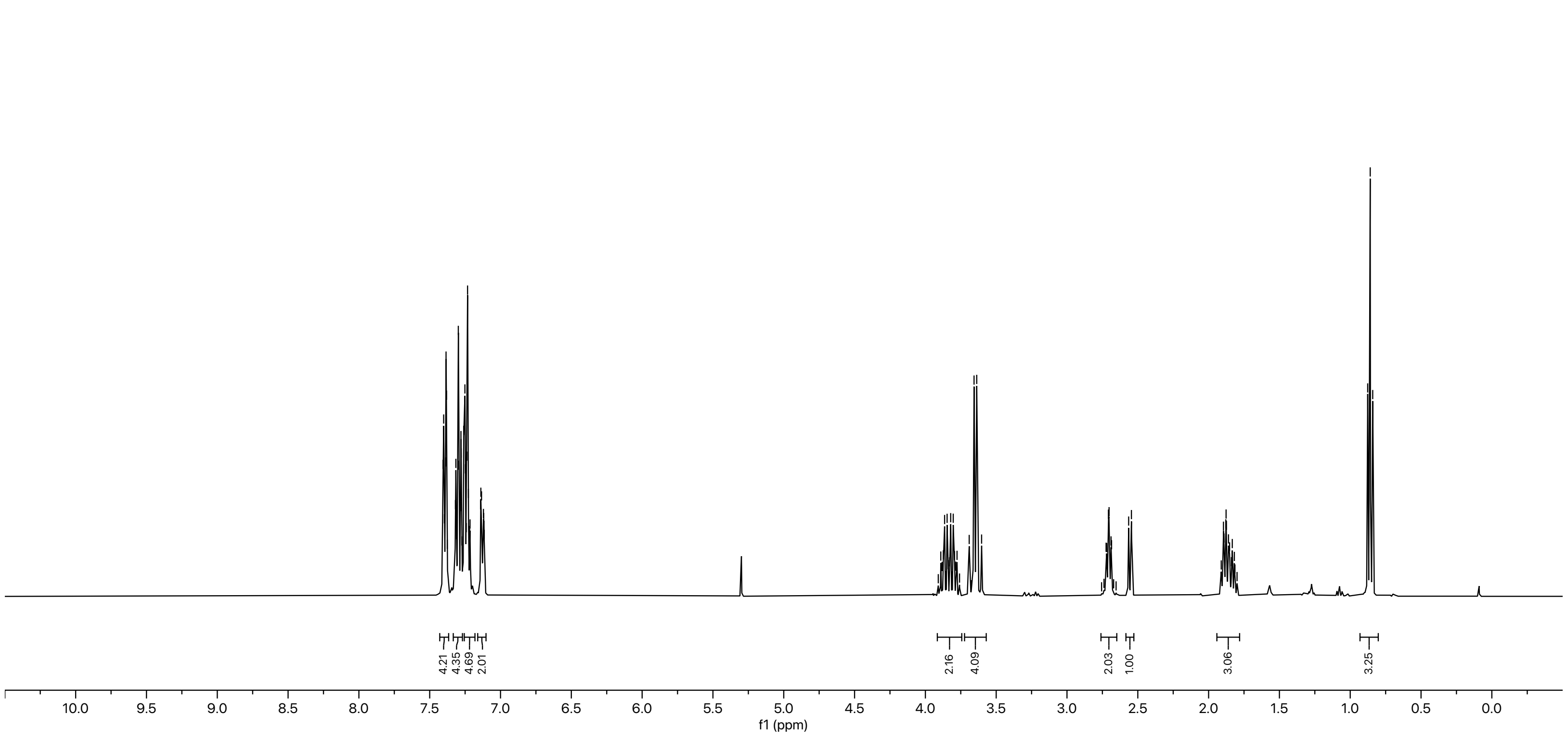

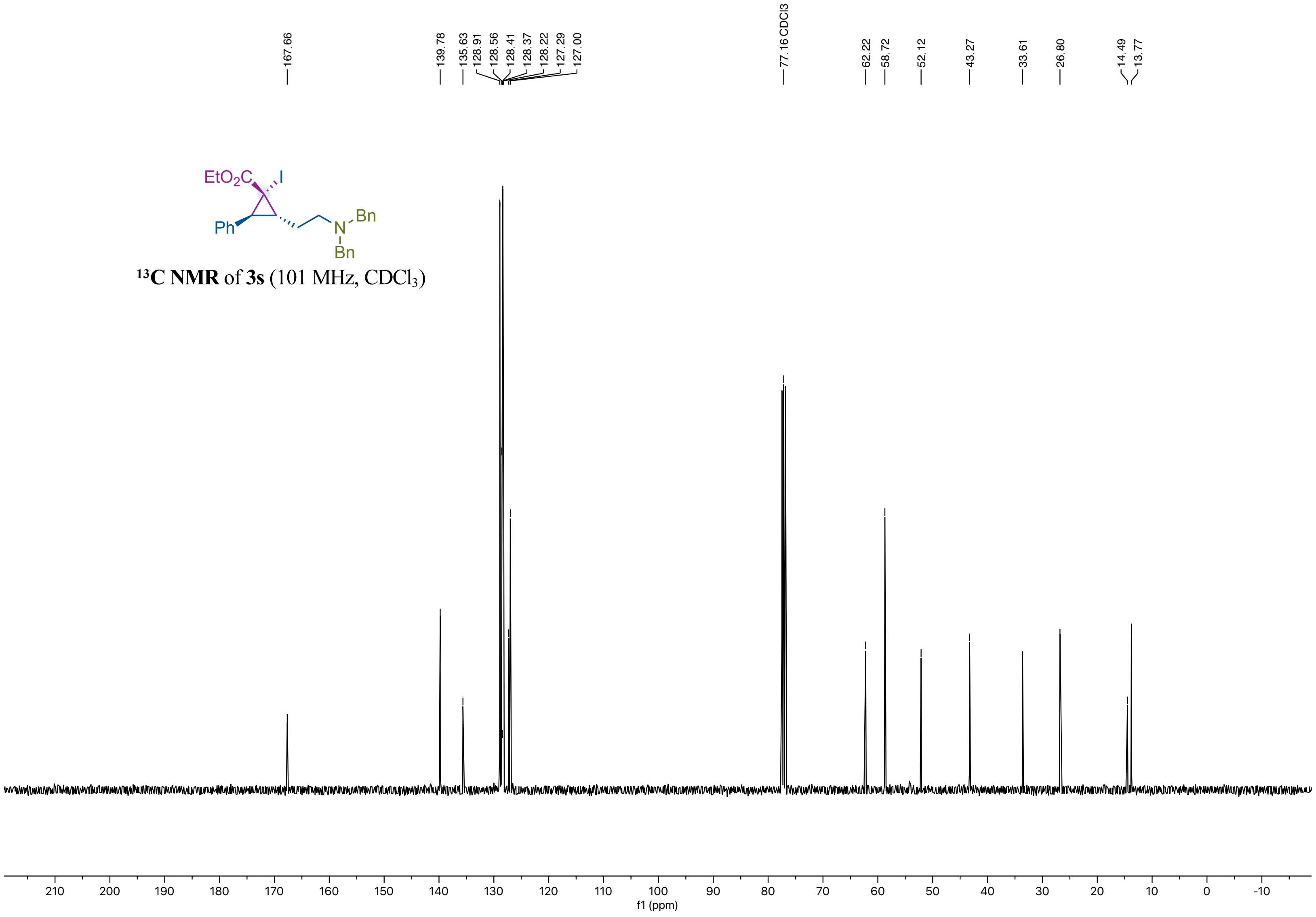

^{13}C NMR of **3s** (101 MHz, $CDCl_3$)

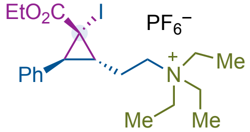

¹H NMR of **3t** (400 MHz, CDCl$_3$)

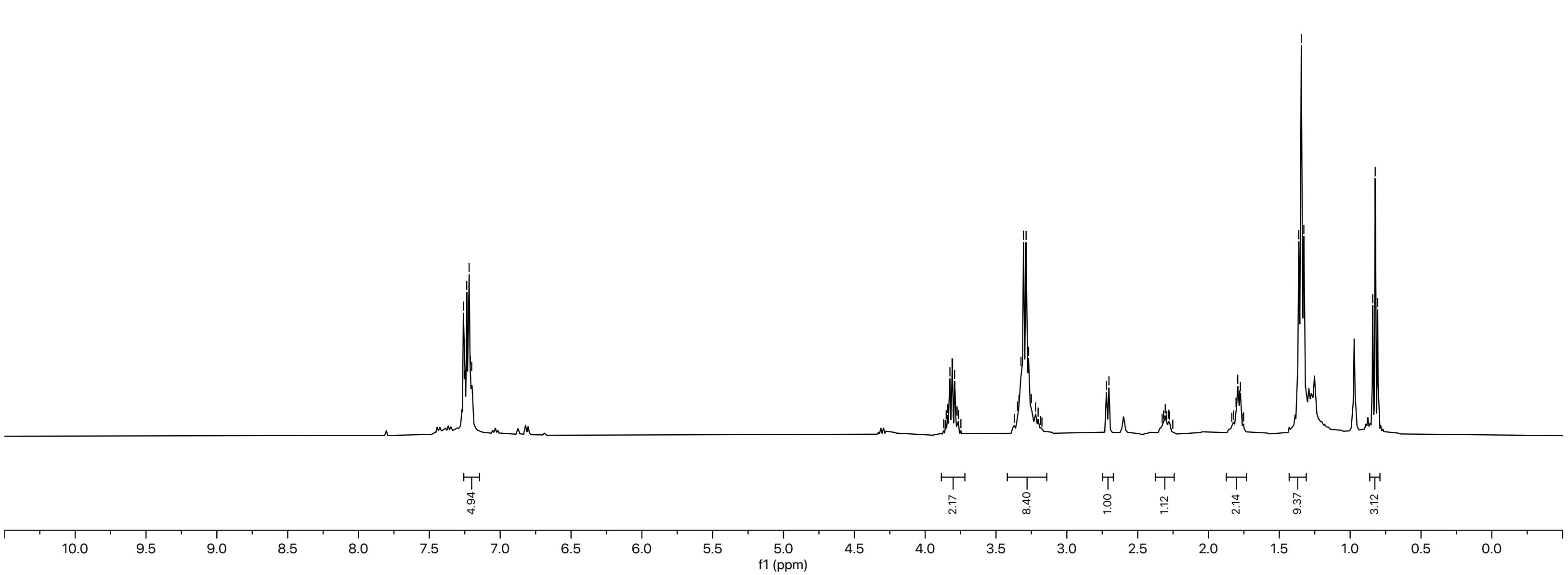

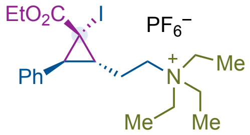

^{13}**C NMR** of **3t** (101 MHz, $CDCl_3$)

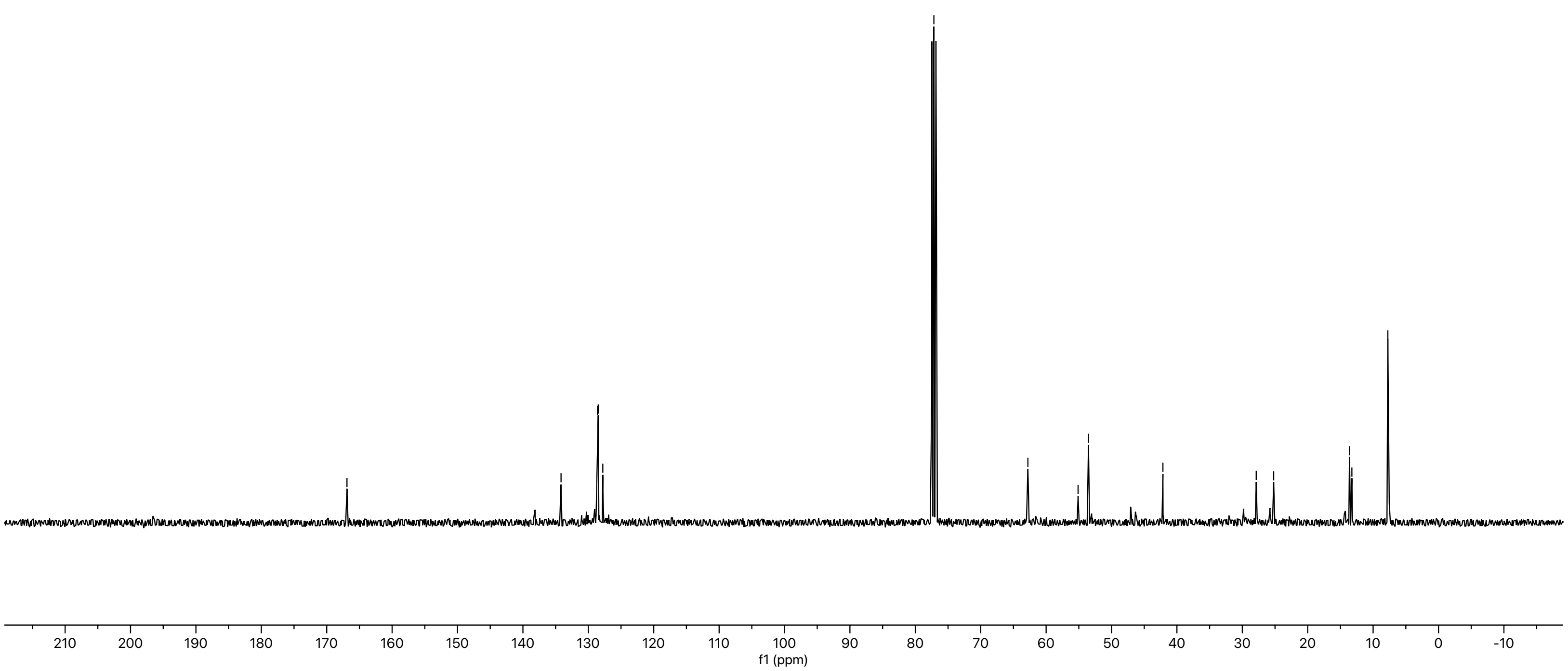

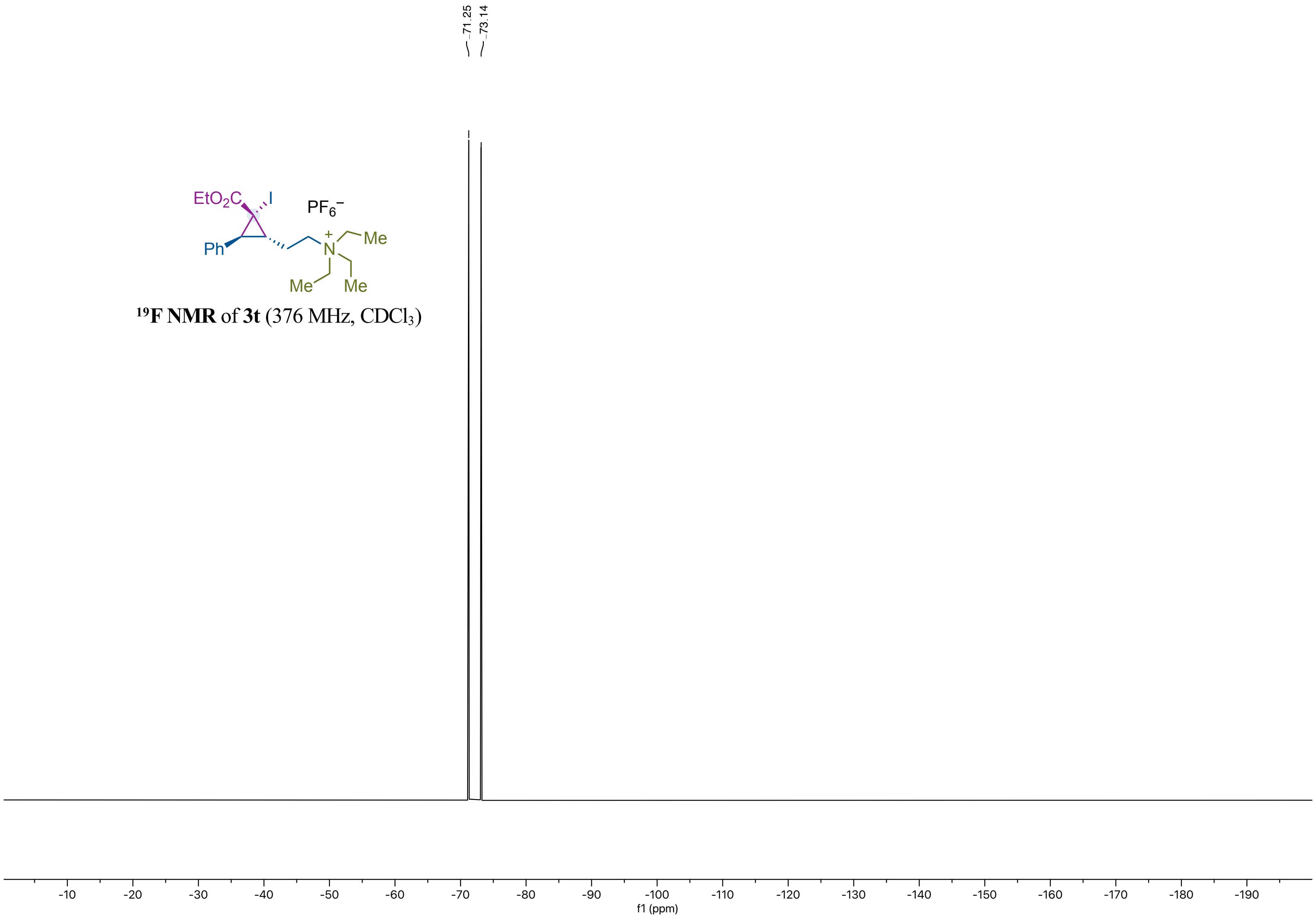

^{19}F NMR of **3t** (376 MHz, $CDCl_3$)

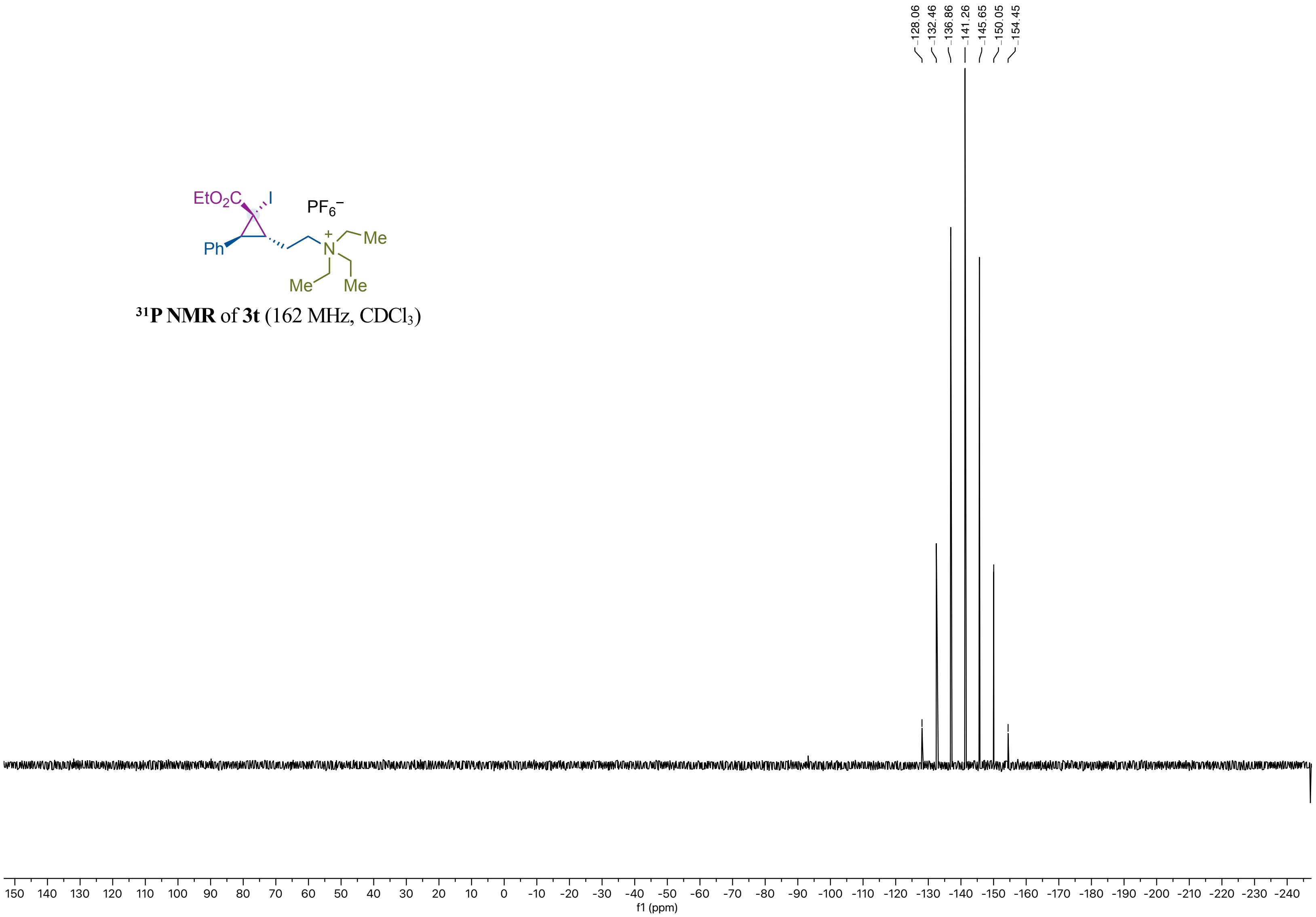

^{31}P NMR of **3t** (162 MHz, $CDCl_3$)

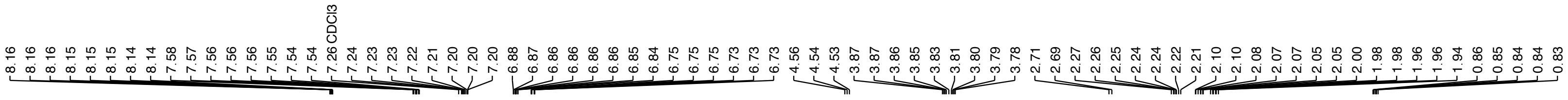

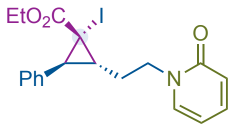

^{1}H NMR of **3u** (400 MHz, $CDCl_3$)

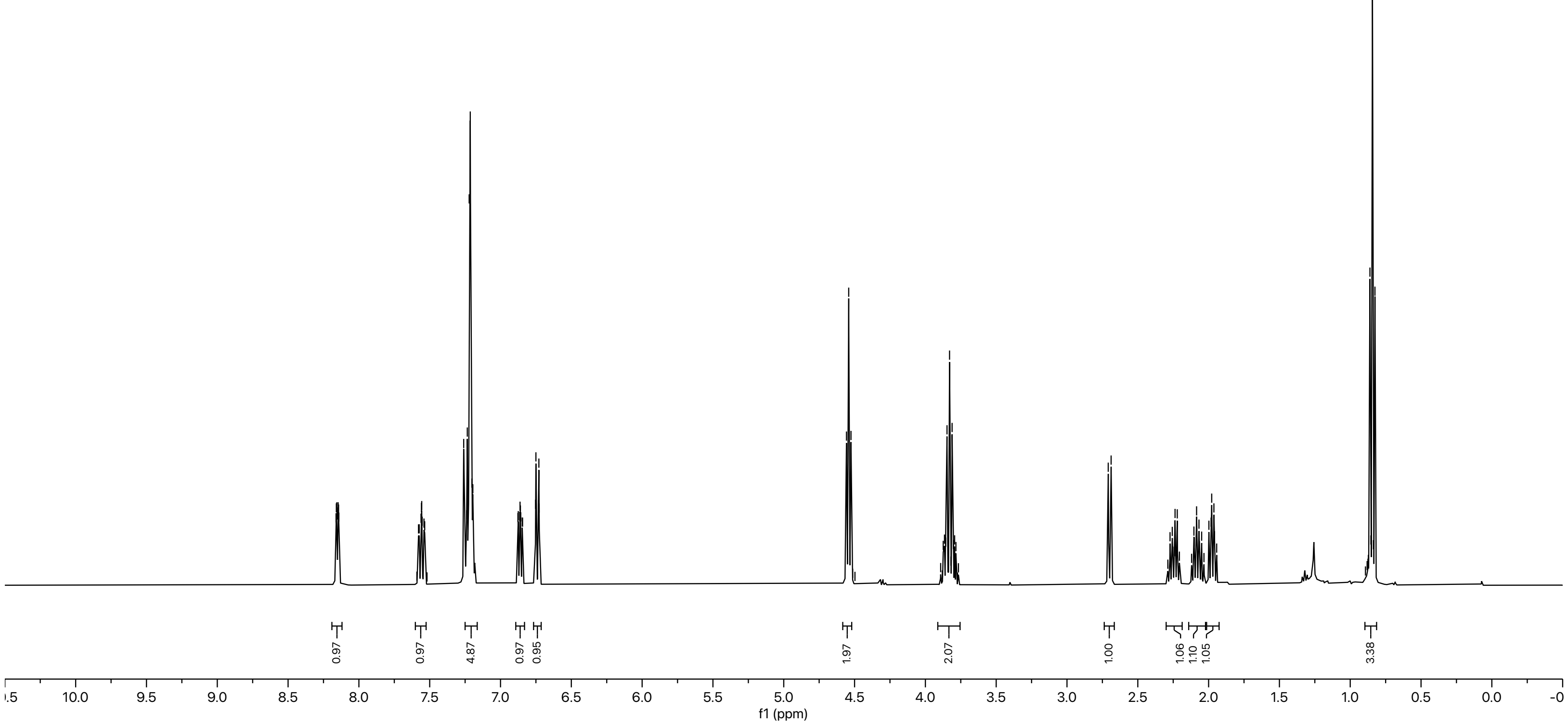

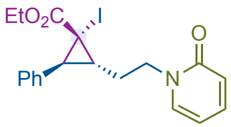

^{13}C NMR of **3u** (101 MHz, $CDCl_3$)

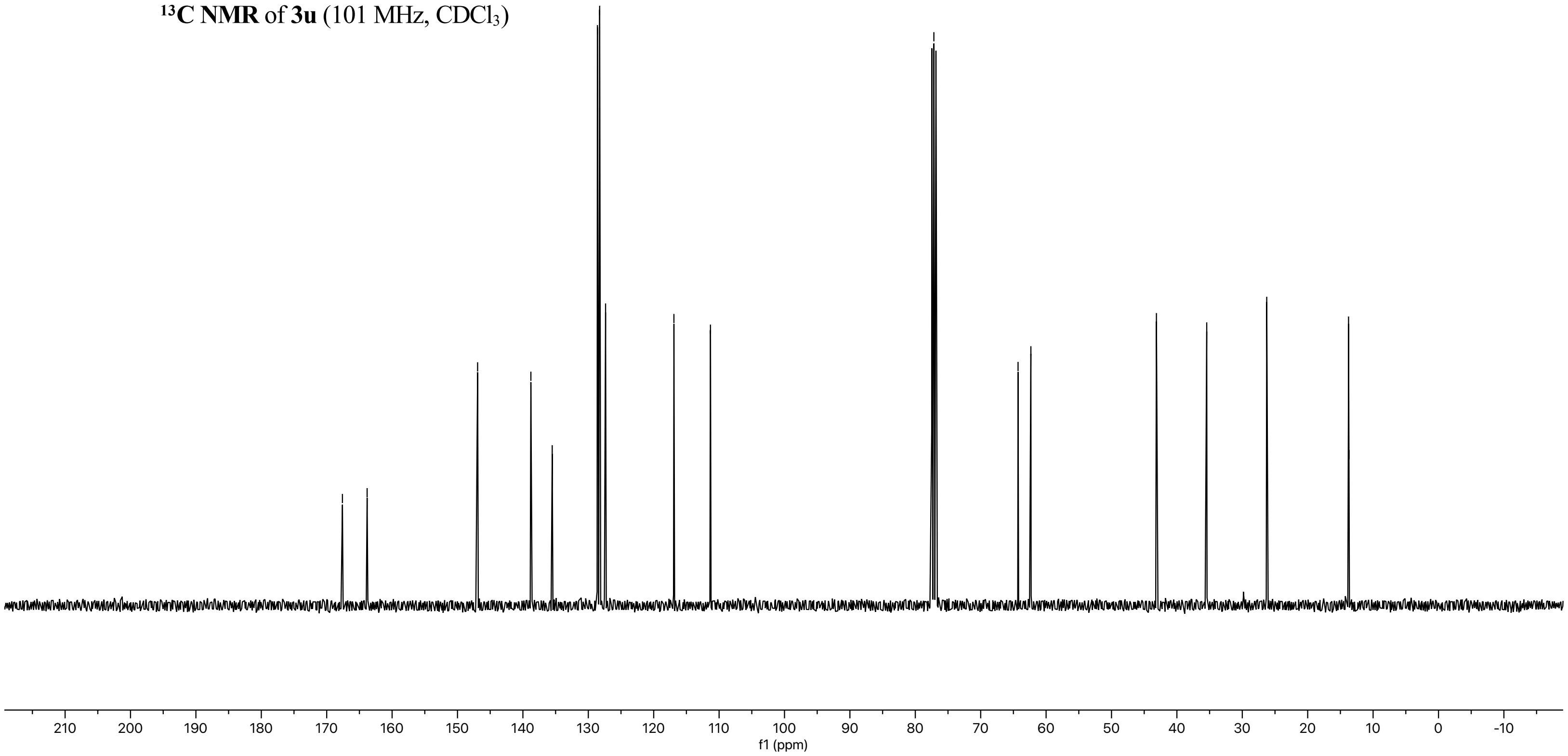

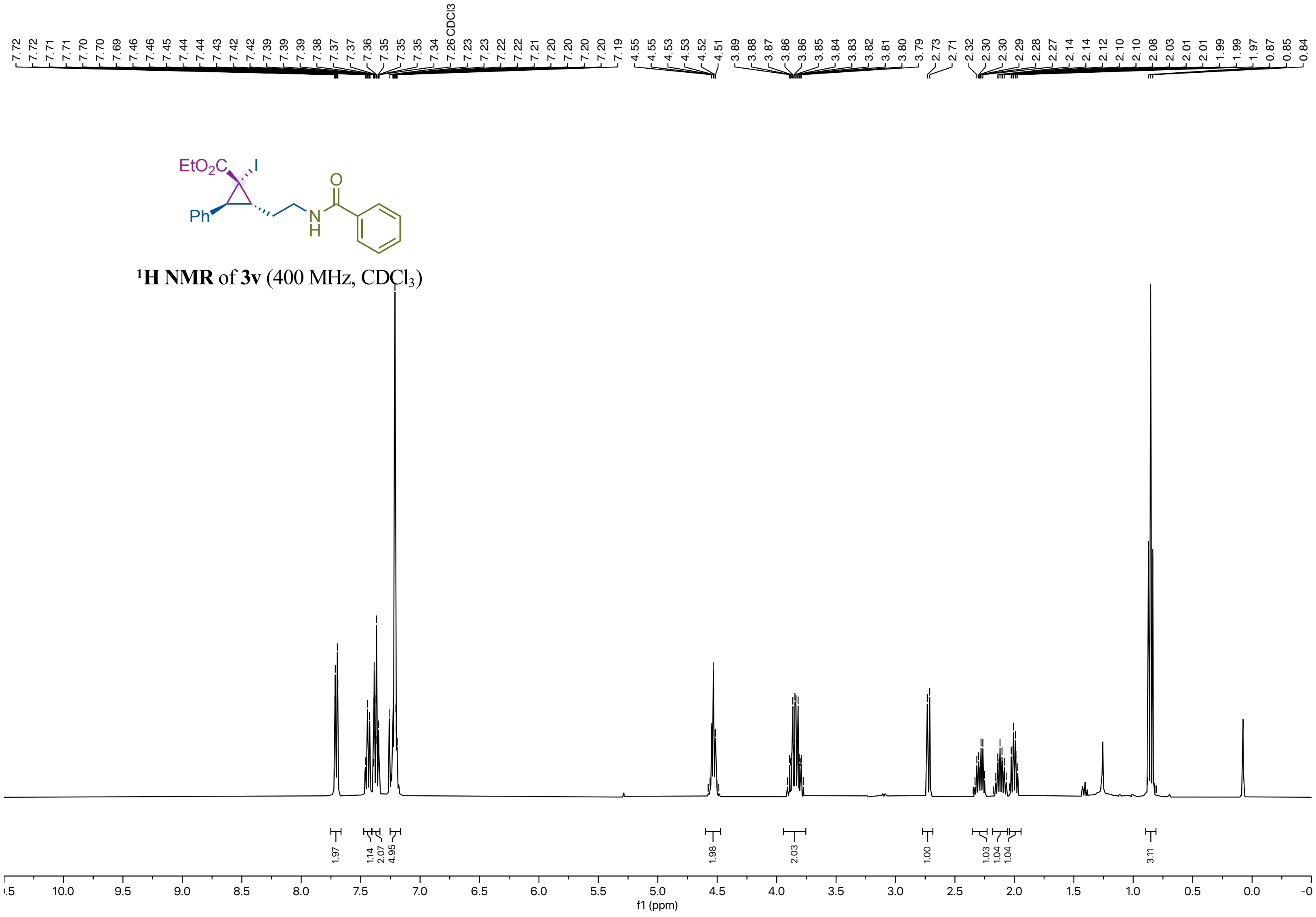

¹H NMR of **3v** (400 MHz, $CDCl_3$)

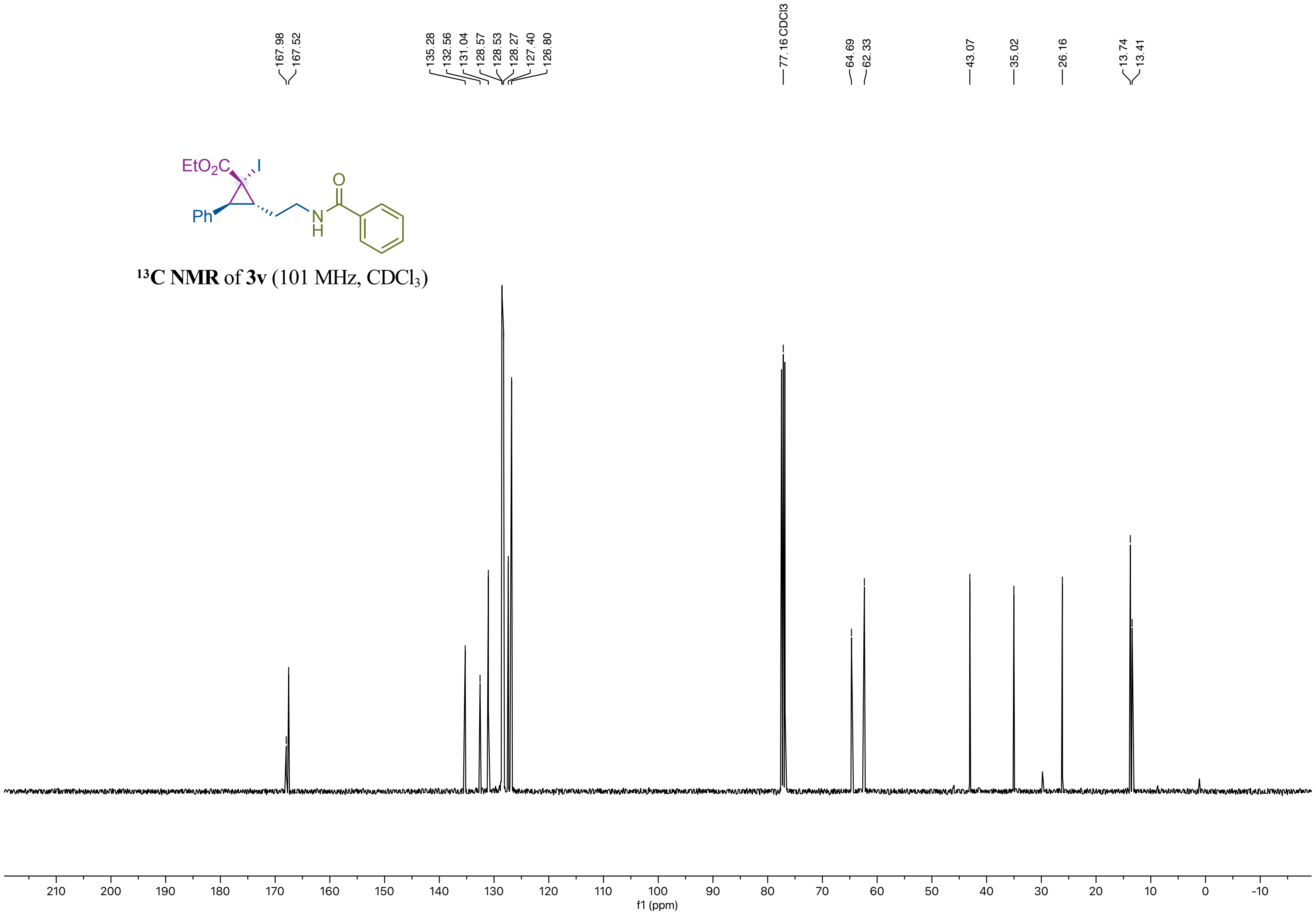

^{13}C NMR of **3v** (101 MHz, $CDCl_3$)

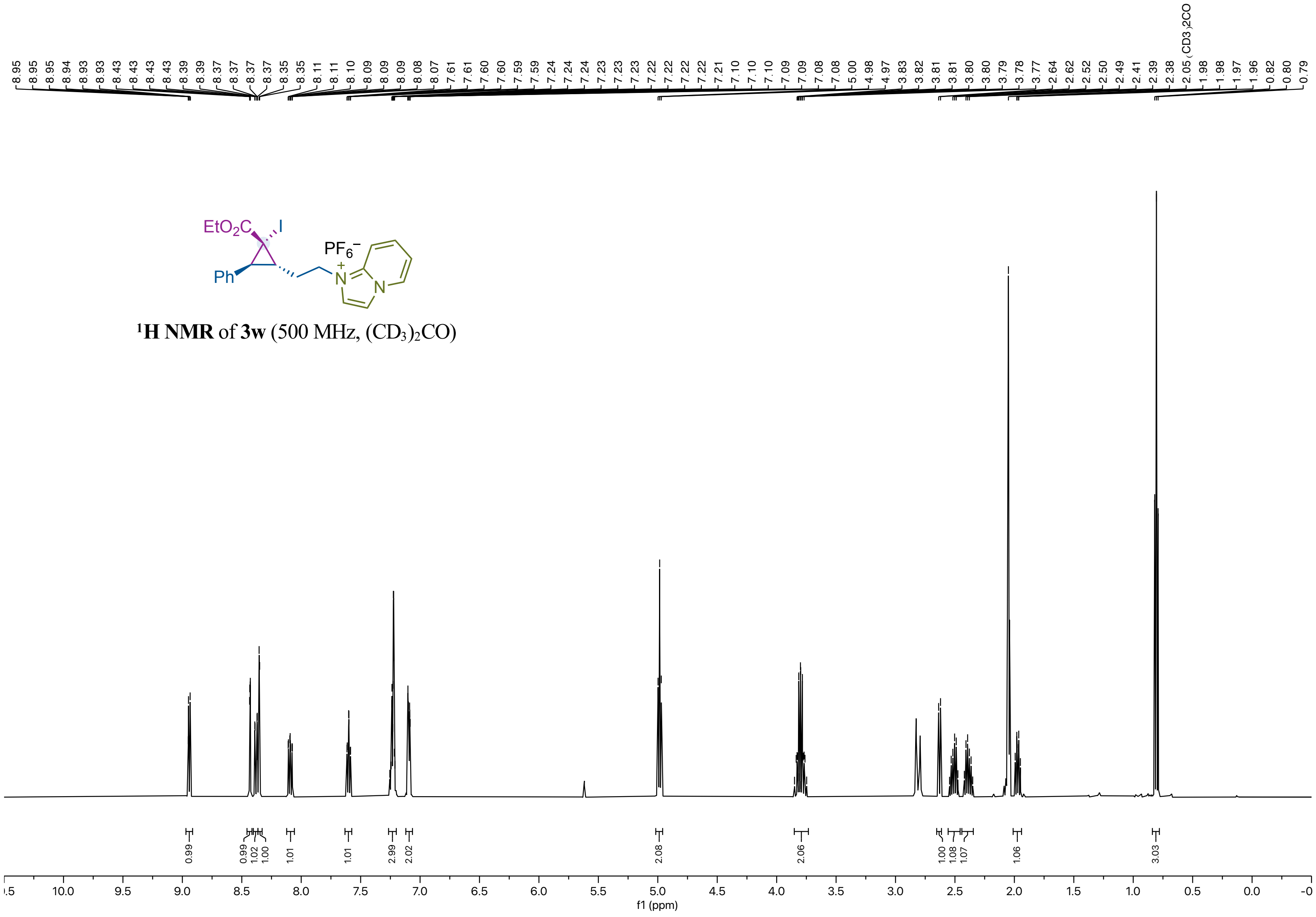

^{1}**H NMR** of **3w** (500 MHz, $(CD_3)_2CO$)

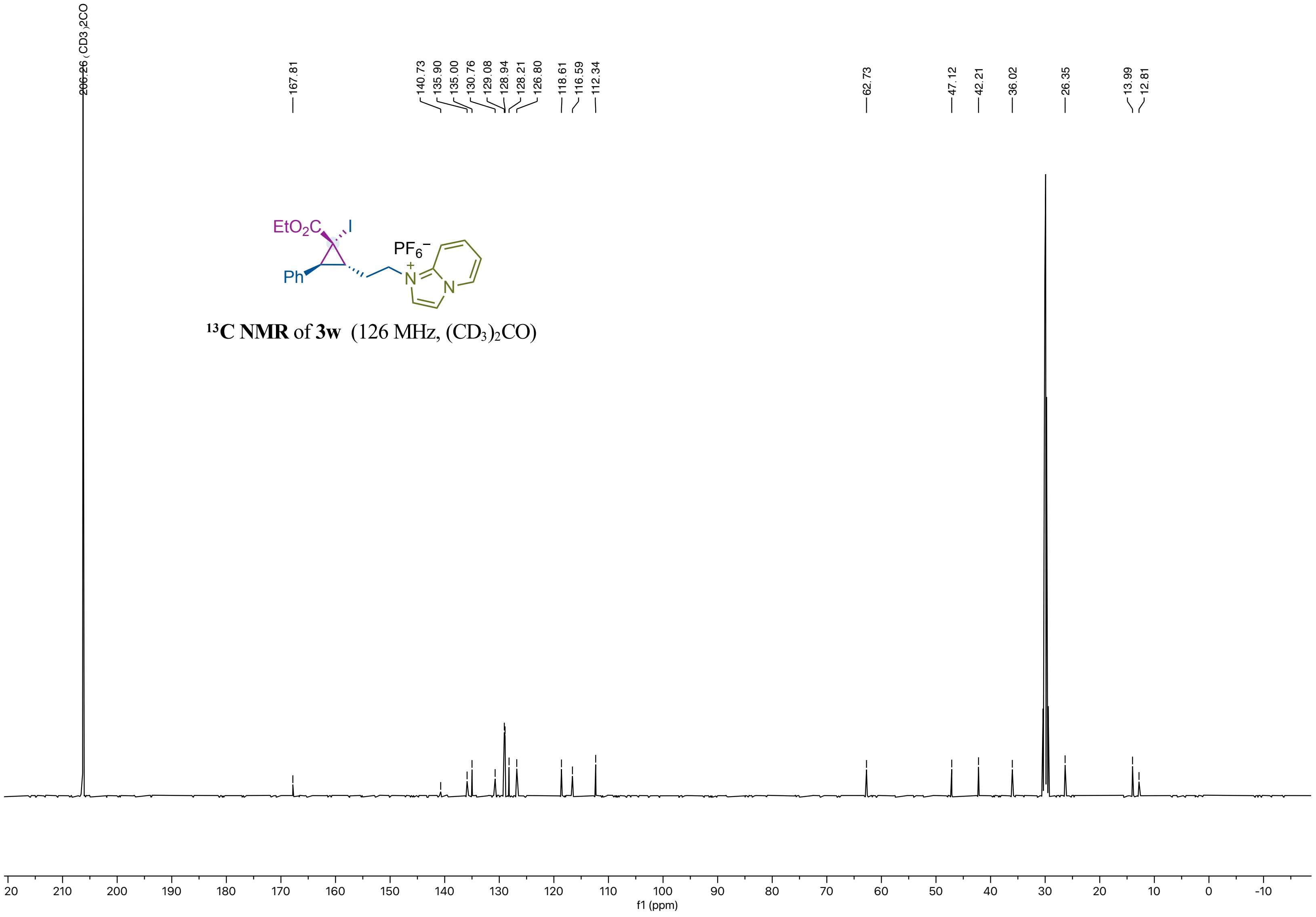
206.26 (CD3)2CO
167.81
140.73
135.90
135.00
130.76
129.08
128.94
128.21
126.80
118.61
116.59
112.34
62.73
47.12
42.21
36.02
26.35
13.99
12.81
EtO2C
I
Ph
PF6−
N
N
13C NMR of 3w (126 MHz, (CD3)2CO)
f1 (ppm)

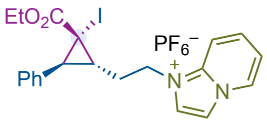

^{31}P NMR of **3w** (162 MHz, $CDCl_3$)

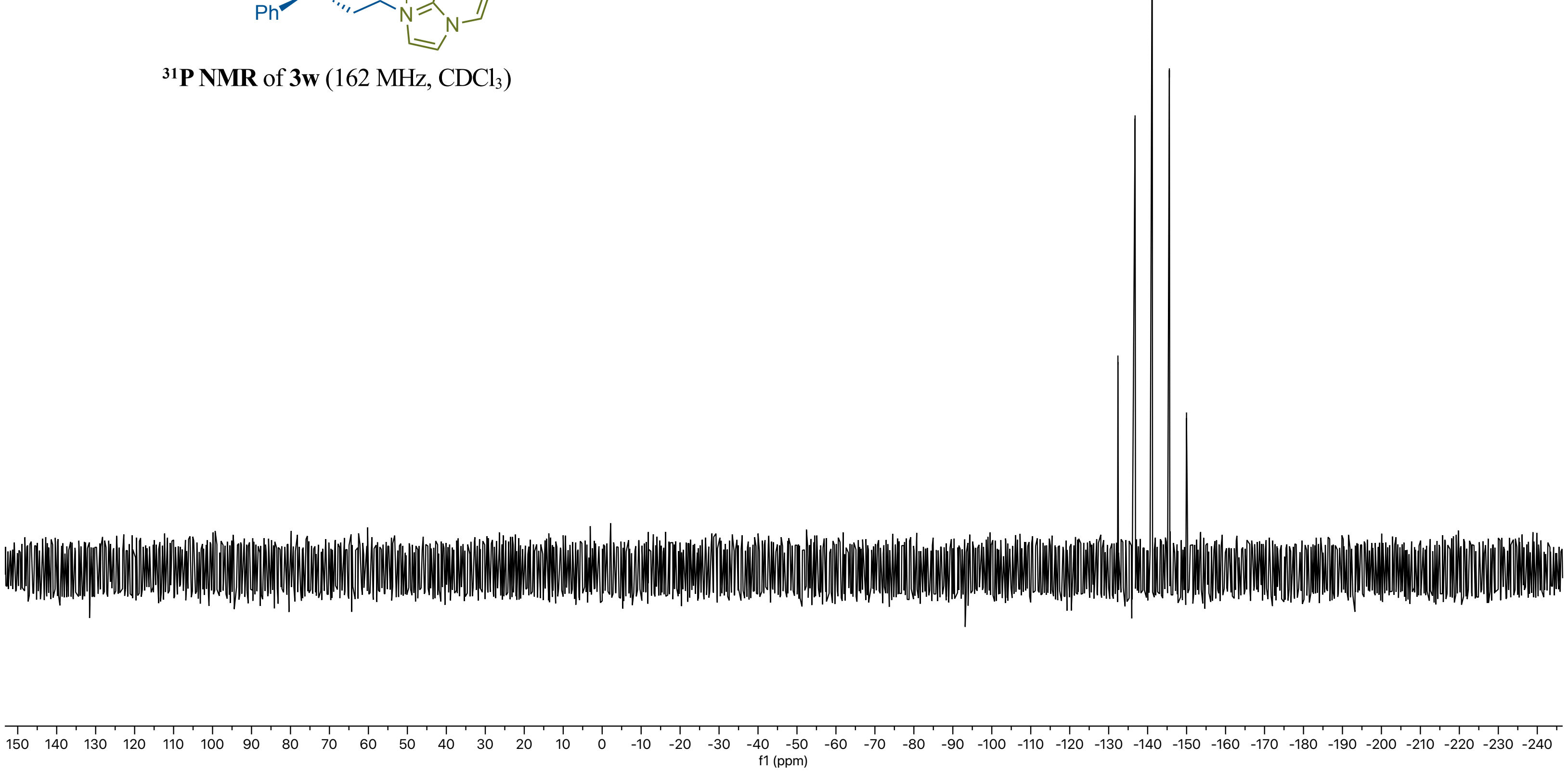

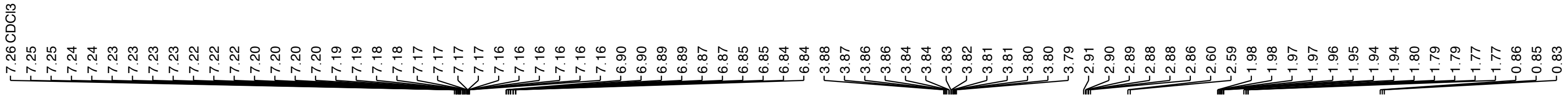

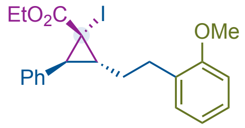

^{1}H NMR of **3x** (500 MHz, $CDCl_3$)

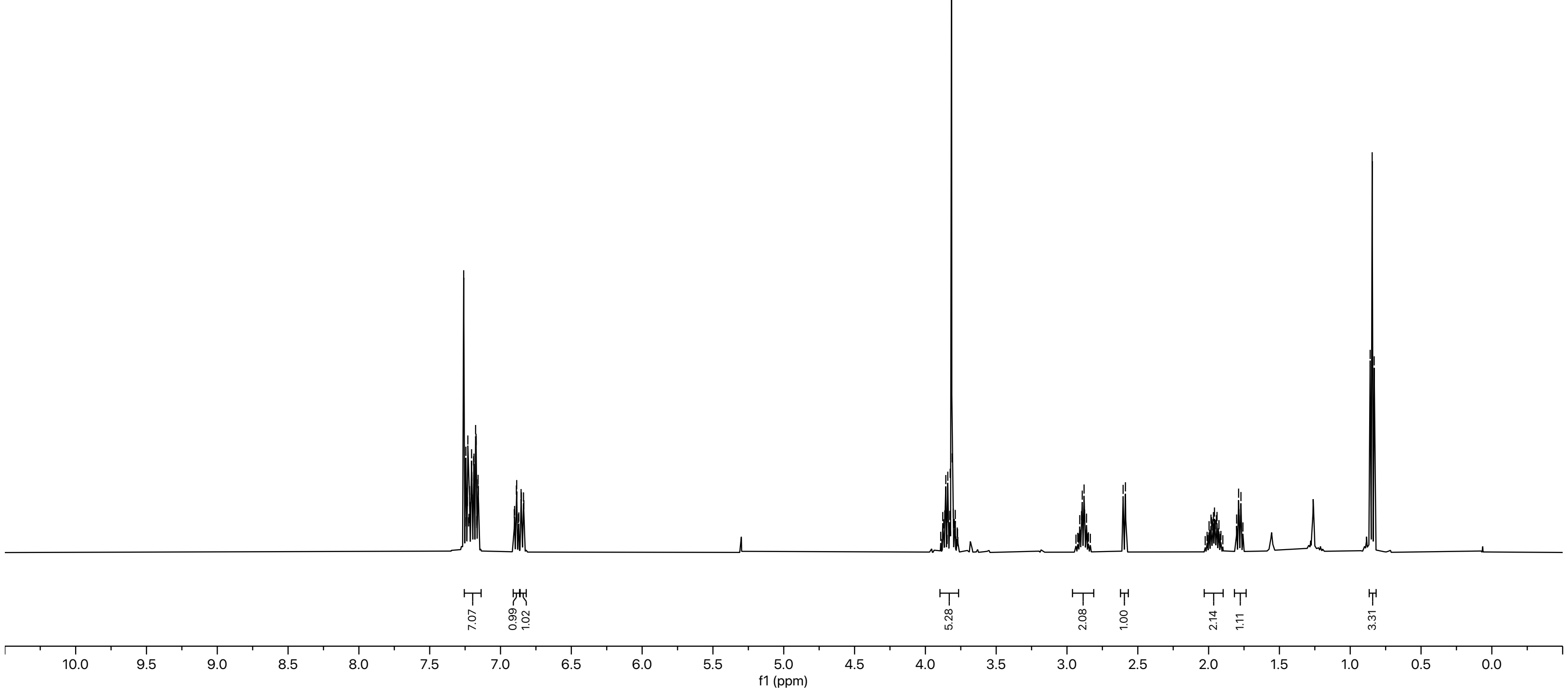

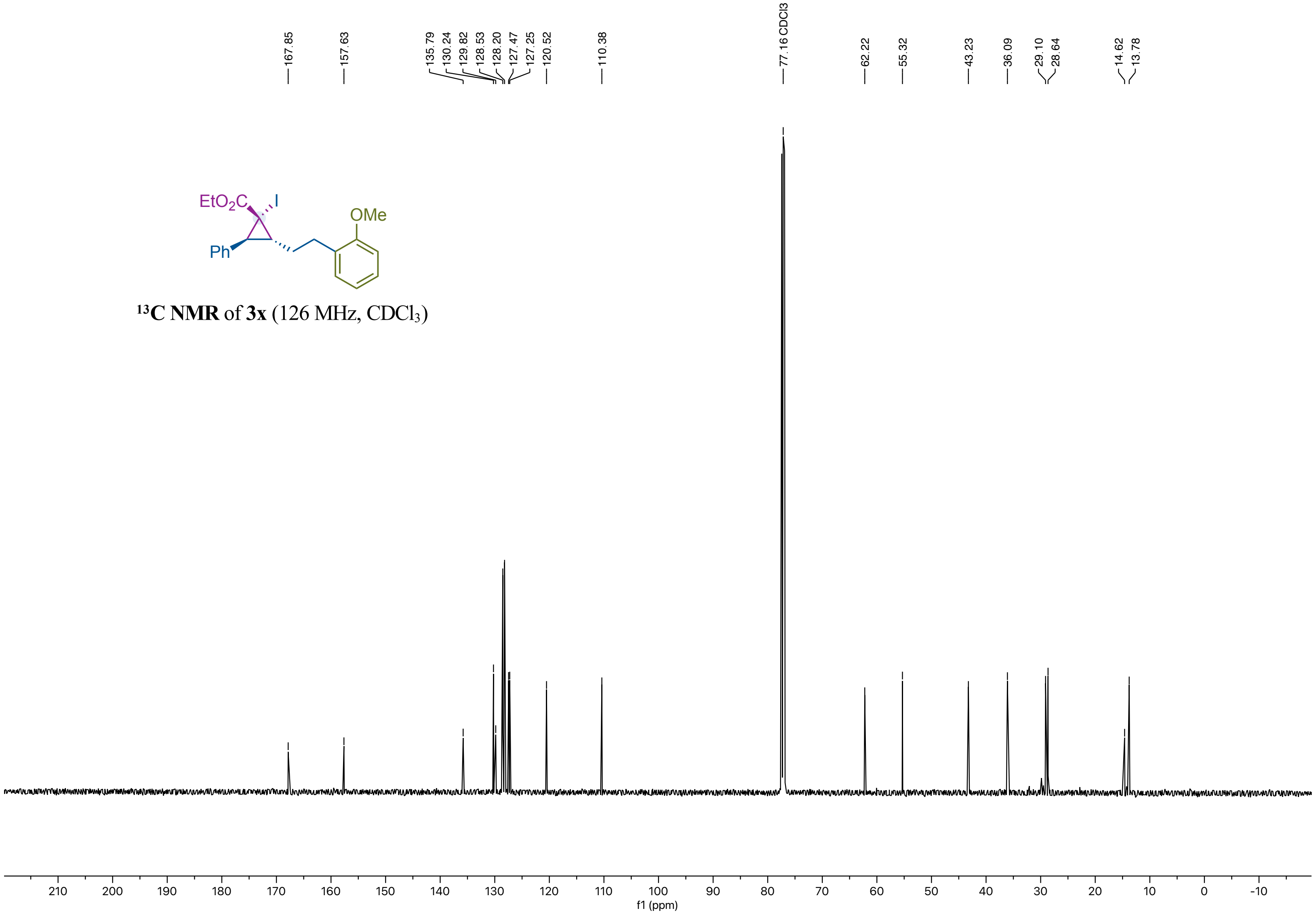

^{13}C NMR of **3x** (126 MHz, $CDCl_3$)

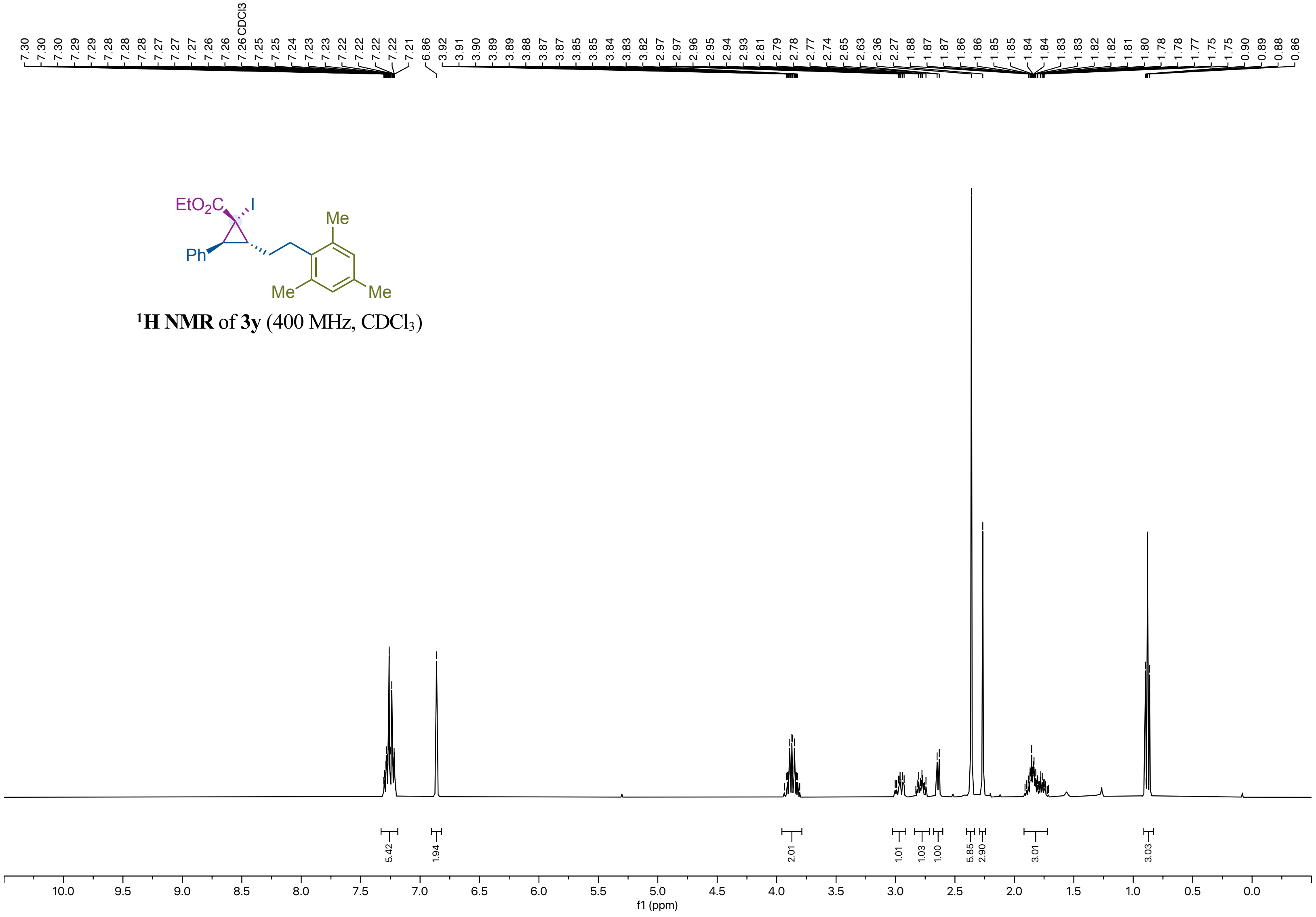

^{1}H NMR of **3y** (400 MHz, $CDCl_3$)

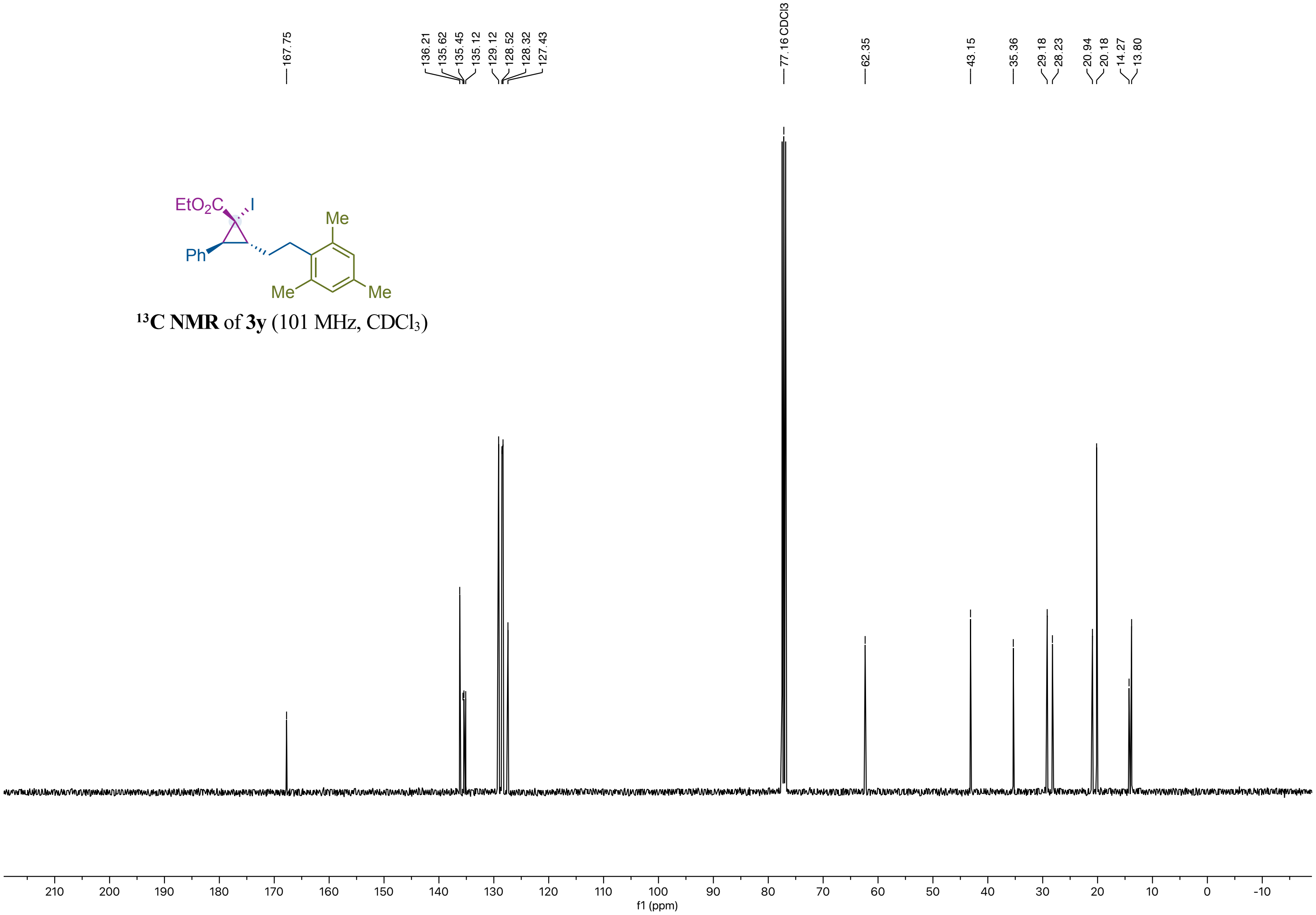

^{13}C NMR of **3y** (101 MHz, $CDCl_3$)

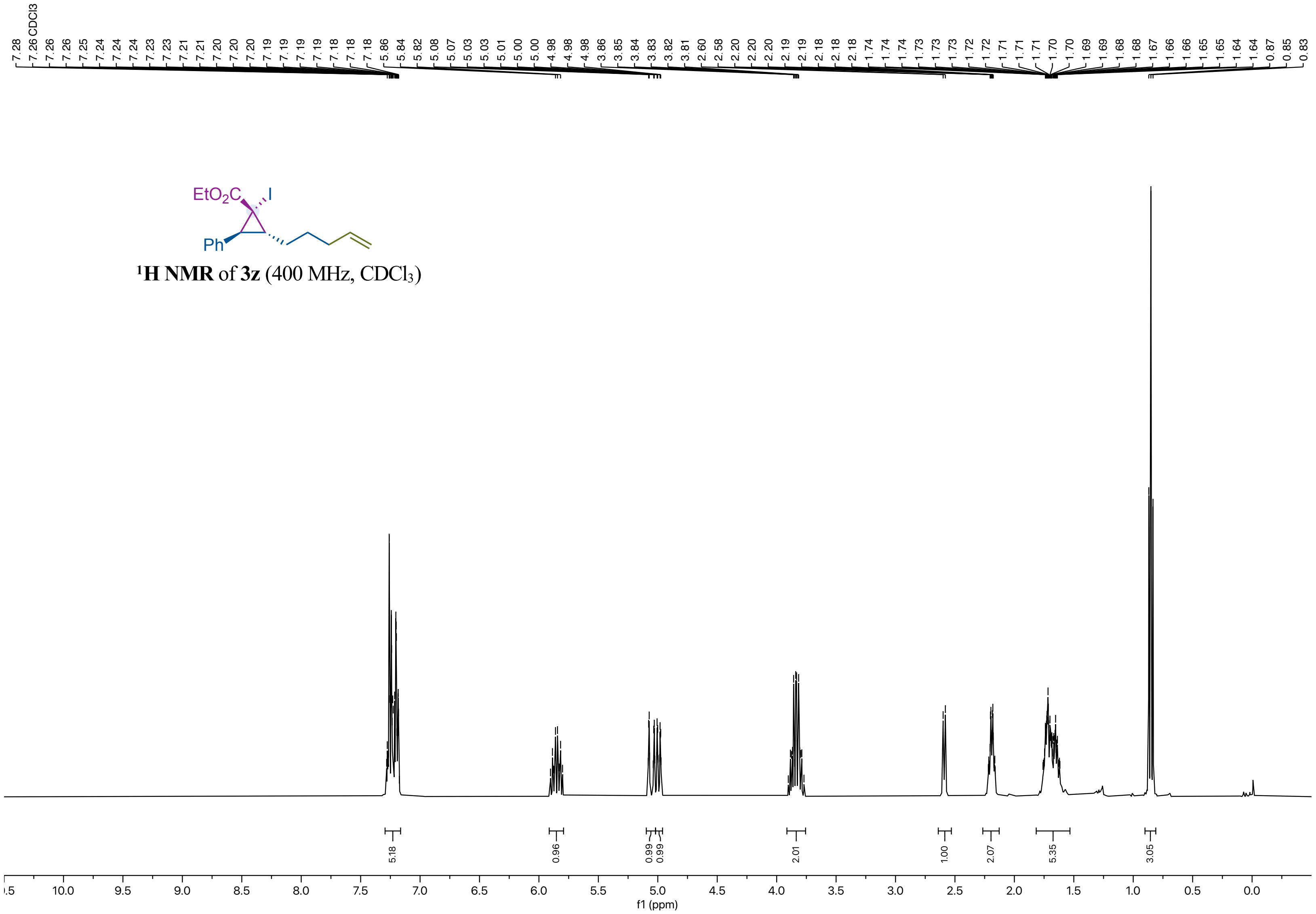

^{1}**H NMR** of **3z** (400 MHz, $CDCl_3$)

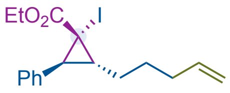

^{13}C NMR of **3z** (126 MHz, $CDCl_3$)

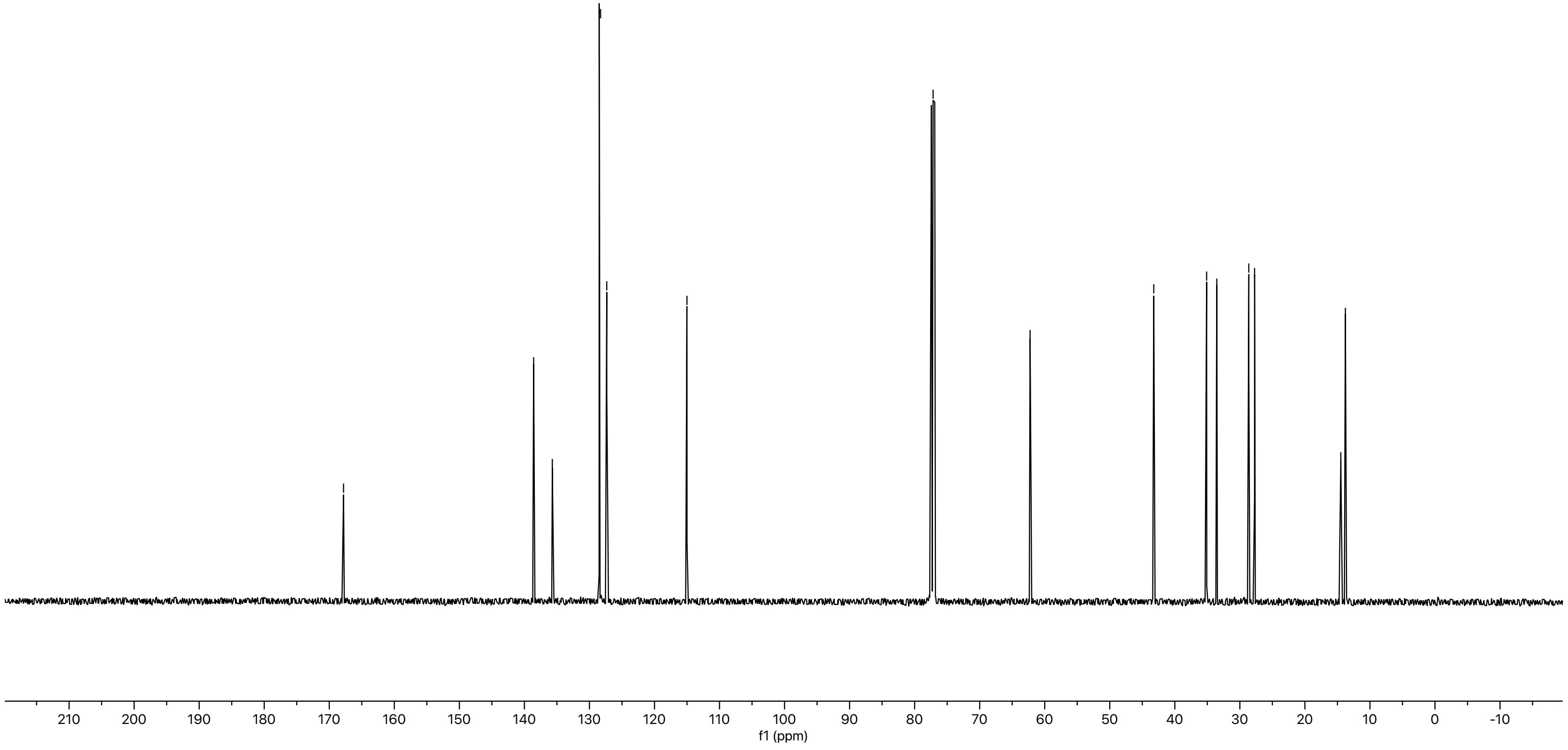

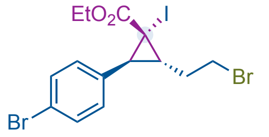

^{1}H NMR of **3aa** (500 MHz, $CDCl_3$)

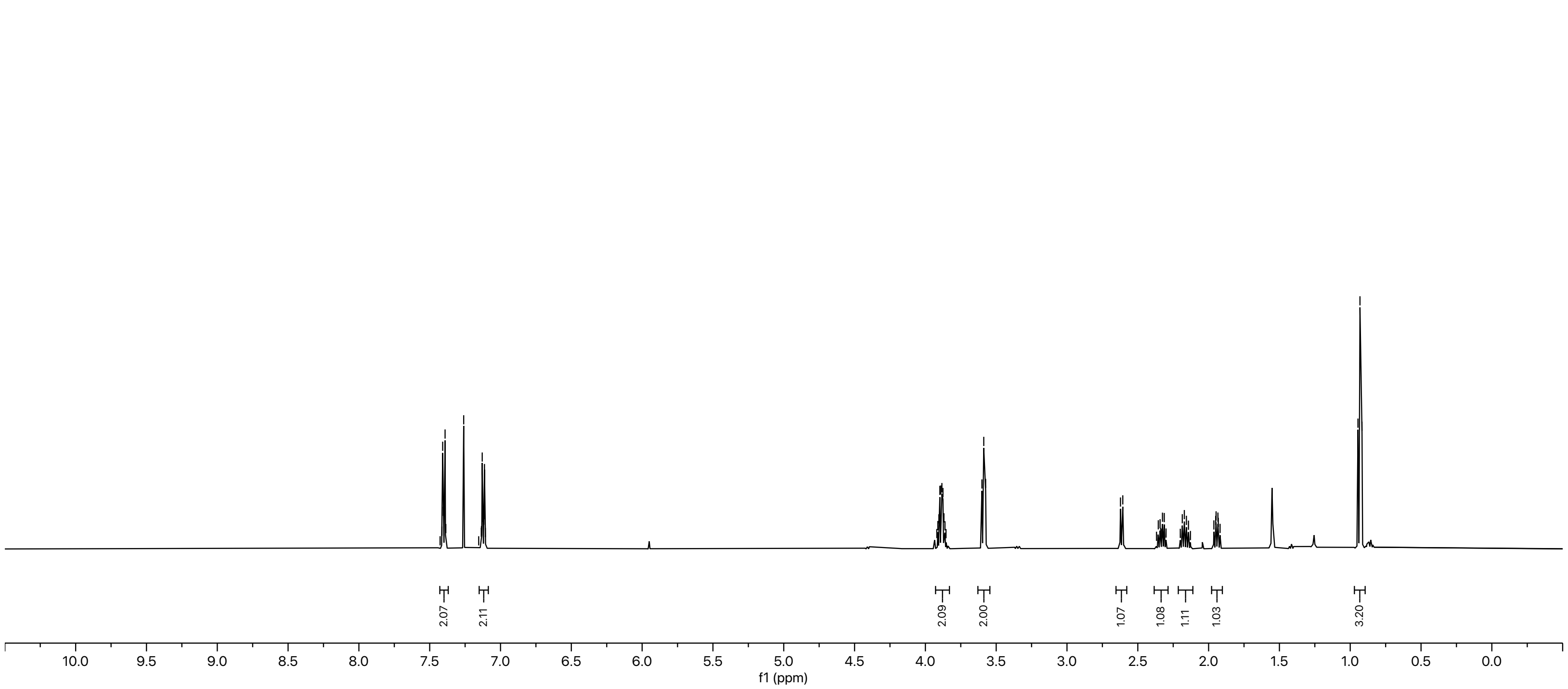

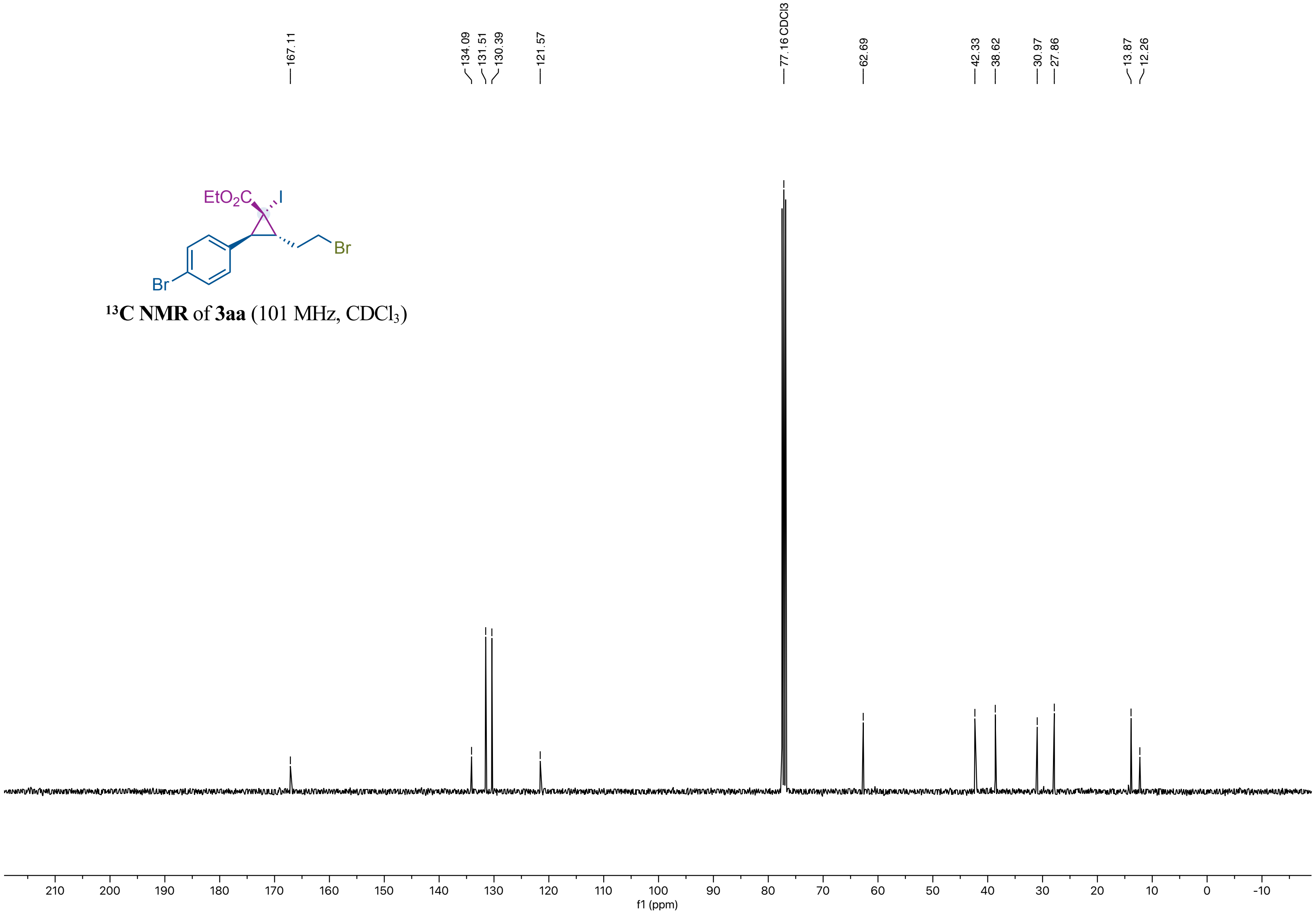

^{13}C NMR of 3aa (101 MHz, $CDCl_3$)

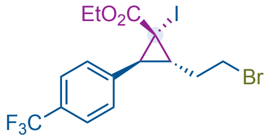

¹H NMR of **3ab** (500 MHz, $CDCl_3$)

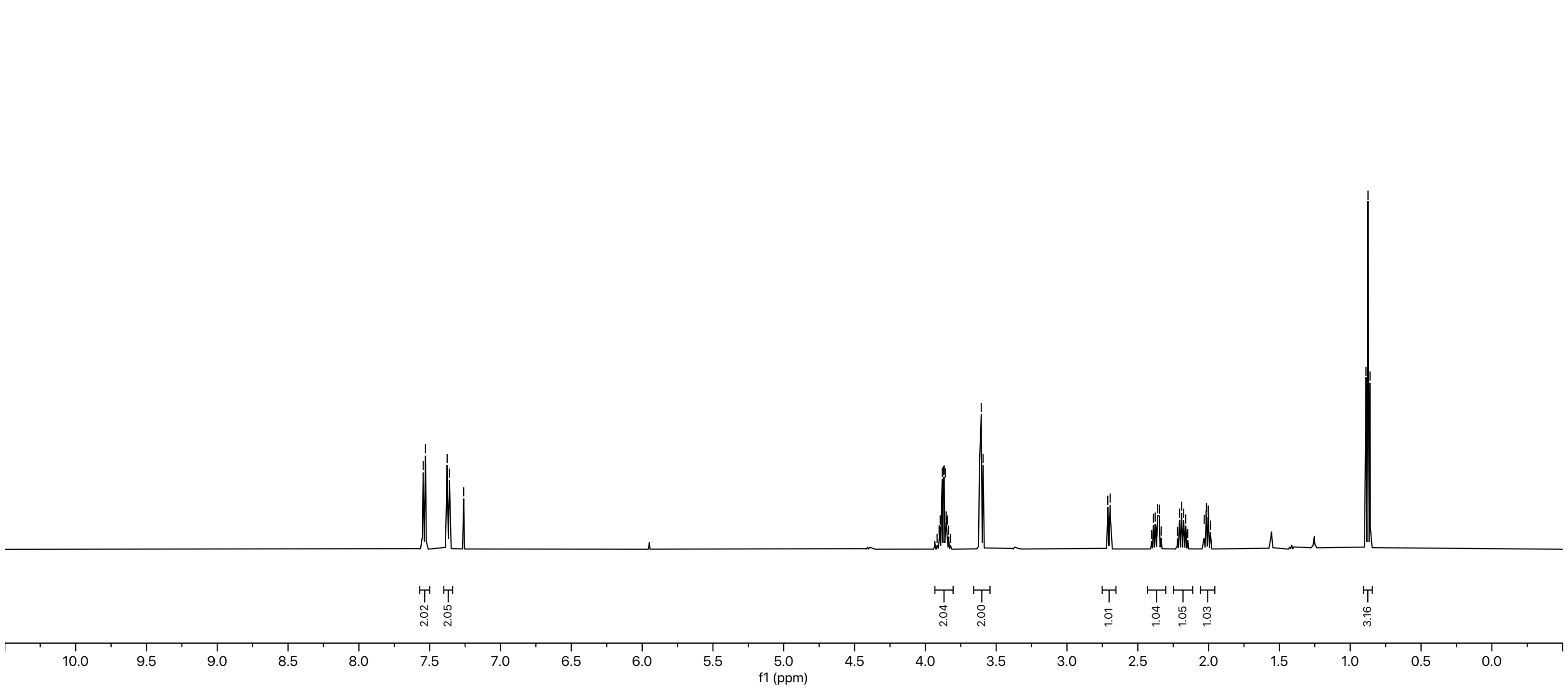

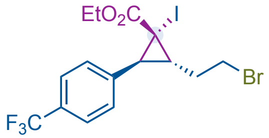

^{13}**C NMR** of **3ab** (126 MHz, $CDCl_3$)

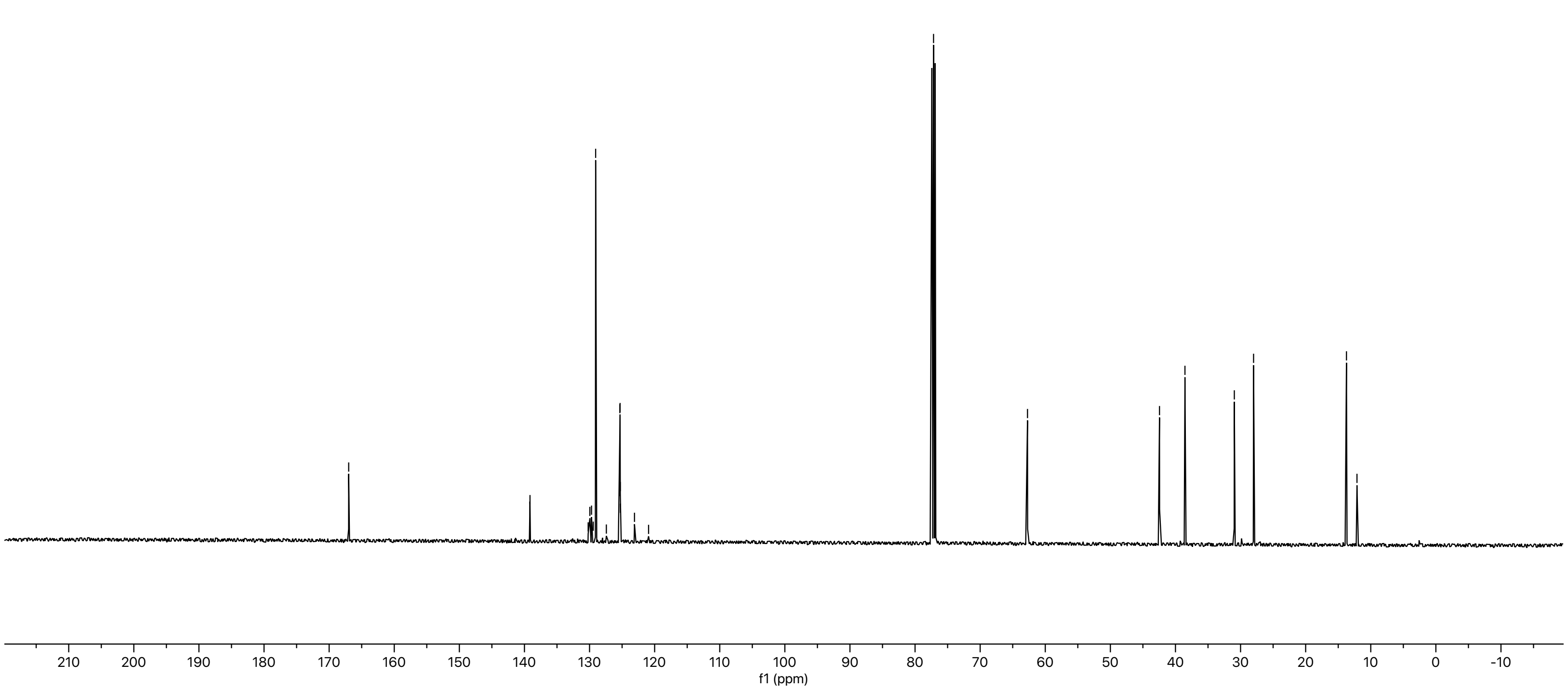

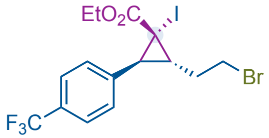

^{19}F NMR of **3ab** (376 MHz, $CDCl_3$)

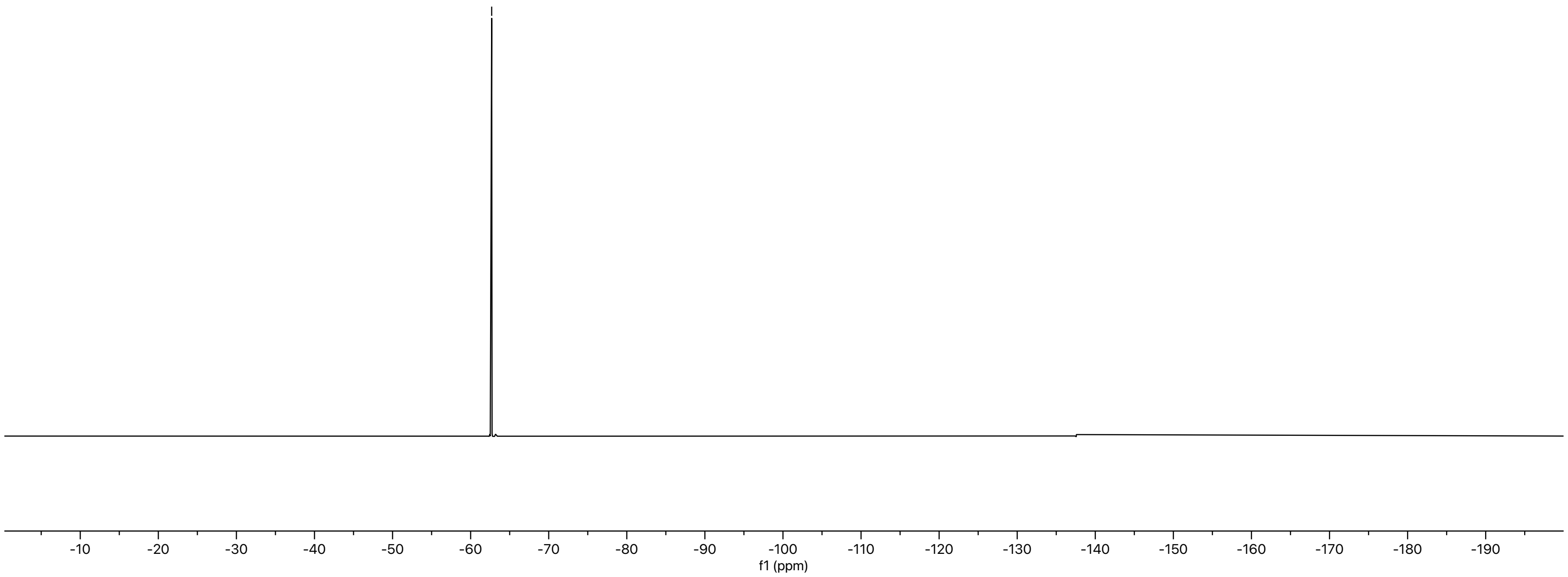

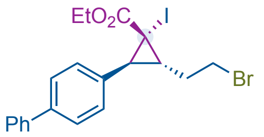

¹H NMR of **3ac** (500 MHz, $CDCl_3$)

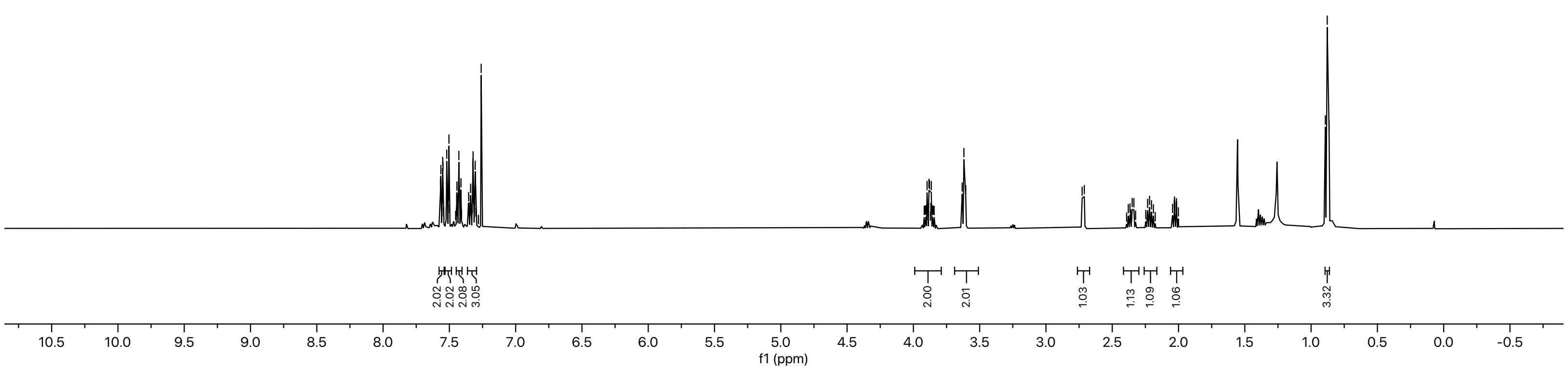

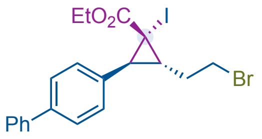

^{13}C NMR of **3ac** (126 MHz, $CDCl_3$)

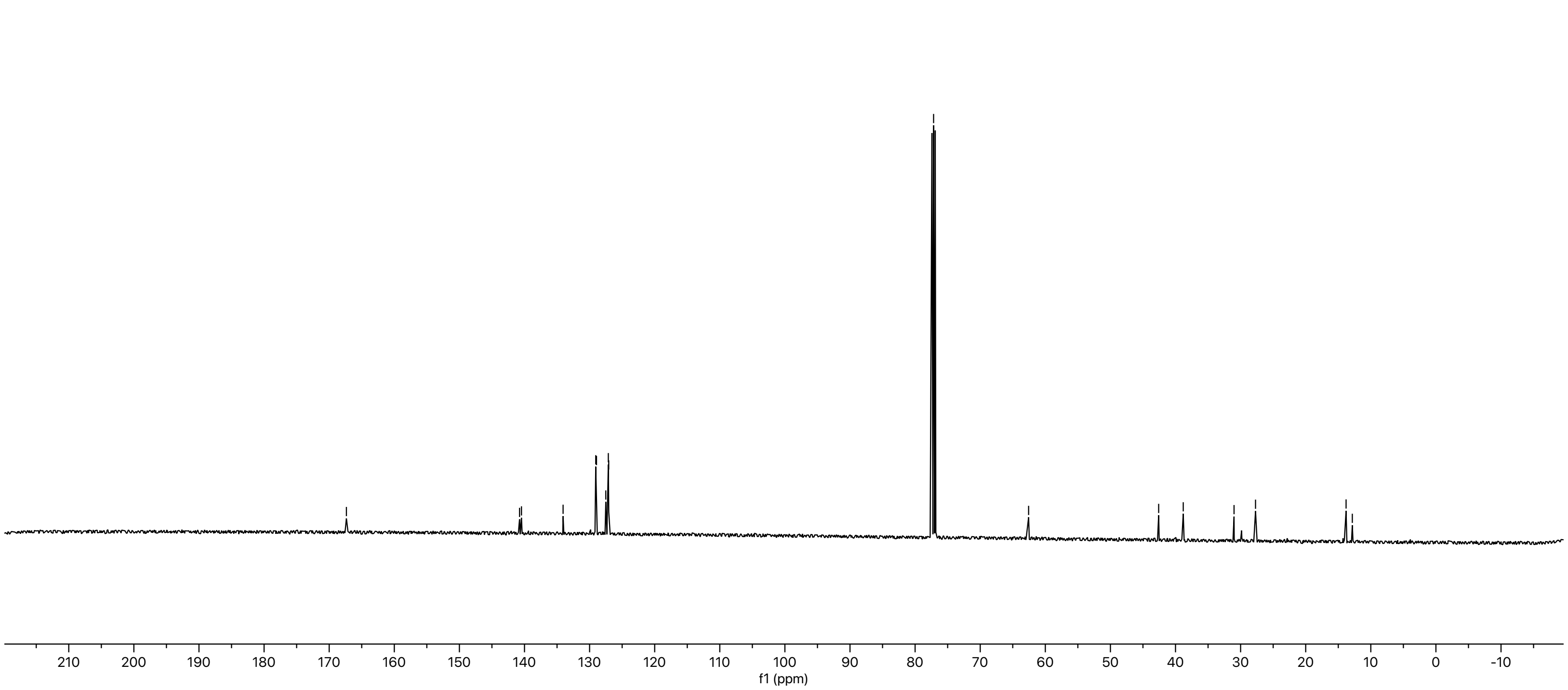

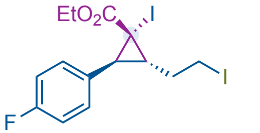

^{1}H NMR of **3ad** (400 MHz, $CDCl_3$)

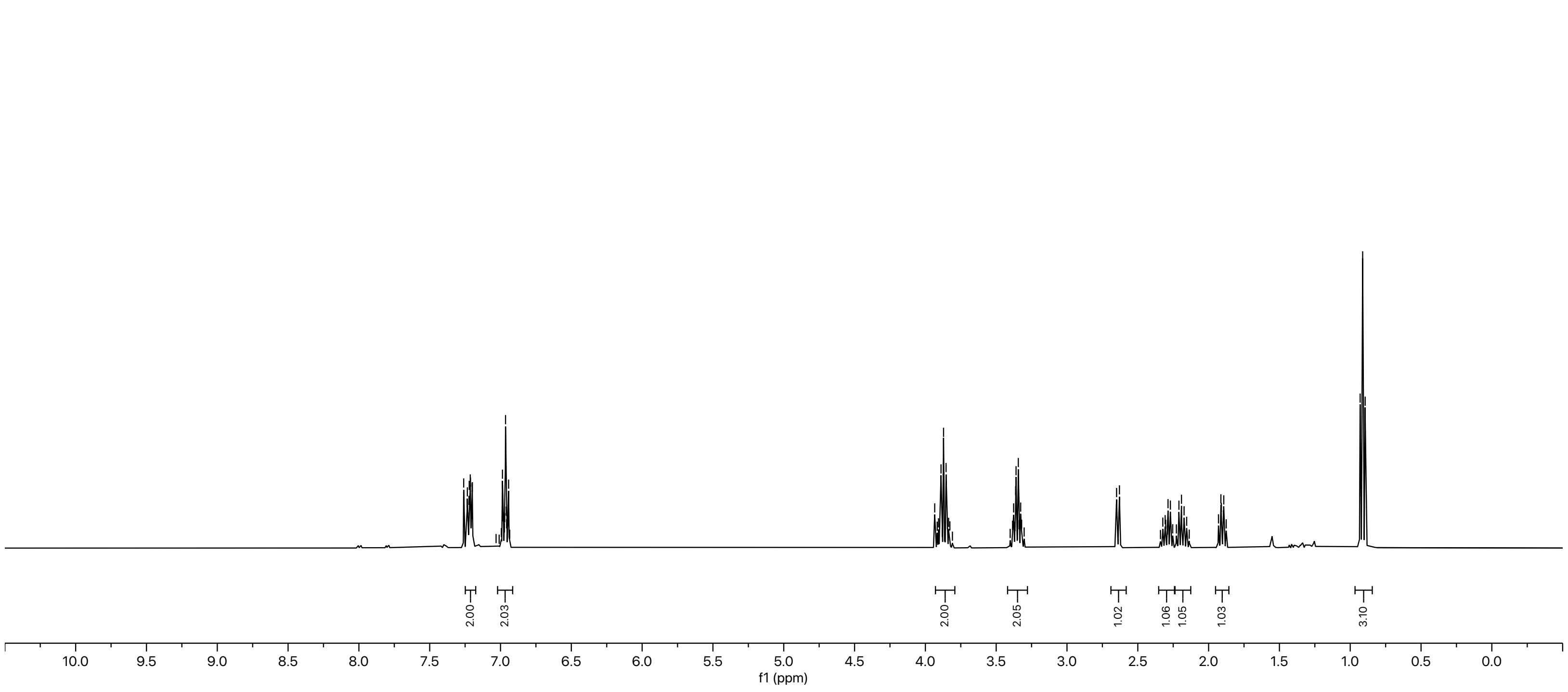

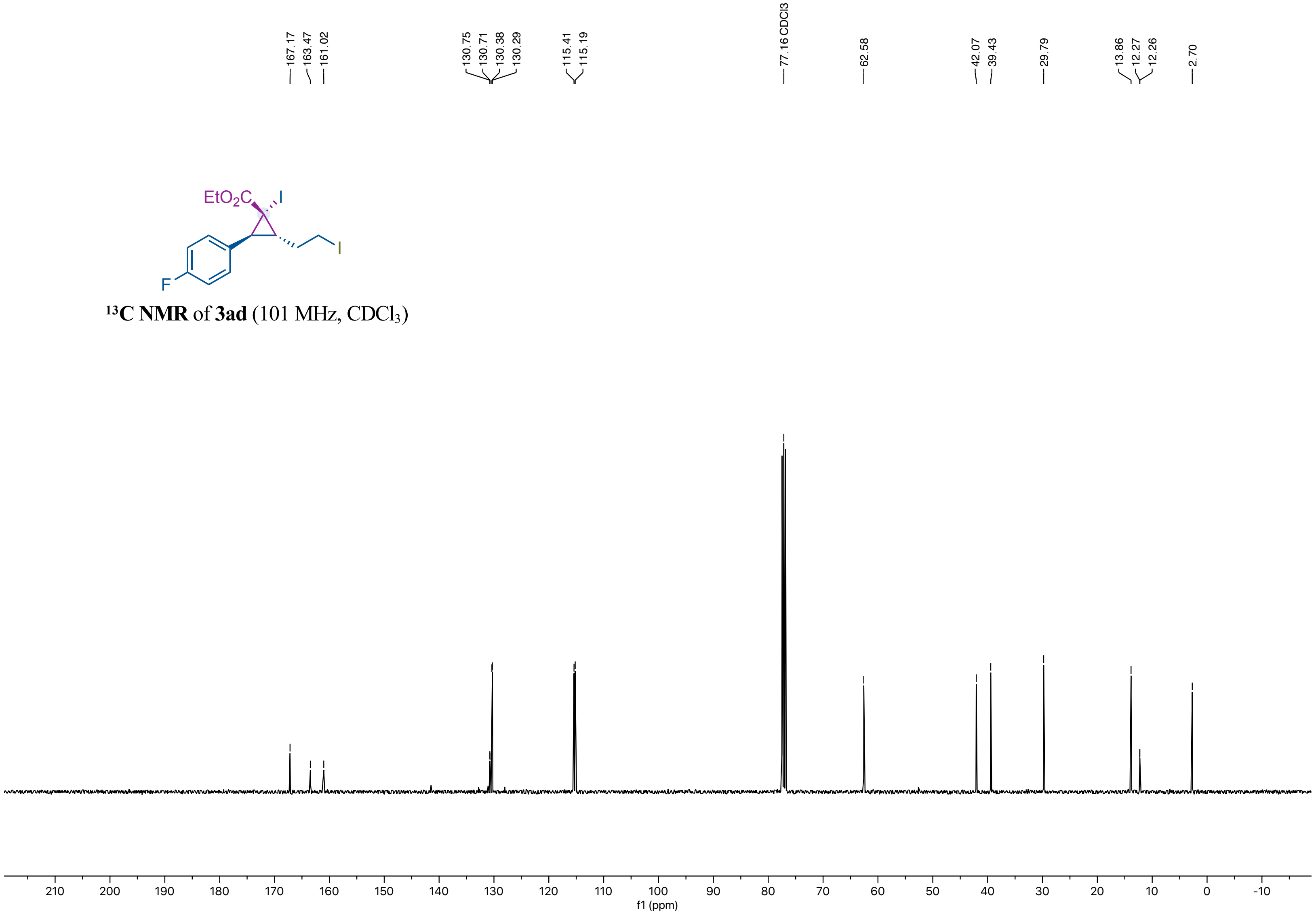

^{13}C NMR of **3ad** (101 MHz, $CDCl_3$)

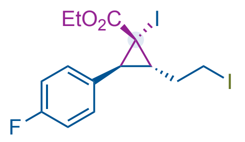

^{19}F NMR of **3ad** (471 MHz, $CDCl_3$)

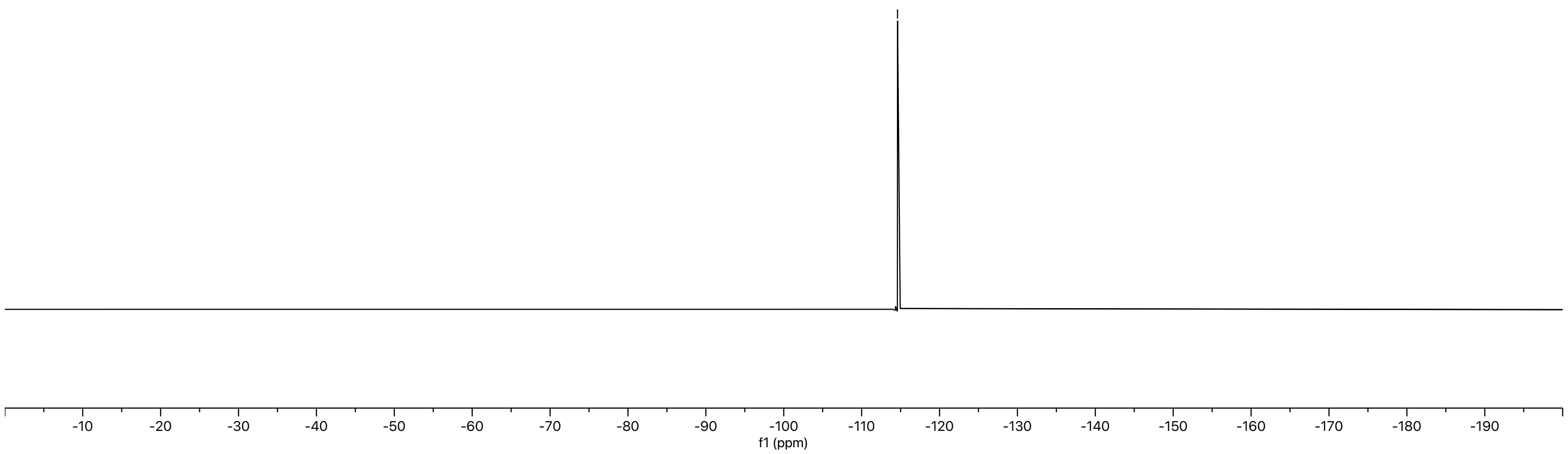

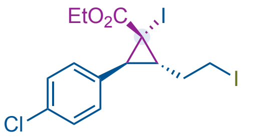

¹H NMR of **3ae** (400 MHz, $CDCl_3$)

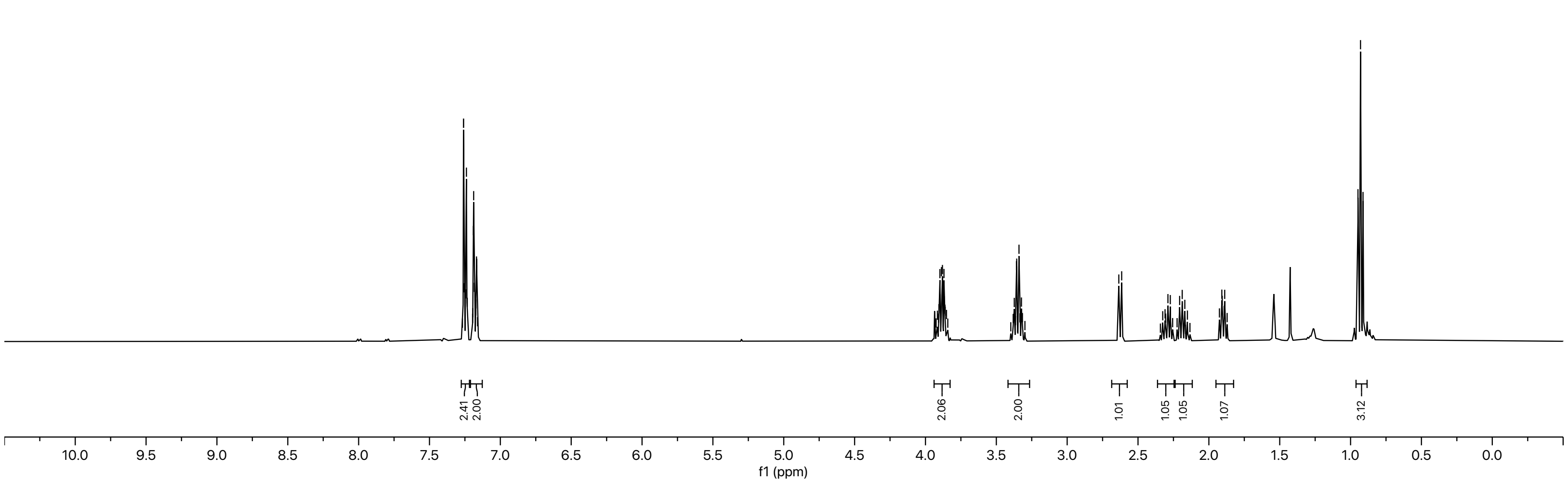

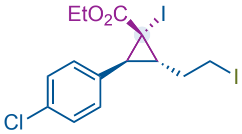

^{13}C NMR of **3ae** (101 MHz, $CDCl_3$)

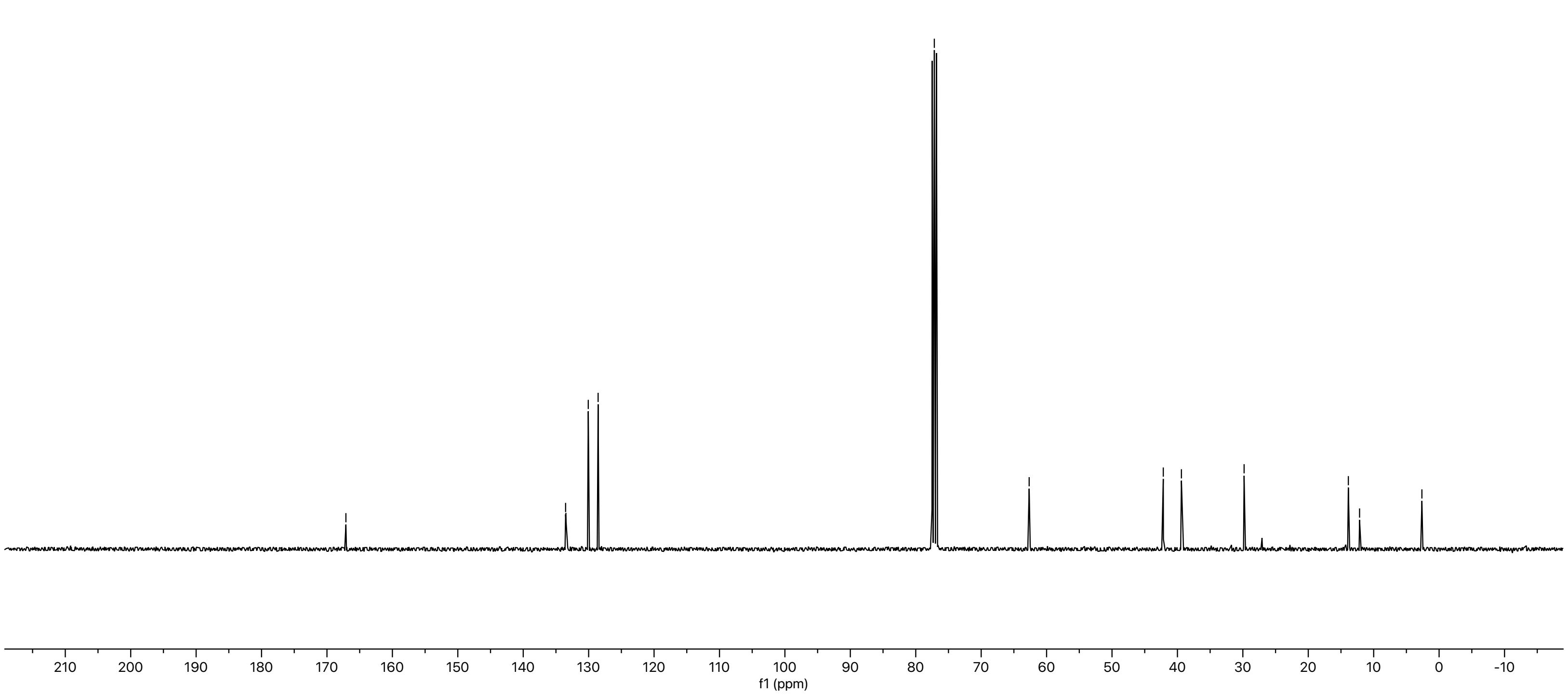

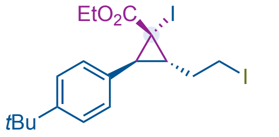

¹H NMR of **3af** (400 MHz, $CDCl_3$)

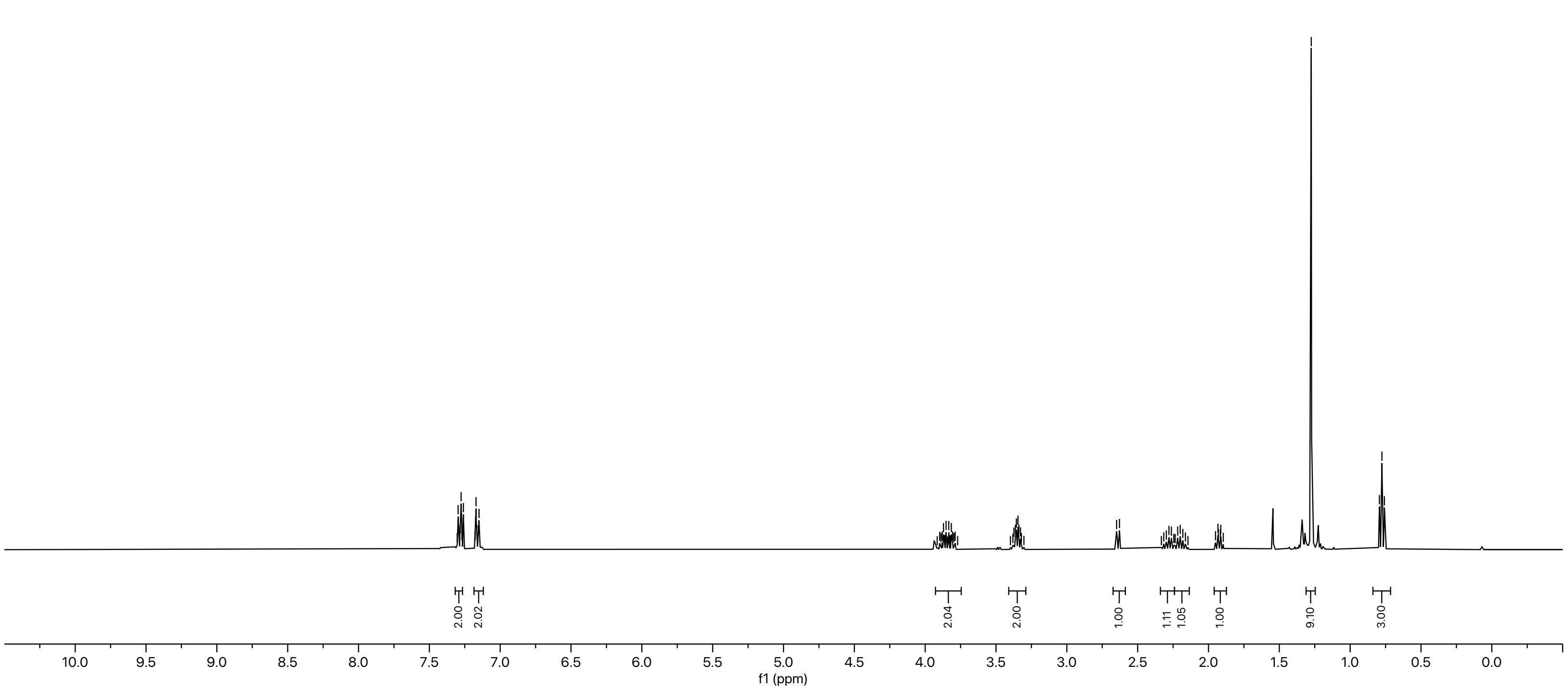

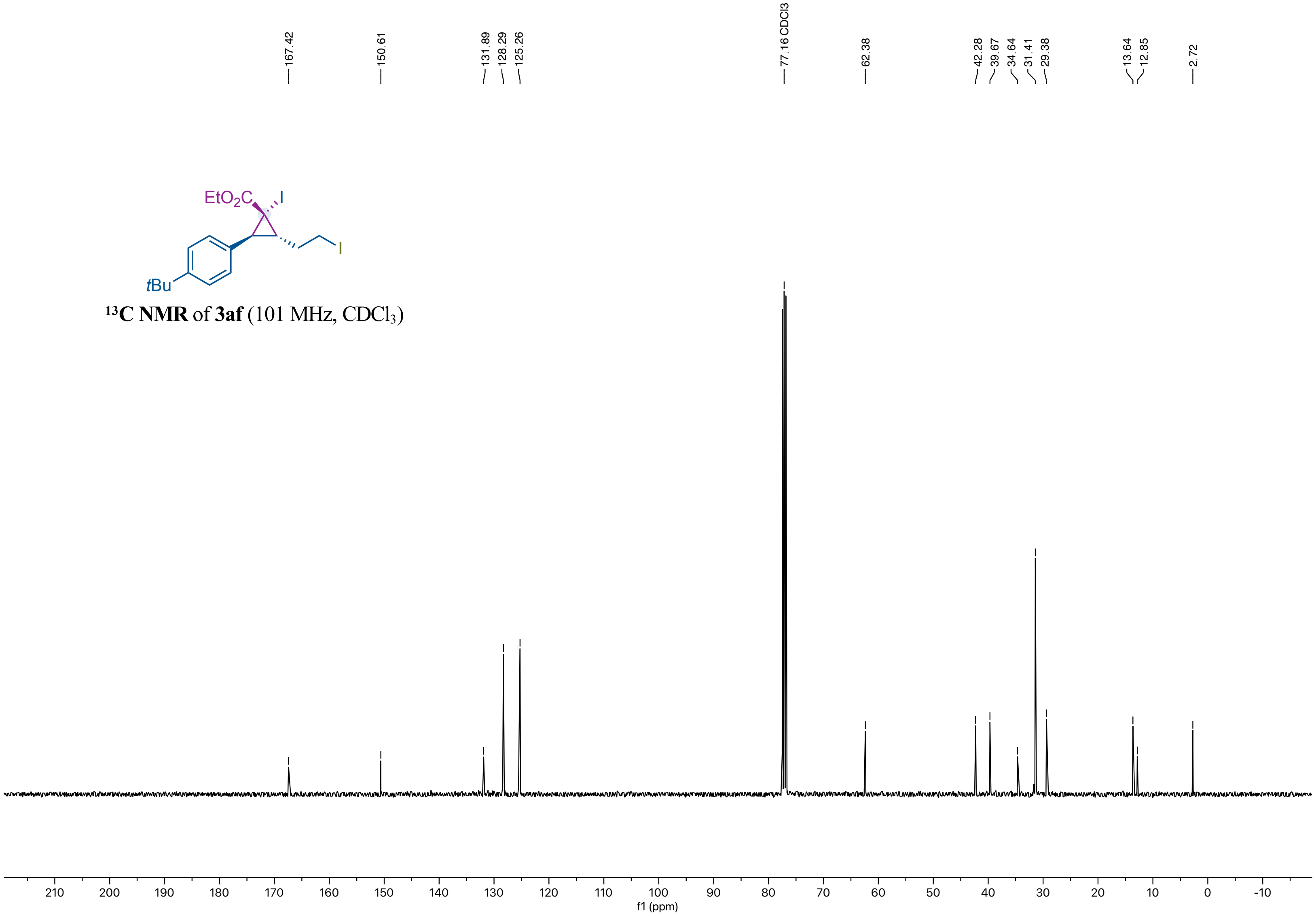

^{13}**C NMR** of **3af** (101 MHz, $CDCl_3$)

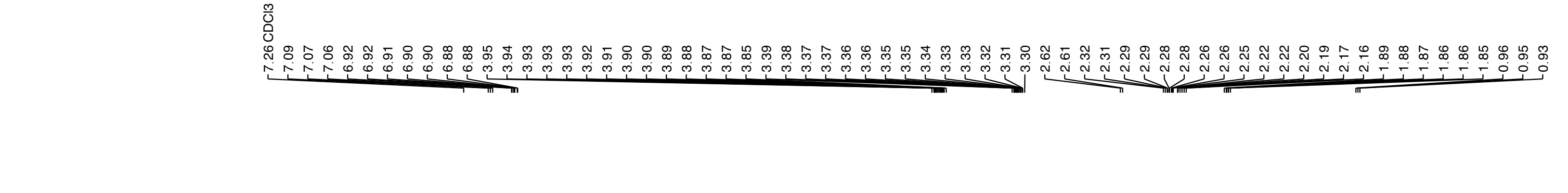

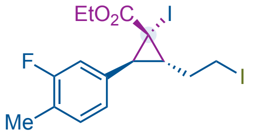

^{1}H NMR of **3ag** (500 MHz, $CDCl_3$)

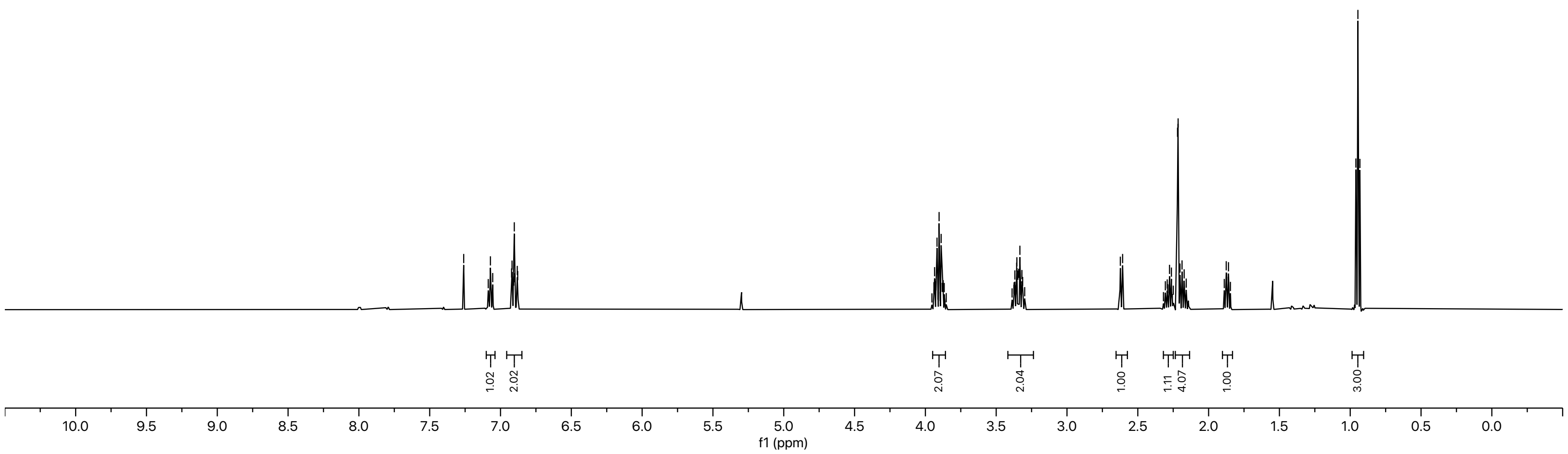

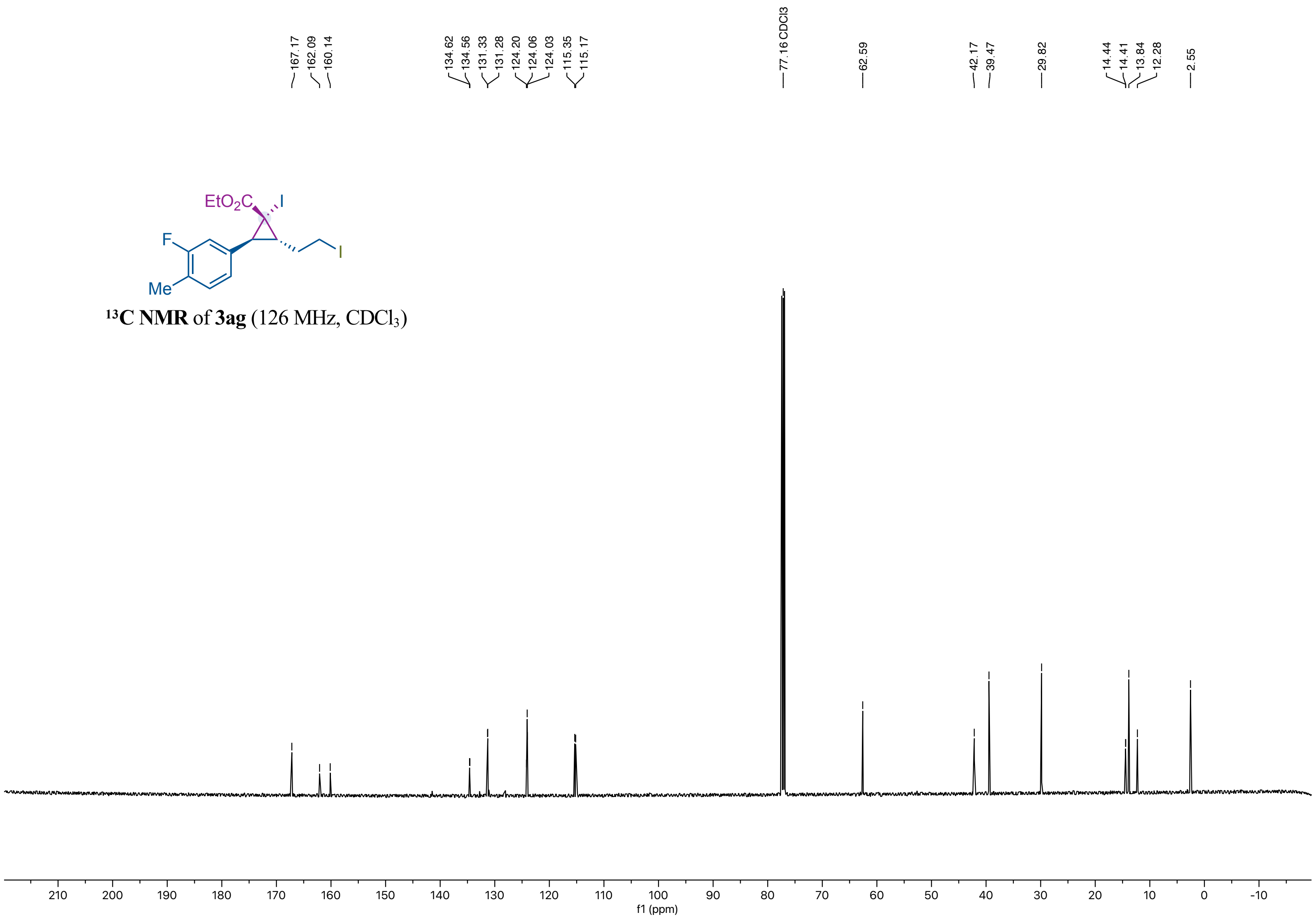

^{13}C NMR of **3ag** (126 MHz, $CDCl_3$)

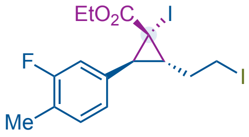

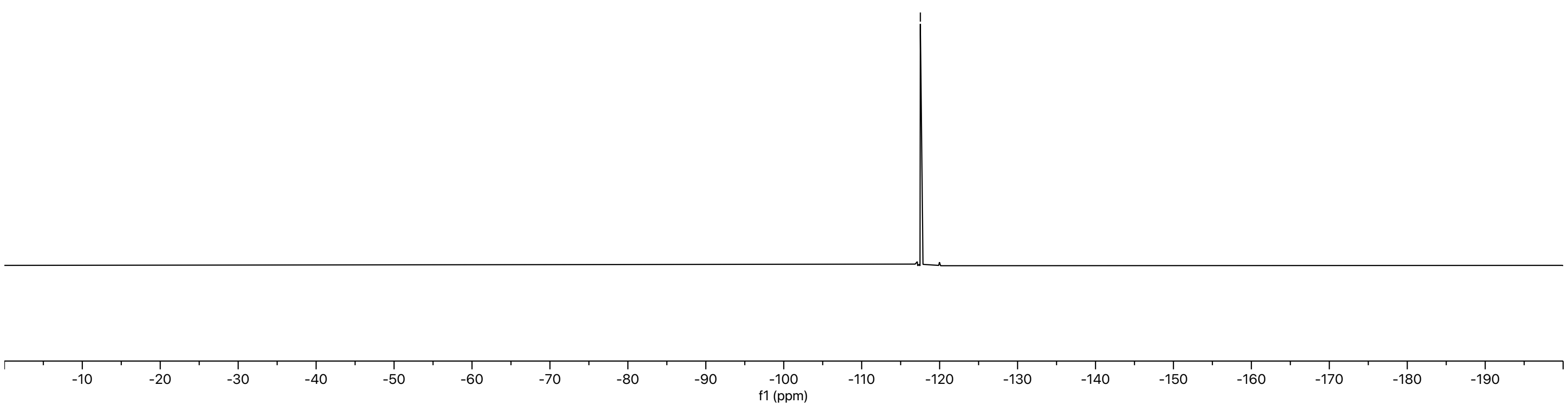

^{19}F NMR of **3ag** (471 MHz, $CDCl_3$)

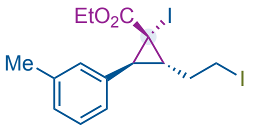

¹H NMR of **3ah** (400 MHz, $CDCl_3$)

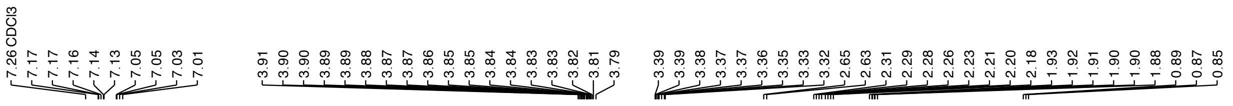
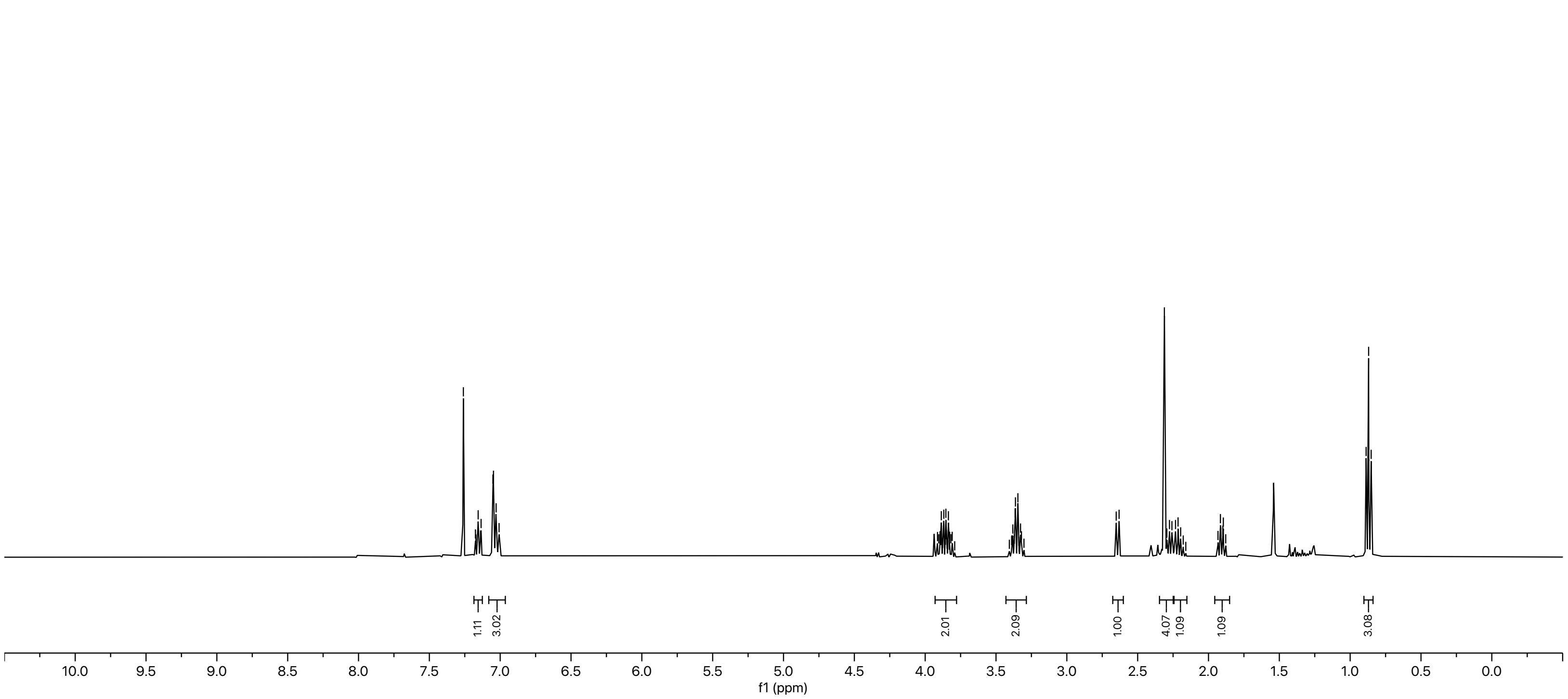

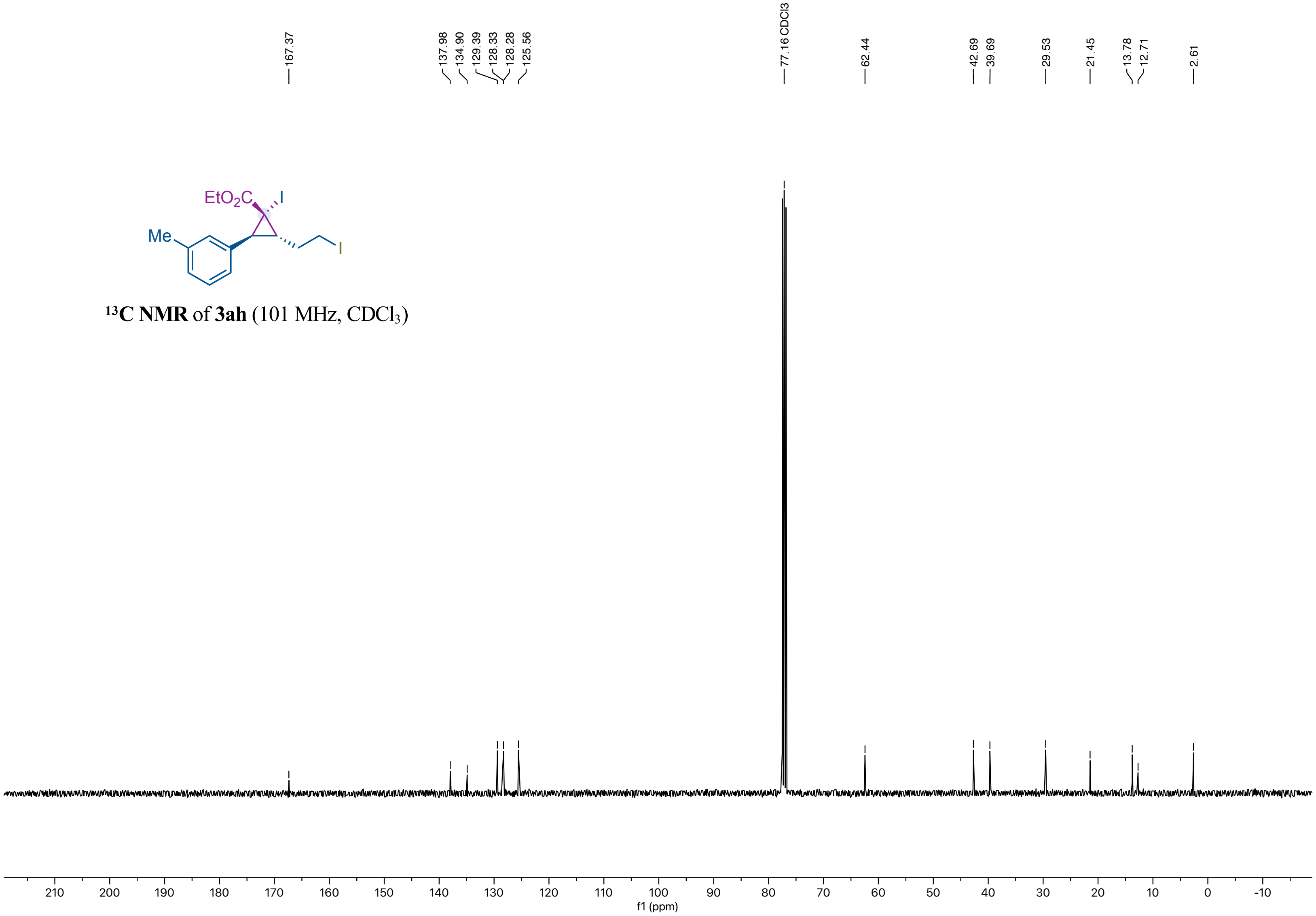

^{13}C NMR of **3ah** (101 MHz, $CDCl_3$)

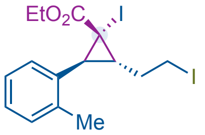

^{1}H NMR of **3ai** (400 MHz, $CDCl_3$)

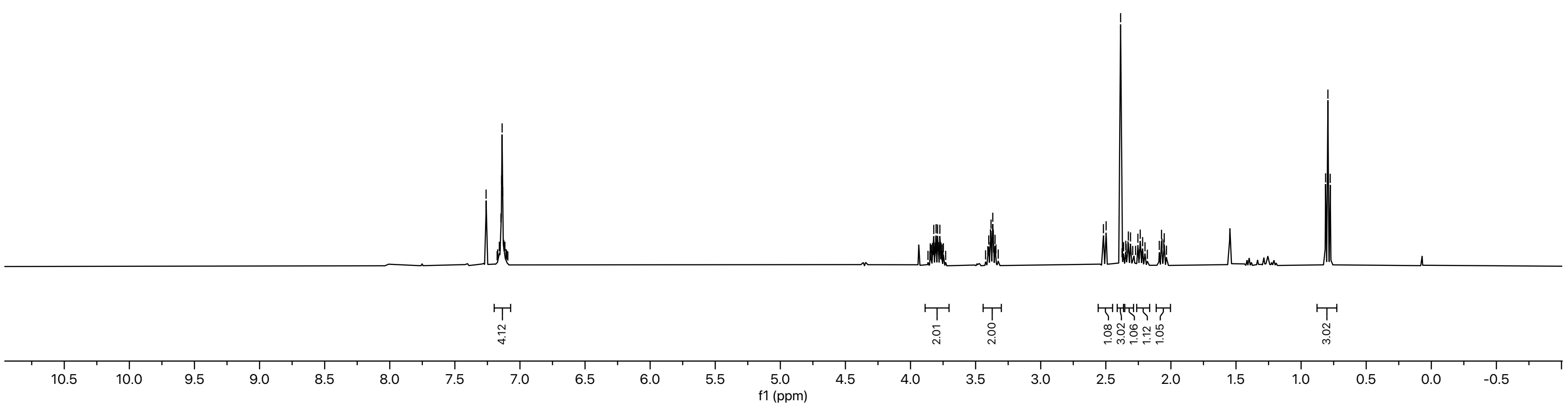

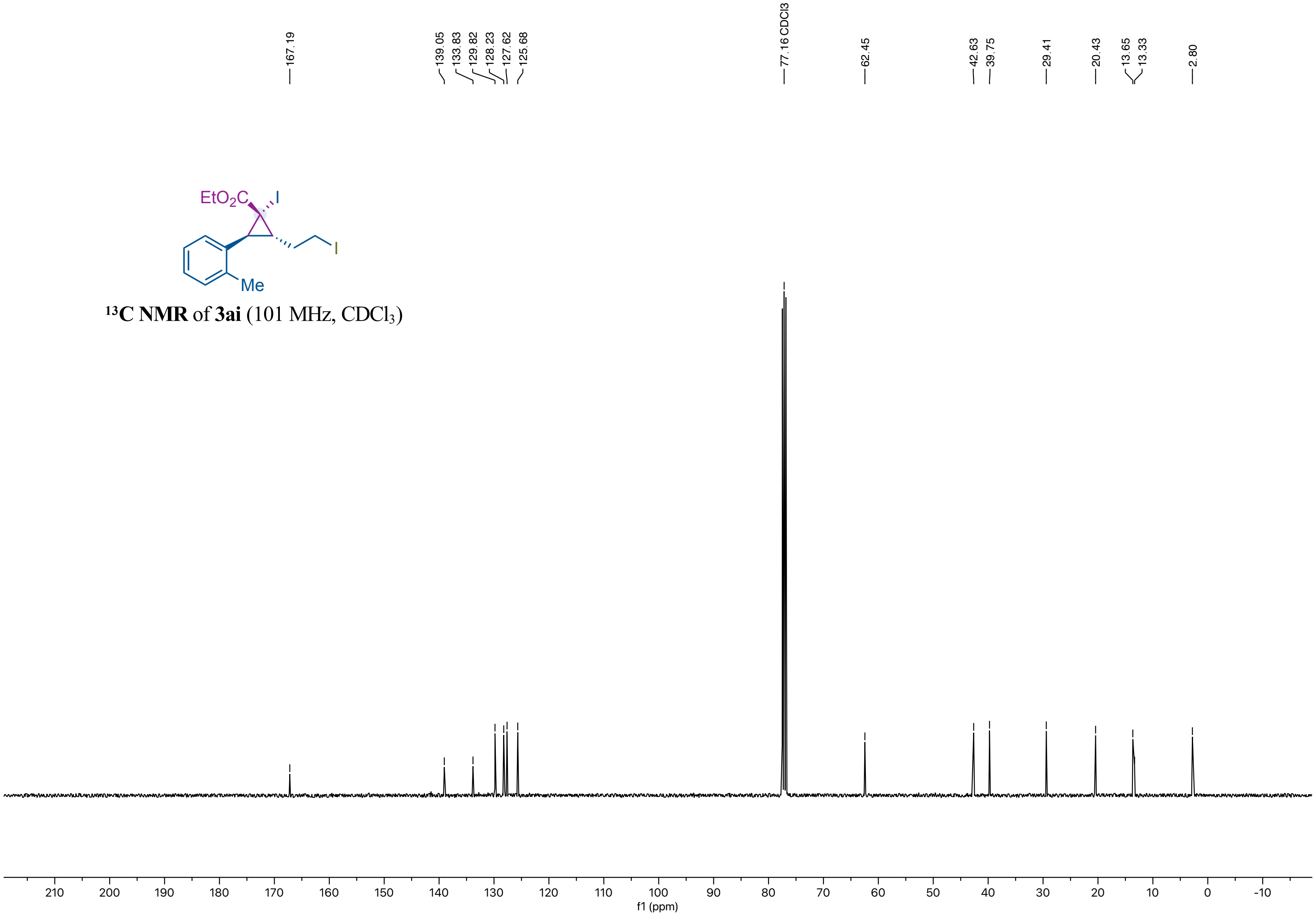

^{13}C NMR of **3ai** (101 MHz, $CDCl_3$)

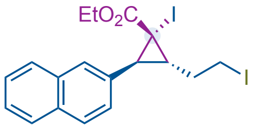

[1]**H NMR** of **3aj** (400 MHz, $CDCl_3$)

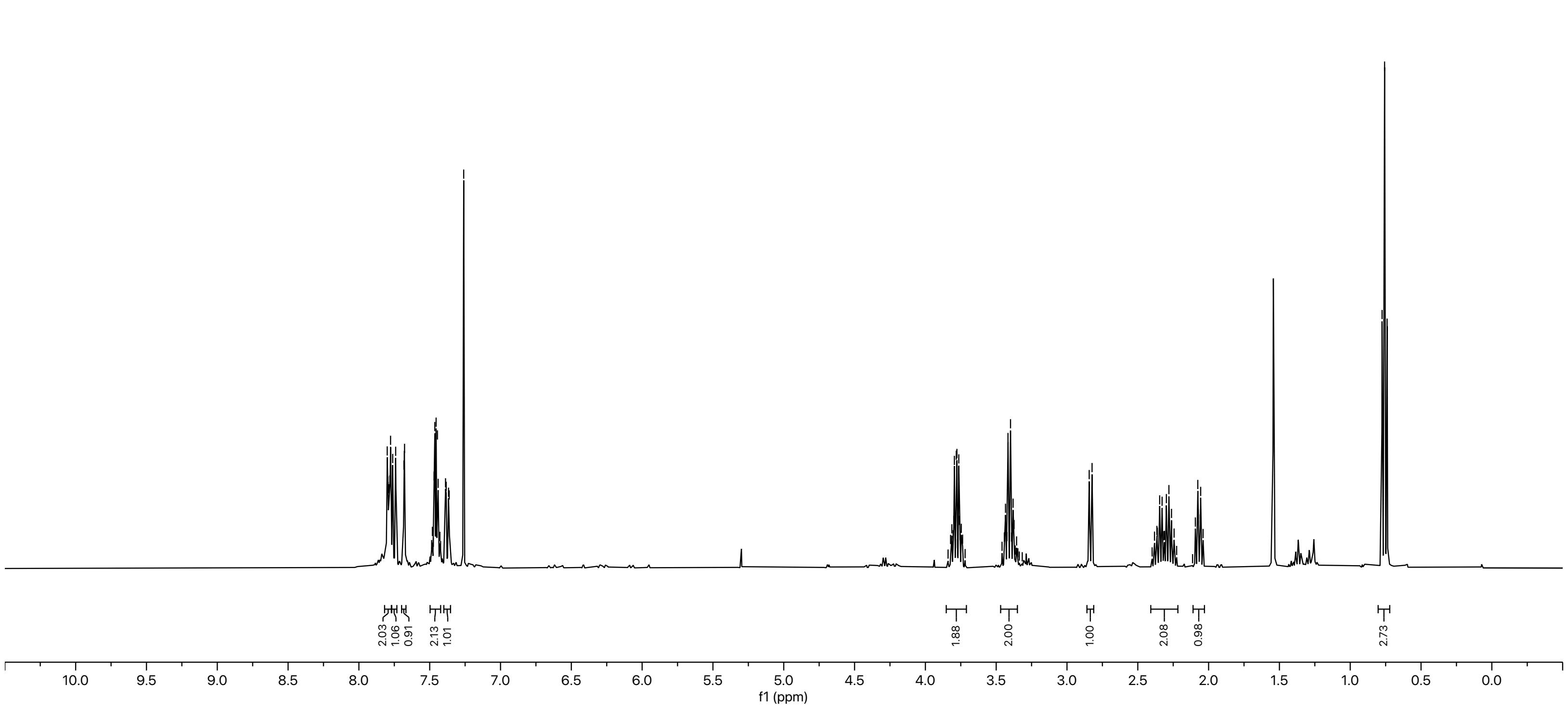

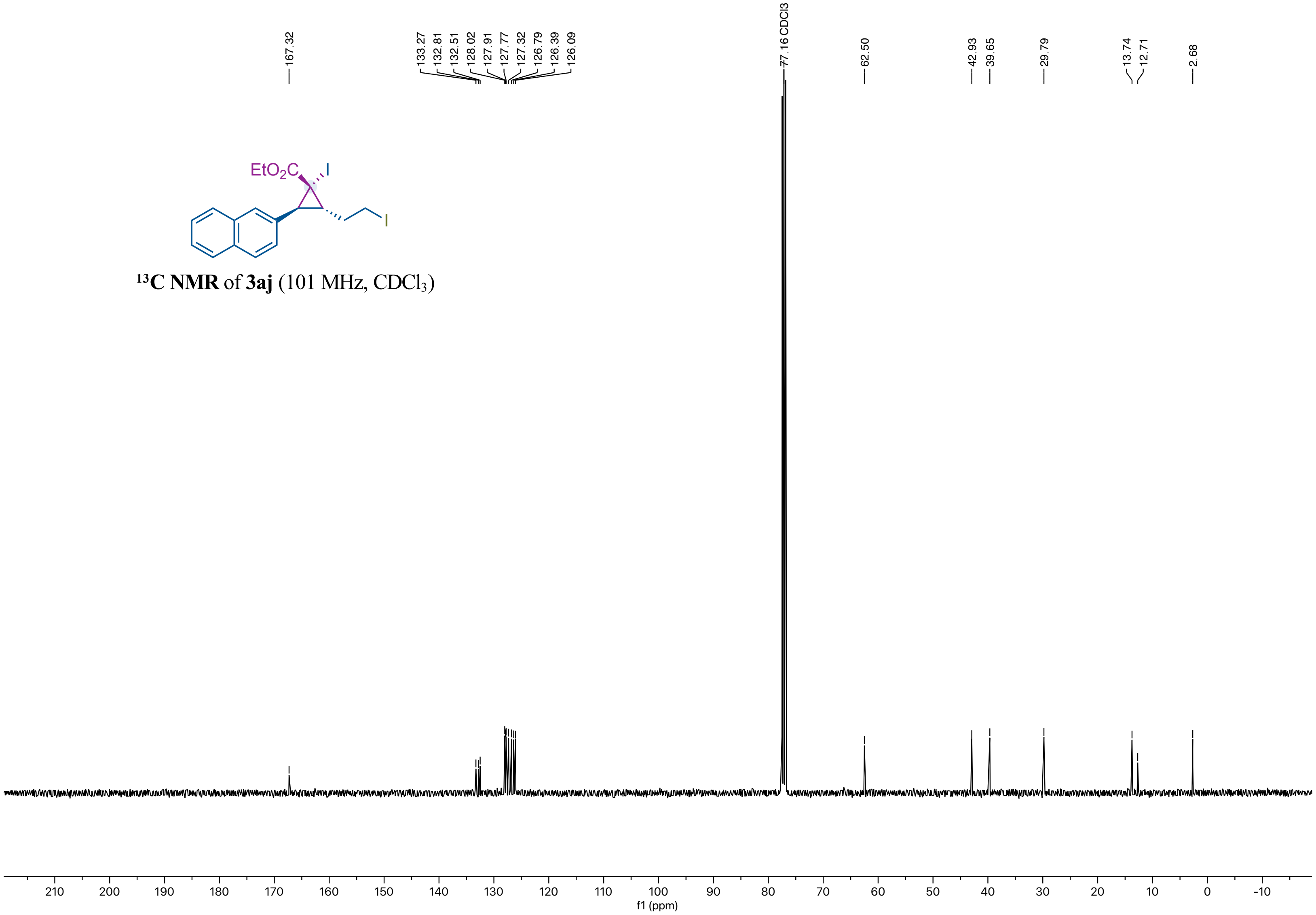

^{13}C NMR of **3aj** (101 MHz, $CDCl_3$)

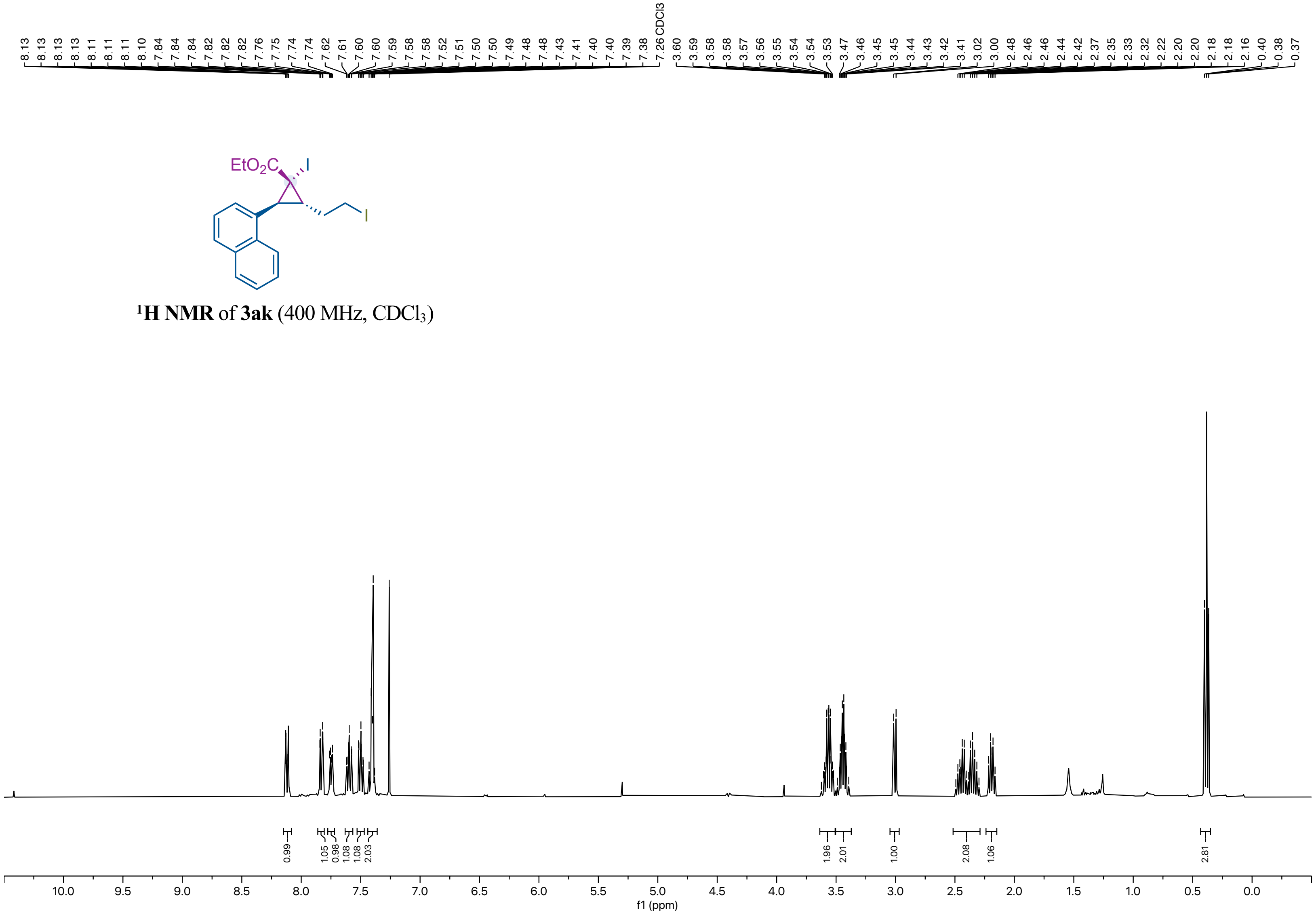

^{1}H NMR of **3ak** (400 MHz, $CDCl_3$)

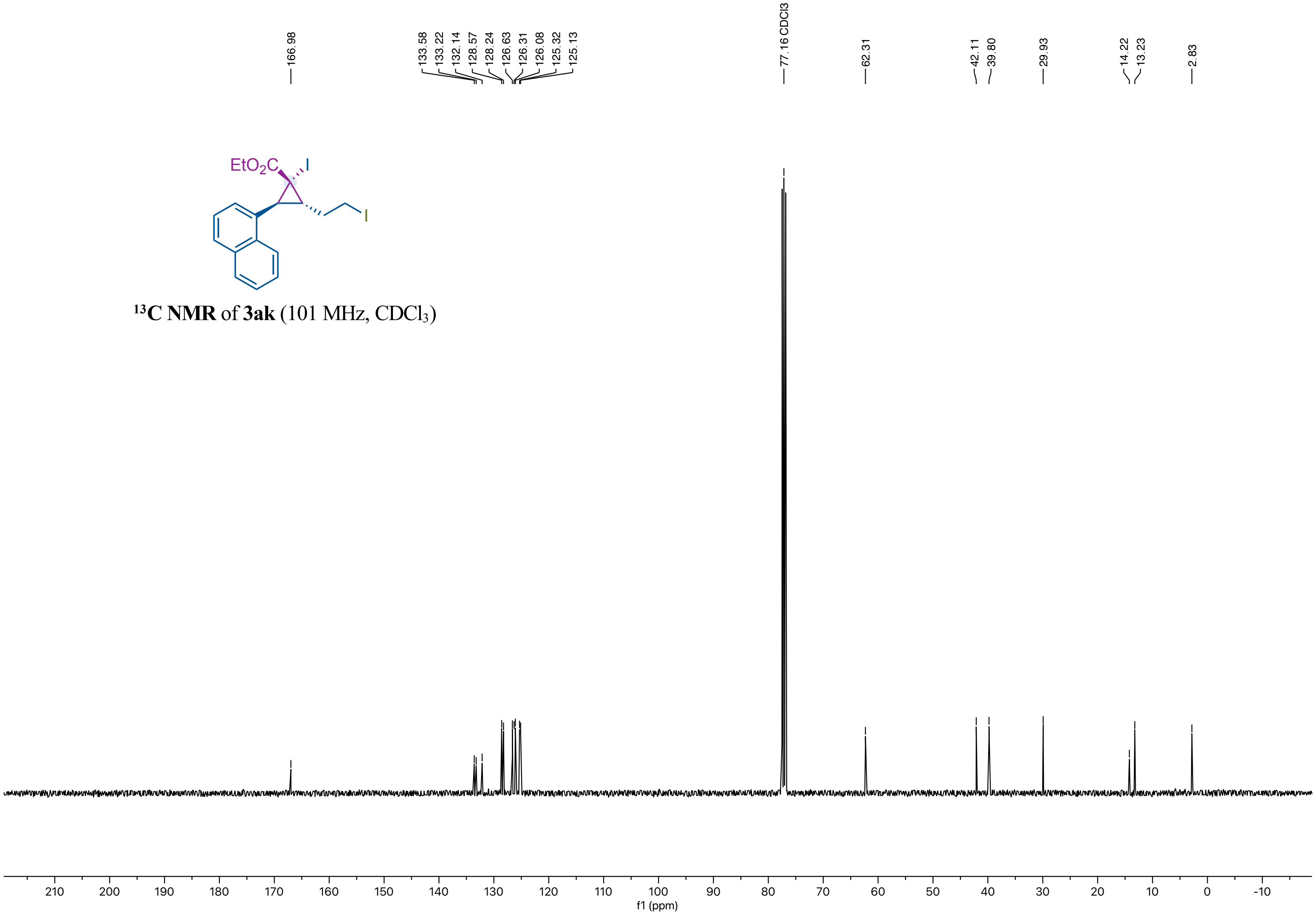

^{13}C NMR of **3ak** (101 MHz, $CDCl_3$)

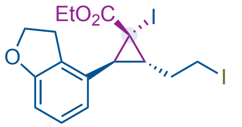

^{1}H NMR of **3al** (500 MHz, $CDCl_3$)

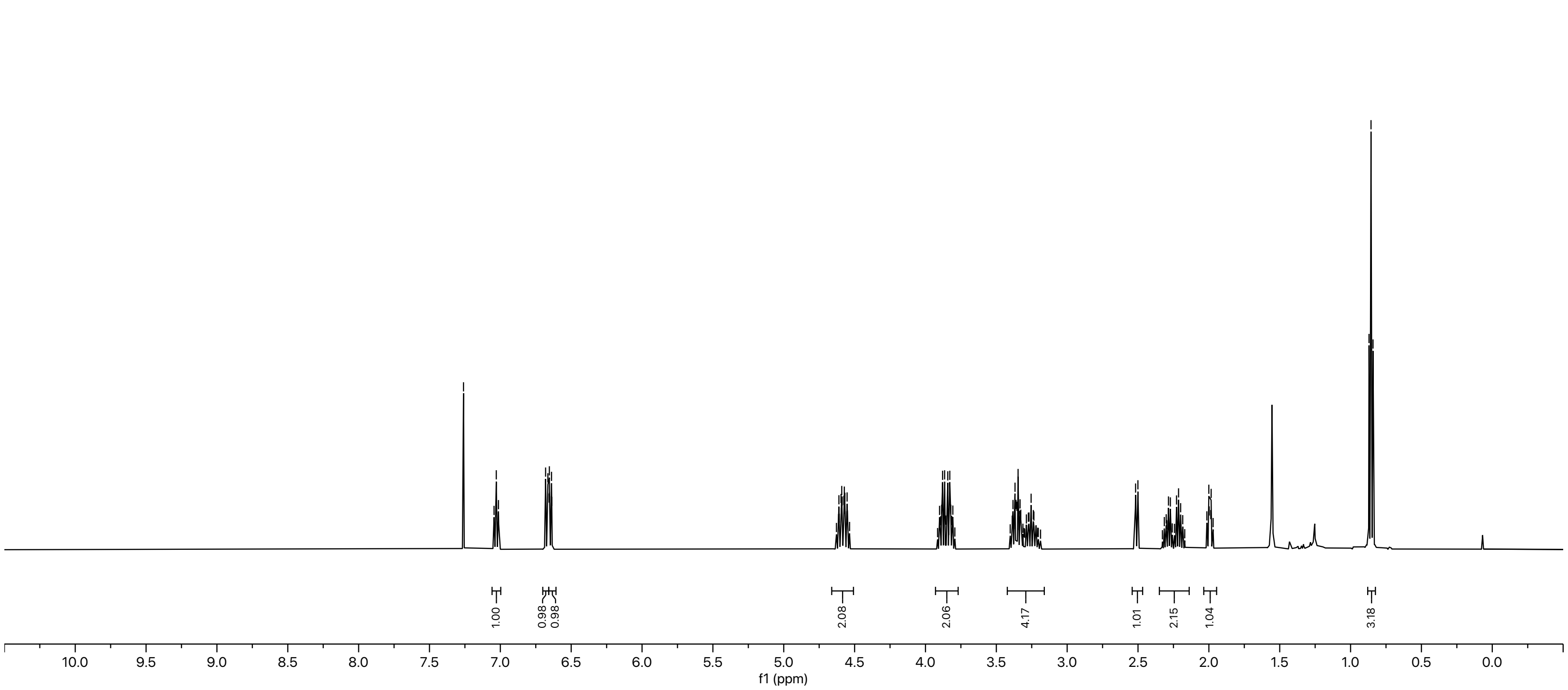

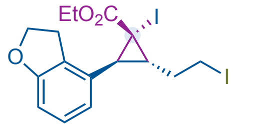

¹³C NMR of **3al** (126 MHz, $CDCl_3$)

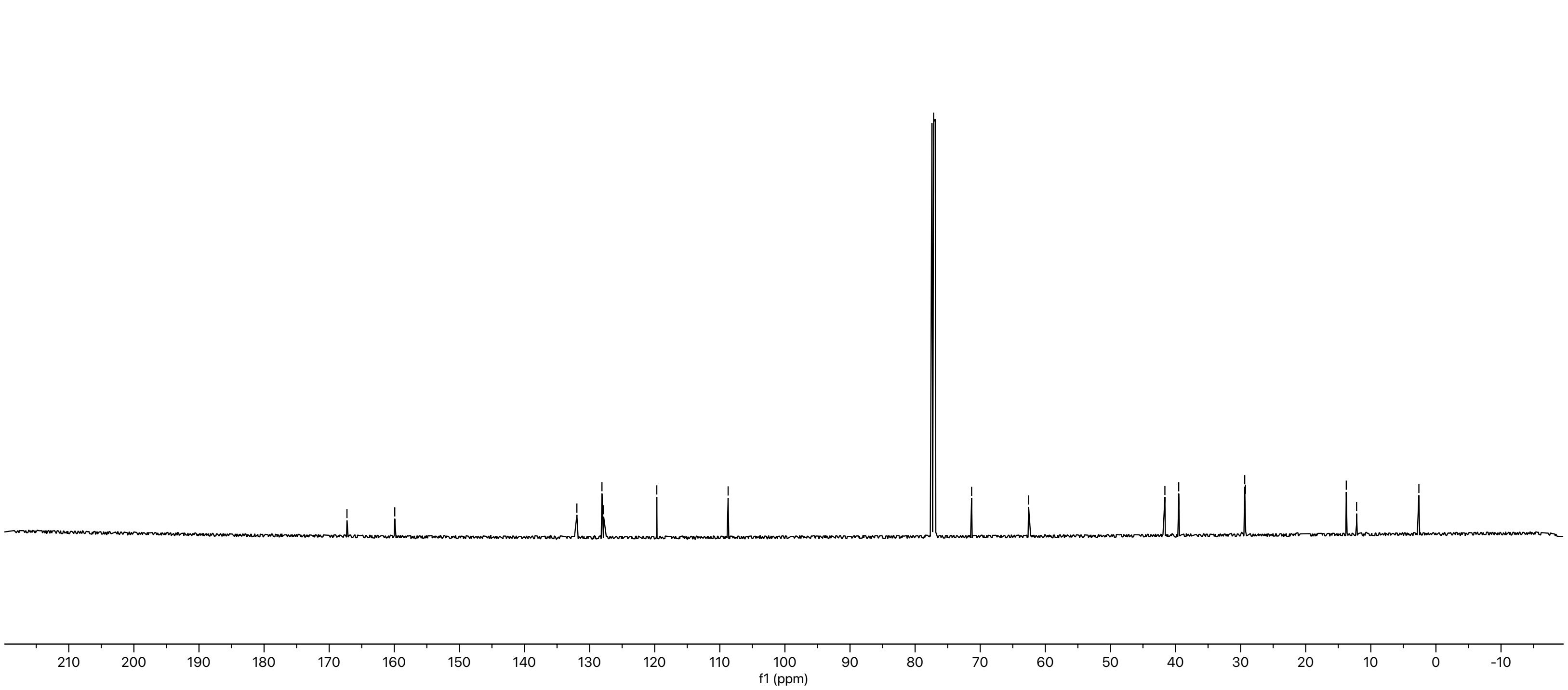

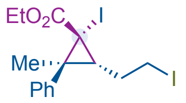

^{1}H NMR of **3am** (400 MHz, $CDCl_3$)

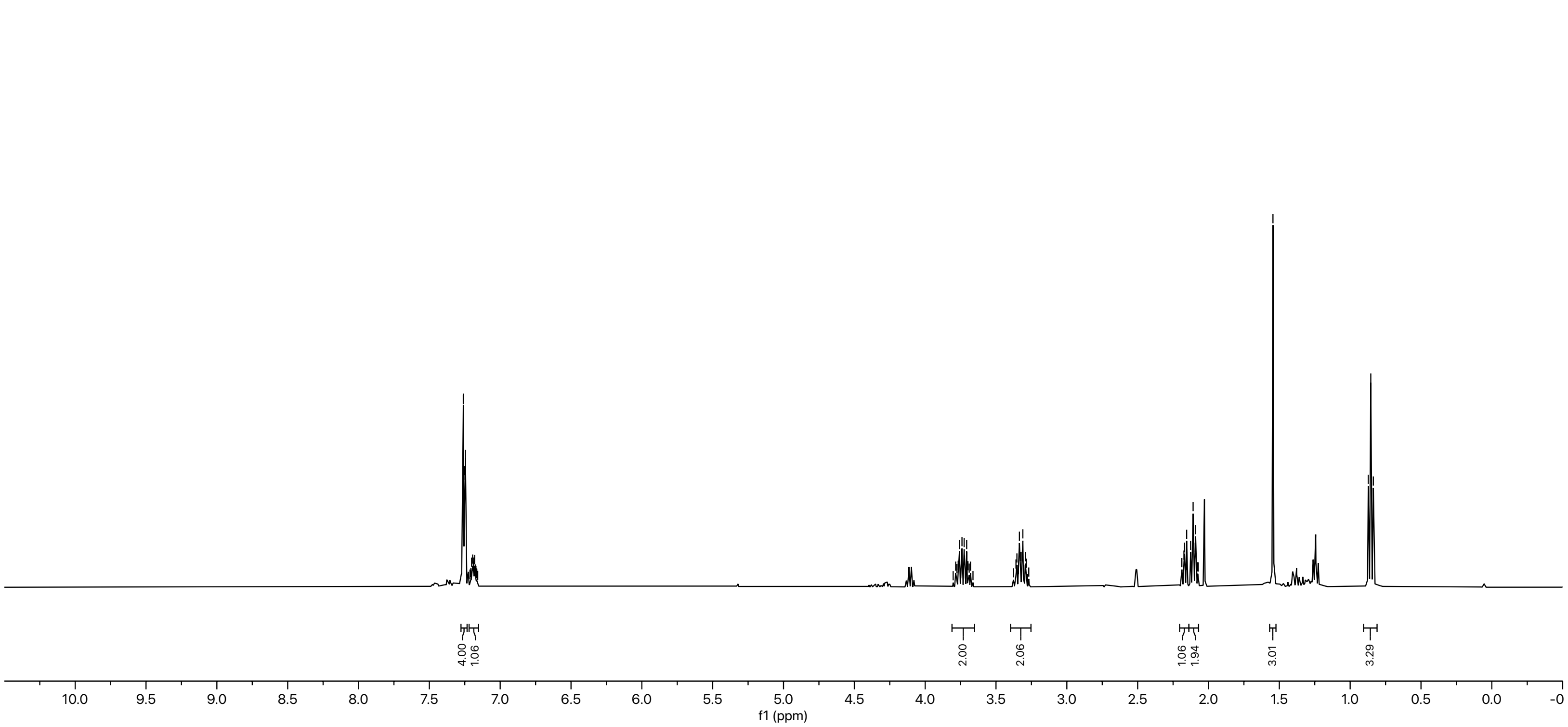

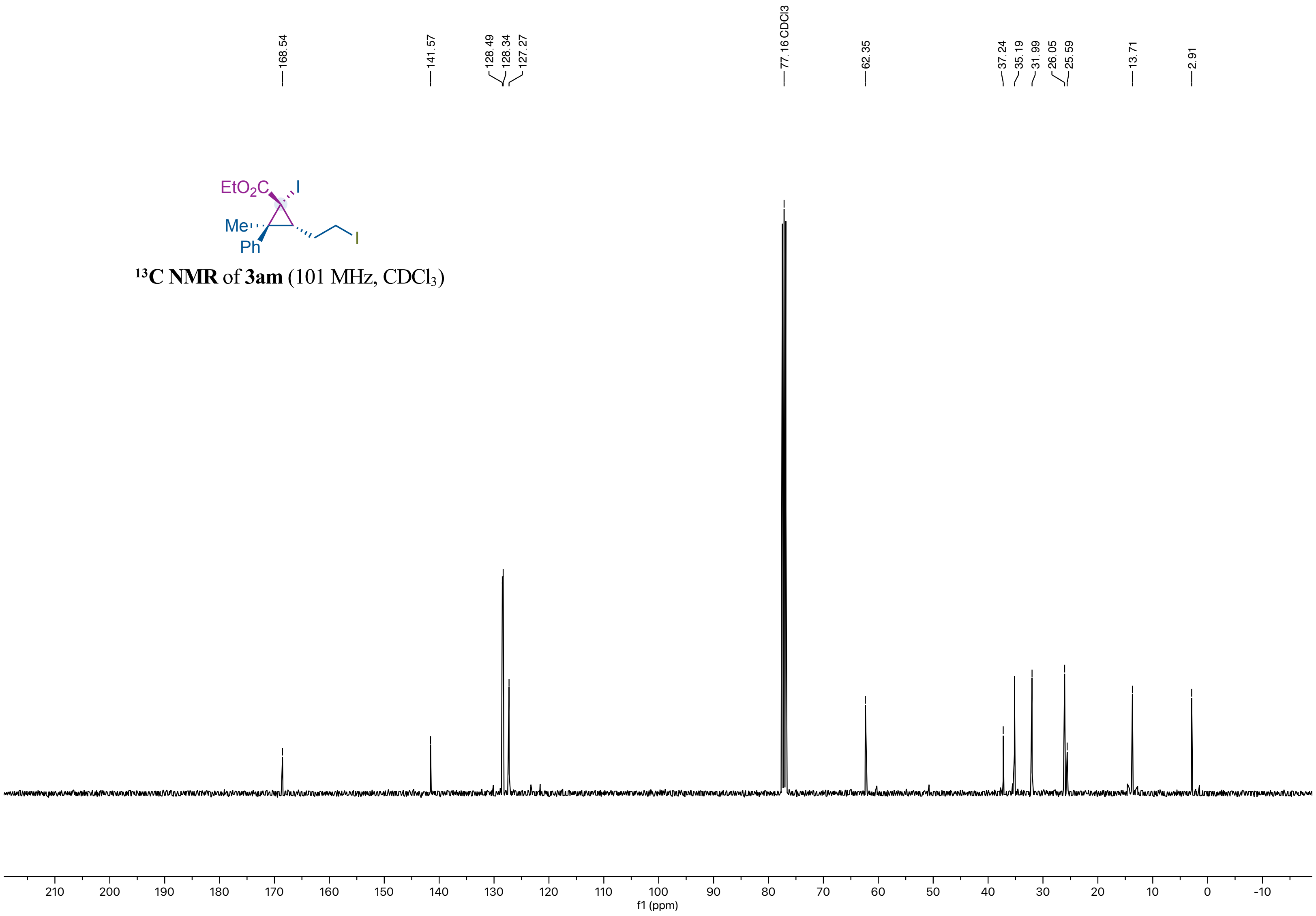

^{13}C NMR of **3am** (101 MHz, $CDCl_3$)

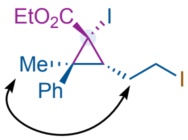
EtO2C
I
Me
Ph
I

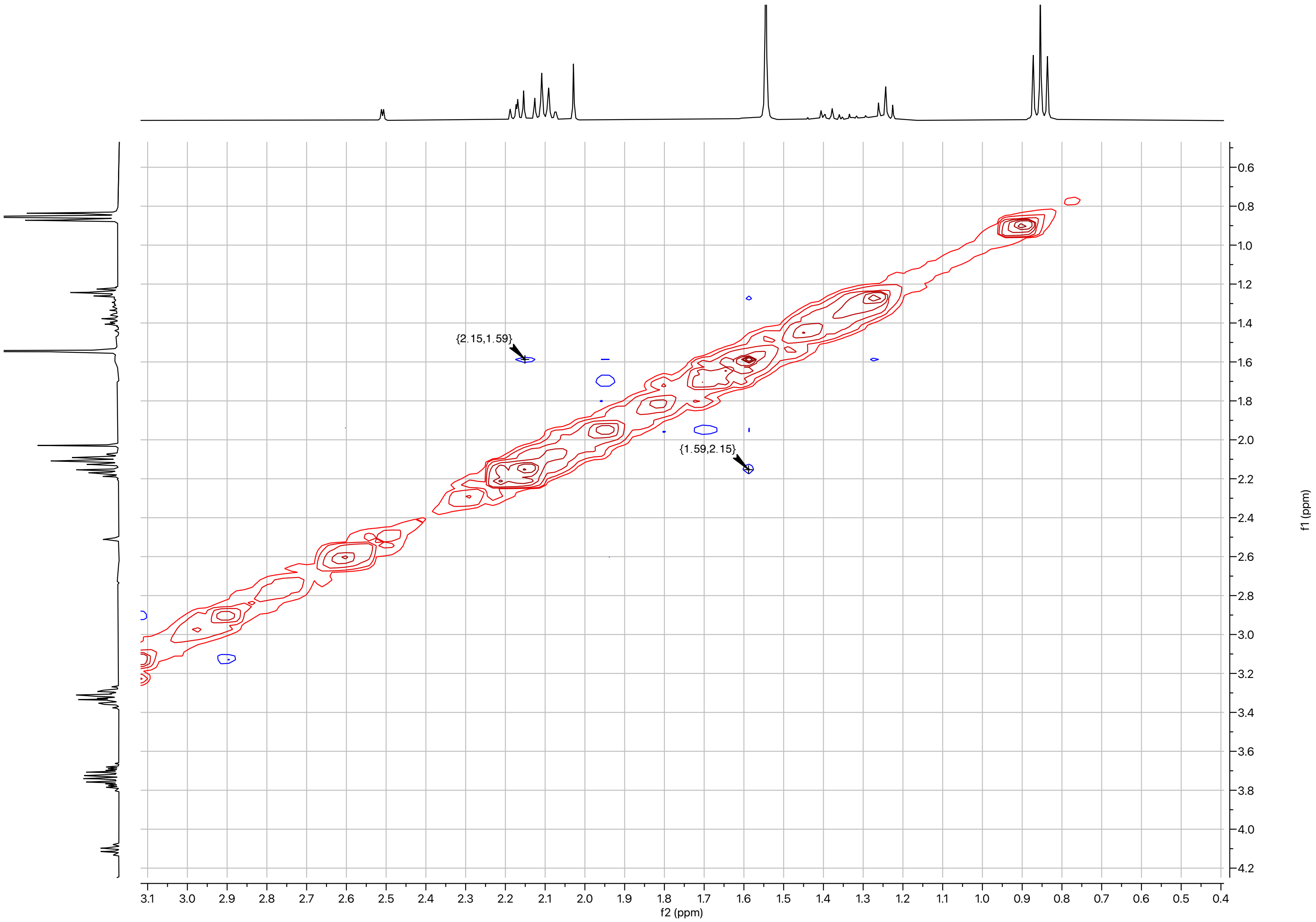
{2.15,1.59}
{1.59,2.15}
f2 (ppm)
f1 (ppm)

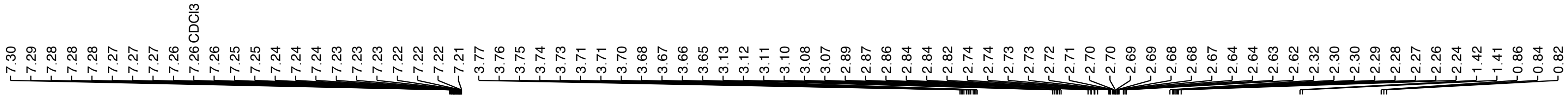

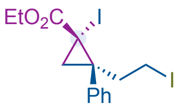

^{1}H NMR of **3an** (400 MHz, $CDCl_3$)

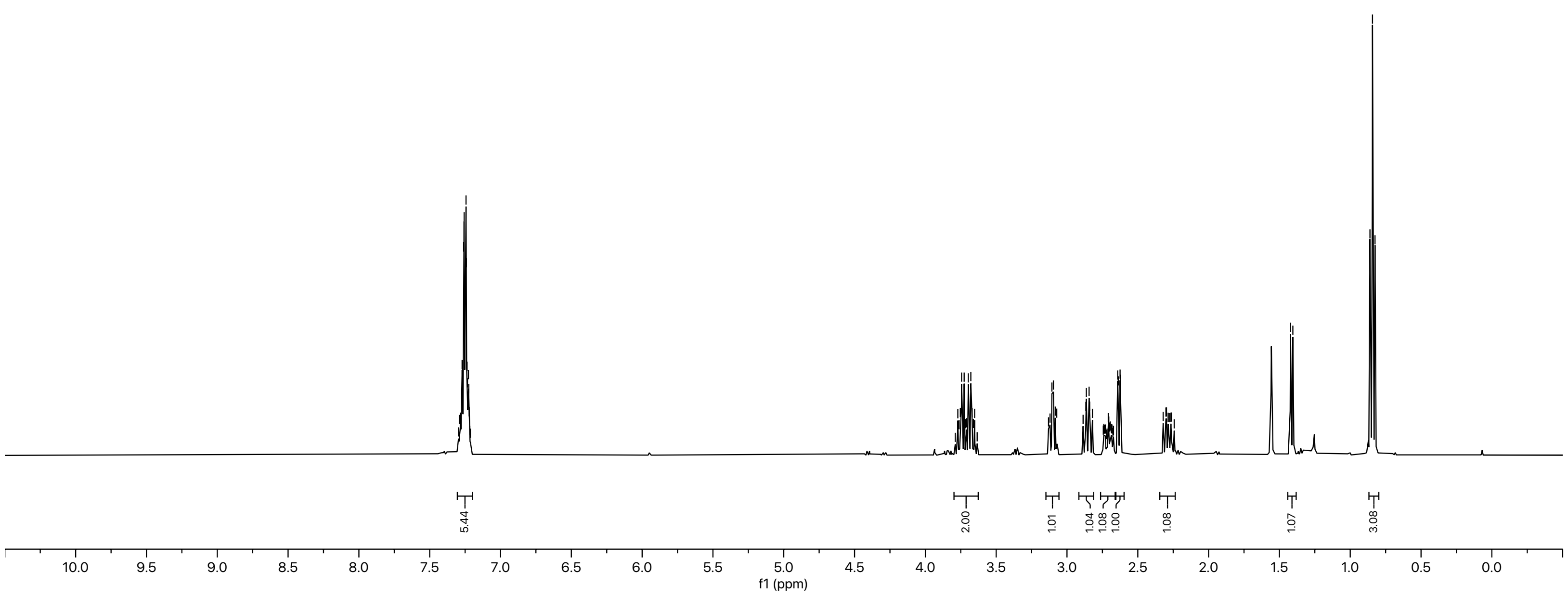

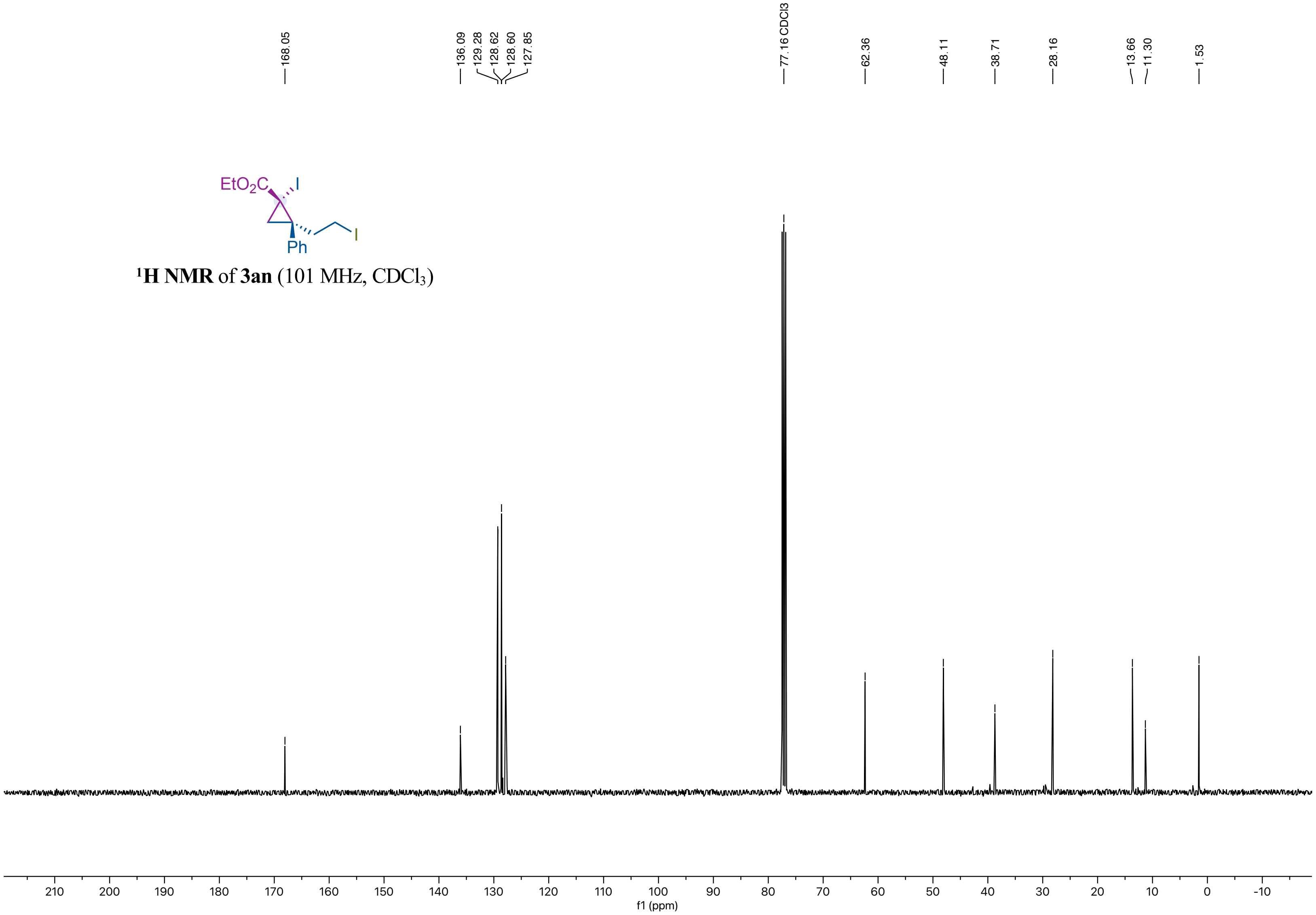

^{1}H NMR of 3an (101 MHz, $CDCl_3$)

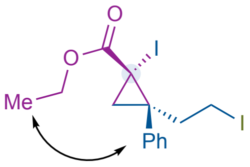
O
O
I
Me
Ph
I

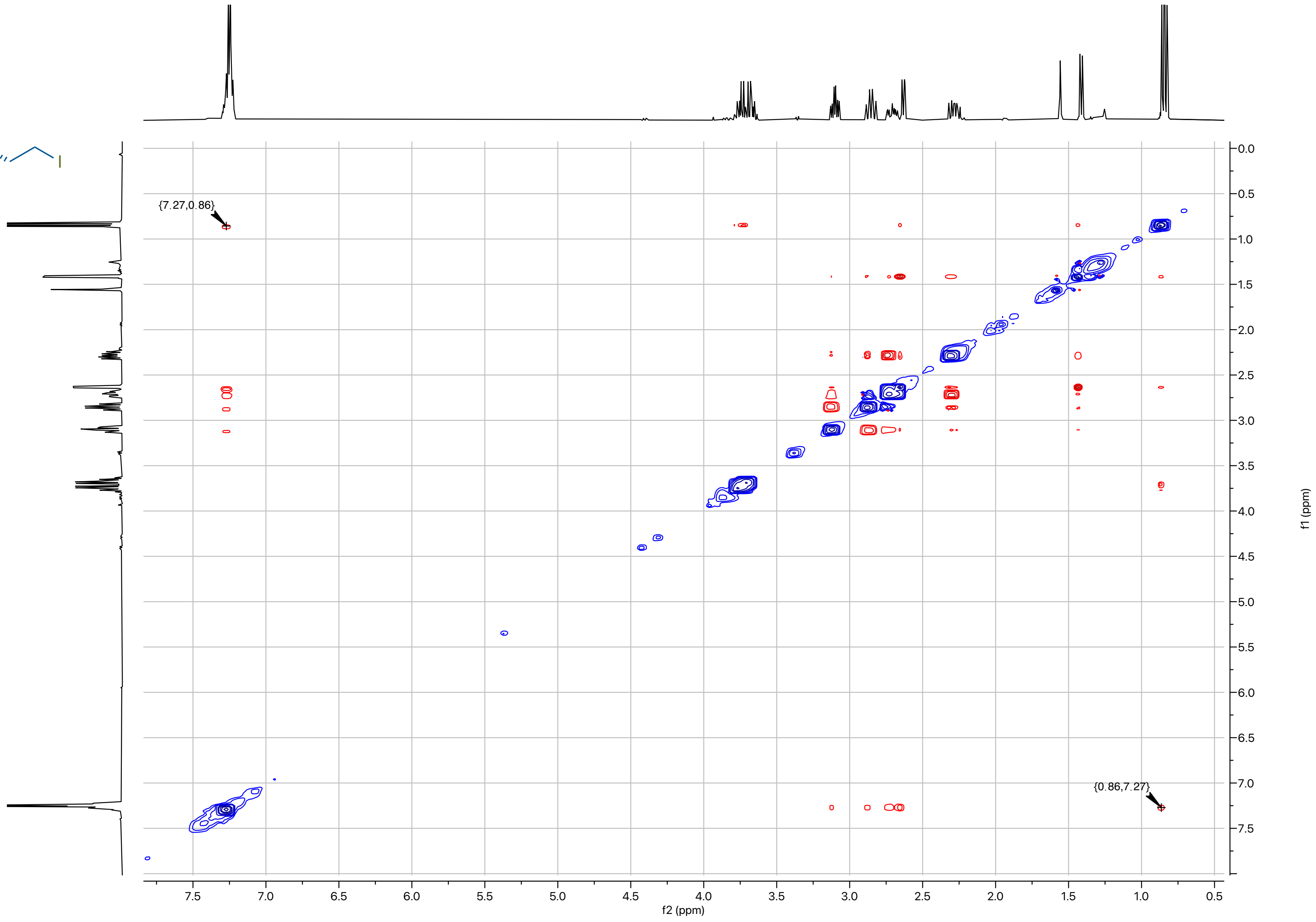
{7.27,0.86}
{0.86,7.27}
f2 (ppm)
f1 (ppm)
7.5
7.0
6.5
6.0
5.5
5.0
4.5
4.0
3.5
3.0
2.5
2.0
1.5
1.0
0.5
0.0

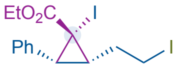

^{1}H NMR of **3ao** (300 MHz, $CDCl_3$)

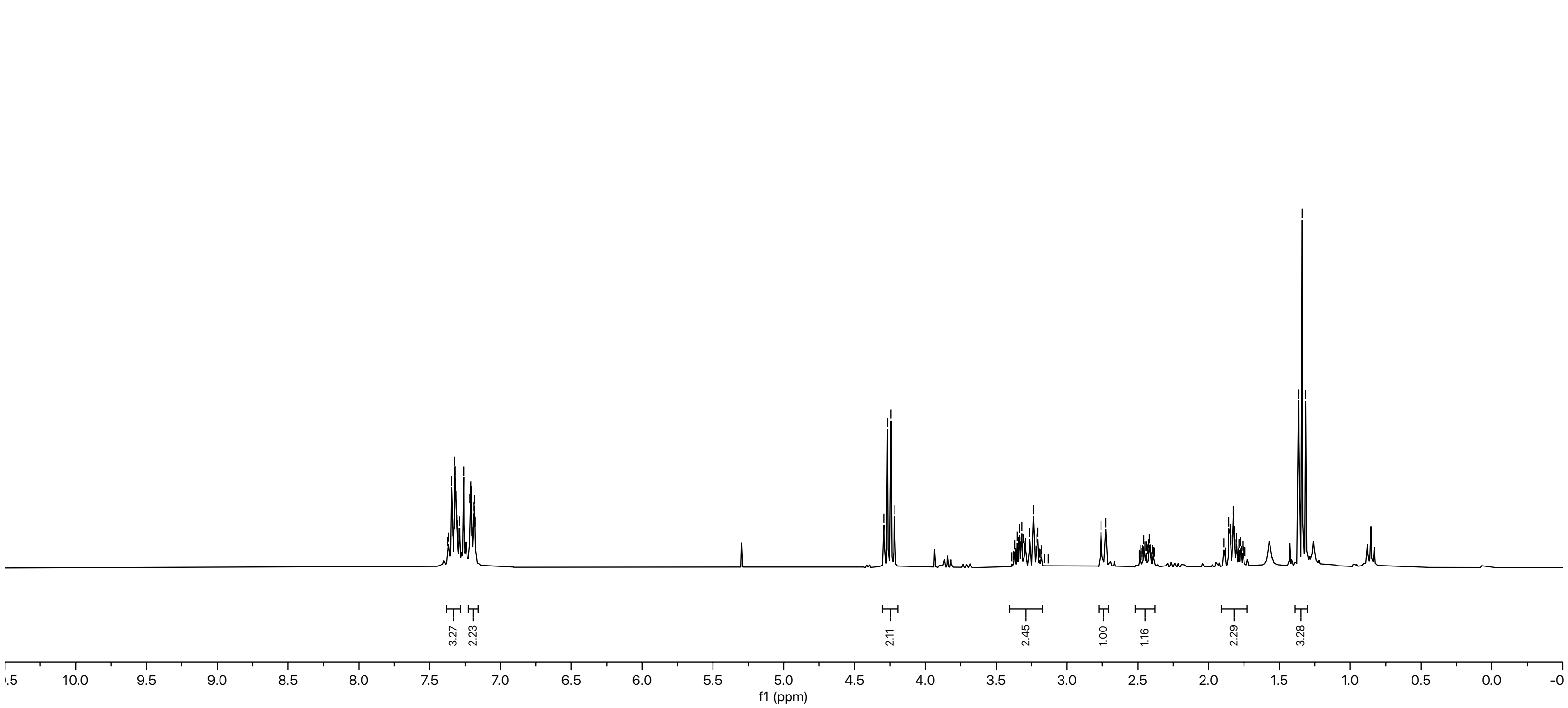

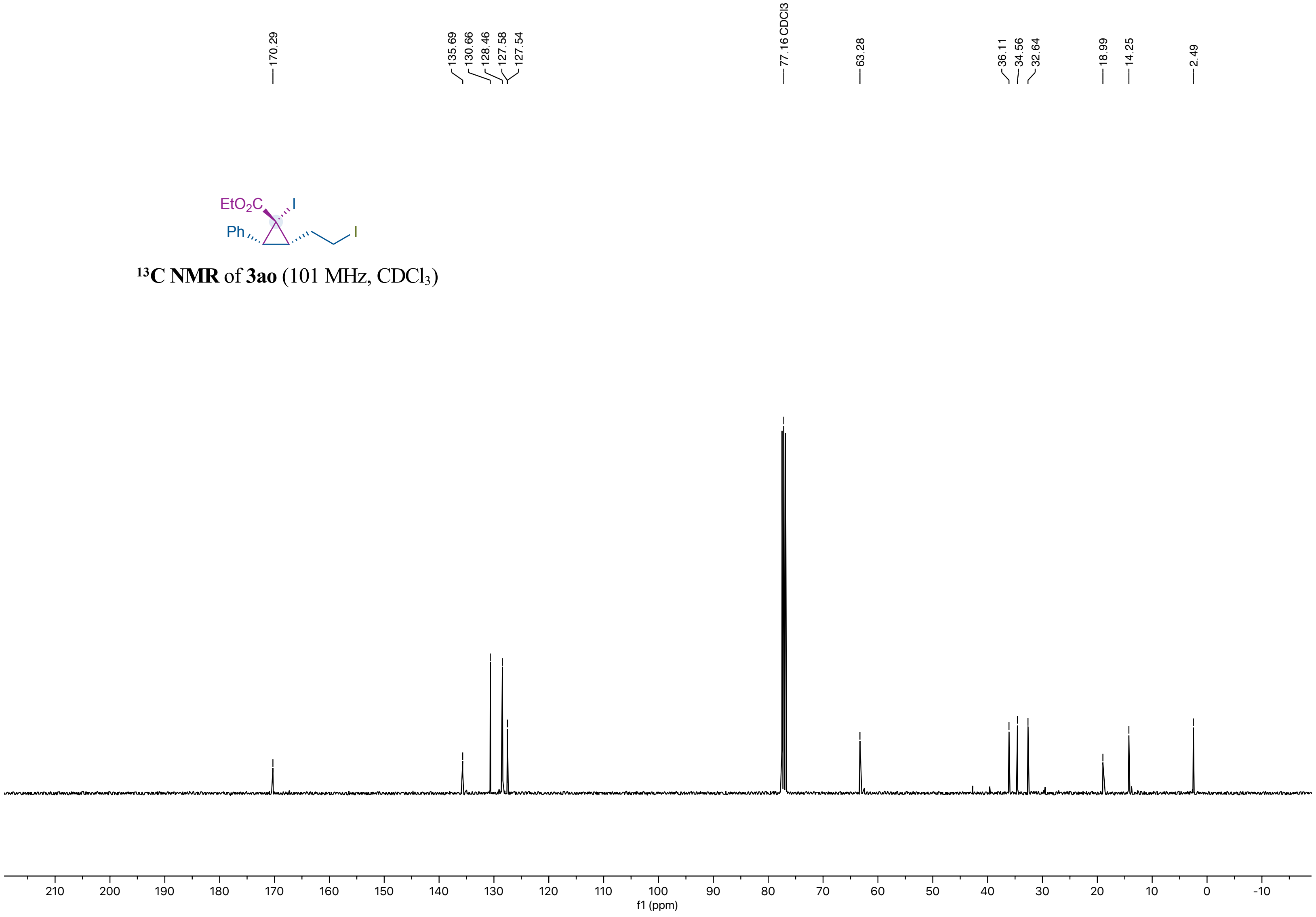

^{13}C NMR of **3ao** (101 MHz, $CDCl_3$)

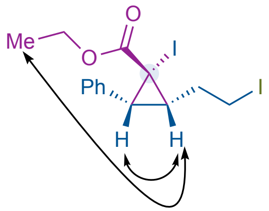

Me
O
O
I
Ph
I
H
H

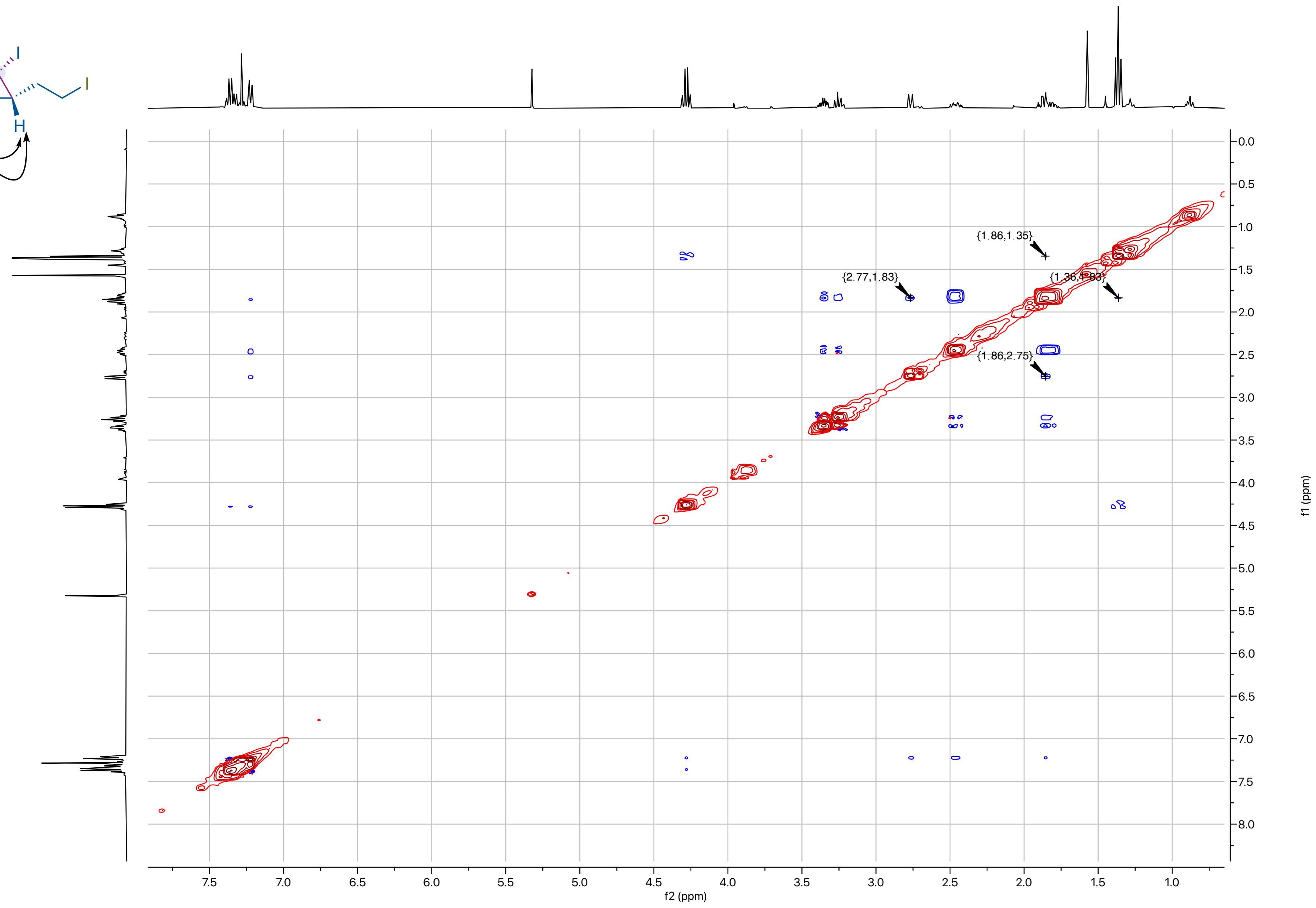

{1.86,1.35}
{2.77,1.83}
{1.36,1.83}
{1.86,2.75}
f2 (ppm)
f1 (ppm)

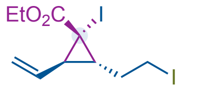

^{1}H NMR of **3ap** (500 MHz, $CDCl_3$)

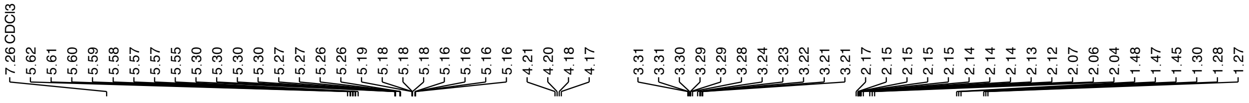
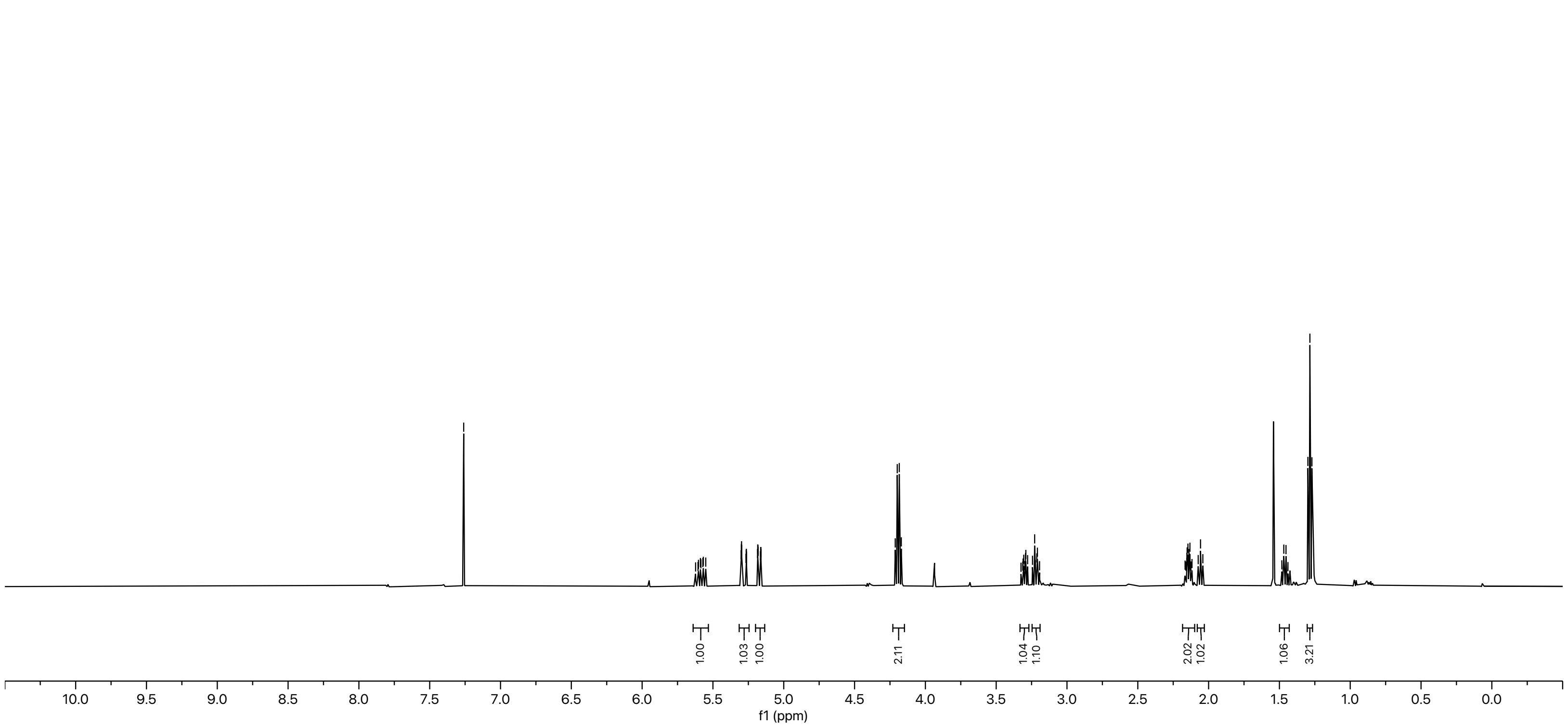

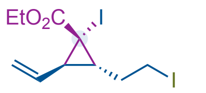

^{13}**C NMR** of **3ap** (126 MHz, $CDCl_3$)

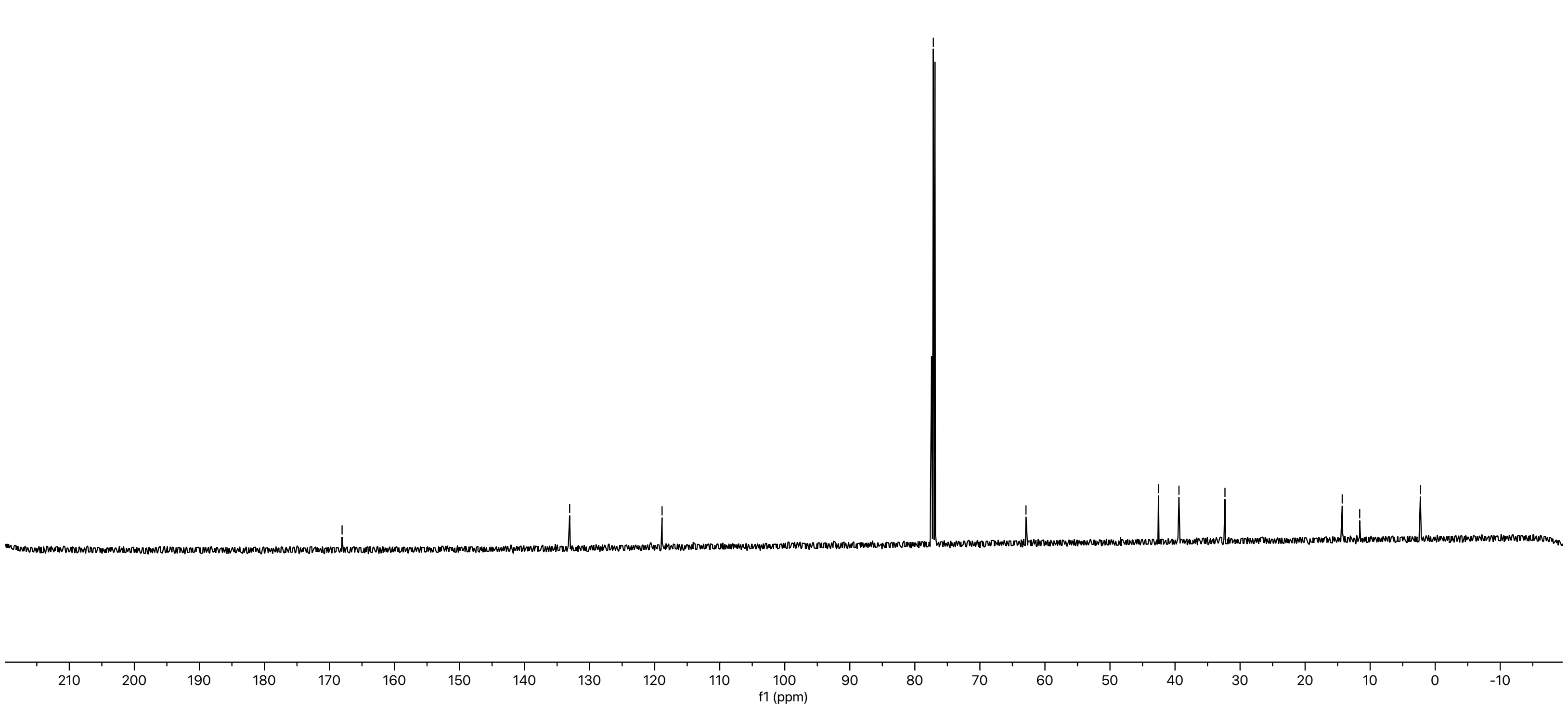

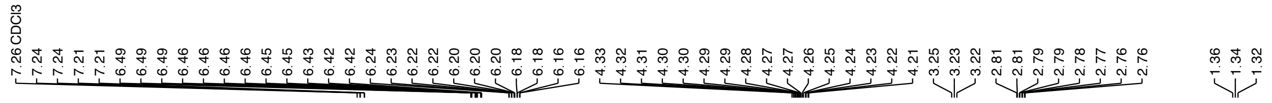

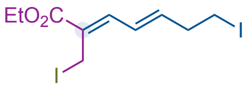

^{1}H NMR of **3ap*** (400 MHz, $CDCl_3$)

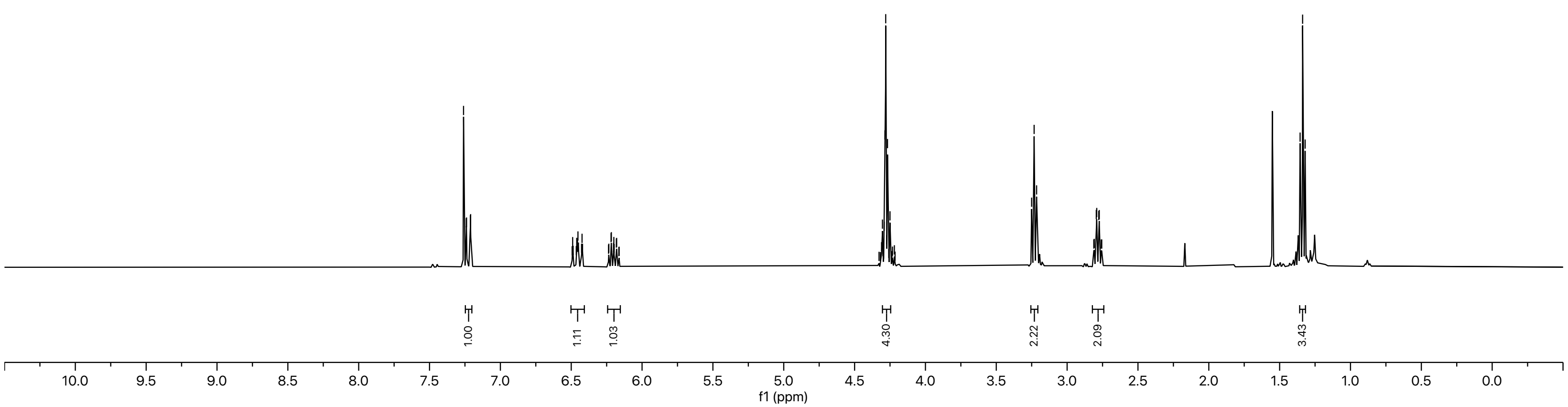

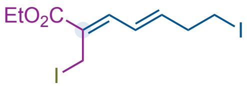

^{13}C NMR of **3ap*** (126 MHz, $CDCl_3$)

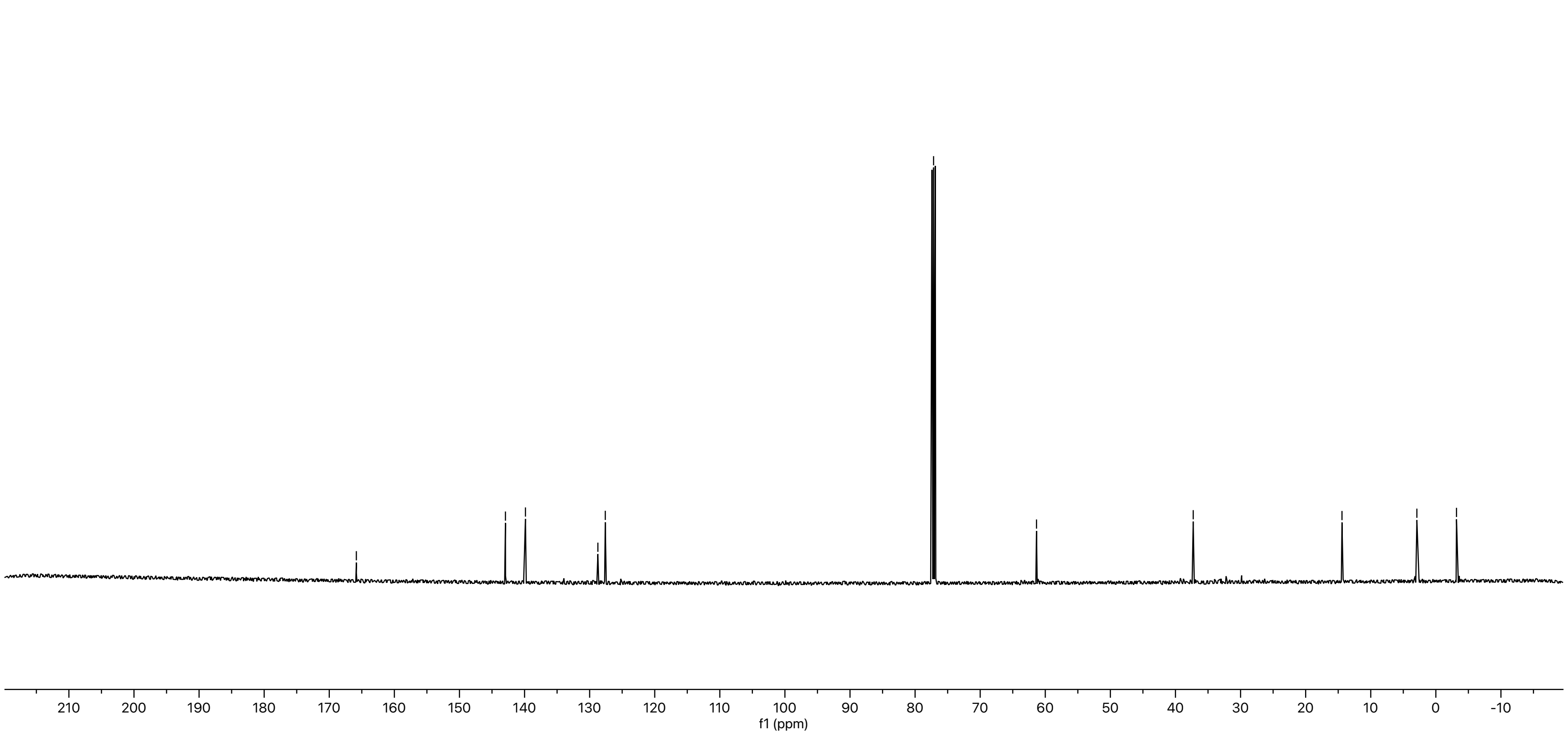

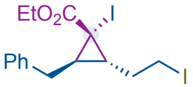

¹H NMR of **3aq** (400 MHz, $CDCl_3$)

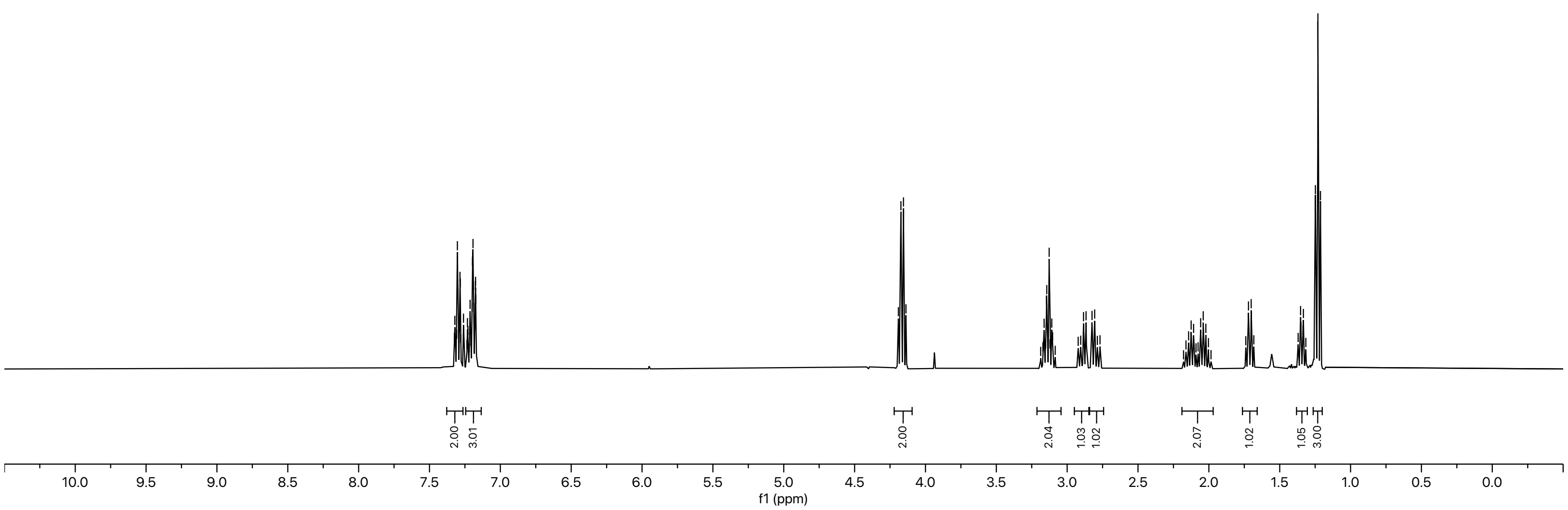

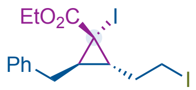

^{13}**C NMR** of **3aq** (101 MHz, $CDCl_3$)

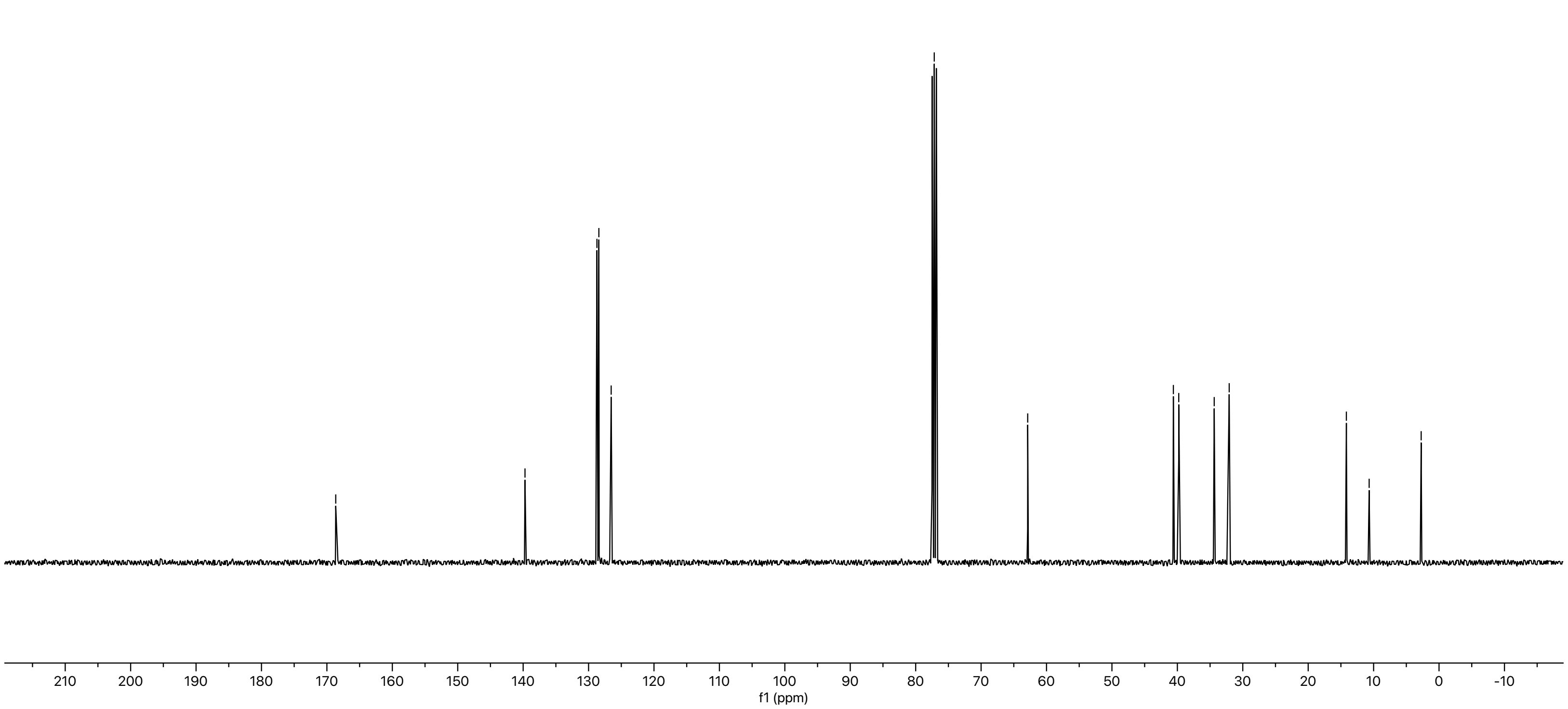

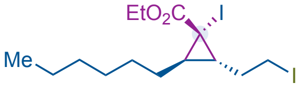

^{1}H NMR of **3ar** (400 MHz, $CDCl_3$)

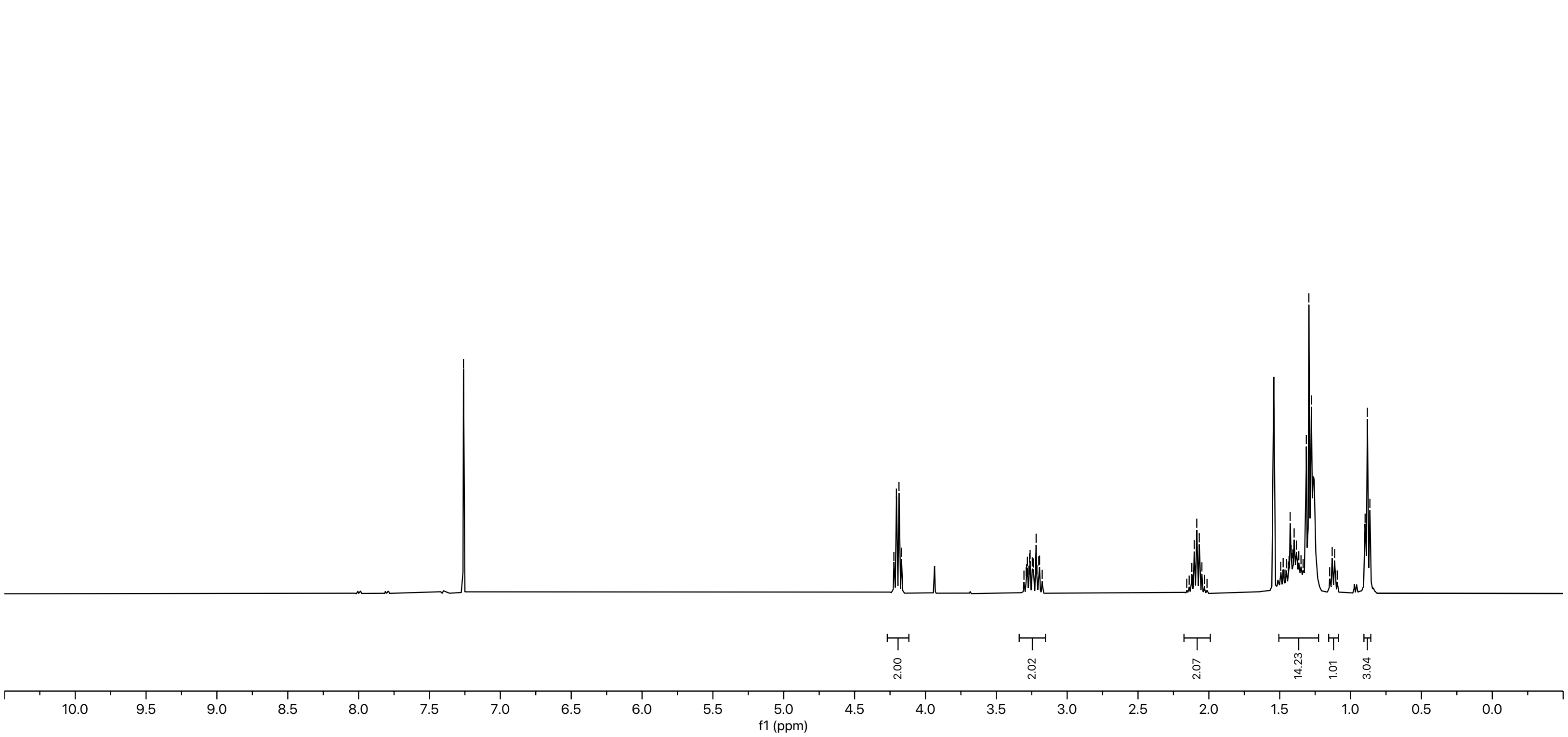

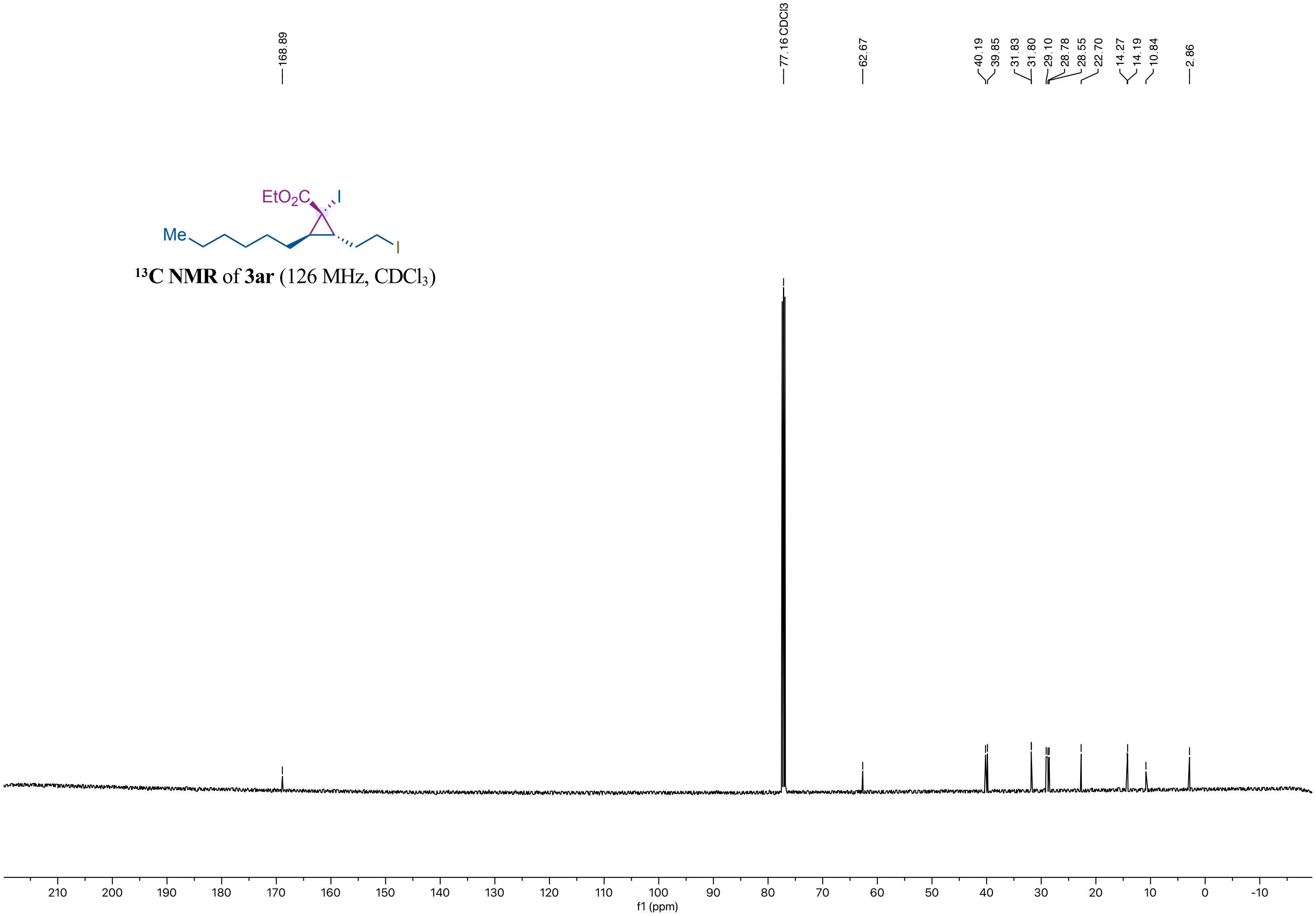

^{13}**C NMR** of **3ar** (126 MHz, $CDCl_3$)

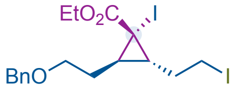

^{1}H NMR of **3as** (400 MHz, $CDCl_3$)

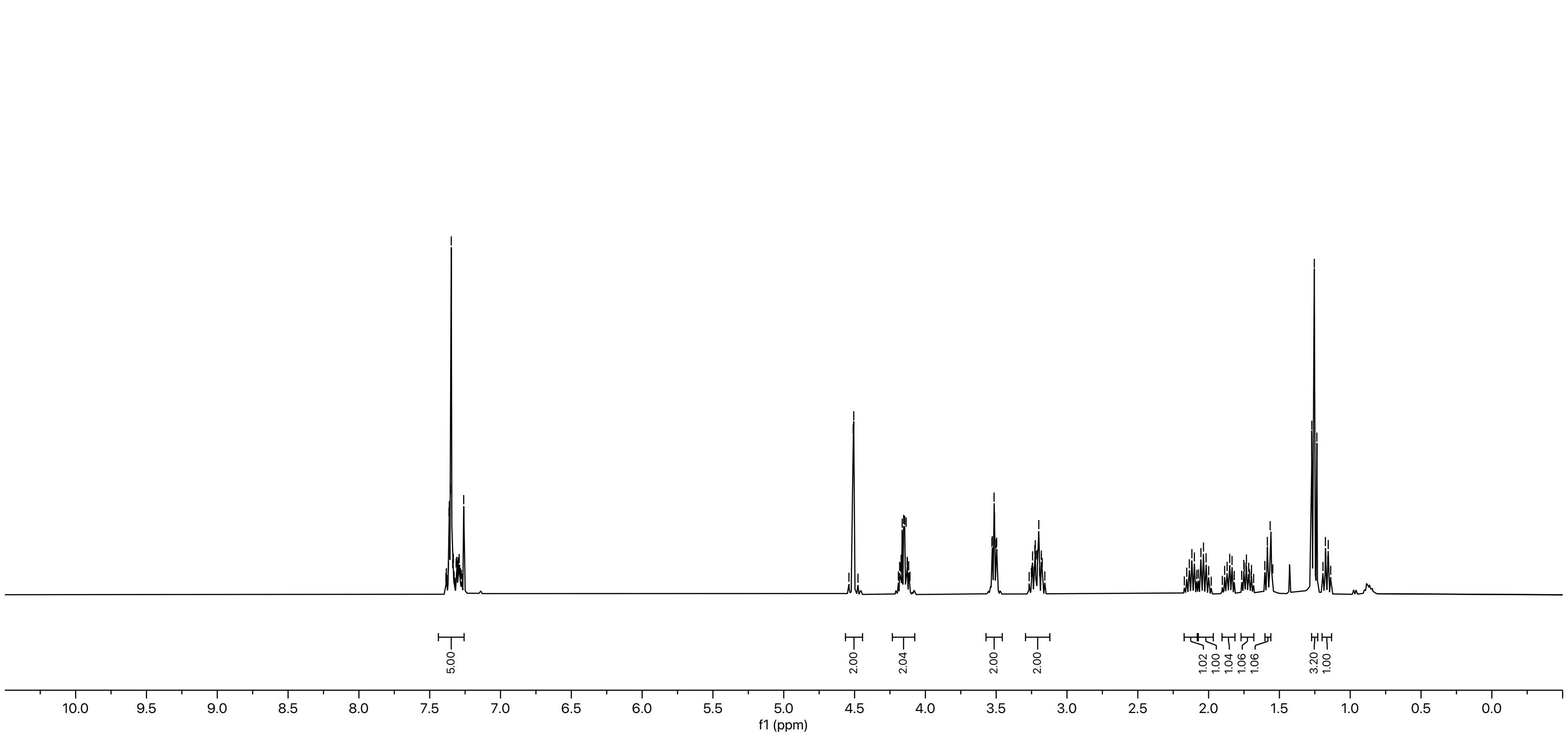

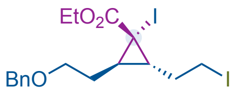

^{13}**C NMR** of **3as** (101 MHz, $CDCl_3$)

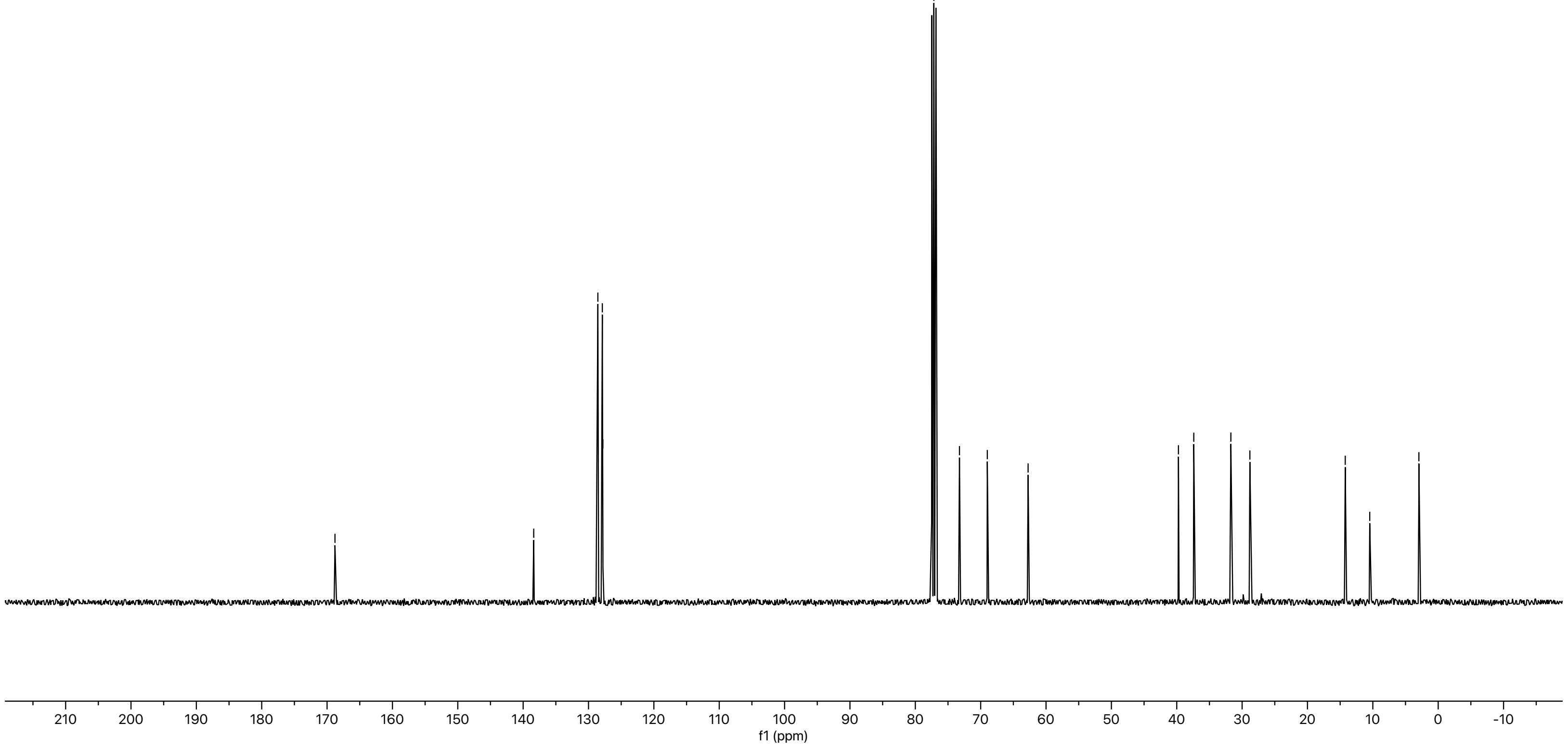

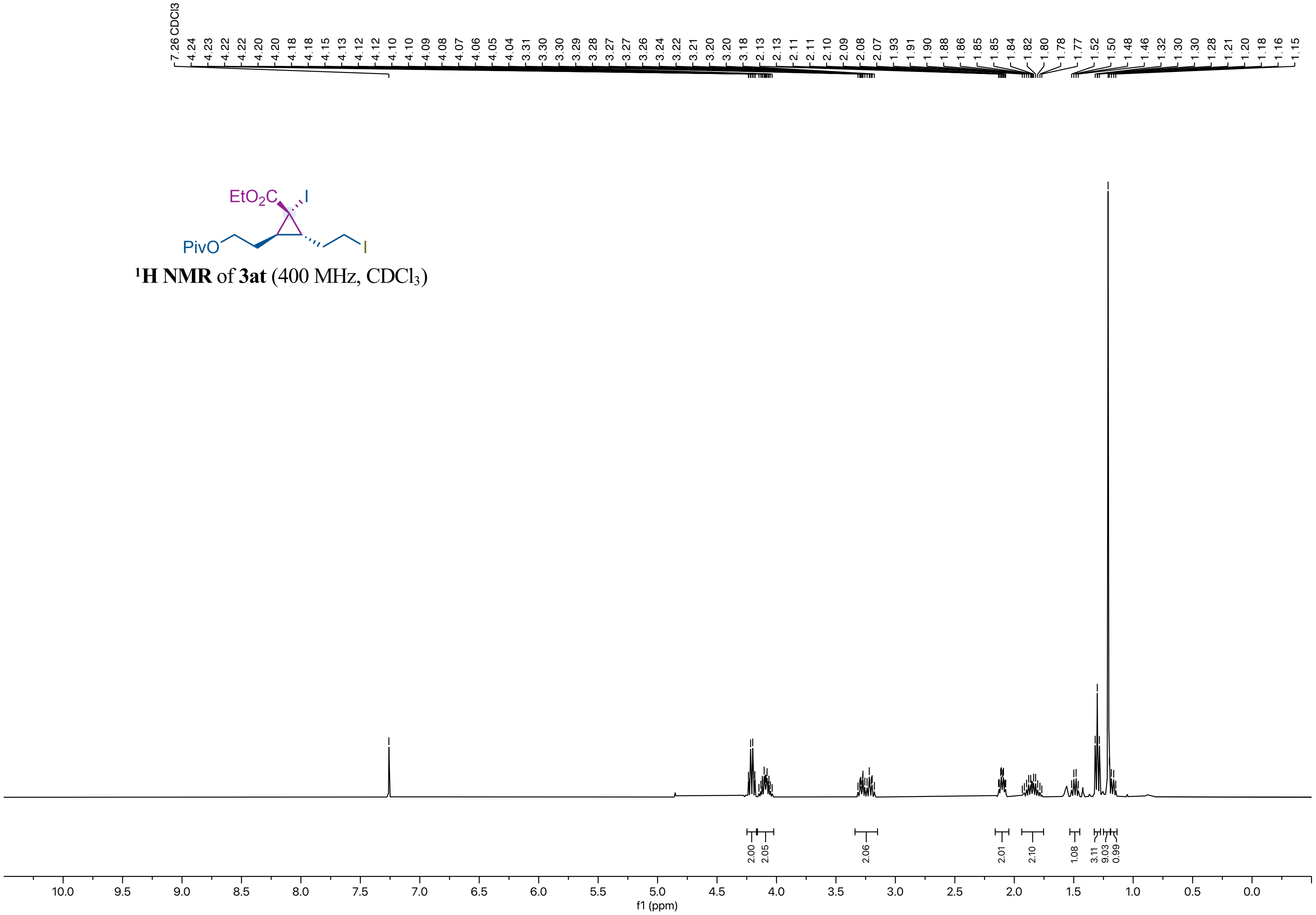

^{1}H NMR of **3at** (400 MHz, $CDCl_3$)

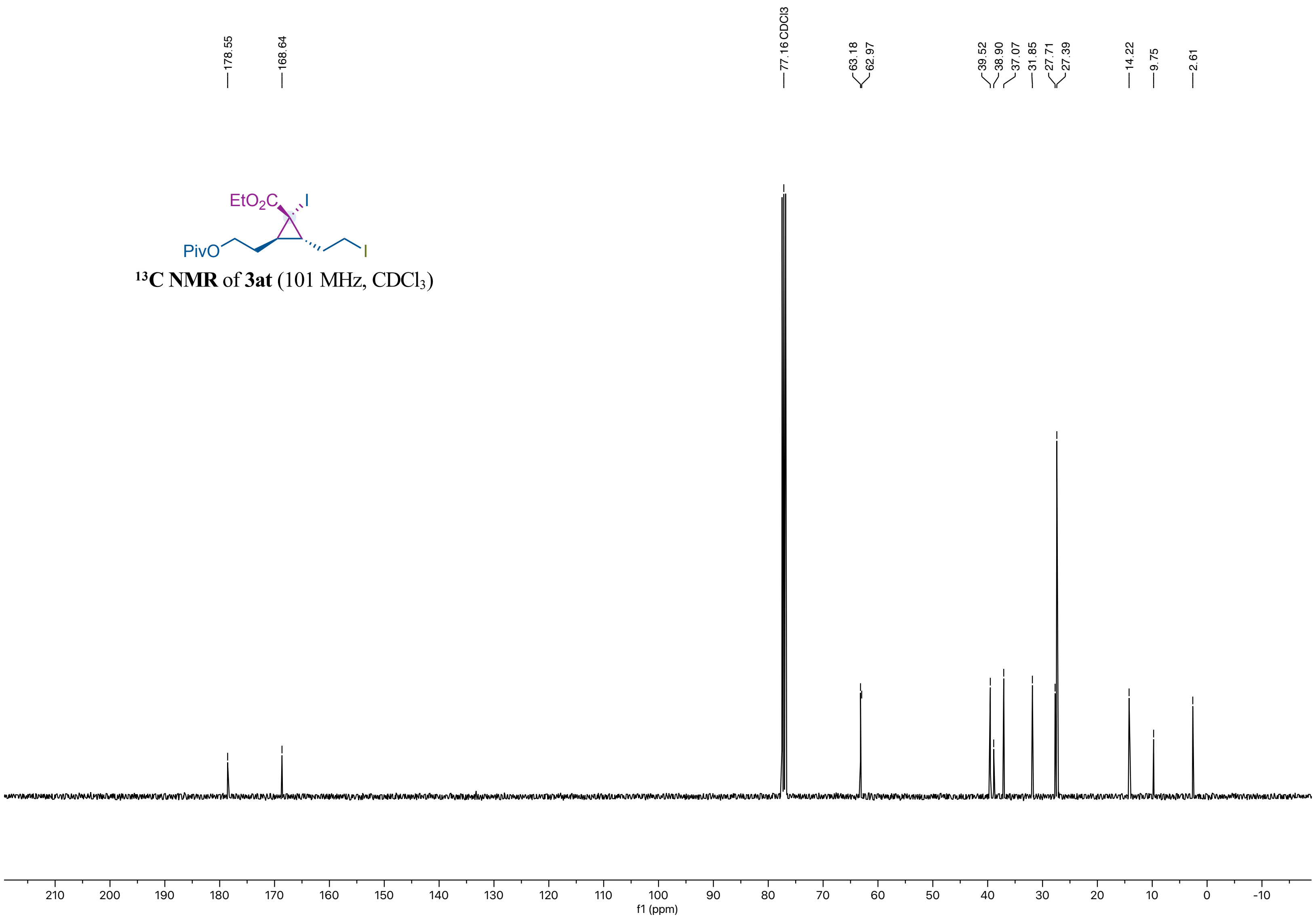

^{13}**C NMR** of **3at** (101 MHz, $CDCl_3$)

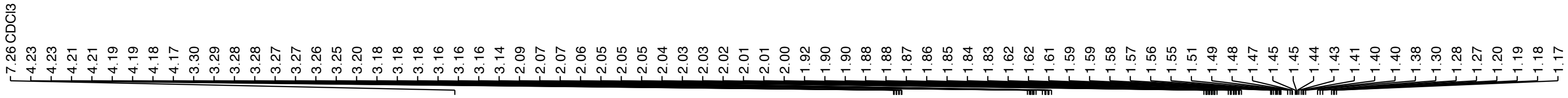

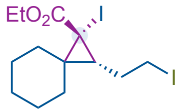

^{1}H NMR of **3au** (400 MHz, $CDCl_3$)

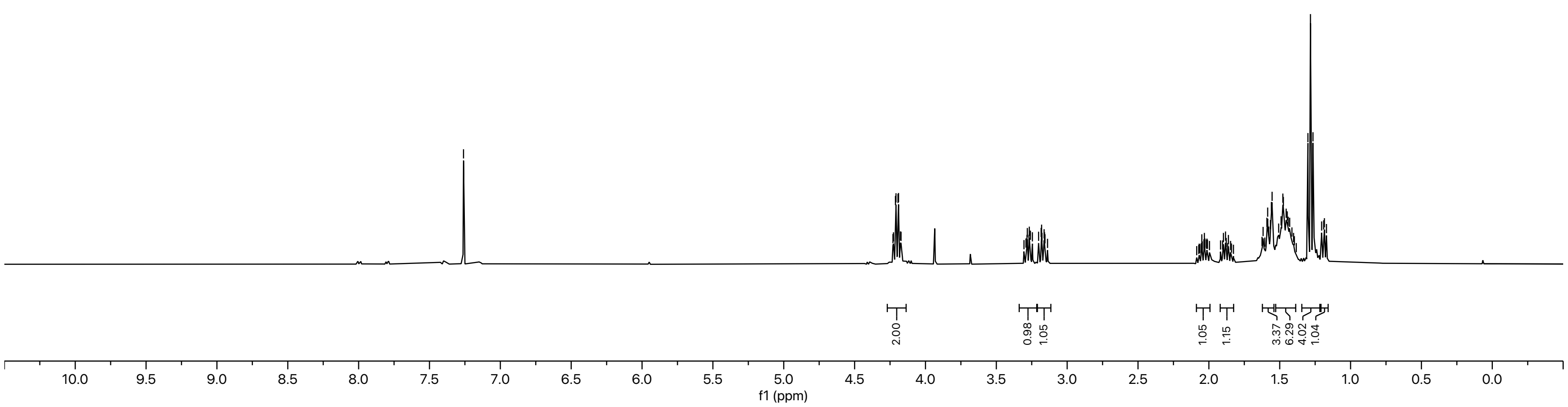

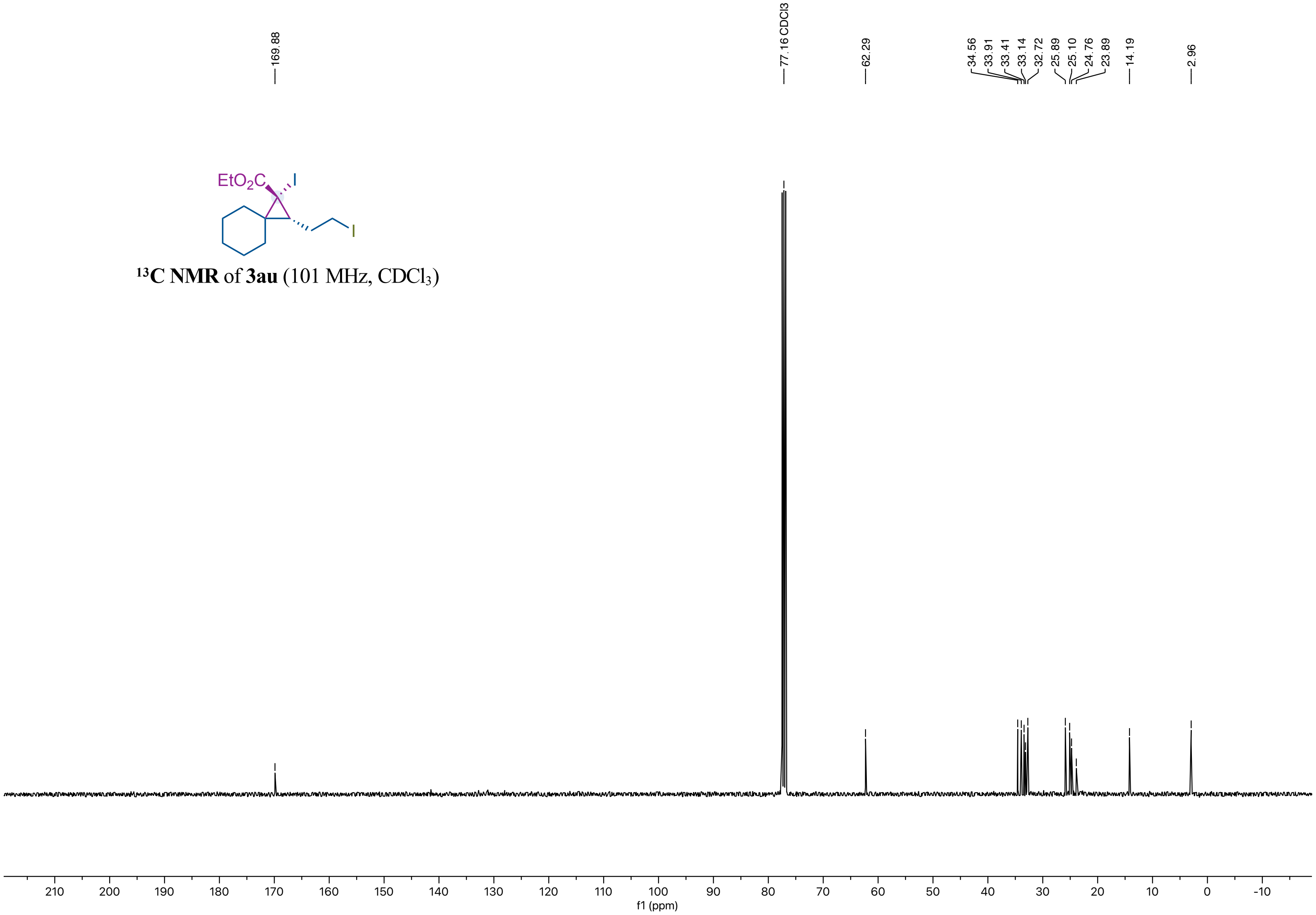

^{13}**C NMR** of **3au** (101 MHz, $CDCl_3$)

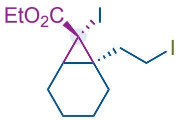

¹H NMR of **3av** (400 MHz, $CDCl_3$)

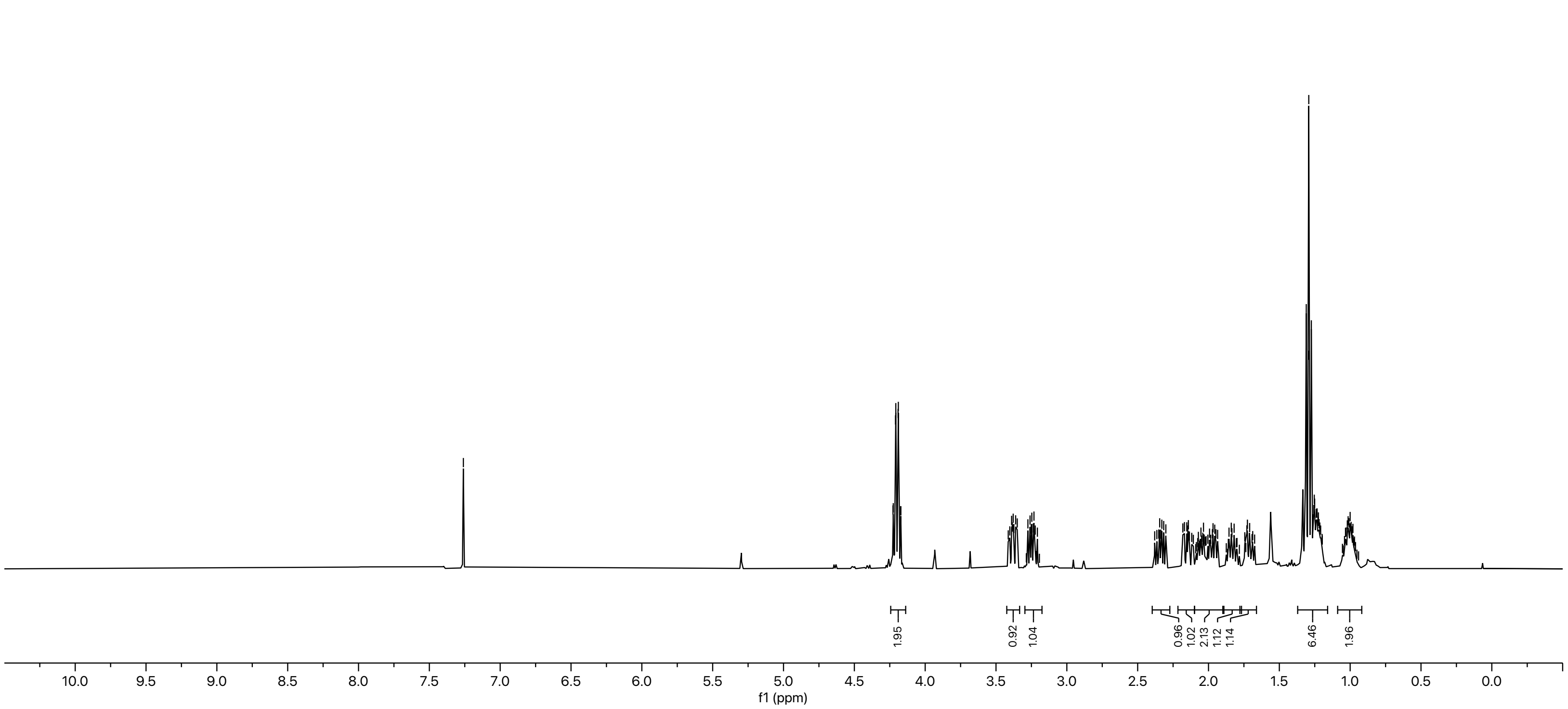

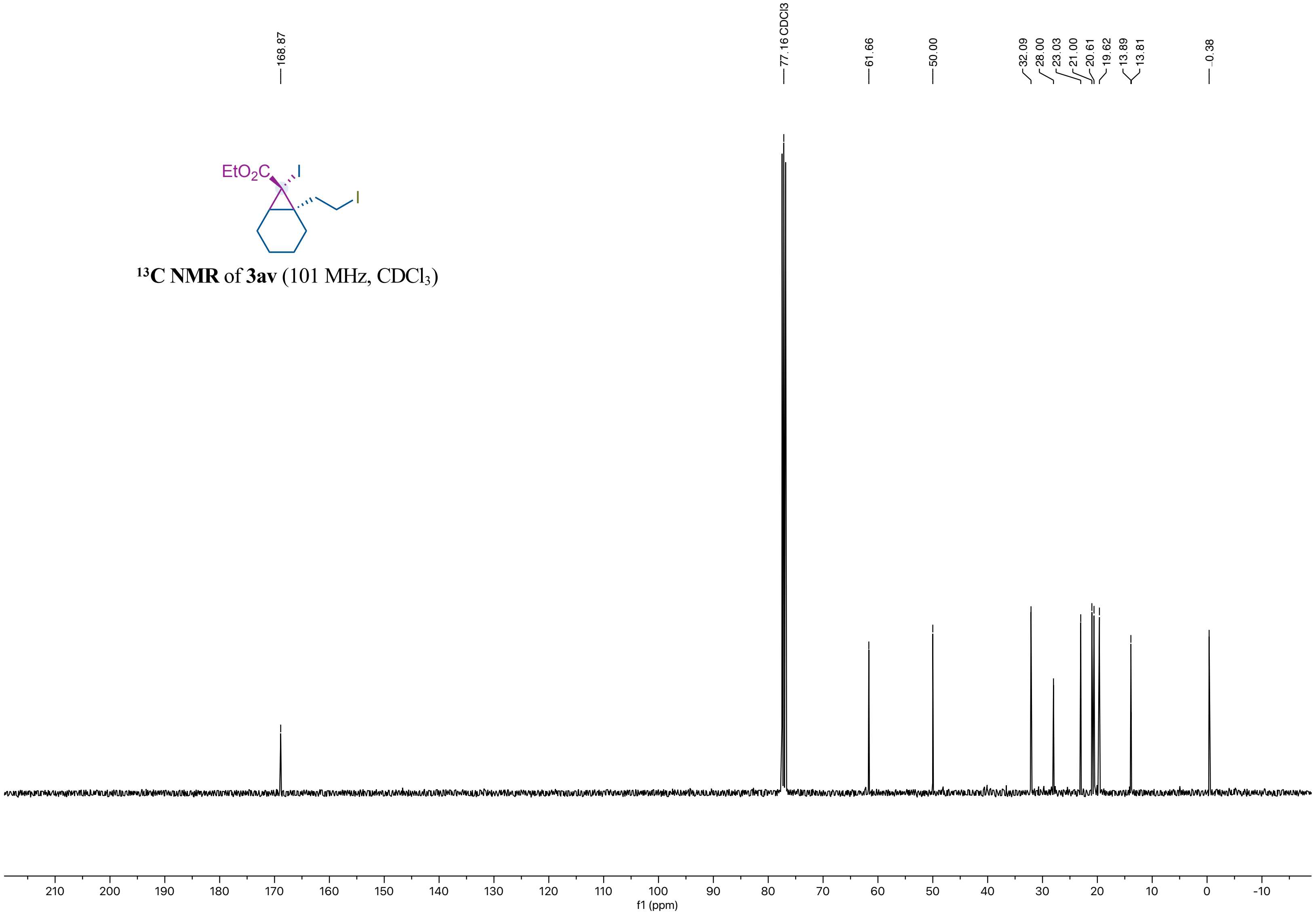

^{13}C NMR of **3av** (101 MHz, $CDCl_3$)

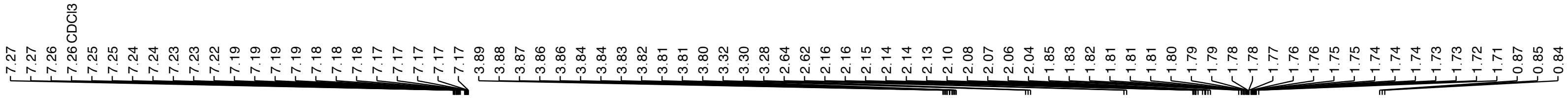

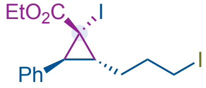

^{1}H NMR of **3ay** (400 MHz, $CDCl_3$)

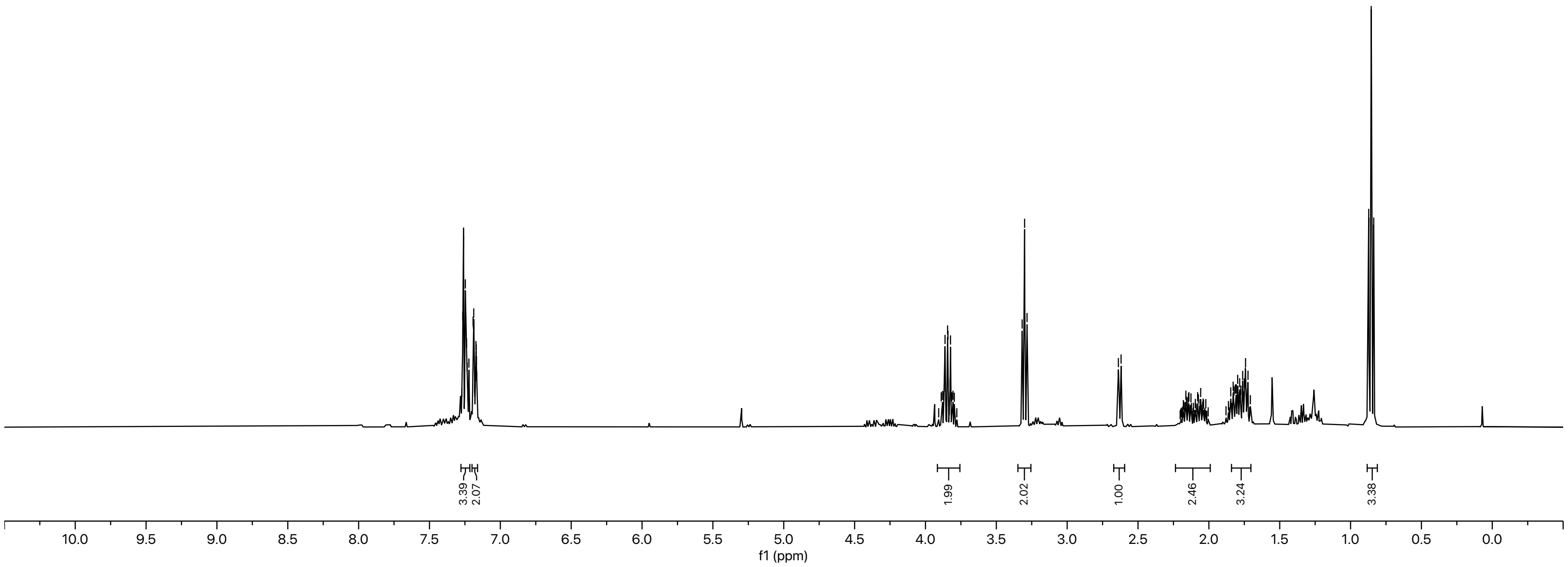

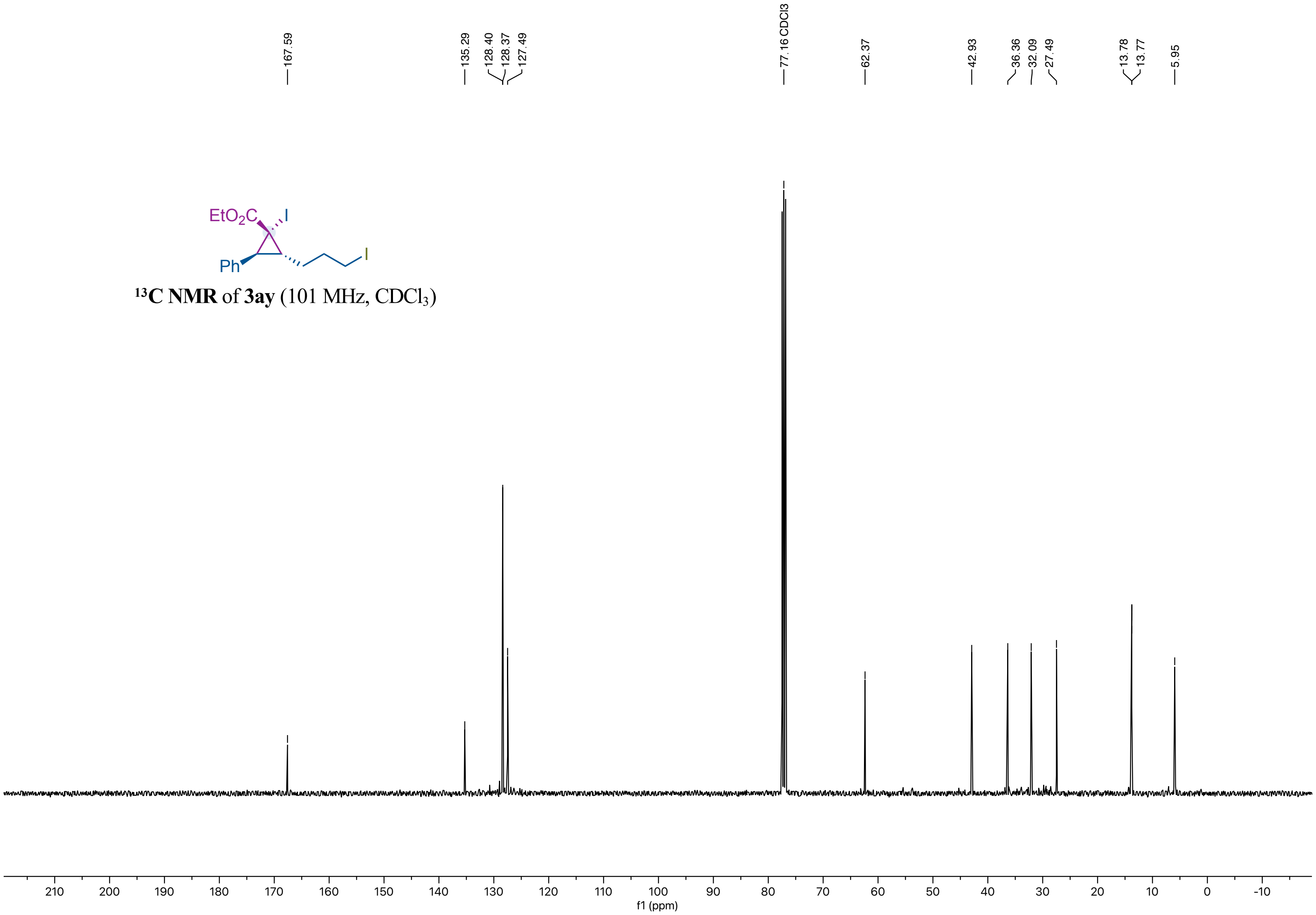

^{13}C NMR of **3ay** (101 MHz, $CDCl_3$)

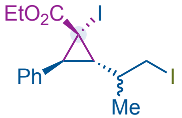

^{1}**H NMR** of **3ba** (500 MHz, $CDCl_3$)

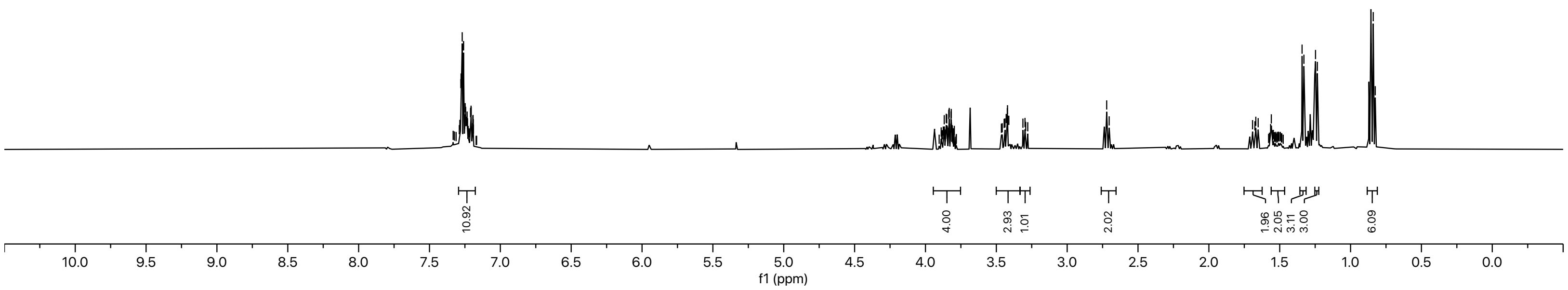

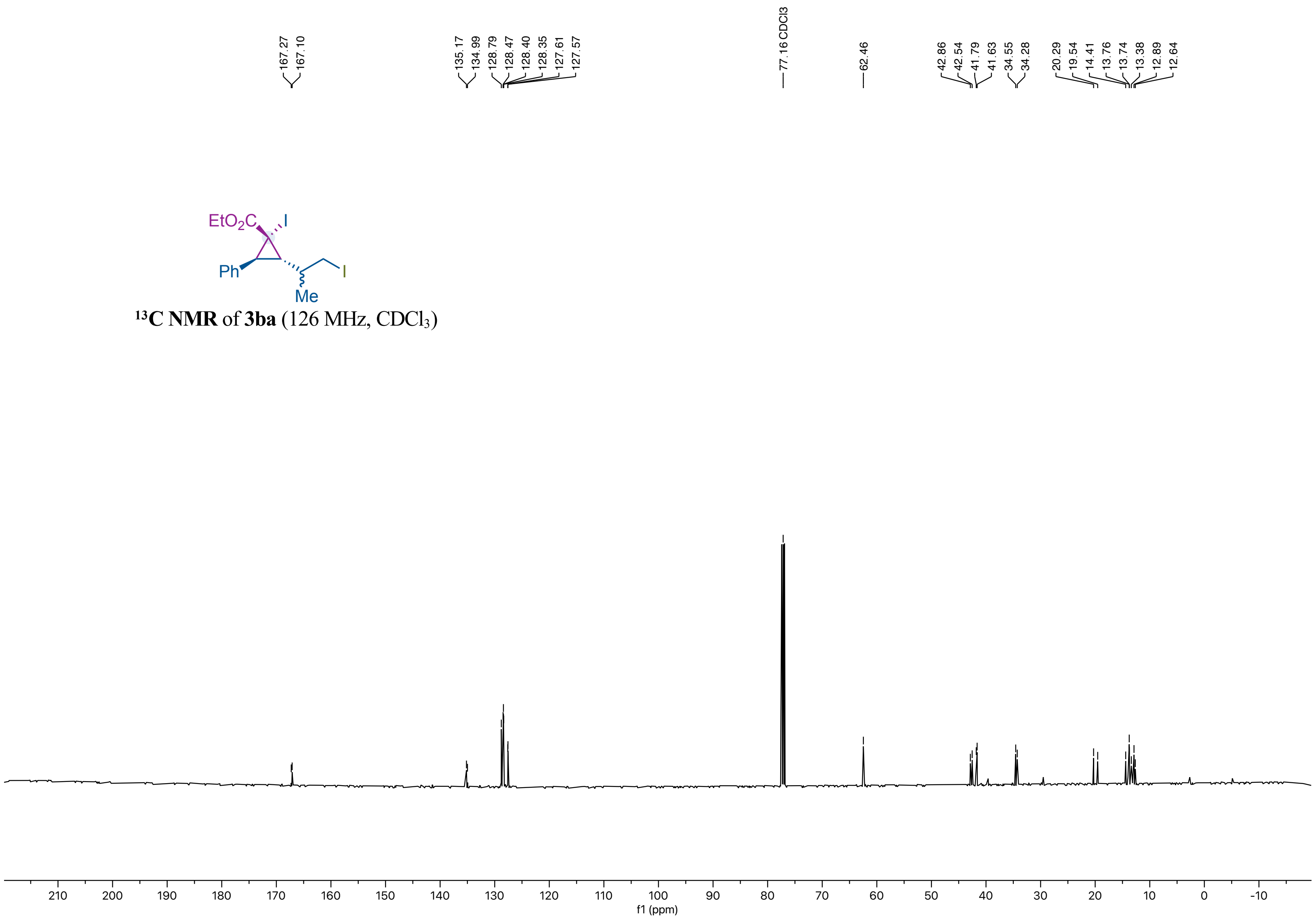

^{13}C NMR of **3ba** (126 MHz, $CDCl_3$)

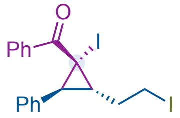

^{1}H NMR of **3bc** (400 MHz, $CDCl_3$)

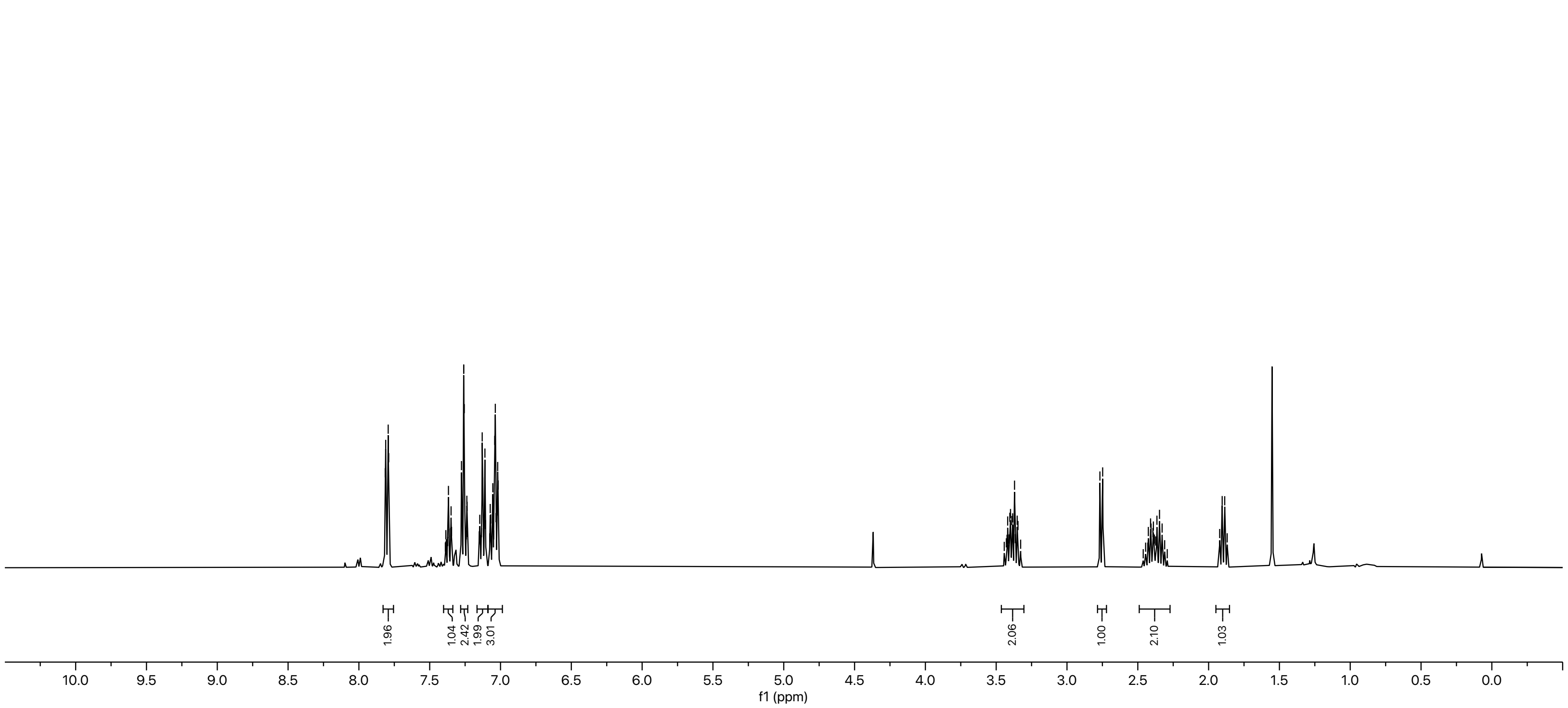

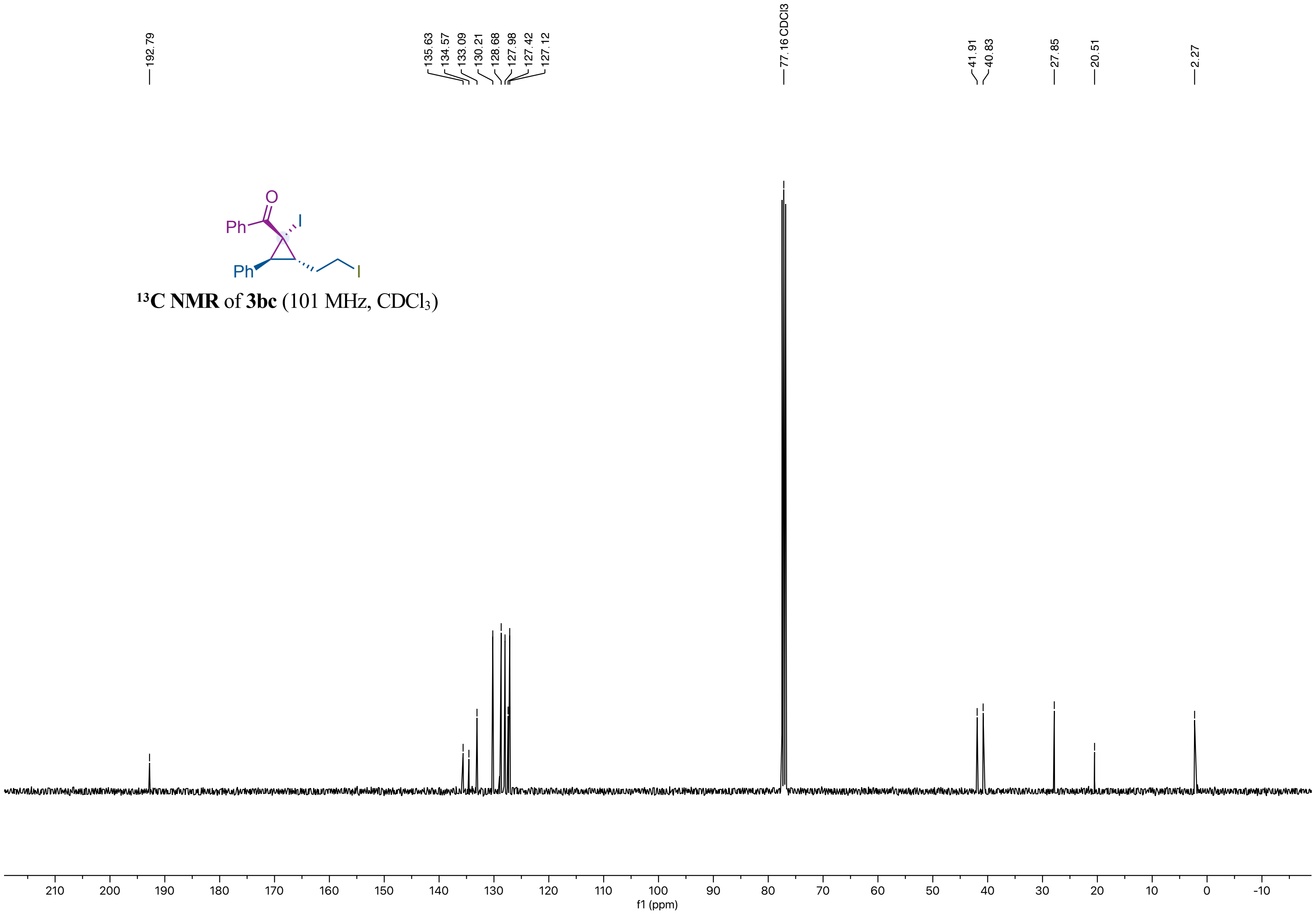

^{13}C NMR of **3bc** (101 MHz, $CDCl_3$)

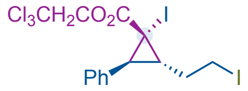

^{1}**H NMR** of **3bd** (500 MHz, $CDCl_3$)

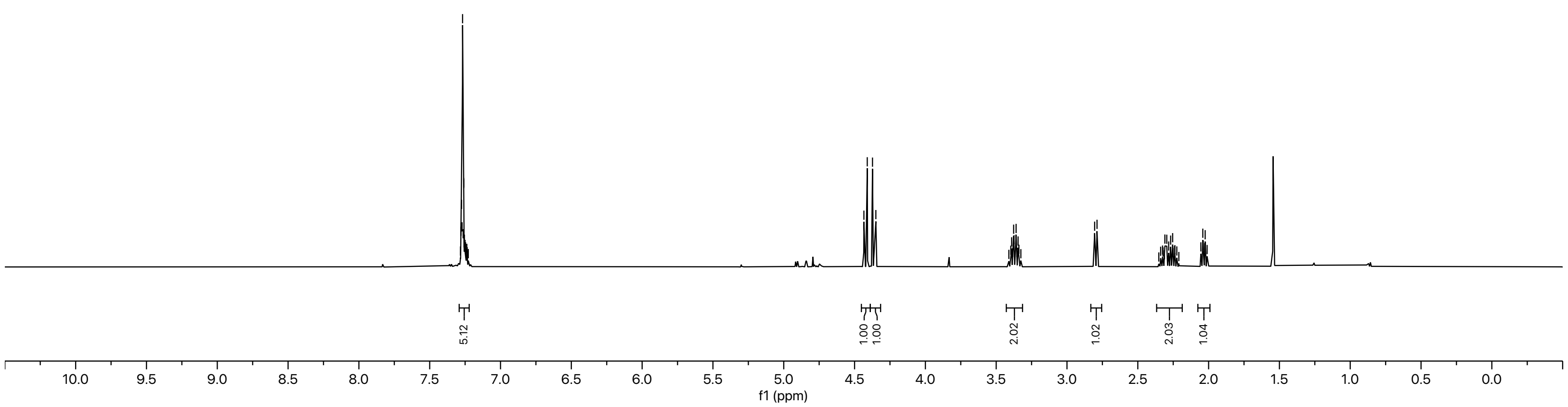

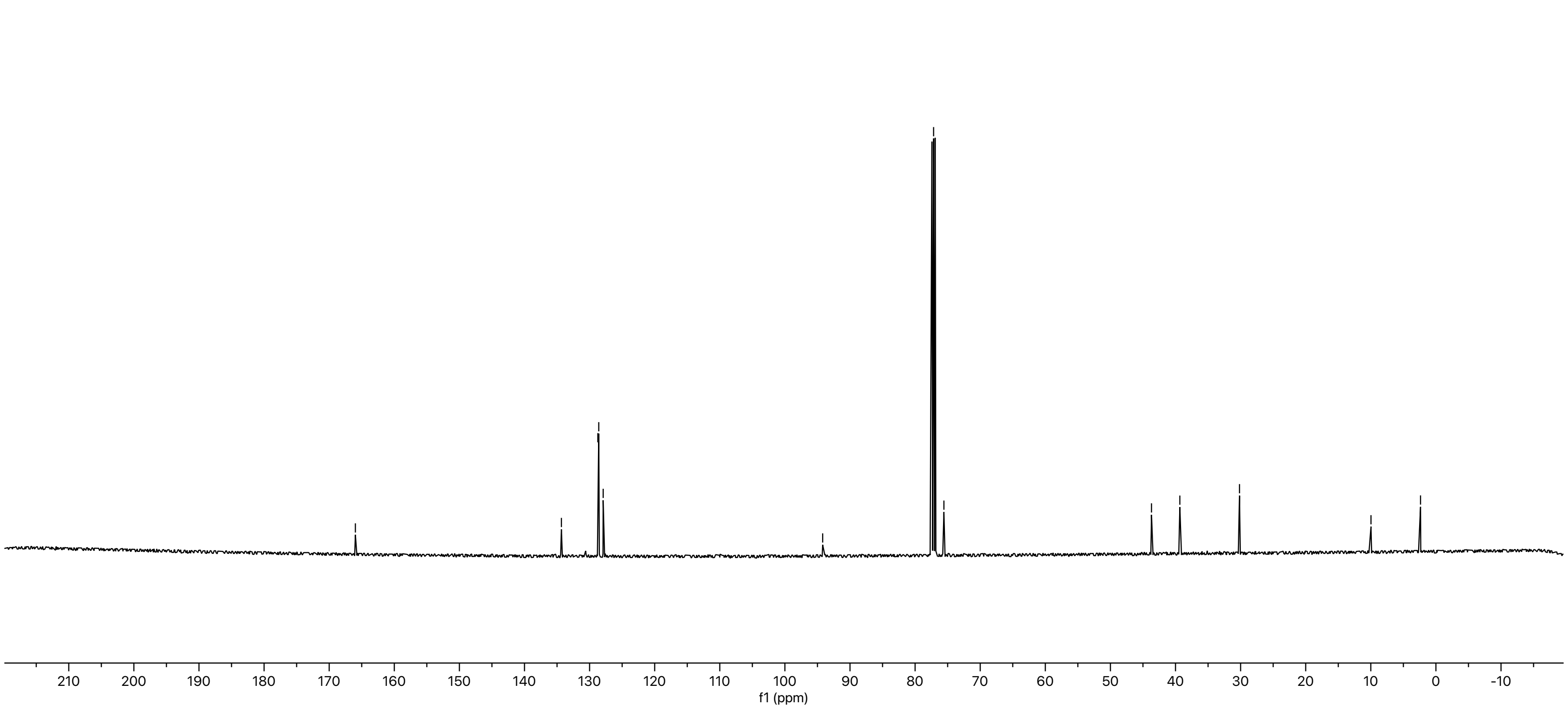

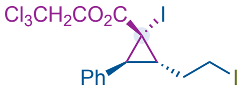

^{13}C NMR of **3bd** (126 MHz, $CDCl_3$)

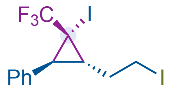

¹H NMR of **3be** (500 MHz, $CDCl_3$)

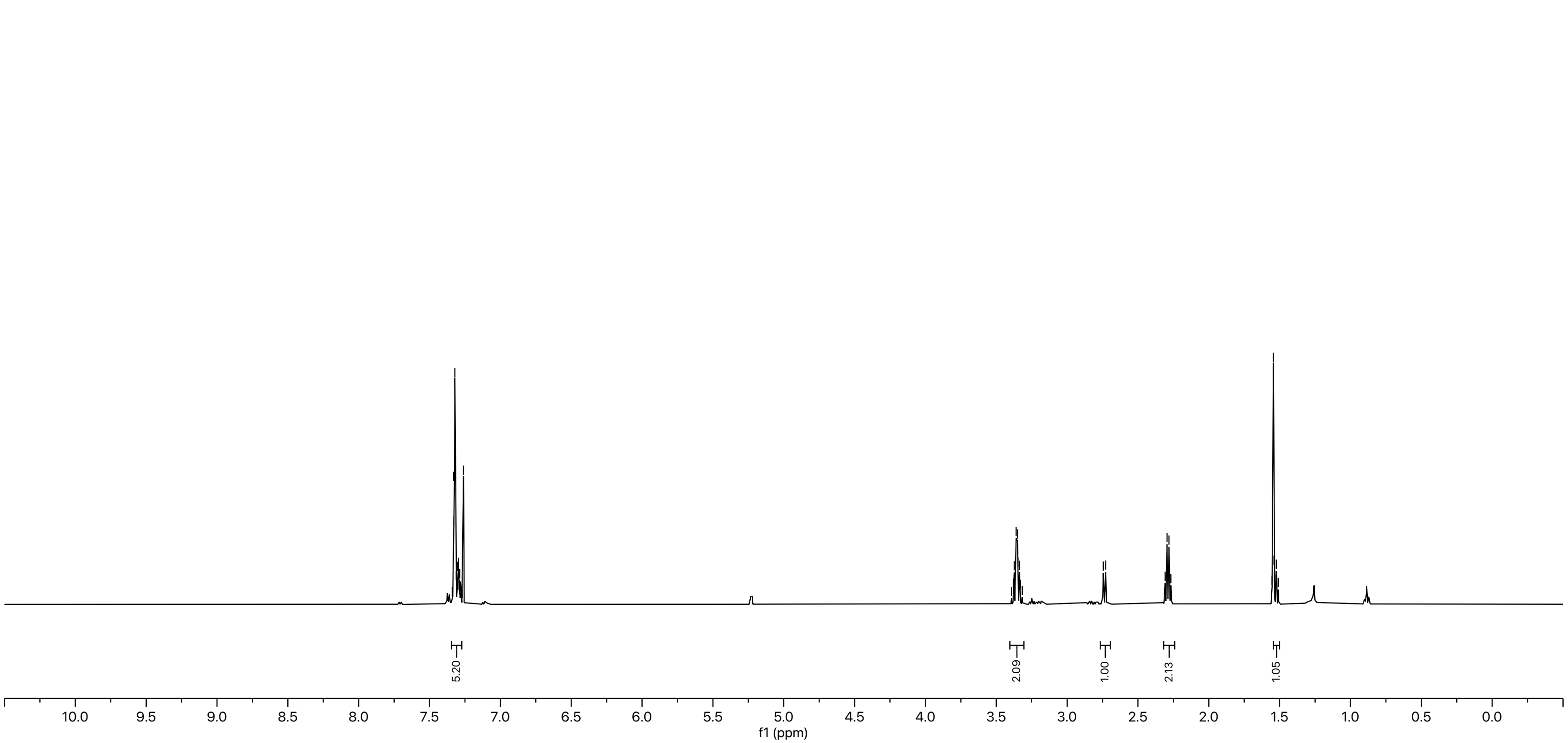

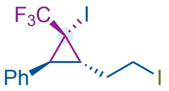

^{13}C NMR of **3be** (126 MHz, $CDCl_3$)

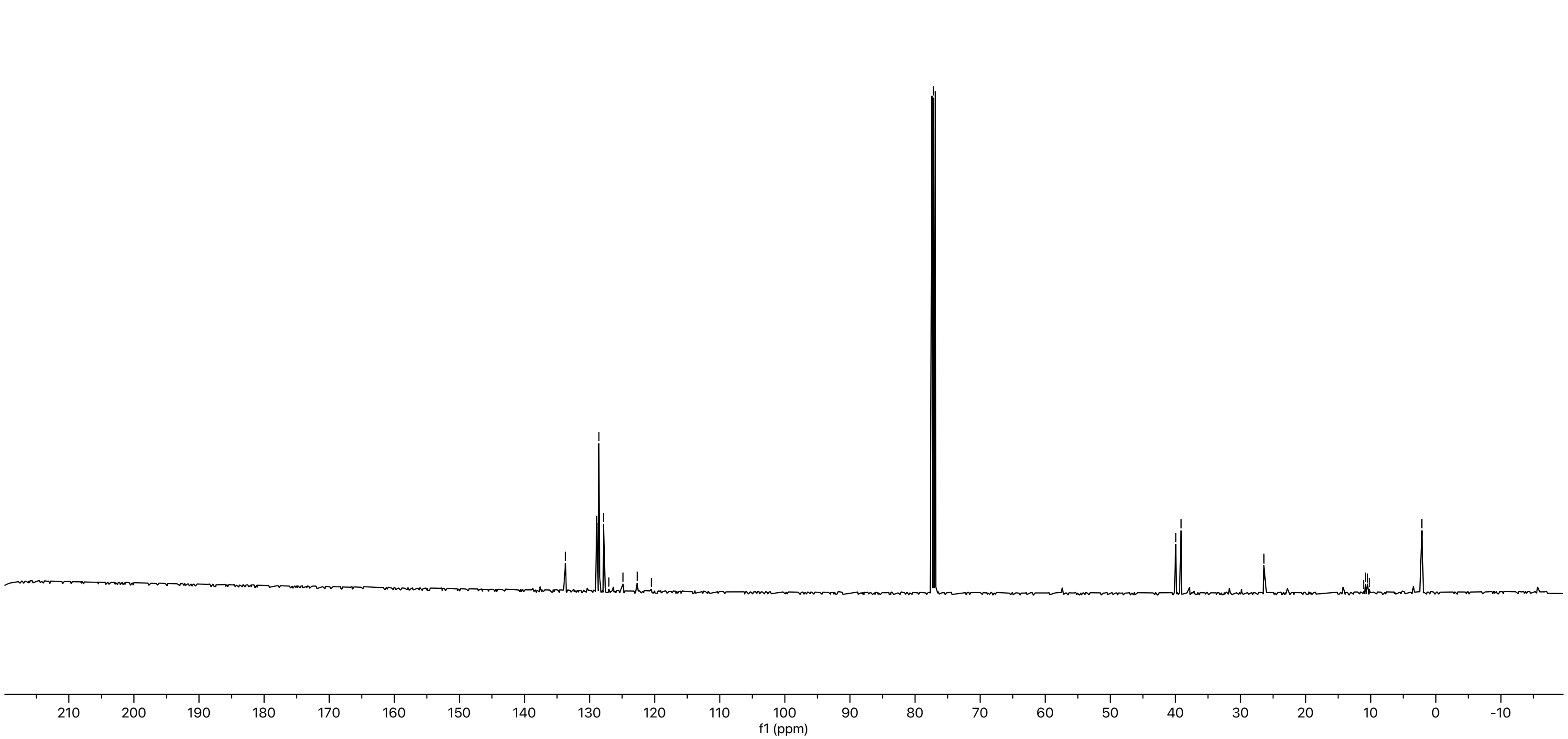

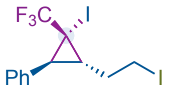

^{19}F NMR of **3be** (471 MHz, $CDCl_3$)

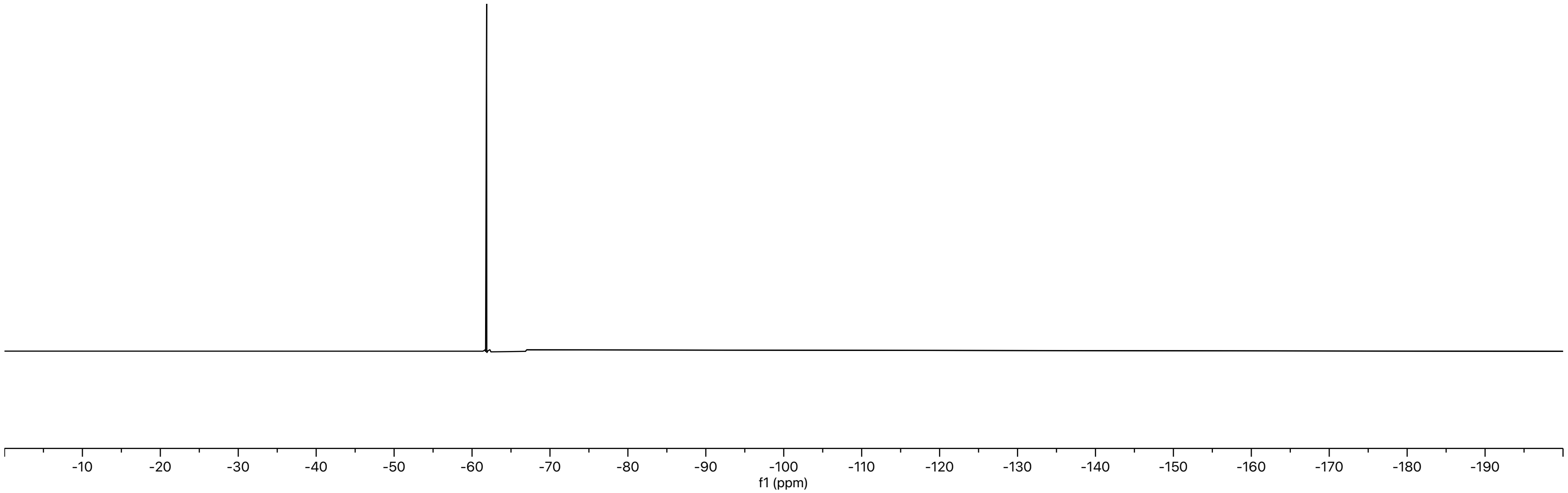

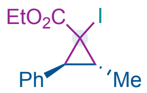

¹H NMR of **4b** (500 MHz, $CDCl_3$)

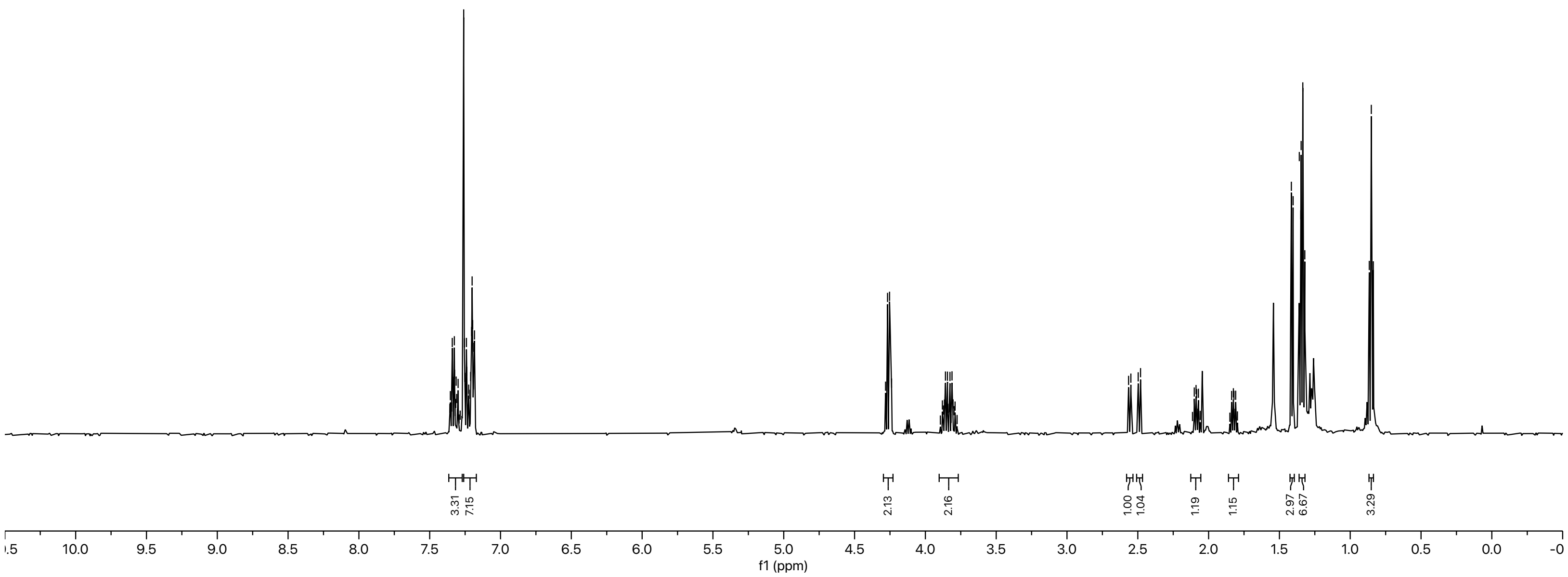

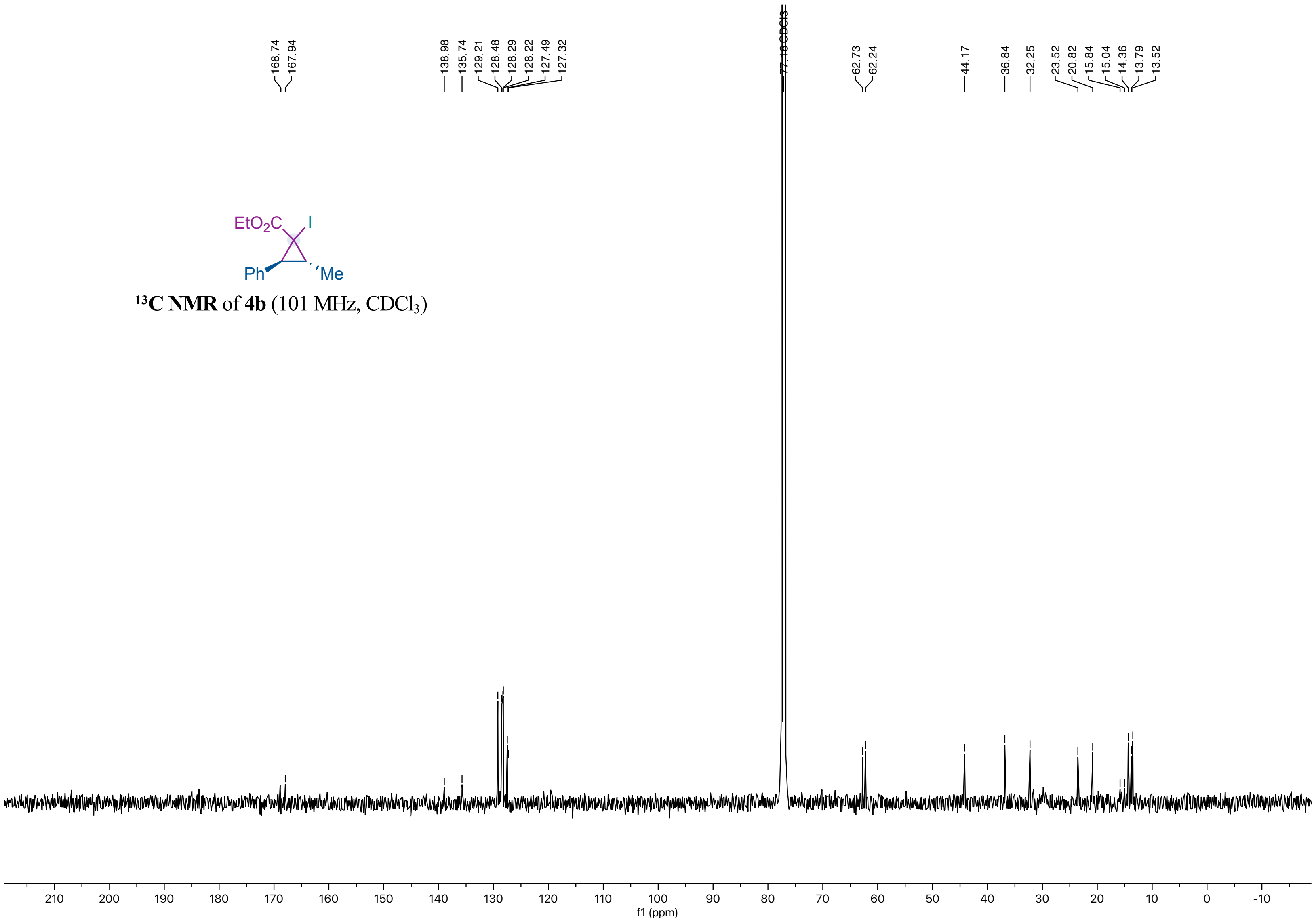

¹³C NMR of **4b** (101 MHz, $CDCl_3$)

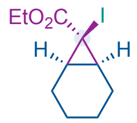

^{1}H NMR of **4c** (500 MHz, $CDCl_3$)

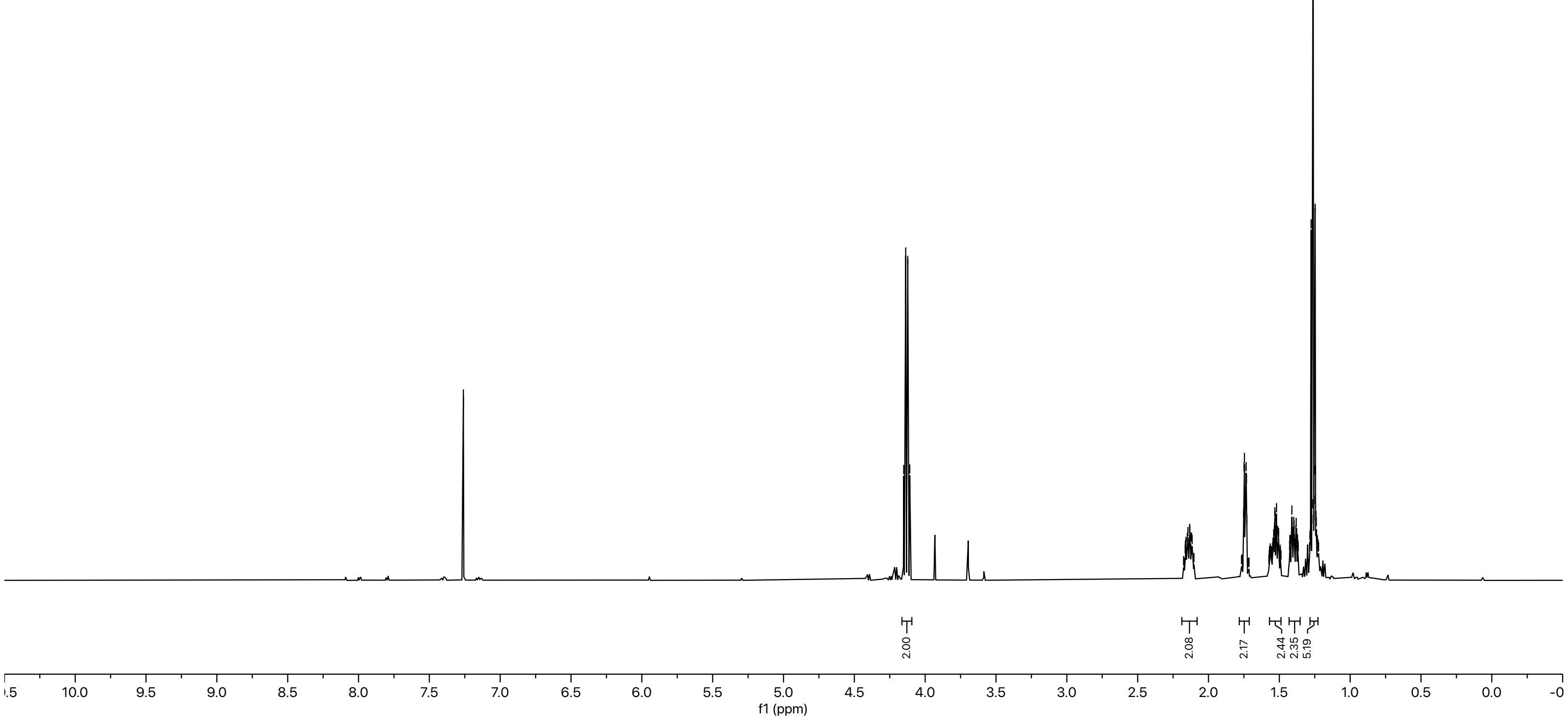

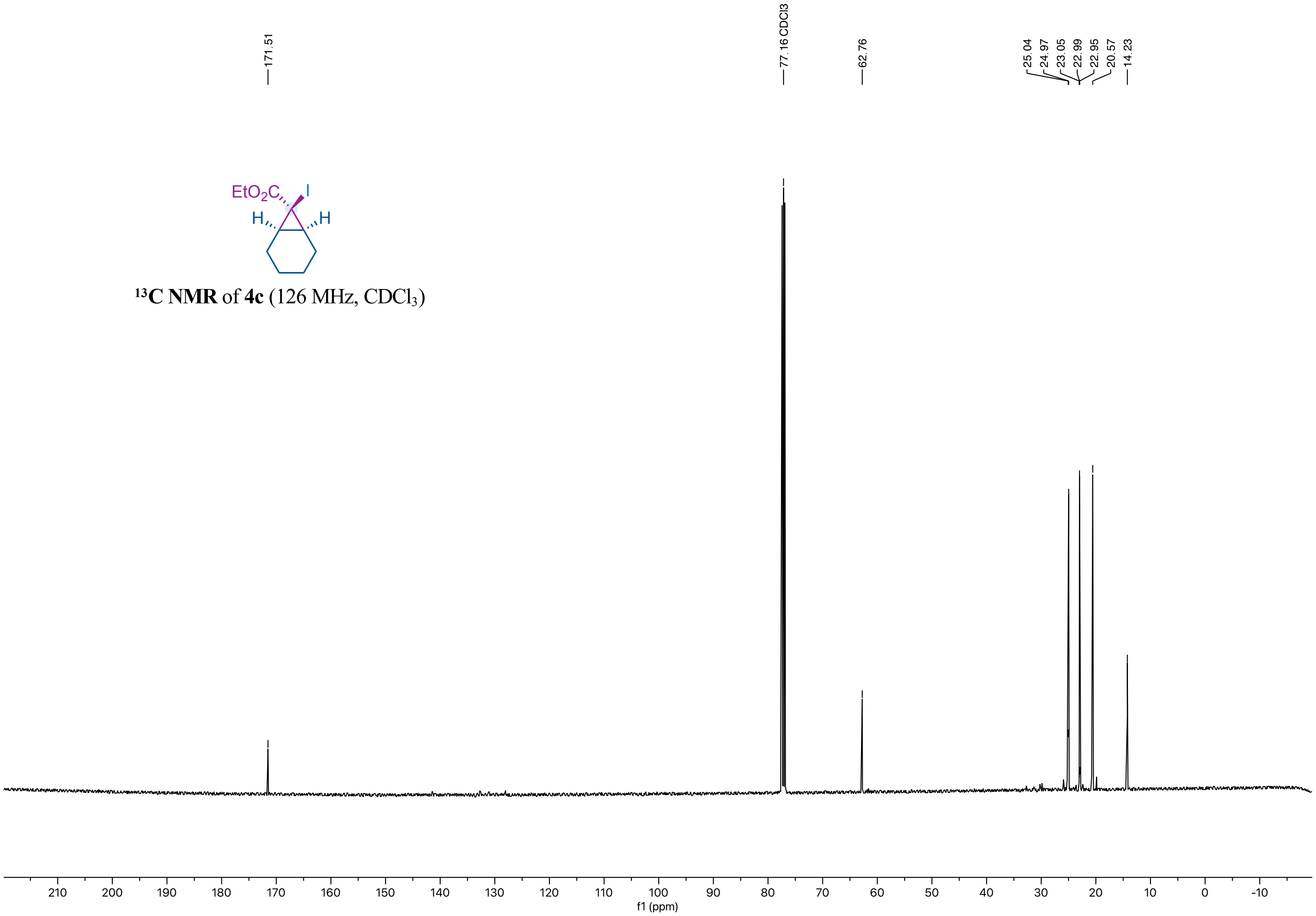

^{13}C NMR of **4c** (126 MHz, $CDCl_3$)

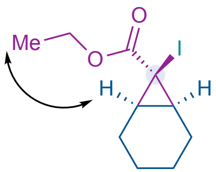
Me
O
O
I
H
H

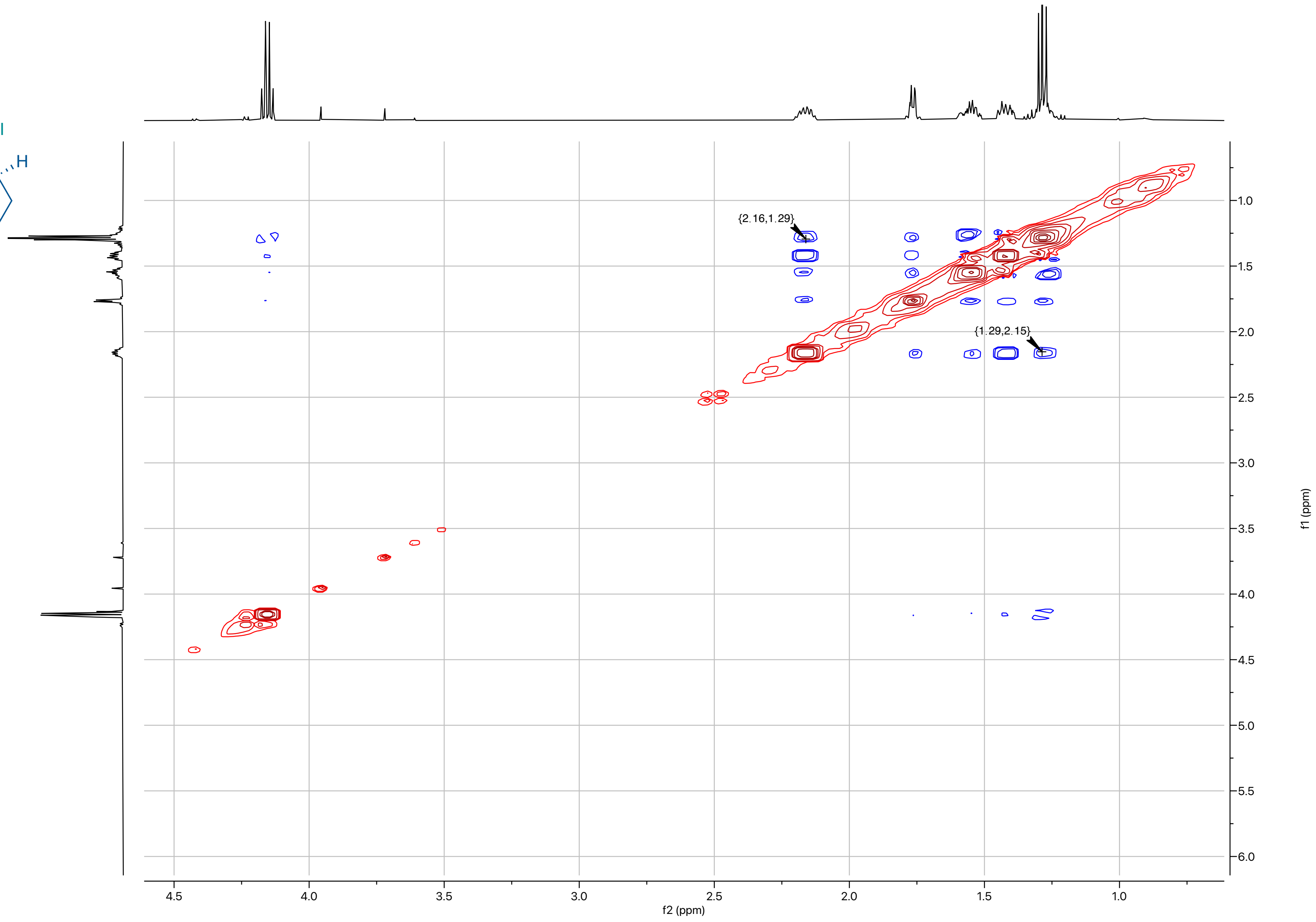
{2.16,1.29}
{1.29,2.15}
f2 (ppm)
f1 (ppm)

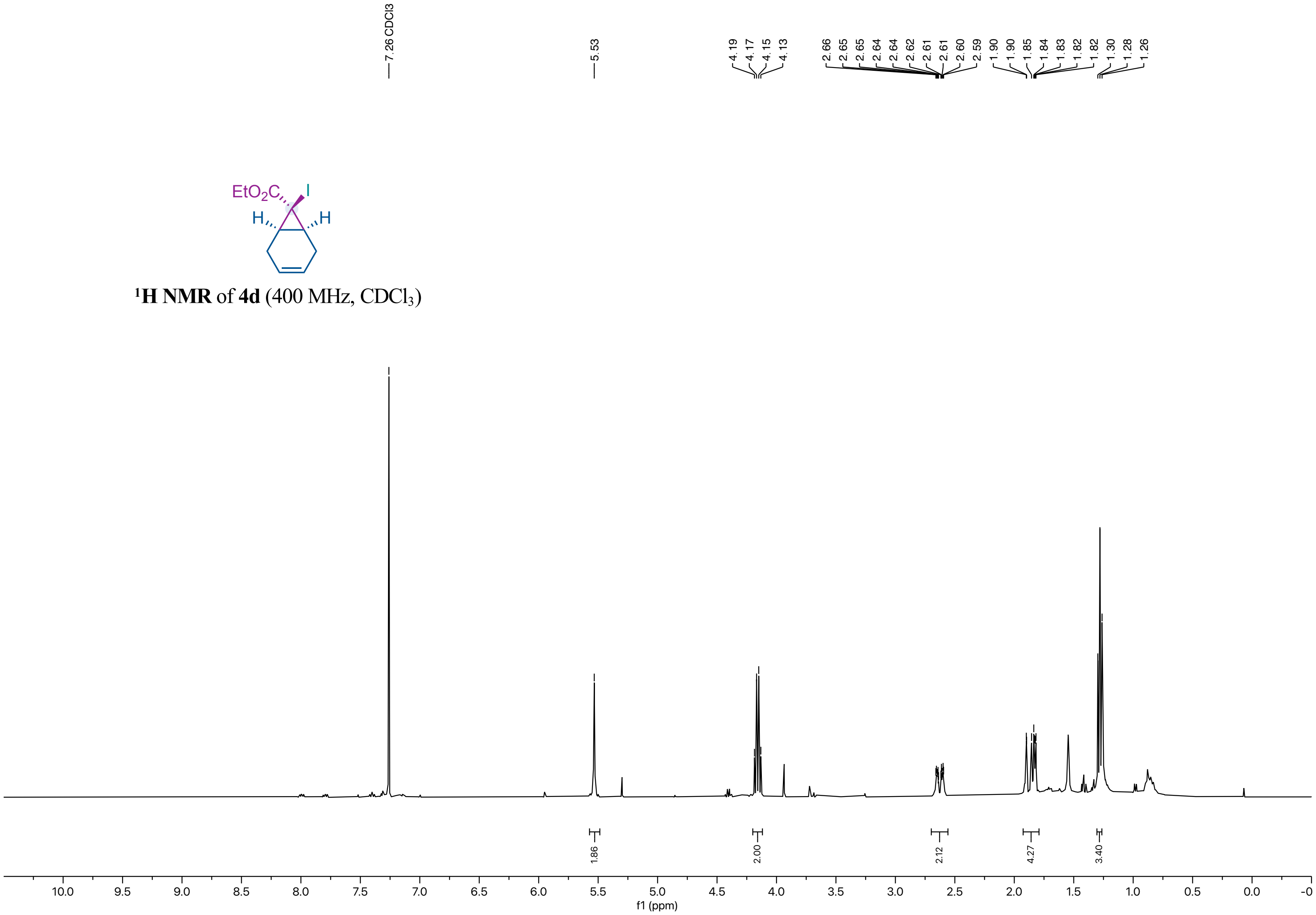

^{1}H NMR of **4d** (400 MHz, $CDCl_3$)

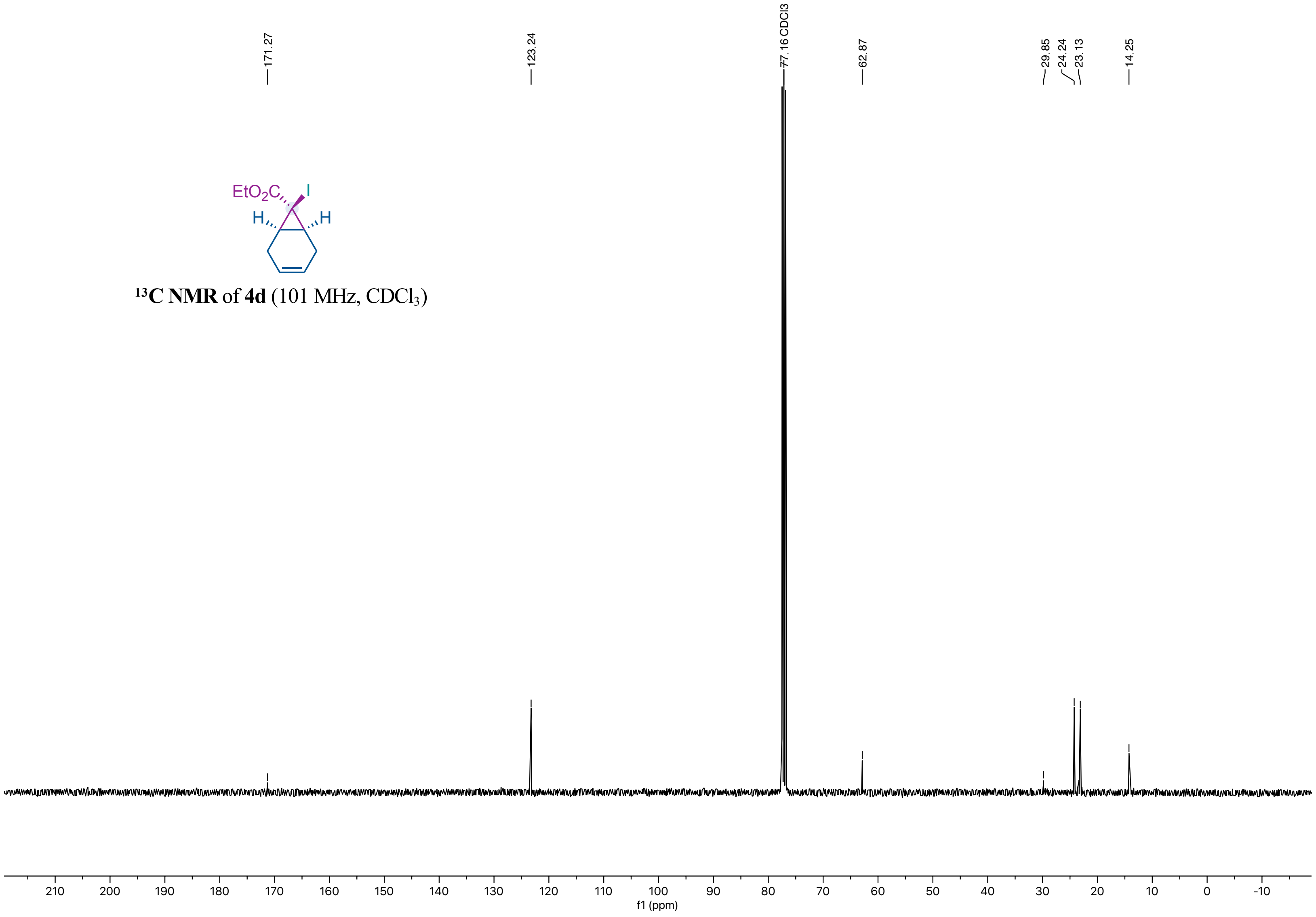

[13]**C NMR** of **4d** (101 MHz, $CDCl_3$)

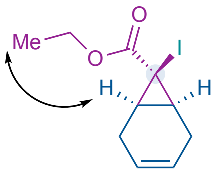
Me
O
O
I
H
H

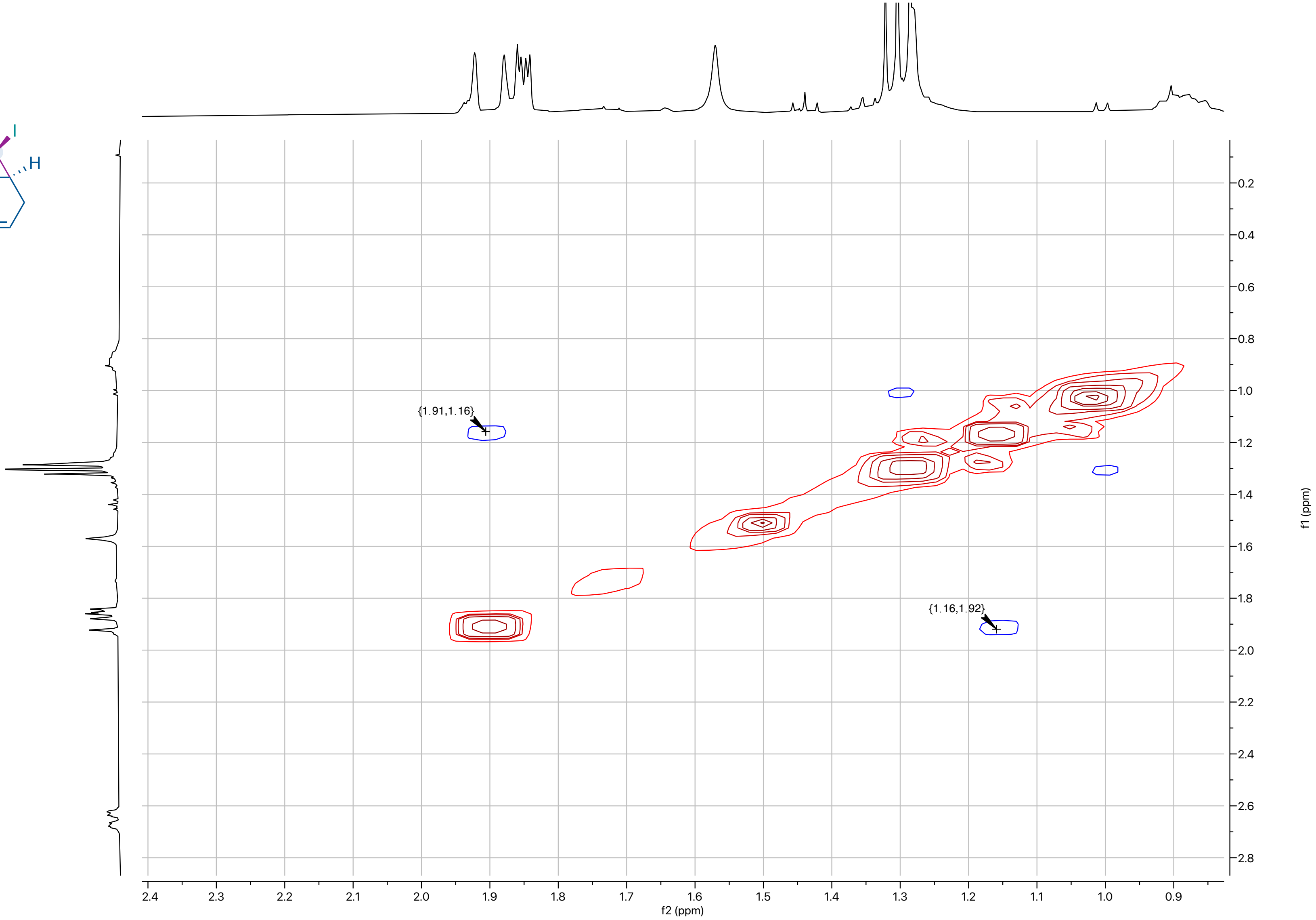
{1.91,1.16}
{1.16,1.92}
f2 (ppm)
f1 (ppm)

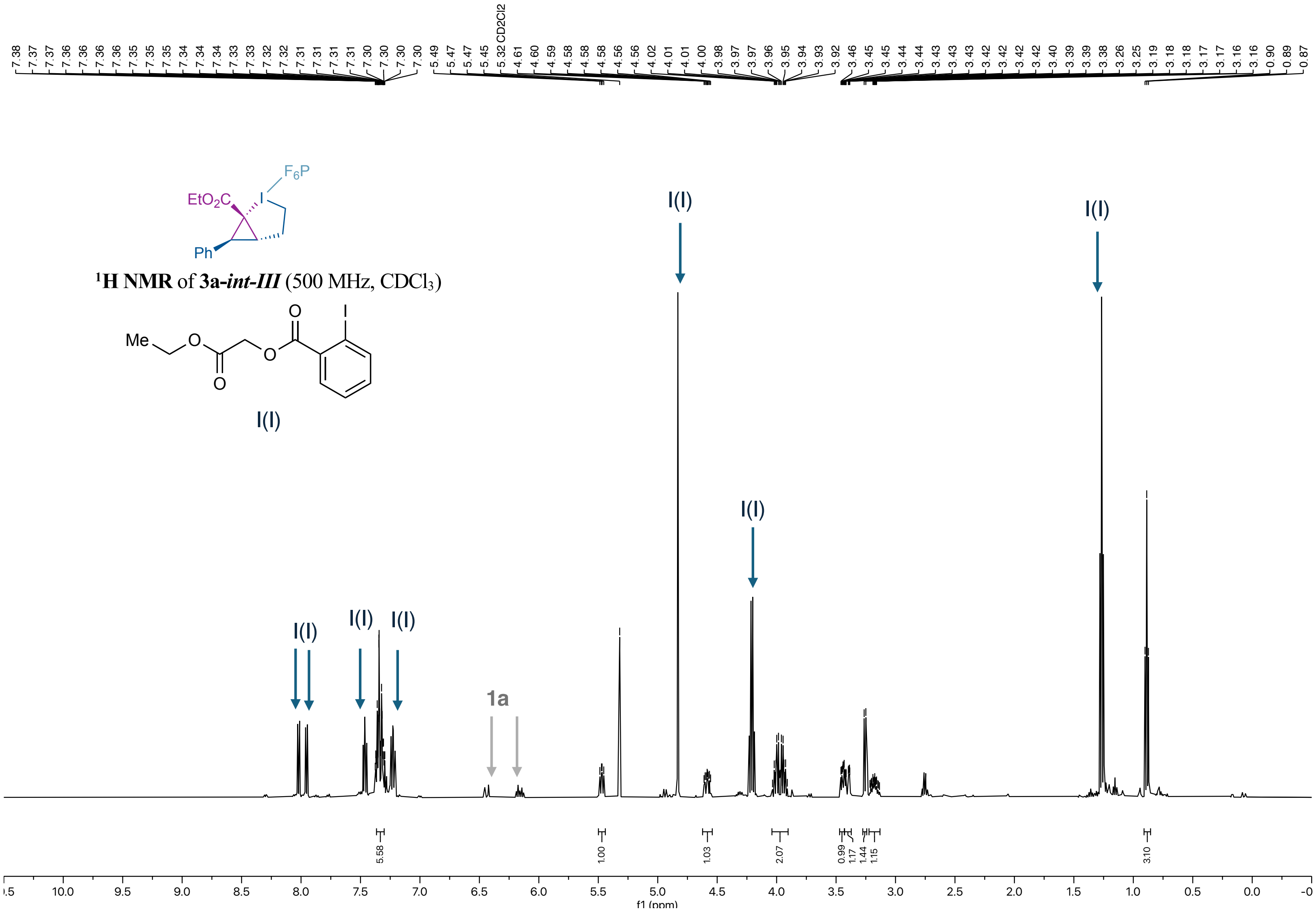

^{1}**H NMR** of **3a-*int-III*** (500 MHz, $CDCl_3$)

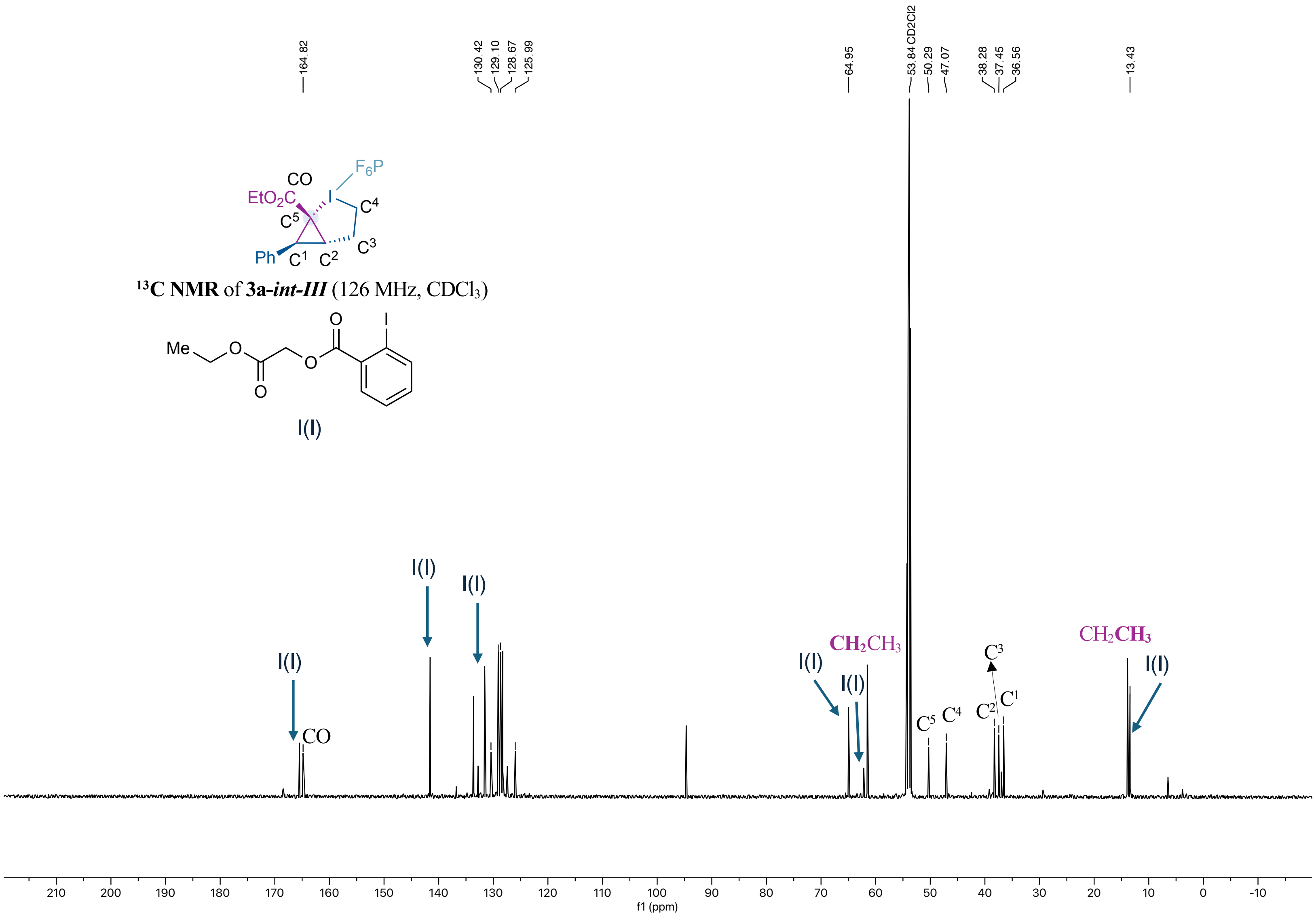

^{13}C NMR of **3a-*int-III*** (126 MHz, $CDCl_3$)

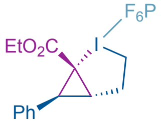

^{31}P NMR of **3a-*int-III*** (202 MHz, $CDCl_3$)

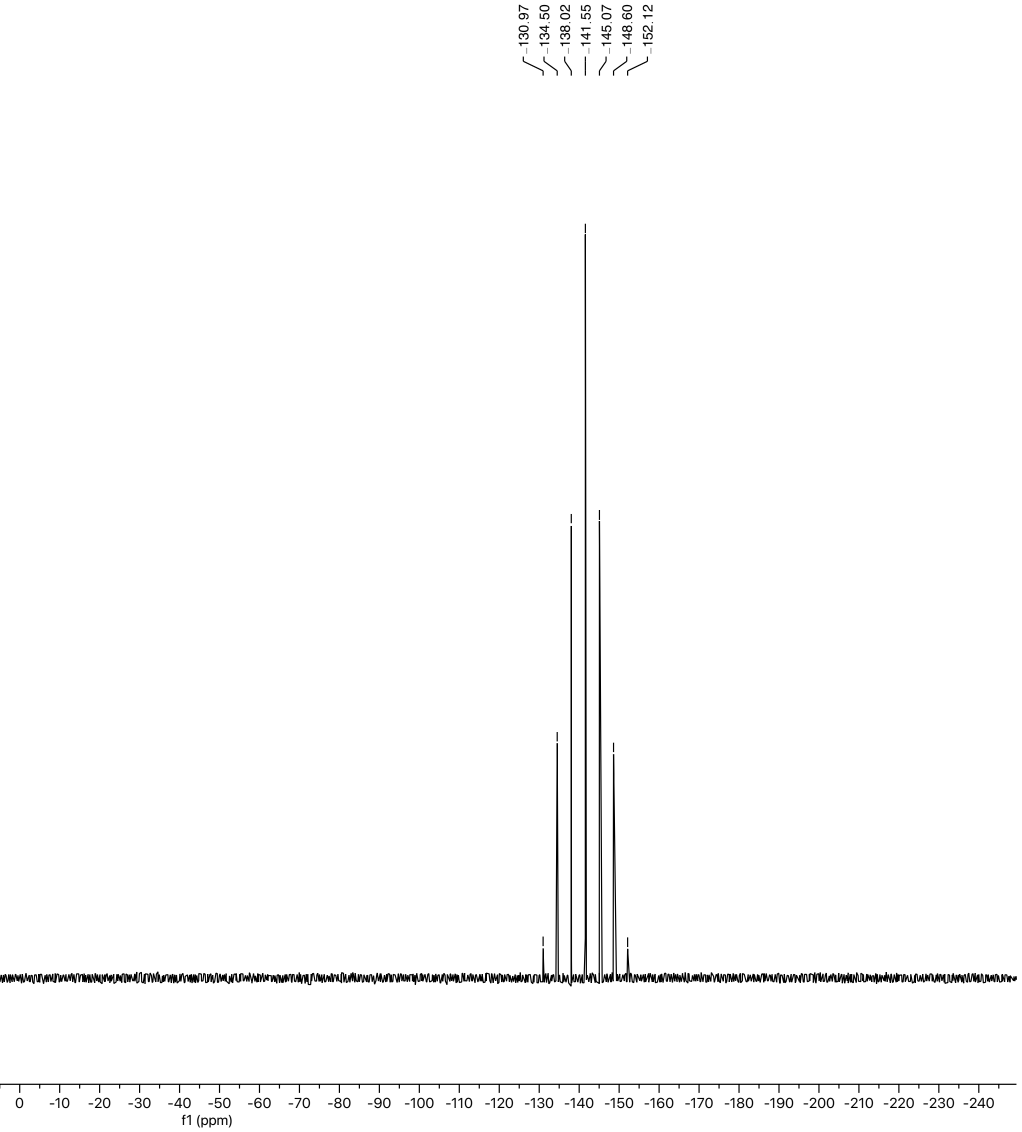

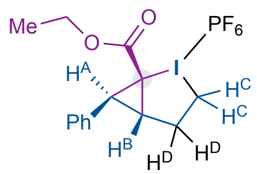

^{1}H-^{13}C HSQC of 3a-*int-III* (CD_2Cl_2)

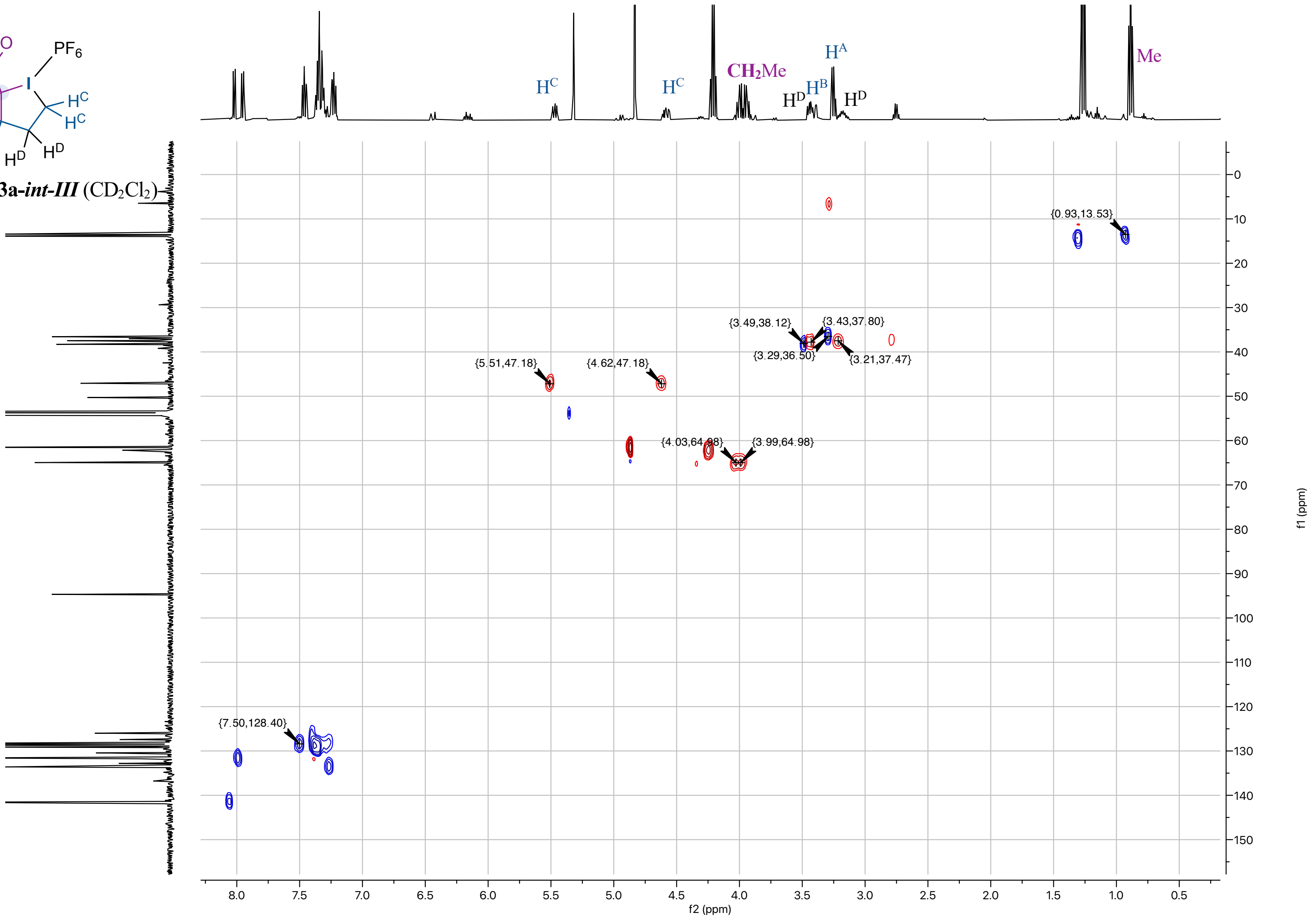

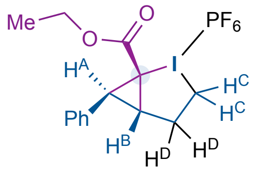

^{1}H-^{13}C HMBC of 3a-*int-III* (CD_2Cl_2)

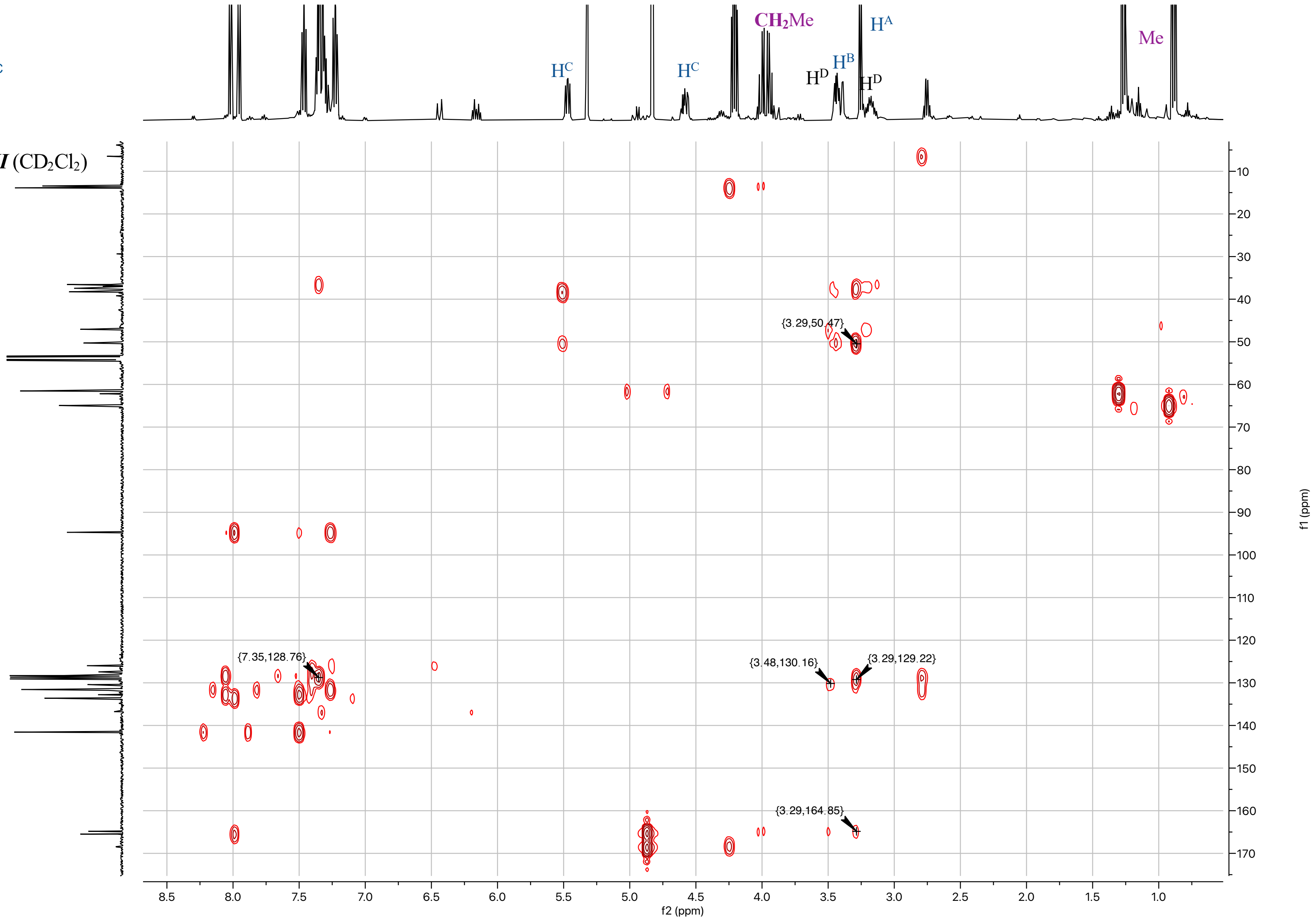

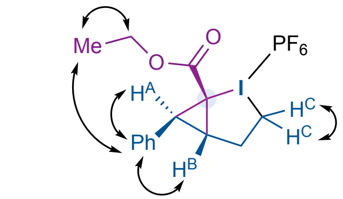

^{1}H-^{1}H 2D-NOESY of **3a-*int-III*** (CD_2Cl_2)

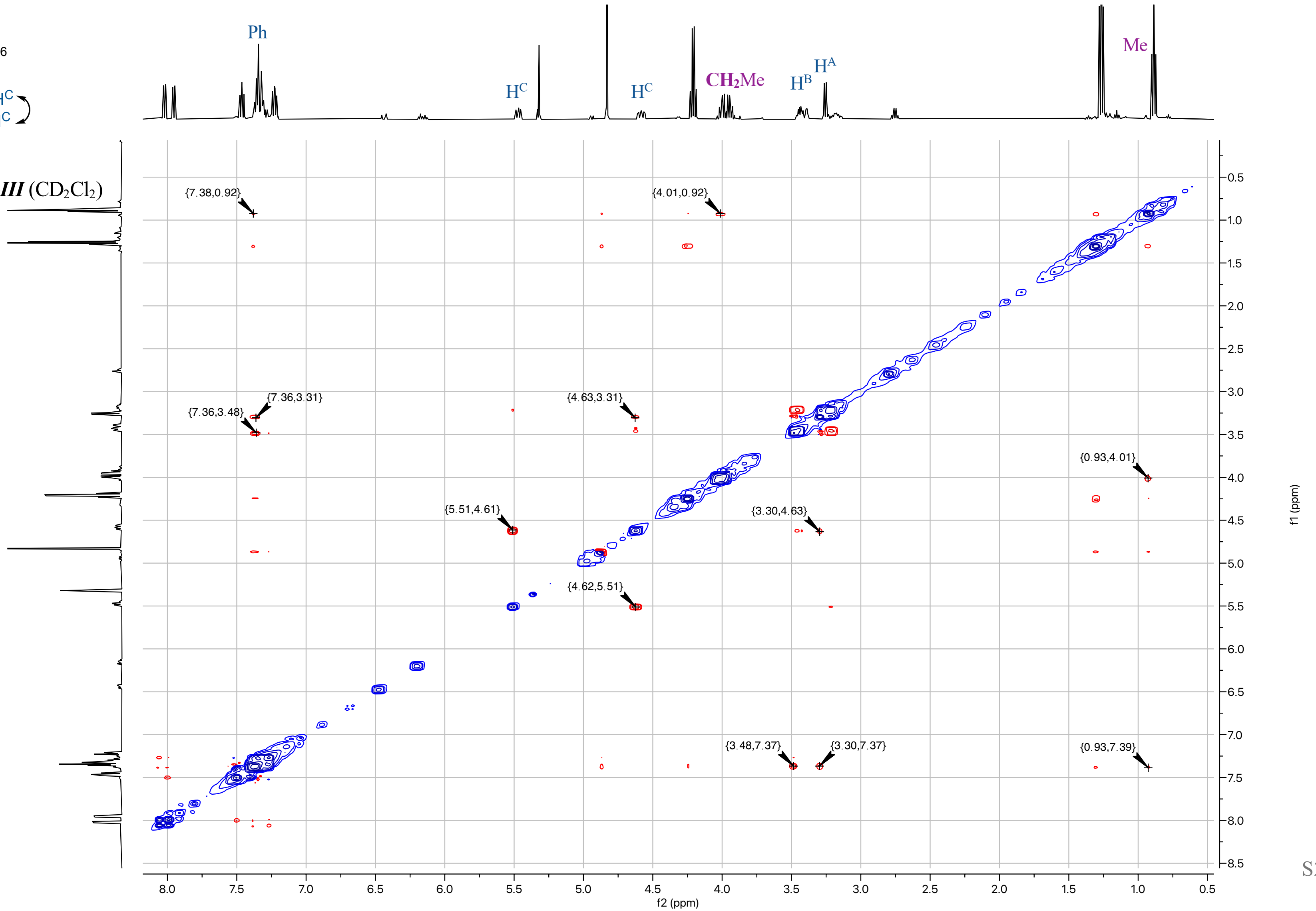

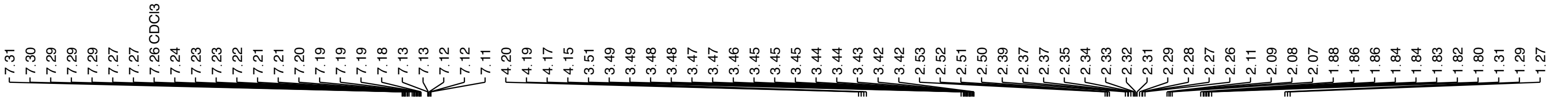

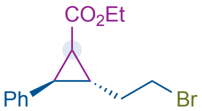

^{1}H NMR of **5a*** dr1 (400 MHz, $CDCl_3$)

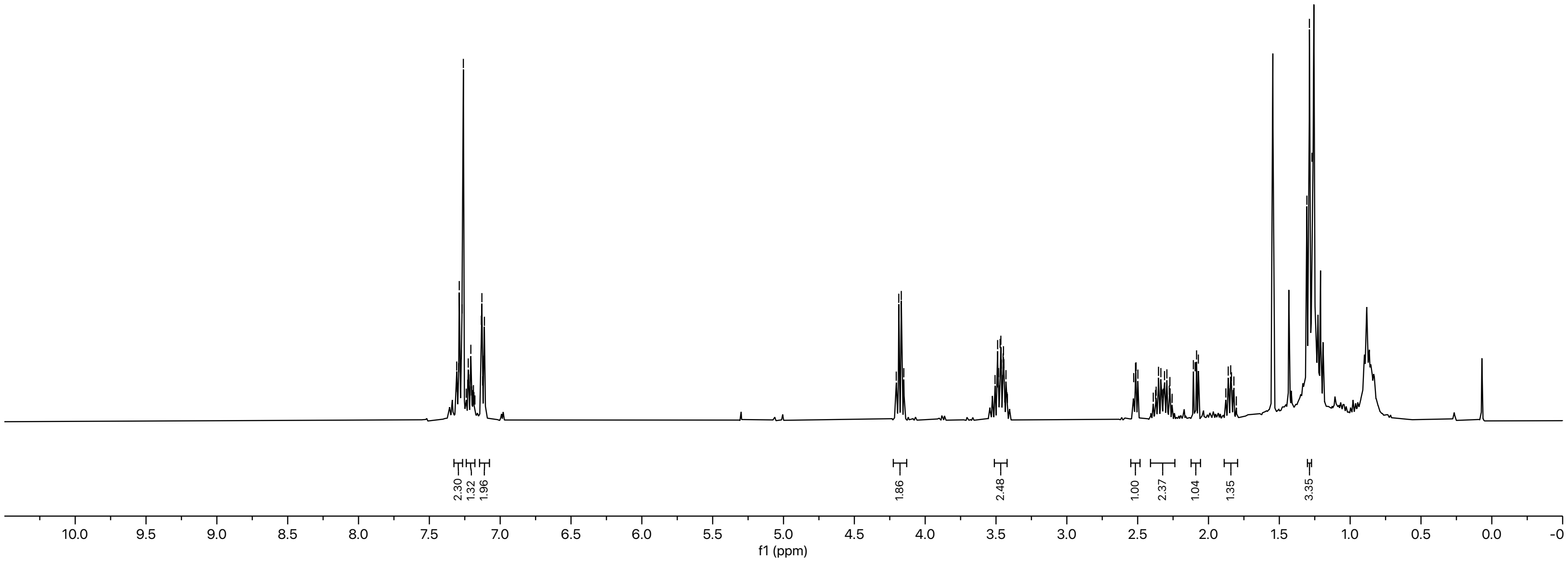

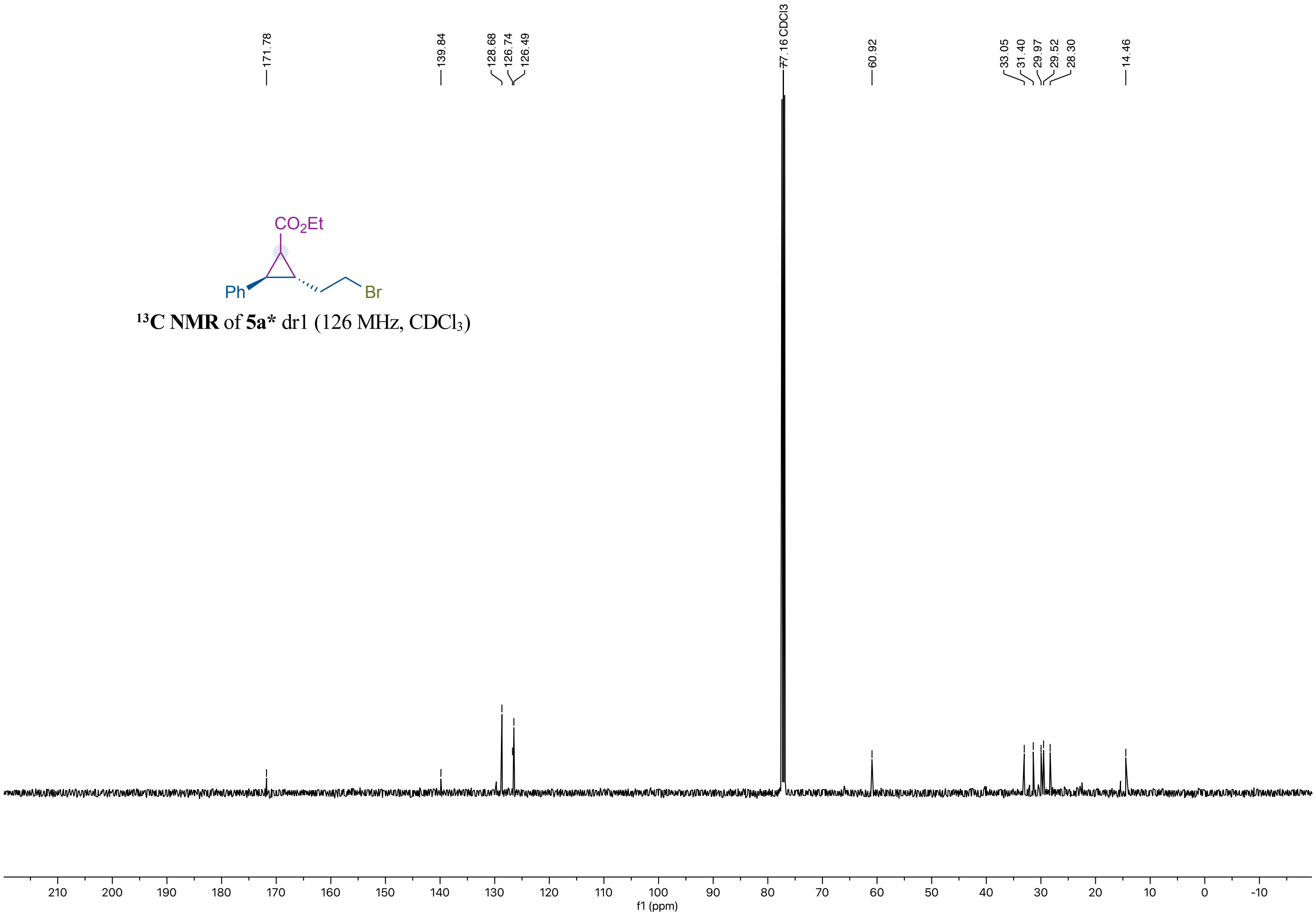

^{13}C NMR of **5a*** dr1 (126 MHz, $CDCl_3$)

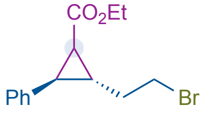

[1]**H NMR** of **5a*** dr2 (500 MHz, $CDCl_3$)

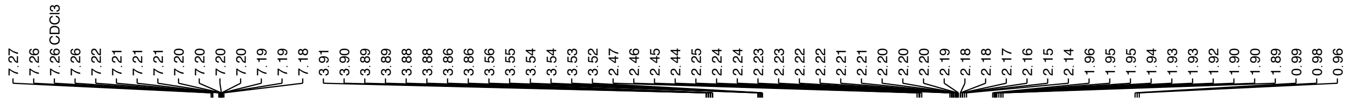

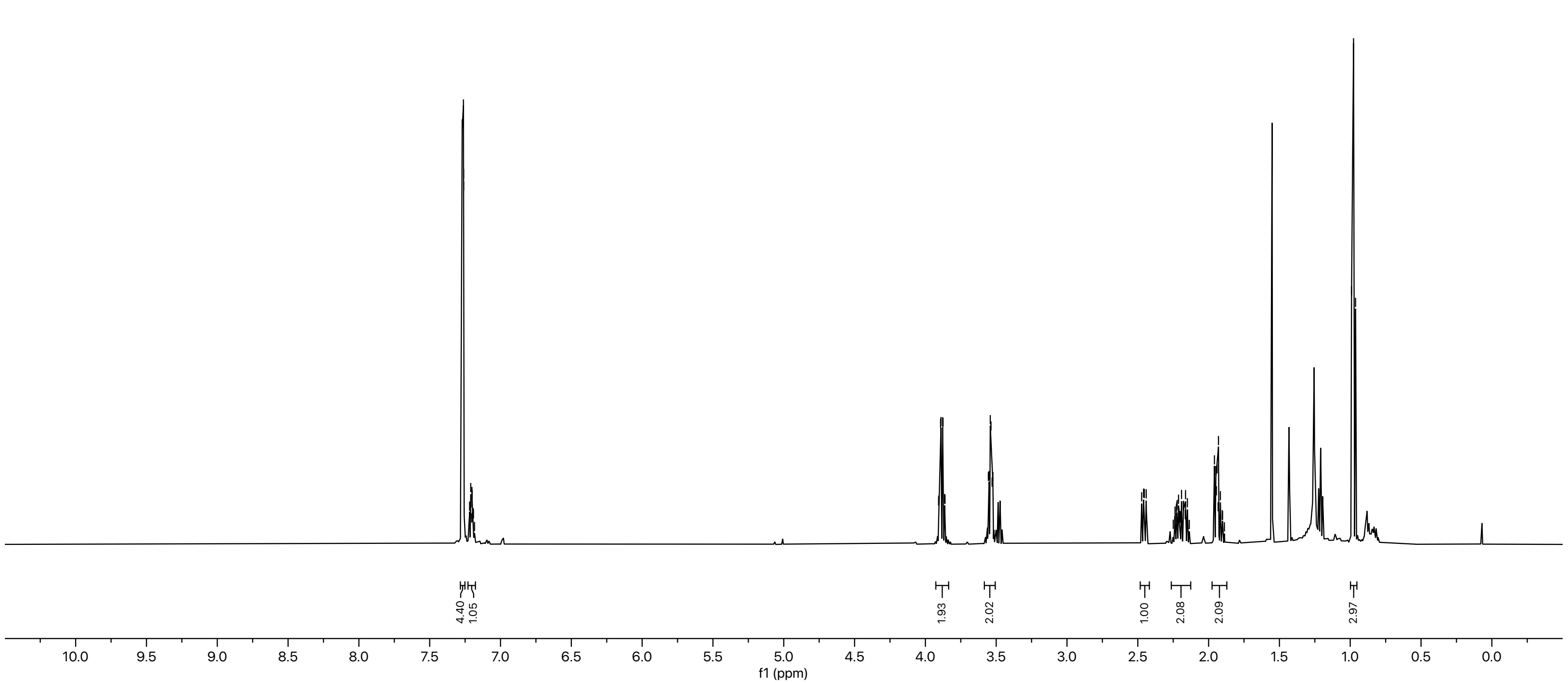

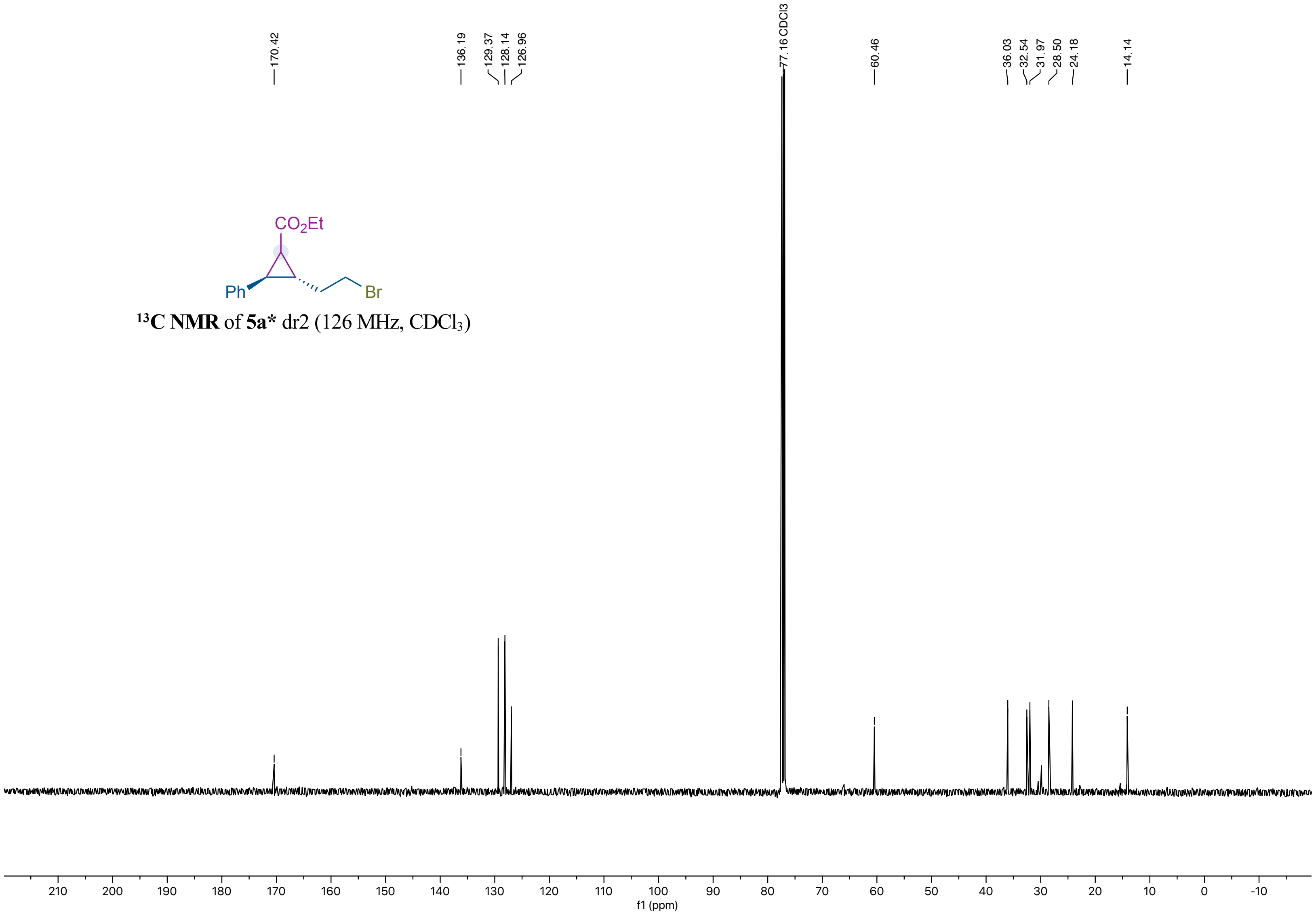

[13]**C NMR** of **5a*** dr2 (126 MHz, $CDCl_3$)

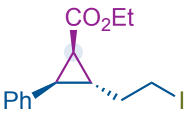

^{1}H NMR of **5b*** (400 MHz, $CDCl_3$)

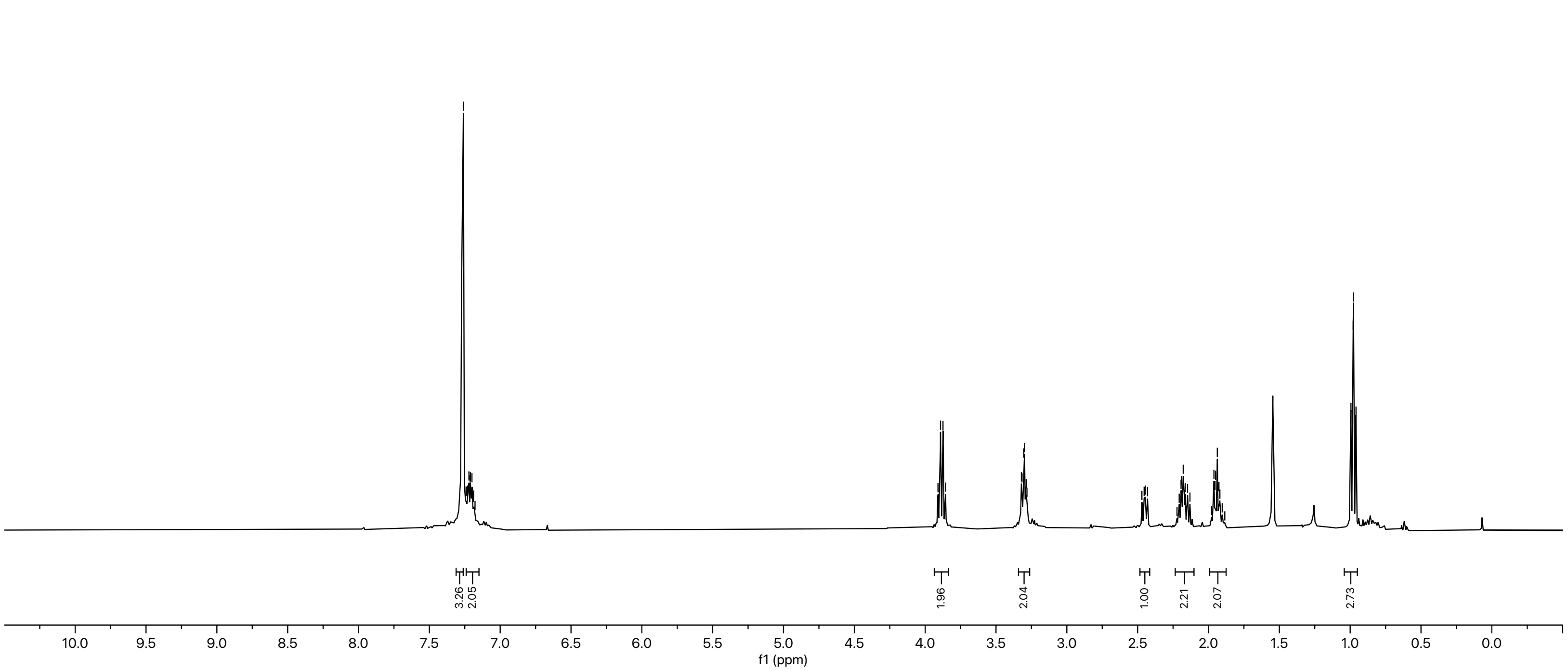

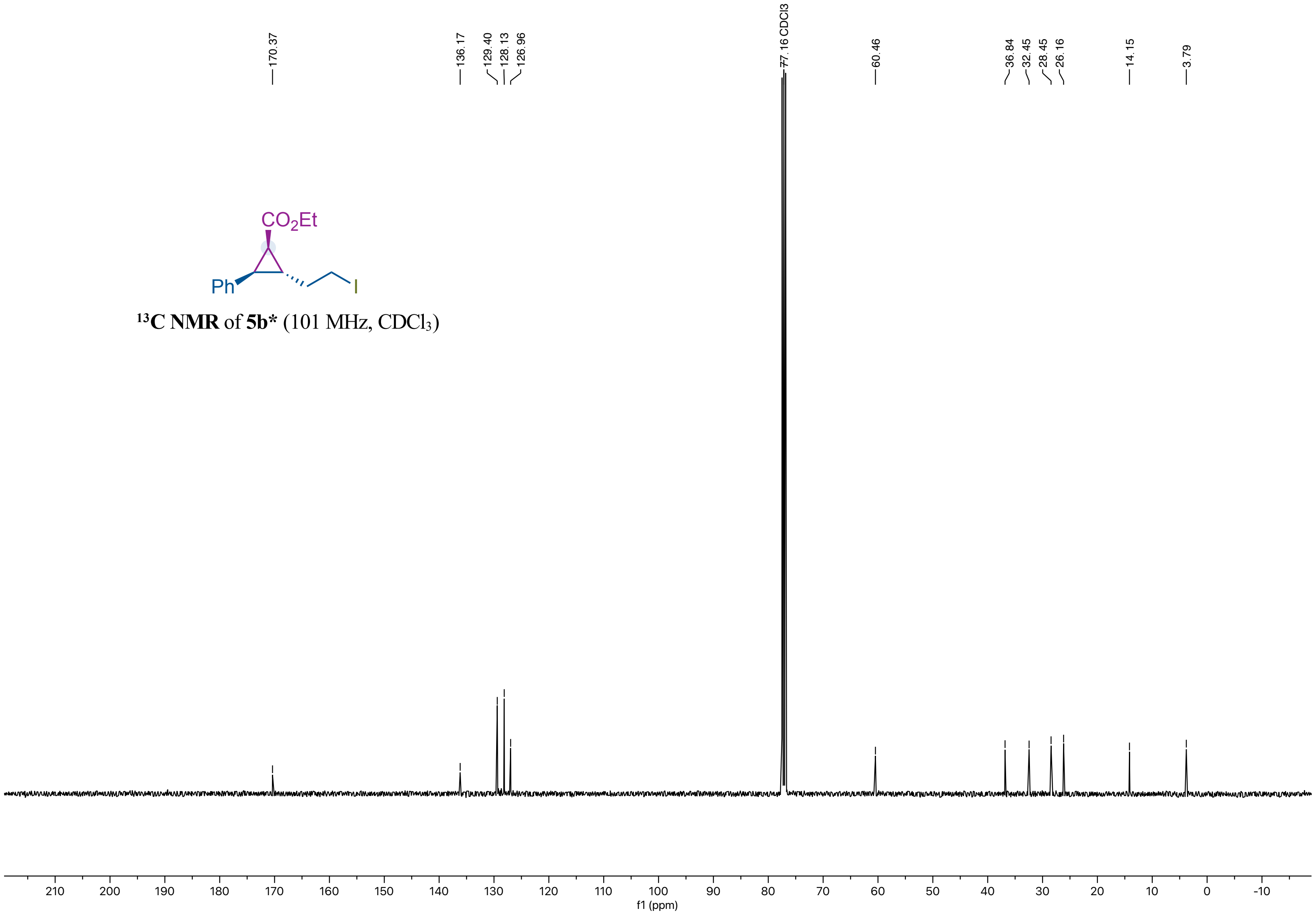

^{13}**C NMR** of **5b*** (101 MHz, $CDCl_3$)

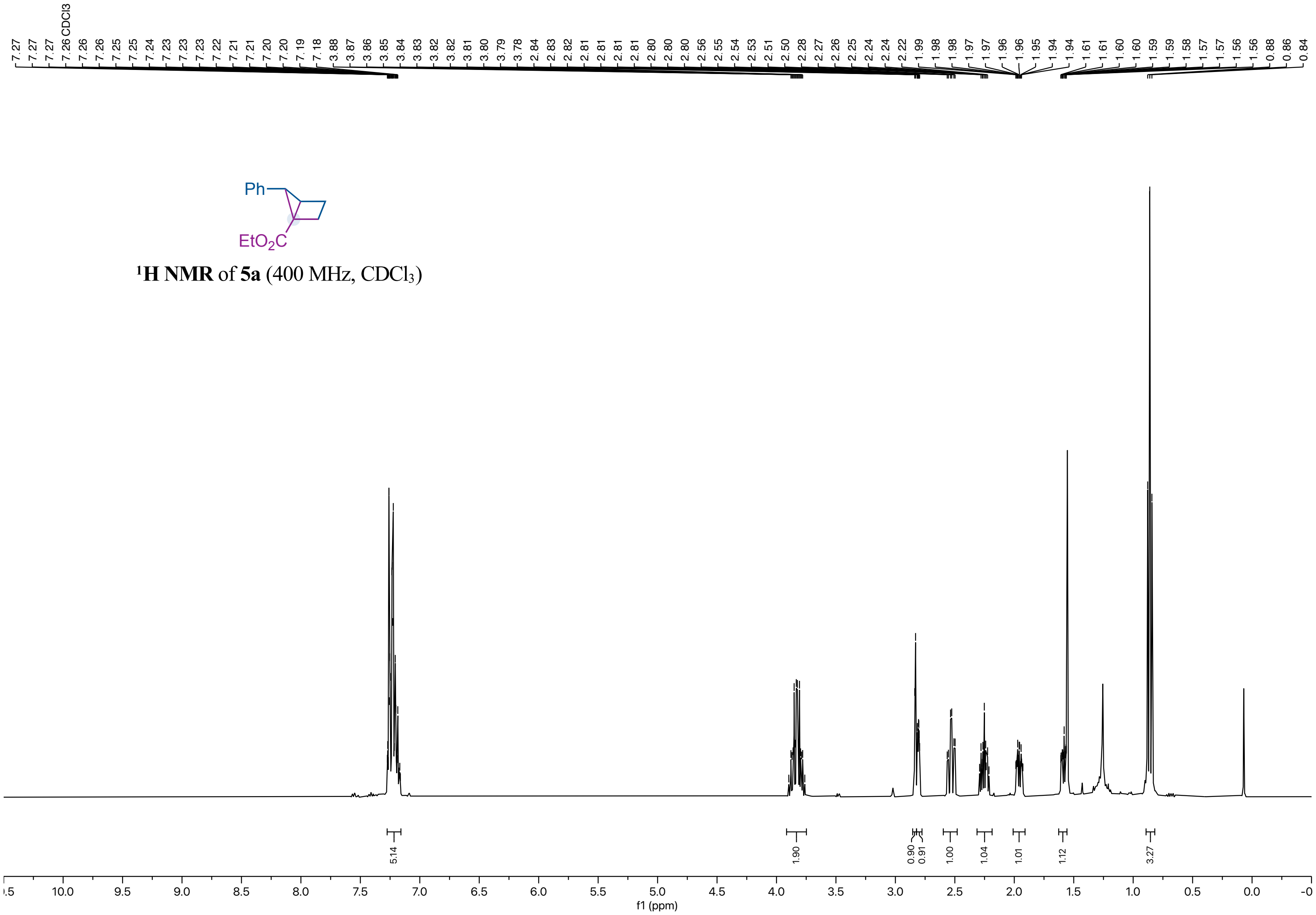

^{1}H NMR of **5a** (400 MHz, $CDCl_3$)

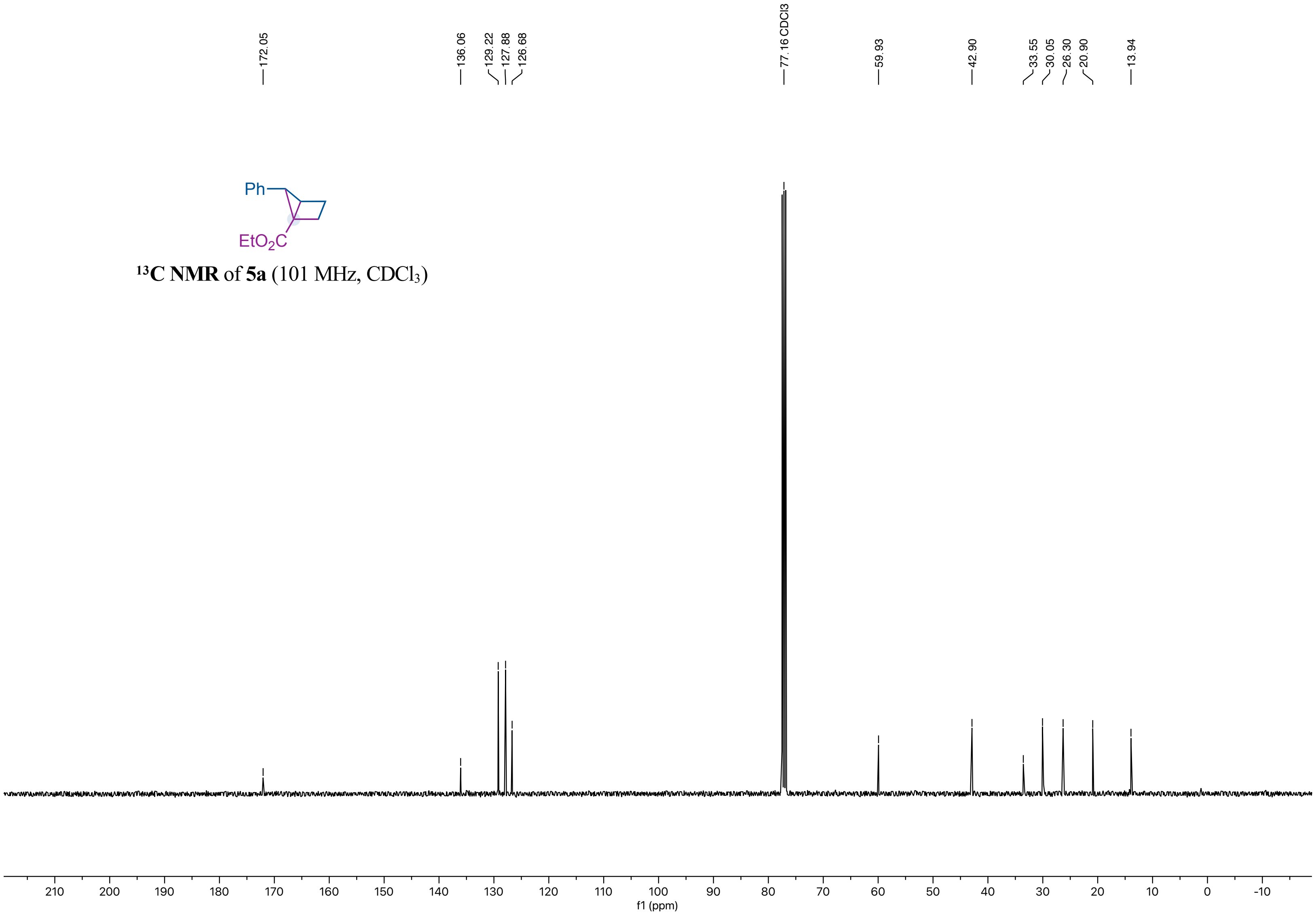

^{13}C NMR of **5a** (101 MHz, $CDCl_3$)

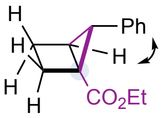

H
H
H
H
Ph
H
CO2Et

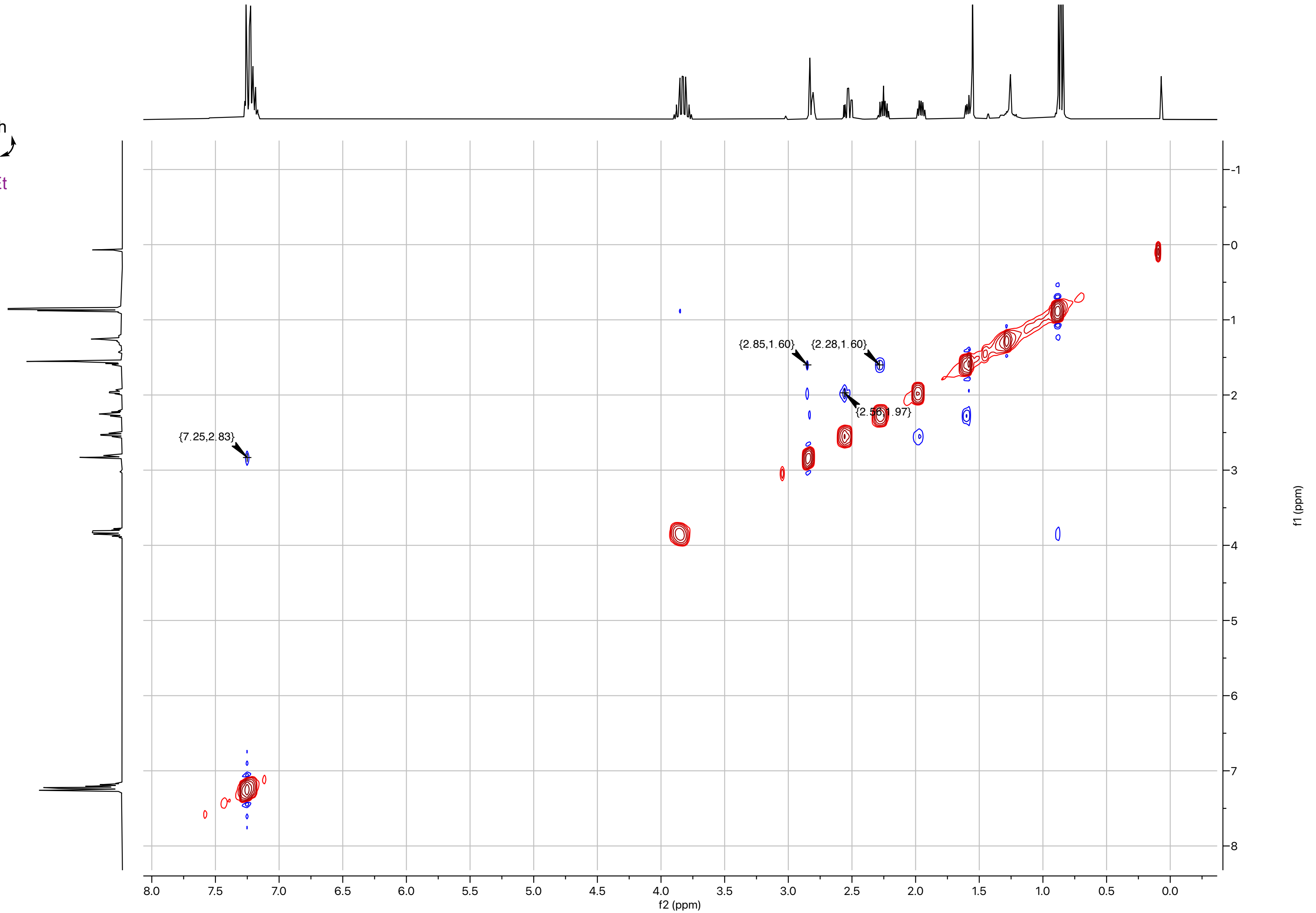

{2.85,1.60}
{2.28,1.60}
{2.56,1.97}
{7.25,2.83}
f2 (ppm)
f1 (ppm)

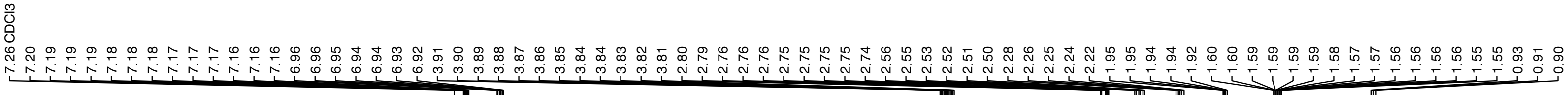

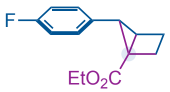

¹H NMR of **5b** (400 MHz, $CDCl_3$)

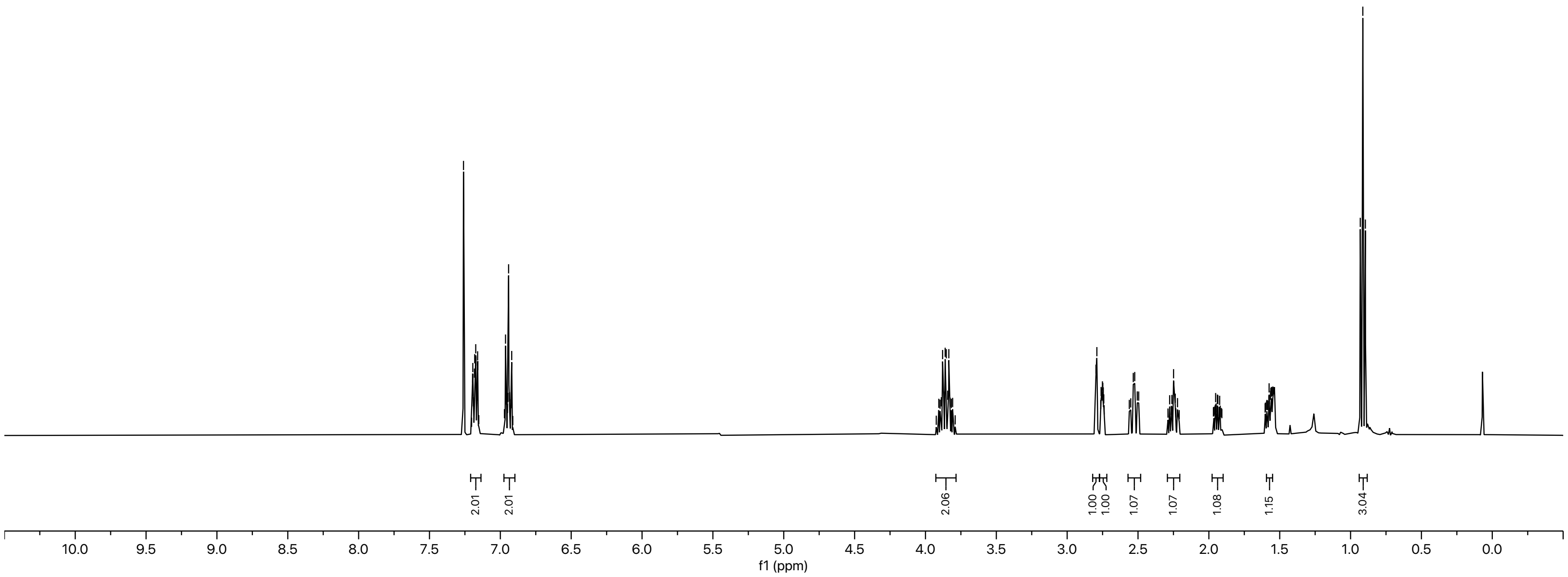

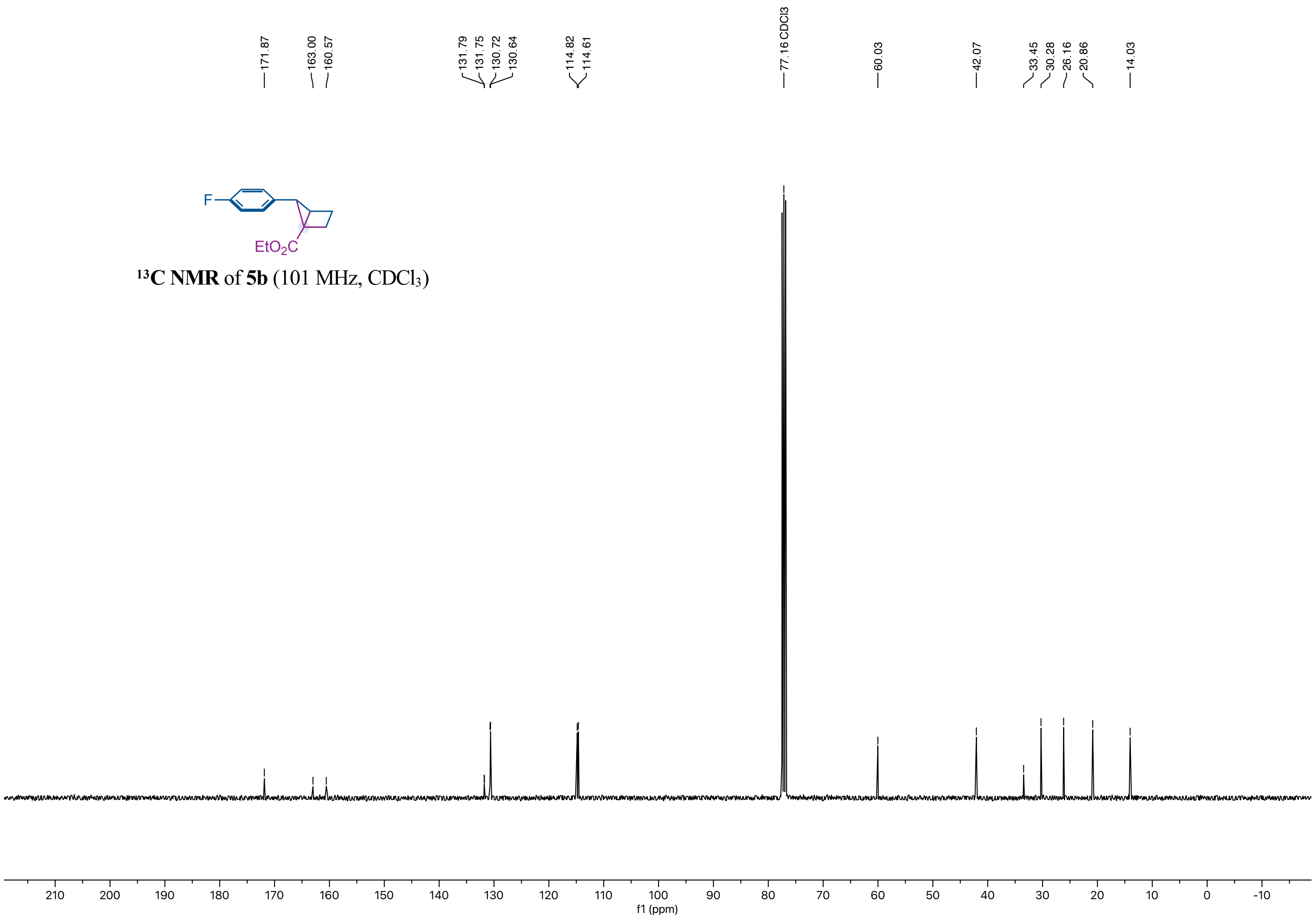

^{13}C NMR of **5b** (101 MHz, $CDCl_3$)

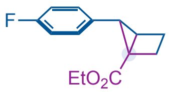

^{19}F NMR of **5b** (471 MHz, $CDCl_3$)

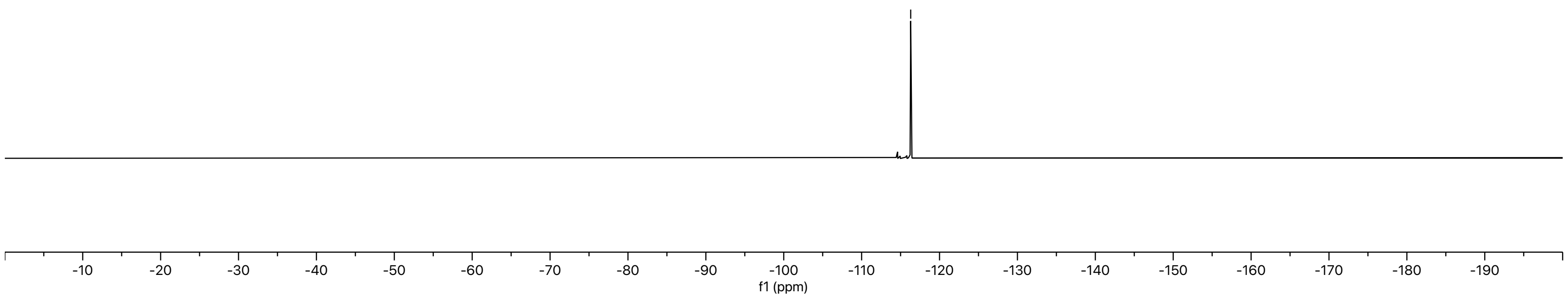

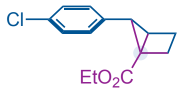

^{1}**H NMR** of **5c** (400 MHz, $CDCl_3$)

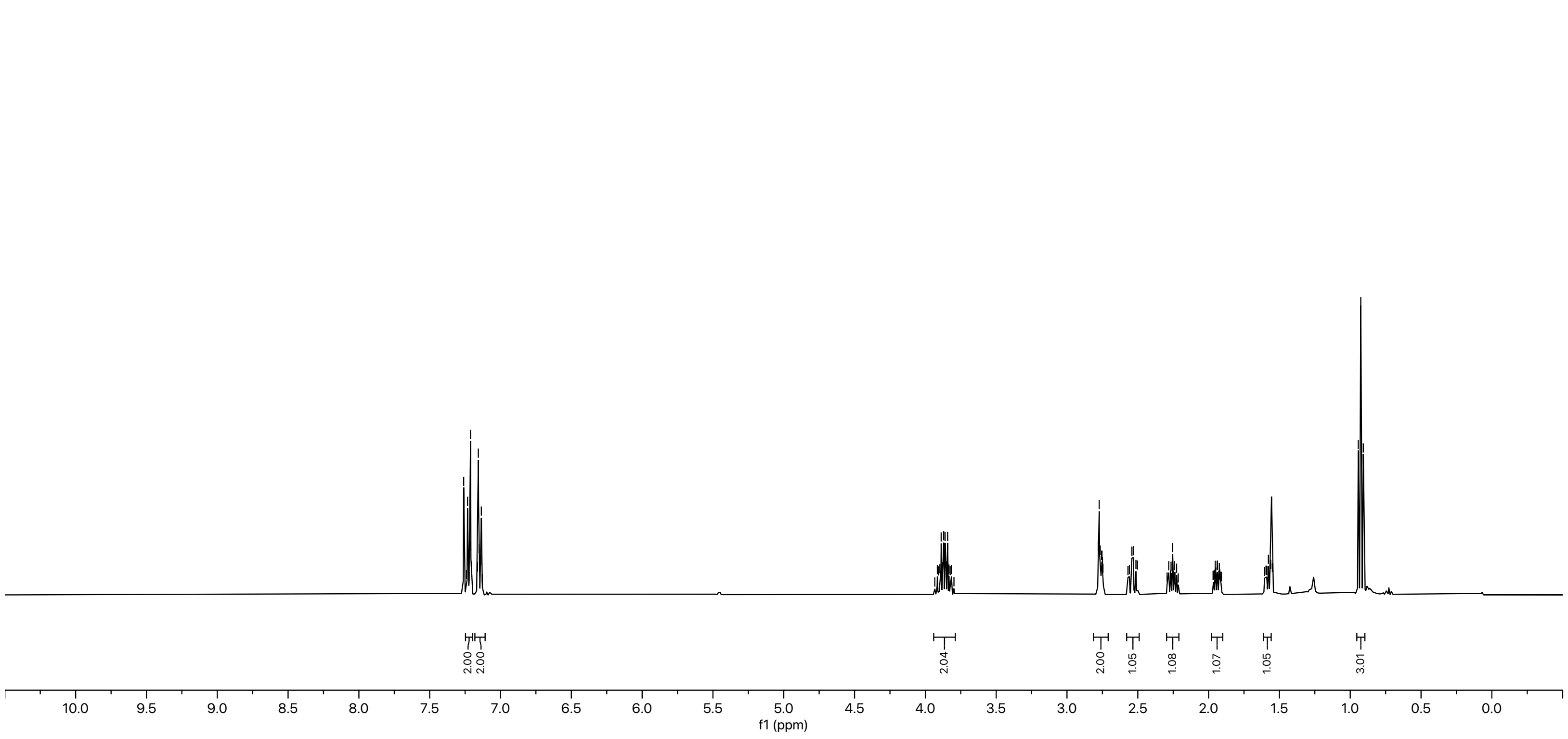

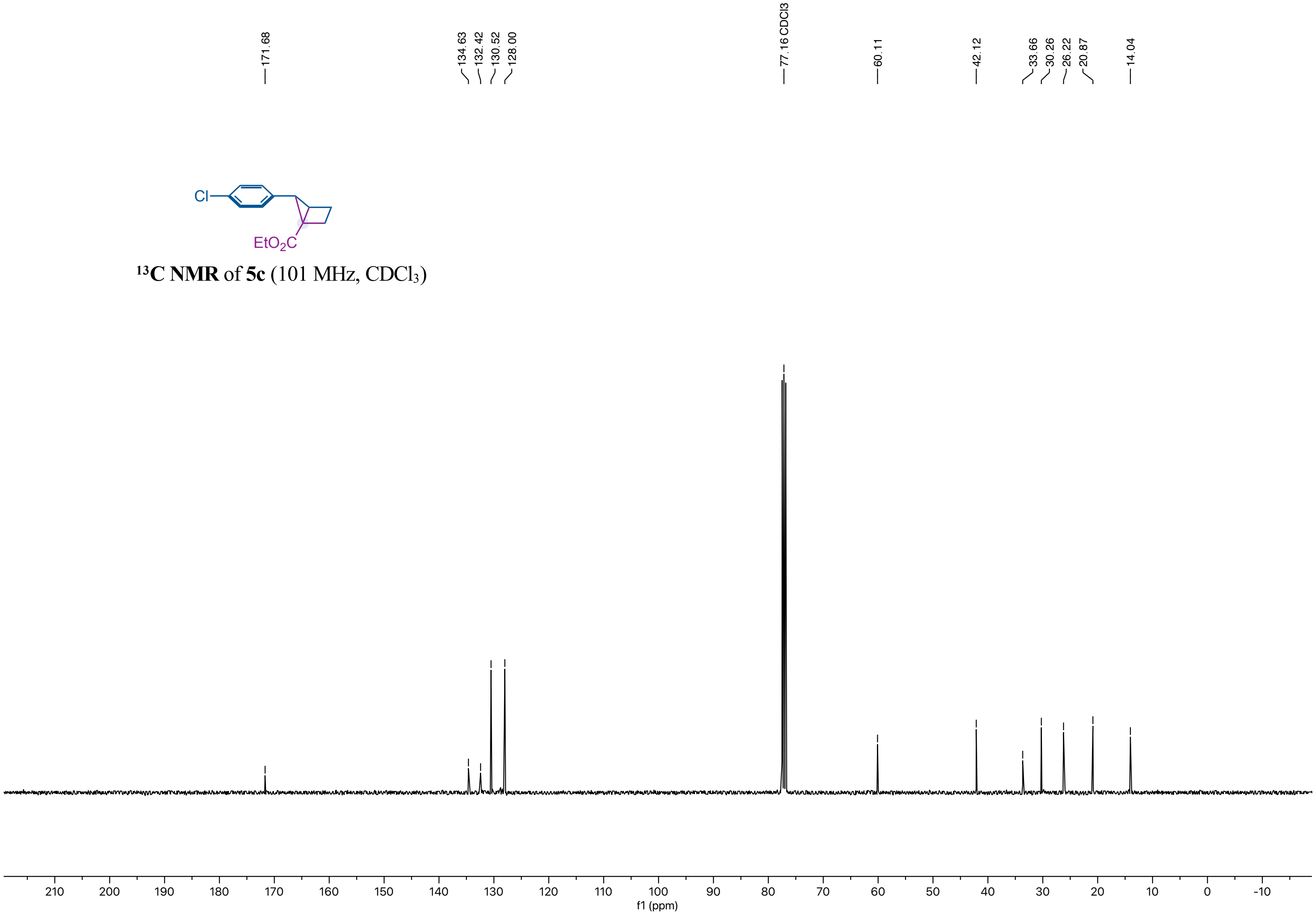

^{13}**C NMR** of **5c** (101 MHz, $CDCl_3$)

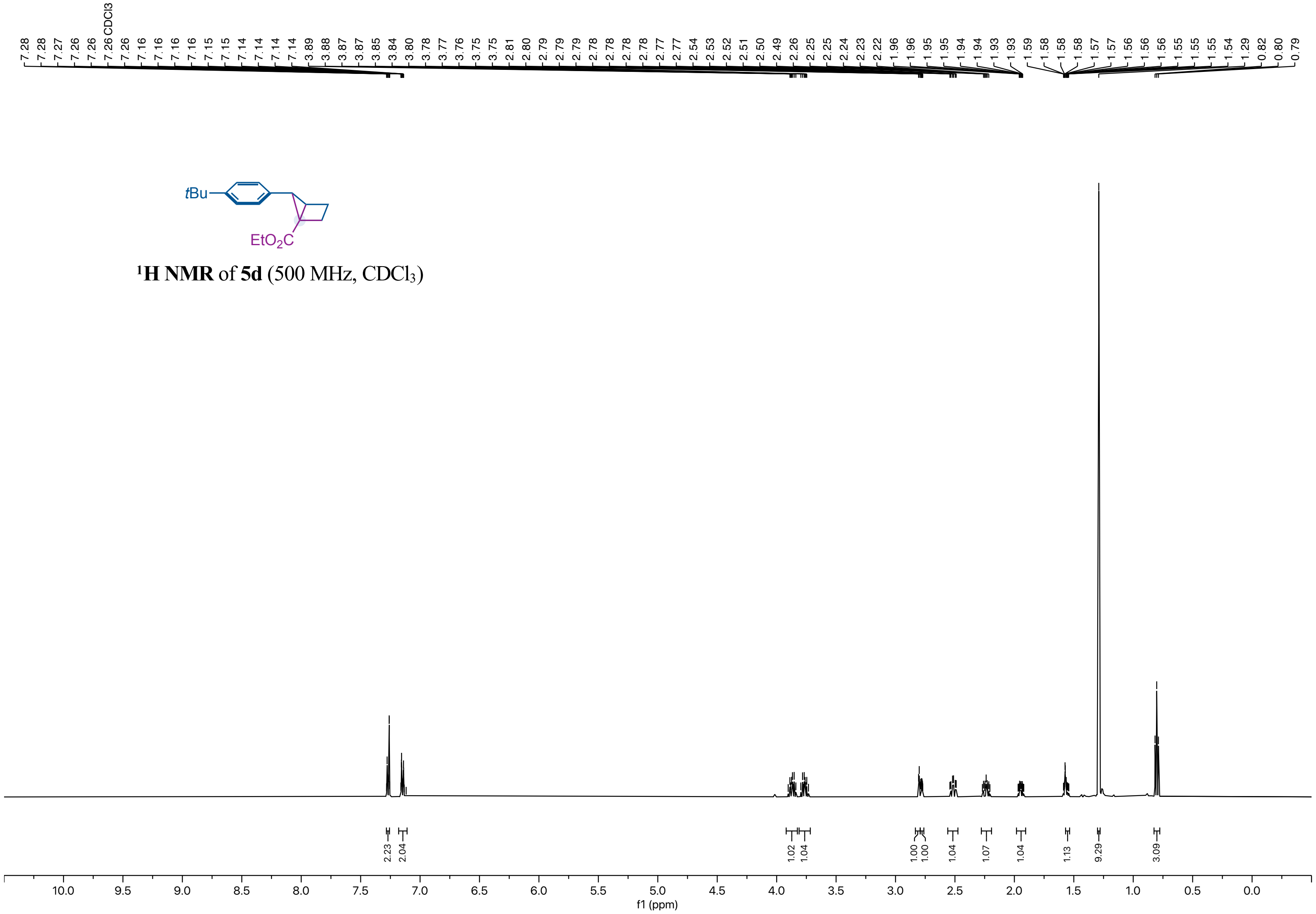

[1]**H NMR** of **5d** (500 MHz, $CDCl_3$)

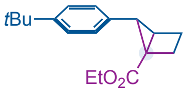

^{13}**C NMR** of **5d** (126 MHz, $CDCl_3$)

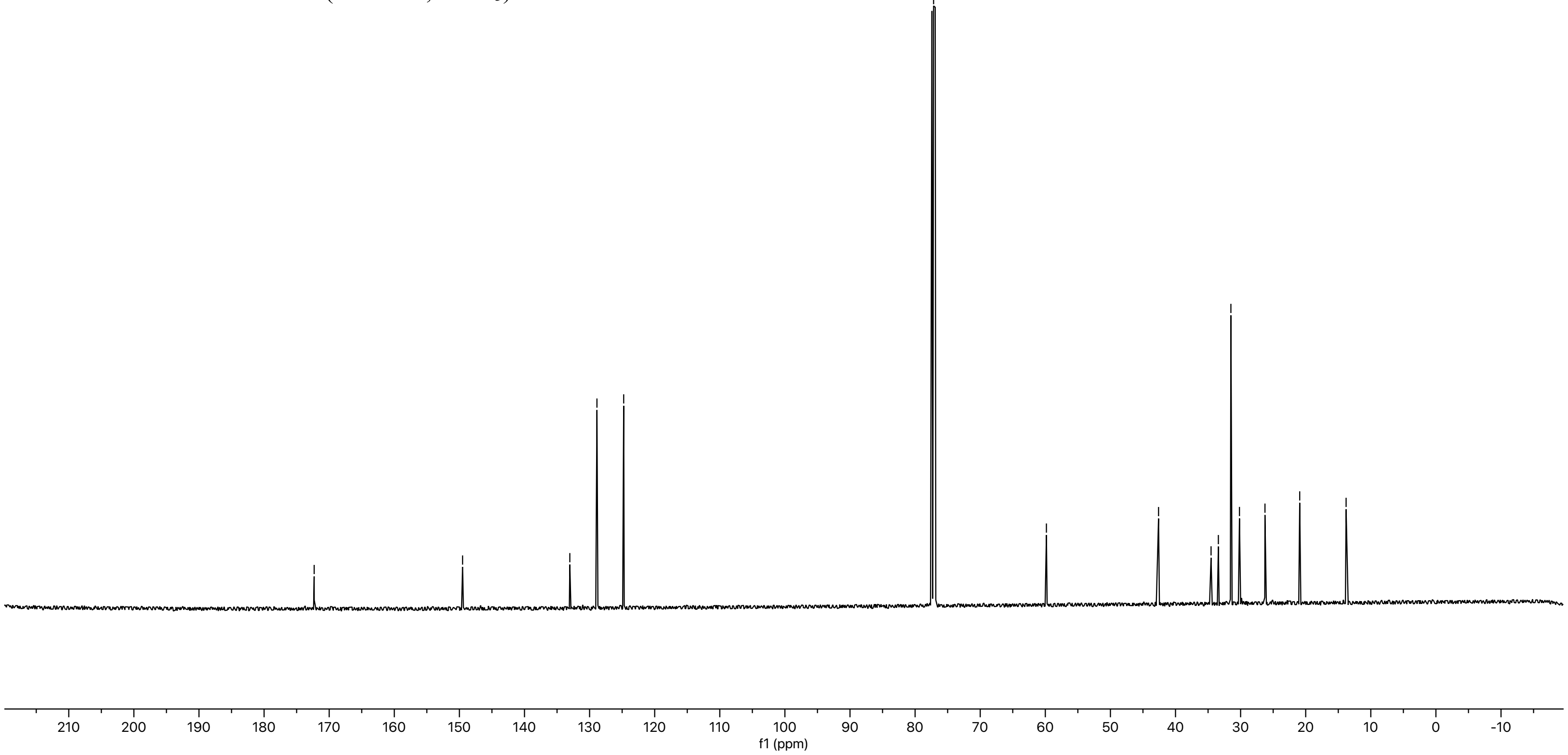

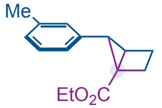

¹H NMR of **5e** (400 MHz, $CDCl_3$)

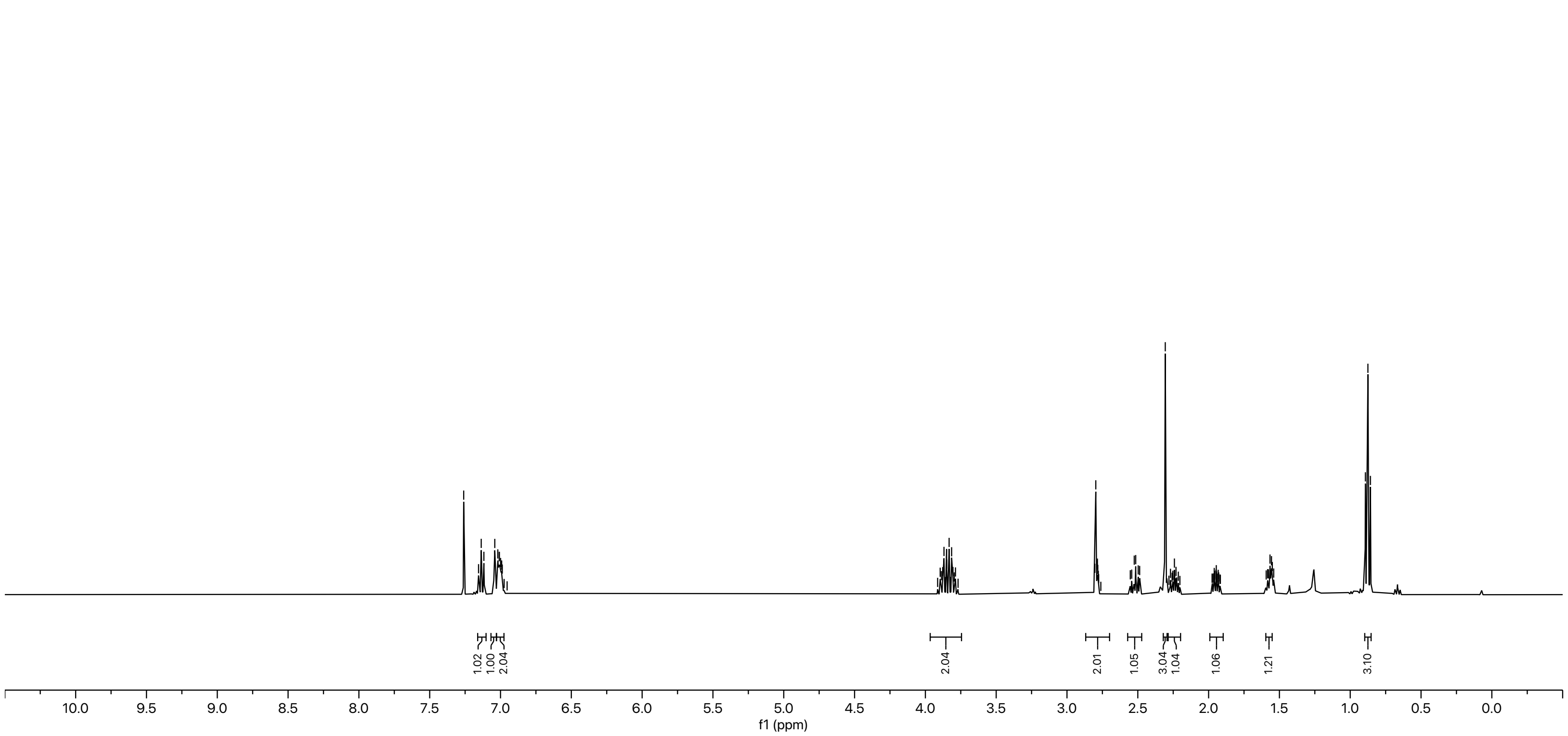

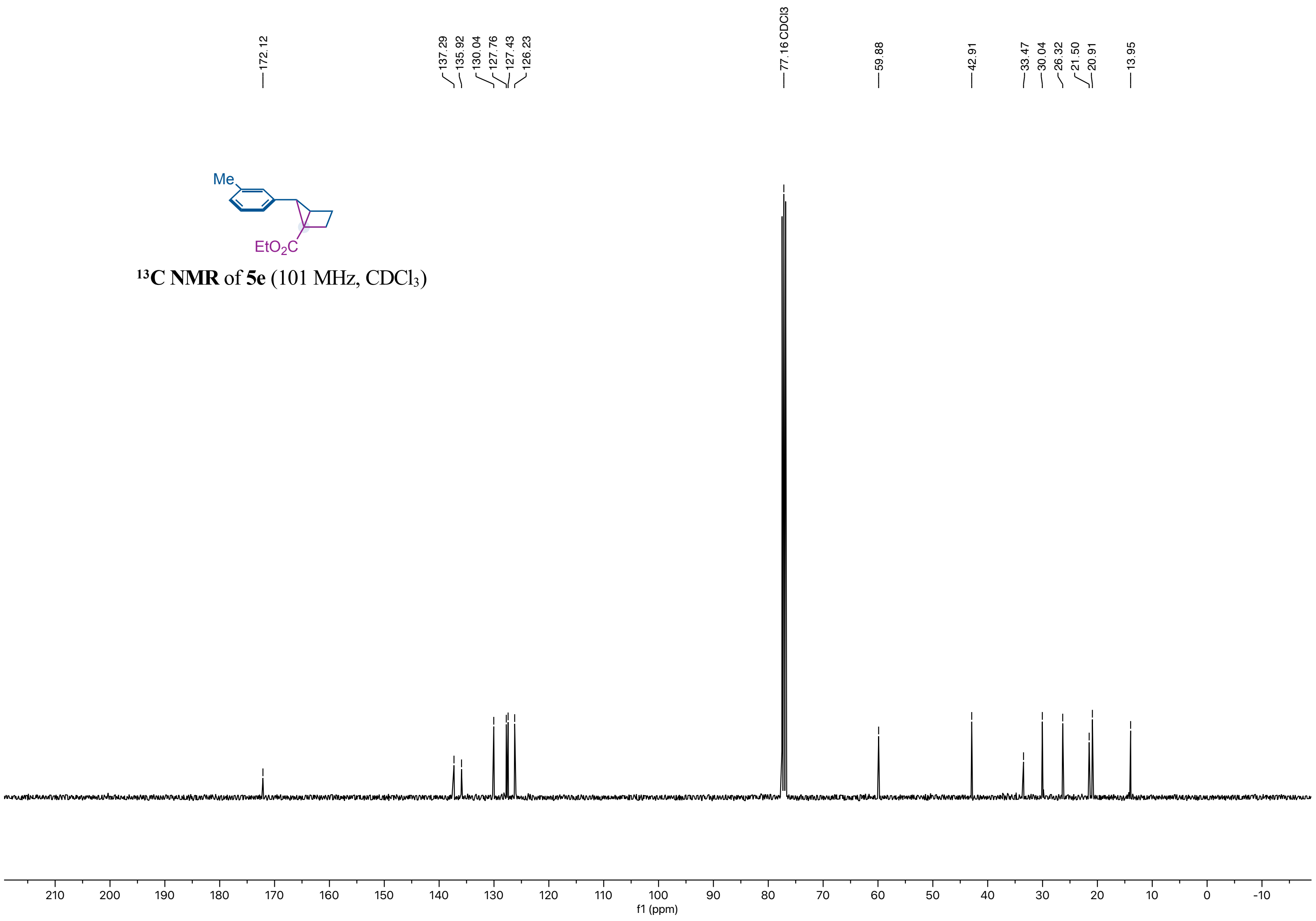

13C NMR of **5e** (101 MHz, $CDCl_3$)

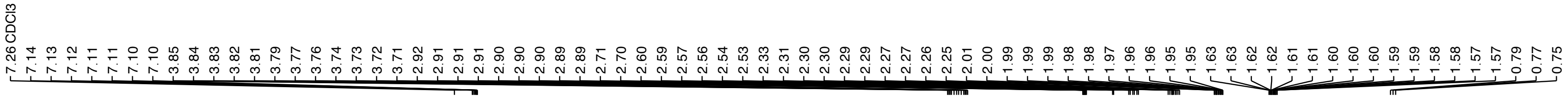

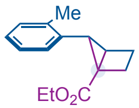

[1]**H NMR** of **5f** (400 MHz, $CDCl_3$)

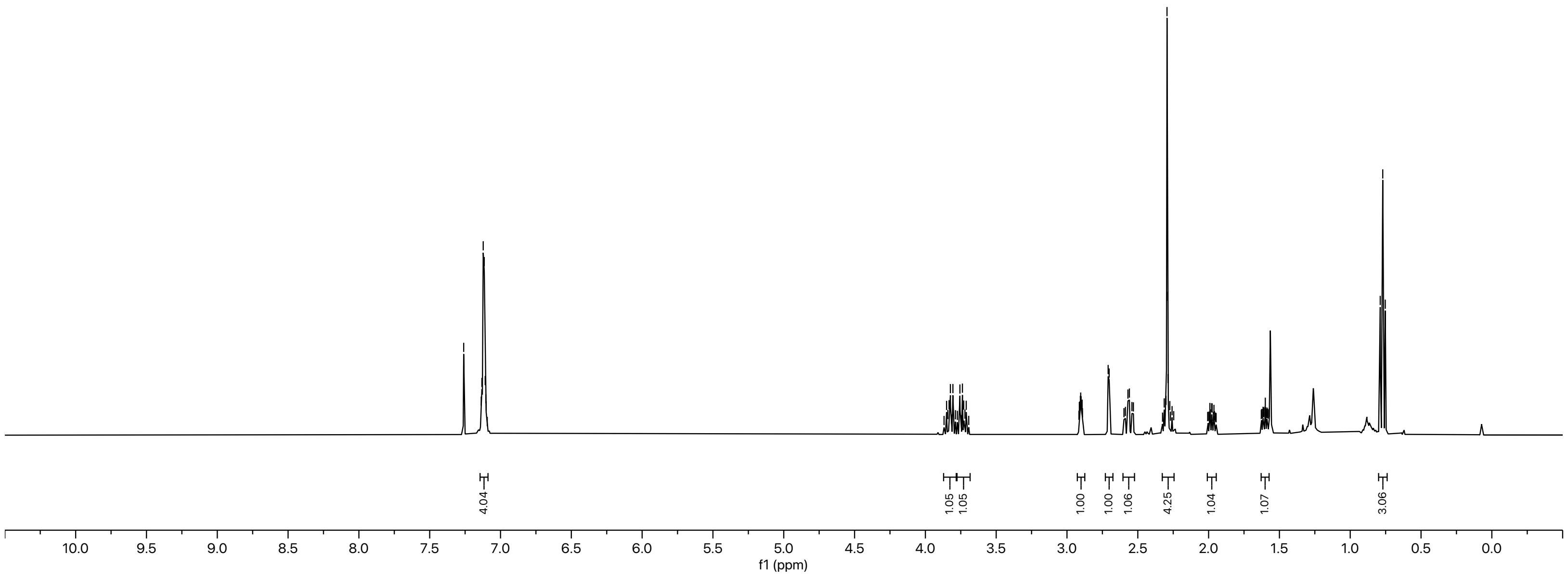

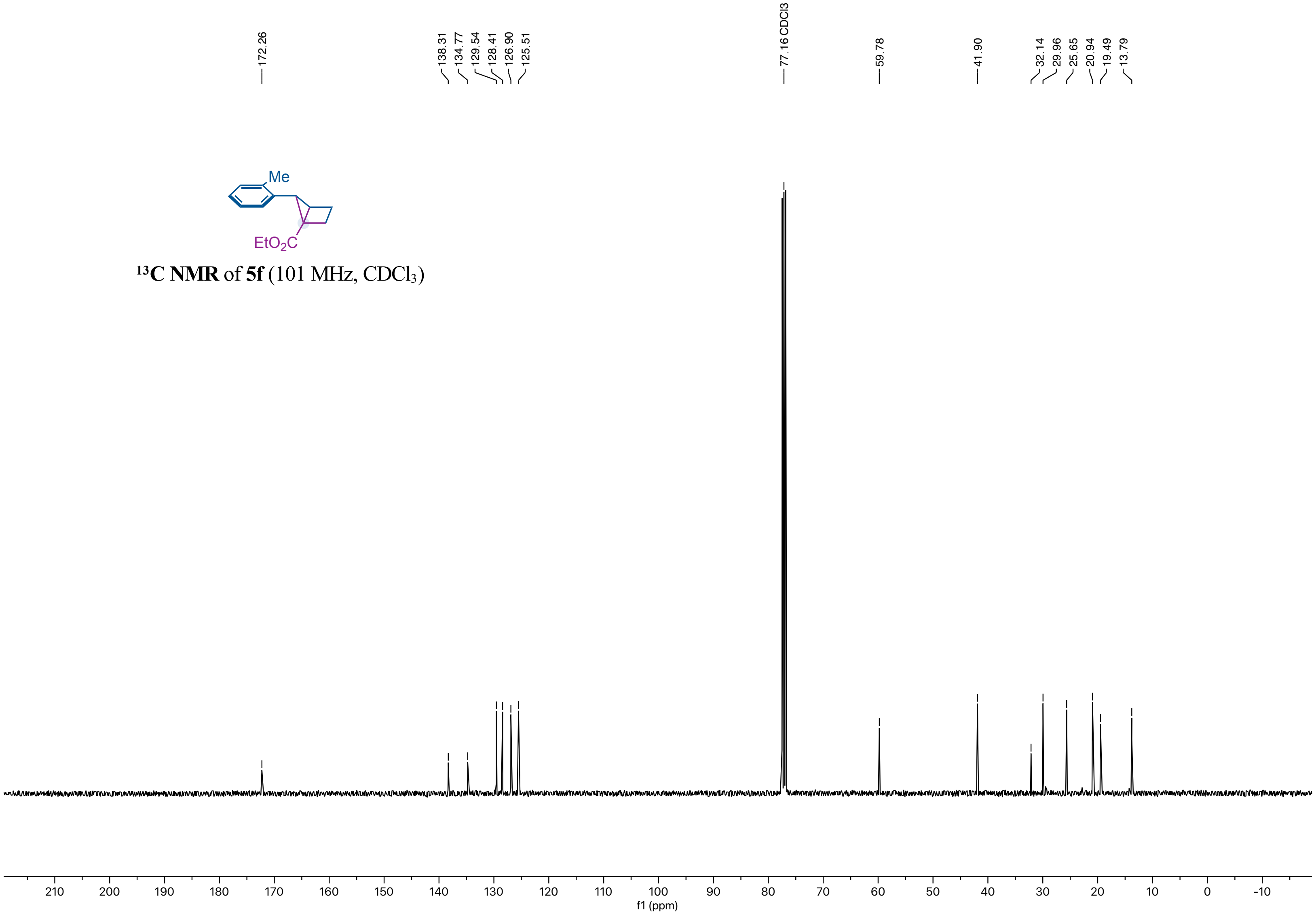

^{13}C NMR of **5f** (101 MHz, $CDCl_3$)

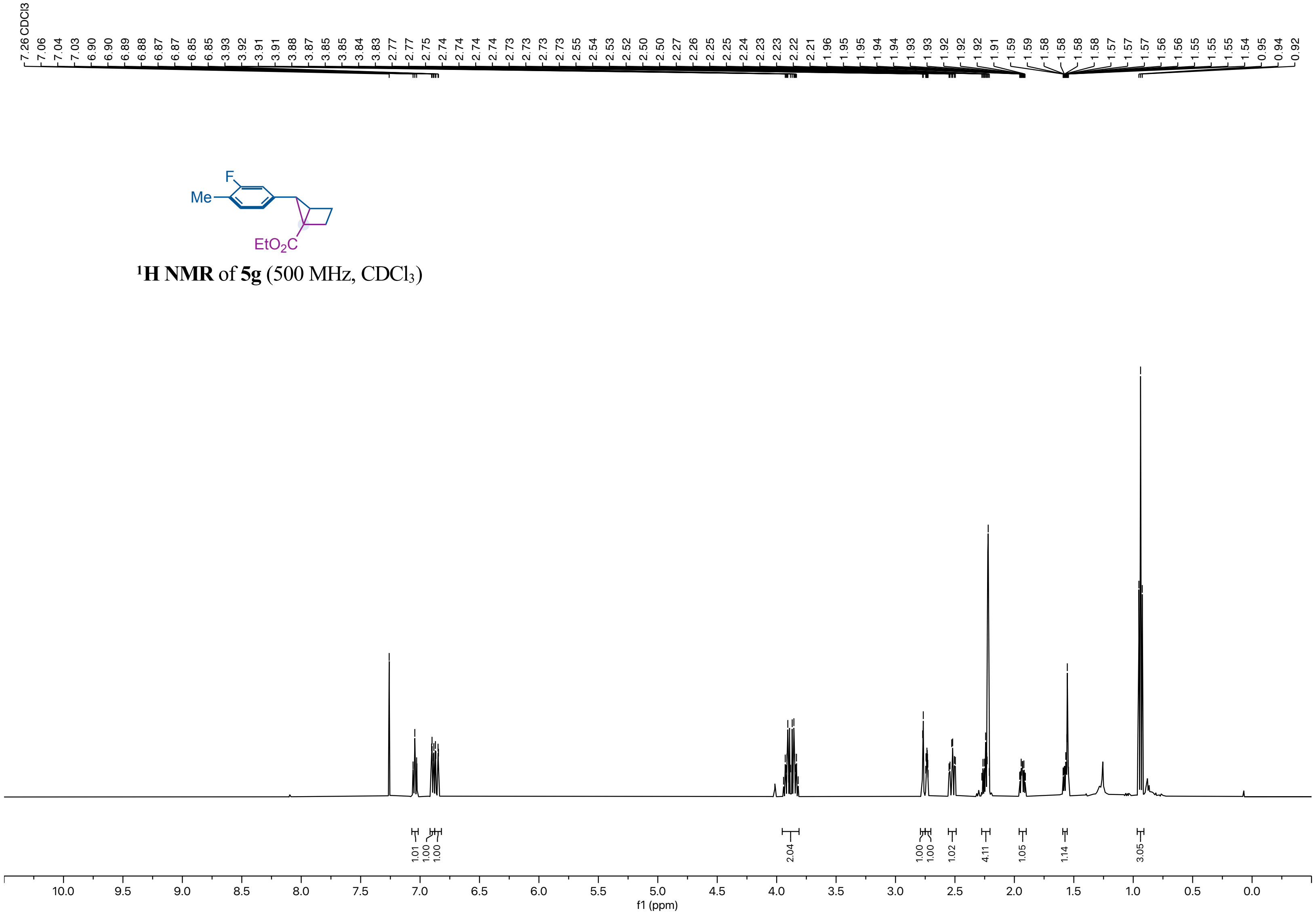

^{1}H NMR of **5g** (500 MHz, $CDCl_3$)

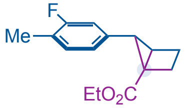

^{13}C NMR of **5g** (126 MHz, $CDCl_3$)

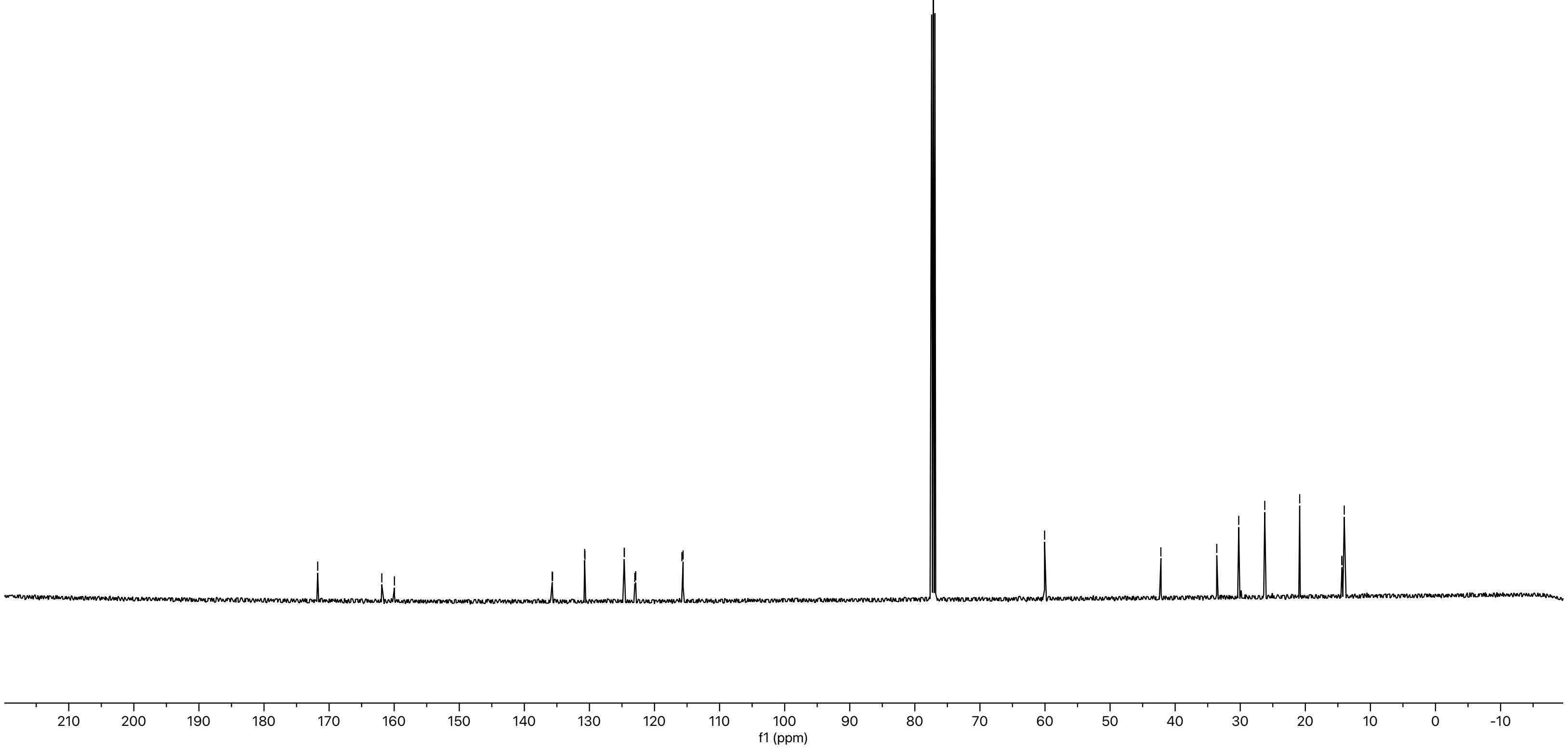

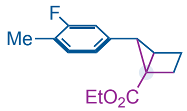

^{19}F NMR of **5g** (471 MHz, $CDCl_3$)

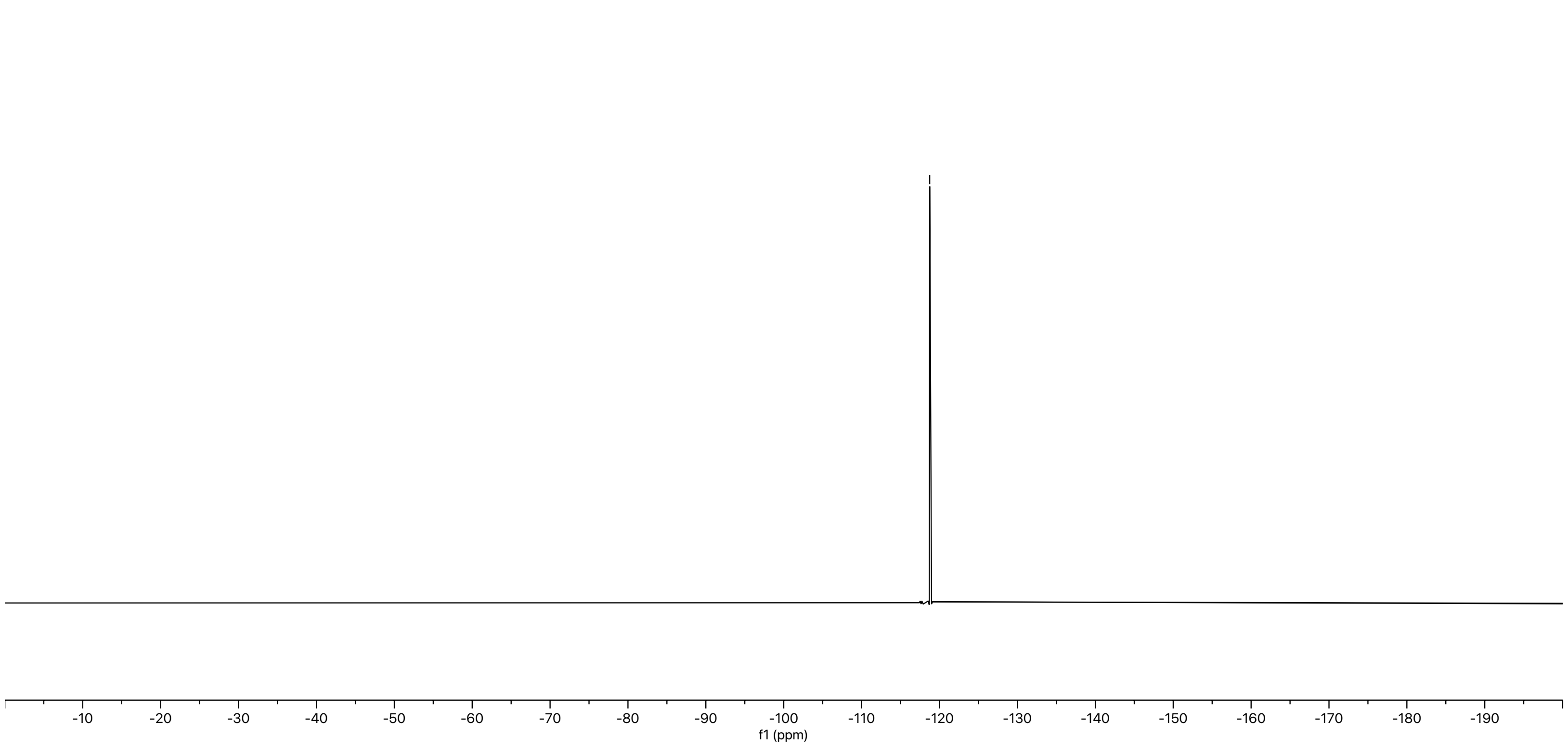

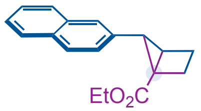

^{1}**H NMR** of **5h** (400 MHz, $CDCl_3$)

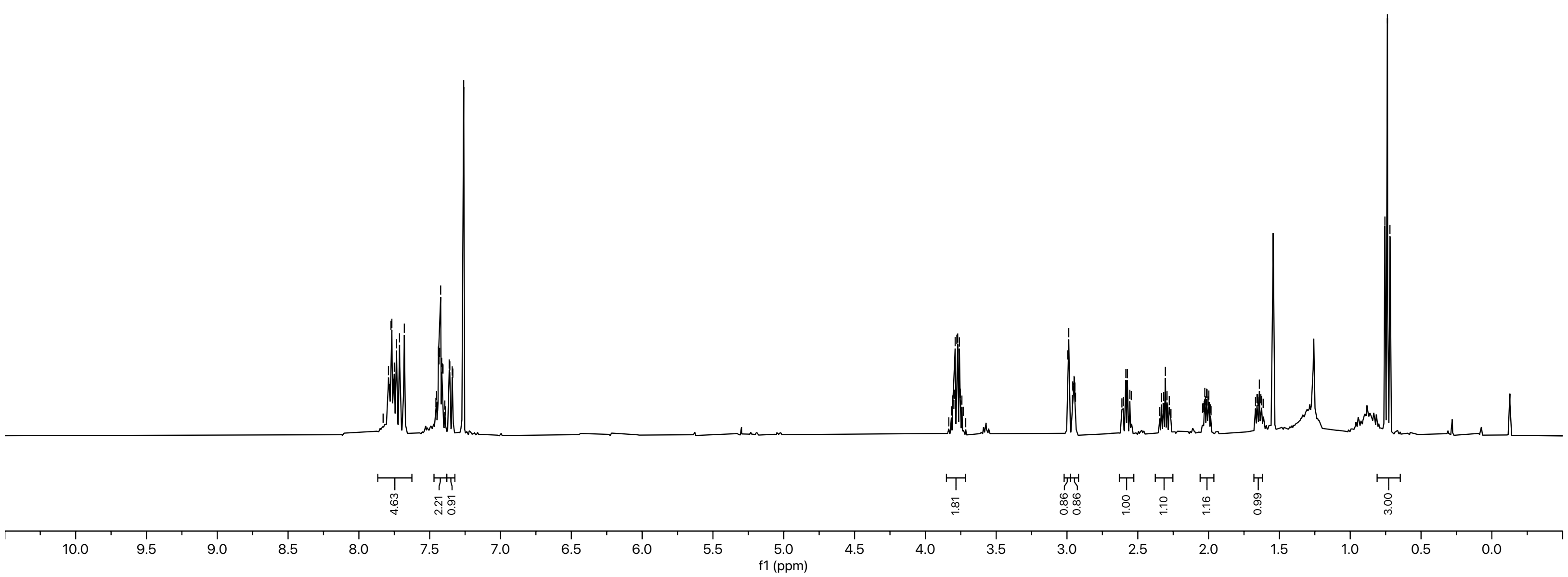

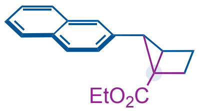

^{13}C NMR of **5h** (101 MHz, $CDCl_3$)

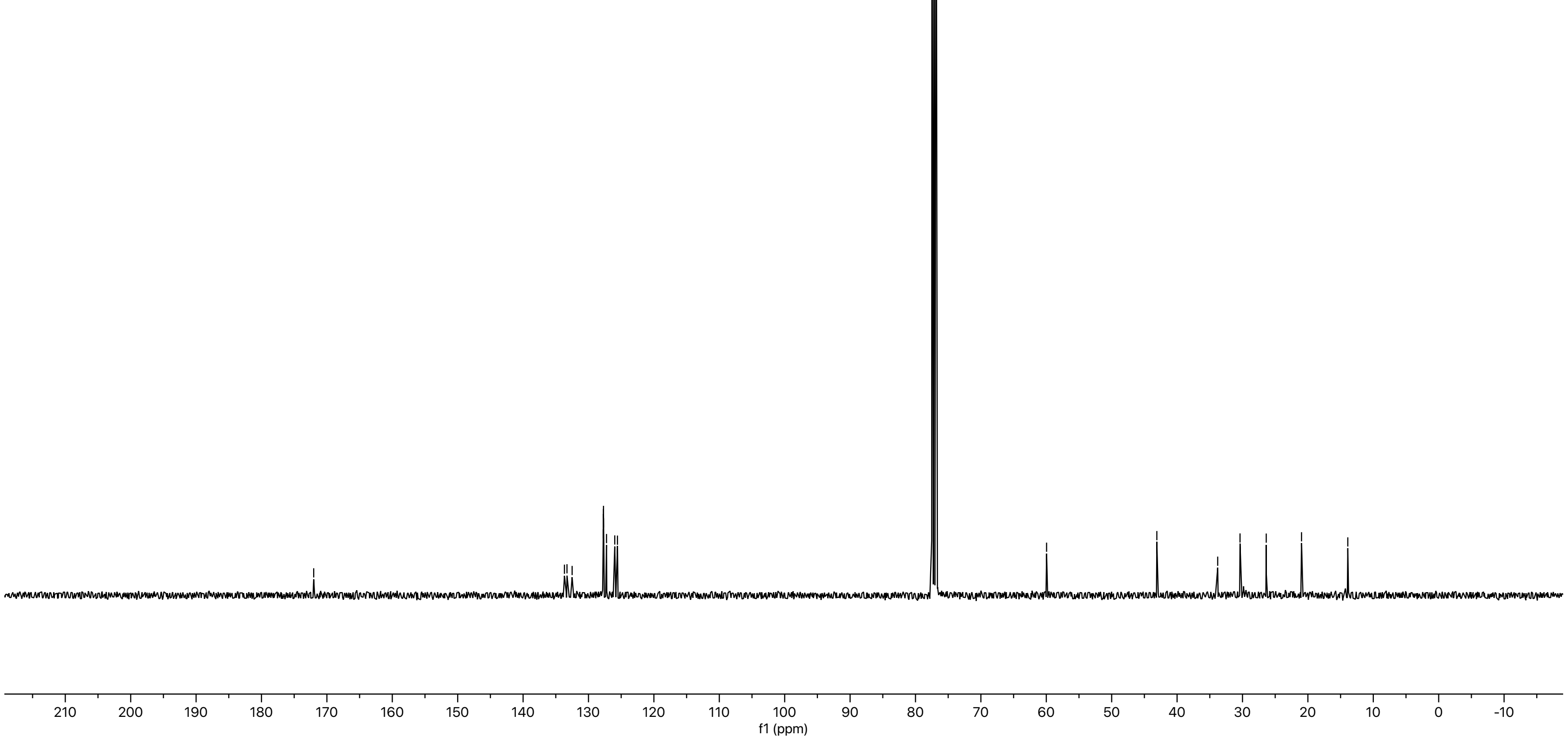

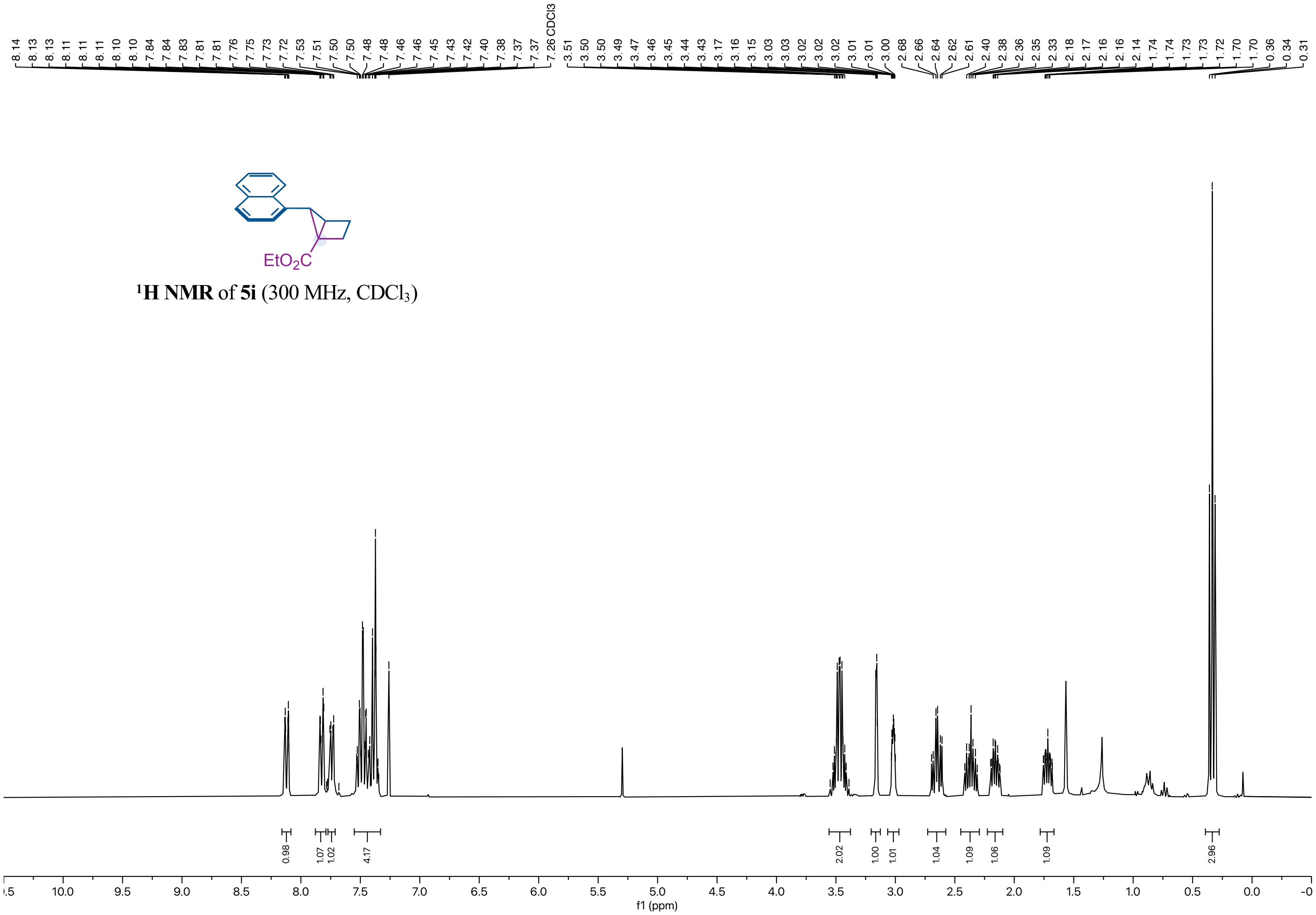

^{1}H NMR of 5i (300 MHz, $CDCl_3$)

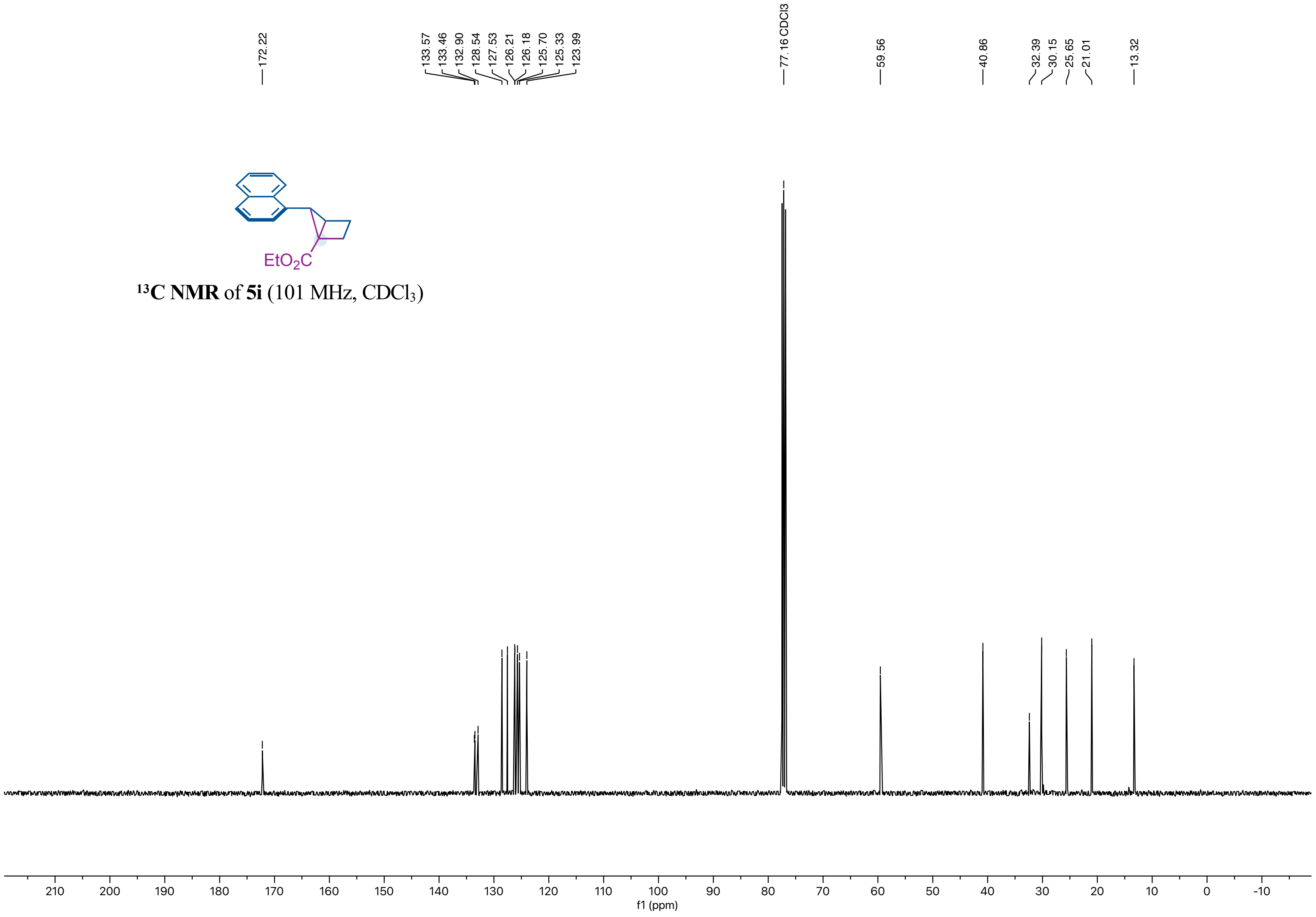

^{13}C NMR of **5i** (101 MHz, $CDCl_3$)

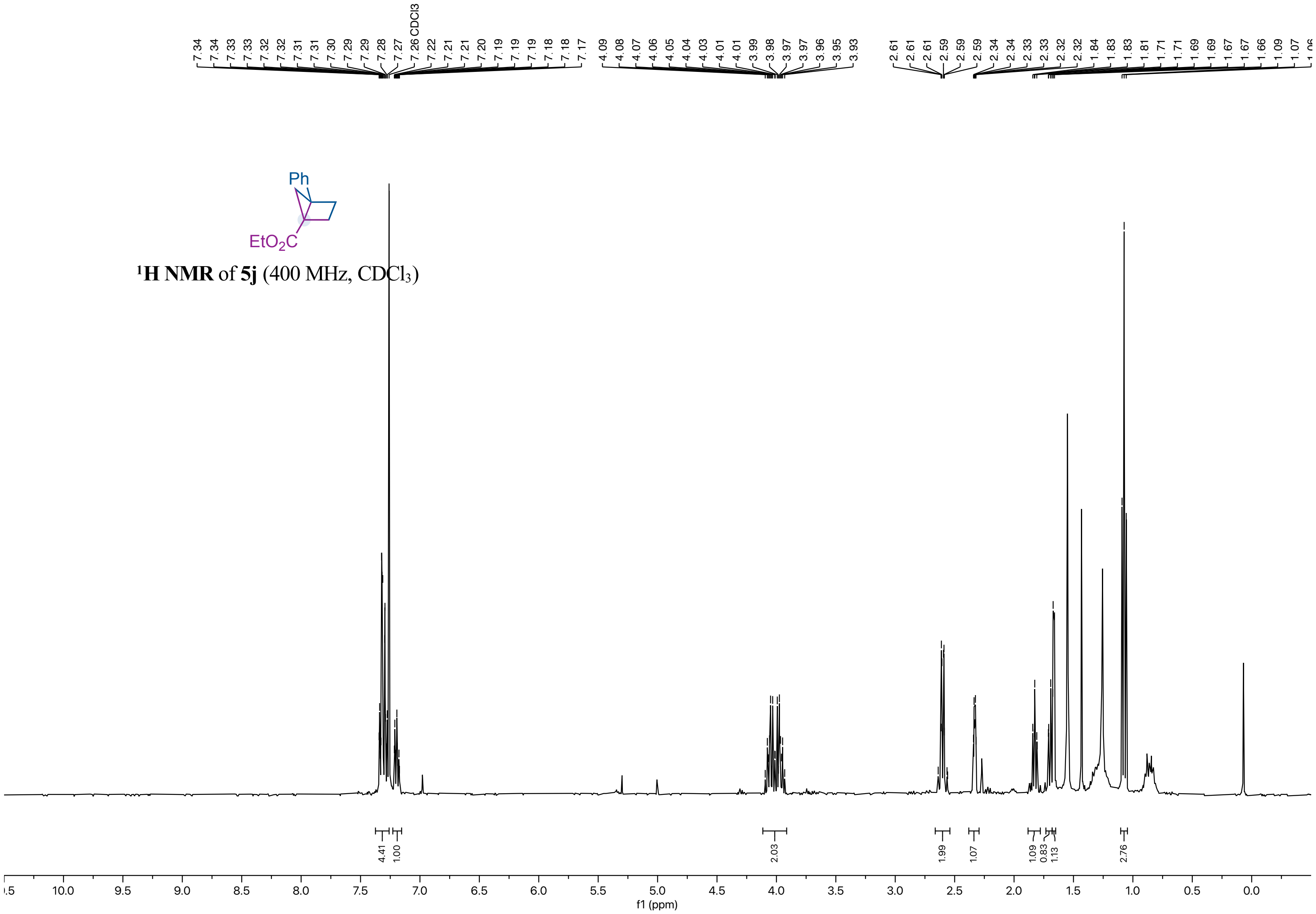

^{1}**H NMR** of **5j** (400 MHz, $CDCl_3$)

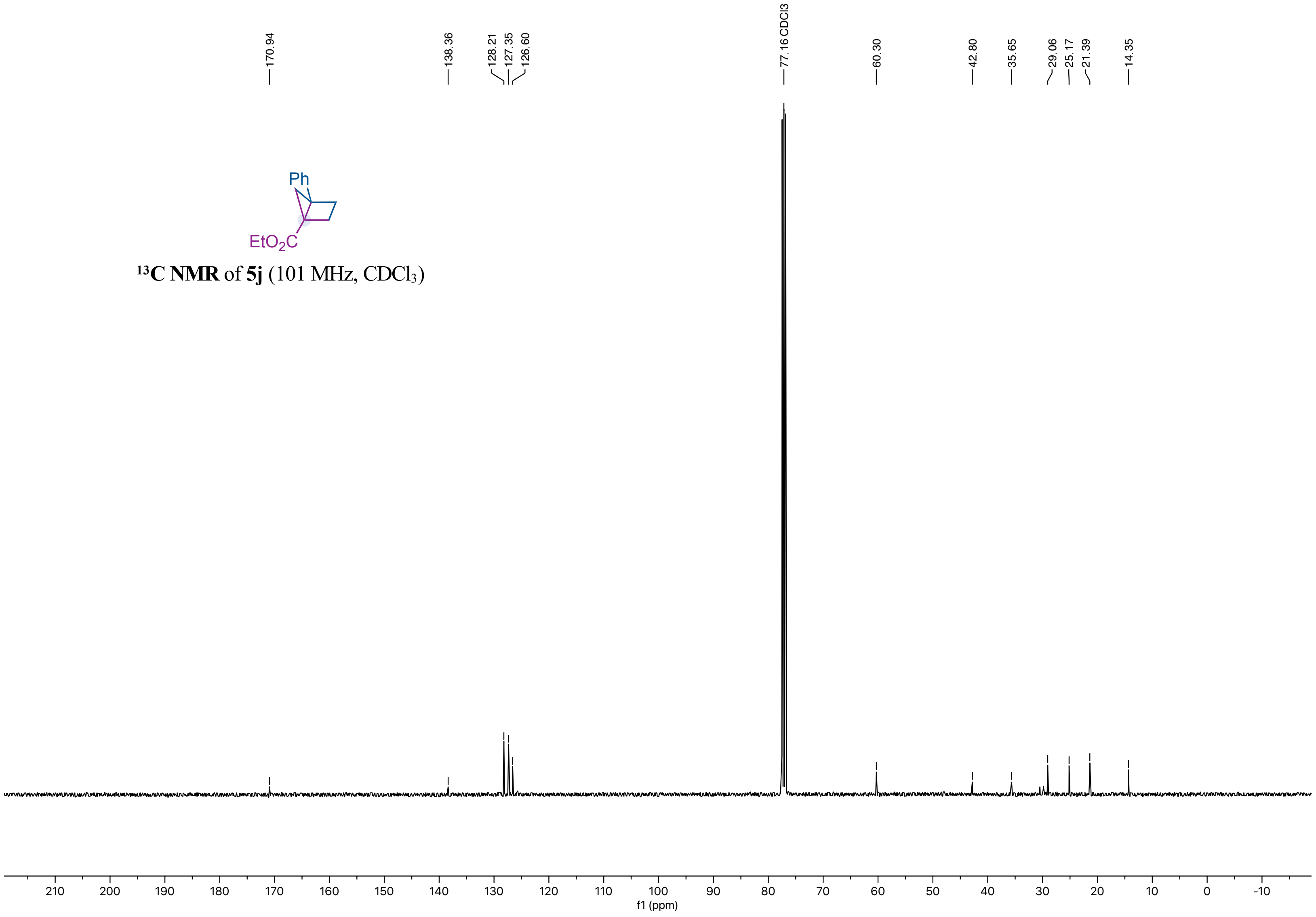

^{13}C NMR of **5j** (101 MHz, $CDCl_3$)

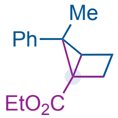

^{1}H NMR of **5k** (400 MHz, $CDCl_3$)

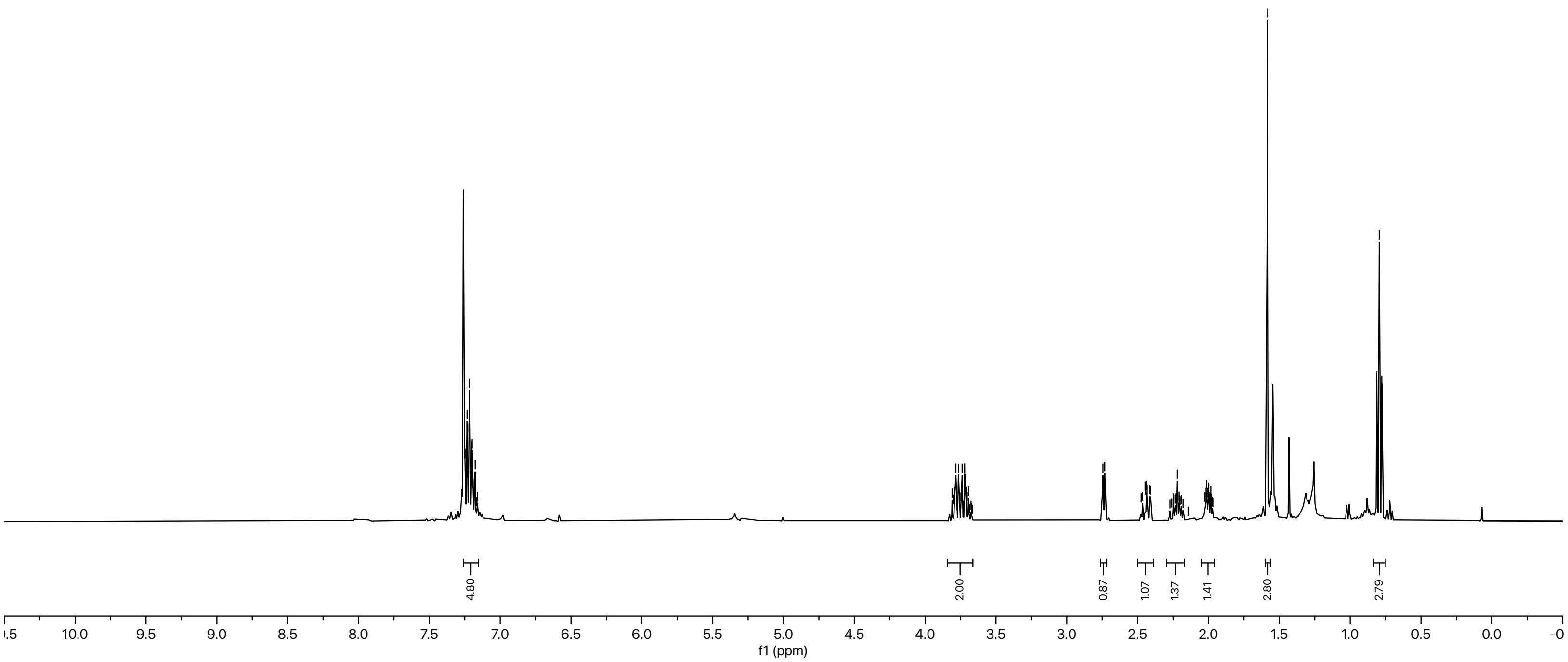

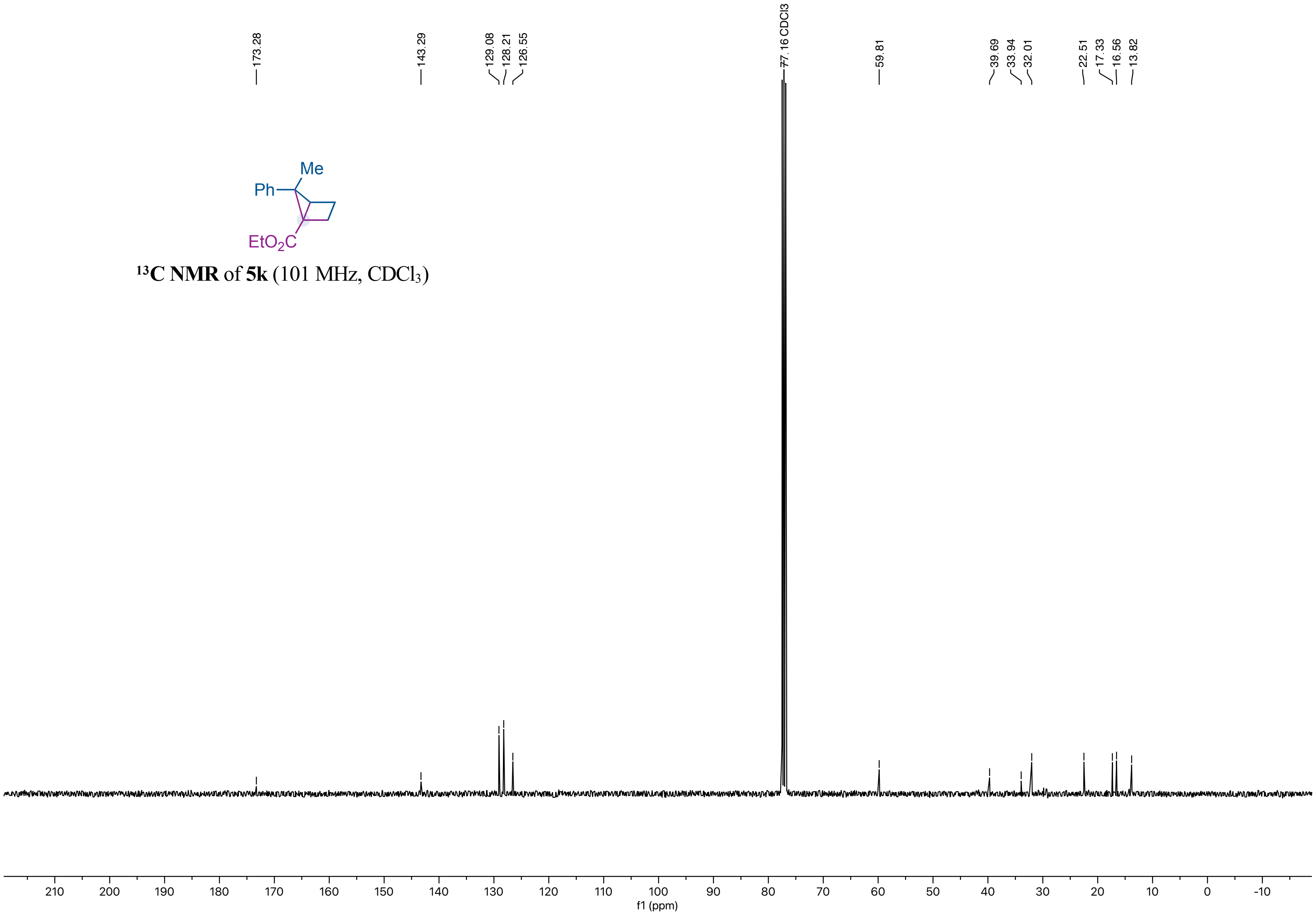

^{13}C NMR of **5k** (101 MHz, $CDCl_3$)

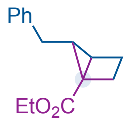

[1]**H NMR** of **5l** (400 MHz, $CDCl_3$)

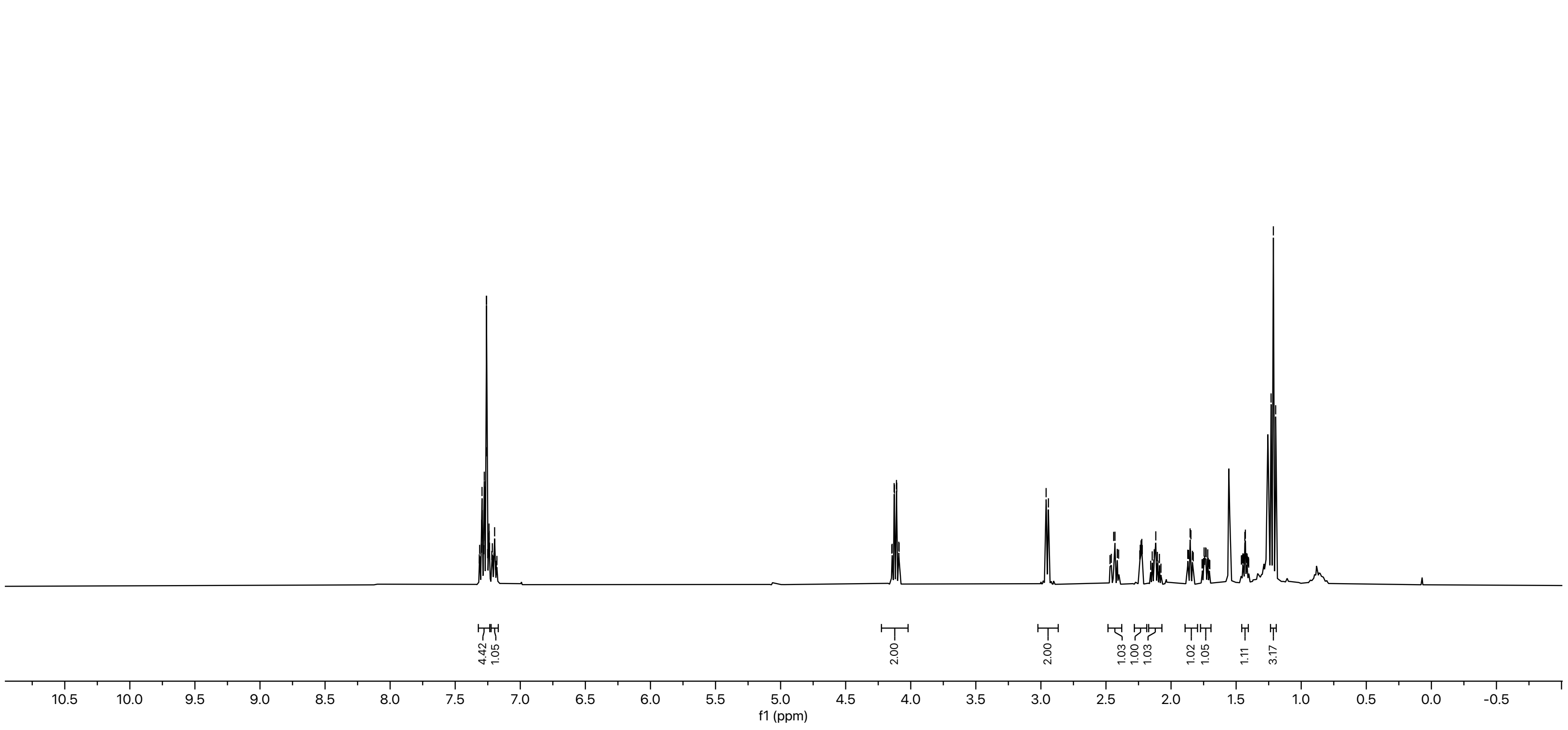

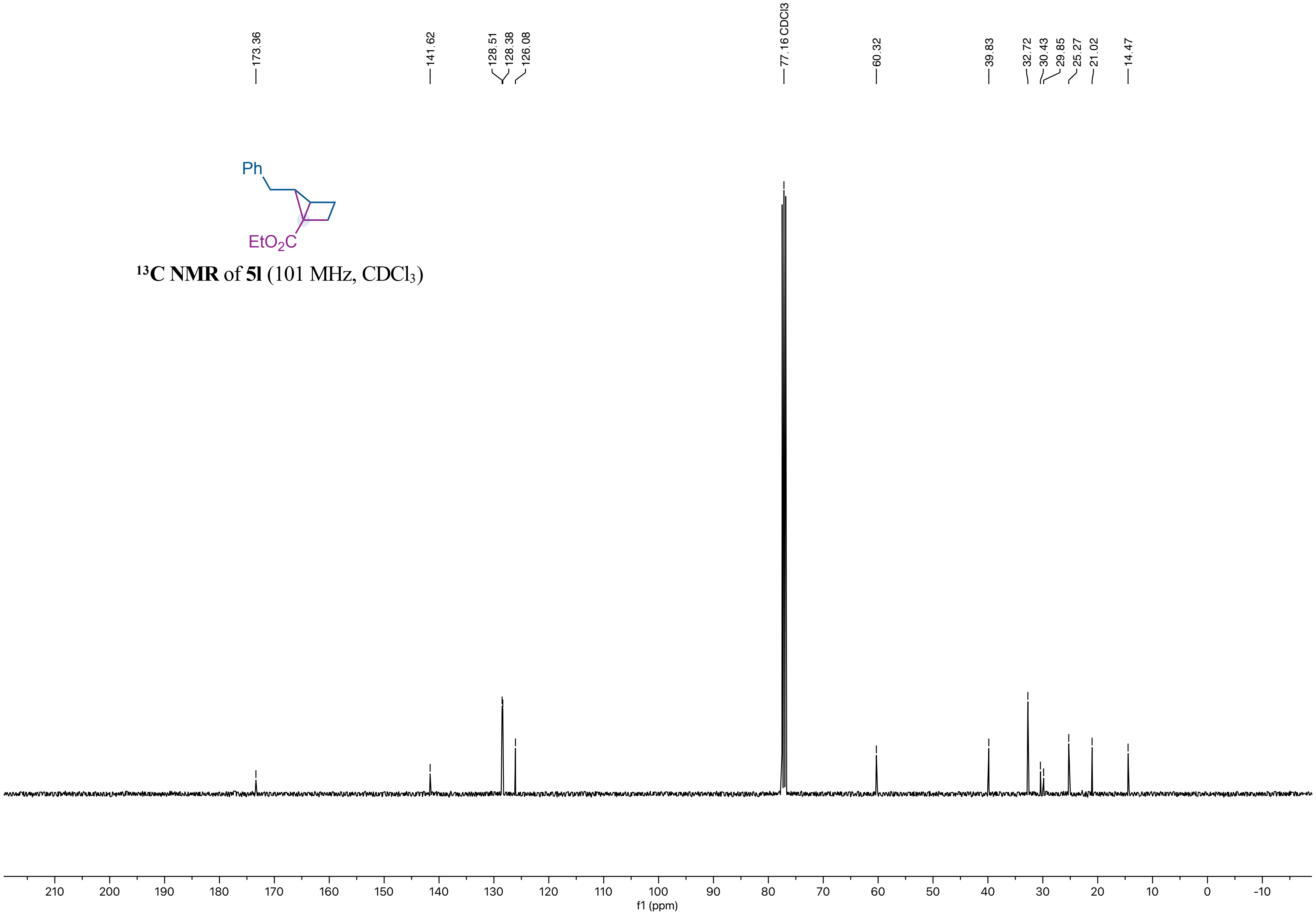

^{13}C NMR of **5l** (101 MHz, $CDCl_3$)

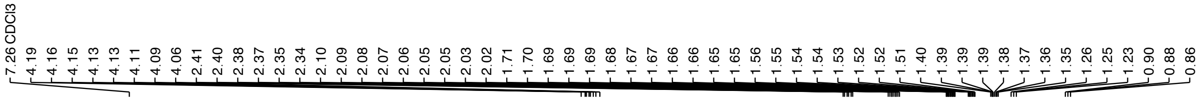

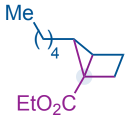

[1]**H NMR** of **5m** (400 MHz, $CDCl_3$)

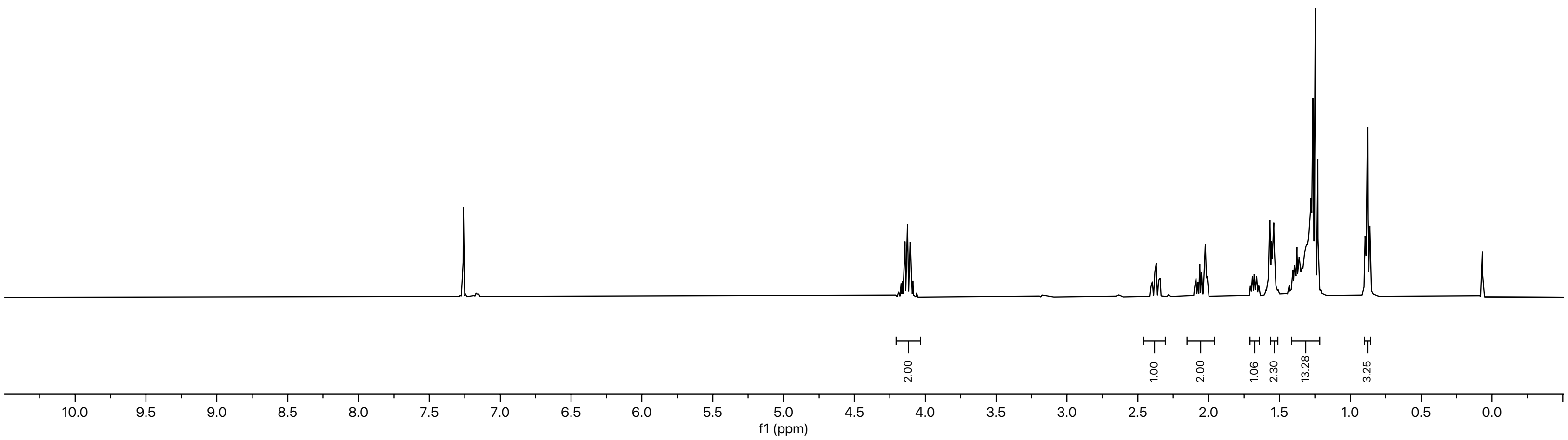

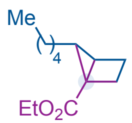

^{13}**C NMR** of **5m** (101 MHz, $CDCl_3$)

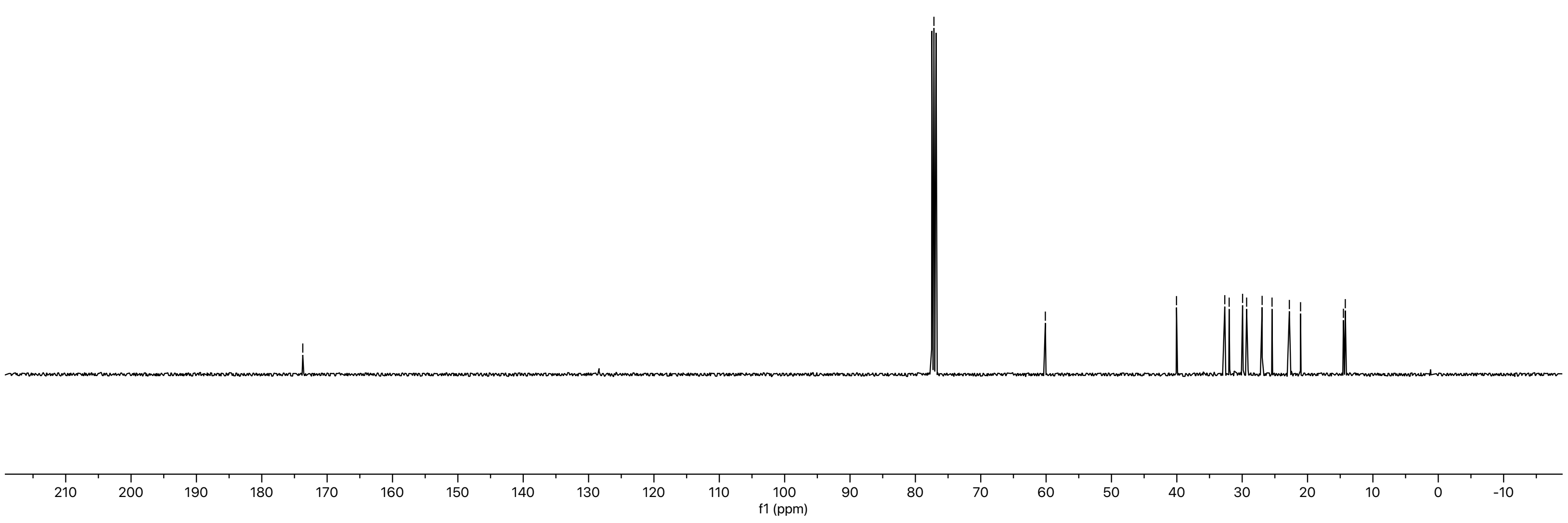

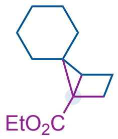

¹H NMR of **5n** (500 MHz, $CDCl_3$)

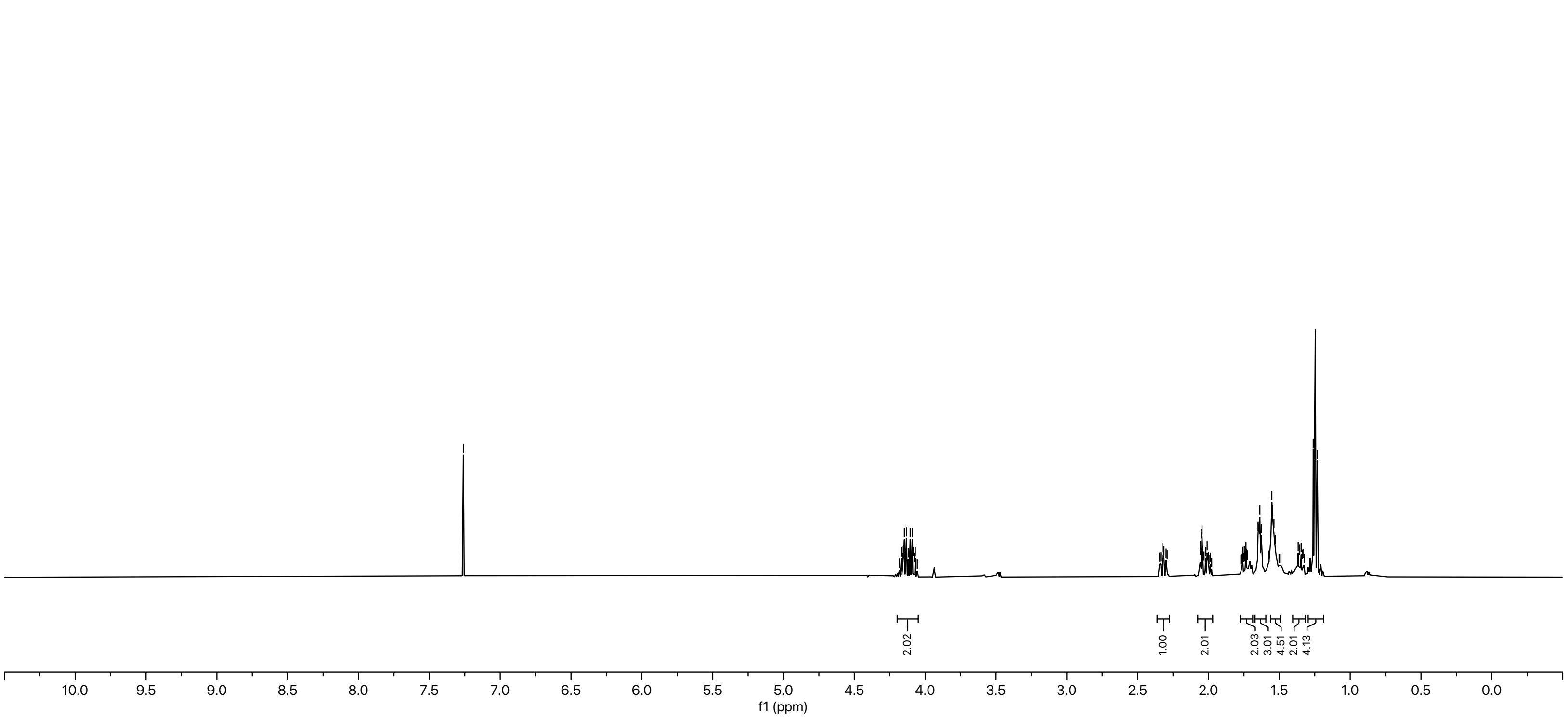

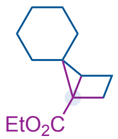

^{13}C NMR of **5n** (126 MHz, $CDCl_3$)

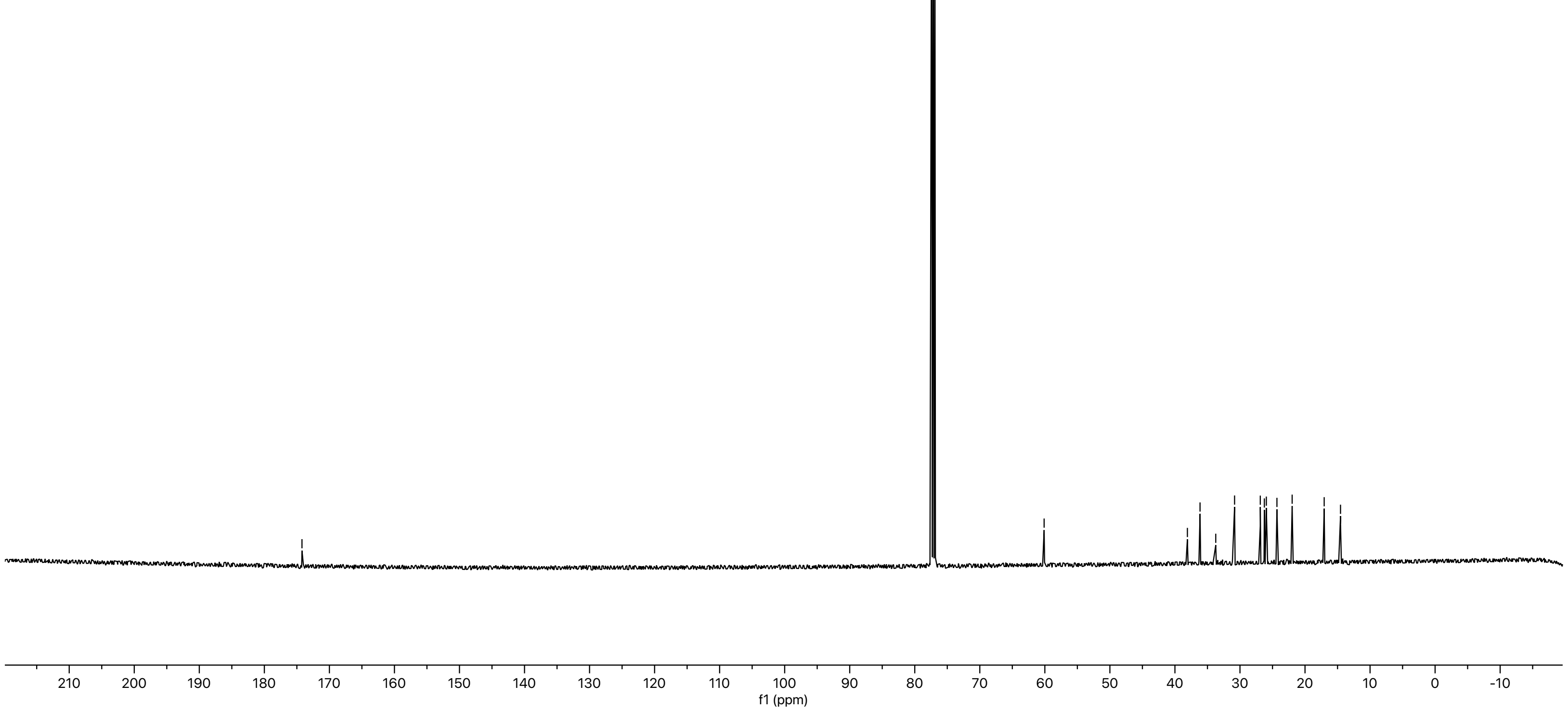

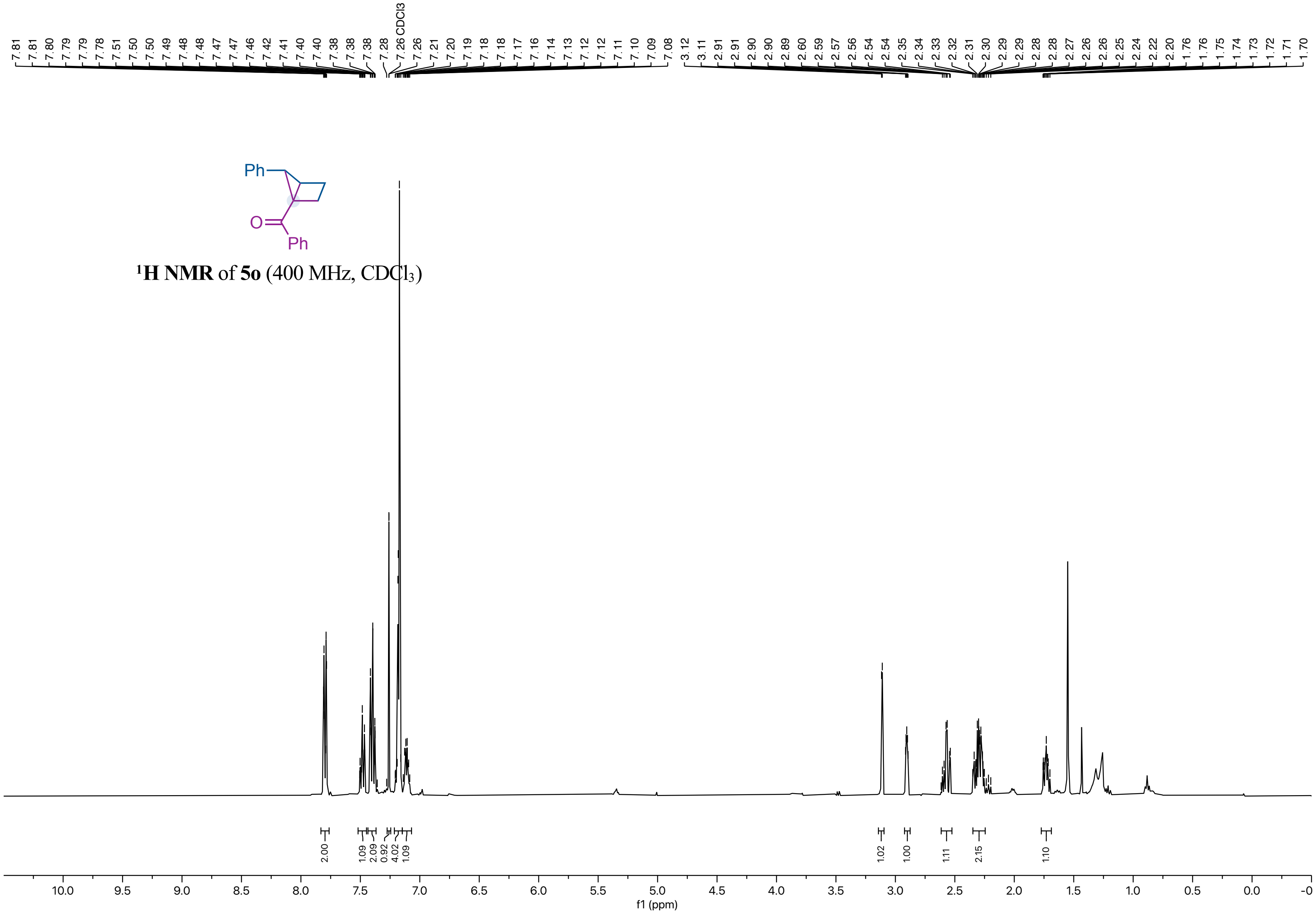

^{1}H NMR of **5o** (400 MHz, $CDCl_3$)

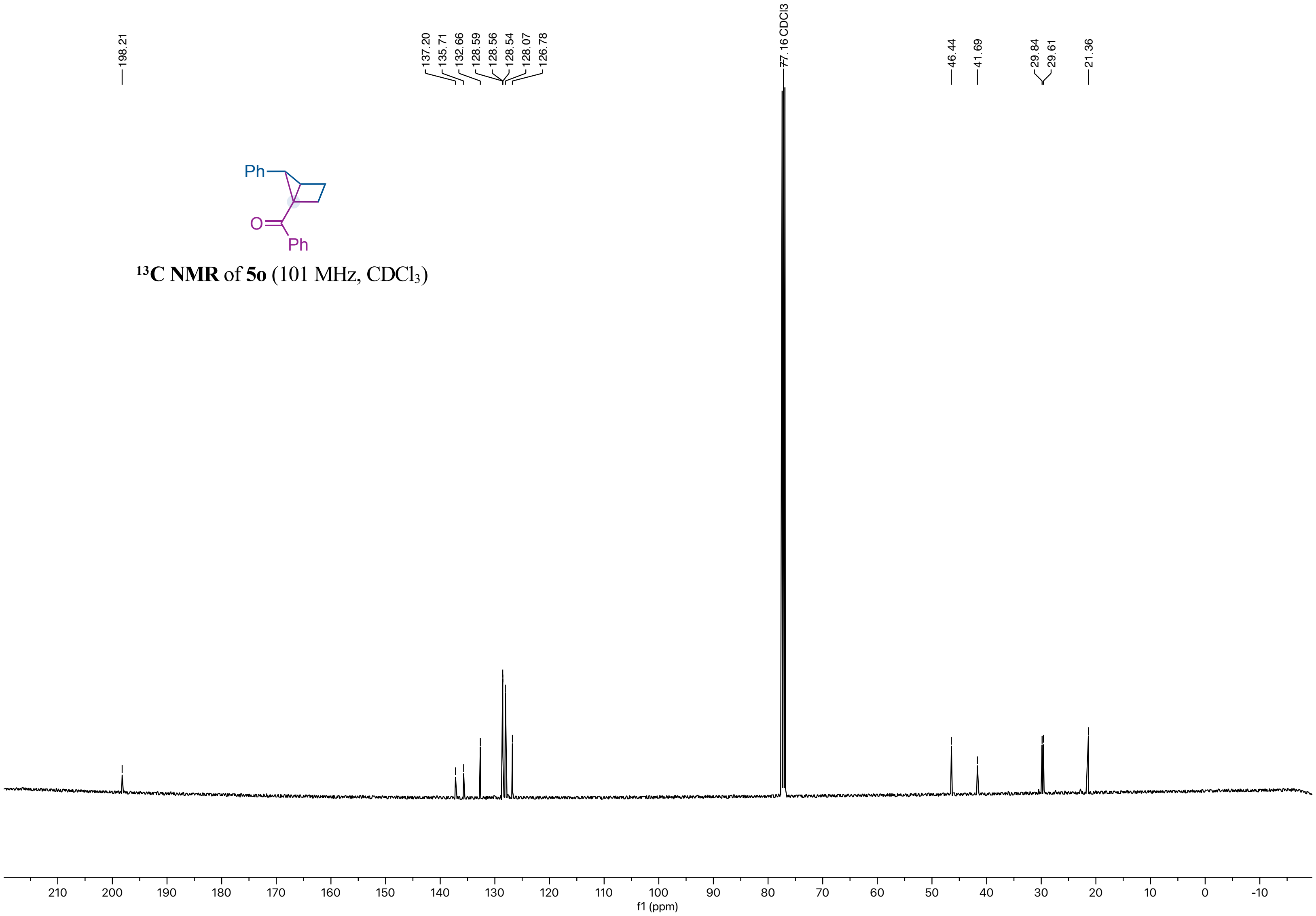

[13]**C NMR** of **5o** (101 MHz, $CDCl_3$)

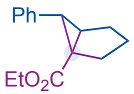

^{1}**H NMR** of **6** (500 MHz, $CDCl_3$)

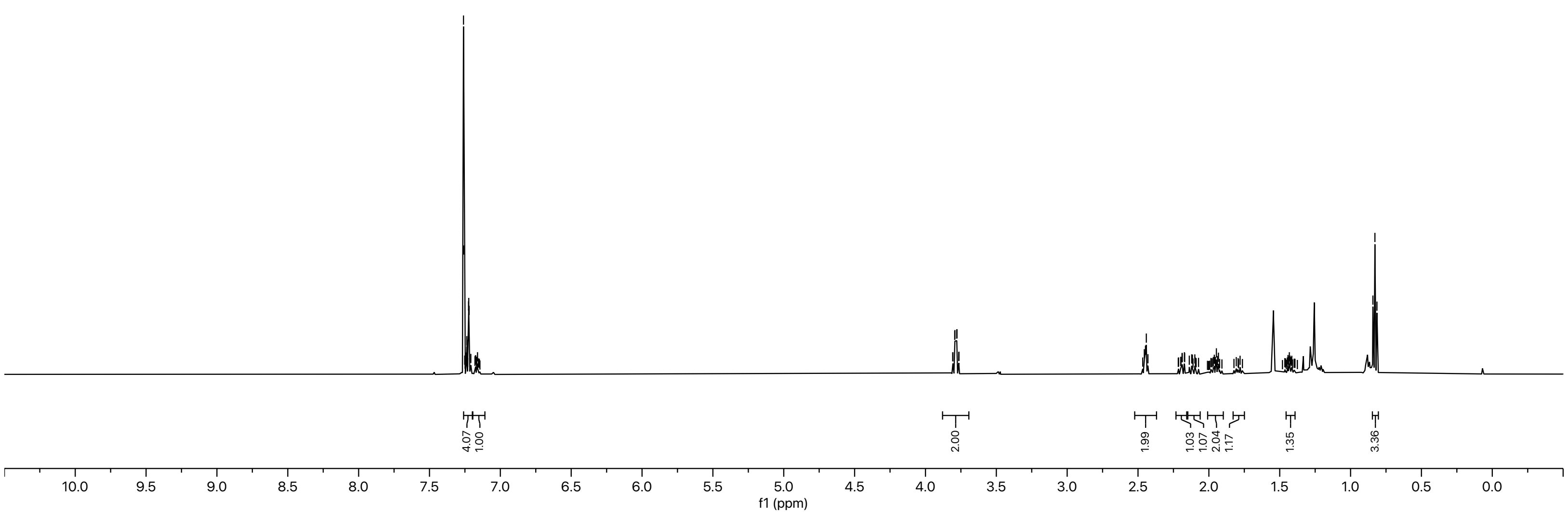

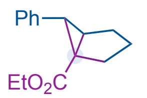

^{13}C NMR of **6** (126 MHz, $CDCl_3$)

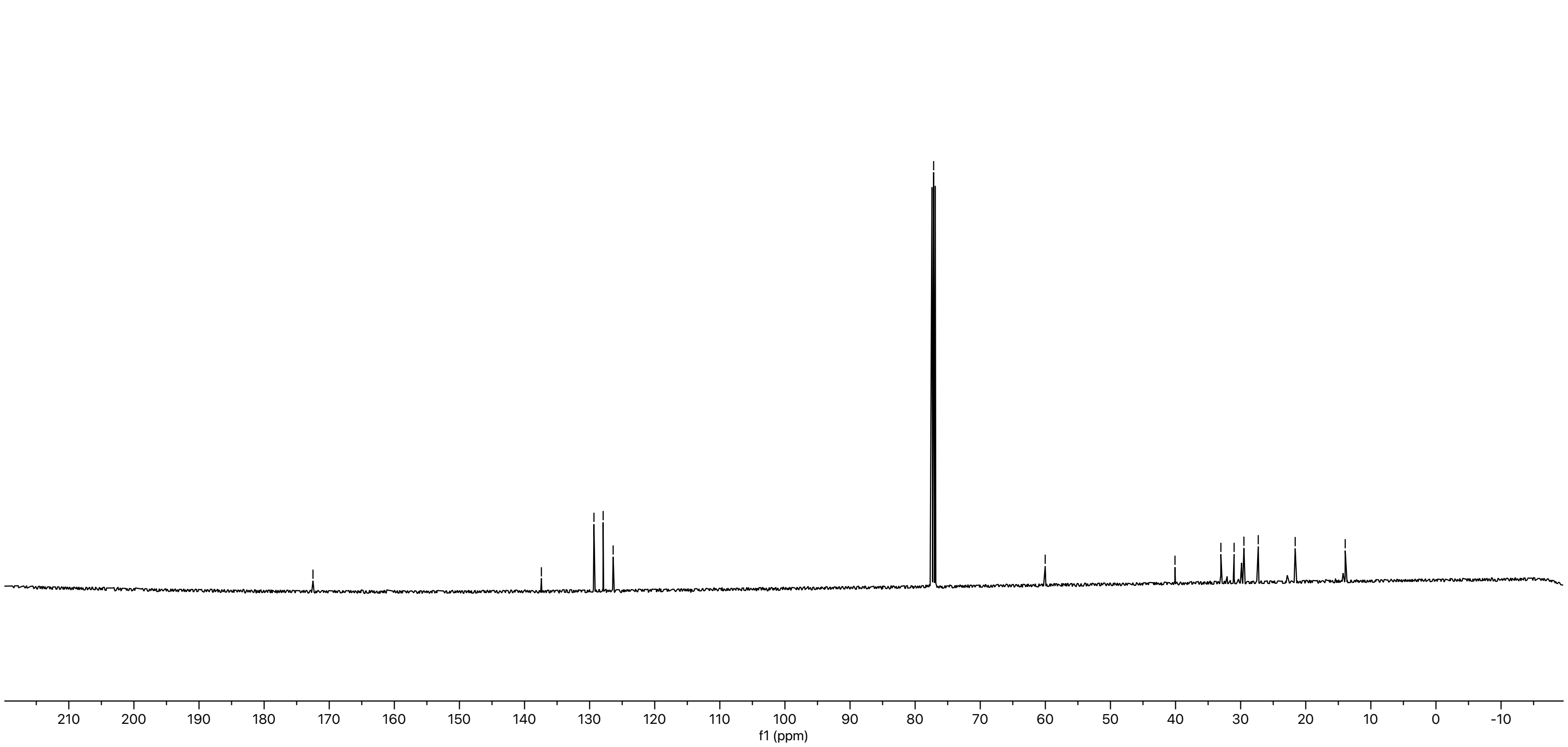

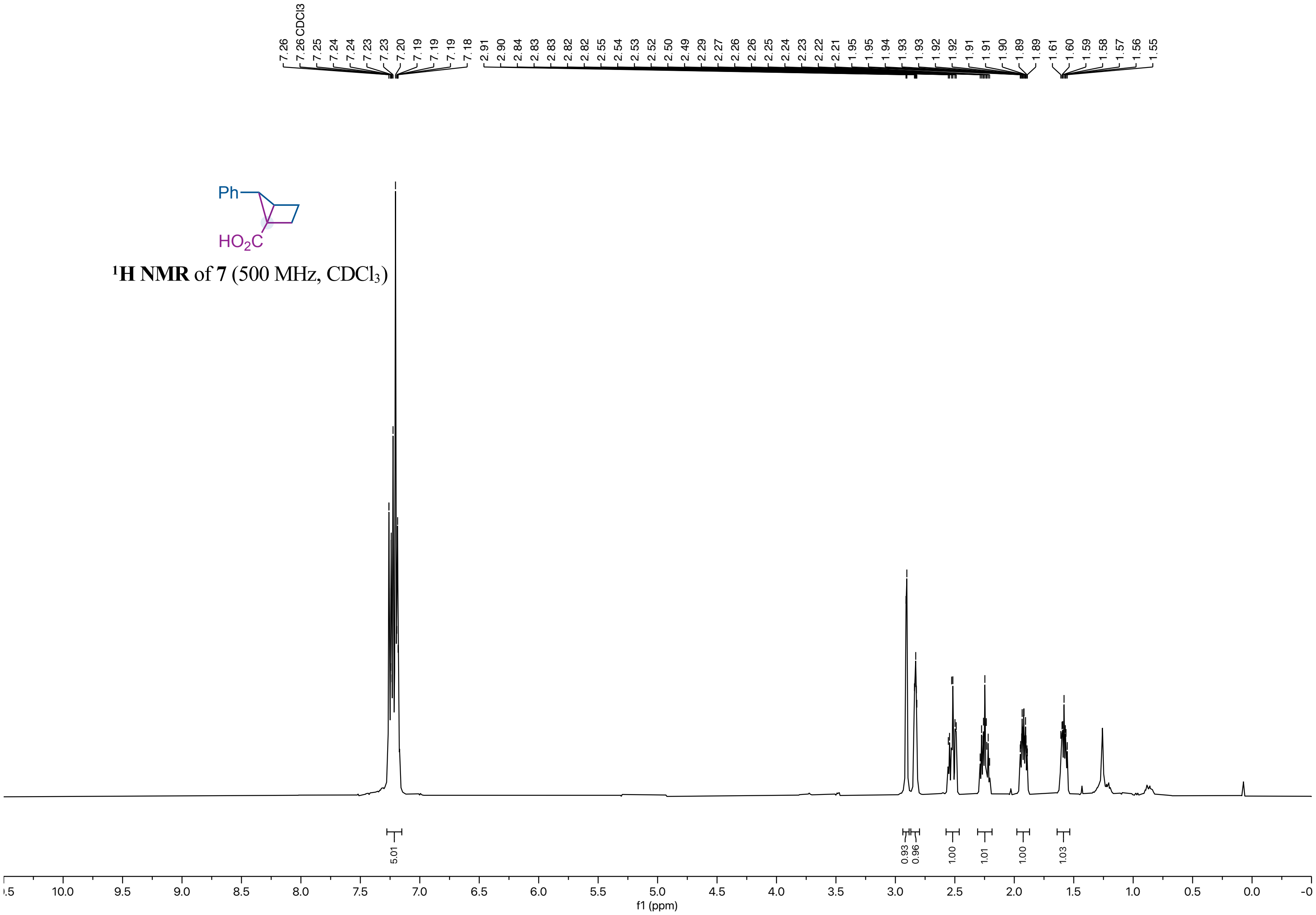

^{1}H NMR of **7** (500 MHz, $CDCl_3$)

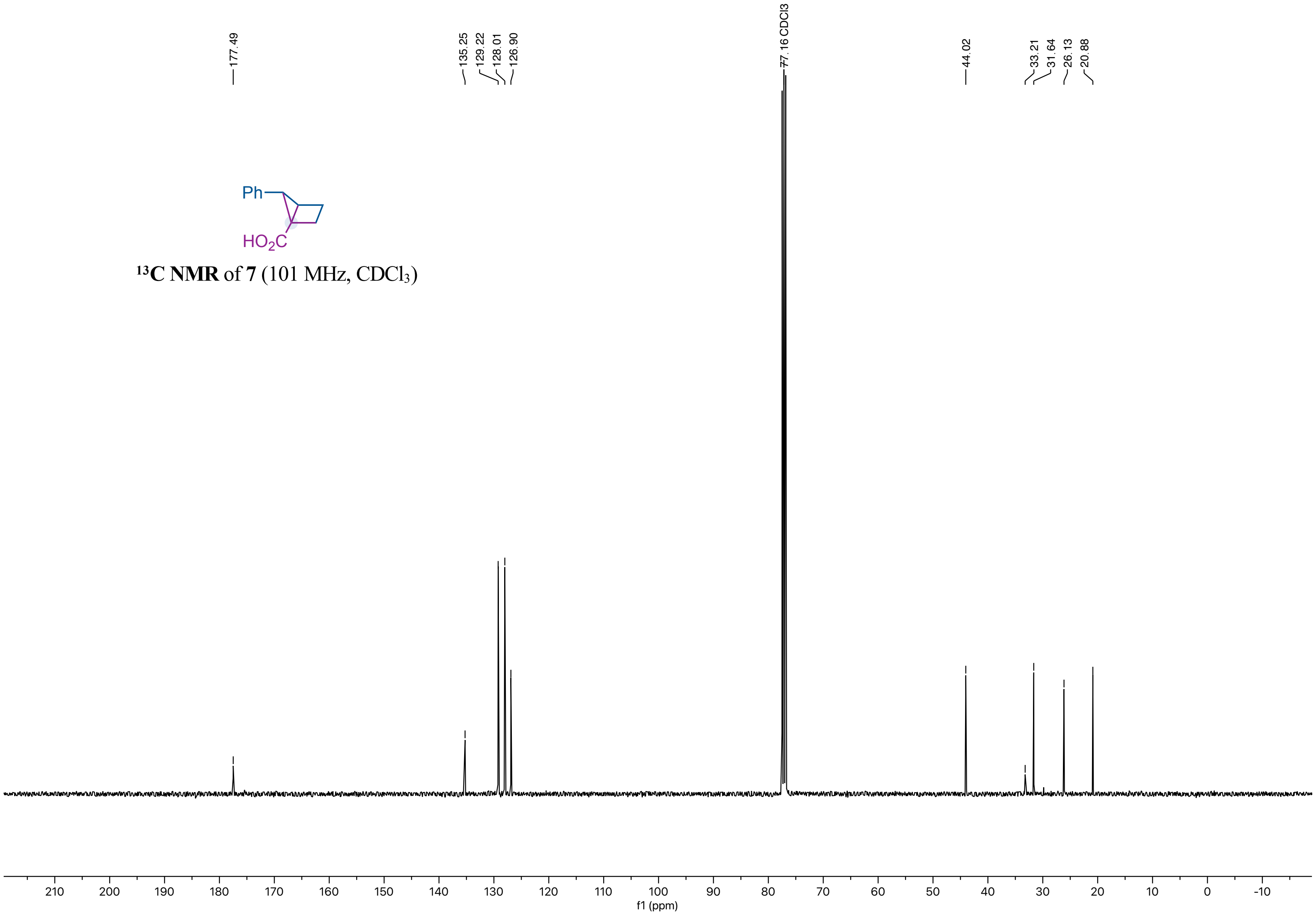

^{13}C NMR of **7** (101 MHz, $CDCl_3$)
